# Comprehensive Reinvestigation of Carbodiimide Guanylation: HCl-Initiated Access to Tri- and Tetrasubstituted Guanidines

**DOI:** 10.1021/acsomega.5c12436

**Published:** 2026-04-16

**Authors:** Lukáš Vlk, Karel Pauk, Maksim A. Samsonov, Zdeňka Růžičková, Tomáš Chlupatý, Aleš Růžička

**Affiliations:** † Department of General and Inorganic Chemistry, Faculty of Chemical Technology, 48252 University of Pardubice, Studentská 573, Pardubice CZ-532 10, Czech Republic; ‡ Institute of Organic Chemistry and Technology, Faculty of Chemical Technology, 48252 University of Pardubice, Studentská 573, Pardubice CZ-532 10, Czech Republic

## Abstract

Guanidines are well-known π-electron-conjugated organic bases used widely in synthesis as well as in industry, with more than 150 years of history. Hence, a plethora of synthetic approaches leading to the formation of the central N_3_C motif has been published, from conventional methods to more sophisticated catalysts. Despite this, some substrates are still not easily obtainable, and the reported procedures lack simplicity and universality. Here, procedures yielding guanidines from carbodiimides and various amines are provided. Thermally conducted reactions of aliphatic amines and carbodiimides led to guanidines, but efforts to extend this method to anilines failed, even with basic or some acidic catalysts. However, when HCl was added to the reaction media at >80 °C, guanidine products were successfully prepared. Thorough mechanistic investigations revealed the complexity of the guanylation, including several proton transfers, the unconventional switch of the reaction mechanism to electrophilic addition, and the regeneration of the catalytically active species. The process was optimized and applicable to a series of various substrates with the use of substoichiometric or even catalytic amounts of HCl. Guanidine structures, the guanylation mechanism, and prototropic tautomerism of aryl-substituted guanidines in solution were investigated by scXRD, NMR spectroscopy, and DFT calculations. An optimized, easy, cheap, and high-yield metal-free procedure catalyzed by HCl was described.

## Graphical abstract

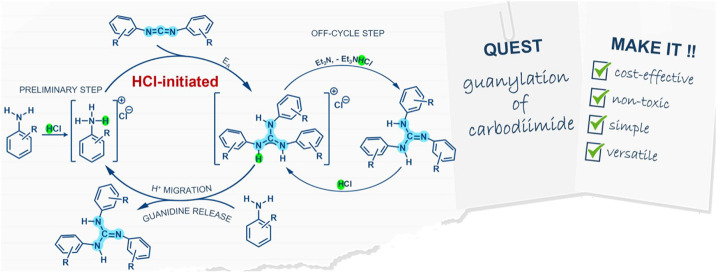

## Introduction

Guanidines are considered organic superbases due to the n−π conjugation of the central N_3_C part and a push–pull effect,^1,2^ with the imino nitrogen being the most basic moiety of the molecule.^2^ The spatial arrangement and characteristics of the *N*-atoms surrounding the central carbon atom of the N_3_C unit provide significant configurability and tunability, which are influenced by steric and electronic effects. In their neutral form, they are useful in organic synthesis or as (non)­nucleophilic catalysts,^1,3–9^ as Barton’s bases resistant to alkylation,^4,5,10^ and have been associated with a range of biological activities, which is linked to the presence of the guanidine moiety in many natural products.^11,12^ Also, guanidines have been utilized in the pharmaceutical^13^ and food and petrochemical industries.^1^ On the other hand, guanidinium cations show exceptional stability due to effective charge delocalization (Y-aromaticity),^14^ the robustness of the cation, and the ability to form hydrogen bonds, and they have been used as building blocks in the design of many organic and semiorganic materials.^15^ Neutral and anionic forms have been widely used as ligands for the stabilization of various metals/elements in different oxidation states.^16–20^

Two general routes exist for guanidine synthesis. Classical methods, using the nucleophilicity/basicity of amines, employ guanylation agents, such as thioureas, isothioureas, aminoiminomethanesulfonic acids, cyanamides, and carbodiimides, and often require activated substrates,^21,22^ increased temperature, toxic or difficult-to-achieve catalysts that are nontolerant to functional groups, and/or offer low yields (Figure 1).^16,21–24^ Thiophilic metal salts like Hg­(II), Cu­(II), or Sc­(III) acetates or chlorides are the most common.^25,26^ In the case of carbodiimides, the guanylation of aliphatic amines is feasible when only thermally conducted, but this guanylation protocol with aromatic amines is insufficient unless initiated or activated (Figure 1) by a usually stoichiometric amount of an obscure/toxic initiator.

**Figure 1:**
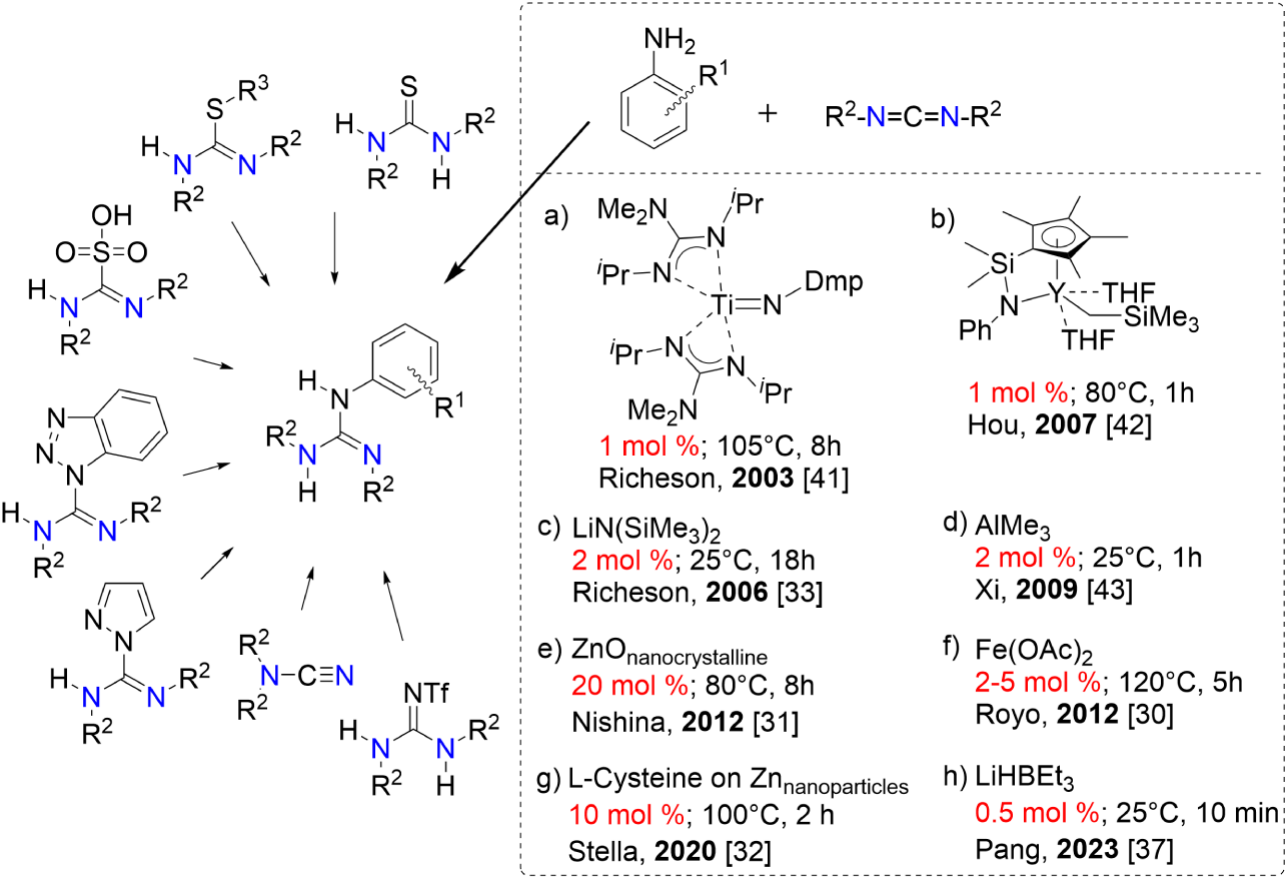
Overview of classical approaches for the guanylation reactions of amines. The guanylation protocol of carbodiimide and aromatic amines using selected catalysts is highlighted in the box. Appropriate references are given in square brackets.

In contrast, the modern route using the catalyzed carbodiimide–amine system offers accessibility to plenty of substituted carbodiimides and overcomes the classical method’s obstacles (Figure 1–highlighted box). These methodologies can sometimes bring challenges in terms of price, air instability, availability of the catalysts, together with respect for environmental aspects. To illustrate, guanylation of aniline with CDI^*i*Pr^ under mild conditions with low loading of half-sandwich complexes of Y, Lu, Er (and others) as catalysts achieved high to almost quantitative conversion.^27^ The reaction of similar substrates catalyzed by the less active dinuclear Ti­(IV) amido complex required longer heating.^28^ If a high-spin NHC-containing Fe­(II) imido complex is used at 5 mol % for the guanylation of aliphatic carbodiimides with a series of anilines, conversions surpass 80% at room temperature after 15 h.^29^ Some metal oxo-species, such as Fe­(II) acetate,^30^ ZnO nanoparticles,^31^ or Zn–S–cysteine nanoparticles,^32^ exhibited lower efficiency. In the field of main group element complexes (Li, Mg, Al, B), similar behavior to transition metal-based catalysts was observed.^33–37^ Guanylations of diisopropylcarbodiimide with selected functionalized anilines, such as 2-bromoaniline or *p*-anisidine, initiated by tris­(pentafluorophenyl)­borane,^38^ also revealed good effectiveness for activated substrates. SnCl_4_ was used to catalyze the annulation of 2-aminobenzonitrile and carbodiimides recently.^39^ During the completion of this work, the guanylation of aliphatic amines, anilines, (sulfon)­amides, ureas, and carbamates by guanidinium salt (HATU), leading to pentasubstituted guanidines, was described.^40–43^

As illustrated in Figure 1, there is a plethora of excellent catalytic systems for the guanylation of carbodiimide described in the literature that are able to achieve high conversion at mild conditions or using relatively cheap, commercially available chemicals (for example, Me_3_Al). However, none of these systems reaches all the characteristics of an all-round/universal catalyst, and the protocols suffer from drawbacks such as the use of a (sometimes toxic) metal or limitations in scope. The most effective catalysts are usually unavailable and sensitive to moisture, while the air-stable species require much higher loading due to lower efficiency (ZnO nanoparticles). This work has the ambition to fulfill the requirements of a simple synthesis of structurally and electronically flexible guanidines while making the synthetic protocol cheap, green, unified, substrate-universal, and atom-efficient.

## Results and Discussion

Guanidines **1**–**17** (Scheme 1) were prepared by a classical guanylation approach^21^ via noncatalyzed thermal nucleophilic addition of an amine to the polarized cumulated NCN double bonds of the carbodiimide moiety in commercial-grade toluene at 100 °C for day(s) (dependent on the nature of the amine and monitored by ^1^H NMR spectroscopy)—**Method A** (for a list of compounds, see Figure S1). Various aliphatic amines and diamines (including also (hetero)­aromatic moieties) and *N*,*N*’-disubstituted carbodiimides (^*i*^Pr, ^*p*^tol, Dipp) gave appropriate guanidines of analytical purity after recrystallization/distillation in high to excellent yields. In the case of guanidine **13**, an aromatic amine was used as the starting material, which can be classified more as an aliphatic amine in this guanylation protocol because of the activation of the NH_2_ group through an intramolecular hydrogen bond to the NMe_2_ group. This subsequently results in enhanced nucleophilicity and increased basicity. Attempts to prepare the monoguanidine analogue of **16** (1:1 ratio product) ended in an equimolar mixture of mono- and bis­(guanidine) due to a competing reaction on both amino groups.

**Scheme 1:**
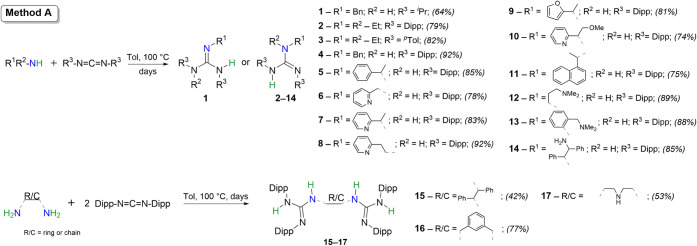
Synthesis of Guanidines **1**–**17** via Noncatalyzed Thermal Guanylation of Carbodiimides (Dipp = 2,6-Diisopropylphenyl)-^a^

a Isolated yields of target guanidines are given in parentheses.

To broaden the scope to anilines, a protocol effective for aliphatic amines failed. No guanidine products were detected by NMR spectroscopy, even after prolonged heating of the mixture of CDIs and anilines. Similarly, acid or base catalysis (NaOH or CH_3_COOH) or the introduction of donor functional groups to the aniline did not yield successful results. Surprisingly, the addition of a few drops of aqueous HCl (the same result was obtained with an anhydrous HCl/CPME solution under Ar) at 100 °C in toluene caused the formation of guanidine (**21**) along with the corresponding guanidinium. No conversion was detected by NMR spectroscopy below 80 °C. The reaction mixture was further treated with a base (Et_3_N or ^*t*^BuOK), and only guanidine was obtained, which inspired further investigation of various substrates using a defined amount of HCl.

First trials conducted for reactions of *o*- or *p*-anisidine with CDI^Dipp^ and a stoichiometric amount of HCl (Scheme 2—**Method B**) yielded the conjugated acids of **21** and **23** (denoted as **21**·HCl and **23**·HCl) quantitatively after 1 h. However, these reactions ended in the guanidinium form, which requires an additional neutralization step and thus may, in some cases, be complex. This approach was extended to other primary aromatic amines (*o*-/*m*-/*p*-MeO, *p*-F, *o*-/*m*-/*p*-NO_2_) and substituted CDIs (Cy, Dipp, Dmp), resulting in guanidines **18**-**28** (Scheme 2 and Figure S2 in ESI) after 12 h of heating in toluene, followed by *in situ* neutralization by Et_3_N (or ^*t*^BuOK in THF for isolated **19**·HCl). During the review process of this article, the most challenging system was identified by one of the reviewers. As a proof-of-concept evaluation, one tetrasubstituted tetraarylguanidine **29** was successfully prepared under an identical protocol. To prevent the easy oxidation of the aliphatic CDI under acidic conditions, the guanylations to **18** and **19** were performed under an inert argon atmosphere. Unexpectedly, during the synthesis of **25**, which originated from an aromatic CDI and *o*-nitroaniline, a significant amount of urea (as well as its protonated form) was observed, even when the reaction was conducted under argon. A negligible increase in the yield of **25** (21% to 28%) was achieved when the amount of HCl was reduced to 50 mol %.

**Scheme 2:**
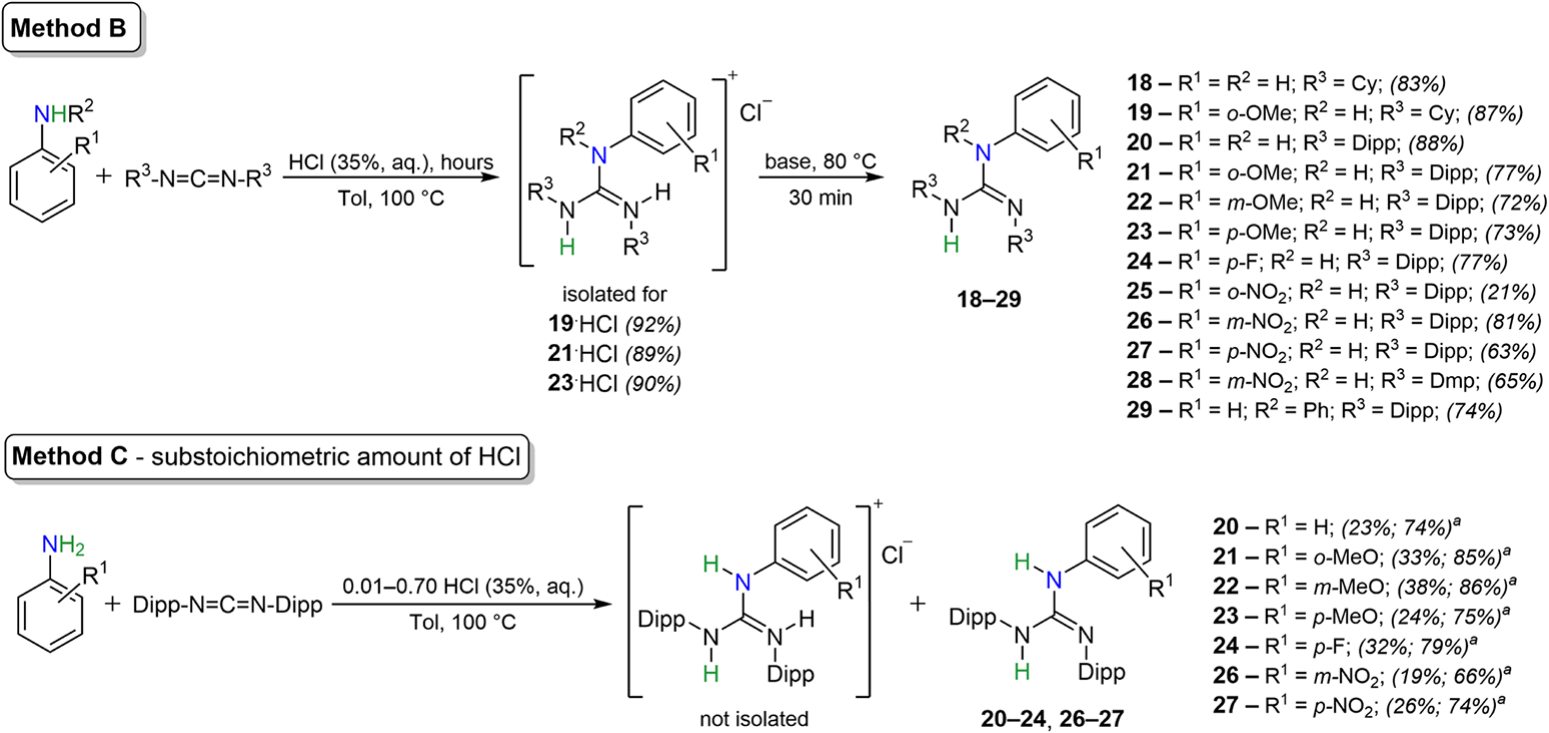
Synthesis of Guanidines **18**–**29** (and Guanidiniums **19**·HCl, **21**·HCl, and **23**·HCl) via the HCl-Initiated Guanylation of Carbodiimides[Fn sch2-fn2]

i Base–Et_3_N (^*t*^BuOK for **19**·HCl). Isolated yields of target guanidines (**Method B**) and NMR yields after 2 days using 50 mol % of HCl (**Method C**) are given in parentheses, where the first number^*a*^ corresponds to free guanidine and the second to the sum of free guanidine and its guanidinium

During the guanylation of the aniline series, it became evident that HCl plays a pivotal role in the reaction mechanism, indicating the greater complexity of the process. To elucidate this mechanism further, the impact of varying substoichiometric/catalytic amounts of HCl on the guanylation of aromatic substrates was investigated (Scheme 2—**Method C**). Specifically, the synthesis of **21** (also present as **21**·HCl) from *o*-anisidine and CDI^Dipp^ was examined using 1–70 mol % of HCl over time (Figures 2 and S9 in ESI). The yields were quantified as a sum of guanidine (**21**) and its guanidinium (**21**·HCl), or only **21,** over the duration of the reaction, while the amount of guanidinium remains constant throughout the whole process and is equal to the amount of HCl used (Figure 2). It should be considered that prolonged heating can lead to the oxidation/degradation of substrates. The findings from the guanylation protocol suggest that using a larger amount of HCl leads to an almost complete reaction (**21**·HCl predominates in the mixtures) within 3 days. Moreover, when a small initiator concentration (under 10 mol %) is used, there is still a significant increase in the yield of **21** due to a lower concentration of **21**·HCl. For instance, the reaction with 50 mol % of HCl produced 29% of **21** after 1 day, with the yield rising to only 40% by day 7. In contrast, when only 1 mol % of HCl was utilized, the yield increased from 19% to 44%. To complete this hypothesis, the catalytic efficiency was screened using 1–100 mol % of HCl loading, plotted as yield against catalyst concentration (Figure 3), showing maximum yield of **21** at 25 mol % of HCl.

**Figure 2:**
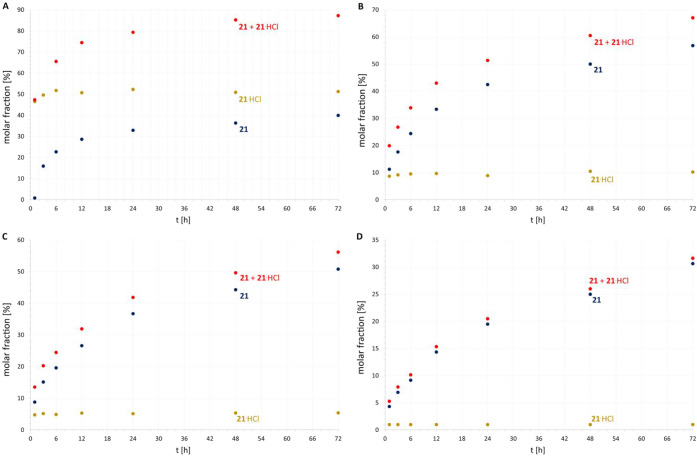
NMR yields of guanylation reactions of CDI^Dipp^ by *o*-anisidine to **21** (blue dots), **21**·HCl (gold dots), or to the cumulative yield of **21** and **21**·HCl (red dots) using: **A**) 50 mol % of HCl; **B**) 10 mol % of HCl; **C**) 5 mol % of HCl; and **D**) 1 mol % of HCl after 3 days.

**Figure 3:**
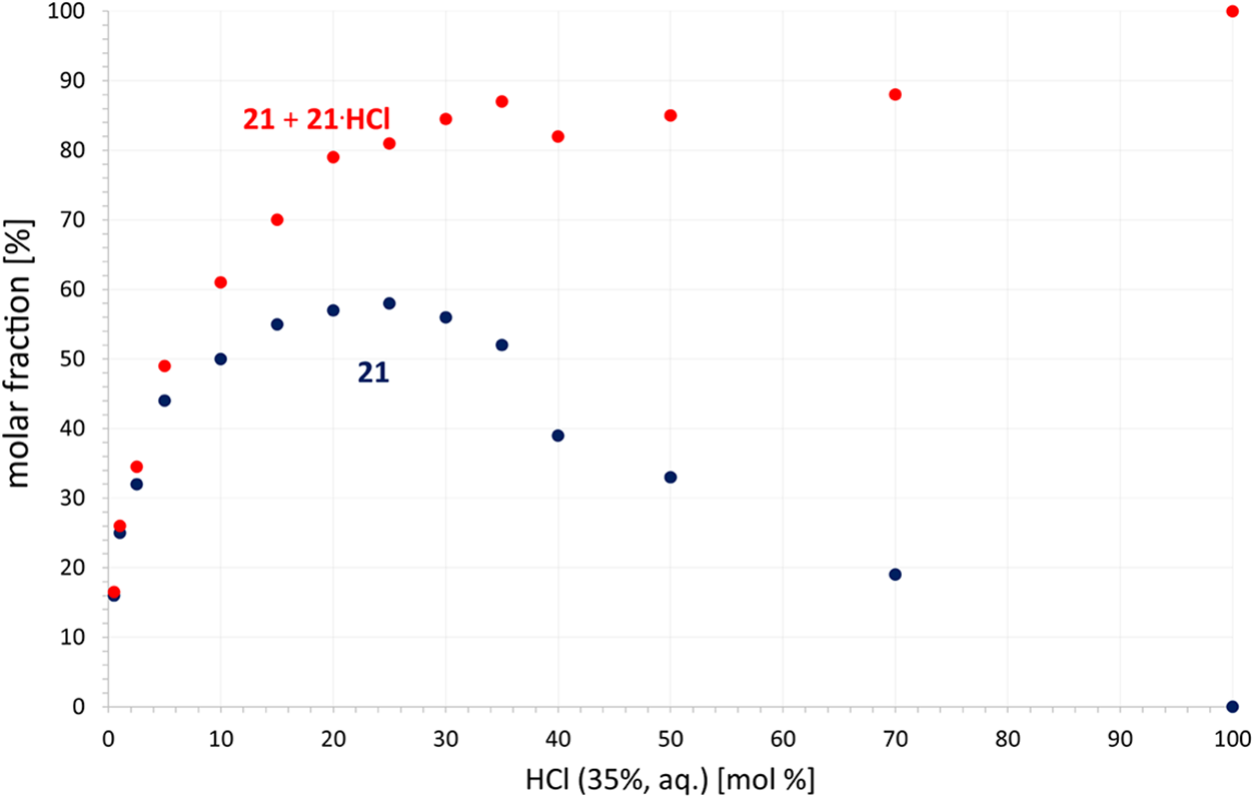
Optimization of the HCl amount for the preparation of **21** (blue dots) or the sum of **21** and **21**·HCl (red dots) after 2 days.

Expanding the portfolio of that method, it was applied to other anilines with the addition of 10 and 50 mol % HCl (Scheme 2—**Method C**), covering various functional groups in different aromatic ring positions, including aniline (**20**—Figure S8), anisidines (**22**–**23**—Figures S10–S11), *p*-fluoroaniline (**24**—Figure S11), and nitroanilines (**26**–**27**—Figures S12–S13).

The overall basicity of the respective aniline is a contribution of mesomeric effects, and induction effects are the determining factors on which the reaction course depends. For the catalytic guanylation, the essential prerequisite is anilines’ high proton affinity to form a conjugated pair with HCl (anilinium). This is evident in the case of the two *meta*-substituted anilines (better-performing *m*-OCH_3_ going to **22** vs *m*-NO_2_ going to **26**), also compared to unsubstituted aniline for the formation of **20** (see SI, Table S2). Another comparison can be drawn between *o*-OCH_3_ (**21**) and *m*-OCH_3_ (**22**), where the yields to **21** are *ca* 20% higher. This was more pronounced in comparative experiments with 10% HCl, where yields were 15% for **26**, 31% for **22**, and 42% for **21** after 1 day (Table S2).

All isolated guanidines **1**–**29**, covering all four typological combinations of starting aliphatic (R^Alif^)/aromatic (R^Ar^) (Figure 4) amines and carbodiimides, and guanidiniums **19**·HCl, **21**·HCl, and **23**·HCl were completely characterized by multinuclear NMR, MALDI-MS, scXRD (except for **3**, **9**, **11,** and **16**; Figures S16–S43 in ESI) techniques, and elemental analysis (**20**–**29**, **21**·HCl and **23**·HCl) (for an overview of the structural characterization methods used, see Figures S1–S2 in ESI). The most significant phenomena used for monitoring the course of the reaction were the hydrogen shift(s) from the starting amino group to nitrogen atom(s) originating from the CDI moiety (change of ^1^H NMR spectral pattern or chemical shift) and the related downfield shift of the central carbon atom (Ar_q_^Gua^) in ^13^C NMR spectra. In nearly solvent-independent ^1^H NMR spectra, the N*H* hydrogen atoms are chemically nonequivalent (except for **1**, **18**, and **19**). For **13** and **25**, one N*H* (originating from aniline) is downfield-shifted because of an intramolecular H-bond. The deshielding of Ar_q_^Gua^ was observed in ^13^C spectra for all prepared guanidines, with typical values of ∼150 ppm for tri-/tetrasubstituted di-/trialkylguanidines **1**–**3**, **18**, **19,** and **29** (measured at 240 K in Tol-d_8_), while for trisubstituted di/triarylguanidines **4**–**17** and **20**–**28**, the values range between 144–147 ppm. To complete the structural description, the C–N distances of the N_3_C unit show partial π-electron delocalization for all guanidines (C^Gua^–NH: 1.36–1.39 Å and C^Gua^–N: ∼1.30 Å) (Figure 4), with slight differences for tetraarylguanidine **29** (Figure S43).

**Figure 4:**
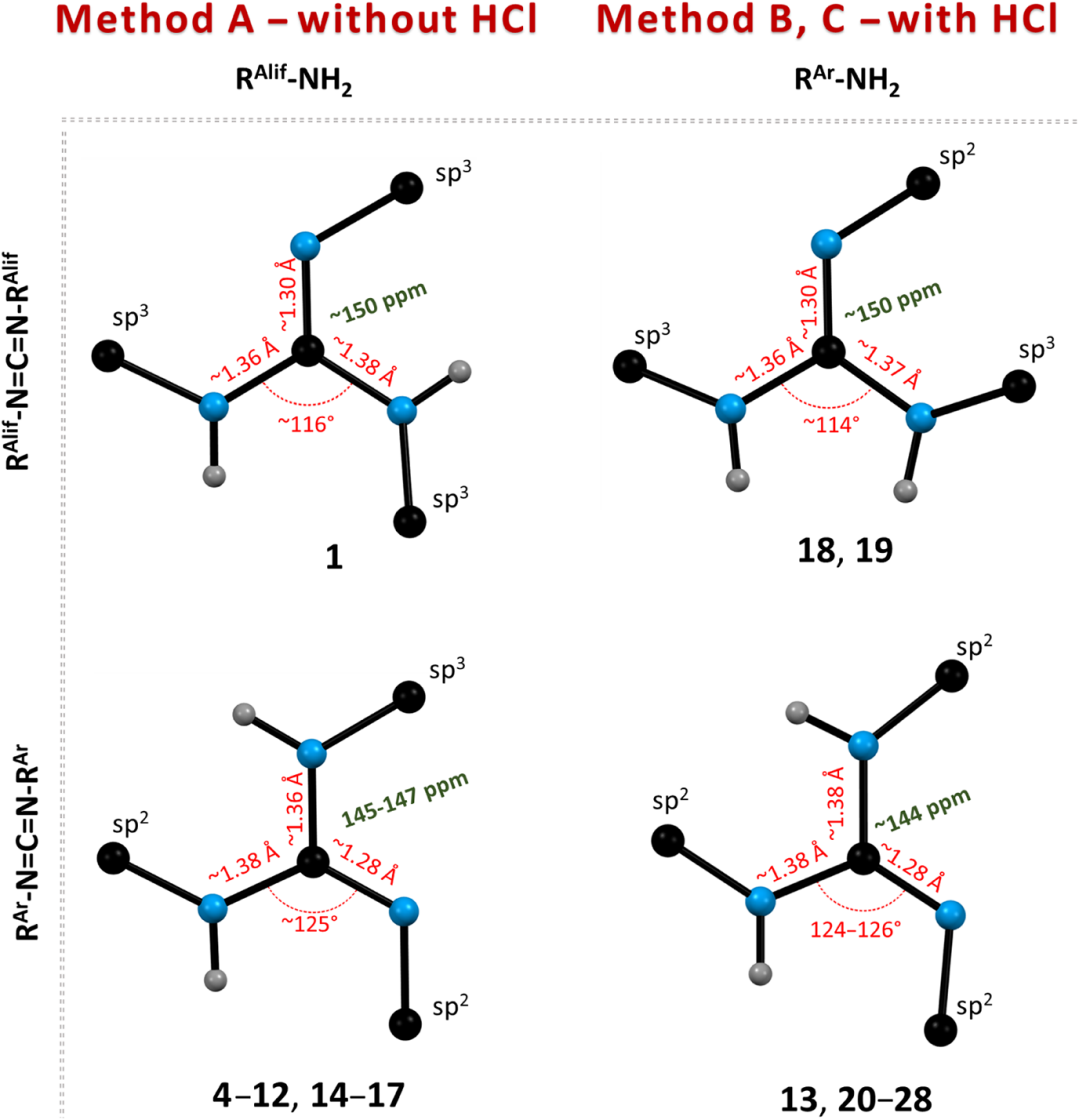
Illustration of selected structural parameters of guanidines, encompassing all four typological combinations. Blue spheres represent N atoms, black for C atoms, and gray for H-atoms. The ^13^C NMR chemical shifts of Ar_q_^Gua^ are shown in green.

In the NMR spectra of “aromatic” guanidines **20**–**27** and **29**, originating from the anilines and aromatic CDI^Dipp^, a second minor set of signals with similar chemical shift values was observed. This phenomenon was more pronounced in THF-d_8_ (for **29,** observed in Tol-d_8_ at 240 and 273 K—Figures S70–S71) solution and was further investigated for an extreme example of **26** by VT-NMR spectroscopy (Figures S4–S7 in ESI) and DFT calculations (Figure 6). Eighteen mol % of the minor form was detected at room temperature in THF-d_8_, contrary to 9 mol % in C_6_D_6_, while only negligible temperature dependency was observed by VT-NMR measurements in both solvents (see Figures S5–S7). However, a significant change of the relative populations of both species was observed in Tol-d_8_ in the range of 183–373 K (Figures 5 and S7 in ESI).

**Figure 5:**
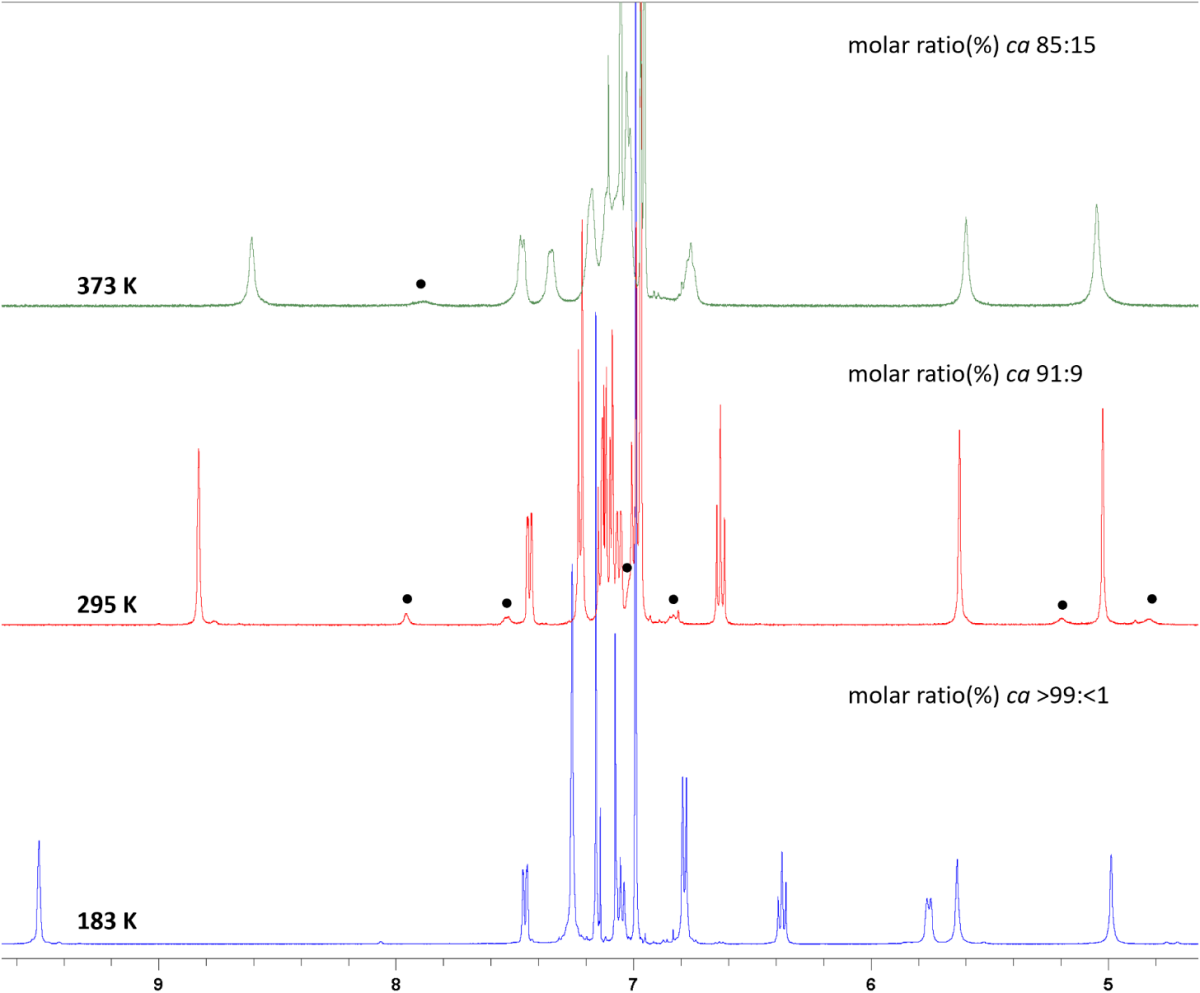
Detail of VT ^1^H NMR spectra of **26** in Tol-d_8_ at 183–373 K. Signals of the minor tautomer are marked with black dots.

This was initially attributed to prototropic guanidine tautomerism/isomerism. The structures of four reliable conformers/isomers, constructed from both ab initio or scXRD/optimized geometries, were prepared. For these species, the NMR shielding constants for ^13^C nuclei were calculated by the GIAO method (B3LYP/6–311+g­(d,p)/D3/CPCM­(THF)).^44^ The most probable isomers/conformers of **26** were selected based on the direct comparison of the structures, Gibbs’ free energies (Figure 6),^45^ and calculated (Table S1, Figure S3) vs measured ^13^C NMR parameters in THF-d_8_ solution.

**Figure 6:**
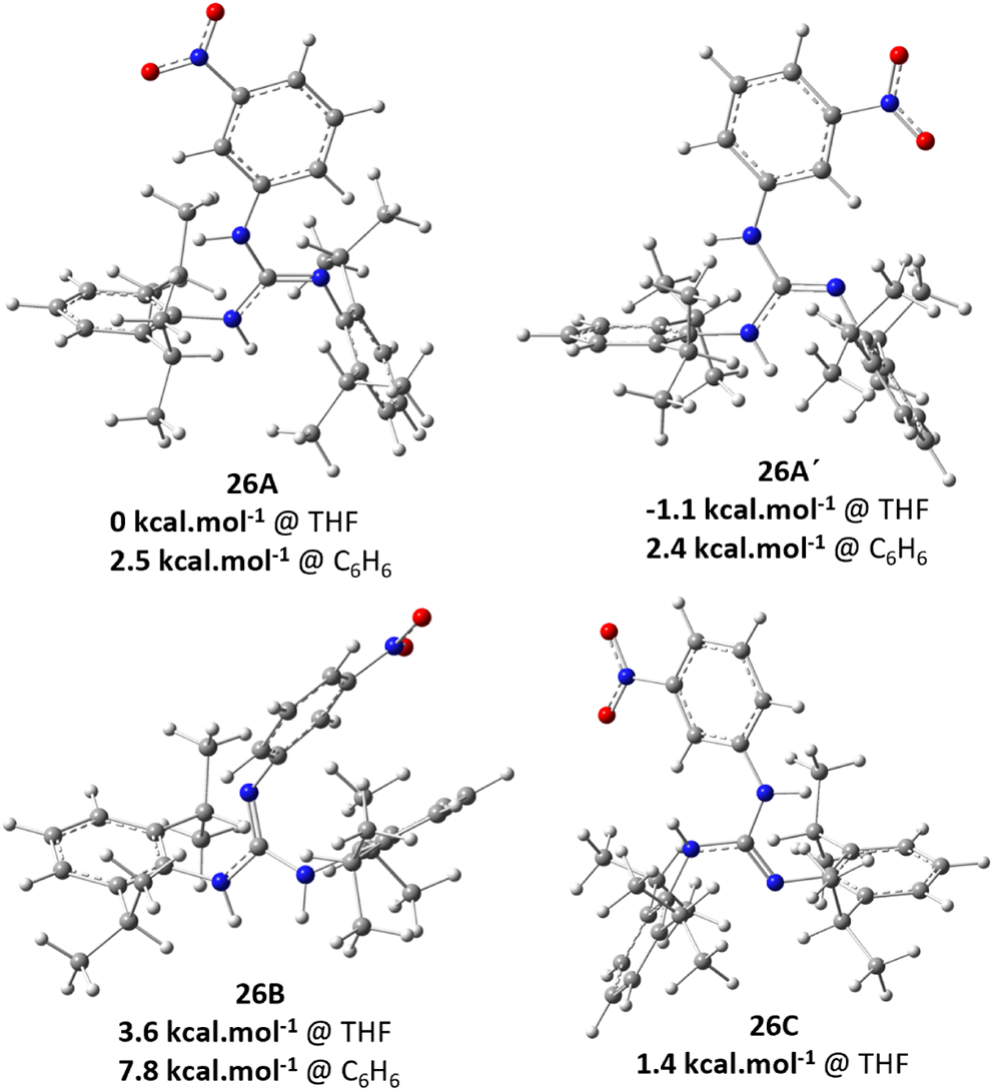
Optimized structures of possible tautomers/isomers of **26,** along with relative Gibbs’ free energies.

Two of the four relevant isomers, specifically the pair of rotamers **26A** and **26A’** (Figure 6), exhibit a minimal energy difference of just 1 kcal·mol^–1^ in THF (virtually identical in C_6_H_6_). Moreover, the rotation barrier from **26A** to **26A’** is only 3.7 kcal·mol^–1^. These isomers are thus indistinguishable in solution by NMR spectroscopy, and their weighted chemical shifts represent the signals of the major isomer of **26**. According to the Boltzmann distribution at room temperature, their respective contributions to the chemical shift values are 15.4% for **26A** and 84.6% for **26A’** (Table S1, Figure S3). For isomer **26B**, analysis of the correlation between the measured signals in the ^13^C NMR spectra and the calculated chemical shieldings indicates that it exists as a minor form in solution NMR (Table S1, Figure S3). In contrast, isomer **26C**, despite being energetically similar, was not detected in either the solution or the solid state.

To provide a deeper understanding of the guanylation mechanism, we conducted a detailed investigation of the *o*-anisidine-CDI^Dipp^-HCl system using NMR and DFT techniques at the B3LYP/cc-pVTZ level of theory.^45^ It clearly showed that the process begins with the immediate protonation of the aniline to **INT-1** (Figure 7; rather than a less basic CDI^Dipp^). This was also evidenced by the fast proton transfer from individually prepared H^+^CDI^Dipp^ and *o*-anisidine.

**Figure 7:**
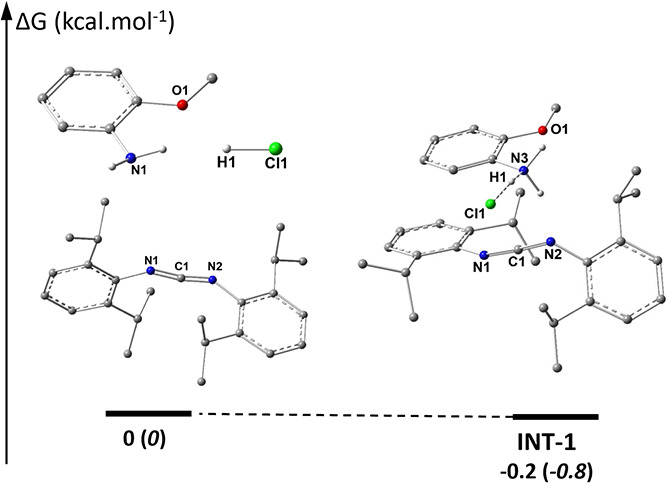
DFT-estimated Gibbs free energy profile (kcal·mol^–1^) for the calculated interaction between *o*-anisidine (values for *p*-anisidine are given in parentheses), CDI^Dipp^, and the HCl molecule.

The thermodynamically lowest **21**·HCl species (−20.31 kcal·mol^–1^) is produced through several consecutive steps (Figure 8 and Figure S14 in ESI), virtually connected by several H-bond interactions. From **INT-1**, H1 from the amino group connects the aniline to the CDI of **INT-2**, thus bringing the N3 and C1 atoms into proximity. The energetically limiting step is **TS-2** (7.75 kcal·mol^–1^), where the N3–C1 bond is formed, followed by an intramolecular proton transfer of H2 (via **INT-1** to **TS-3**). The proposed slow migration of the proton from **21**·HCl to the next starting *o*-anisidine molecule (**TS-4**, ΔΔ*G*: 18.67 kcal·mol^–1^) allows the initiation of a new catalytic cycle (blue lines in profile). The guanidine **21** is released and accumulates over time, which is compensated for by the fast generation of another new **21**·HCl. An alternative pathway, which is more thermodynamically favored, is the neutralization of **21**·HCl by Et_3_N addition—**TS-4́** (the red trace in Figure 4). To explore the influence of a sterically less demanding substrate of the same basicity, the same calculations were performed for *p*-anisidine to provide **23** (Figure 8 in parentheses), with negligible energy changes. When virtually replacing the proton within the mechanism for a metal ion, each individual reaction step would be similar. As expected, calculations of all four model systems, covering combinations of CDI-amine (aliphatic or aromatic, Figure S15) showed thermodynamically favorable catalytic processes in the series of aliphatic amines. Based on these findings, additional guanylation experiments, mimicking a substoichiometric amount of acid catalyst, were successfully initiated by 50 mol % *o*-anisidinium hydrochloride or guanidinium **21**·HCl (Scheme S1 in ESI). To be honest, there are decades-old papers reporting a few examples of ammonium salts and their interactions with carbodiimide (amine and carbodiimide in an acidic environment), but the results were misunderstood and useful details overlooked.^46–53^ Taking these reports into account, a stoichiometric reaction of *o*-anisidinium and **21** in toluene was performed, yielding exclusively **21**·HCl and *o*-anisidine after 1 h, even at room temperature (Scheme S1 in ESI).

**Figure 8:**
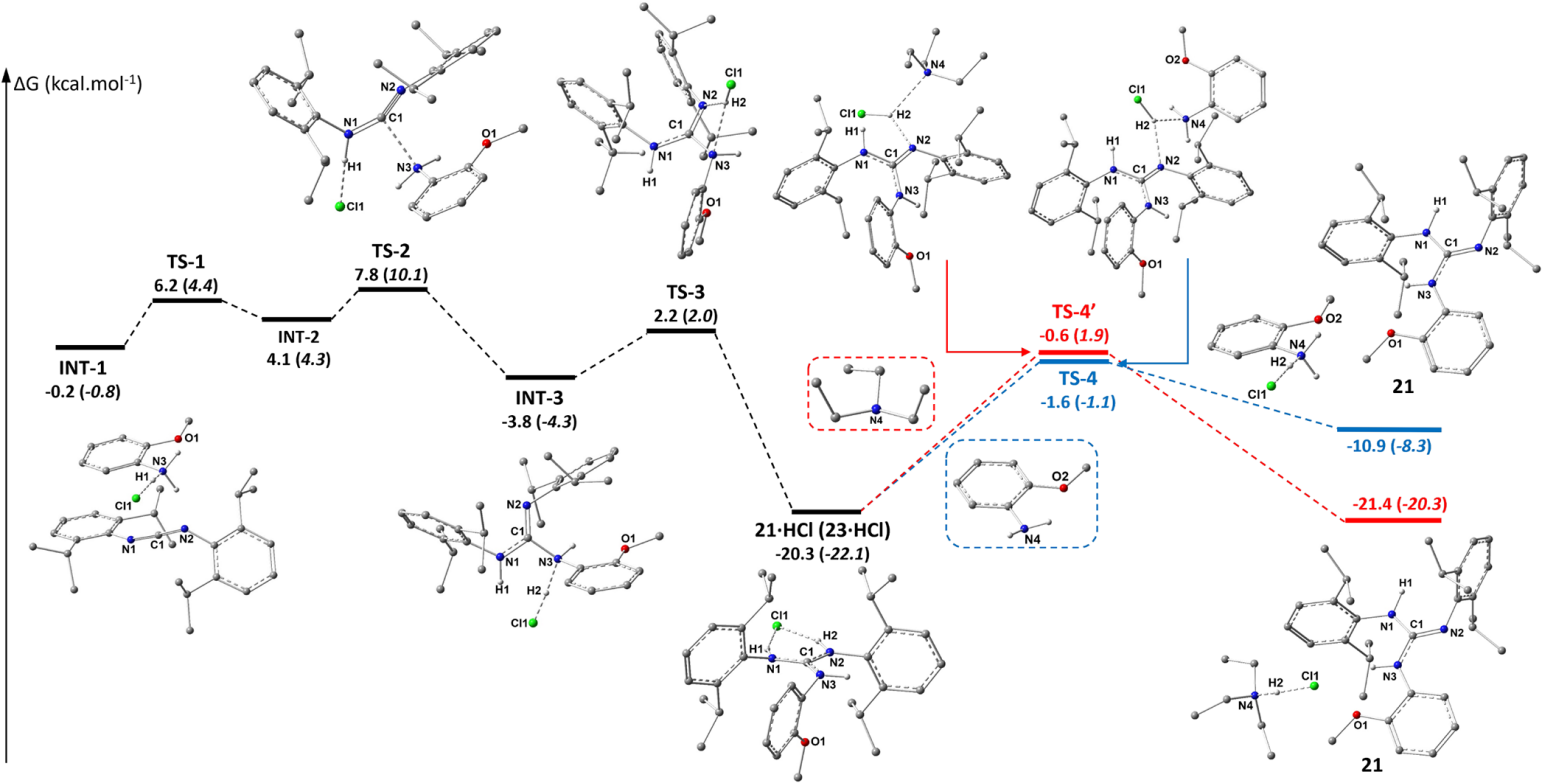
DFT-estimated Gibbs free energy profile (kcal·mol^–1^) for **21** (**23** in italics in parentheses). The preliminary step to **INT-1** (see Figure 7 above), and structures describing **TS-1** and **INT-2** are omitted for clarity. Full energy profile is available in Figure S14 in the ESI.

## Conclusions

There is no comprehensive guanylation approach covering a wide array of substrates with respect to functional groups, while maintaining low economic and accessibility requirements. It is reasonable to assert that the literature describes numerous catalytic carbodiimide guanylation systems that exhibit enhanced conversion values and reduced reaction times; however, these systems are often constrained by limitations related to the steric accessibility or reactivity of substrates, as well as the use of toxic, sensitive, or difficult-to-obtain catalysts. In contrast, this study presents a cost-effective, nontoxic, and universally applicable alternative for the synthesis of tri- and tetrasubstituted guanidines, employing HCl as an initiator. Concretely, the suggested guanylation mechanism (to **21**) was supported by extended model experiments and theoretical calculations. The rate-determining step, connected to the highest computed activation barrier for **TS-4** (release of **21**, ΔΔ*G* = 18.67 kcal·mol^–1^), corresponds to the proton transfer from the earlier formed guanidinium to an adjacent molecule of aniline. The reaction time can be drastically shortened by employing a higher amount of HCl, followed by the addition of a stronger base with subsequent workup.

## Materials and Methods

All solvents and chemical reagents were purchased from commercial sources and used without further purification. Some synthetic procedures were performed using standard Schlenk techniques under an inert argon atmosphere (99.999%) (the inert gas was passed through the oxygen/moisture trap Supelco before entering the vacuum/inert line), and solvents were dried with the help of the solvent purification system PureSolv MD 7 supplied by Innovative Technology, Inc., degassed, and then stored under an argon atmosphere over a potassium or sodium mirror, if needed. Single crystals suitable for X-ray analysis were obtained from the corresponding saturated solutions of products in organic solvent(s) cooled to 7 or −30 °C or by slow evaporation at room temperature. Deuterated solvents for NMR spectroscopy, if needed, were distilled, degassed, and stored over a K or Na mirror under an argon atmosphere.

Elemental analysis (C, H, N, Cl) was performed on an automatic microanalyser Flash 2000 Organic Elemental Analyzer. Mass spectrometry with high resolution was determined by the “dried droplet” method using a MALDI LTQ Orbitrap XL mass spectrometer (Thermo Fisher Scientific) equipped with a nitrogen UV laser (337 nm, 60 Hz). Spectra were measured in positive ion mode and in regular mass extent with a resolution of 100,000 at a mass-to-charge ratio (*m*/*z*) of 400, with 2,5-dihydrobenzoic acid (DHB) used as the matrix. Mass spectrum of **29** was performed on Vanquish HPLC/ISQ system from Thermo Scientific equipped with a quaternary pump (VC-P20-A), split sampler (VC-A12-A), column compartment (VC-C10-A), diode array detector (VC-D11-A), and mass spectrometer (ISQ ICMS Family).

NMR spectra were recorded from solutions of appropriate compounds in deuterated solvent(s) on a Bruker Avance 500 spectrometer (equipped with a Z-gradient 5 mm Prodigy cryoprobe) at frequencies for ^1^H (500.13 MHz), ^13^C­{^1^H} (125.76 MHz), and ^15^N (50.66 MHz) or on a Bruker UltraShield 400 spectrometer at frequencies for ^1^H (400.13 MHz) and ^13^C­{^1^H} (100.58 MHz) at 295 K, or in some cases, at various temperatures. Solutions were obtained by dissolving approximately 40 mg of each compound in 0.6 mL of deuterated solvents. Values of ^1^H chemical shifts were calibrated to residual signals of benzene (δ­(^1^H) = 7.16), THF (δ­(^1^H) = 1.73), toluene (δ­(^1^H) = 2.09), or DMSO (δ­(^1^H) = 2.50). Values of 13C chemical shifts were calibrated to signals of THF (δ­(13C) = 67.6), benzene (δ­(13C) = 128.4), toluene (δ­(13C) = 20.4), or DMSO (δ­(13C) = 20.4), and ^15^N to external nitromethane (δ­(^15^N) = 0.0). All 13C NMR spectra were measured using a standard proton-decoupled experiment, and CH and CH_3_ vs C and CH_2_ were differentiated with the help of the APT method.^54^ Determination of signals of chemically nonequivalent protons, carbon, and nitrogen atoms in the NMR spectra was supported by ^1^H,^1^H–COSY, ^1^H,^1^H-NOESY, ^1^H,^13^C-HSQC or/and ^1^H,^13^C-HMBC techniques.

### General Methods for Guanylation Reactions (**1–29**)

#### Method A: Guanylation without HCl (**1–17**)

To a round-bottom flask equipped with a condenser, one equivalent of colorless *N*,*N*′-diisopropylcarbodiimide, 1,3-di-*p*-tolylcarbodiimide, or *N*,*N*’-bis­(2,6-diisopropylphenyl)­carbodiimide with one (or half) equivalent of the appropriate aliphatic (except **13**) amine/diamine was dissolved in toluene. The reaction mixture was refluxed for several days, depending on the type of amine (**2**–**19** days). After that, the toluene was evaporated under vacuum, and the crude products were purified by recrystallization from organic solvent(s) or by distillation (for **1**).

#### Method B: Guanylation with a Stoichiometric Amount of HCl (**18**–**29**, **19**·HCl, **21**·HCl, and **23**·HCl)

To a round-bottom flask equipped with a condenser, one equivalent of colorless *N,N′*-dicyclohexylcarbodiimide, *N*,*N*’-bis­(2,6-dimethylphenyl)­carbodiimide, or *N*,*N*’-bis­(2,6-diisopropylphenyl)­carbodiimide with one equivalent of an appropriate aromatic amine was dissolved in toluene, followed by the addition of the same equivalent of HCl solution. The reaction mixture became heterogeneous and was heated to 100 °C overnight, with subsequent cooling to room temperature. Appropriate crude guanidinium chlorides were formed.

In the case of **19**·HCl, **21**·HCl, and **23**·HCl, crude products were additionally isolated by filtration and purified by washing/recrystallization from organic solvent. However, for **25**, all solvents from the reaction mixture were evaporated, the crude product (mainly guanidinium) was washed by a large portion of Et_2_O and suspended in hexane.

Then, 1.1 equiv of Et_3_N was added to the mixture (all protonated forms were transformed to their neutral species), which led to the gradual precipitation of a white solid, Et_3_N·HCl, for 30 min at 80 °C (except **19**·HCl—^*t*^BuOK in THF). After that, the prepared guanidines **19**–**28** were filtered off, toluene was evaporated under vacuum, and the crude products were purified by recrystallization from organic solvent(s). Details of the complex purification process for **18** are described below.

#### Method C: Guanylation with a Substoichiometric Amount of HCl (**20**–**24**, **26**–**27**)

To a round-bottom flask equipped with a condenser, colorless *N*,*N*’-bis­(2,6-diisopropylphenyl)­carbodiimide (0.5 g, 1.38 mmol) with one equivalent of the appropriate aromatic amine was dissolved in toluene (30 mL), followed by the addition of a substoichiometric amount of HCl solution (35% aqueous solution, ρ = 1.180 g·cm^–3^), which caused the immediate formation of a white precipitate (related to the amount of HCl). The reaction mixture was heated to 100 °C for a certain period, which depended on the degree of conversion monitored by the integration of ^1^H NMR spectra in THF-d_8_ of reaction mixture aliquots over the timeline.

In some cases, Et_3_N (10% excess relative to HCl) was added to the mixture, which led to the gradual precipitation of a white solid, Et_3_N·HCl, in 30 min at 80 °C. All protonated forms were transformed to their neutral species to confirm the amount of formed guanidines, as determined by NMR yield, without further isolation/purification.

### Crystallography

Full sets of diffraction data for **2**, **4**–**8**, **10**, **12**–**14,** and **17** (see Tables S4–S13 and S15) were obtained at 150 K using an Oxford Cryostream low-temperature device on a Nonius KappaCCD diffractometer with MoK_α_ radiation (λ = 0.71073 Å), a graphite monochromator, and the ϕ and χ scan mode. Data reductions were performed with DENZO-SMN.^55^ The absorption was corrected by the multiscan method—SADABS or by integration methods.^56^ Structures were solved by direct methods (Sir92)^57^ and refined by full-matrix least-squares based on *F* ^*2*^ (SHELXL97).^58^

Full sets of diffraction data for **1**, **15**, **18**–**29**, **19**·HCl, **21**·HCl, and **23**·HCl (see Tables S3, S14, and S16–S30) were collected at 150(2)K with a Bruker D8-Venture diffractometer equipped with Cu (Cu/*K*_α_ radiation; λ = 1.54178 Å) or Mo (Mo/*K*_α_ radiation; λ = 0.71073 Å) microfocus X-ray (IμS) sources. A photon CMOS detector and an Oxford Cryosystems cooling device were used for data collection.

The frames were integrated with the Bruker SAINT software package by using a narrow frame algorithm. Data were corrected for absorption effects using the multiscan method (SADABS). The obtained data were treated by XT-version 2014/5 and SHELXL-2017/1 software implemented in the APEX3 v2016.5–0 (Bruker AXS) system.^59^

Hydrogen atoms were mostly localized on a difference Fourier map; however, to ensure uniformity in the treatment of the crystal, all hydrogen atoms were recalculated into idealized positions (riding model) and assigned temperature factors H_iso_(H) = 1.2 U_eq_ (pivot atom) or 1.5U_eq_ (methyl). H atoms in methyl, methylene, moieties, and hydrogen atoms in aromatic rings were placed with C–H distances of 0.96, 0.97, and 0.93 Å, respectively. Hydrogen atoms of N–H groups were refined freely or with fixed distances of 0.92 Å. Disordered parts of isopropyl and coordinated THF molecules in **1** were treated by standard methods.

Crystallographic data for structural analysis have been deposited with the Cambridge Crystallographic Data Center, CCDC 2120564–2120577, 2120580–2120589, 2382648–2382649, 2442574, and 2524367 for **1**–**2**, **4**–**8**, **10**, **12**–**15**, **17**–**29**, **19**·HCl, **21**·HCl, and **23**·HCl. Copies of this information may be obtained free of charge from The Director, CCDC, 12 Union Road, Cambridge CB2 1EY, UK (fax: + 44–1223–336033; e-mail: deposit@ccdc.cam.ac.uk or www: http://www.ccdc.cam.ac.uk).

### DFT Calculations

All calculations were performed using the Gaussian 16 program.^60^ Reaction energy profiles were computed at the B3LYP/cc-pVTZ,^45^ level of theory, incorporating solvation effects through the polarizable continuum model (PCM)^44^ for toluene. Additionally, dispersion corrections were applied using the D3 version of Grimme’s dispersion method.^61^ Frequency analysis at the same level of theory confirmed that all computed structures correspond to minima on the potential energy surface, with transition states exhibiting a single imaginary frequency.

## Supplementary Material













## Data Availability

Complete NMR spectra (DOI: http://10.6084/m9.figshare.28816364) of all prepared compounds.

## Notes

Synthetic procedures, explanation of structures, and tautomerism­(DOCX)
(CIF)
(CIF)
(CIF)
Guanylation mechanism (XYZ)
Complete NMR spectra of all prepared compounds (PDF)
