# Supplementary material for: Comprehensive Reinvestigation of Carbodiimide Guanylation: HCl-Initiated Access to Tri- and Tetrasubstituted Guanidines
Source: ACS Omega. 2026 Apr 16;11(16):24075–85. doi: 10.1021/acsomega.5c12436 (PMC13129877; doi:10.1021/acsomega.5c12436)
Supplement: Supplementary file 1 [file ao5c12436_si_001.docx]

**Comprehensive Reinvestigation of Carbodiimide Guanylation: HCl-initiated Access to Tri- and Tetrasubstituted Guanidines**

Lukáš Vlk,^a^ Karel Pauk,^b^ Maksim A. Samsonov,^a^ Zdeňka Růžičková, ^a^ Tomáš Chlupatý,^a,^* and Aleš Růžička^a,^*

*^a^University of Pardubice, Studentská 573, CZ-532 10, Pardubice, Czech Republic.*

*^b^Institute of Organic Chemistry and Technology, Faculty of Chemical Technology, University of Pardubice, Studentská 573, CZ-532 10, Pardubice, Czech Republic.*

*Tomáš Chlupatý: tomas.chlupaty@upce.cz; Aleš Růžička: – ales.ruzicka@upce.cz

**TABLE OF CONTENTS**

Synthetic aspects and structural behavior.............................................................................................pages S3-S32

List of guanidines **1**–**17** – Figure S1….............................................................................................................page S3

List of guanidines **18**–**29** and **19**^.^HCl, **21**^.^HCl and **23**^.^HCl – Figure S2…...........................................................page S4

List of ^13^C NMR chemical shifts and its calculated chemical shieldings for **26** – Table S1…............................page S5

Correlation of ^13^C NMR chemical shifts and its calculated chemical shieldings for **26** – Figure S3………………page S6

VT ^1^H NMR spectra of **26** @C_6_D_6_ in the range of 295 - 333 K - Figure S4…………………………………………….……...page S7

VT ^1^H NMR spectra of **26** @THF-d_8_ in the range of 173 - 333 K - Figures S5-S6……….…………………..……...pages S8-S9

VT ^1^H NMR spectra of **26** @Tol-d_8_ in the range of 183 - 373 K - Figures S7…….………..…………..………………...page S10

Guanylations initiated by *o*-anisidinium or **21·**HCl and reaction of *o*-anisidinium and **21** – Scheme S1……page S11

NMR yields comparison of guanylation of CDI^Dipp^ and anilines (**20**-**24**, **26-27**) - Figures S8-S13….….…pages S11-S14

Progress of guanylation to **20**, **21**, **22**, **26** using 10 mol % of HCl. -Table S2……………………………………………..page S14

DFT calculations…………………………………………………………………………………………………………………………..…pages S15-S17

The DFT-estimated Gibbs free energy profile

for **21** (**23** in italics in parentheses) – Figure S14…………..…………….…………………………………..……………………….page S16

The DFT-estimated Gibbs free energy profile of guanylation of amines and

carbodiimides with various substituents - Figure S15……………………………………………………..……………………….page S17

Molecular structures of **1**, **2**, **4**-**8**, **10**, **12**-**15**, **17**-**29**, **19**^.^HCl, **21**^.^HCl and **23**^.^HCl - Figures S16-S43…....pages S18-S32

General methods.................................................................................................................................pages S33-S34

General methods for guanylation reactions (**1**–**29**) – **Method A**, **B** and **C**...........................................pages S34-S35

Preparation of guanidines **1**–**29**……………………………………………………….................................................pages S35-S62

Crystallography……………………………………………………………………………………….........................................pages S62-S91

Diffraction data for **1**-**2**, **4**-**8**, **10**, **12**-**15**, **17**–**29**, **19^.^**HCl, **21**^.^HCl and **23**^.^HCl – Tables S3-S30…….…….....pages S64-S91

NMR spectra of **20**-**27** and **29** - Figures S43-S71…………………………………………………………………………..pages S92-S120^[[1]](#footnote-1)^

SYNTHETIC ASPECTS AND STRUCTURAL BEHAVIOR


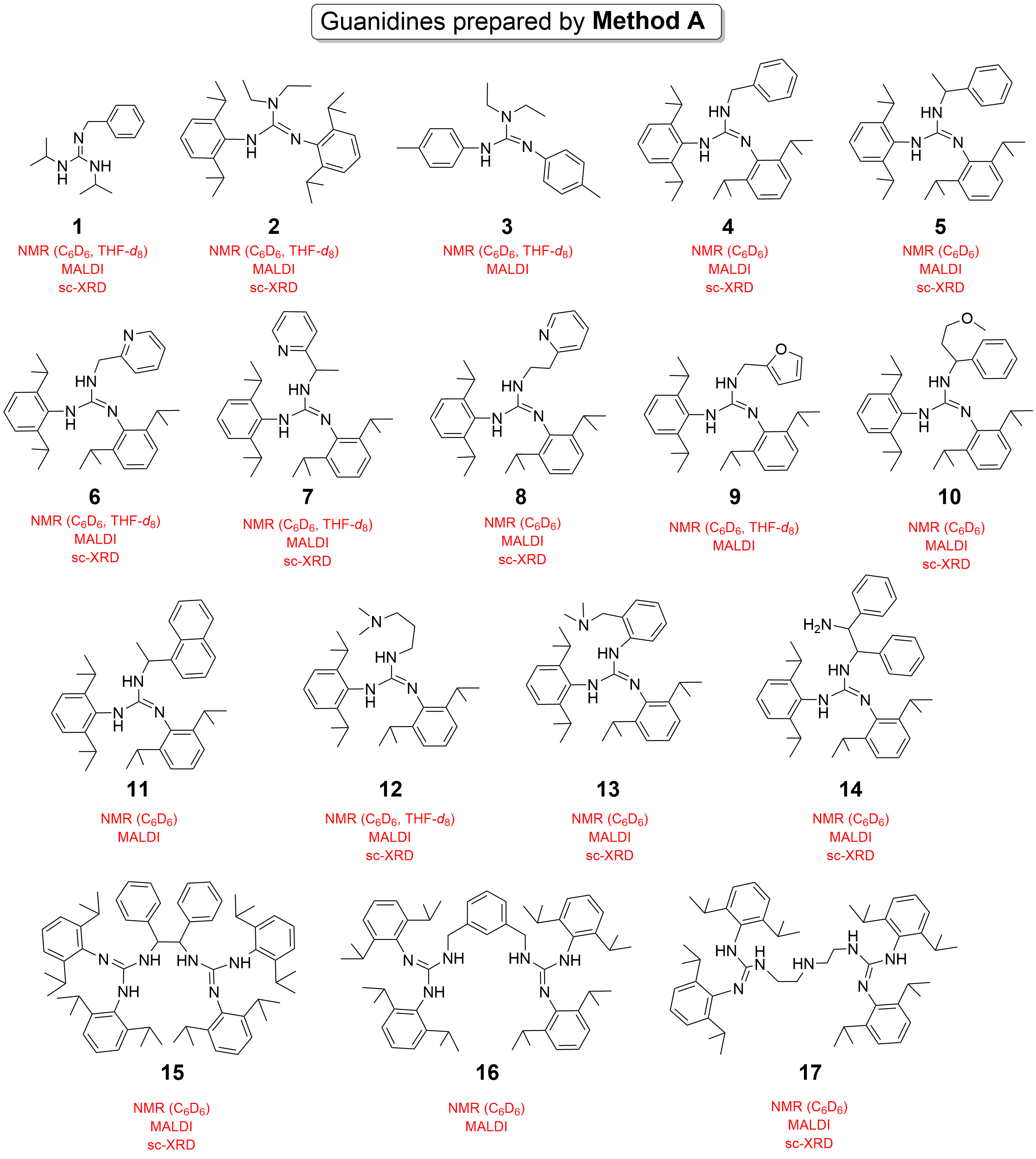


**Figure S1**. Structural formulae of guanidines **1**–**17** prepared by **Method A** (methods used for structure characterization are given in red).


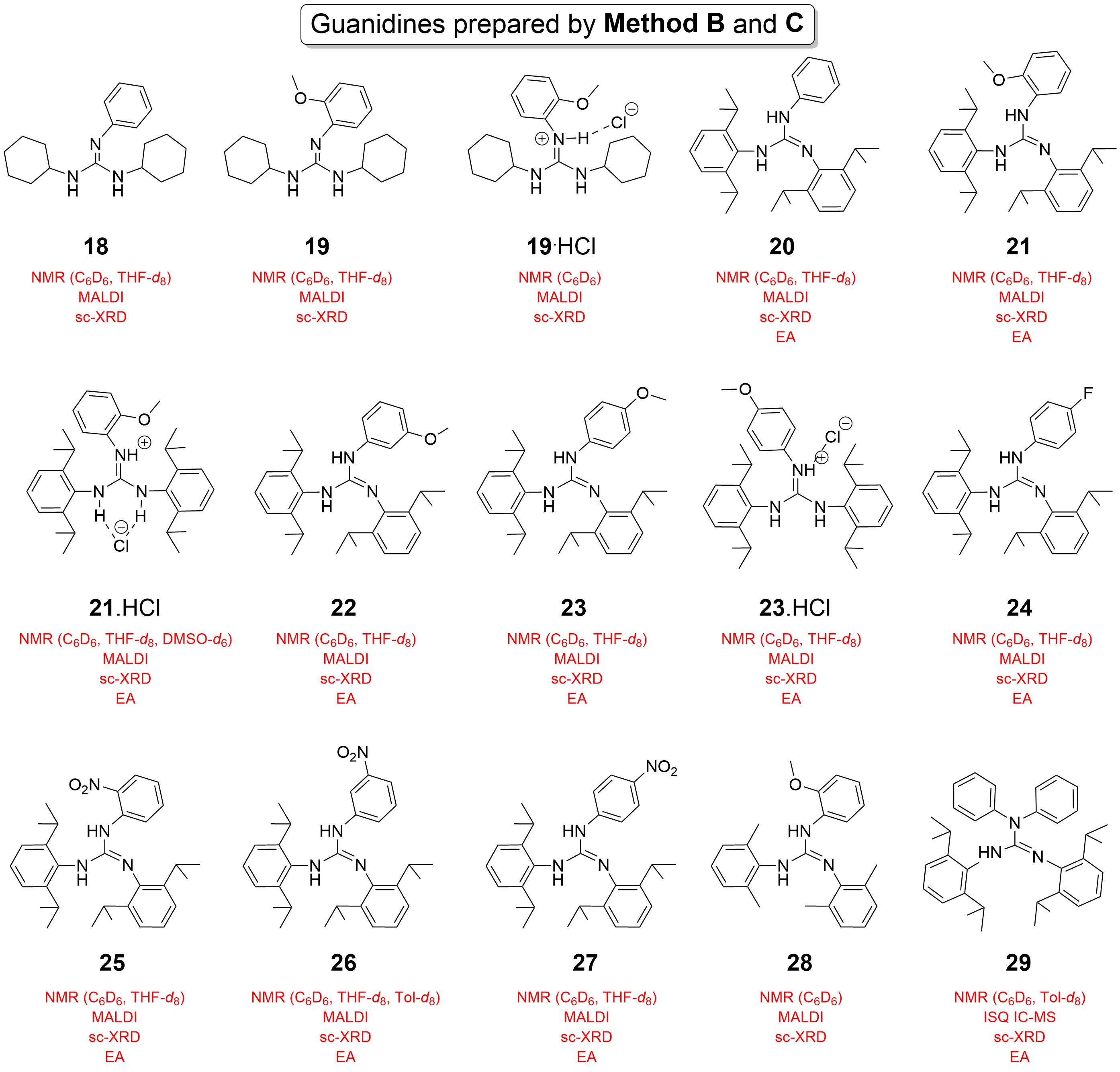


**Figure S2**. Structural formulae of guanidines **18**–**29** and guanidiniums **19**^.^HCl, **21**^.^HCl and **23**^.^HCl prepared by **Methods B** and/or **C** (methods used for structure characterization are given in red).

**Table S1.** List of measured ^13^C NMR chemical shifts in THF-d_8_ solution and calculated chemical shieldings for particular carbon atoms in major **26A**+**26A´** and minor **26B** forms.

|  | **major tautomer**  15.4% of **26A** + 84.6% of **26A´**^a^ | | **minor tautomer**  **26B** | |
| --- | --- | --- | --- | --- |
| **carbon**  **atom** | **measured**  **δ(^13^C) [ppm]** | **calculated**  **shielding [ppm]** | **measured**  **δ(^13^C) [ppm]** | **calculated**  **shielding [ppm]** |
| Ar_q_^MNO^-NO_2_ | 149.7 | 27.19 | 154.2 | 28.33 |
| Ar_q_^Gua^ | 144.4 | 34.79 | 150.7 | 29.93 |
| Ar_q_^MNO^-NH | 143.3 | 34.64 | 150.4 | 22.74 |
| Ar_q_^Dipp^ | 149.2 | 25.07 | 149.7 | 25.28 |
| Ar_q_^Dipp^ | 144.4 | 31.97 | 148.0 | 26.32 |
| Ar_q_^Dipp^ | 141.1 | 34.75 | 134.9 | 42.33 |
| Ar_q_^Dipp^ | 133.0 | 45.81 | 133.5 | 43.45 |
| ArH^MNO^ | 129.8 | 48.67 | 130.4 | 47.61 |
| ArH^MNO^ | 125.7 | 53.75 | 130.9 | 49.30 |
| ArH^Dipp^ | 129.9 | 47.15 | 129.8 | 49.09 |
| ArH^Dipp^ | 125.0 | 53.98 | 128.1 | 49.56 |
| ArH^Dipp^ | 123.7 | 54.96 | 124.7 | 54.47 |
| ArH^Dipp^ | 123.6 | 55.09 | 123.7 | 54.92 |
| ArH^MNO^ | 116.7 | 61.95 | 117.9 | 57.59 |
| ArH^MNO^ | 114.7 | 66.06 | 115.5 | 63.47 |
| CH^Dipp^ | 29.5 | 148.74 | 29.7 | 148.41 |
| CH^Dipp^ | 29.3 | 148.74 | 29.4 | 148.69 |
| CH_3_^Dipp^ | 25.2 | 157.97 | 25.4 | 156.48 |
| CH_3_^Dipp^ | 24.4 | 158.30 | 25.1 | 157.45 |
| CH_3_^Dipp^ | 24.4 | 159.92 | 24.2 | 159.84 |
| CH_3_^Dipp^ | 22.9 | 161.21 | 23.3 | 160.62 |

^a^ based on Boltzmann probability distribution


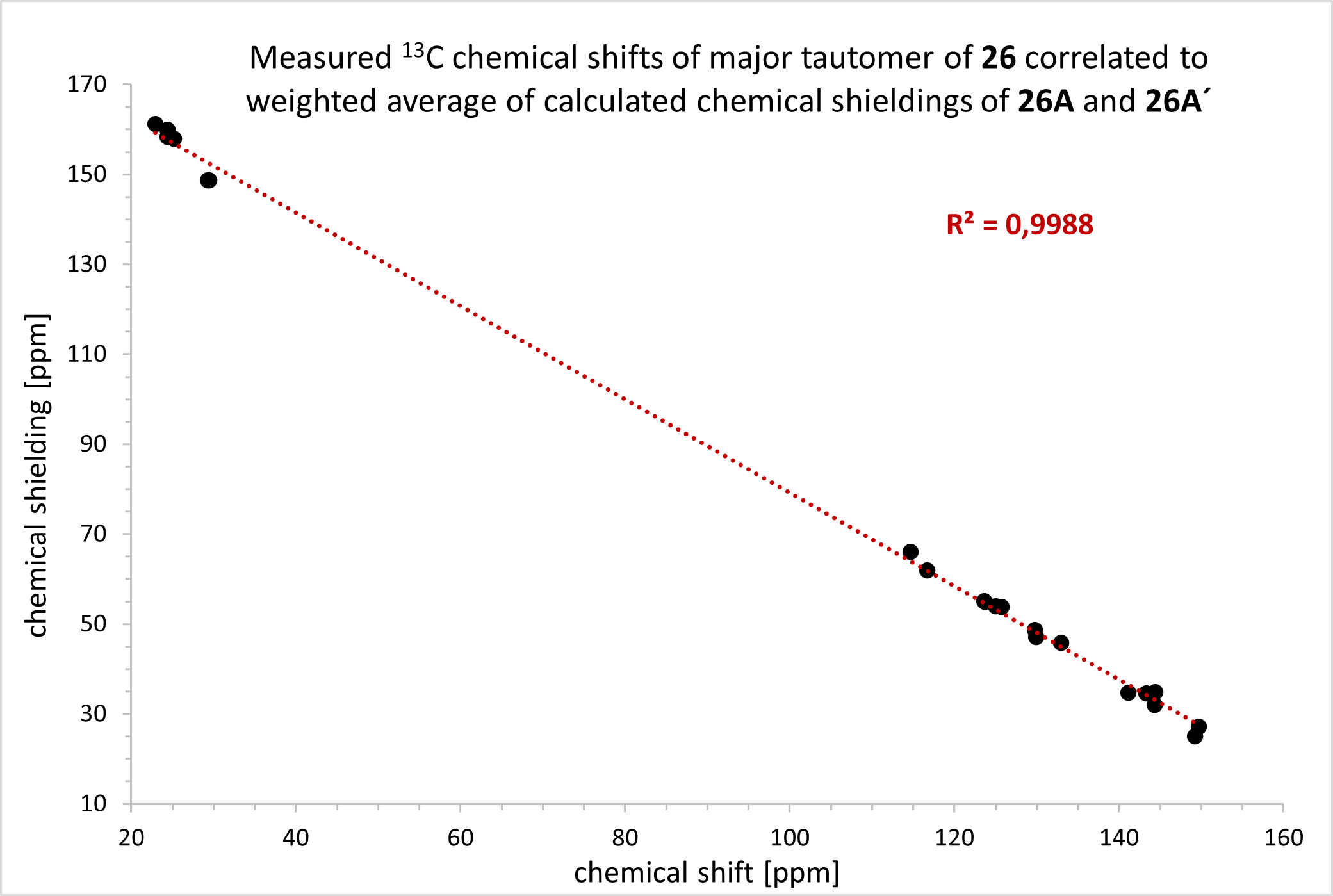


**R^2^ = 0.9988**


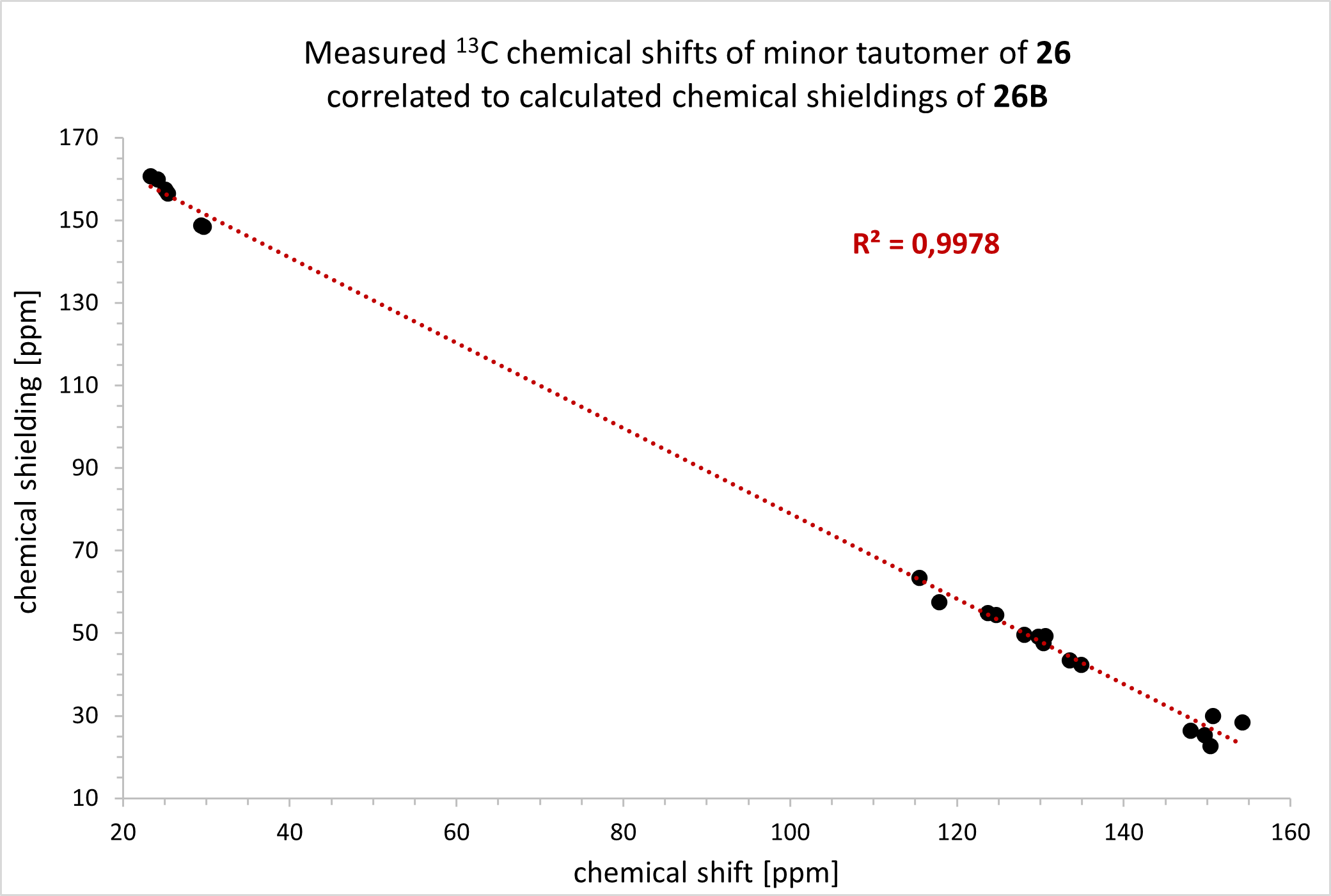


**R^2^ = 0.9978**

**Figure S3**. Correlation (including coefficient of determination R^2^) of measured ^13^C NMR chemical shifts and its calculated chemical shieldings for major **26A** and **26A´** and minor **26B** tautomers of **26**.


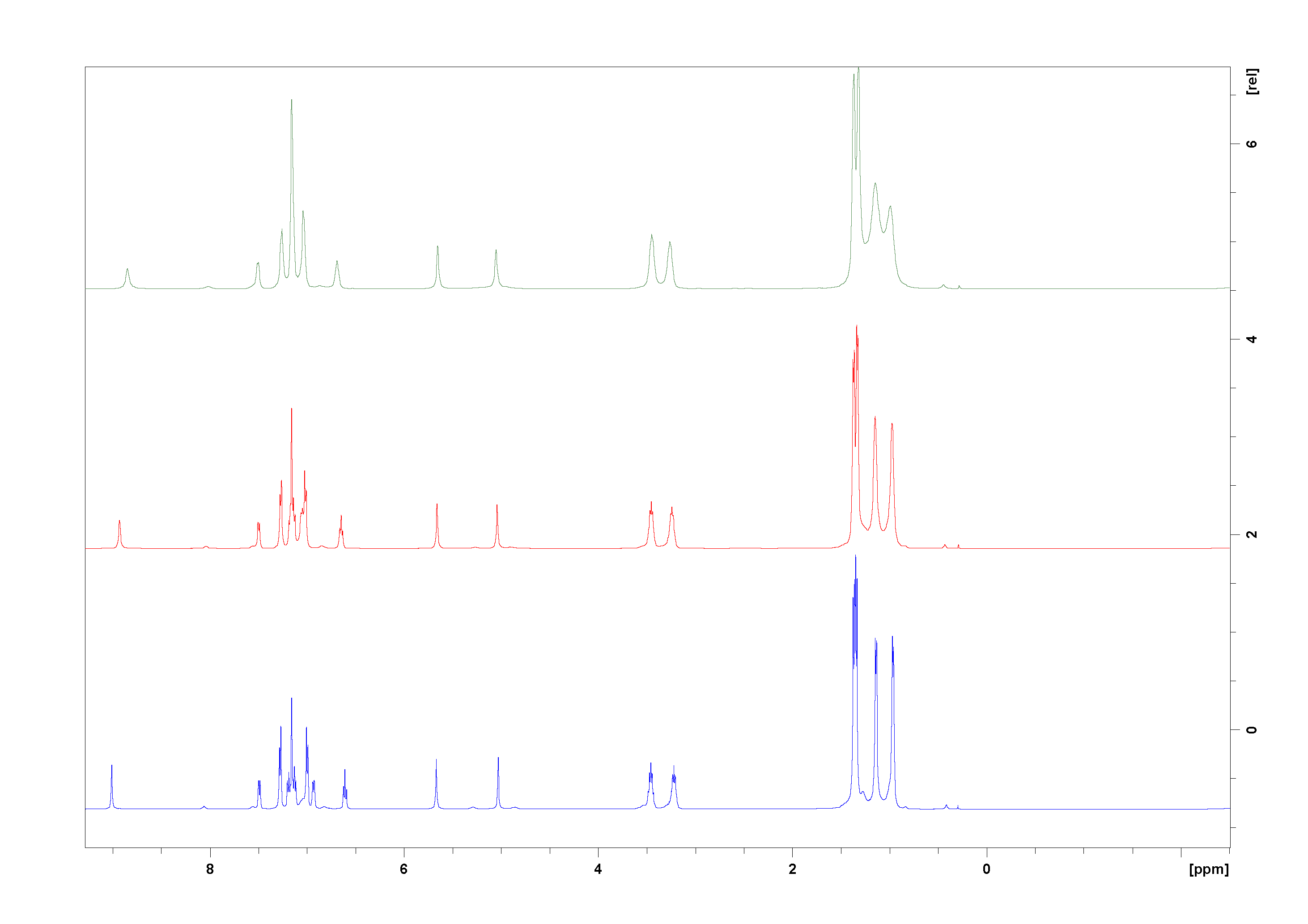


**●**

**●**

**●**

**●**

**●**

**●**

**●**

**●**

**●**

**●**

**●**

**●**

**●**

**●**

**●**

**●**

**●**

**●**

**●**

**●**

**●**

molar ratio(%) *ca* 91:9

molar ratio(%) *ca* 91:9

molar ratio(%) *ca* 91:9

**295 K**

**313 K**

**333 K**

**Figure S4**. VT ^1^H NMR spectra of **26** @C_6_D_6_, 295 - 333 K. Signals of minor tautomer are marked with black dots.


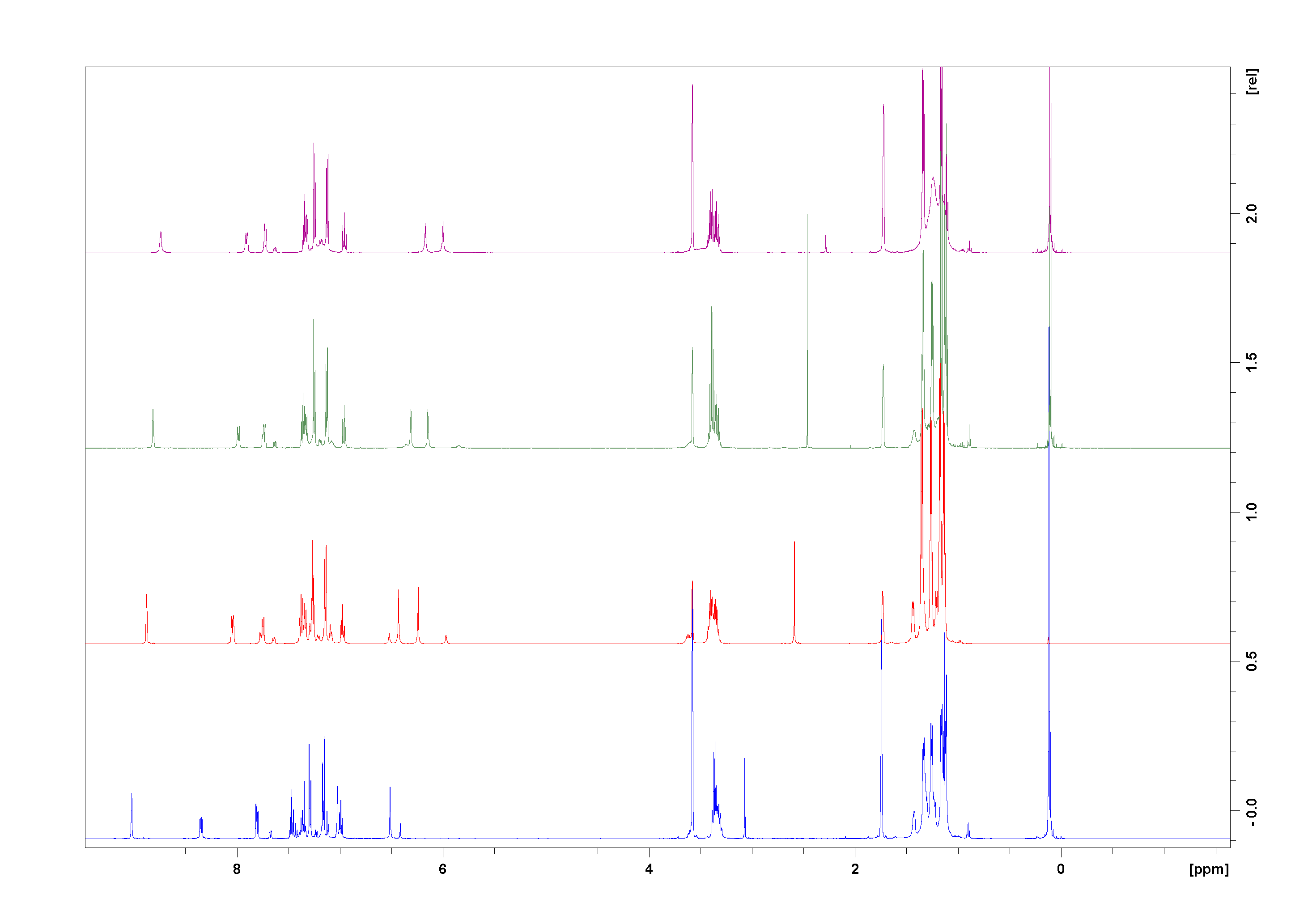


**●**

**●**

**●**

**●**

**●**

**●**

**●**

**●**

**●**

**●**

**●**

**●**

**●**

**●**

**●**

**●**

**●**

**●**

**●**

**●**

**●**

**●**

**●**

**●**

**●**

**●**

**●**

molar ratio(%) *ca* 80:20

molar ratio(%) *ca* 81:19

molar ratio(%) *ca* 82:18

**●**

**173 K**

**280 K**

**295 K**

**333 K**

molar ratio(%) *ca* 82:18

**Figure S5**. VT ^1^H NMR spectra of **26** @THF-d_8_, 173 - 333 K. Signals of minor tautomer are marked with black dots.


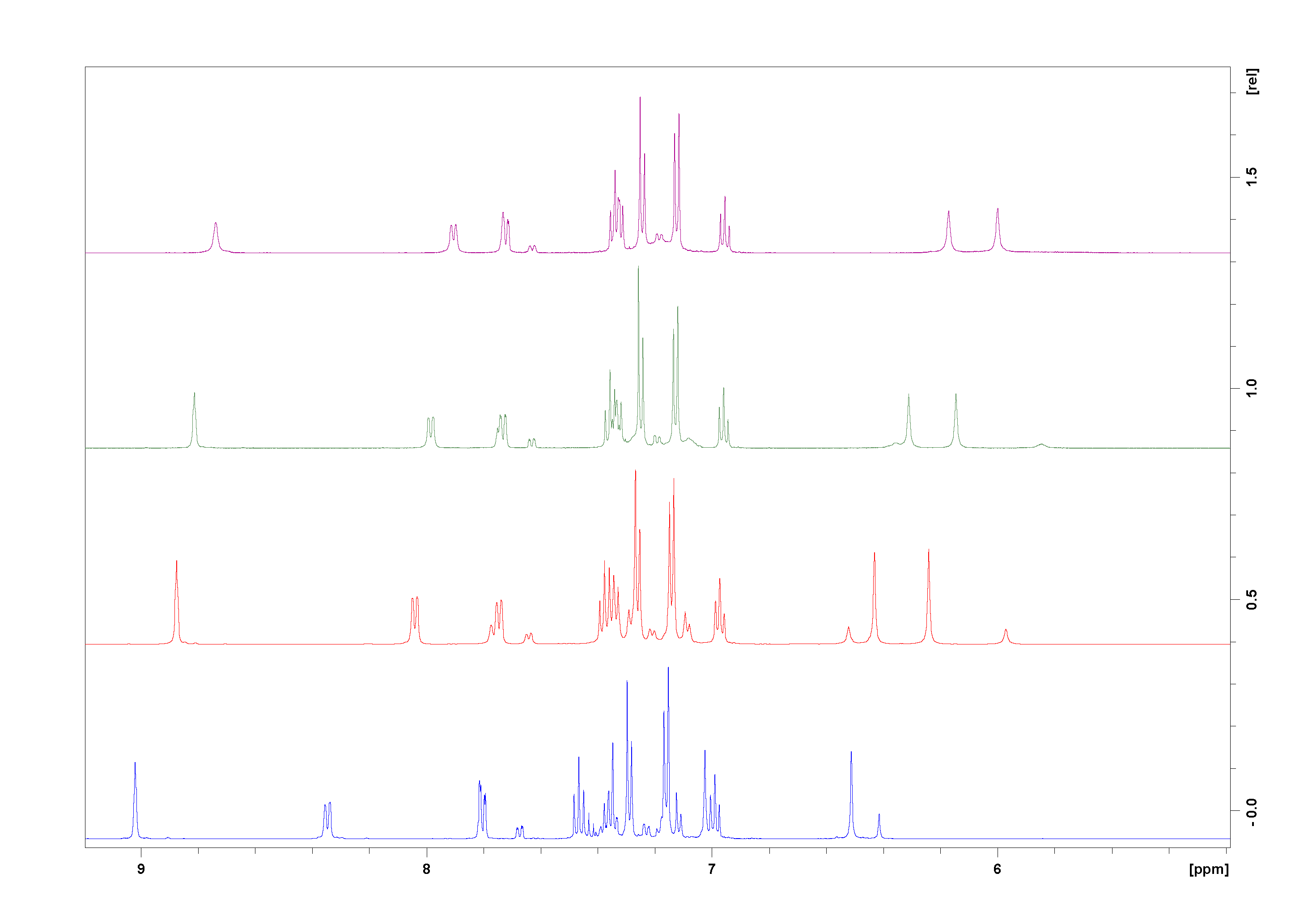


**●**

**●**

**●**

**●**

**●**

**●**

**●**

**●**

**●**

**●**

**●**

**●**

**●**

**●**

**●**

**●**

**●**

**●**

**●**

molar ratio(%) *ca* 81:19

molar ratio(%) *ca* 82:18

molar ratio(%) *ca* 82:18

molar ratio(%) *ca* 80:20

**173 K**

**280 K**

**295 K**

**333 K**

**Figure S6**. Detail of VT ^1^H NMR spectra of **26** @THF-d_8_, 173 - 333 K. Signals of minor tautomer are marked with black dots.


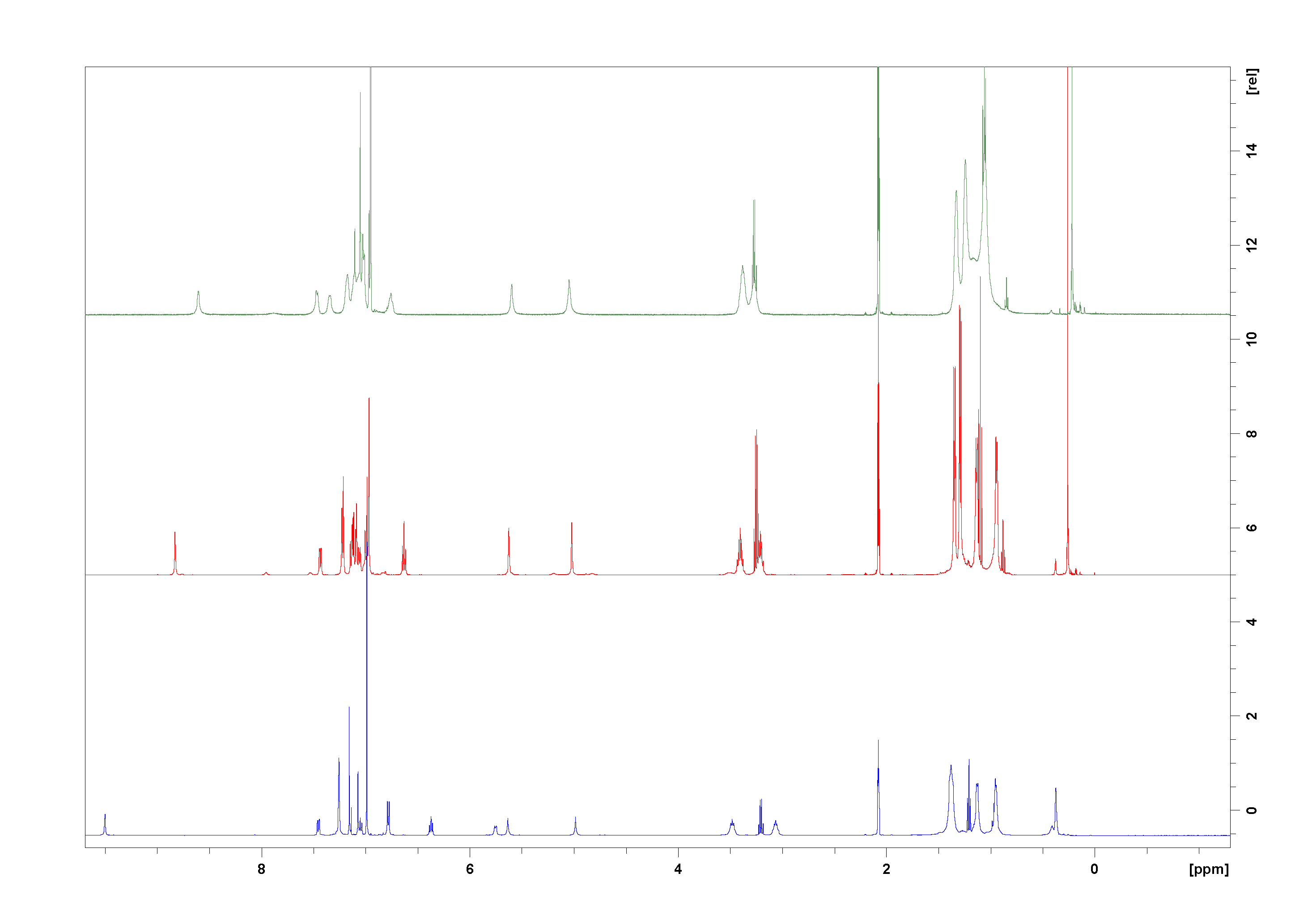


**●**

**●**

**●**

**●**

**●**

**●**

**●**

**●**

**●**

molar ratio(%) *ca* >99:<1

molar ratio(%) *ca* 91:9

molar ratio(%) *ca* 85:15

**183 K**

**295 K**

**373 K**

**Figure S7**. VT ^1^H NMR spectra of **26** @Tol-d_8_, 183 - 373 K. Signals of minor tautomer are marked with black dots.


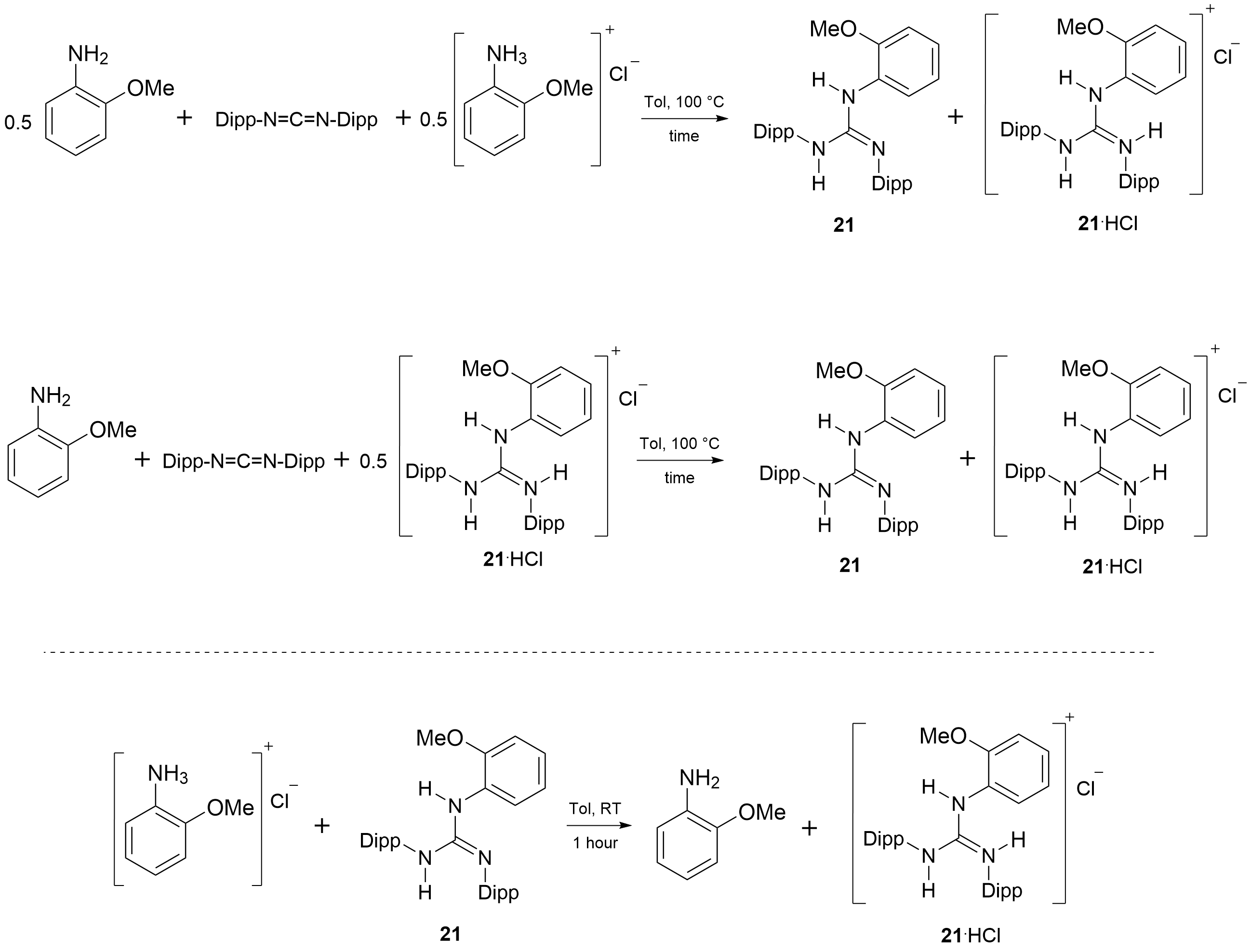


**Scheme S1.** Comparative experiments/guanylations initiated by *o*-anisidinium chloride (top) or **21**·HCl (middle) mimicking 50 mol % of HCl. The reaction of *o*-anisidinium chloride with **21** is presented in a bottom.


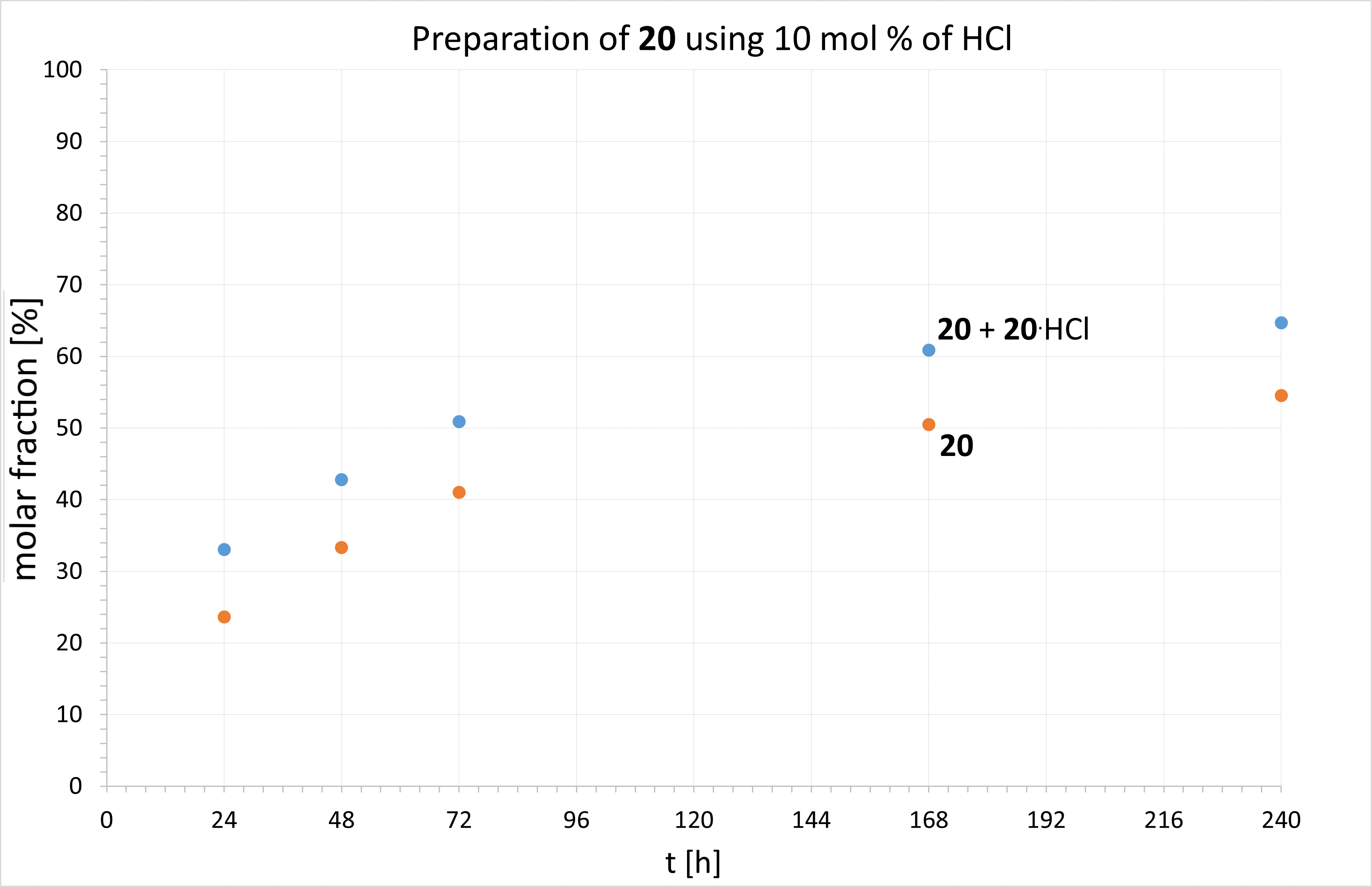

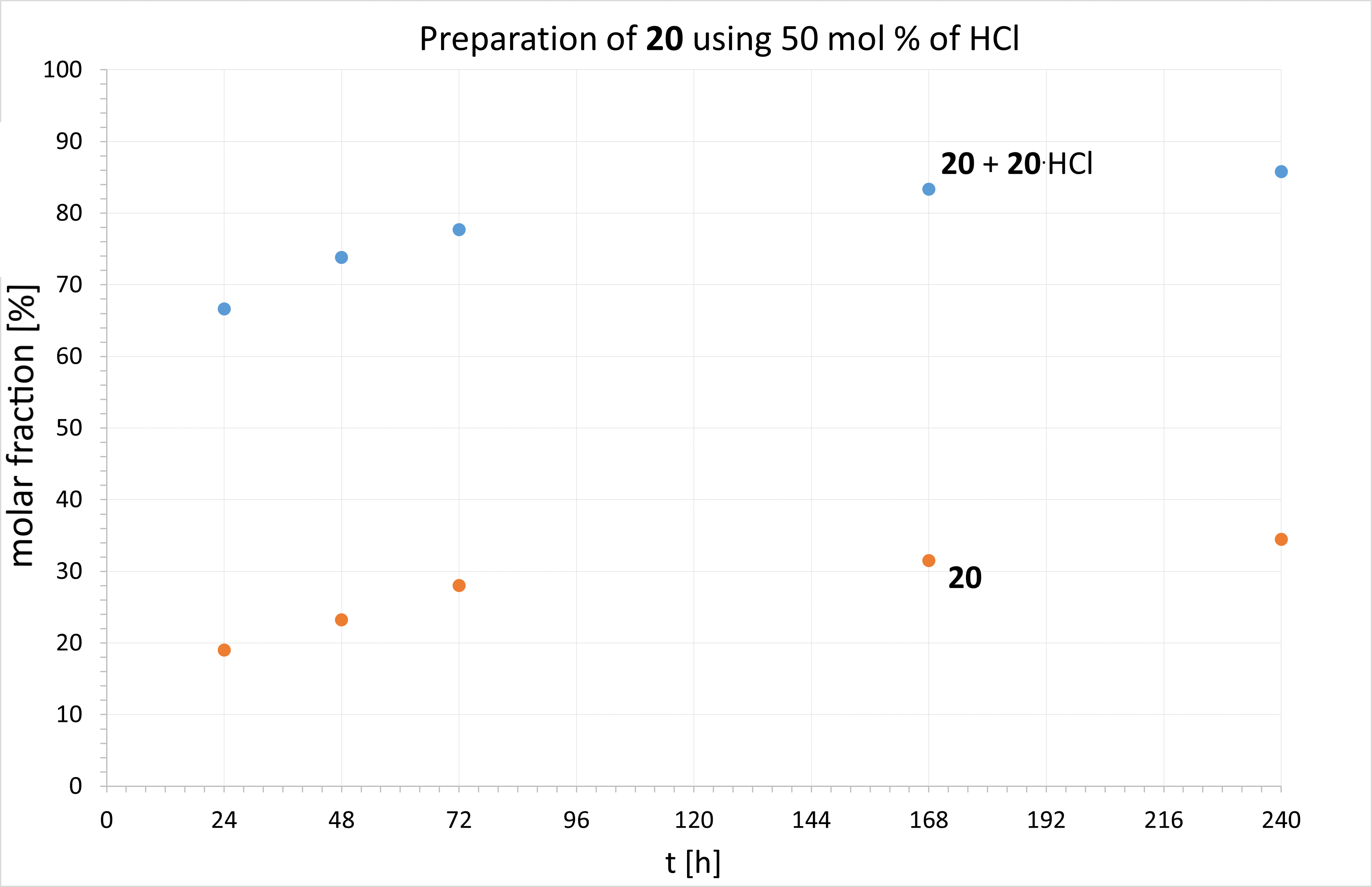


**Figure S8**. NMR yield of guanylation reactions of CDI^Dipp^ by aniline to **20** using 10 (left) and 50 (right) mol % of HCl.


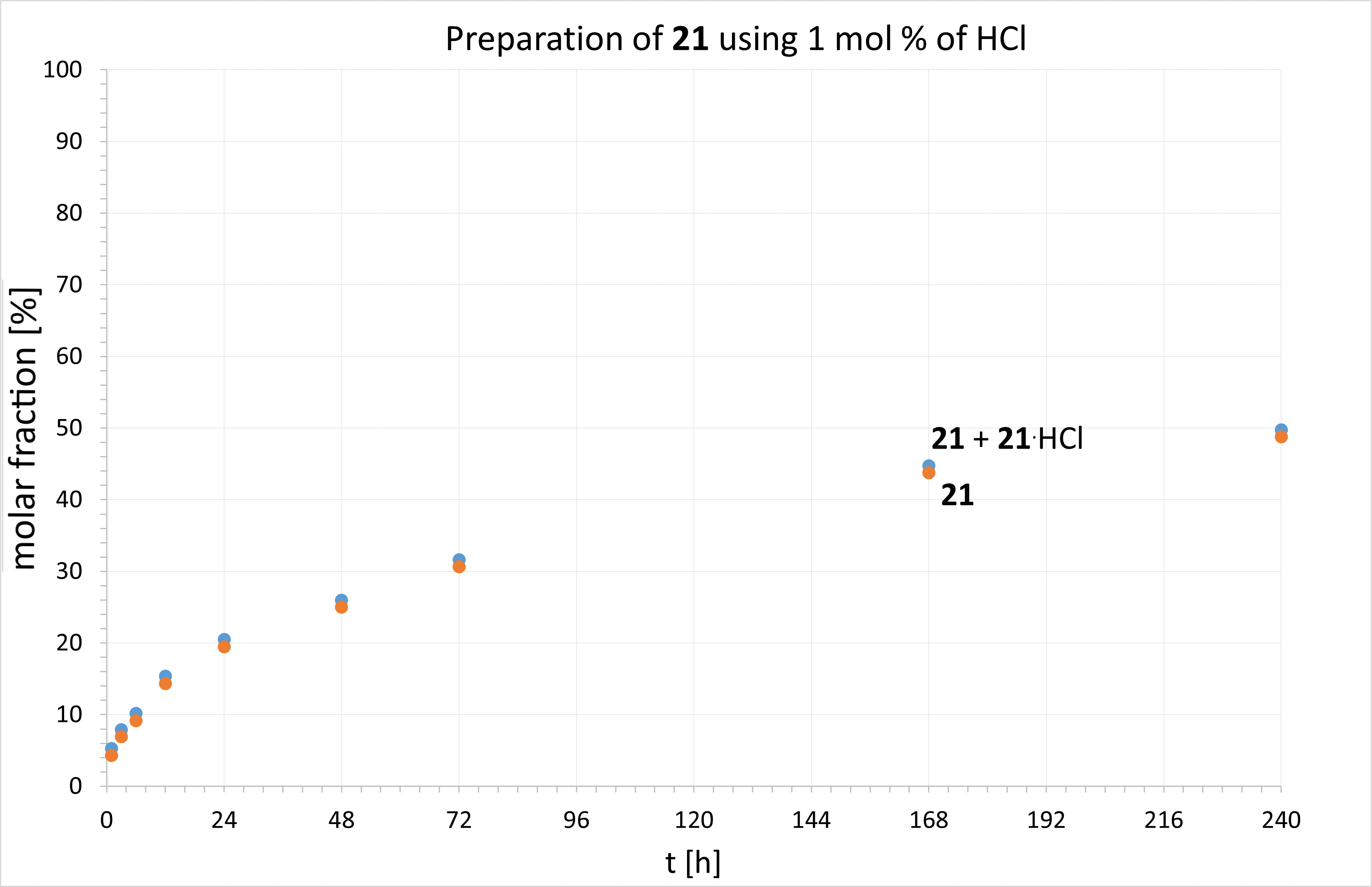

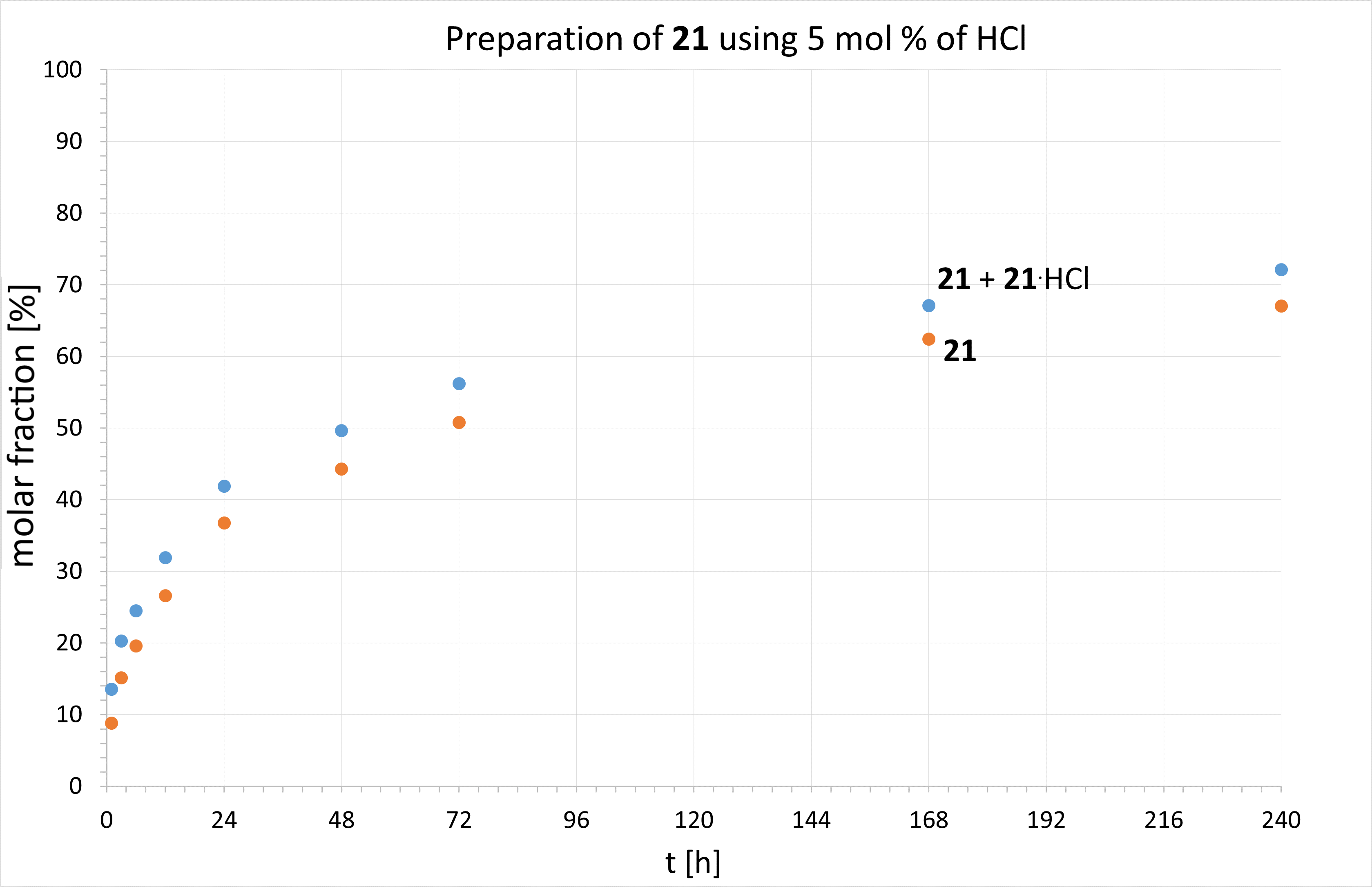

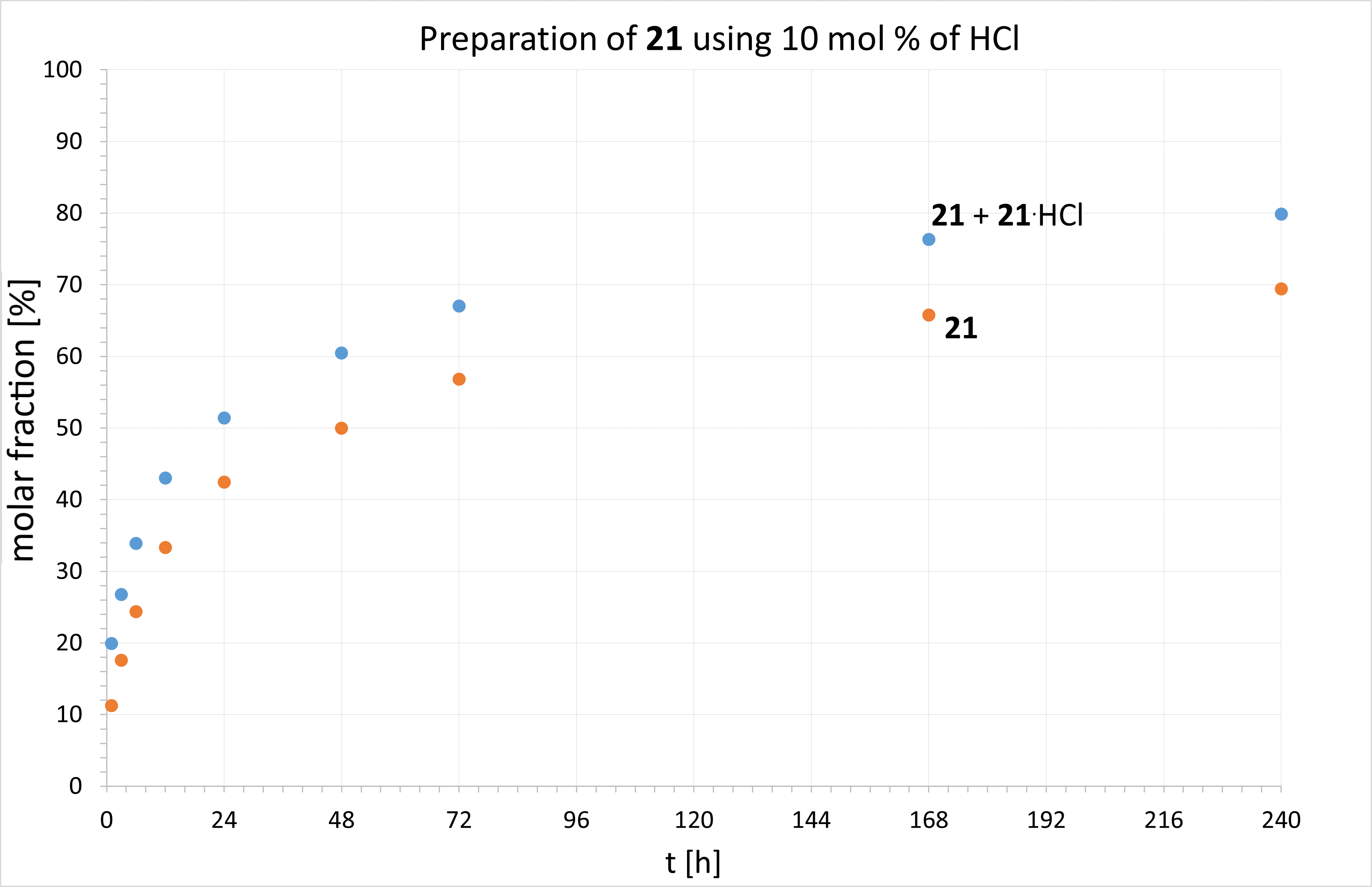

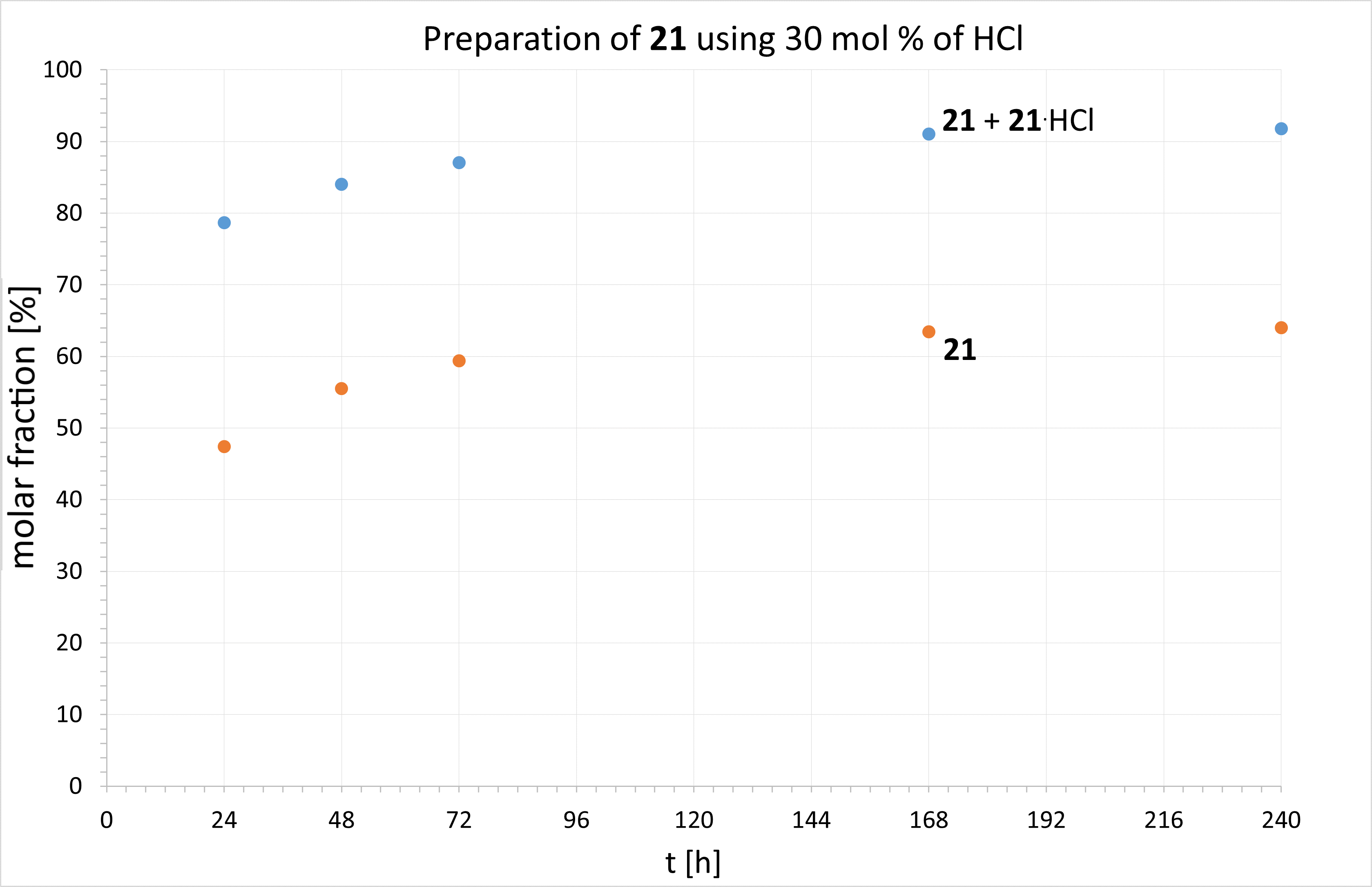

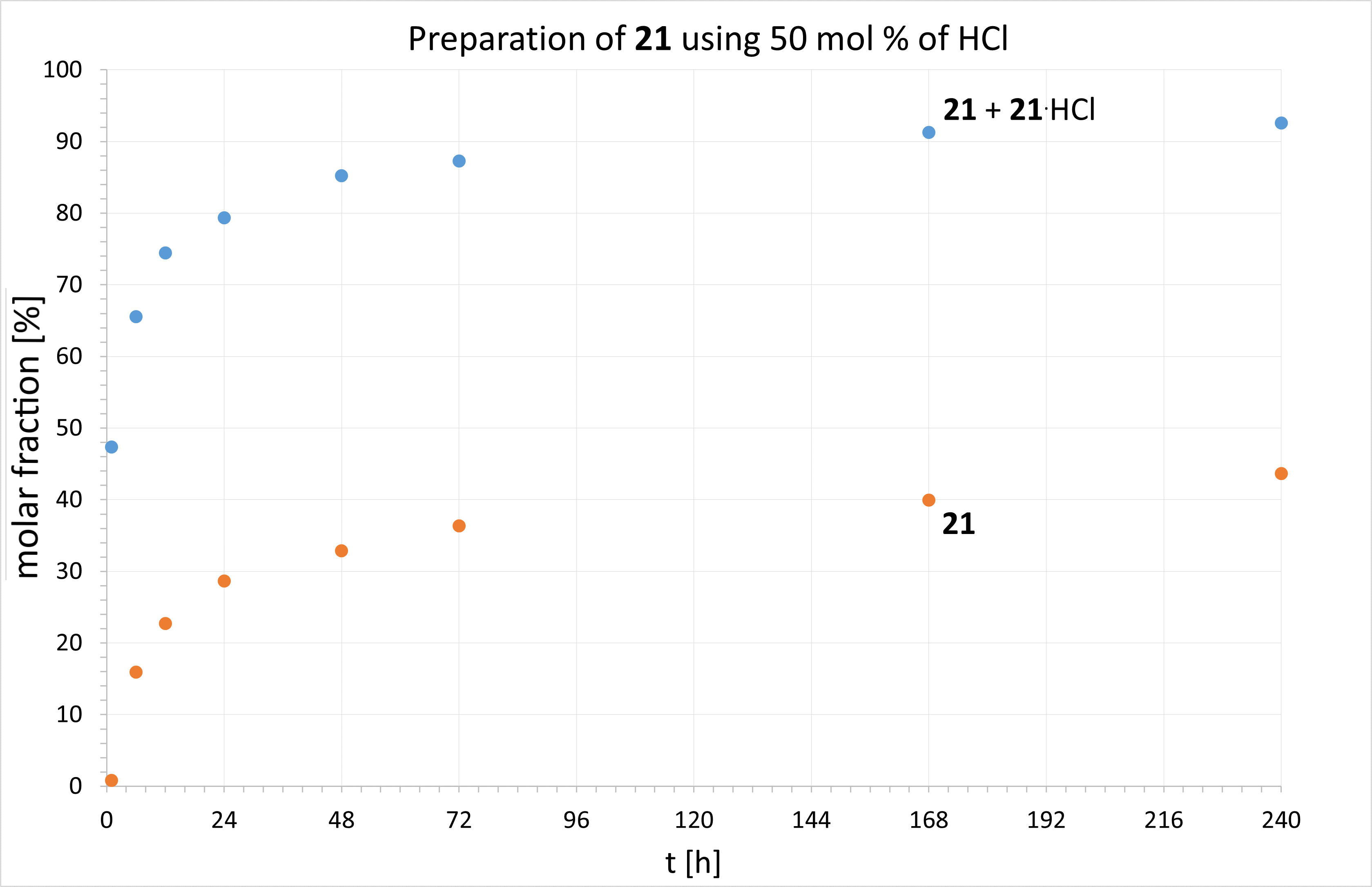

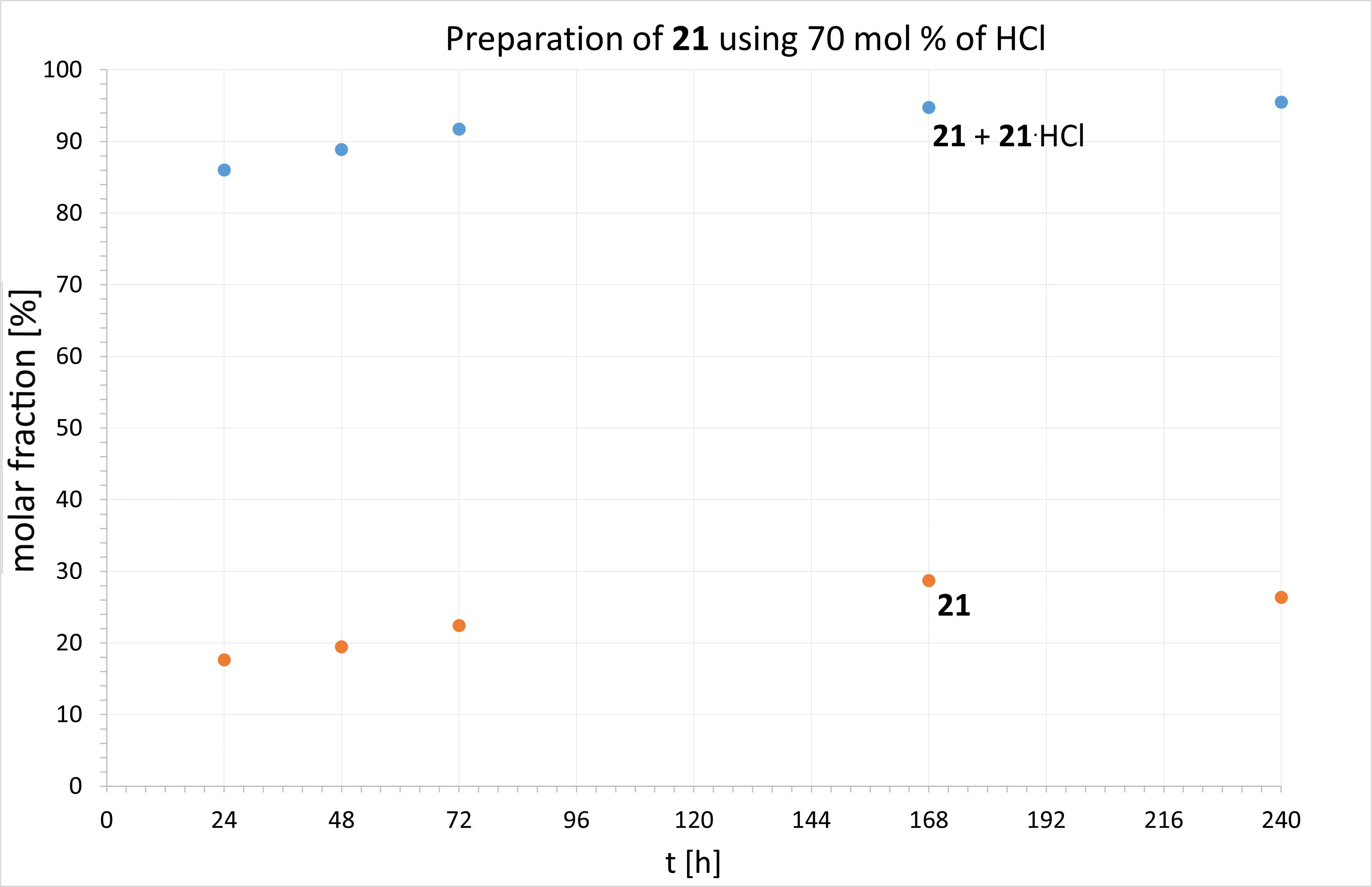


**Figure S9**. NMR yield of guanylation reactions of CDI^Dipp^ by *o*-anisidine to **21** using 1, 5, 10, 30, 50 and 70 mol % of HCl.


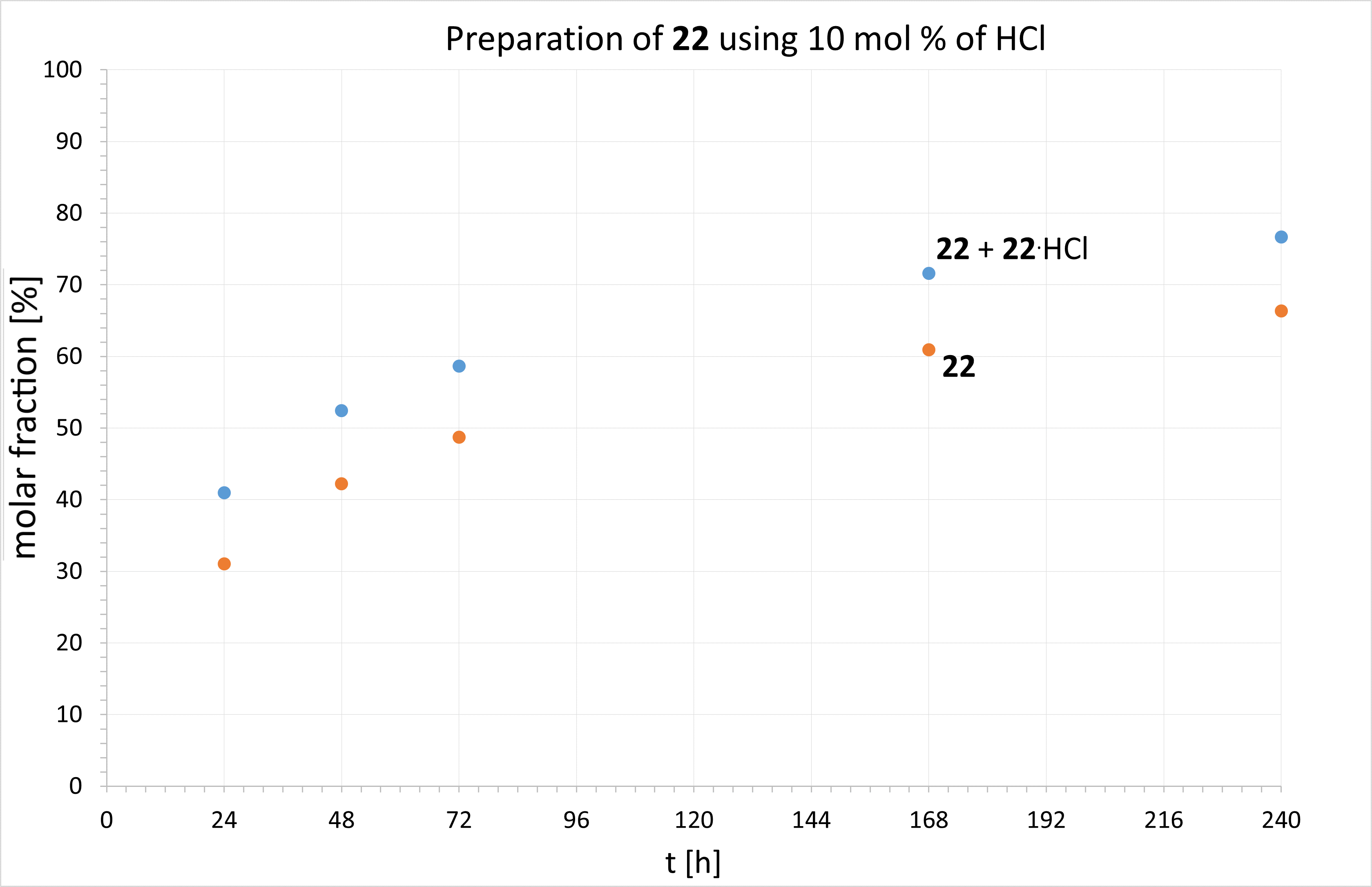

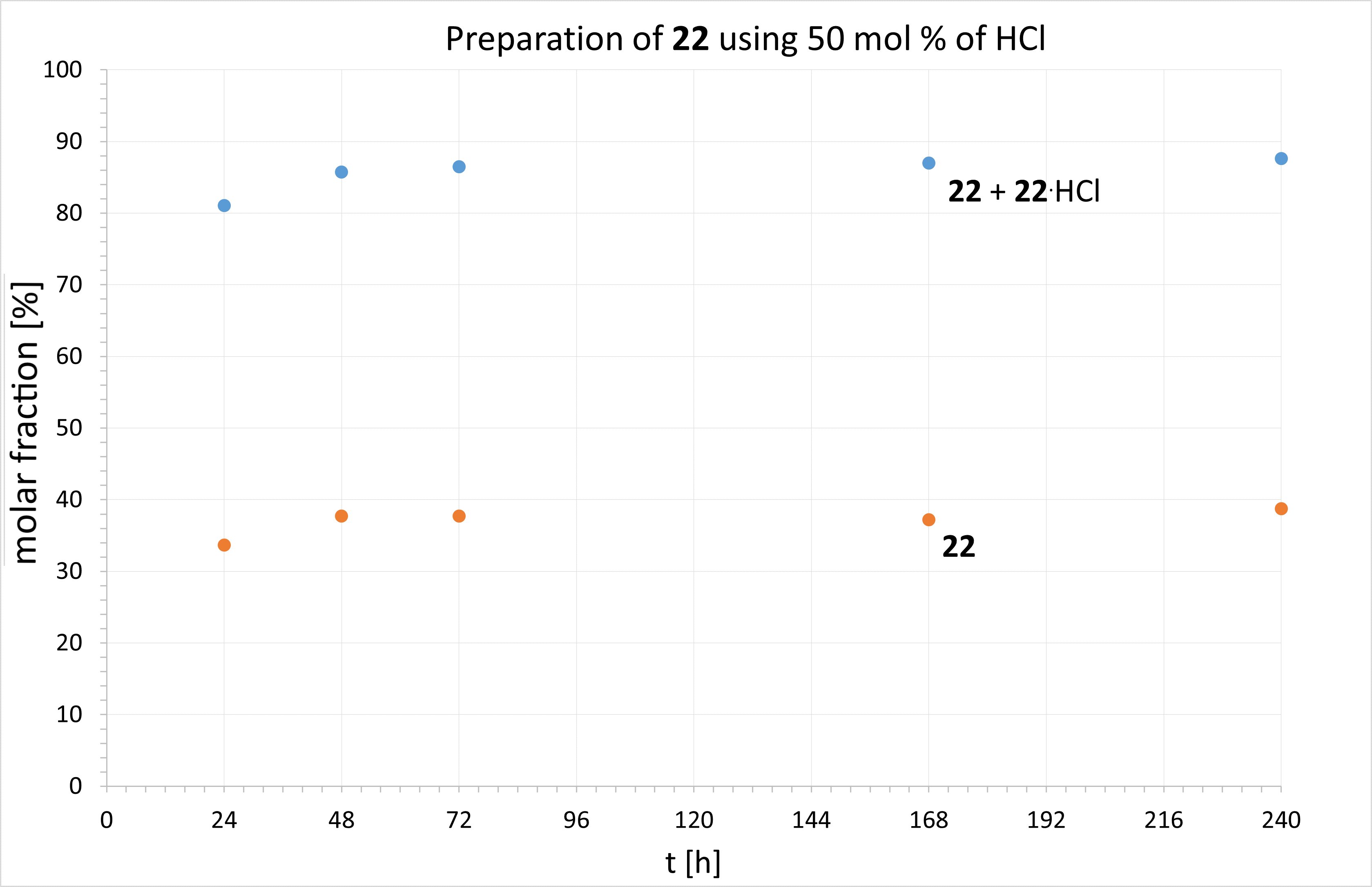


**Figure S10**. NMR yield of guanylation reactions of CDI^Dipp^ by *m*-anisidine to **22** using 10 (left) and 50 (right) mol % of HCl.


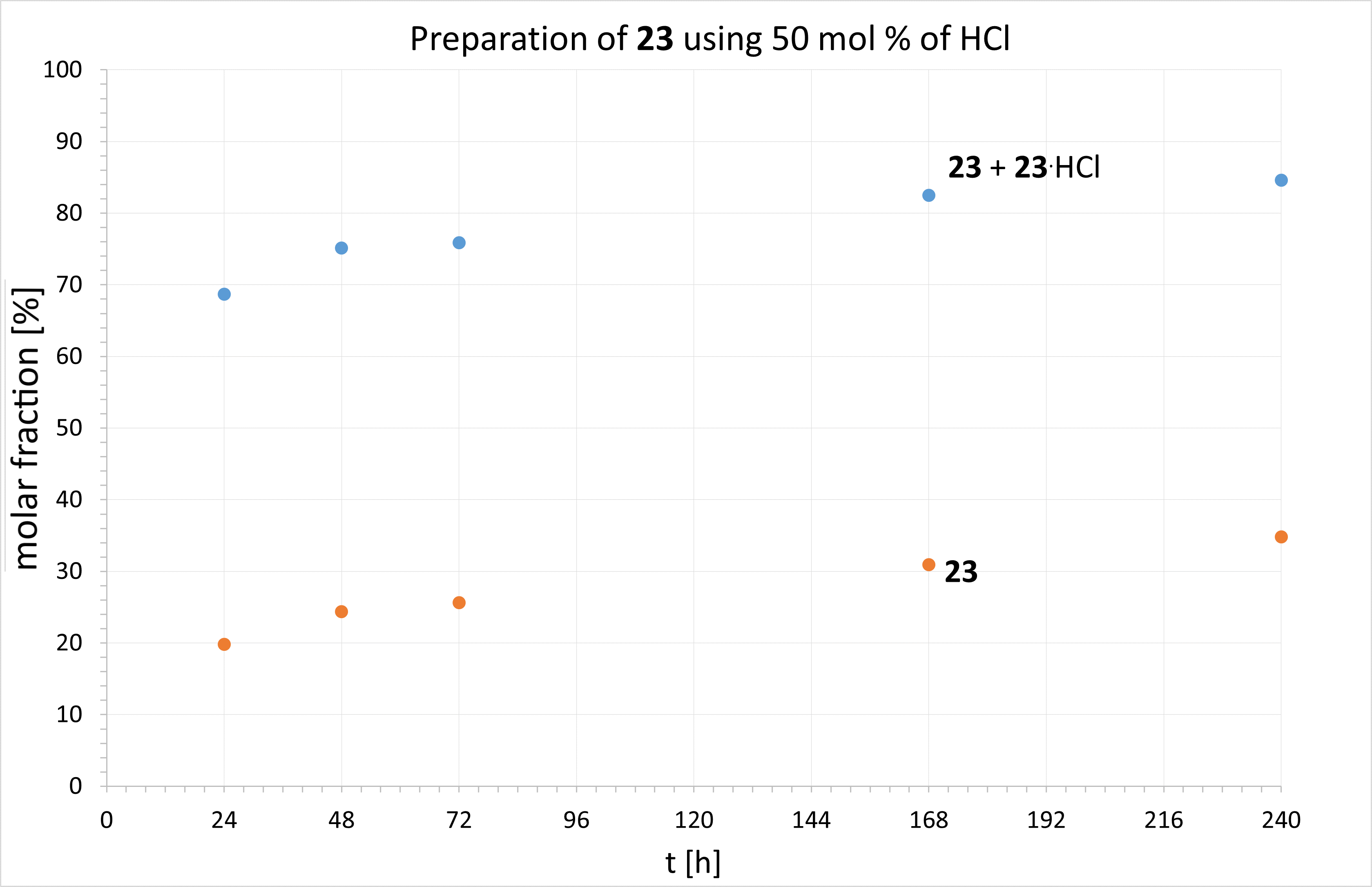

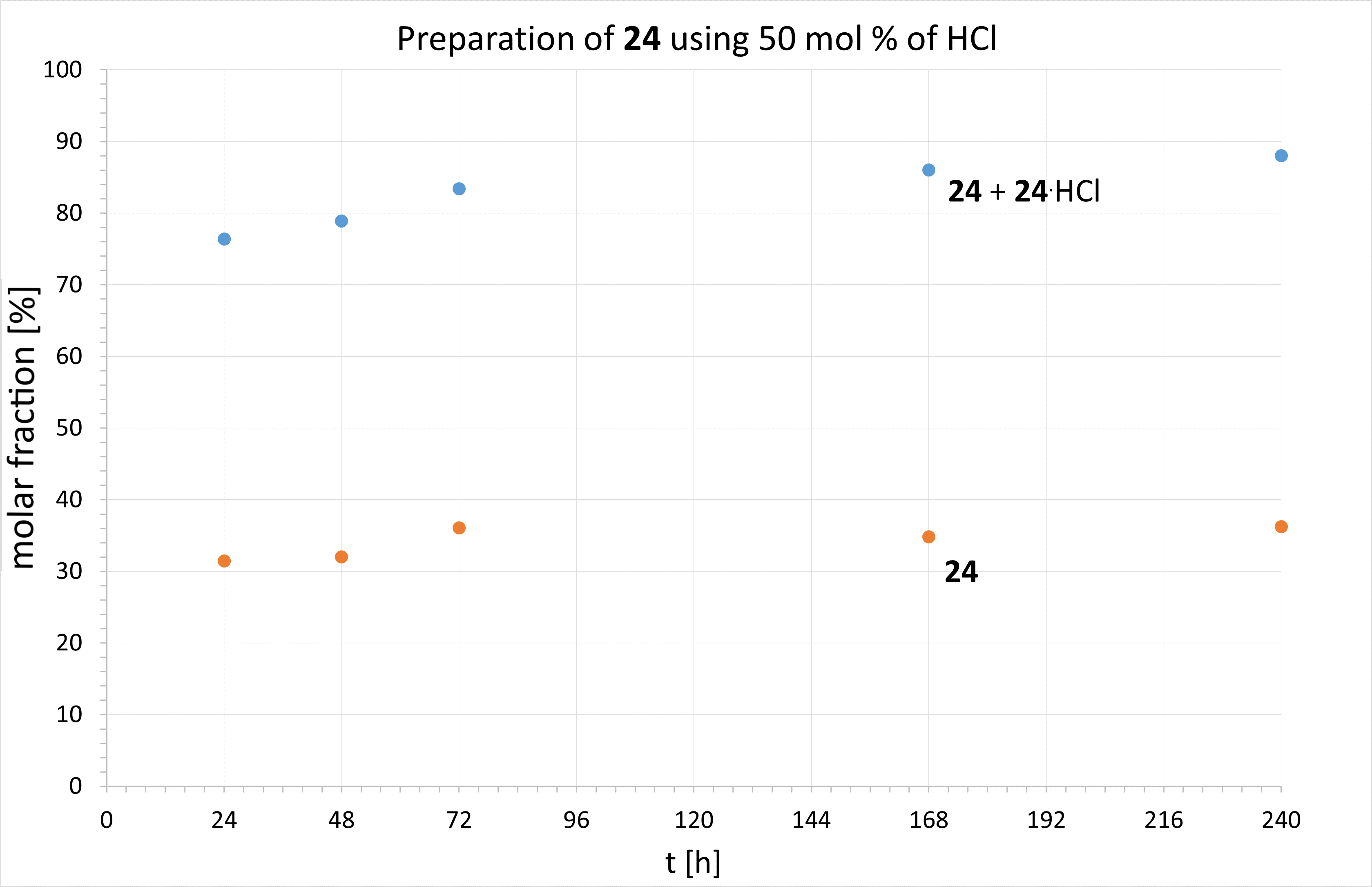


**Figure S11**. NMR yield of guanylation reactions of CDI^Dipp^ by *p*-anisidine to **23** (left) or *p*-fluoroaniline to **24** (right) using 50 mol % of HCl.


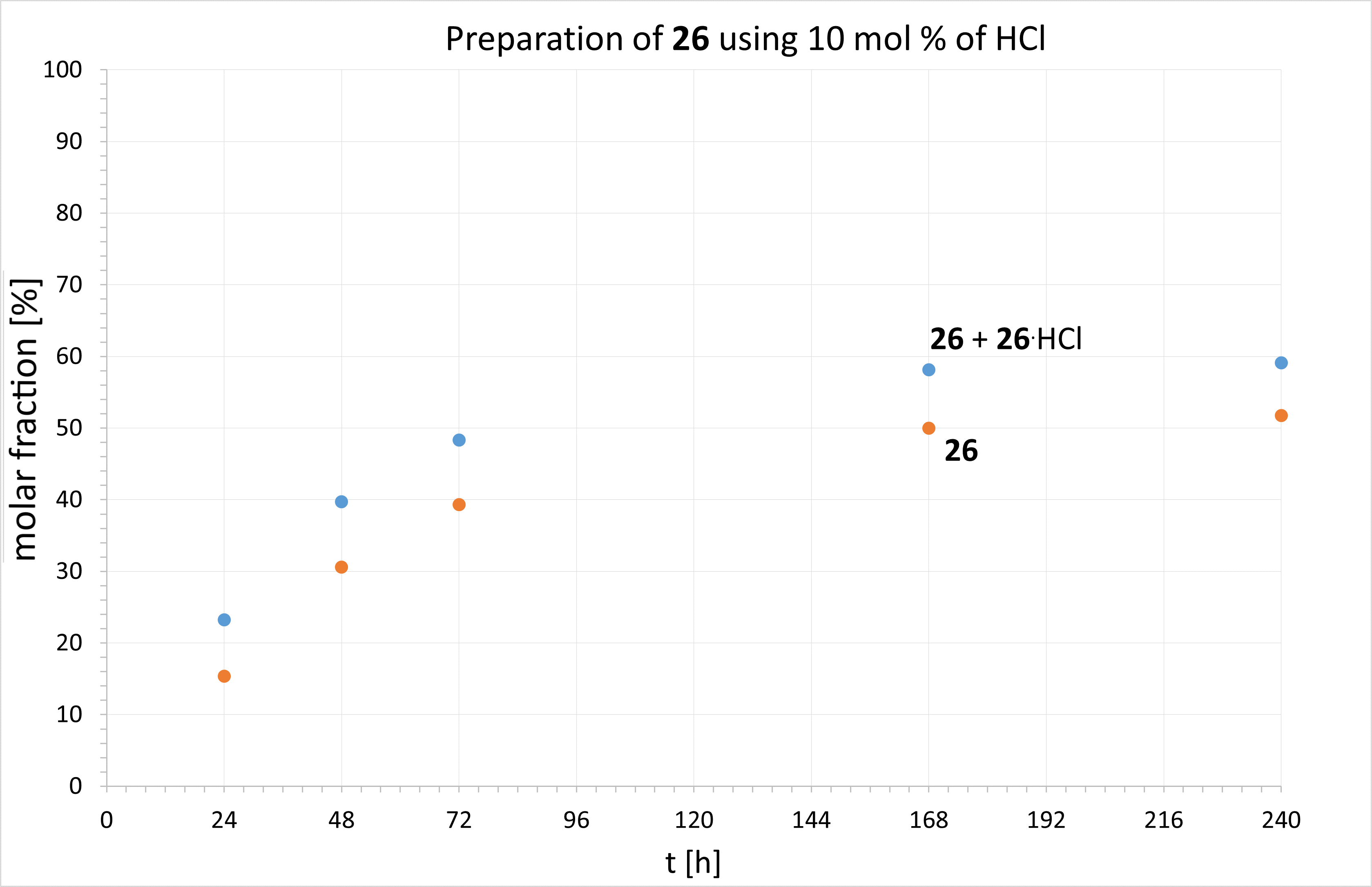

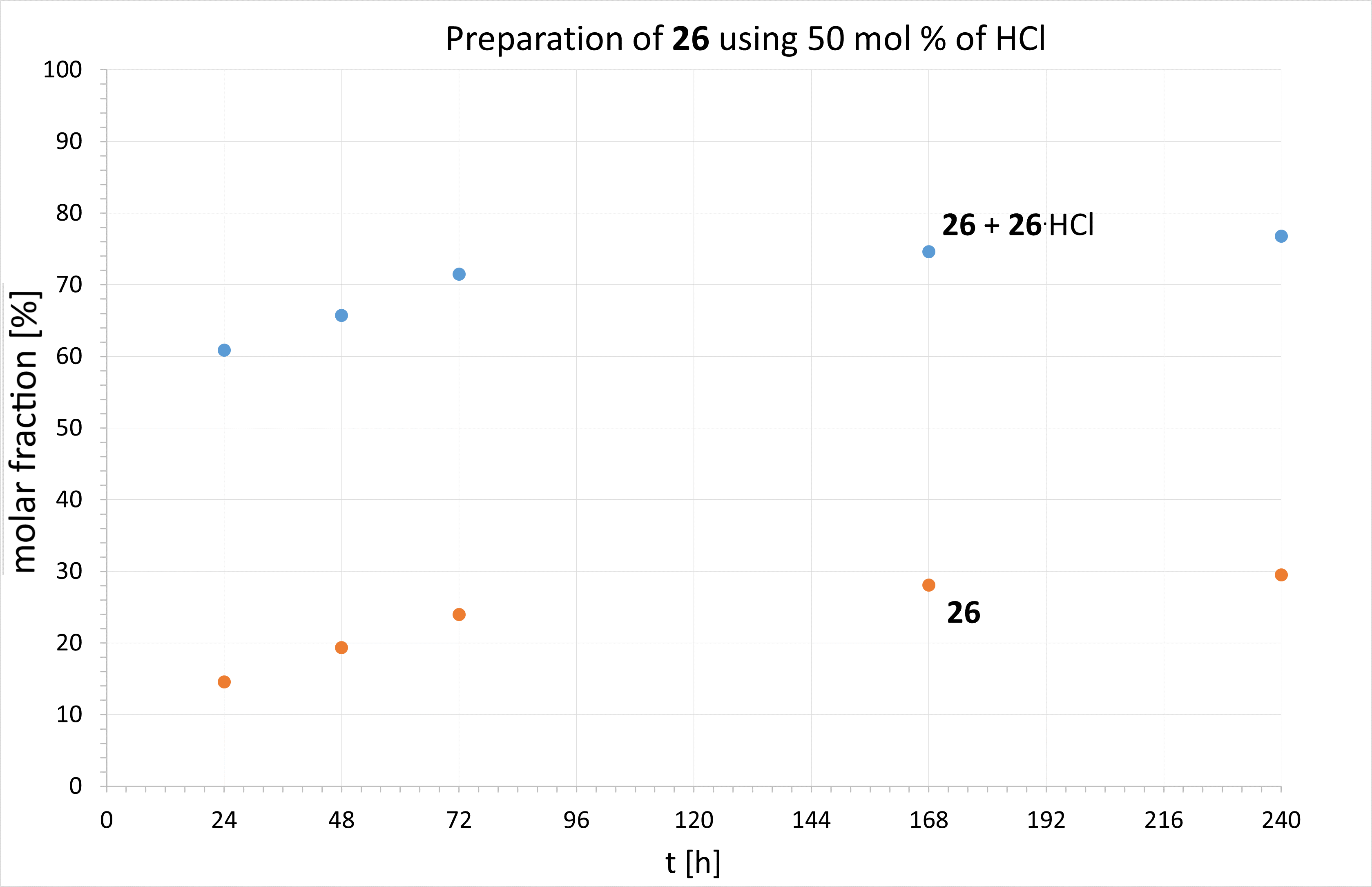


**Figure S12**. NMR yield of guanylation reactions of CDI^Dipp^ by *m*-nitroaniline to **26** using 10 (left) and 50 (right) mol % of HCl.


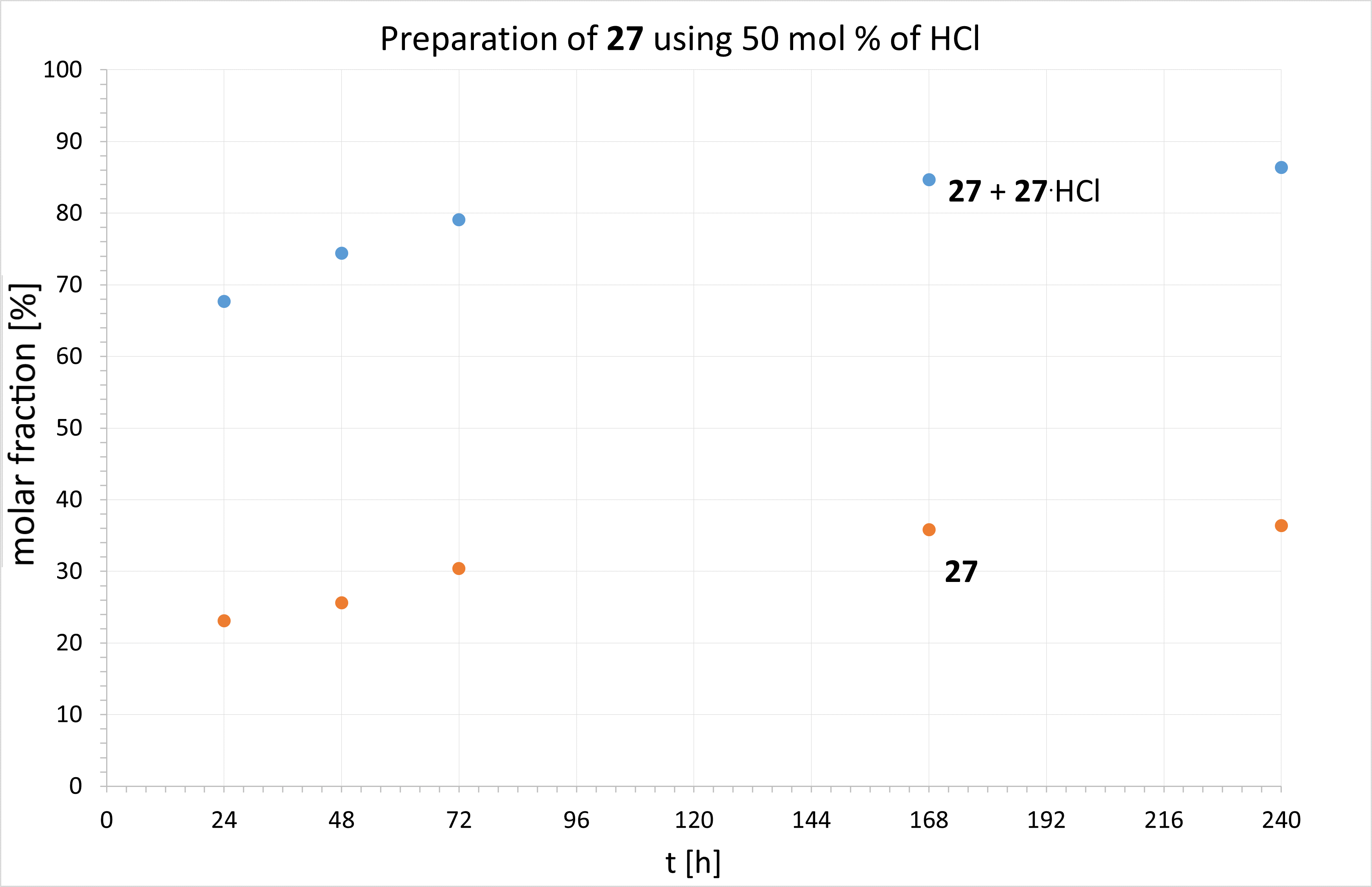


**Figure S13**. NMR yield of guanylation reactions of CDI^Dipp^ by *p*-nitroaniline to **27** using 50 mol % of HCl.

**Table S2.** Progress of guanylation reaction to **20**, **21**, **22**, **26** (CDI^Dipp^ with selected anilines) using 10 mol % of HCl.

|  | **Compounds/conversion [%]*^a^*** | | | |
| --- | --- | --- | --- | --- |
| **t [days]** | **20** | **21** | **22** | **26** |
|  |  |  |  |  |
| 1 | 33 | 51 | 41 | 23 |
|  | (24/9) | (42/9) | (31/10) | (15/8) |
| 2 | 43 | 61 | 52 | 40 |
|  | (33/10) | (50/11) | (42/10) | (31/9) |
| 3 | 51 | 67 | 59 | 48 |
|  | (41/10) | (57/10) | (49/10) | (39/9) |
| 7 | 61 | 76 | 72 | 58 |
|  | (50/11) | (66/10) | (61/11) | (50/10) |
| 10 | 65 | 80 | 76 | 59 |
|  | (55/10) | (69/11) | (69/7) | (52/7) |

*^a^* NMR conversions of carbodiimide, NMR yields of appropriate guanidine/guanidinium are given in parenthesis

**DFT calculations**

All calculations were performed using the Gaussian 16 program.^[[2]](#footnote-2)^ Reaction energy profiles were computed at the B3LYP/cc-pVTZ level of theory^[[3]](#footnote-3),^^[[4]](#footnote-4)^, incorporating solvation effects through the polarizable continuum model (PCM) for toluene^[[5]](#footnote-5)^. Additionally, dispersion corrections were applied using the D3 version of Grimme’s dispersion method^[[6]](#footnote-6)^. Frequency analysis at the same level of theory confirmed that all computed structures correspond to minima on the potential energy surface, with transition states exhibiting a single imaginary frequency.

We performed theoretical calculations for four model cases to evaluate the influence of substituents of amines and carbodiimides on the reaction energy profile (Figure S15). All substituents are aliphatic (R^1^=R^2^=Me; blue profile), all aromatic (R^1^=R^2^=Ph; yellow profile), and two mixed cases — aliphatic amine with aromatic carbodiimide (R^1^=Me, R^2^=Ph; orange profile) and aromatic amine with aliphatic carbodiimide (R^1^=Ph, R^2^=Me; green profile). The mechanism is same to that described in the main part of the paper with the most thermodynamically favored reactions for aliphatic amines (blue and orange).


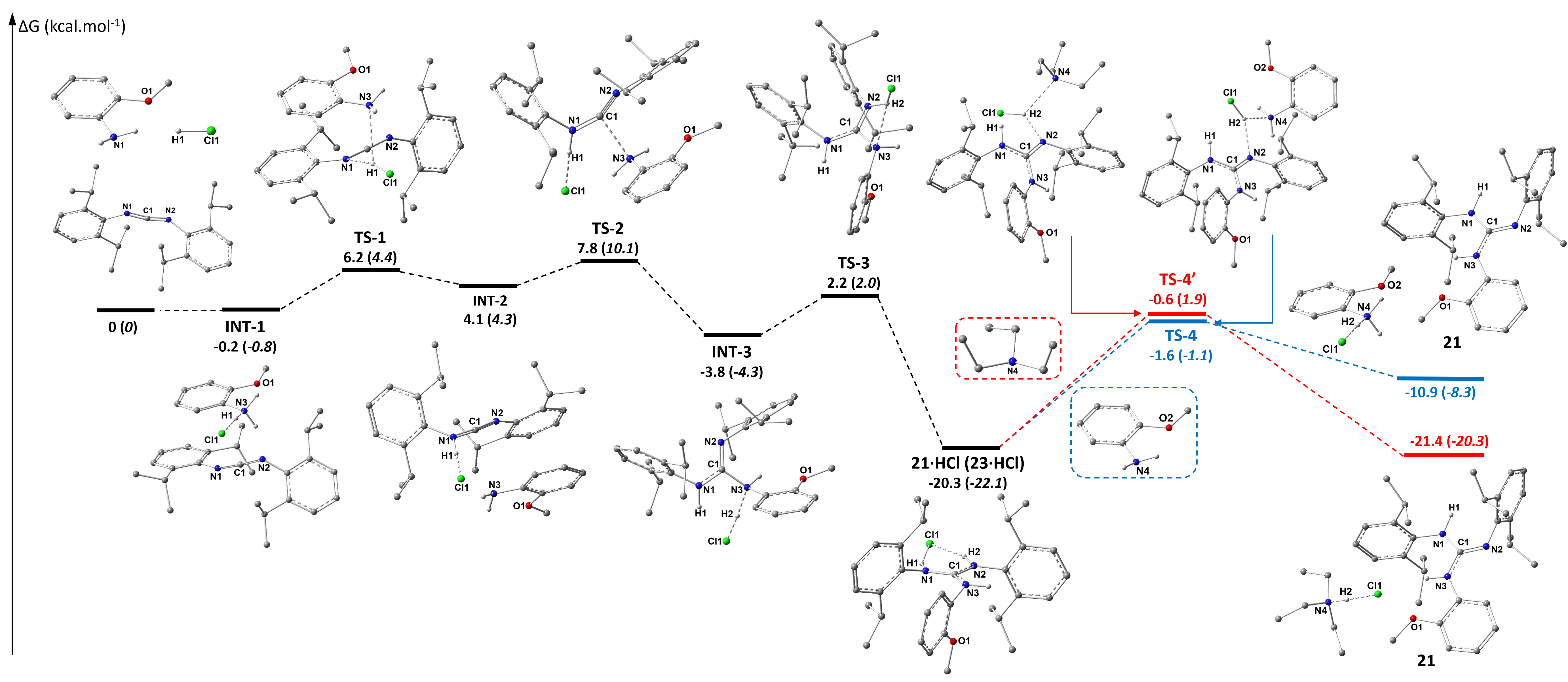


**Figure S14.** The DFT-estimated Gibbs free energy profile (kcal.mol^-1^) for **21** (**23** in italics in parentheses).


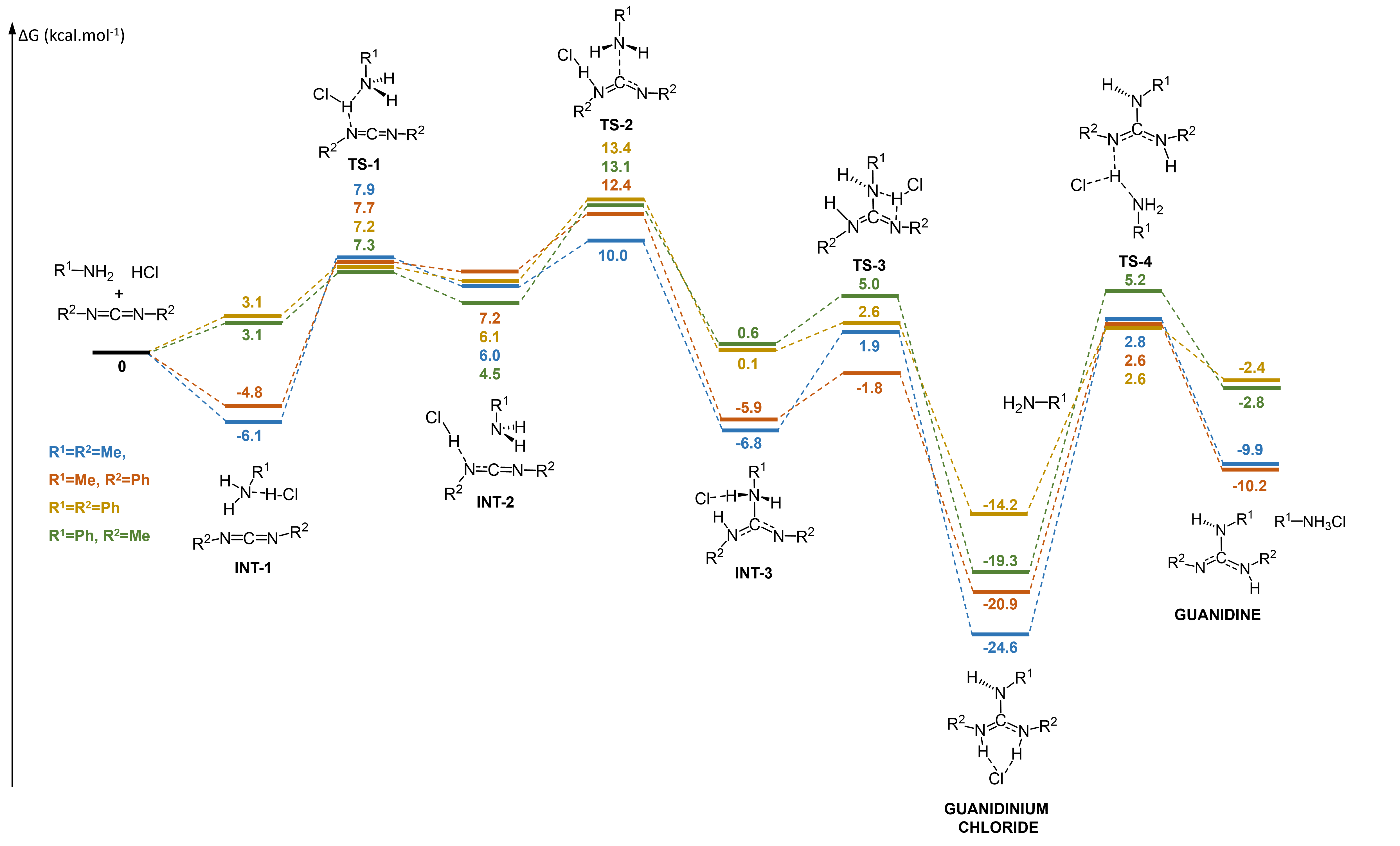


**Figure S15.** The DFT-estimated Gibbs free energy profile (kcal.mol^-1^) for suggested reaction mechanisms of amines and carbodiimides with various substituents (R^1^, R^2^).


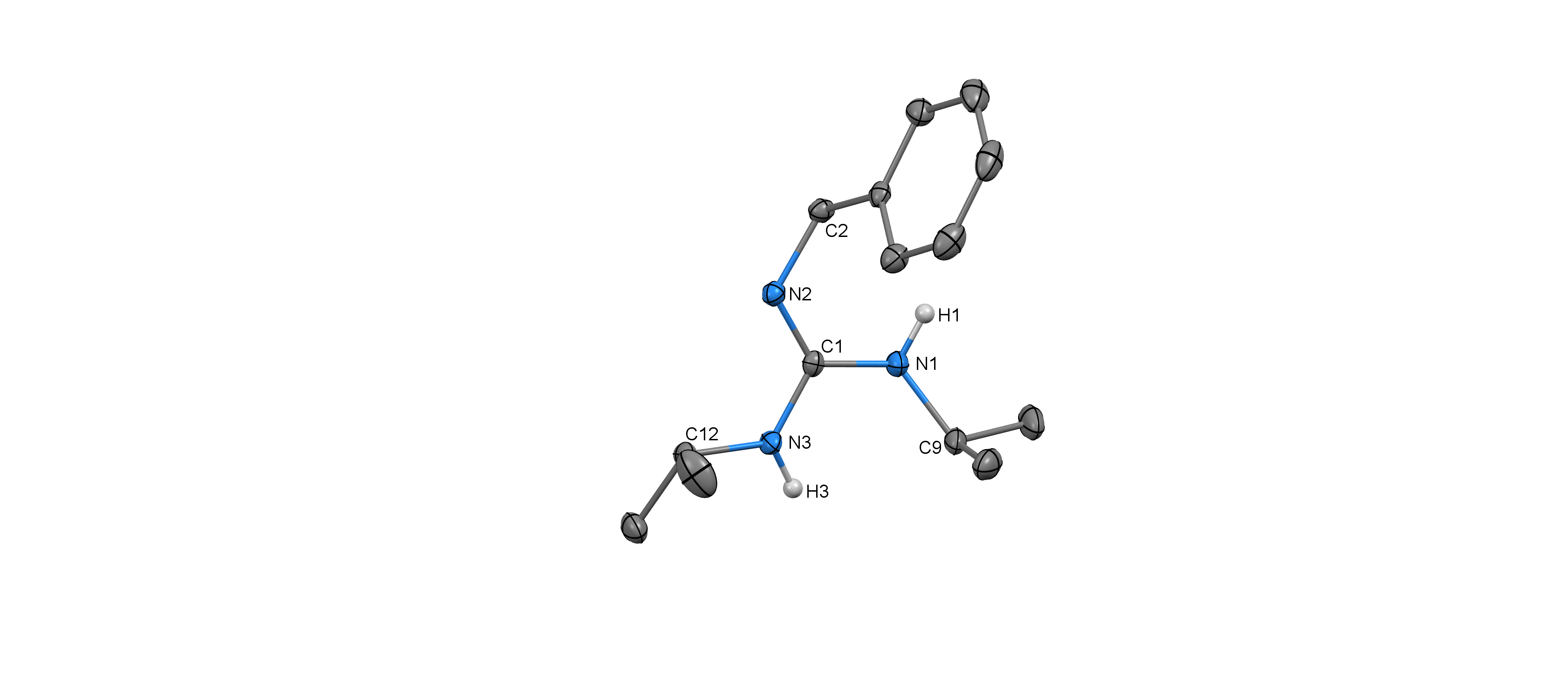


**Figure S16**. Molecular structure of **1** (ORTEP view, 30% probability level). Hydrogen atoms (except of N–H) are omitted for clarity. Selected interatomic distances [Å] and angles [°]: C1–N1 1.3829(16), C1–N2 1.3001(16), C1–N3 1.3585(17), C2–N2 1.4493(16), C9–N1 1.4649(17), C12–N3 1.4580(16), N2–C1–N3 119.79(11), N2–C1–N1 124.32(12), N3–C1–N1 115.85(11).


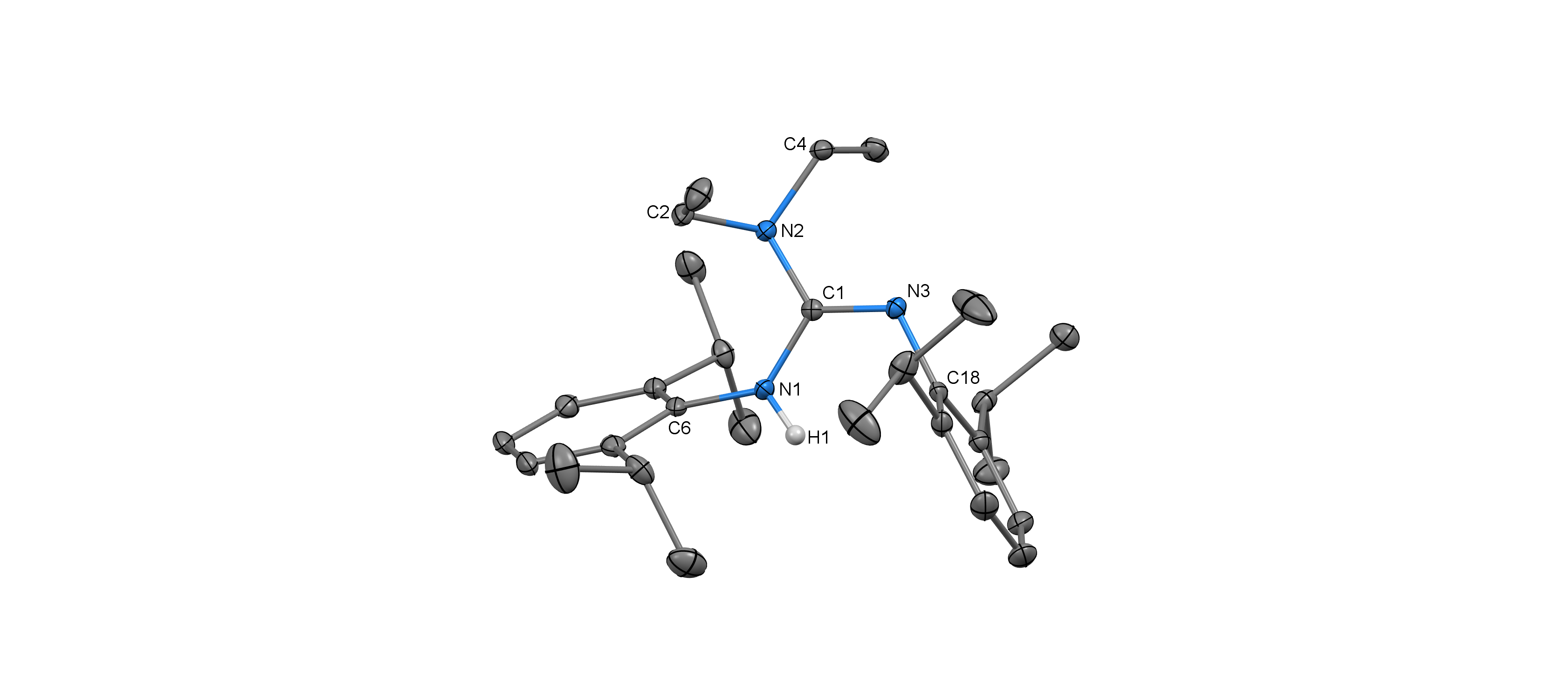


**Figure S17**. Molecular structure of **2** (ORTEP view, 30% probability level). Hydrogen atoms (except of N–H) are omitted for clarity. Selected interatomic distances [Å] and angles [°]: C1–N1 1.384(3), C1–N2 1.392(3), C1–N3 1.288(3), C2–N2 1.473(3), C4–N2 1.467(3), C6–N1 1.427(3), C18–N3 1.422(3), N2–C1–N3 121.15(18), N2–C1–N1 116.70(17), N3–C1–N1 122.14(19).


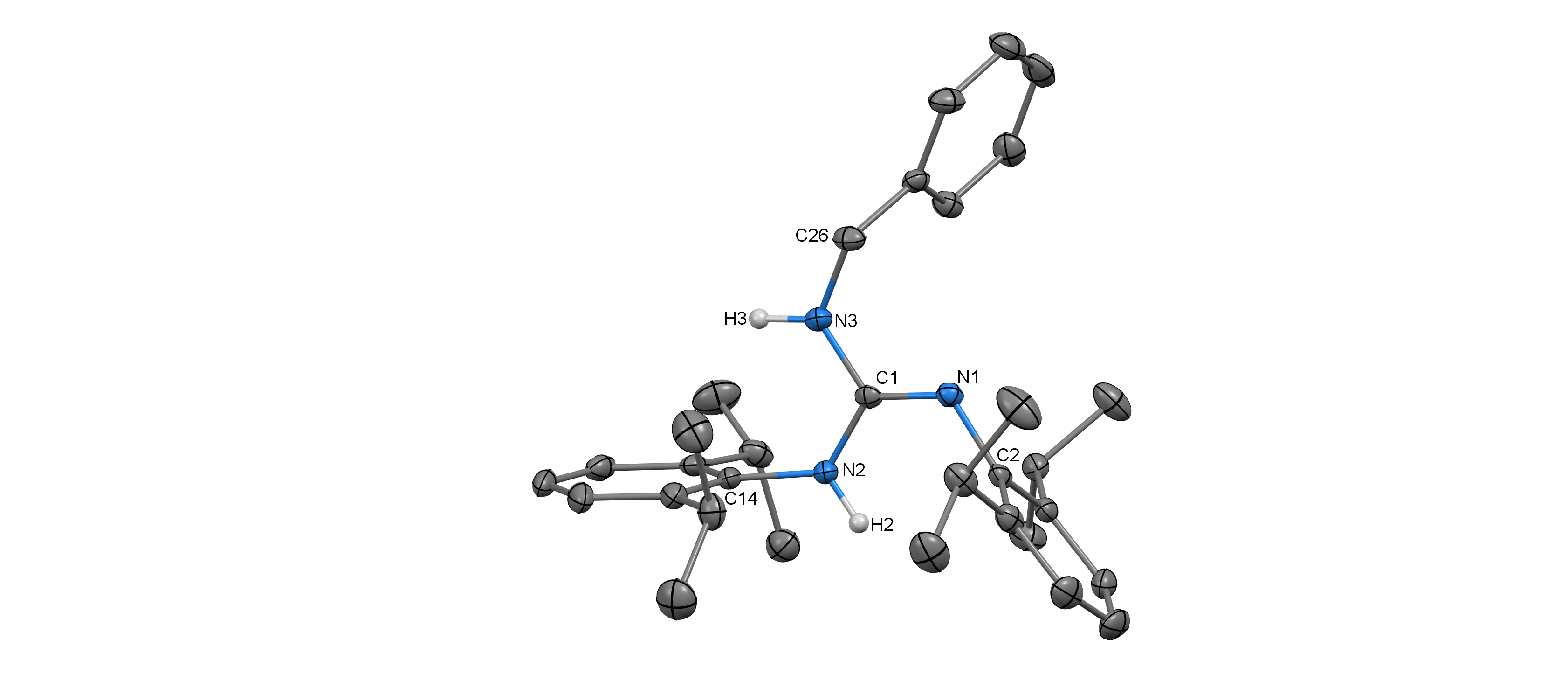


**Figure S18.** Molecular structure of **4** (ORTEP view, 30% probability level). Hydrogen atoms (except of N–H) are omitted for clarity. Selected interatomic distances [Å] and angles [°]: C1–N1 1.284(3), C1–N2 1.382(3), C1–N3 1.364(3), C2–N1 1.414(3), C14–N2 1.439(3), C26–N3 1.447(3), N2–C1–N3 115.05(17), N2–C1–N1 125.11(18), N3–C1–N1 119.80(18).


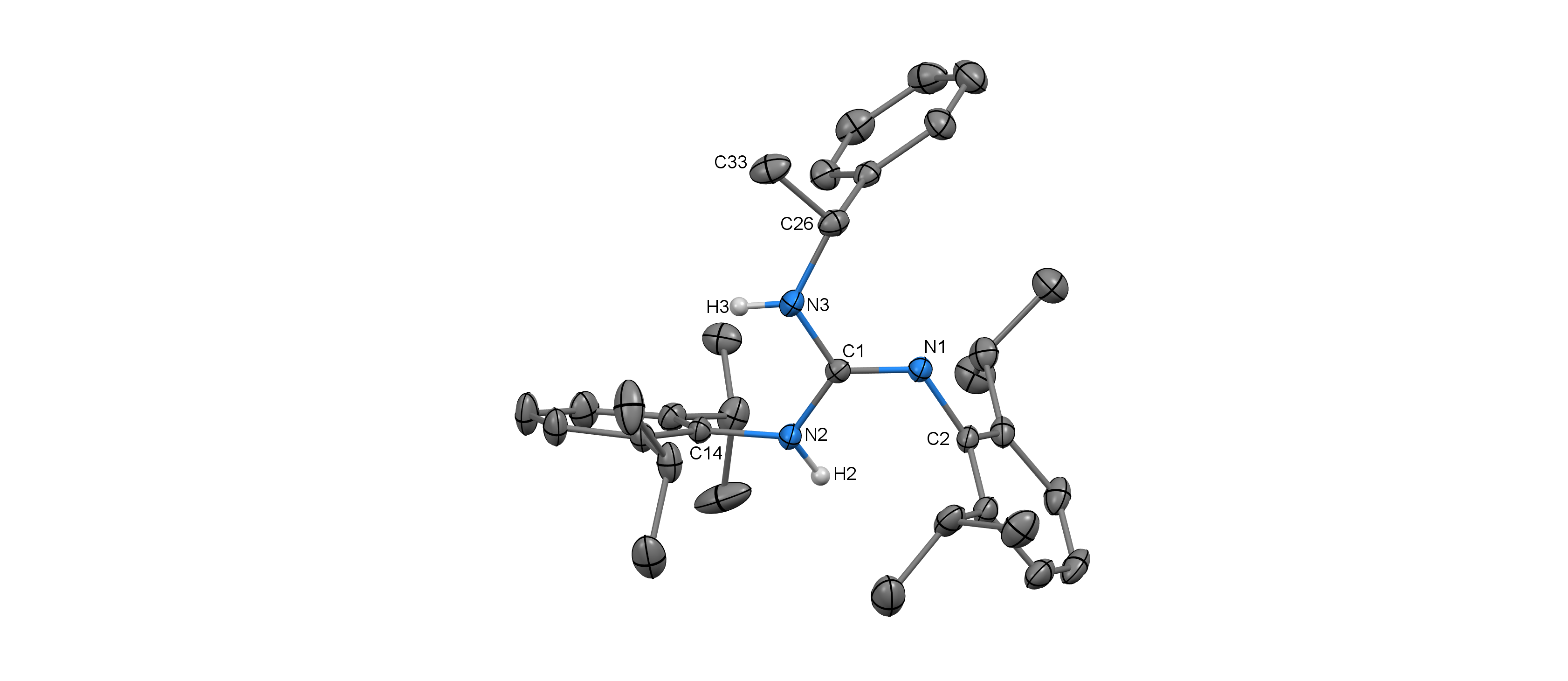


**Figure S19.** Molecular structure of **5** (ORTEP view, 30% probability level). Hydrogen atoms (except of N–H) are omitted for clarity. Selected interatomic distances [Å] and angles [°]: C1–N1 1.288(3), C1–N2 1.370(3), C1–N3 1.379(3), C2–N1 1.417(3), C14–N2 1.443(3), C26–N3 1.456(4), C26–C33 1.519(4), N2–C1–N3 115.4(2), N2–C1–N1 125.5(2), N3–C1–N1 119.0(2).


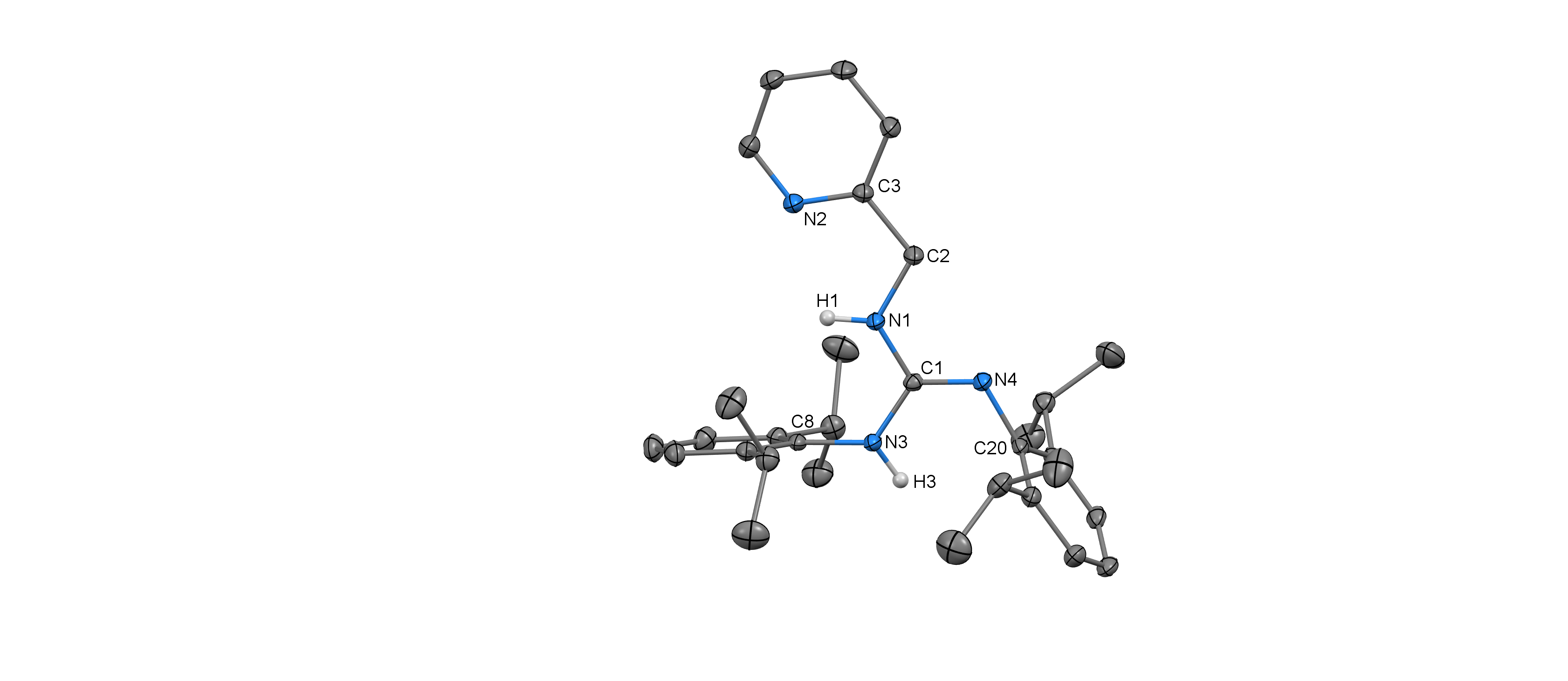

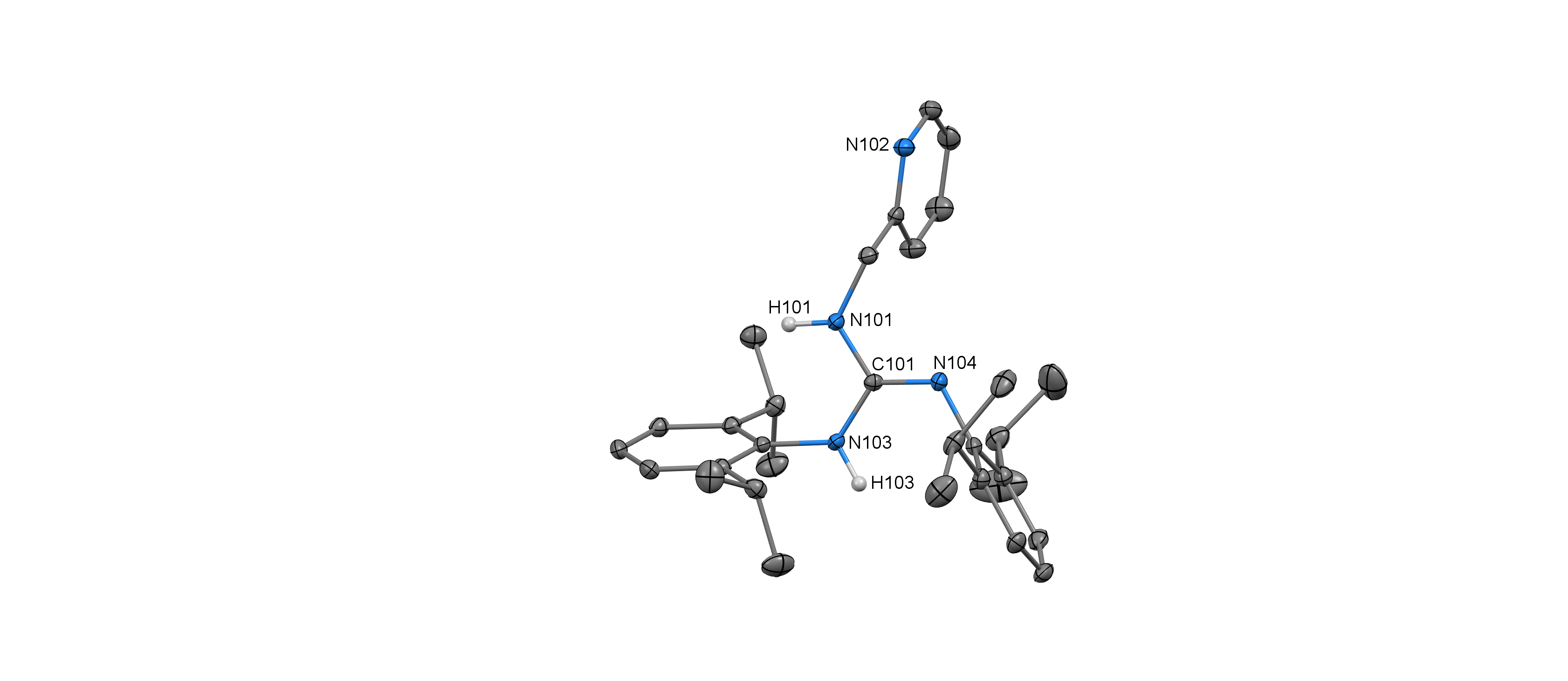


**Figure S20.** Molecular structure of two independent molecules of **6** (ORTEP view, 30% probability level). Hydrogen atoms (except of N–H) are omitted for clarity. Selected interatomic distances [Å] and angles [°], appropriate values of the second independent molecule are given in italics: C1–N1 1.363(3), *1.367(3)*; C1–N4 1.285(3), *1.285(3)*; C1–N3 1.378(3), *1.380(3)*; C2–N1 1.441(3), *1.448(3)*; C8–N3 1.436(3), *1.431(3)*; C20–N4 1.416(3), *1.411(3)*; N4–C1–N3 124.1(2), *124.17(19)*; N4–C1–N1 119.8(2), *120.55(19)*; N3–C1–N1 116.02(19), *115.28(18)*.


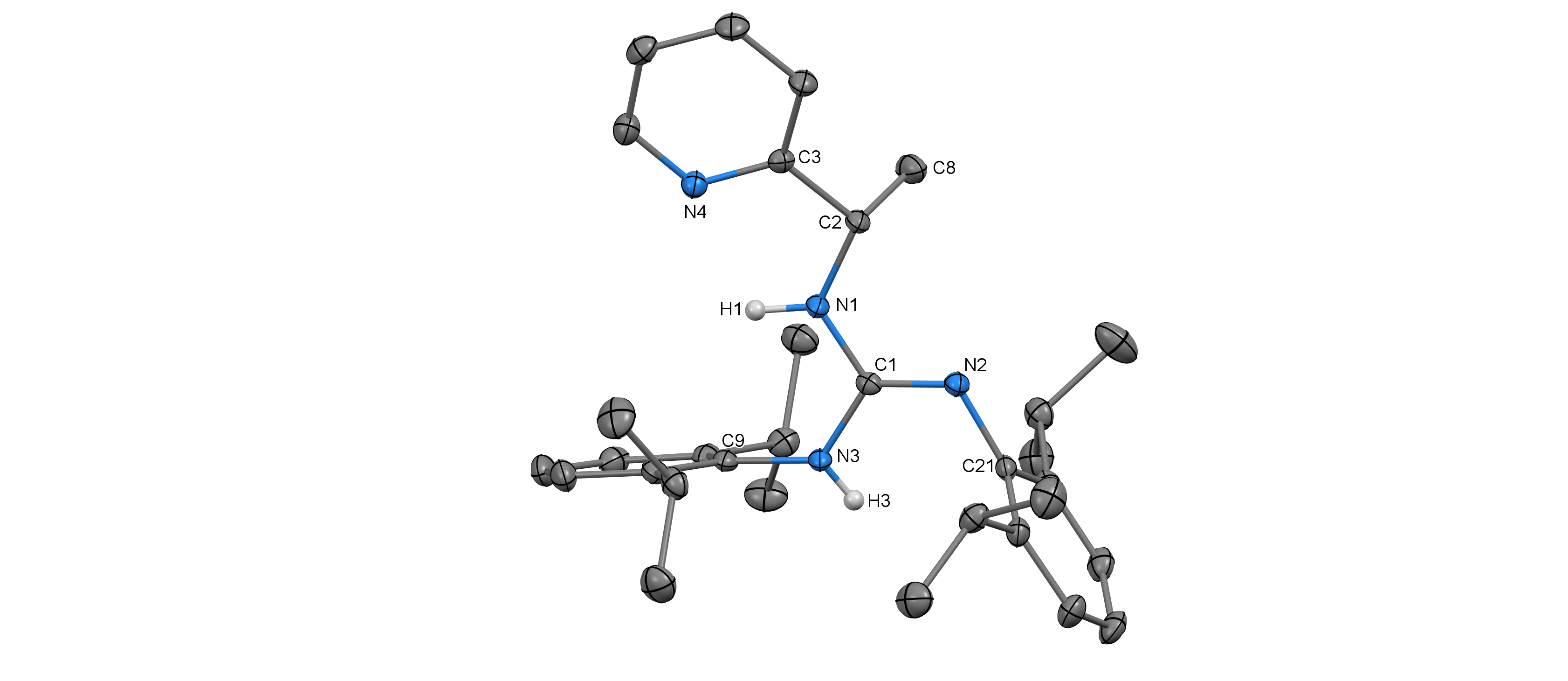


**Figure S21.** Molecular structure of **7** (ORTEP view, 30% probability level). Hydrogen atoms (except of N–H) are omitted for clarity. Selected interatomic distances [Å] and angles [°], appropriate values of the second independent molecule are given in italics: C1–N1 1.356(3), *1.356(3)*; C1–N2 1.286(3), *1.284(3)*; C1–N3 1.380(3), *1.374(3)*; C2–N1 1.448(3), *1.447(3)*; C9–N3 1.431(3), *1.439(3)*; C21–N2 1.421(3), *1.424(3)*; N2–C1–N3 123.49(19), *123.92(19)*; N2–C1–N1 121.18(19), *120.76(19)*; N3–C1–N1 115.31(18), *115.32(19)*.


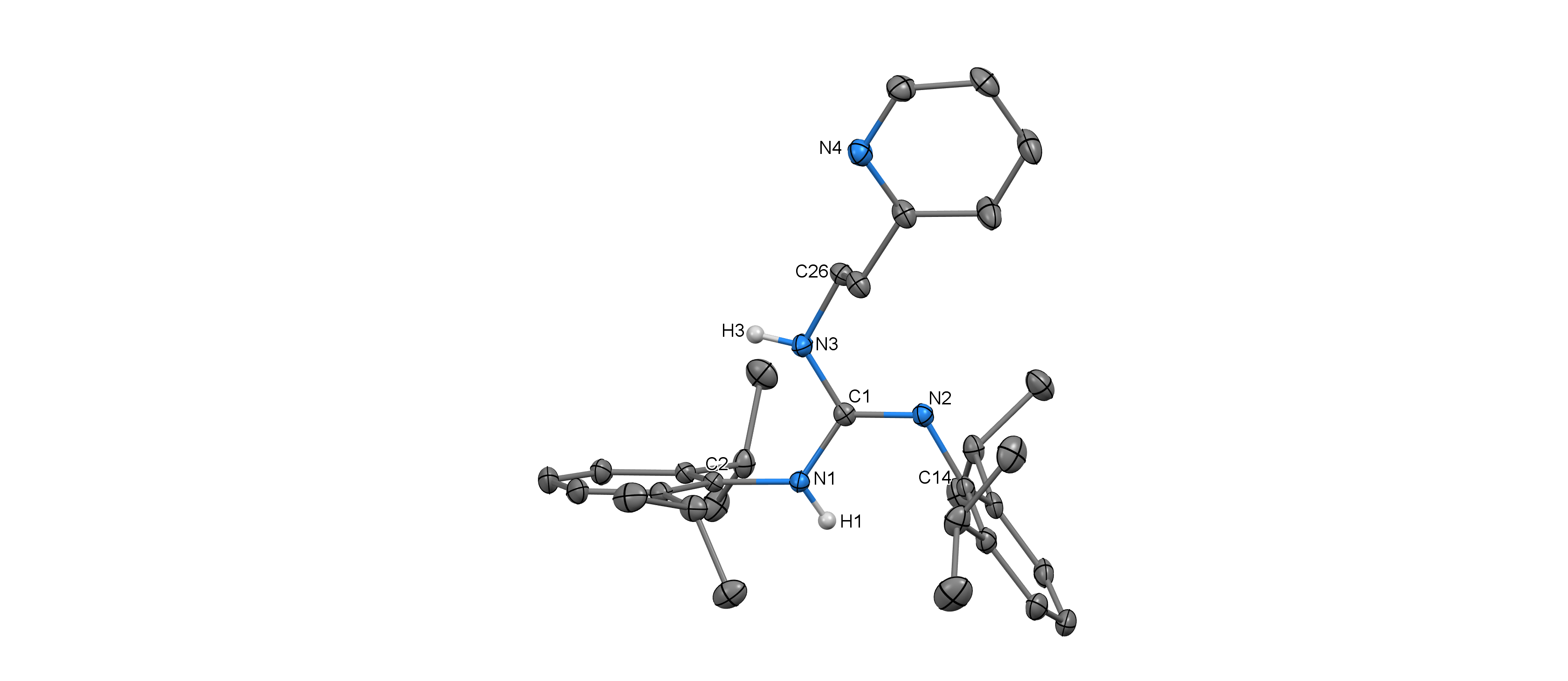


**Figure S22.** Molecular structure of **8** (ORTEP view, 30% probability level). Hydrogen atoms (except of N–H) are omitted for clarity. Selected interatomic distances [Å] and angles [°]: C1–N1 1.3769(16), C1–N2 1.2930(16), C1–N3 1.3567(17), C2–N1 1.4343(16), C14–N2 1.4171(17), C26–N3 1.4517(17), N2–C1–N3 120.50(12), N2–C1–N1 123.39(12), N3–C1–N1 116.09(11).


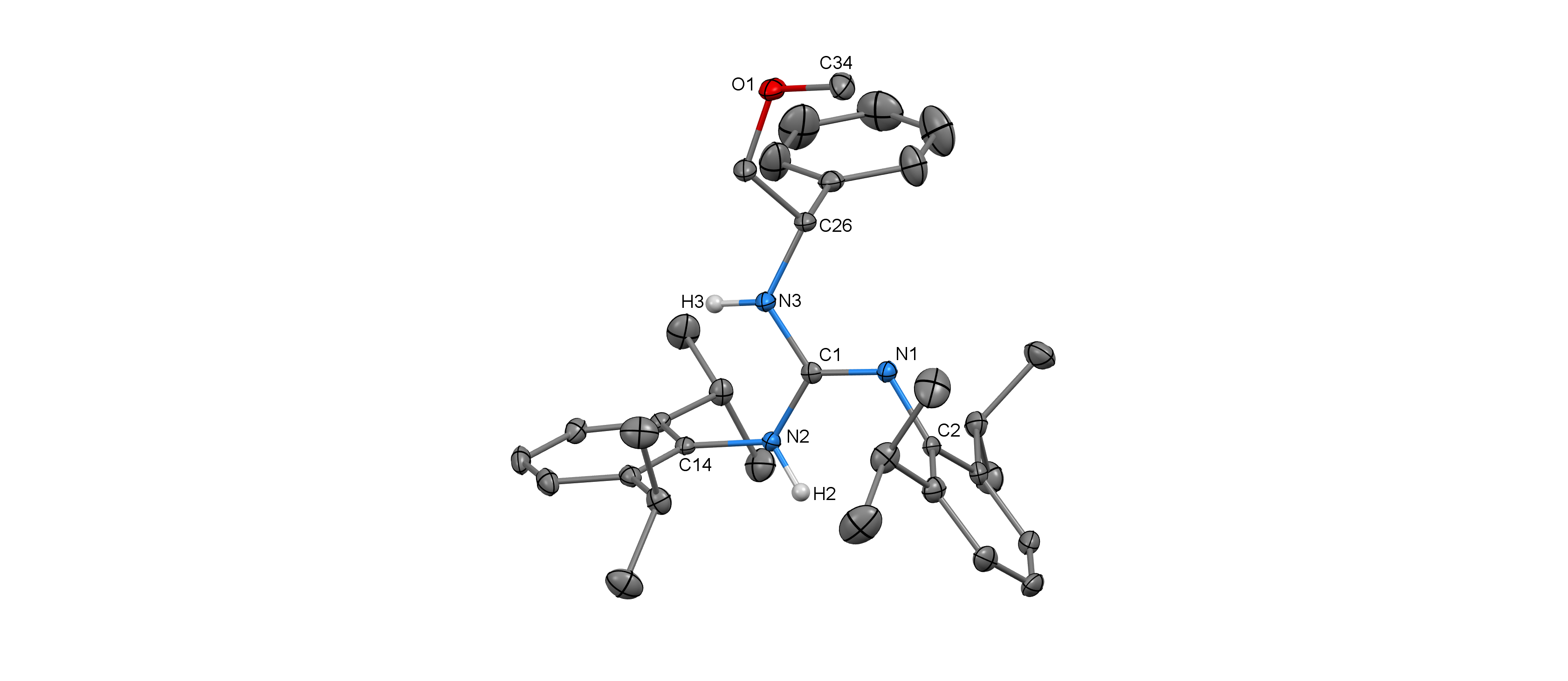


**Figure S23.** Molecular structure of **10** (ORTEP view, 30% probability level). Hydrogen atoms (except of N–H) are omitted for clarity. Selected interatomic distances [Å] and angles [°]: C1–N1 1.281(3), C1–N2 1.368(3), C1–N3 1.371(3), C2–N1 1.415(3), C14–N2 1.429(3), C26–N3 1.457(3), N2–C1–N3 115.5(2), N2–C1–N1 124.7(2), N3–C1–N1 119.8(2).


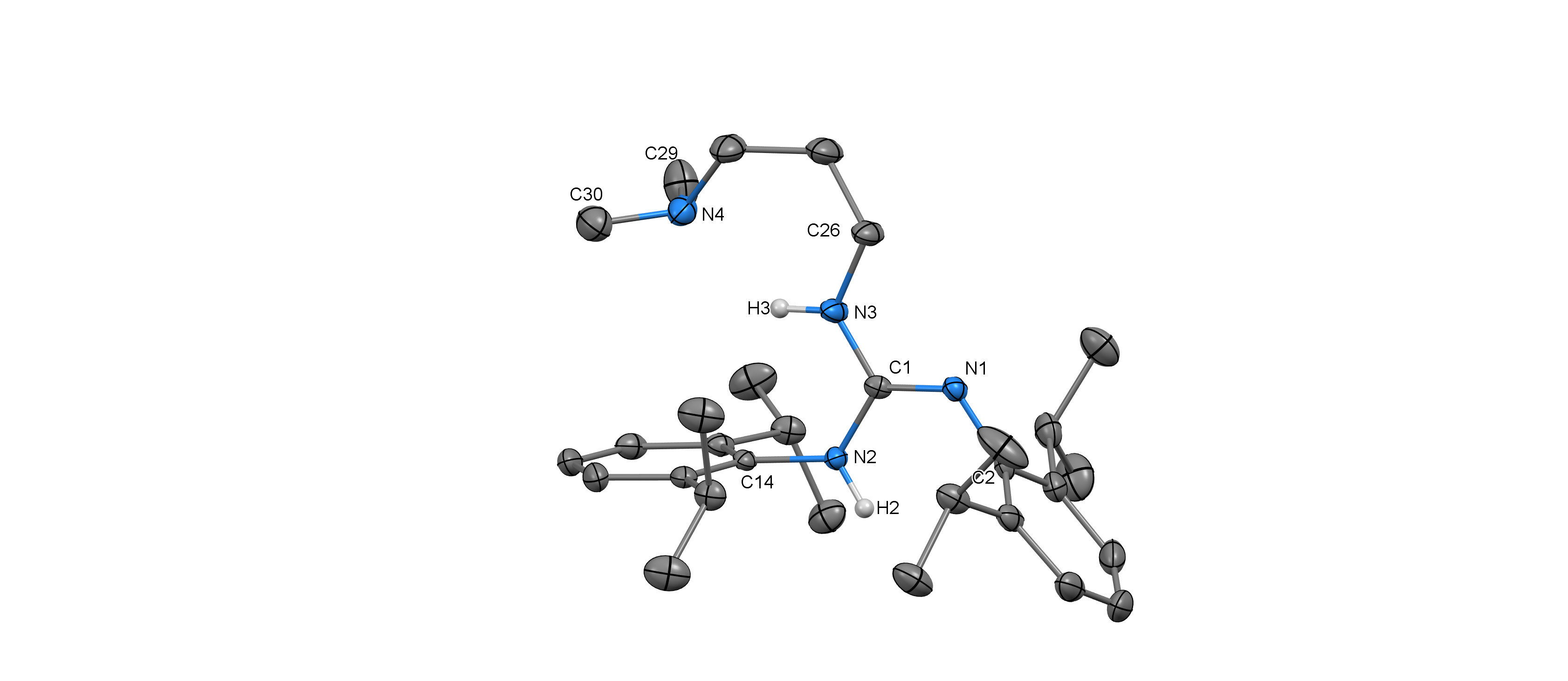


**Figure S24.** Molecular structure of **12** (ORTEP view, 30% probability level). Hydrogen atoms (except of N–H) are omitted for clarity. Selected interatomic distances [Å] and angles [°]: C1–N1 1.288(3), C1–N2 1.380(3), C1–N3 1.353(3), C2–N1 1.410(3), C14–N2 1.440(3), C26–N3 1.449(4), N3–N4 2.856(3), N2–C1–N3 115.5(2), N2–C1–N1 124.4(2), N3–C1–N1 120.1(2).


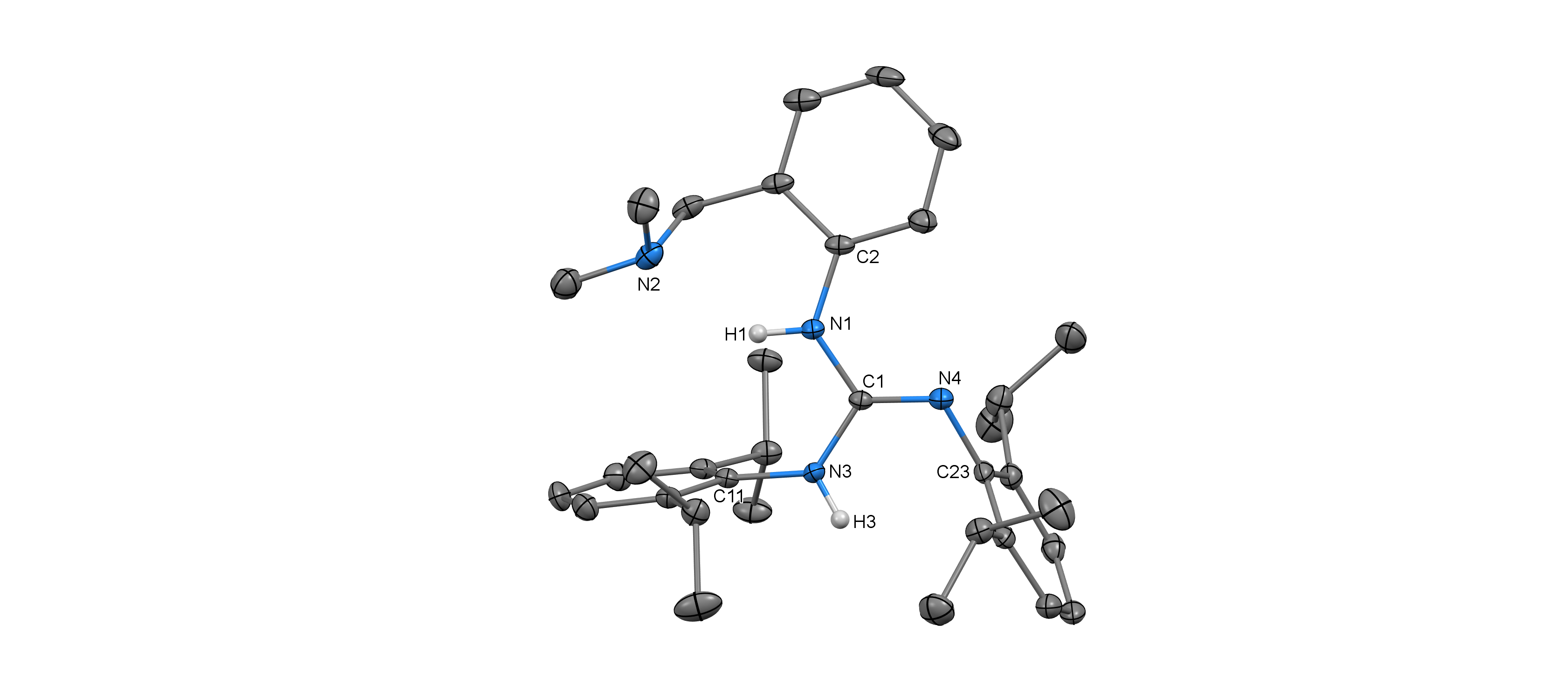


**Figure S25.** Molecular structure of **13** (ORTEP view, 30% probability level). Hydrogen atoms (except of N–H) are omitted for clarity. Selected interatomic distances [Å] and angles [°]: C1–N1 1.378(4), C1–N3 1.381(4), C1–N4 1.278(4), C2–N1 1.408(4), C11–N3 1.434(4), C23–N4 1.420(4), N1–N2 2.888(4), N4–C1–N3 123.6(3), N4–C1–N1 122.8(3), N3–C1–N1 113.6(3).


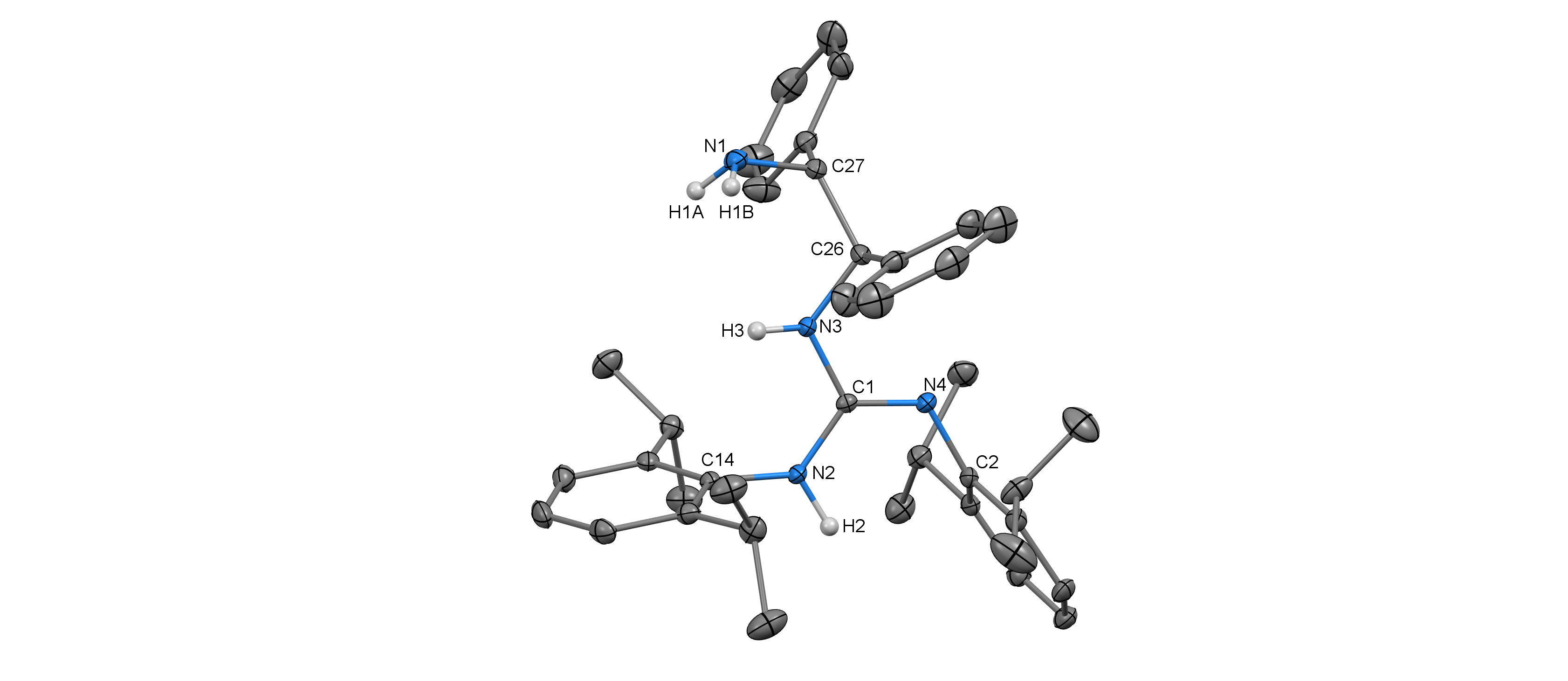


**Figure S26.** Molecular structure of **14** (ORTEP view, 30% probability level). Hydrogen atoms (except of N–H) are omitted for clarity. Selected interatomic distances [Å] and angles [°]: C1–N2 1.368(3), C1–N3 1.386(3), C1–N4 1.286(3), C2–N4 1.411(3), C14–N2 1.436(3), C26–N3 1.459(3), N4–C1–N3 118.3(2), N4–C1–N2 124.6(2), N2–C1–N3 117.1(2).


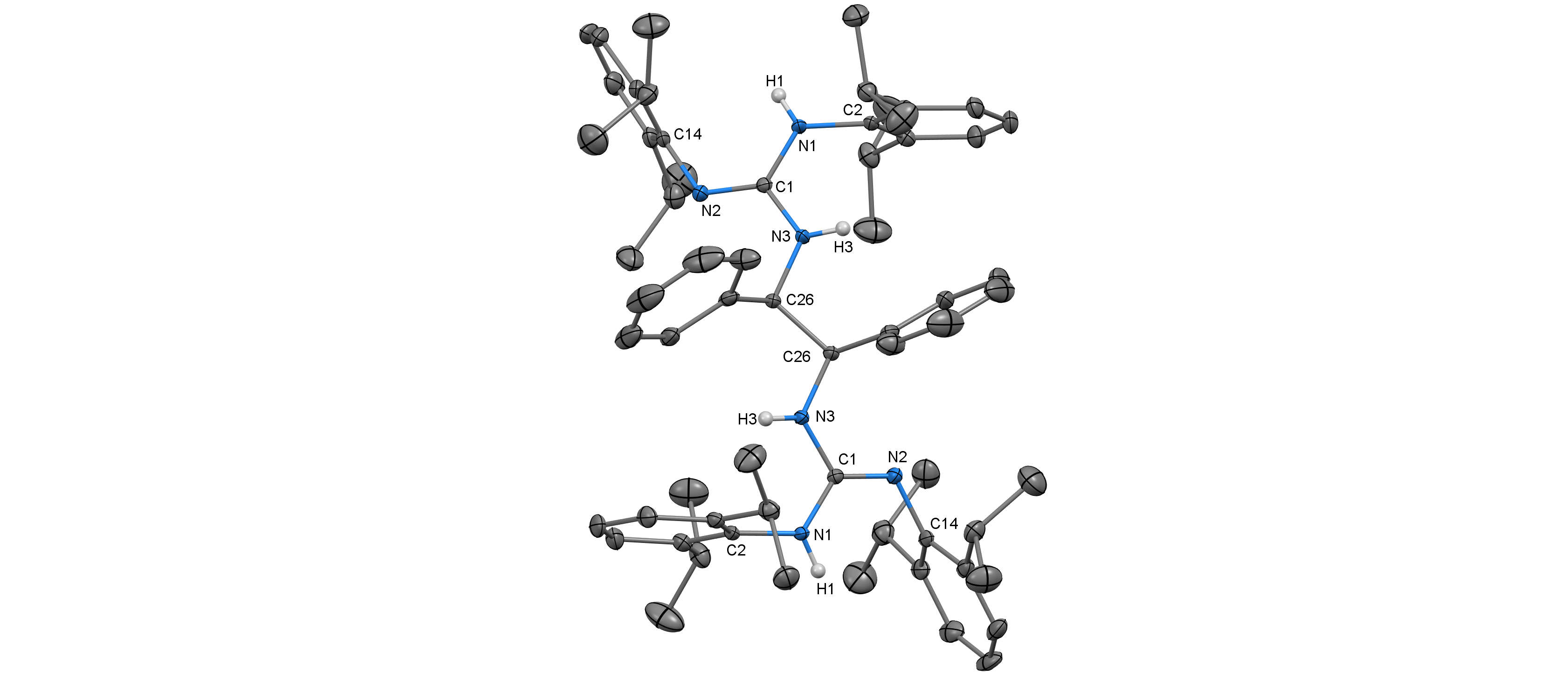


**Figure S27.** Molecular structure of **15** (ORTEP view, 30% probability level). Hydrogen atoms (except of N–H) are omitted for clarity. Selected interatomic distances [Å] and angles [°]: C1–N1 1.382(2), C1–N2 1.285(2), C1–N3 1.3645(19), C2–N1 1.431(2), C14–N2 1.415(2), C26–N3 1.4484(19), N1–C1–N2 125.27(13), N1–C1–N3 114.67(14), N2–C1–N3 120.06(14).


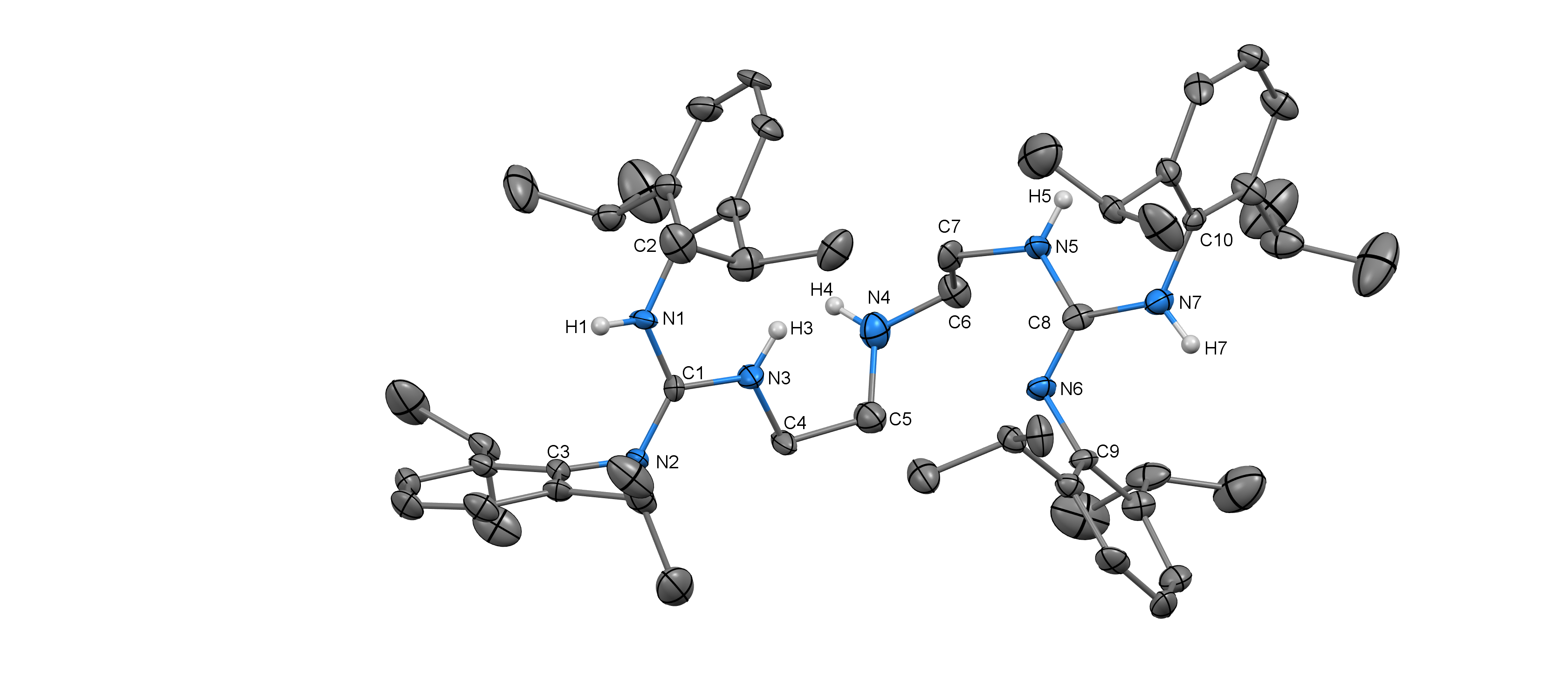


**Figure S28.** Molecular structure of **17** (ORTEP view, 30% probability level). Hydrogen atoms (except of N–H) are omitted for clarity. Selected interatomic distances [Å] and angles [°], appropriate values of the second independent molecule are given in italics: C1–N1 1.379(8), *1.388(9)*; C1–N2 1.276(8), *1.279(9)*; C1–N3 1.385(8), *1.355(9)*; C8–N5 1.385(8), *1.340(8)*; C8–N6 1.271(8), *1.287(8)*; C8–N7 1.378(9), *1.365(8)*; N1–C2 1.443(8), *1.434(10)*; N2–C3 1.435(8), *1.443(9)*; N3–C4 1.434(8), *1.481(9)*; N5–C7 1.469(8), *1.447(8)*; N6–C9 1.397(8), *1.405(8)*; N7–C10 1.431(8), *1.429(8)*; N1–C1–N2 124.5(6), *125.0(6)*; N1–C1–N3 116.2(5), *114.6(6)*; N2–C1–N3 119.4(5), *120.4(6)*, N5–C8–N6 120.2(6), *120.2(6)*; N5–C8–N7 113.7(6), *114.7(6)*; N6–C8–N7 126.0(6), *125.0(6)*.


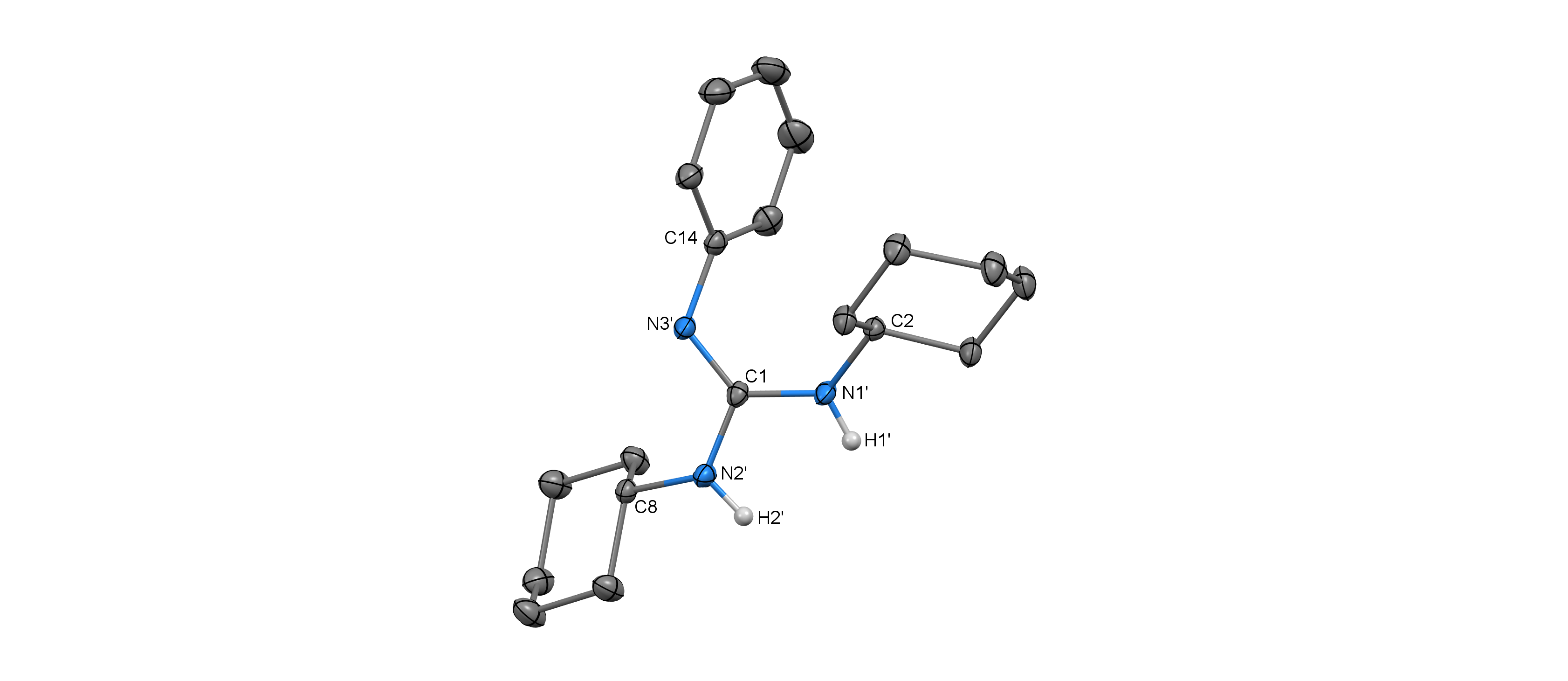


**Figure S29.** Molecular structure of **18** (ORTEP view, 30% probability level). Hydrogen atoms (except of N–H) are omitted for clarity. Selected interatomic distances [Å] and angles [°], appropriate values of the second independent molecule are given in italics: C1–N1’ 1.3633(18), *1.3733(18)*; C1–N2’ 1.3664(19), *1.360(2)*; C1–N3’ 1.3089(18), *1.3035(18)*; C2–N1’ 1.4602(18), *1.4579(19)*; C8–N2’ 1.4664(18), *1.4583(18)*; C14–N3’ 1.409(2), *1.4114(19)*; N1’–C1–N2’ 113.46(13), *113.63(13)*; N1’–C1–N3’ 127.10(14), *127.14(14)*; N2’–C1–N3’ 119.39(13), *119.20(13)*.


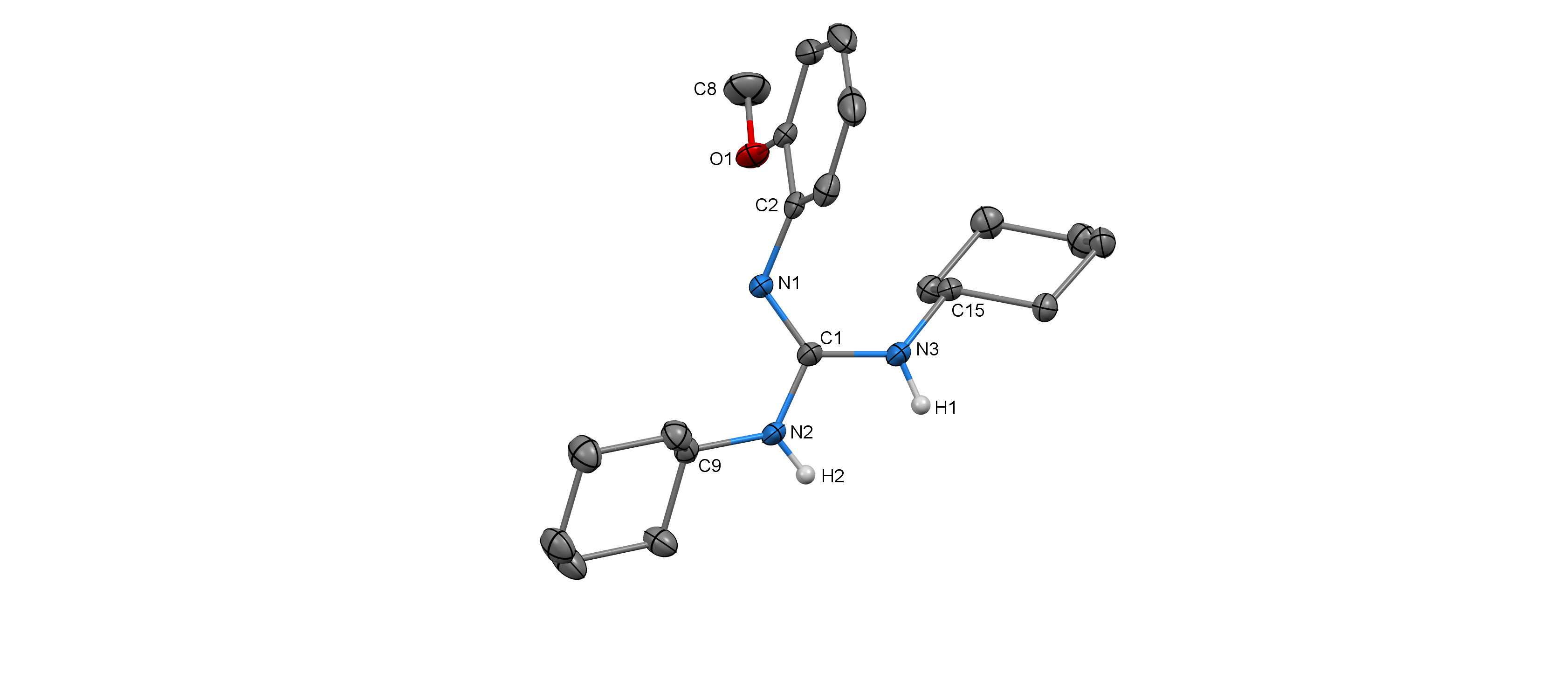


**Figure S30.** Molecular structure of **19** (ORTEP view, 30% probability level). Hydrogen atoms (except of N–H) are omitted for clarity. Selected interatomic distances [Å] and angles [°]: C1–N1 1.293(5), C1–N2 1.348(4), C1–N3 1.366(4), C2–N1 1.406(6), C9–N2 1.444(4), C15–N3 1.467(4), N1–C1–N2 119.7(3), N1–C1–N3 125.6(3), N2–C1–N3 114.5(2).


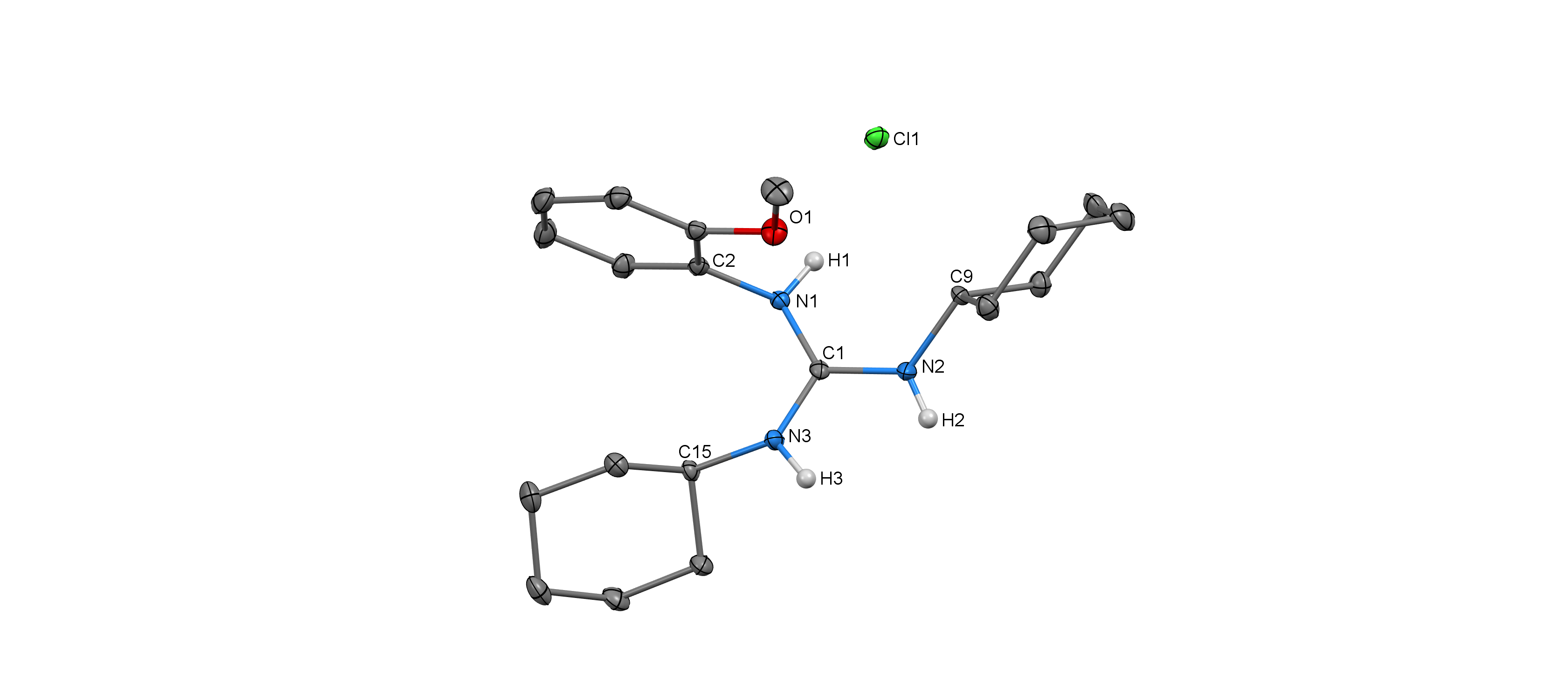


**Figure S31.** Molecular structure of **19**^.^HCl (ORTEP view, 30% probability level). Hydrogen atoms (except of N–H) are omitted for clarity. Selected interatomic distances [Å] and angles [°]: C1–N1 1.3481(17), C1–N2 1.3348(18), C1–N3 1.3281(18), C2–N1 1.4323(17), C9–N2 1.4675(18), C15–N3 1.4676(17), N1–Cl1 3.1676(13), N2–Cl1 3.4059(14), N3–Cl1 3.1735(12), N1–C1–N2 120.38(13), N1–C1–N3 121.60(13), N2–C1–N3 117.98(13).


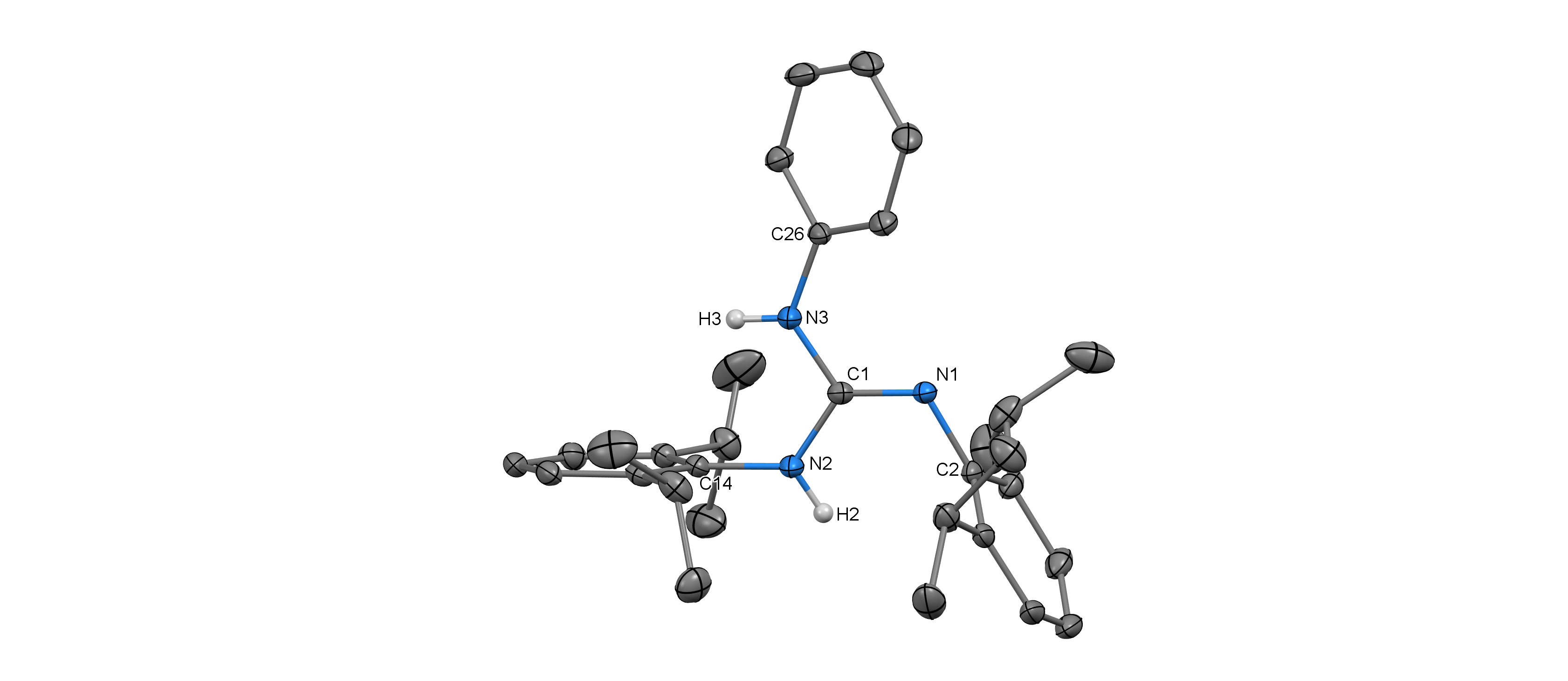

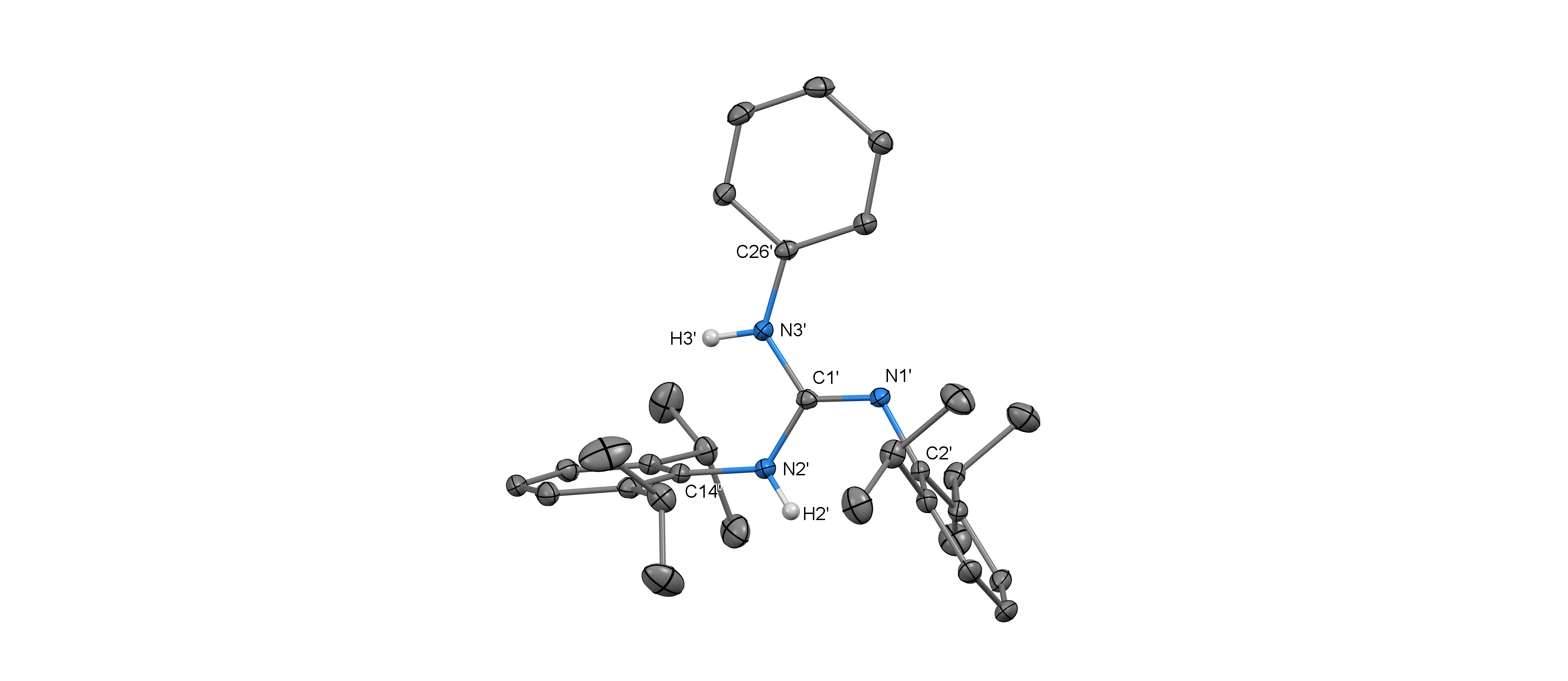


**Figure S32.** Molecular structure of two independent molecules of **20** (ORTEP view, 30% probability level). Hydrogen atoms (except of N–H) are omitted for clarity. Selected interatomic distances [Å] and angles [°], appropriate values of the second independent molecule are given in italics: C1–N1 1.282(2), *1.281(2)*; C1–N2 1.377(2), *1.386(2)*; C1–N3 1.376(2), *1.375(2)*; C2–N1 1.419(2), *1.417(2)*; C14–N2 1.431(2), *1.434(2)*; C26–N3 1.408(2), *1.411(2)*; N1–C1–N2 125.03(16), *124.67(16)*; N1–C1–N3 121.75(15), *122.12(15)*; N2–C1–N3 113.23(16), *113.19(15)*; C1–N3–C26–C27 -39.5(3), *19.1(3)*.


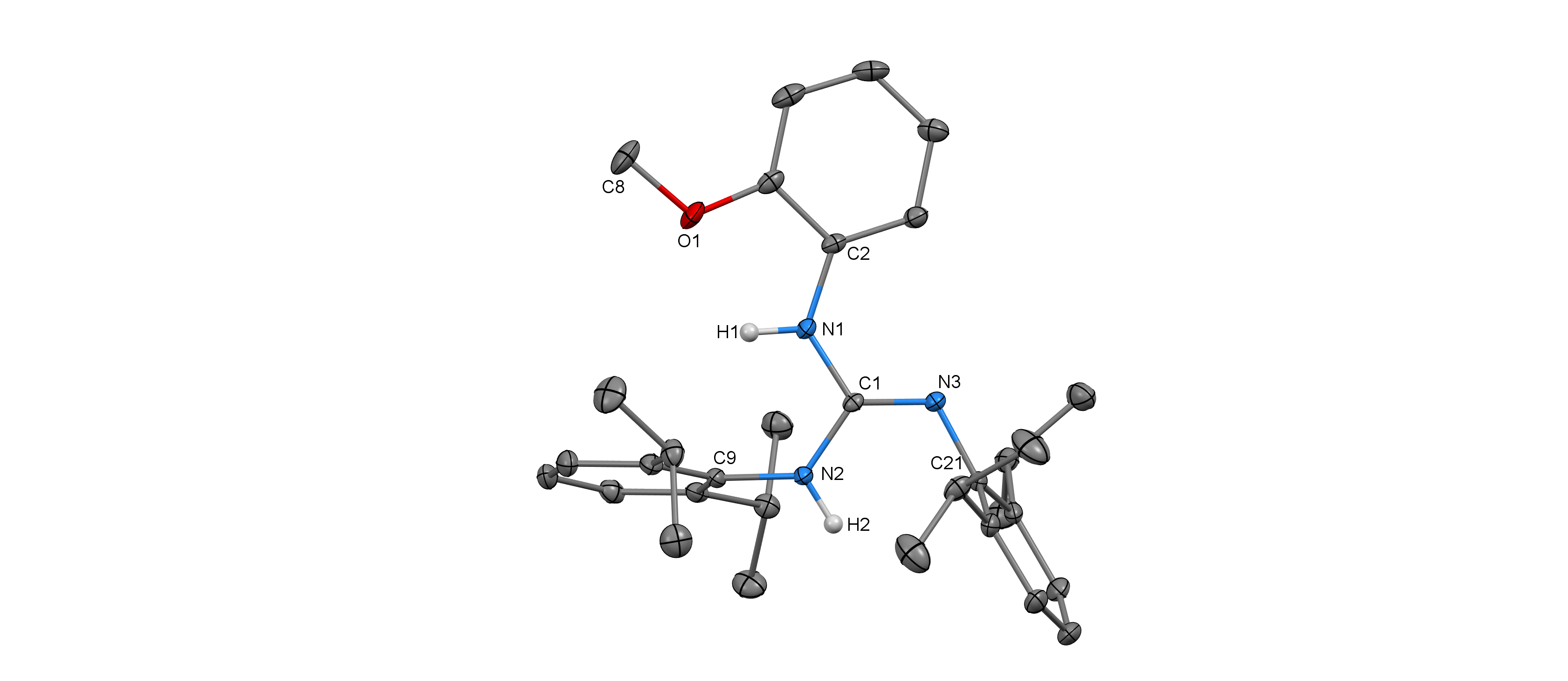


**Figure S33.** Molecular structure of **21** (ORTEP view, 30% probability level). Hydrogen atoms (except of N–H) are omitted for clarity. Selected interatomic distances [Å] and angles [°]: C1–N1 1.371(2), C1–N2 1.390(2), C1–N3 1.284(2), C2–N1 1.402(2), C9–N2 1.436(2), C21–N3 1.417(2), N1–O1 2.564(2), N1–C1–N2 113.18(15), N1–C1–N3 122.04(16), N2–C1–N3 124.74(16).


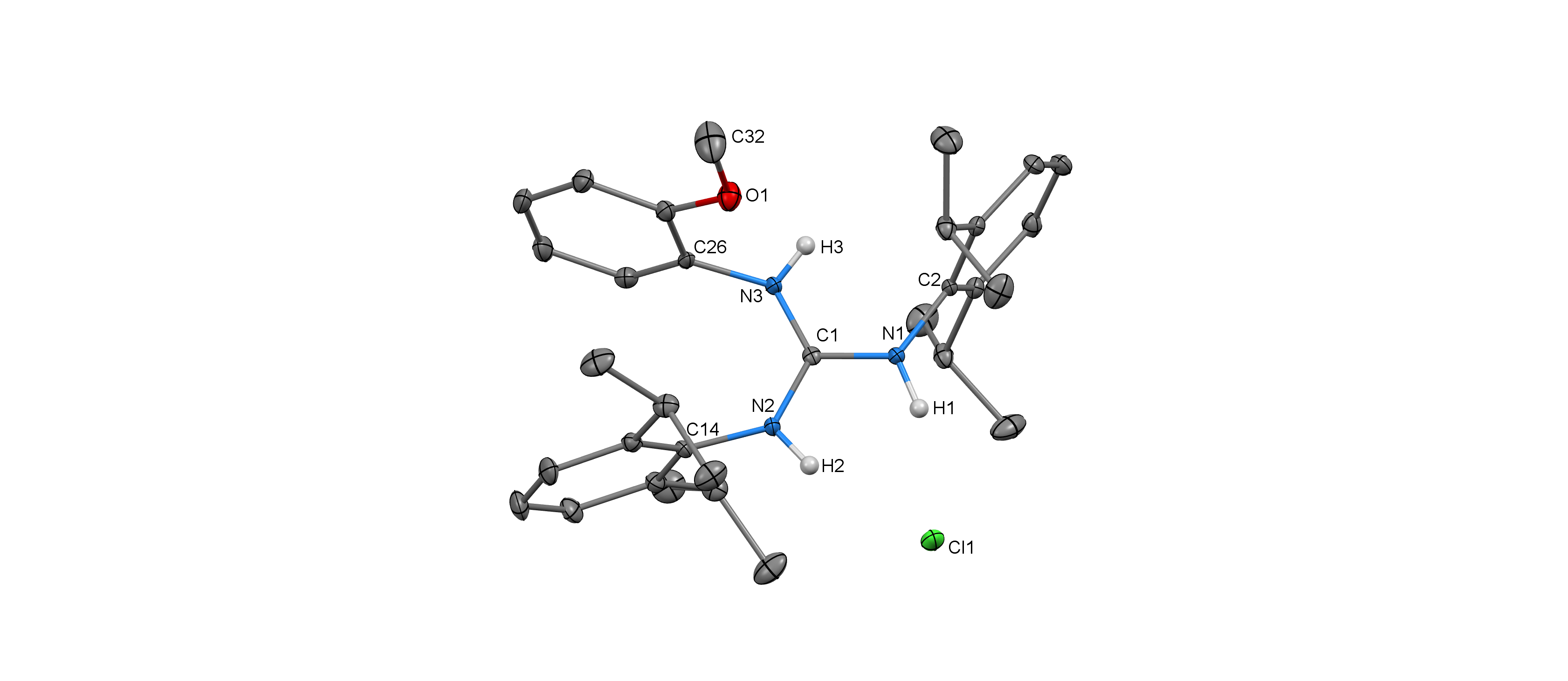


**Figure S34.** Molecular structure of **21**^.^HCl (ORTEP view, 30% probability level). Hydrogen atoms (except of N–H) are omitted for clarity. Selected interatomic distances [Å] and angles [°]: C1–N1 1.331(3), C1–N2 1.327(3), C1–N3 1.344(3), C2–N1 1.442(3), C14–N2 1.444(3), C26–N3 1.440(3), N1–Cl1 3.068(2), N2–Cl1 3.140(2), N1–C1–N2 116.8(2), N1–C1–N3 118.2(2), N2–C1–N3 125.0(2).


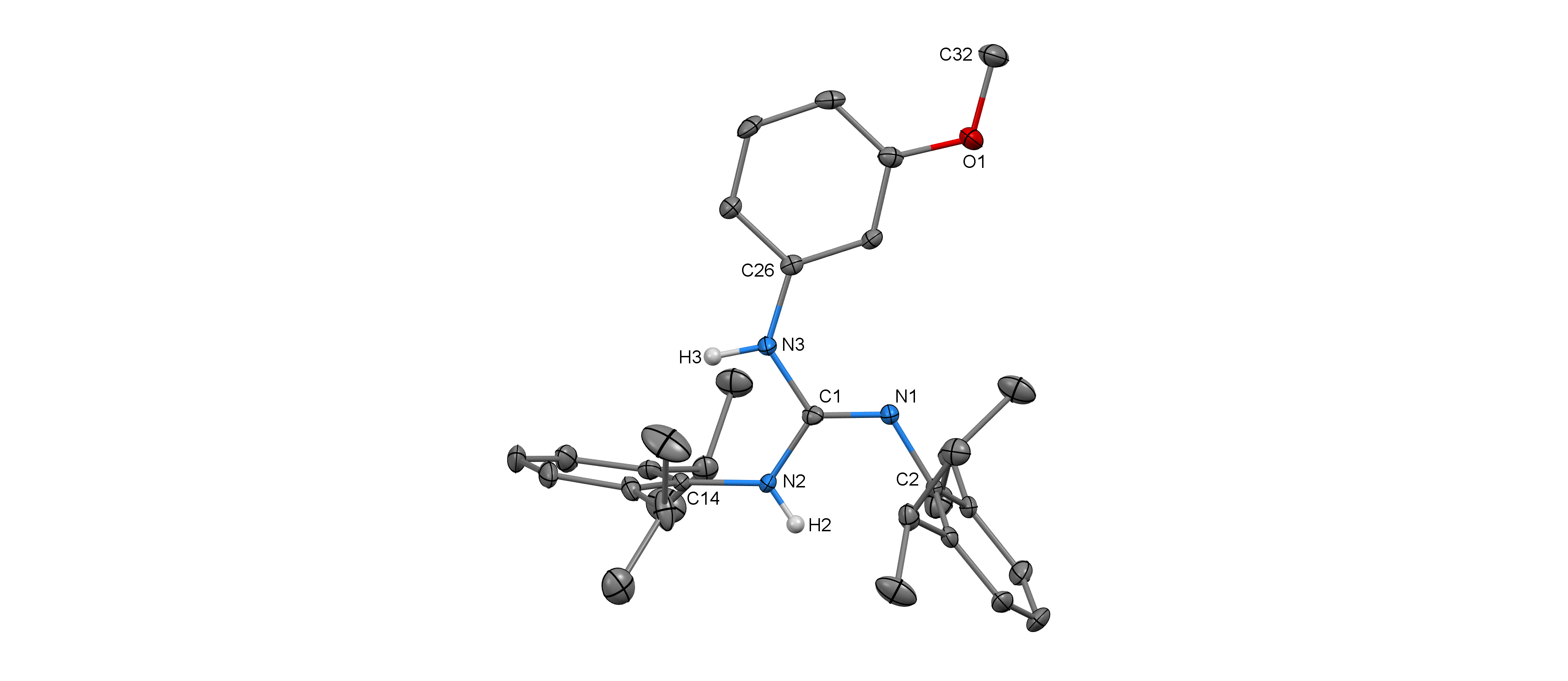


**Figure S35.** Molecular structure of **22** (ORTEP view, 30% probability level). Hydrogen atoms (except of N–H) are omitted for clarity. Selected interatomic distances [Å] and angles [°]: C1–N1 1.279(4), C1–N2 1.378(5), C1–N3 1.377(4), C2–N1 1.423(4), C14–N2 1.441(4), C26–N3 1.399(5), N1–C1–N2 125.2(3), N1–C1–N3 121.2(3), N2–C1–N3 113.6(3).


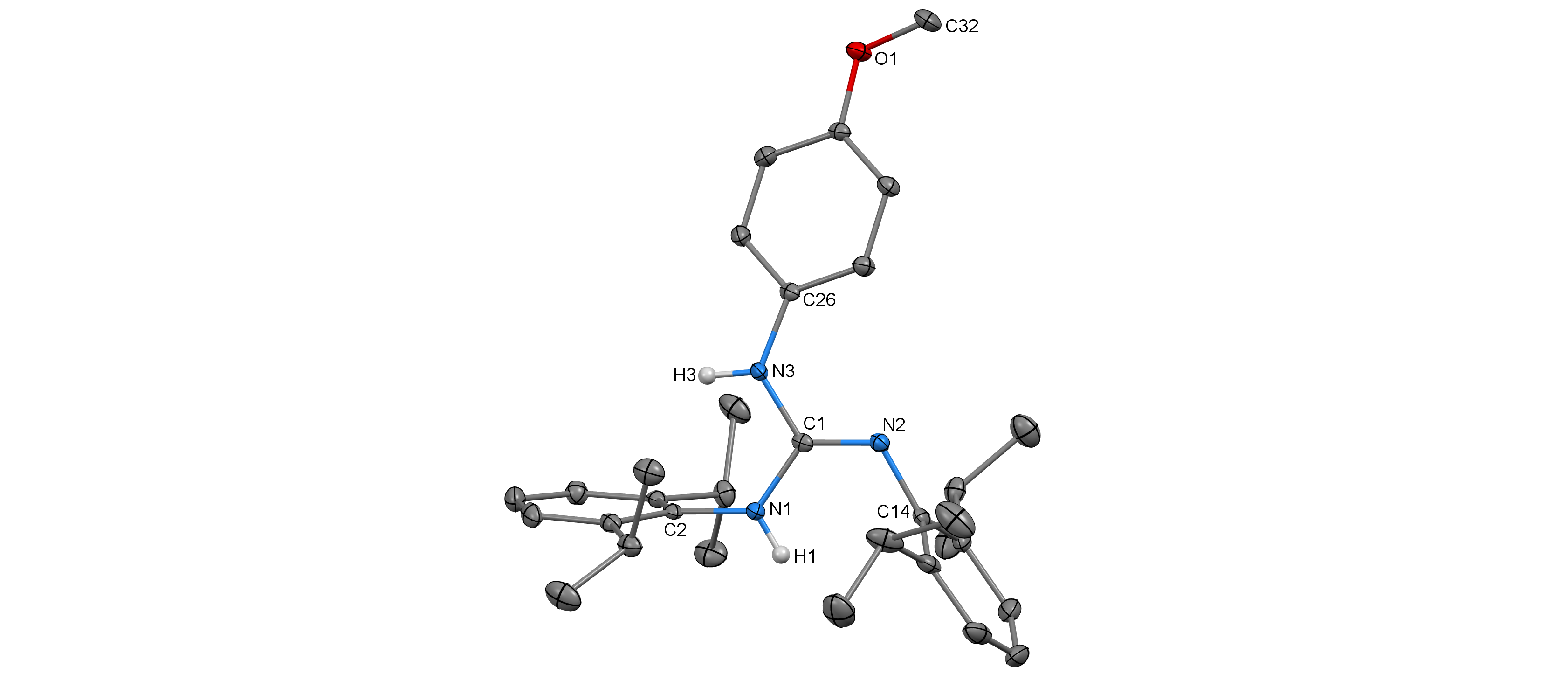


**Figure S36.** Molecular structure of **23** (ORTEP view, 30% probability level Hydrogen atoms (except of N–H) are omitted for clarity. Selected interatomic distances [Å] and angles [°]: C1–N1 1.381(2), C1–N2 1.286(2), C1–N3 1.377(2), C2–N1 1.440(2), C14–N2 1.418(2), C26–N3 1.419(2), N1–C1–N2 124.64(16), N1–C1–N3 113.85(16), N2–C1–N3 121.48(16).


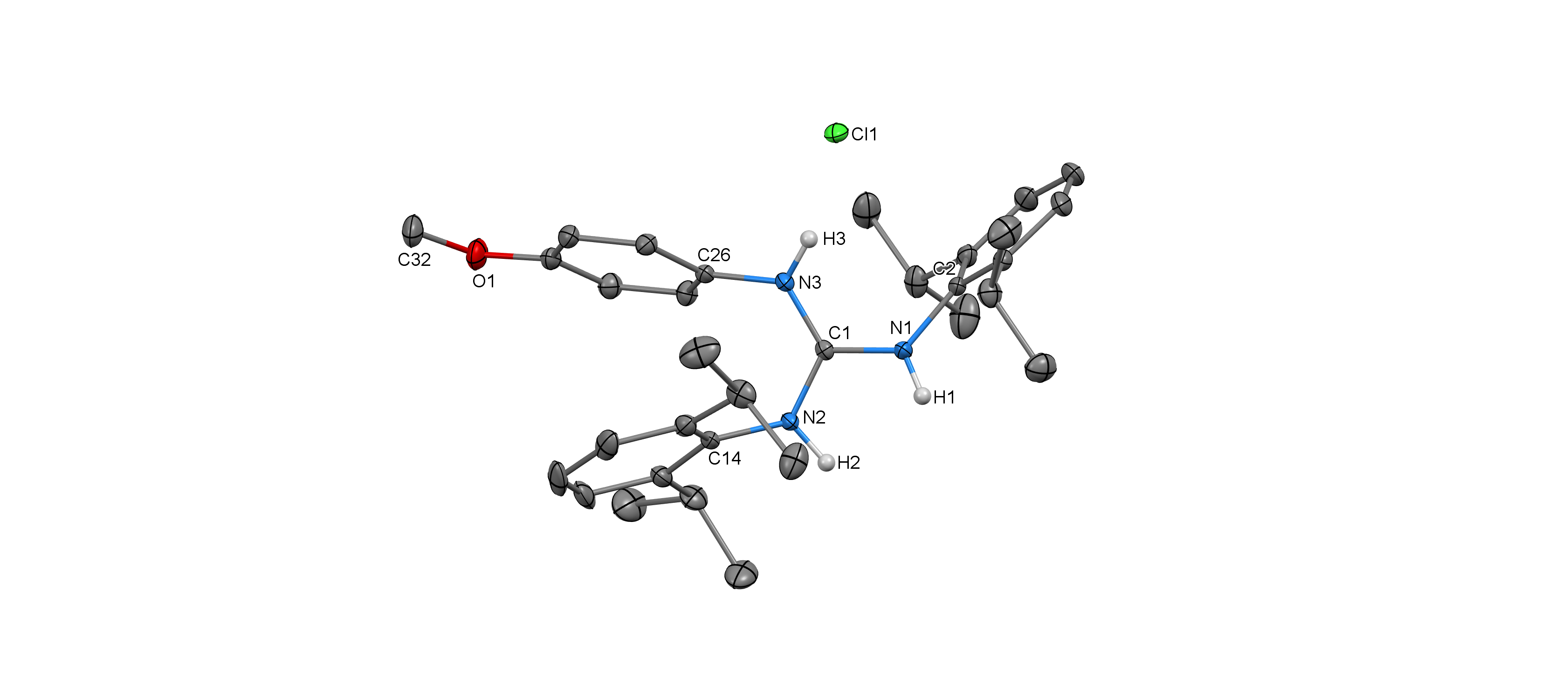


**Figure S37.** Molecular structure of **23**^.^HCl (ORTEP view, 30% probability level). Hydrogen atoms (except of N–H) are omitted for clarity. Selected interatomic distances [Å] and angles [°]: C1–N1 1.335(3), C1–N2 1.342(3), C1–N3 1.330(3), C2–N1 1.434(3), C14–N2 1.438(3), C26–N3 1.437(3), N3–Cl1 3.0938(19), N1–C1–N2 115.84(19), N1–C1–N3 120.68(19), N2–C1–N3 123.47(19).


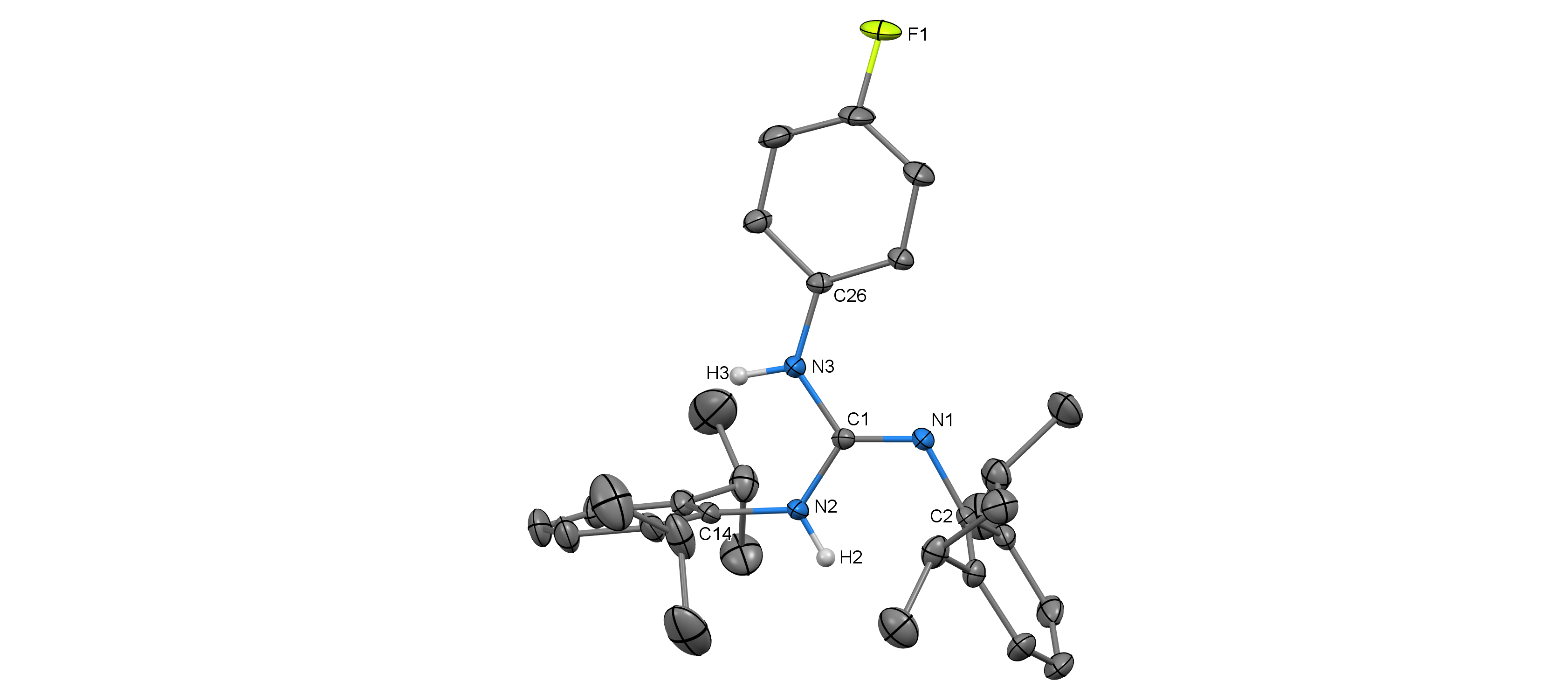


**Figure S38.** Molecular structure of **24** (ORTEP view, 30% probability level). Hydrogen atoms (except of N–H) are omitted for clarity. Selected interatomic distances [Å] and angles [°]: C1–N1 1.278(3), C1–N2 1.376(3), C1–N3 1.383(3), C2–N1 1.426(3), C14–N2 1.434(3), C26–N3 1.396(3), N1–F1 2.874(6), N1–C1–N2 124.06(19), N1–C1–N3 122.7(2), N2–C1–N3 113.19(18).


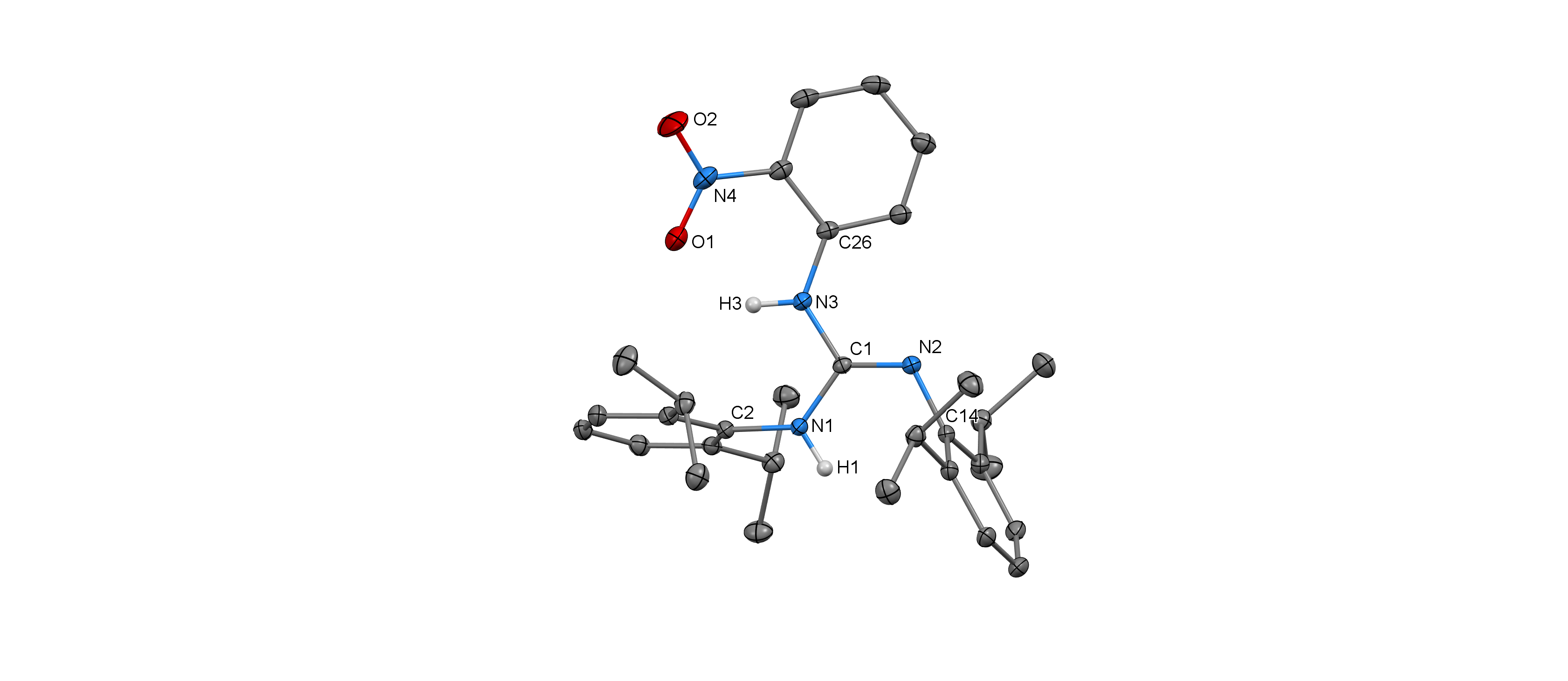


**Figure S39.** Molecular structure of **25** (ORTEP view, 30% probability level). Hydrogen atoms (except of N–H) are omitted for clarity. Selected interatomic distances [Å] and angles [°]: C1–N1 1.3806(12), C1–N2 1.2821(12), C1–N3 1.3876(11), C2–N1 1.4358(11), C14–N2 1.4230(11), C26–N3 1.3845(12), N3–O1 2.6237(11), N1–C1–N2 124.55(8), N1–C1–N3 112.87(8), N2–C1–N3 122.53(8).


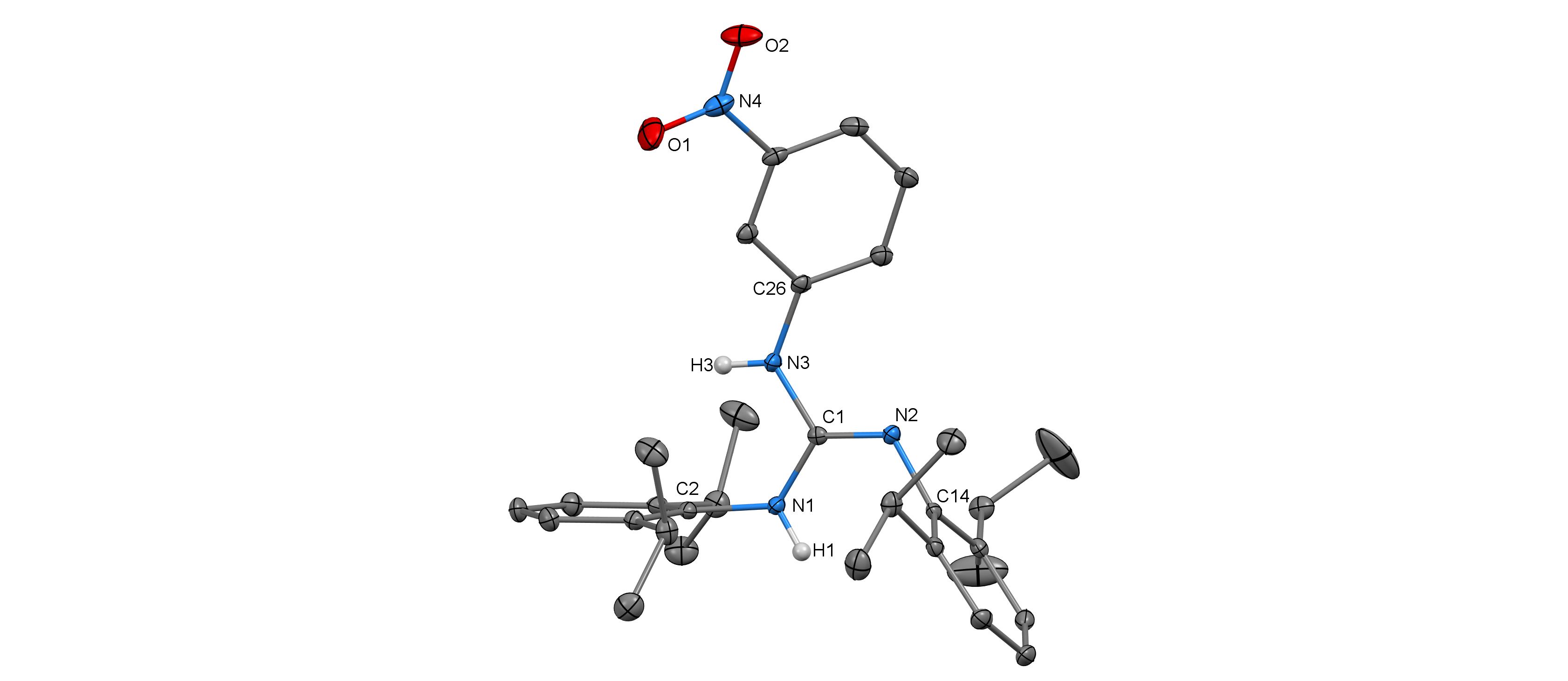


**Figure S40.** Molecular structure of **26** (ORTEP view, 30% probability level). Hydrogen atoms (except of N–H) are omitted for clarity. Selected interatomic distances [Å] and angles [°]: C1–N1 1.380(2), C1–N2 1.278(2), C1–N3 1.383(2), C2–N1 1.437(2), C14–N2 1.420(2), C26–N3 1.406(2), N1–C1–N2 125.26(15), N1–C1–N3 114.48(14), N2–C1–N3 120.24(15).


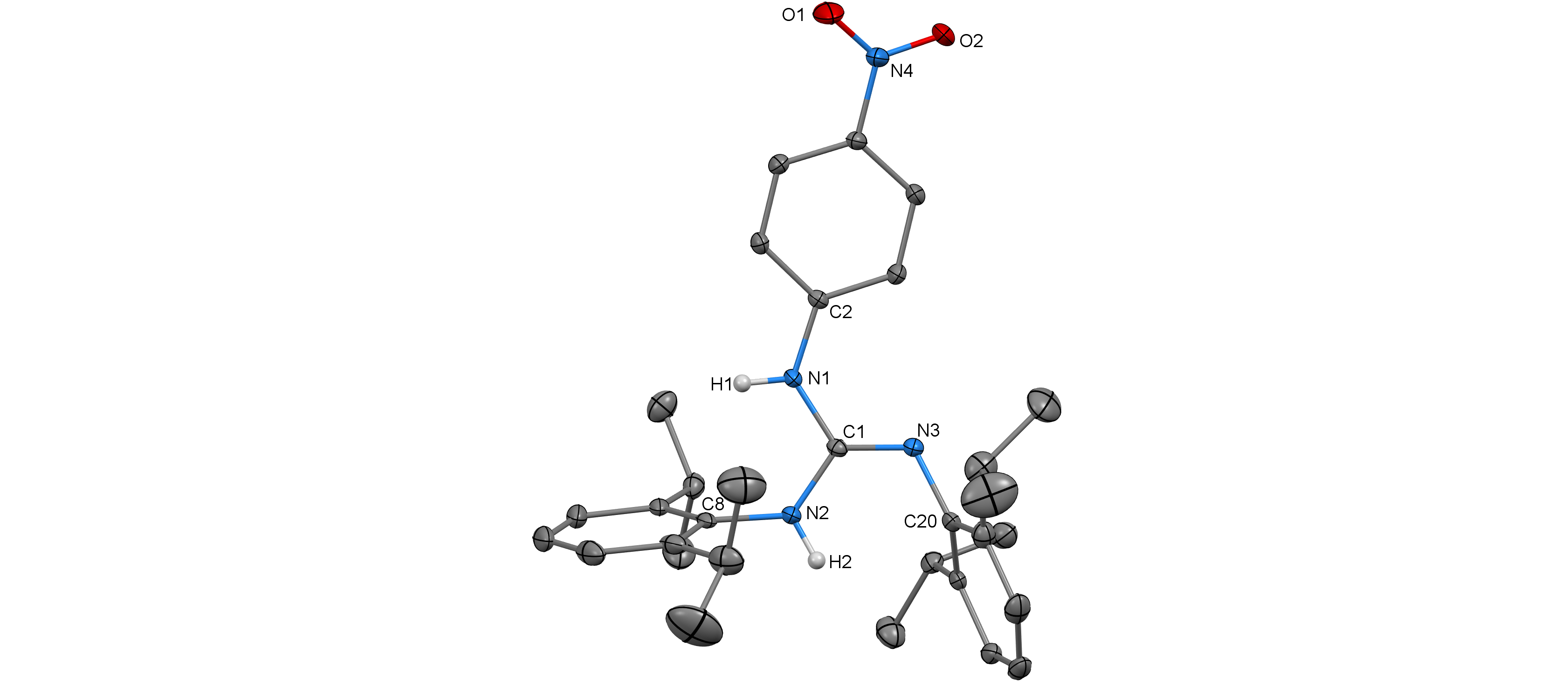


**Figure S41.** Molecular structure of **27** (ORTEP view, 30% probability level). Hydrogen atoms (except of N–H) are omitted for clarity. Selected interatomic distances [Å] and angles [°]: C1–N1 1.385(2), C1–N2 1.374(2), C1–N3 1.282(2), C2–N1 1.398(2), C8–N2 1.436(2), C20–N3 1.426(2), N1–C1–N2 113.30(16), N1–C1–N3 122.12(17), N2–C1–N3 124.57(17).


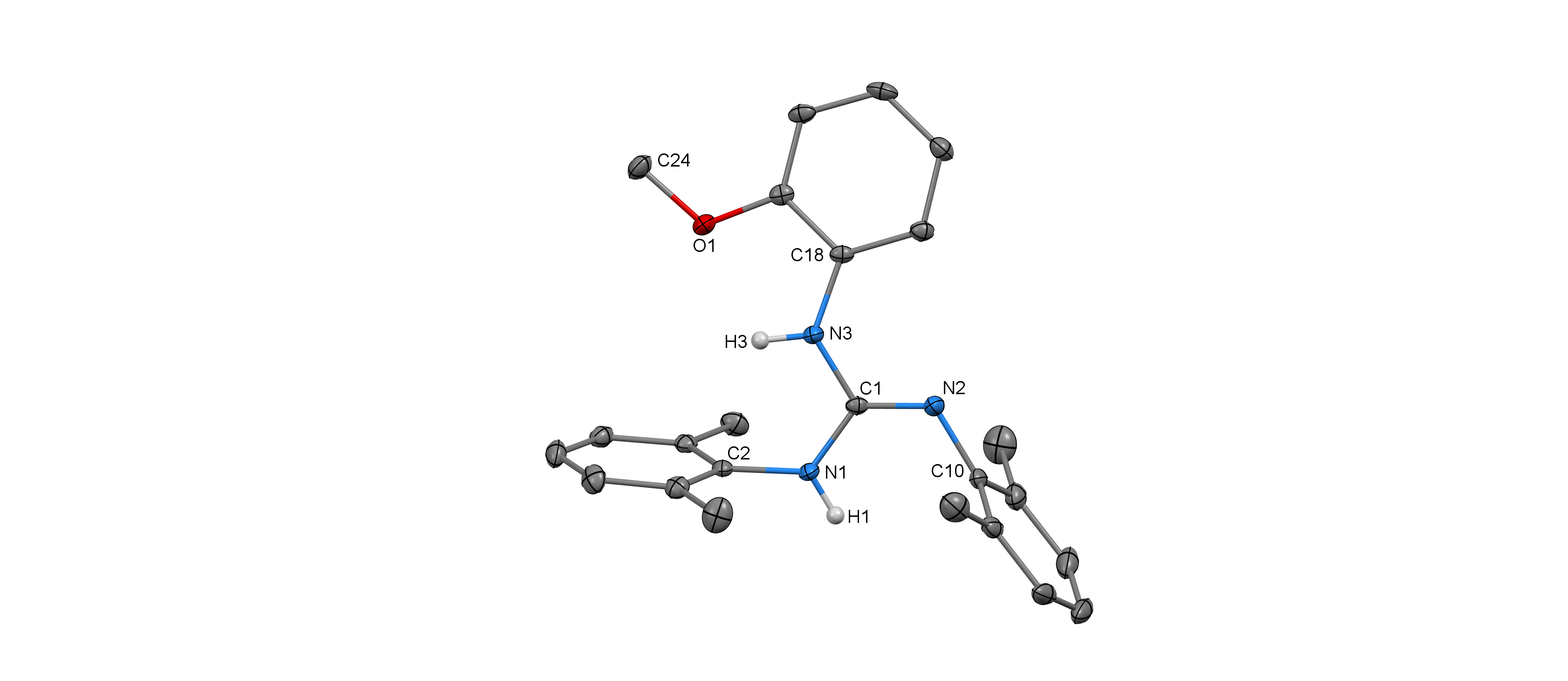


**Figure S42.** Molecular structure of **28** (ORTEP view, 30% probability level). Hydrogen atoms (except of N–H) are omitted for clarity. Selected interatomic distances [Å] and angles [°]: C1–N1 1.370(3), C1–N2 1.280(3), C1–N3 1.382(3), C2–N1 1.432(3), C10–N2 1.413(3), C18–N3 1.407(3), N1–C1–N2 125.9(2), N1–C1–N3 113.1(2), N2–C1–N3 121.0(2).


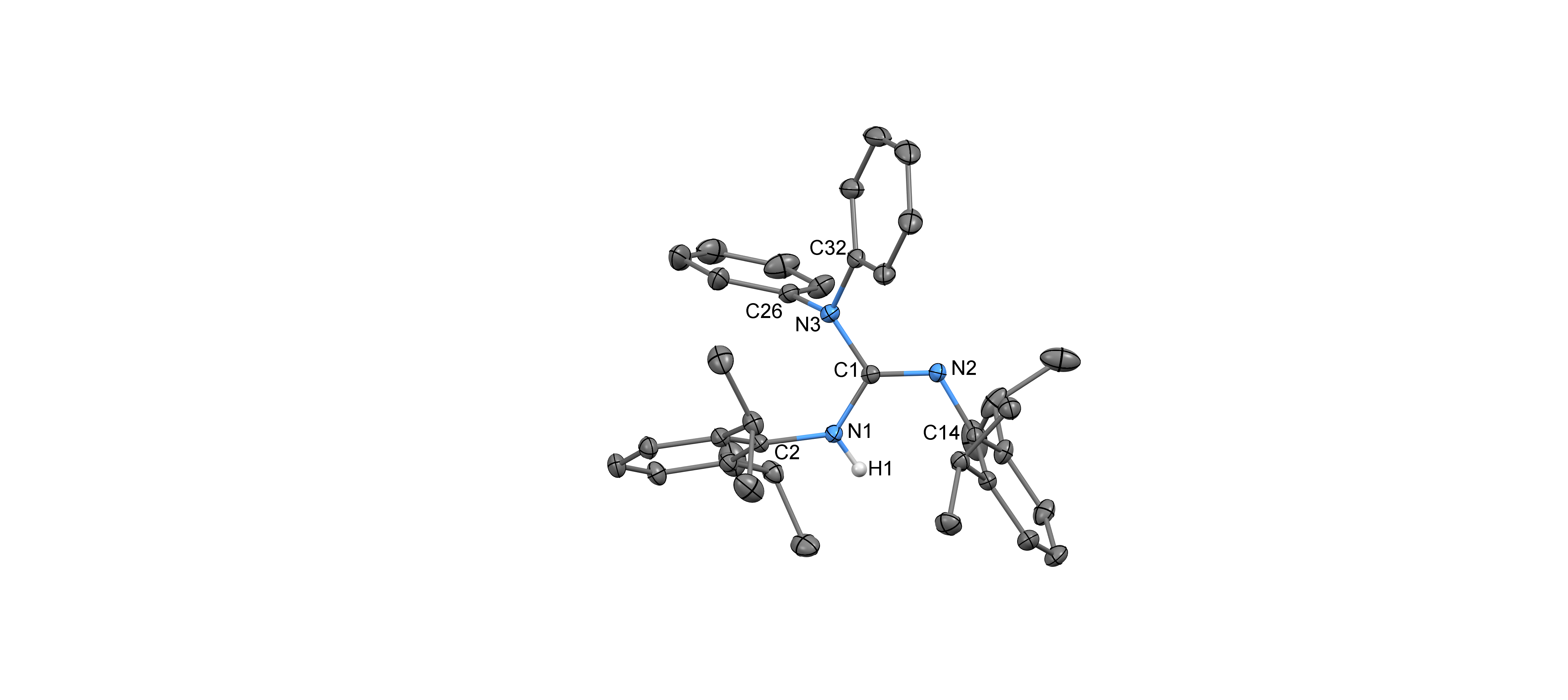


**Figure S43.** Molecular structure of **29** (ORTEP view, 40% probability level). Hydrogen atoms (except of N–H) are omitted for clarity. Selected interatomic distances [Å] and angles [°]: N1—C1 1.3679(13), N1—C2 1.4295(13), N2—C1 1.2859(13), N2—C14 1.4251(13), N3—C1 1.4132(12), N3—C32 1.4316(14), N3—C26 1.4398(14), C1—N1—C2 128.36(9), C1—N2—C14 118.13(9), C1—N3—C32 119.02(9), C1—N3—C26 115.09(8), C32—N3—C26 117.10(9), N2—C1—N1 123.69(9), N2—C1—N3 120.97(9), N1—C1—N3 115.33(9).

**General methods**

All solvents and chemical reagents were purchased from commercial sources, and used without further purification. Some synthetic procedures were performed using the standard Schlenk techniques under an inert argon atmosphere (99.999%) (inert gas was passed through the oxygen/moisture trap Supelco before entering the vacuum/inert line) and solvents were dried with the help of solvent purification system PureSolv MD 7 supplied by Innovative Technology, Inc., degassed and then stored under argon atmosphere over a potassium or sodium mirror, if needed. Single crystals suitable for X-ray analysis were obtained from corresponding saturated solutions of products in organic solvent(s) cooled to 7 or -30 °C or by slow evaporation at room temperature. Deuterated solvents for NMR spectroscopy, if needed, were distilled, degassed, and stored over a K or Na-mirror under an argon atmosphere.

Elemental analysis (C, H, N, Cl) were performed on an automatic microanalyser Flash 2000 Organic elemental analyzer. Mass spectrometry with high resolution was determined by the “dried droplet” method using a MALDI mass spectrometer LTQ Orbitrap XL (Thermo Fisher Scientific) equipped with a nitrogen UV laser (337 nm, 60 Hz). Spectra were measured in positive ion mode and in regular mass extent with a resolution of 100,000 at a mass-to-charge ratio (m/z) of 400, with 2,5-dihydrobenzoic acid (DHB) used as the matrix. Mass spectrum of **29** was performed on Vanquish HPLC/ISQ system from Thermo Scientific equipped with quaternary pump (VC-P20-A), split sampler (VC-A12-A), column compartment (VC-C10-A), diode array detector (VC-D11-A) and mass spectrometer (ISQ ICMS Family).

NMR spectra were recorded from solutions of appropriate compounds in deuterated solvent(s) on a Bruker Avance 500 spectrometer (equipped with a Z-gradient 5 mm Prodigy™ cryoprobe) at frequencies for ^1^H (500.13 MHz), ^13^C{^1^H} (125.76 MHz) and ^15^N (50.66 MHz) or a Bruker UltraShield™ 400 spectrometer at frequencies for ^1^H (400.13 MHz), ^13^C{^1^H} (100.58 MHz) at 295 K or in some cases at various temperatures. Solutions were obtained by dissolving approximately 40 mg of each compound approximately in 0.6 ml of deuterated solvents. Values of ^1^H chemical shifts were calibrated to residual signals of benzene (δ(^1^H) = 7.16), THF (δ(^1^H) = 1.73), toluene (δ(^1^H) = 2.09) or DMSO (δ(^1^H) = 2.50). Values of ^13^C chemical shifts were calibrated to signals of THF (δ(^13^C) = 67.6), benzene (δ(^13^C) = 128.4), toluene (δ(^13^C) = 20.4) or DMSO (δ(^13^C) = 20.4) and ^15^N to external nitromethane (δ(^15^N) = 0.0). All ^13^C NMR spectra were measured using a standard proton-decoupled experiment and CH and CH_3_ vs. C and CH_2_ were differentiated with the help of the APT method^[[7]](#footnote-7)^. Determination of signals of chemically nonequivalent protons, carbon and nitrogen atoms in the NMR spectra were supported by ^1^H,^1^H-COSY, ^1^H,^1^H-NOESY, ^1^H,^13^C-HSQC, ^1^H,^13^C-HMBC or/and ^1^H,^15^C-HMBC techniques.

**General methods for guanylation reactions (1 – 29)**

**Method A: Guanylation without HCl (1 – 17)**

To a round-bottom flask equipped with condenser, one equivalent of colorless *N*,*N*′-diisopropylcarbodiimide, 1,3-di-*p*-tolylcarbodiimide or *N*,*N*'-bis(2,6-diisopropylphenyl)carbodiimide with one (or half) equivalent of appropriate aliphatic (except **13**) amine/diamine was dissolved in toluene. The reaction mixture was refluxed for several days, depending on the type of amine (2 – 19 days). After that, the toluene was evaporated under vacuum, and crude products were purified by recrystallization from organic solvent(s) or by distillation (for **1**).

**Method B: Guanylation with stoichiometric amount of HCl (18**–**29**, **19**^.^HCl, **21**^.^HCl and **23**^.^HCl**)**

To a round-bottom flask equipped with condenser, one equivalent of colorless *N,N′*-dicyclohexylcarbodiimide, *N*,*N*'-bis(2,6-dimethylphenyl)carbodiimide or *N*,*N*'-bis(2,6-diisopropylphenyl)carbodiimide with one equivalent of appropriate aromatic amine was dissolved in toluene followed by addition of same equivalent of HCl solution. The reaction mixture became heterogeneous and was heated to 100 °C overnight with subsequent cooling to room temperature. Appropriate crude guanidinium chlorides were formed.

In the case of **19**^.^HCl, **21**^.^HCl and **23**^.^HCl crude products were additionally isolated by filtration and purified by washing/recrystallization from organic solvent. However, for **25**, all solvents from the reaction mixture were evaporated, crude product (mainly guanidinium) washed by a big portion of Et_2_O and suspended in hexane.

Then 1.1 equivalent of Et_3_N was added into the mixture (all protonated forms were transformed to their neutral species) which led to gradual precipitation of white solid of Et_3_N^.^HCl for 30 min at 80 °C (except **19**^.^HCl – *^t^*BuOK in THF). After that, prepared guanidines **19** – **28** were filtered off, toluene evaporated under vacuum, and crude products were purified by recrystallization from organic solvent(s). Detail complex purification process of **18** is described below.

**Method C: Guanylation with sub-stoichiometric amount of HCl (20** – **24, 26** - **27)**

To a round-bottom flask equipped with condenser, colorless *N*,*N*'-bis(2,6-diisopropylphenyl)carbodiimide (0.5 g, 1.38 mmol) with one equivalent of appropriate aromatic amine was dissolved in toluene (30 ml) followed by addition of sub-stoichiometric amount of HCl solution (35% aqueous solution, ρ = 1.180 g.cm^-3^), which caused immediate formation of white precipitate (related to amount of HCl). The reaction mixture was heated to 100 °C for a certain period, which depends on degree of conversion monitored by integration of ^1^H NMR spectra in THF-d_8_ of reaction mixture aliquots in timeline.

In some cases, Et_3_N (10% excess relative to HCl) was added into the mixture, which led to gradual precipitation of white solid of Et_3_N^.^HCl for 30 min at 80 °C. All protonated forms were transformed to their neutral species, to confirm the amount of formed guanidines, determined by NMR yield without further isolation/purification.

**Preparation of 1**

**Method A:** *N*,*N*′-diisopropylcarbodiimide (28.35 ml, ρ = 0.815 g.cm^-3^ 0.18 mol); benzylamine (20 ml, ρ = 0.981 g.cm^-3^, 0.18 mol); 5 days. Purified by distillation (fraction at 175 - 185 °C, 0.5 mbar) to give 26.882 g (64%) of white solid **1**. Single crystals suitable for sc-XRD analysis were obtained by slow evaporation of hexane from saturated solution of **1** at room temperature. Mp <50 °C.

^1^H NMR (C_6_D_6_, 400 MHz, 295 K) δ: 7.52 (br s, 2H, Ar*H*); 7.24 (s, 2H, Ar*H*); 7.11 (m, 1H, Ar*H*); 4.38 (br s, 2H, C*H*_2_); 3.99 (br s, 2H, N*H*); 3.11 (br s, 2H, C*H*); 1.07 (br s, 12H, C*H*_3_). ^13^C NMR (C_6_D_6_, 125 MHz, 295 K) δ: 150.9 (br s, Ar_q_^Gua^); 143.6 (br s, Ar); 128.9 (br s, Ar); 128.4 (br s, Ar); 126.9 (br s, Ar); 49.8 (br s, *C*H_2_); 44.3 (br s, *C*H); 24.3 (br s, *C*H_3_). ^1^H NMR (THF-d_8_, 500 MHz, 295 K) δ: 7.38 (d, 2H, ^3^*J* = 6.8 Hz, Ar*H*); 7.22 (t, 2H, ^3^*J* = 7.1 Hz, Ar*H*); 7.11 (s, 1H, Ar*H*); 4.24 (br s, 2H, C*H*_2_); 3.88 (br s, 2H, N*H*); 3.68 (br s, 2H, C*H*); 1.11 (br s, 12H, C*H*_3_). ^13^C NMR (THF-d_8_, 125 MHz, 295 K) δ: 151.4 (br s, Ar_q_^Gua^); 144.7 (br s, Ar); 128.7 (br s, Ar); 128.2 (br s, Ar); 126.5 (br s, Ar); 49.7 (br s, *C*H_2_); 44.5 (br s, *C*H); 24.3 (br s, *C*H_3_).

MALDI: [M+H]^+^ Calcd. for C_14_H_23_N_3_ 234.19702; found 234.19586.

**Preparation of 2**

**Method A:** *N*,*N*'-bis(2,6-diisopropylphenyl)carbodiimide (5.000 g, 13.79 mmol); diethylamine (1.43 ml, ρ = 0.707 g.cm^-3^, 13.79 mmol); 2 days; recrystallized from petroleum ether (*ca* 35 ml). Yield of 4.746 g (79%) of white crystalline **2**. Single crystals suitable for sc-XRD analysis were obtained by cooling of saturated solution of **2** in petroleum ether to -30 °C. Mp 103 - 103.5 °C.

^1^H NMR (C_6_D_6_, 500 MHz, 295 K) δ: 7.28 (d, ^3^*J* = 7.7 Hz, 2H, Ar*H*); 7.14 (t, ^3^*J* = 7.7 Hz, 1H, Ar*H*); 7.08 (t, ^3^*J* = 7.6 Hz, 1H, Ar*H*); 7.01 (d, ^3^*J*_H,H_ = 7.6 Hz, 2H, Ar*H*); 5.21 (s, 1H, N*H*); 3.46 (m, ^3^*J* = 6.8 Hz, 2H, C*H*); 3.38 (m, ^3^*J* = 6.8 Hz, 2H, C*H*); 3.01 (q, ^3^*J* = 7.1 Hz, 4H, C*H*_2_^Et^); 1.40 (m, 12H, C*H*_3_^Dipp^); 1.26 (br s, 6H, C*H*_3_^Dipp^); 0.96 (t, ^3^*J* = 7,0 Hz, 6H, C*H*_3_^Et^); 0.91 (br s, 6H, C*H*_3_^Dipp^). ^13^C NMR (C_6_D_6_, 125 MHz, 295 K) δ: 150.7 (Ar_q_^Gua^); 145.8 (Ar); 145.7 (Ar); 140.3 (Ar); 135.5 (Ar); 127.5 (Ar); 124.4 (Ar); 123.8 (Ar); 123.5 (Ar); 43.4 (*C*H_2_^Et^); 29.5 (*C*H); 28.9 (*C*H); 25.7 (*C*H_3_^Dipp^); 25.0 (*C*H_3_^Dipp^); 23.4 (*C*H_3_^Dipp^); 22.6 (*C*H_3_^Dipp^); 13.1 (*C*H_3_^Et^). ^1^H NMR (THF-d_8_, 400 MHz, 295 K) δ: 7.18-7.04 (br m, 5H, Ar*H*); 6.88 (t, ^3^*J* = 7.6 Hz, 1H, Ar*H*); 5.29 (s, 1H, N*H*); 3.32 (m, ^3^*J* = 6.8 Hz, 2H, C*H*); 3.26 (m, ^3^*J* = 6.9 Hz, 2H, C*H*); 3.05 (q, ^3^*J* = 7.1 Hz, 4H, C*H*_2_^Et^); 1.32 (d, ^3^*J* = 7.1 Hz, 6H, C*H*_3_^Dipp^); 1.27 (br s, 6H, C*H*_3_^Dipp^); 1.15 (d, ^3^*J* = 6.7 Hz, 6H, C*H*_3_^Dipp^); 0.99 (br s, 6H, C*H*_3_^Dipp^); 0.95 (t, ^3^*J* = 7,0 Hz, 6H, C*H*_3_^Et^). ^13^C NMR (THF-d_8_, 100 MHz, 295 K) δ: 151.3 (Ar_q_^Gua^); 146.6 (Ar); 146.0 (Ar); 140.4 (Ar); 136.3 (Ar); 127.8 (Ar); 124.6 (Ar); 123.6 (Ar); 123.1 (Ar); 43.6 (*C*H_2_^Et^); 29.6 (*C*H); 29.3 (*C*H); 25.7 (*C*H_3_^Dipp^); 24.8 (*C*H_3_^Dipp^); 23.4 (*C*H_3_^Dipp^); 22.5 (*C*H_3_^Dipp^); 13.0 (*C*H_3_^Et^).

MALDI: [M+H]^+^ Calcd. for C_29_H_45_N_3_ 436.36917; found 436.36858.

**Preparation of 3**

**Method A:** 1,3-di-p-tolylcarbodiimide (1.000 g, 4.50 mmol); diethylamine (0.47 ml, ρ = 0.707 g.cm^-3^, 4.50 mmol); 3 days; recrystallized from hexane (*ca* 15 ml). Yield of 1.090 g (82%) of white solid of **3**. Mp 79.5 - 81 °C.

^1^H NMR (C_6_D_6_, 500 MHz, 295 K) δ: 7.04 (s, 2H, Ar*H*); 6.91 (s, 4H, Ar*H*); 6.66 (s, 2H, Ar*H*); 5.27 (s, 1H, N*H*); 3.23 (q, ^3^*J* = 7.1 Hz, 4H, C*H*_2_^Et^); 2.09 (s, 6H, C*H*_3_); 1.02 (t, ^3^*J* = 7.0 Hz, 6H, C*H*_3_^Et^). ^13^C NMR (C_6_D_6_, 125 MHz, 295 K) δ: 150.4 (Ar_q_^Gua^); 148.8 (Ar); 141.8 (Ar); 131.4 (Ar); 130.5 (Ar); 123.1 (Ar); 118.7 (Ar); 42.6 (*C*H_2_^Et^); 21.1 (*C*H_3_); 13.4 (*C*H_3_^Et^). ^1^H NMR (THF-d_8_, 500 MHz, 295 K) δ: 6.92 (s, 4H, Ar*H*); 6.78 (s, 2H, Ar*H*); 6.67 (s, 2H, Ar*H*); 6.33 (s, 1H, N*H*); 3.31 (q, 4H, ^3^*J* = 7.0 Hz, C*H*_2_^Et^); 2.20 (s, 6H, C*H*_3_); 1.11 (t, 6H, ^3^*J* = 7.0 Hz, C*H*_3_^Et^).

MALDI: [M+H]^+^ Calcd. for C_19_H_25_N_3_ 296.21267; found 296.21241.

**Preparation of 4**

**Method A:** *N*,*N*'-bis(2,6-diisopropylphenyl)carbodiimide (1.000 g, 2.76 mmol); benzylamine (0.30 ml, ρ = 0.981 g.cm^-3^, 2.76 mmol); 3 days; recrystallized from petroleum ether (*ca* 15 ml). Yield of 1.190 g (92%) of white crystalline **4**. Single crystals suitable for sc-XRD analysis were obtained by slow evaporation of Et_2_O from saturated solution of **4** at room temperature. Mp 85 - 85.5 °C.

^1^H NMR (C_6_D_6_, 500 MHz, 295 K) δ: 7.29 (d, ^3^*J* = 7.4 Hz, 1H, Ar*H*^Dipp^); 7.21 (d, ^3^*J* = 7.4 Hz, 1H, Ar*H*^Bn^); 7.18 (t, ^3^*J* = 7.7 Hz, 1H, Ar*H*^Dipp^); 7.12 (d, ^3^*J* = 7.7 Hz, 1H, Ar*H*^Bn^); 7,05 (m, 2H, Ar*H*^Dipp^+Ar*H*^Bn^); 6.96 (d, ^3^*J* = 7.7 Hz, 1H, Ar*H*^Dipp^); 4.84 (s, 1H, N*H*^Dipp^); 4.36 (d, ^3^*J* = 5.9 Hz, 2H, C*H*_2_); 3.73 (t, ^3^*J* = 5.6 Hz, 1H, N*H*^Bn^); 3.48 (m, ^3^*J* = 6.8 Hz, 2H, C*H*); 3.28 (m, ^3^*J* = 6.8 Hz, 2H, C*H*); 1.41 (d, ^3^*J* = 7.0 Hz, 6H, C*H*_3_); 1.33 (d, ^3^*J* = 6.7 Hz, 6H, C*H*_3_); 1.16 (d, ^3^*J* = 6.8 Hz, 6H, C*H*_3_); 0.97 (d, ^3^*J* = 6.7 Hz, 6H, C*H*_3_). ^13^C NMR (C_6_D_6_, 125 MHz, 295 K) δ: 148.6 (Ar_q_^Dipp^); 146.5 (Ar_q_^Gua^); 145.2 (Ar_q_^Dipp^); 141.5 (Ar_q_^Dipp^+Ar_q_^Bn^); 132.6 (Ar_q_^Dipp^); 129.3 (Ar_q_^Dipp^); 128.7 (ArH^Bn^); 128.3 (ArH^Bn^); 127.3 (ArH^Bn^); 124.5 (ArH^Dipp^); 123.8 (ArH^Dipp^); 123.5 (ArH^Dipp^); 45.4 (*C*H_2_); 29.2 (*C*H); 28.7 (*C*H); 25.2 (*C*H_3_); 24.6 (*C*H_3_); 24.2 (*C*H_3_); 23.1 (*C*H_3_).

MALDI: [M+H]^+^ Calcd. for C_32_H_43_N_3_ 470.35352; found 470.35353.

**Preparation of 5**

**Method A:** *N*,*N*'-bis(2,6-diisopropylphenyl)carbodiimide (1.000 g, 2.76 mmol); 1-phenylethylamine (0.36 ml, ρ = 0.940 g.cm^-3^, 2.76 mmol); 3 days; recrystallized from petroleum ether (*ca* 15 ml). Yield of 1.134 g (85%) of white crystalline **5**. Single crystals suitable for sc-XRD analysis were obtained by slow evaporation of Et_2_O from saturated solution of **5** at room temperature. Mp 104.5 - 105 °C.

^1^H NMR (C_6_D_6_, 500 MHz, 295 K) δ: 7.29 (d, ^3^*J* = 7.6 Hz, 1H, Ar*H*^Dipp^); 7.26 (d, ^3^*J* = 7.4 Hz, 1H, Ar*H*^Dipp^); 7.20 (d, ^3^*J* = 7.4 Hz, 2H, Ar*H*^Ph^); 7.17 (t, ^3^*J* = 7.8 Hz, 1H, Ar*H*^Dipp^); 7.11 (t, ^3^*J* = 7.4 Hz, 2H, Ar*H*^Ph^); 7.08 (t, ^3^*J* = 7.8 Hz, 1H, Ar*H*^Dipp^); 7.05-6.98 (br m, 2H, Ar*H*^Dipp^+Ar*H*^Ph^); 6.96 (d, ^3^*J* = 7.6 Hz, 1H, Ar*H*^Dipp^); 5.48 (m, ^3^*J* = 7.0 Hz, 1H, C*H*-NH); 4.85 (s, 1H, N*H*^Dipp^); 3.74 (d, ^3^*J* = 7.2 Hz, 1H, CH-N*H*); 3.59 (m, ^3^*J* = 6.8 Hz, 1H, C*H*^Dipp^); 3.38 (m, ^3^*J* = 6.8 Hz, 1H, C*H*^Dipp^); 3.28-3.16 (br m, 2H, 2x C*H*^Dipp^); 1.46 (d, ^3^*J* = 7.0 Hz, 3H, C*H*_3_); 1.43 (d, ^3^*J* = 6.7 Hz, 3H, C*H*_3_^Dipp^); 1.35-1.28 (m, 6H, 2x C*H*_3_^Dipp^); 1.19 (d, ^3^*J* = 7.0 Hz, 3H, CH-C*H*_3_); 1.14 (d, ^3^*J* = 6.6 Hz, 3H, C*H*_3_^Dipp^); 1.11 (d, ^3^*J* = 6.7 Hz, 3H, C*H*_3_^Dipp^); 1.03 (d, ^3^*J* = 6.7 Hz, 3H, C*H*_3_^Dipp^); 0.90 (d, ^3^*J* = 6.7 Hz, 3H, C*H*_3_^Dipp^). ^13^C NMR (C_6_D_6_, 500 MHz, 295 K) δ: 148.5 (2x Ar_q_^Dipp^); 146.2 (Ar_q_^Ph^); 145.6 (Ar_q_^Gua^); 145.2 (Ar_q_^Dipp^); 141.7 (Ar_q_^Dipp^); 141.3 (Ar_q_^Dipp^); 132.5 (Ar_q_^Dipp^); 129.3 (ArH^Dipp^); 128.8 (ArH^Ph^); 127.3 (ArH^Ph^); 126.8 (ArH^Ph^); 124.5 (ArH^Dipp^); 124.4 (ArH^Dipp^); 123.8 (ArH^Dipp^); 123.7 (ArH^Dipp^); 123.4 (ArH^Dipp^); 50.9 (*C*H-NH); 29.2 (*C*H^Dipp^); 29.1 (*C*H^Dipp^); 28.7 (2x *C*H^Dipp^); 25.1 (2x *C*H_3_^Dipp^); 24.8 (*C*H_3_^Dipp^); 24.6 (*C*H_3_^Dipp^); 24.5 (*C*H_3_^Dipp^); 23.8 (*C*H_3_^Dipp^); 23.1 (*C*H_3_^Dipp^); 23.0 (CH-*C*H_3_); 22.9 (*C*H_3_^Dipp^).

MALDI: [M+H]^+^ Calcd. for C_33_H_45_N_3_ 484.36917; found 484.36913.

**Preparation of 6**

**Method A:** *N*,*N*'-bis(2,6-diisopropylphenyl)carbodiimide (4.643 g, 12.81 mmol); 2-picolylamine (1.32 ml, ρ = 1.049 g.cm^-3^, 12.81 mmol); 3 days; recrystallized from petroleum ether (*ca* 40 ml). Yield of 4.702 g (78%) of white crystalline **6**. Single crystals suitable for sc-XRD analysis were obtained by cooling of saturated solution of **6** in hexane to 7 °C. Mp 118.5 - 120 °C.

^1^H NMR (C_6_D_6_, 500 MHz, 295 K) δ: 8.20 (d, ^3^*J* = 4.5 Hz, 1H, Ar*H*^Py^); 7.30 (d, ^3^*J* = 7.5 Hz, 2H, Ar*H*^Dipp^); 7.22-7.11 (br m, 2H, Ar*H*^Dipp^); 7.06 (d, ^3^*J* = 7.6 Hz, 2H, Ar*H*^Dipp^); 6.96 (t, ^3^*J* = 7.2 Hz, 1H, Ar*H*^Py^); 6.88 (d, ^3^*J* = 7.7 Hz, 1H, Ar*H*^Py^); 6.50 (t, ^3^*J* = 5.8 Hz, 1H, Ar*H*^Py^); 4.93 (s, 1H, N*H*^Dipp^); 4.82 (t, ^3^*J* = 4.8 Hz, 1H, N*H*^Py^); 4.63 (d, 3*J* = 4.9 Hz, 2H, C*H*_2_); 3.49 (m, ^3^*J* = 6.8 Hz, 2H, C*H*); 3.40 (m, ^3^*J* = 6.8 Hz, 2H, C*H*); 1.42 (d, ^3^*J* = 6.9 Hz, 6H, C*H*_3_); 1.31 (d, ^3^*J* = 6.7 Hz, 6H, C*H*_3_); 1.29 (d, ^3^*J* = 6.7 Hz, 6H, C*H*_3_); 1.02 (d, ^3^*J* = 6.0 Hz, 6H, C*H*_3_). ^13^C NMR (C_6_D_6_, 125 MHz, 295 K) δ: 159.5 (Ar_q_^Py^); 149.2 (ArH^Py^); 148.6 (Ar_q_^Dipp^); 147.2 (Ar_q_^Gua^); 145.4 (Ar_q_^Dipp^); 141.6 (Ar_q_^Dipp^); 136.0 (ArH^Py^); 132.7 (Ar_q_^Dipp^); 129.2 (ArH^Dipp^); 124.4 (ArH^Dipp^); 123.8 (ArH^Dipp^); 123.5 (ArH^Dipp^); 122.3 (ArH^Py^); 121.8 (ArH^Py^); 47.1 (*C*H_2_); 29.1 (*C*H); 28.8 (*C*H); 25.2 (*C*H_3_); 24.8 (*C*H_3_); 24.2 (*C*H_3_); 23.1 (*C*H_3_). ^1^H NMR (THF-d_8_, 500 MHz, 295 K) δ: 8.35 (d, ^3^*J* = 4.3 Hz, 1H, Ar*H*^Py^); 7.60 (t, ^3^*J* = 6.9 Hz, 1H, Ar*H*^Py^); 7.42 (d, ^3^*J* = 7.8 Hz, 1H, Ar*H*^Py^); 7.25 (t, ^3^*J* = 7.8 Hz, 1H, Ar*H*^Dipp^); 7.17 (d, ^3^*J* = 7.6 Hz, 2H, Ar*H*^Dipp^); 7.07 (t, ^3^*J* = 5.7 Hz, 1H, Ar*H*^Py^); 7.04 (d, ^3^*J* = 7.6 Hz, 2H, Ar*H*^Dipp^); 6.86 (t, ^3^*J* = 7.6 Hz, 1H, Ar*H*^Dipp^); 5.68 (s, 1H, N*H*^Dipp^); 4.92 (t, ^3^*J* = 5.1 Hz, 1H, N*H*^Py^); 4.61 (d, ^3^*J* = 5.4 Hz, 2H, C*H*_2_); 3.43 (m, ^3^*J* = 6.8 Hz, 2H, C*H*); 3.30 (m, ^3^*J* = 6.8 Hz, 2H, C*H*); 1.30 (d, ^3^*J* = 7.0 Hz, 6H, C*H*_3_); 1.27 (d, ^3^*J* = 6.9 Hz, 6H, C*H*_3_); 1.09 (d, ^3^*J* = 6.9 Hz, 6H, C*H*_3_); 1.06 (d, ^3^*J* = 7.1 Hz, 6H, C*H*_3_). ^13^C NMR (THF-d_8_, 125 MHz, 295 K) δ: 161.2 (Ar_q_^Py^); 149,6 (ArH^Py^); 149,2 (Ar_q_^Dipp^); 147.6 (Ar_q_^Gua^); 146.2 (Ar_q_^Dipp^); 141.6 (Ar_q_^Dipp^); 136.6 (ArH^Py^); 134.0 (Ar_q_^Dipp^); 129.0 (ArH^Dipp^); 124.5 (ArH^Dipp^); 123.3 (ArH^Dipp^); 122.6 (ArH^Dipp^); 122.5 (ArH^Py^); 122.2 (ArH^Py^); 47.5 (*C*H_2_); 29.2 (*C*H); 29.1 (*C*H); 24.9 (*C*H_3_); 24.8 (*C*H_3_); 24.3 (*C*H_3_); 23.1 (*C*H_3_).

MALDI: [M+H]^+^ Calcd. for C_31_H_42_N_4_ 471.34877; found 471.34810.

**Preparation of 7**

**Method A:** *N*,*N*'-bis(2,6-diisopropylphenyl)carbodiimide (1.000 g, 2.76 mmol); 2-(1-aminoethyl)pyridine (0.34 ml, ρ = 1.002 g.cm^-3^, 2.76 mmol); 3 days; recrystallized from petroleum ether (*ca* 15 ml). Yield of 1.110 g (83%) of white crystalline **7**. Single crystals suitable for sc-XRD analysis were obtained by cooling of saturated solution of **7** in petroleum ether to -30 °C. Mp 124.5 - 125 °C.

^1^H NMR (C_6_D_6_, 500 MHz, 295 K) δ: 8.15 (d, ^3^*J* = 4.5 Hz, 1H, Ar*H*^Py^); 7.32 (d, ^3^*J* = 7.5 Hz, 1H, Ar*H*^Dipp^); 7.29 (d, ^3^*J* = 7.5 Hz, 1H, Ar*H*^Dipp^); 7.19 (t, ^3^*J* = 7.8 Hz, 1H, Ar*H*^Dipp^); 7.16 (m, 1H, Ar*H*^Dipp^); 7.11-7.03 (m, 2H, Ar*H*^Dipp^); 6.93 (dt, ^3^*J* = 7.6 Hz, ^2^*J* = 1.6 Hz, 1H, Ar*H*^Py^); 6.83 (d, ^3^*J* = 7.8 Hz, 1H, Ar*H*^Py^); 6.47 (dd, ^3^*J* = 6.5 Hz, ^2^*J* = 1.0 Hz, 1H, Ar*H*^Py^); 5.54 (m, ^3^*J* = 6.8 Hz, 1H, C*H*^Py^); 5.18 (d, ^3^*J* = 7.4 Hz, 1H, N*H*^Py^); 4.91 (s, 1H, N*H*^Dipp^); 3.67 (m, ^3^*J* = 6.8 Hz, 1H, C*H*^Dipp^); 3.47 (m, ^3^*J* = 6.8 Hz, 1H, C*H*^Dipp^); 3.38-3.25 (br m, 2H, 2x C*H*^Dipp^); 1.50 (d, ^3^*J* = 6.7 Hz, 3H, C*H*_3_^Py^); 1.48 (m, 6H, 2x C*H*_3_^Dipp^); 1.39 (d, ^3^*J* = 6.7 Hz, 3H, C*H*_3_^Dipp^); 1.35 (d, ^3^*J* = 7,0 Hz, 3H, C*H*_3_^Dipp^); 1.21 (d, ^3^*J* = 6.7 Hz, 6H, 2x C*H*_3_^Dipp^); 1.07 (d, ^3^*J* = 6.8 Hz, 3H, C*H*_3_^Dipp^); 0.96 (d, ^3^*J* = 6.8 Hz, 3H, C*H*_3_^Dipp^). ^13^C NMR (C_6_D_6_, 125 MHz, 295 K) δ: 162.7 (Ar_q_^Py^); 148.4 (ArH^Py^); 147.8 (Ar_q_^Dipp^); 147.6 (Ar_q_^Dipp^); 145.6 (Ar_q_^Gua^); 144.6 (Ar_q_^Dipp^); 140.8 (2x Ar_q_^Dipp^); 135.4 (ArH^Py^); 131.8 (Ar_q_^Dipp^); 128.3 (ArH^Dipp^); 123.7 (ArH^Dipp^); 123.4 (ArH^Dipp^); 123.0 (2x ArH^Dipp^); 122.6 (ArH^Dipp^); 121.1 (ArH^Py^); 121.0 (ArH^Py^); 50.9 (*C*H^Py^); 28.4 (*C*H^Dipp^); 28.0 (*C*H^Dipp^); 27.9 (*C*H^Dipp^); 27.8 (*C*H^Dipp^); 24.4 (2x *C*H_3_^Dipp^); 24.0 (*C*H_3_^Dipp^); 23.9 (*C*H_3_^Dipp^); 23.7 (*C*H_3_^Dipp^); 23.2 (*C*H_3_^Dipp^); 22.6 (*C*H_3_^Py^); 22.3 (2x *C*H_3_^Dipp^).^1^H NMR (THF-d_8_, 500 MHz, 295 K) δ: 8.33 (d, ^3^*J* = 4.4 Hz, 1H, Ar*H*^Py^); 7.60 (dt, ^3^*J* = 7.2 Hz, ^2^*J* = 1.1 Hz, 1H, Ar*H*^Py^); 7.33 (d, ^3^*J* = 7.8 Hz, 1H, Ar*H*^Py^); 7.26 (t, ^3^*J* = 7.6 Hz, 1H, Ar*H*^Dipp^); 7.19 (d, *J* = 7.5 Hz, 1H, Ar*H*^Dipp^); 7.16 (d, ^3^*J* = 7.6 Hz, 1H, Ar*H*^Dipp^); 7.11 - 7.05 (m, 2H, Ar*H*^Dipp^ + Ar*H*^Py^); 7.02 (dd, ^3^*J* = 7.2 Hz, 1H, Ar*H*^Dipp^); 6.87 (t, ^3^*J* = 7.6 Hz, 1H, Ar*H*^Dipp^); 5.61 (s, 1H, N*H*^Dipp^); 5.35 (m, ^3^*J* = 6.9 Hz, 1H, C*H*^Py^); 4.88 (d, ^3^*J* = 7.3 Hz, 1H, N*H*^Py^); 3.50-3.39 (m, 2H, 2x C*H*^Dipp^); 3.33 (m, ^3^*J* = 6.8 Hz, 1H, C*H*^Dipp^); 3.10 (m, ^3^*J* = 6.9 Hz, 1H, C*H*^Dipp^); 1.44 (d, ^3^*J* = 6.8 Hz, 3H, C*H*_3_^Py^); 1.40-1.31 (m, 6H, 2x C*H*_3_^Dipp^); 1.25-1.18 (m, 6H, 2x C*H*_3_^Dipp^); 1.17-1.10 (m, 6H, 2x C*H*_3_^Dipp^); 1.03 (d, ^3^*J* = 6.9 Hz, 3H, C*H*_3_^Dipp^); 0.96 (d, ^3^*J* = 6.7 Hz, 3H, C*H*_3_^Dipp^). ^13^C NMR (THF-d_8_, 125 MHz, 295 K) δ: 164.3 (Ar_q_^Py^); 149.6 (ArH^Py^); 149.2 (2x Ar_q_^Dipp^); 146.7 (Ar_q_^Gua^); 146.2 (Ar_q_^Dipp^); 141.7 (Ar_q_^Dipp^); 141.6 (Ar_q_^Dipp^); 137.0 (ArH^Py^); 133.9 (Ar_q_^Dipp^); 129.1 (ArH^Dipp^); 124.5 (ArH^Dipp^); 124.4 (ArH^Dipp^); 123.4 (ArH^Dipp^); 123.3 (ArH^Dipp^); 122.6 (ArH^Dipp^); 122.5 (ArH^Py^); 122.4 (ArH^Py^); 51.9 (*C*H^Py^); 29.3 (*C*H^Dipp^); 29.1 (*C*H^Dipp^); 29.0 (2x *C*H^Dipp^); 25.0 (*C*H_3_^Dipp^); 24.9 (2x *C*H_3_^Dipp^); 24.6 (2x *C*H_3_^Dipp^); 24.0 (*C*H_3_^Dipp^); 23.3 (*C*H_3_^Py^); 23.1 (*C*H_3_^Dipp^); 23.0 (*C*H_3_^Dipp^).

MALDI: [M+H]^+^ Calcd. for C_32_H_44_N_4_ 485.36442; found 48536365.

**Preparation of 8**

**Method A:** *N*,*N*'-bis(2,6-diisopropylphenyl)carbodiimide (1.482 g, 4.09 mmol); 2-(2-aminoethyl)pyridine (0.49 ml, ρ = 1.021 g.cm^-3^, 4.09 mmol); 3 days; recrystallized from petroleum ether (*ca* 20 ml). Yield of 1.823 g (92%) of white crystalline **8**. Single crystals suitable for sc-XRD analysis were obtained by slow evaporation of Et_2_O from saturated solution of **8** at room temperature. Mp 124.5 - 125 °C.

^1^H NMR (C_6_D_6_, 500 MHz, 295 K) δ: 8.10 (d, ^3^*J* = 4.5 Hz, 1H, Ar*H*^Py^); 7.32 (d, ^3^*J* = 7.6 Hz, 2H, Ar*H*^Dipp^); 7.20 (d, ^3^*J* = 7.4 Hz, 1H, Ar*H*^Dipp^); 7.13 (t, ^3^*J* = 7.4 Hz, 1H, Ar*H*^Dipp^); 7.01 (d, ^3^*J* = 7.5 Hz, 2H, Ar*H*^Dipp^); 6.93 (t, ^3^*J* = 7.5 Hz, 1H, Ar*H*^Py^); 6.67 (d, ^3^*J* = 7.6 Hz, 1H, Ar*H*^Py^); 6.51 (t, ^3^*J* = 6.1 Hz, 1H, Ar*H*^Py^); 4.83 (s, 1H, N*H*^Dipp^); 4.60 (t, ^3^*J* = 6.3 Hz, 1H, N*H*^Py^); 3.82 (q, ^3^*J* = 5.7 Hz, 2H, C*H*_2-_NH); 3.59 (m, ^3^*J* = 6.6 Hz, 2H, C*H*^Dipp^); 3.29 (m, ^3^*J* = 6.6 Hz, 2H, C*H*^Dipp^); 2.90 (q, ^3^*J* = 6.1 Hz, 2H, C*H*_2_); 1.43 (d, ^3^*J* = 5.6 Hz, 12H, 2x C*H*_3_^Dipp^); 1.11 (d, ^3^*J* = 5.6 Hz, 6H, C*H*_3_^Dipp^); 0.99 (d, ^3^*J* = 5.5 Hz, 6H, C*H*_3_^Dipp^). ^13^C NMR (C_6_D_6_, 125 MHz, 295 K) δ: 161.2 (Ar_q_^Py^); 149.5 (Ar*H*^Py^); 148.6 (Ar_q_^Dipp^); 147.2 (Ar_q_^Gua^); 145.7 (Ar_q_^Dipp^); 141.6 (Ar_q_^Dipp^); 136.3 (Ar*H*^Py^); 132.9 (Ar_q_^Dipp^); 128.9 (ArH^Dipp^); 124.3 (ArH^Dipp^); 123.8 (ArH^Dipp^); 123.5 (Ar*H*^Py^); 123.3 (ArH^Dipp^); 121.2 (Ar*H*^Py^); 41.5 (*C*H_2-_NH); 37.8 (*C*H_2_); 29.1 (*C*H); 28.7 (*C*H); 25.2 (*C*H_3_); 24.6 (*C*H_3_); 24.2 (*C*H_3_); 23.1 (*C*H_3_).

MALDI: [M+H]^+^ Calcd. for C_32_H_44_N_4_ 485.36442; found 485.36388.

**Preparation of 9**

**Method A:** *N*,*N*'-bis(2,6-diisopropylphenyl)carbodiimide (3.732 g, 10.30 mmol); 2-aminomethylfuran (0.91 ml, ρ = 1.099 g.cm^-3^, 10.30 mmol); 3 days; recrystallized from petroleum ether (*ca* 40 ml). Yield of 3.833 g (81%) of white crystalline **9**. Mp 102.5 - 103.5 °C.

^1^H NMR (C_6_D_6_, 500 MHz, 295 K) δ: 7.28 (d, ^3^*J* = 7.6 Hz, 2H, Ar*H*^Dipp^); 7.17 (t, ^3^*J* = 7.6 Hz, 1H, Ar*H*^Dipp^); 7.08 (t, ^3^*J* = 7.6 Hz, 1H, Ar*H*^Dipp^); 6.98 (d, ^3^*J* = 7.7 Hz, 2H, Ar*H*^Dipp^); 6.96 (s, 1H, Ar*H*^Fu-5^); 6.06 (d, ^3^*J* = 2.7 Hz, 1H, Ar*H*^Fu-3^); 6.01 (dd, ^3^*J* = 3.1 Hz, ^3^*J* = 1.7 Hz, 1H, Ar*H*^Fu-4^); 4.81 (s, 1H, N*H*^Dipp^); 4.40 (d, ^3^*J* = 5.8 Hz, 2H, C*H*_2-_NH); 3.69 (t, ^3^*J* = 5.6 Hz, 1H, N*H*^Fu^); 3.46 (m, ^3^*J* = 6.9 Hz, 2H, C*H*^Dipp^); 3.23 (m, ^3^*J* = 6.8 Hz, 2H, C*H*^Dipp^); 1.39 (d, ^3^*J* = 7.0 Hz, 6H, C*H*_3_^Dipp^); 1.34 (d, ^3^*J* = 6.8 Hz, 6H, C*H*_3_^Dipp^); 1.16 (d, ^3^*J* = 6.6 Hz, 6H, C*H*_3_^Dipp^); 0.96 (d, ^3^*J* = 6.5 Hz, 6H, C*H*_3_^Dipp^). ^13^C NMR (C_6_D_6_, 125 MHz, 295 K) δ: 154.4 (Ar_q_^Fu-2^); 148.6 (Ar_q_^Dipp^); 146.3 (Ar_q_^Gua^); 145.0 (Ar_q_^Dipp^); 141.7 (ArH^Fu-5^); 141.6 (Ar_q_^Dipp^); 132.4 (Ar_q_^Dipp^); 129.3 (ArH^Dipp^); 124.5 (ArH^Dipp^); 123.8 (ArH^Dipp^); 123.6 (ArH^Dipp^); 110.8 (Ar*H*^Fu-4^); 107.2 (ArH^Fu-3^); 38.4 (*C*H_2-_NH); 29.0 (*C*H); 28.7 (*C*H); 25.3 (*C*H_3_); 24.6 (*C*H_3_); 24.1 (*C*H_3_); 23.1 (*C*H_3_). ^1^H NMR (THF-d_8_, 500 MHz, 295 K) δ: 7.31 (s, 1H, Ar*H*^Fu-5^); 7.22 (t, ^3^*J* = 7.8 Hz, 1H, Ar*H*^Dipp^); 7.14 (d, ^3^*J* = 7.7 Hz, 2H, Ar*H*^Dipp^); 7.04 (d, ^3^*J* = 7.4 Hz, 2H, Ar*H*^Dipp^); 6.86 (t, ^3^*J* = 7.6 Hz, 1H, Ar*H*^Dipp^); 6.26 (s, 1H, Ar*H*^Fu-3^); 6.18 (s, 1H, Ar*H*^Fu-4^); 5.67 (s, 1H, N*H*^Dipp^); 4.50 (d, ^3^*J* = 5.4 Hz, 2H, C*H*_2-_NH); 4.33 (t, ^3^*J* = 5.6 Hz, 1H, N*H*^Fu^); 3.32 (m, 4H, C*H*^Dipp^); 1.29 (d, ^3^*J* = 6.7 Hz, 6H, C*H*_3_^Dipp^); 1.19 (d, ^3^*J* = 6.7 Hz, 6H, C*H*_3_^Dipp^); 1.12 (d, ^3^*J* = 6.7 Hz, 6H, C*H*_3_^Dipp^); 1.06 (d, ^3^*J* = 6.4 Hz, 6H, C*H*_3_^Dipp^). ^13^C NMR (THF-d_8_, 125 MHz, 295 K) δ: 155.7 (Ar_q_^Fu-2^); 149.3 (Ar_q_^Dipp^); 146.9 (Ar_q_^Gua^); 146.1 (Ar_q_^Dipp^); 142.0 (ArH^Fu-5^); 141.6 (Ar_q_^Dipp^); 133.9 (Ar_q_^Dipp^); 129.1 (ArH^Dipp^); 124.5 (ArH^Dipp^); 123.4 (ArH^Dipp^); 122.6 (ArH^Dipp^); 110.9 (Ar*H*^Fu-4^); 107.0 (ArH^Fu-3^); 38.7 (*C*H_2-_NH); 29.1 (*C*H); 29.0 (*C*H); 25.1 (*C*H_3_); 24.6 (*C*H_3_); 24.2 (*C*H_3_); 23.1 (*C*H_3_).

MALDI: [M+H]^+^ Calcd. for C_30_H_41_N_3_O 460.33279; found 460.33228.

**Preparation of 10**

**Method A:** *N*,*N*'-bis(2,6-diisopropylphenyl)carbodiimide (2.393 g, 6.60 mmol); (R)-(-)-2-methoxy-1-phenylethylamine (0.99 ml, ρ = 1.011 g.cm^-3^, 6.60 mmol); 3 days; recrystallized from petroleum ether (*ca* 30 ml). Yield of 2.509 g (74%) of off-white crystalline **10**. Single crystals suitable for sc-XRD analysis were obtained by cooling of saturated solution of **10** in petroleum ether to -30 °C. Mp 97 - 98 °C.

^1^H NMR (C_6_D_6_, 500 MHz, 295 K) δ: 7.35 (d, ^3^*J* = 7.4 Hz, 2H, Ar*H*^Ph^); 7.28 (d, ^3^*J* = 7.5 Hz, 1H, Ar*H*^Dipp^); 7.24 (d, ^3^*J* = 7.4 Hz, 1H, Ar*H*^Dipp^); 7.20 – 7.12 (m, 3H, Ar*H*^Ph^ + Ar*H*^Dipp^); 7.11 (d, ^3^*J* = 7.6 Hz, 1H, Ar*H*^Dipp^); 7.09 – 7.03 (m, 2H, Ar*H*^Ph^ + Ar*H*^Dipp^); 7.01 (d, ^3^*J* = 7.4 Hz, 1H, Ar*H*^Dipp^); 5.47 (AB quartet, ^3^*J*_AB_ = 5.9 Hz, 1H, C*H*-NH); 4.91 (s, 1H, N*H*^Dipp^); 4.42 (d, ^3^*J* = 5.6 Hz, 1H, CH-N*H*); 3.58 (m, ^3^*J* = 6.9 Hz, 1H, C*H*^Dipp^); 3.44 (m, ^3^*J* = 6.8 Hz, 1H, C*H*^Dipp^); 3.34; 3.21 (ABX spin system, two dd, ^3^*J* = 6.4, 8.1 Hz, 1H, diastereotopic C*H*_2_); 3.34 (m, ^3^*J* = 6.8 Hz, 1H, C*H*^Dipp^); 3.31 (m, ^3^*J* = 7.0 Hz, 1H, C*H*^Dipp^); 2.83 (s, 3H, OC*H*_3_); 1.46 (d, ^3^*J* = 7.1 Hz, 3H, C*H*_3_^Dipp^); 1.40 (m, 6H, C*H*_3_^Dipp^); 1.31 (d, ^3^*J* = 7.0 Hz, 3H, C*H*_3_^Dipp^); 1.26 (d, ^3^*J* = 6.8 Hz, 3H, C*H*_3_^Dipp^); 1.06 (d, ^3^*J* = 6.8 Hz, 3H, C*H*_3_^Dipp^); 0.98 (d, ^3^*J* = 6.7 Hz, 3H, C*H*_3_^Dipp^); 0.92 (d, ^3^*J* = 6.8 Hz, 3H, C*H*_3_^Dipp^). ^13^C NMR (C_6_D_6_, 125 MHz, 295 K) δ: 148.6 (Ar_q_^Dipp^); 148.5 (Ar_q_^Dipp^); 146.1 (Ar_q_^Gua^); 145.2 (Ar_q_^Dipp^); 143.0 (Ar_q_^Ph^); 141.7 (Ar_q_^Dipp^); 141.1 (Ar_q_^Dipp^); 132.6 (Ar_q_^Dipp^); 129.3 (ArH^Dipp^); 128.6 (ArH^Ph^); 127.7 (ArH^Ph^); 127.5 (ArH^Ph^); 124.4 (2x ArH^Dipp^); 123.8 (ArH^Dipp^); 123.7 (ArH^Dipp^); 123.4 (ArH^Dipp^); 76.0 (*C*H_2_); 58.5 (O*C*H_3_); 55.4 (*C*HNH); 29.2 (*C*H^Dipp^); 29.1 (*C*H^Dipp^); 28.9 (*C*H^Dipp^); 28.7 (*C*H^Dipp^); 25.2 (*C*H_3_^Dipp^); 24.9 (3x *C*H_3_^Dipp^); 24.0 (*C*H_3_^Dipp^); 23.9 (*C*H_3_^Dipp^); 23.1 (*C*H_3_^Dipp^); 22.8 (*C*H_3_^Dipp^).

MALDI: [M+H]^+^ Calcd. for C_34_H_47_N_3_O 514.37919; found 514.37995.

**Preparation of 11**

**Method A:** *N*,*N*'-bis(2,6-diisopropylphenyl)carbodiimide (1.000 g, 2.76 mmol); 1-(1-naftyl)ethylamine (0.46 ml, ρ = 1.067 g.cm^-3^, 2.76 mmol); 19 days; recrystallized from the mixture of hexane:Et_2_O (1:1, *ca* 30 ml). Further purification was performed via protonated **11** obtained by addition of HCl (0.37 ml of 35% aqueous solution, ρ = 1.180 g.cm^-3^, 3.04 mmol) in hexane (30 ml). Corresponding guanidinium salt was washed with Et_2_O (50 ml) and toluene (50 ml) with subsequent transformation to **11** using Et_3_N (0.42 ml, ρ = 0.727 g.cm^-3^, 3.04 mmol) in hexane (50 ml) for 30 minutes. Yield of 1.104 g (75%) of white solid **11**. Mp 114 - 117.5 °C.

^1^H NMR (C_6_D_6_, 500 MHz, 295 K) δ: 8.69 (d, ^3^*J* = 7.8 Hz, 1H, Ar*H*^Naph^); 7.67 (d, ^3^*J* = 8.2 Hz, 1H, Ar*H*^Naph^); 7.57-7.49 (m, 2H, Ar*H*^Naph^); 7.37-7.29 (m, 3H, Ar*H*^Dipp^+Ar*H*^Naph^); 7.21 (t, ^3^*J* = 7.6 Hz, 1H, Ar*H*^Dipp^); 7.08 (m, 2H, Ar*H*^Dipp^); 6.91 (m, 2H, Ar*H*^Dipp^); 6.73 (m, 1H, Ar*H*^Naph^); 6.49 (m, ^3^*J* = 6.7 Hz, 1H, C*H*(Naph)); 4.92 (s, 1H, N*H*^Dipp^); 3.72 (d, ^3^*J* = 8.2 Hz, 1H, N*H*-CH(Naph)); 3.65 (m, ^3^*J* = 6.9 Hz, 2H, C*H*^Dipp^); 3.29 (m, ^3^*J* = 6.8 Hz, 1H, C*H*^Dipp^); 3.15 (m, ^3^*J* = 6.8 Hz, 1H, CH^Dipp^); 1.54 (d, ^3^*J* = 6.7 Hz, 3H, C*H*_3_-CH(Naph)); 1.50 (m, 6H, C*H*_3_^Dipp^); 1.43 (d, ^3^*J* = 7.1 Hz, 3H, C*H*_3_^Dipp^); 1.39 (d, ^3^*J* = 6.7 Hz, 3H, C*H*_3_^Dipp^); 1.32 (d, ^3^*J* = 6.7 Hz, 3H, C*H*_3_^Dipp^); 0.98 (d, ^3^*J* = 6.8 Hz, 3H, C*H*_3_^Dipp^); 0.82 (d, ^3^*J* = 6.8 Hz, 3H, C*H*_3_^Dipp^); 0.45 (d, ^3^*J* = 6.8 Hz, 3H, C*H*_3_^Dipp^). ^13^C NMR (C_6_D_6_, 125 MHz, 295 K) δ: 148.6 (Ar_q_^Dipp^); 147.9 (Ar_q_^Dipp^); 145.5 (Ar_q_^Gua^); 145.3 (Ar_q_^Dipp^); 141.7 (2x Ar_q_^Dipp^); 140.4 (Ar_q_^1-Naph^); 134.8 (Ar_q_^Naph^); 132.7 (Ar_q_^Naph^); 132.1 (Ar_q_^Dipp^); 129.3 (ArH^Naph^); 129.2 (ArH^Dipp^); 128.7 (ArH^Naph^); 126.6 (ArH^Naph^); 126.3 (ArH^Naph^); 125.7 (ArH^Dipp^); 125.0 (ArH^Naph^); 124.4 (ArH^Naph^); 124.2 (ArH^Dipp^); 124.0 (ArH^Naph^); 123.7 (ArH^Dipp^); 123.6 (ArH^Dipp^); 122.8 (ArH^Dipp^); 45.8 (*C*H(Naph)); 29.5 (*C*H^Dipp^); 29.2 (*C*H^Dipp^); 28.7 (*C*H^Dipp^); 28.6 (*C*H^Dipp^); 25.2 (*C*H_3_^Dipp^); 25.1 (*C*H_3_^Dipp^); 25.0 (*C*H_3_^Dipp^); 24.9 (*C*H_3_^Dipp^); 24.0 (*C*H_3_^Dipp^); 23.3 (*C*H_3_^Dipp^); 22.8 (*C*H_3_^Dipp^); 22.7 (*C*H_3_^Dipp^); 20.8 (*C*H_3_-CH(Naph)).

MALDI: [M+H]^+^ Calcd. for C_37_H_47_N_3_ 534.38482; found 534.38625.

**Preparation of 12**

**Method A:** *N*,*N*'-bis(2,6-diisopropylphenyl)carbodiimide (1.640 g, 4.52 mmol); 3-dimethylamino-1-propylamine (0.57 ml, ρ = 0.812 g.cm^-3^, 4.52 mmol); 3 days; recrystallized from petroleum ether (*ca* 20 ml). Yield of 1.871 g (89%) of off-white crystalline **12**. Single crystals suitable for sc-XRD analysis were obtained by cooling a saturated solution of **12** in Et_2_O to -30 °C. Mp 108.5 - 109 °C.

^1^H NMR (C_6_D_6_, 500 MHz, 295 K) δ: 7.31 (d, ^3^*J* = 6.9 Hz, 2H, Ar*H*^Dipp^); 7.18 (t, ^3^*J* = 7.1 Hz, 1H, Ar*H*^Dipp^); 7.06 (t, ^3^*J* = 7.1 Hz, 1H, Ar*H*^Dipp^); 6.96 (d, ^3^*J* = 6.9 Hz, 2H, Ar*H*^Dipp^); 4.84 (t, ^3^*J* = 4.8 Hz, 1H, N*H-*CH_2_^γ^); 4.81 (s, 1H, N*H*^Dipp^); 3.59 (m, ^3^*J* = 6.2 Hz, 2H, C*H*^Dipp^); 3.49 (AB_q_, ^2^*J* = 6.9 Hz, 2H, C*H*_2_^γ^); 3.33 (m, ^3^*J* = 6.0 Hz, 2H, C*H*^Dipp^); 1.96 (t, ^3^*J* = 5.6 Hz, 2H, C*H*_2_^α^); 1.72 (s, 6H, N(C*H*_3_)_2_); 1.45 (d, ^3^*J* = 6.7 Hz, 6H, C*H*_3_^Dipp^); 1.42 (d, ^3^*J* = 6.4 Hz, 6H, C*H*_3_^Dipp^ + br s, 2H, C*H*_2_^β^); 1.26 (d, ^3^*J* = 6.0 Hz, 6H, C*H*_3_^Dipp^); 1.01 (d, ^3^*J* = 5.6 Hz, 6H, C*H*_3_^Dipp^). ^13^C NMR (C_6_D_6_, 125 MHz, 295 K) δ: 148.6 (Ar_q_^Dipp^); 147.6 (Ar_q_^Gua^); 146.0 (Ar_q_^Dipp^); 141.7 (Ar_q_^Dipp^); 133.4 (Ar_q_^Dipp^); 128.7 (ArH^Dipp^); 124.2 (ArH^Dipp^); 123.8 (ArH^Dipp^); 123.2 (ArH^Dipp^); 59.7 (*C*H_2_^α^); 45.8 (N(*C*H_3_)_2_); 42.6 (NH-*C*H_2_^γ^); 29.2 (*C*H^Dipp^); 28.8 (*C*H^Dipp^); 26.9 (C*H*_2_^β^); 25.3 (*C*H_3_^Dipp^); 24.7 (*C*H_3_^Dipp^); 24.3 (*C*H_3_^Dipp^); 23.1 (*C*H_3_^Dipp^). ^1^H NMR (THF-d_8_, 500 MHz, 295 K) δ: 7.22 (t, ^3^*J* = 7.5 Hz, 1H, Ar*H*^Dipp^); 7.14 (d, ^3^*J* = 7.6 Hz, 2H, Ar*H*^Dipp^); 7.04 (d, ^3^*J* = 7.5 Hz, 2H, Ar*H*^Dipp^); 6.84 (t, ^3^*J* = 7.5 Hz, 1H, Ar*H*^Dipp^); 5.53 (s, 1H, N*H*^Dipp^); 4.84 (t, ^3^*J* = 4.8 Hz, 1H, N*H-*CH_2_^γ^); 3.42 – 3.28 (m, 6H, C*H*^Dipp^ + C*H*_2_^γ^); 2.21 (t, ^3^*J* = 6.1 Hz, 2H, C*H*_2_^α^); 1.89 (s, 6H, N(C*H*_3_)_2_); 1.64 (m, ^3^*J* = 6.3 Hz, 2H, C*H*_2_^β^); 1.31 (d, ^3^*J* = 6.9 Hz, 6H, C*H*_3_^Dipp^); 1.27 (d, ^3^*J* = 6.7 Hz, 6H, C*H*_3_^Dipp^); 1.14 (d, ^3^*J* = 6.6 Hz, 6H, C*H*_3_^Dipp^); 1.07 (d, ^3^*J* = 6.5 Hz, 6H, C*H*_3_^Dipp^). ^13^C NMR (THF-d_8_, 125 MHz, 295 K) δ: 149.2 (Ar_q_^Dipp^); 147.9 (Ar_q_^Gua^); 146.7 (Ar_q_^Dipp^); 141.6 (Ar_q_^Dipp^); 133.5 (Ar_q_^Dipp^); 128.8 (ArH^Dipp^); 124.4 (ArH^Dipp^); 123.3 (ArH^Dipp^); 122.3 (ArH^Dipp^); 60.0 (*C*H_2_^α^); 46.0 (N(*C*H_3_)_2_); 42.4 (NH-*C*H_2_^γ^); 29.2 (*C*H^Dipp^); 29.0 (*C*H^Dipp^); 27.9 (C*H*_2_^β^); 24.9 (*C*H_3_^Dipp^); 24.7 (*C*H_3_^Dipp^); 24.3 (*C*H_3_^Dipp^); 23.0 (*C*H_3_^Dipp^).

MALDI: [M+H]^+^ Calcd. for C_30_H_48_N_4_ 465.39572; found 465.39471.

**Preparation of 13**

**Method A:** *N*,*N*'-bis(2,6-diisopropylphenyl)carbodiimide (12.066 g, 33.29 mmol); 2-[(dimethylamino)methyl]aniline (5.021 g, 33.29 mmol); 3 days; recrystallized from petroleum ether (*ca* 100 ml). Yield of 15.018 g (88%) of off-white crystalline **13**. Single crystals suitable for sc-XRD analysis were obtained by slow evaporation of benzene from saturated solution of **13** at room temperature. Mp 136 - 136.5 °C.

^1^H NMR (C_6_D_6_, 500 MHz, 295 K) δ: 9.51 (d, ^3^*J* = 8.3 Hz, 1H, Ar*H*^DAMB^); 8.42 (s, 1H, N*H*^DAMB^); 7.34 (m, 1H, Ar*H*^DAMB^); 7.32 (d, ^3^*J* = 7.6 Hz, 2H, Ar*H*^Dipp^); 7.20 (t, ^3^*J* = 7.5 Hz, 1H, Ar*H*^Dipp^); 7.02 (t, ^3^*J* = 7.6 Hz, 1H, Ar*H*^Dipp^); 6.92 (d, ^3^*J* = 7.6 Hz, 2H, Ar*H*^Dipp^); 6.90 – 6.79 (m, 2H, Ar*H*^DAMB^); 5.02 (s, 1H, N*H*^Dipp^); 3.65 (m, ^3^*J* = 6.7 Hz, 2H, C*H*^Dipp^); 3.37 (m, ^3^*J* = 6.8 Hz, 2H, C*H*^Dipp^); 2.95 (s, 2H, C*H*_2_); 1.49 – 1.40 (m, 12H, N(C*H*_3_)_2_ + C*H*_3_^Dipp^); 1.37 (d, ^3^*J* = 6.6 Hz, 6H, C*H*_3_^Dipp^); 1.19 (d, ^3^*J* = 6.8 Hz, 6H, C*H*_3_^Dipp^); 1.00 (d, ^3^*J* = 6.7 Hz, 6H, C*H*_3_^Dipp^). ^13^C NMR (C_6_D_6_, 125 MHz, 295 K) δ: 148.7 (Ar_q_^Dipp^); 144.9 (Ar_q_^Dipp^); 144.8 (Ar_q_^Gua^); 141.9 (Ar_q_^DAMB^); 141.1 (Ar_q_^Dipp^); 132.8 (Ar_q_^Dipp^); 130.3 (ArH^DAMB^); 129.1 (ArH^Dipp^); 129.0 (ArH^DAMB^); 125.7 (Ar_q_^DAMB^); 124.3 (ArH^Dipp^); 123.9 (ArH^Dipp^); 123.6 (ArH^Dipp^); 121.3 (ArH^DAMB^); 120.9 (ArH^DAMB^); 64.1 (*C*H_2_); 45.0 (N(*C*H_3_)_2_); 29.3 (*C*H^Dipp^); 29.0 (*C*H^Dipp^); 25.6 (*C*H_3_^Dipp^); 24.7 (*C*H_3_^Dipp^); 24.3 (*C*H_3_^Dipp^); 22.9 (*C*H_3_^Dipp^).

MALDI: [M+H]^+^ Calcd. for C_34_H_48_N_4_ 513.39572; found 513.39596.

**Preparation of 14**

**Method A:** *N*,*N*'-bis(2,6-diisopropylphenyl)carbodiimide (1.580 g, 4.36 mmol); 1,2-diphenylethane-1,2-diamine (0.925 g, 4.36 mmol); 3 days; recrystallized from Et_2_O (*ca* 20 ml). Yield of 2.120 g (85%) of white crystalline **14**. Single crystals suitable for sc-XRD analysis were obtained by cooling a saturated solution of **14** in Et_2_O to -30 °C. Mp 123.5 - 124.5 °C.

^1^H NMR (C_6_D_6_, 500 MHz, 295 K) δ: 7.29 (d, ^3^*J* = 7.6 Hz, 2H, Ar*H*^Ph^); 7.24 (d, ^3^*J* = 7.4 Hz, 1H, Ar*H*^Dipp^); 7.22 – 7.17 (m, 3H, Ar*H*^Ph^ + Ar*H*^Dipp^); 7.15 - 6.99 (br m, 10H, Ar*H*^Ph^ + Ar*H*^Dipp^); 5.46 (t, ^3^*J* = 4.0 Hz, 1H, C*H*^Ph^(NH)Ph); 4.98 (d, ^3^*J* = 4.7 Hz, 1H, CH^Ph^(N*H*)Ph); 4.94 (s, 1H, N*H*^Dipp^); 4.98 (d, ^3^*J* = 4.1 Hz, 1H, C*H*^Ph^(NH_2_)Ph); 3.54 (m, ^3^*J* = 6.8 Hz, 1H, C*H*^Dipp^); 3.32 (m, ^3^*J* = 6.8 Hz, 1H, C*H*^Dipp^); 3.15 (m, ^3^*J* = 6.6 Hz, 1H, C*H*^Dipp^); 3.07 (m, ^3^*J* = 6.8 Hz, 1H, C*H*^Dipp^); 1.40 (d, ^3^*J* = 7.1 Hz, 3H, C*H*_3_^Dipp^); 1.38 (d, ^3^*J* = 7.2 Hz, 3H, C*H*_3_^Dipp^); 1.35 (d, ^3^*J* = 6.7 Hz, 3H, C*H*_3_^Dipp^); 1.30 (d, ^3^*J* = 7.0 Hz, 3H, C*H*_3_^Dipp^); 1.05 (d, ^3^*J* = 6.7 Hz, 3H, C*H*_3_^Dipp^); 0.97 (d, ^3^*J* = 6.7 Hz, 3H, C*H*_3_^Dipp^); 0.93 – 0.88 (m, 6H, 2x C*H*_3_^Dipp^). ^13^C NMR (C_6_D_6_, 125 MHz, 295 K) δ: 148.5 (Ar_q_^Dipp^); 148.2 (Ar_q_^Dipp^); 145.8 (Ar_q_^Dipp^); 145.2 (Ar_q_^Gua^); 144.1 (Ar_q_^Ph^); 143.9 (Ar_q_^Ph^); 141.7 (Ar_q_^Dipp^); 141.0 (Ar_q_^Dipp^); 133.0 (Ar_q_^Dipp^); 129.0 (ArH^Dipp^); 128.8 (ArH^Ph^); 128.7 (ArH^Ph^); 127.6 (ArH^Ph^); 127.5 (ArH^Ph^); 127.3 (ArH^Ph^); 127.2 (ArH^Ph^); 124.3 (ArH^Dipp^); 124.2 (ArH^Dipp^); 123.8 (ArH^Dipp^); 123.4 (ArH^Dipp^); 123.2 (ArH^Dipp^); 60.8 (*C*H^Ph^(NH)Ph); 60.1 (CH^Ph^(NH_2_)Ph); 29.2 (*C*H^Dipp^); 29.0 (*C*H^Dipp^); 28.8 (2x *C*H^Dipp^); 25.8 (*C*H_3_^Dipp^); 25.0 (*C*H_3_^Dipp^); 24.8 (2x *C*H_3_^Dipp^); 23.6 (*C*H_3_^Dipp^); 23.2 (*C*H_3_^Dipp^); 23.0 (*C*H_3_^Dipp^); 22.6 (*C*H_3_^Dipp^).

MALDI: [M+H]^+^ Calcd. for C_39_H_50_N_4_ 575.41137; found 575.41147.

**Preparation of 15**

**Method A:** *N*,*N*'-bis(2,6-diisopropylphenyl)carbodiimide (8.000 g, 22.07 mmol); 1,2-diphenylethane-1,2-diamine (2.343 g, 11.03 mmol); 5 days; recrystallized from the mixture of Et_2_O:hexane (3:1, *ca* 70 ml). Yield of 4.308 g (42%) of white crystalline **15**. Single crystals suitable for sc-XRD analysis were obtained by slow evaporation of hexane from saturated solution of **15** at room temperature. Mp 123.5 - 124.5 °C. Mp 247.5 - 249 °C.

^1^H NMR (C_6_D_6_, 500 MHz, 295 K) δ: 7.25 (d, ^3^*J* = 7.1 Hz, 2H, Ar*H*^Dipp^); 7.16 – 7.12 (m, 4H, Ar*H*^Dipp^ +Ar*H*^Ph^); 7.12 – 7.07 (m, 4H, Ar*H*^Dipp^); 7.01 (d, ^3^*J* = 7.2 Hz, 2H, Ar*H*^Dipp^); 6.96 (t, ^3^*J* = 7.4 Hz, 4H, Ar*H*^Ph^); 6.91 (d, ^3^*J* = 7.4 Hz, 2H, Ar*H*^Dipp^); 6.68 (d, ^3^*J* = 7.3 Hz, 4H, Ar*H*^Dipp^); 5.56 (t, ^3^*J* = 6.0 Hz, 2H, C*H*^Ph^); 4.73 (s, 2H, N*H*^Dipp^); 4.24 (d, ^3^*J* = 6.0 Hz, 2H, C*H*^Ph^-N*H*); 3.49 (m, ^3^*J* = 6.7 Hz, 2H, C*H*^Dipp^); 3.31 (m, ^3^*J* = 6.7 Hz, 2H, C*H*^Dipp^); 3.17 (m, ^3^*J* = 6.8 Hz, 2H, C*H*^Dipp^); 2.77 (m, ^3^*J* = 6.8 Hz, 2H, C*H*^Dipp^); 1.45 (d, ^3^*J* = 6.8 Hz, 12H, 2x C*H*_3_^Dipp^); 1.37 (d, ^3^*J* = 6.7 Hz, 6H, C*H*_3_^Dipp^); 1.18 (d, ^3^*J* = 7.0 Hz, 6H, C*H*_3_^Dipp^); 1.03 (d, ^3^*J* = 6.7 Hz, 6H, C*H*_3_^Dipp^); 0.86 (d, ^3^*J* = 6.9 Hz, 6H, C*H*_3_^Dipp^); 0.84 (d, ^3^*J* = 6.7 Hz, 6H, C*H*_3_^Dipp^); 0.66 (d, ^3^*J* = 6.5 Hz, 6H, C*H*_3_^Dipp^). ^13^C NMR (C_6_D_6_, 125 MHz, 295 K) δ: 148.7 (Ar_q_^Dipp^); 148.5 (Ar_q_^Dipp^); 145.1 (Ar_q_^Dipp^); 144.9 (Ar_q_^Gua^); 141.3 (Ar_q_^Dipp^); 140.7 (Ar_q_^Dipp^); 140.6 (Ar_q_^Ph^); 132.6 (Ar_q_^Dipp^); 129.3 (ArH^Dipp^); 128.4 (ArH^Ph^); 128.2 (ArH^Ph^); 127.4 (ArH^Ph^); 123.6 (2x ArH^Dipp^); 123.7 (ArH^Dipp^); 123.5 (ArH^Dipp^); 123.3 (ArH^Dipp^); 57.9 (*C*H^Ph^(NH)Ph); 29.3 (*C*H^Dipp^); 28.9 (*C*H^Dipp^); 28.8 (*C*H^Dipp^); 28.4 (*C*H^Dipp^); 25.6 (*C*H_3_^Dipp^); 24.8 (2x *C*H_3_^Dipp^); 24.2 (2x *C*H_3_^Dipp^); 22.9 (*C*H_3_^Dipp^); 22.7 (*C*H_3_^Dipp^); 22.6 (*C*H_3_^Dipp^).

MALDI: [M+H]^+^ Calcd. for C_64_H_84_N_6_ 937.68357; found 937.68602.

**Preparation of 16**

**Method A:** *N*,*N*'-bis(2,6-diisopropylphenyl)carbodiimide (2.000 g, 5.52 mmol); 1,3-di(aminomethyl)benzene (0.36 ml, ρ = 1.040 g.cm^-3^, 2.76 mmol); 3 days; recrystallized from hexane (*ca* 30 ml). Further purification was performed via protonated **16** obtained by addition of HCl (0.54 ml of 35% aqueous solution, ρ = 1.180 g.cm^-3^, 6.07 mmol) in hexane (30 ml). Corresponding guanidinium salt was extracted by THF (50 ml), transformed to **16** using Et_3_N (0.85 ml, ρ = 0.727 g.cm^-3^, 6.07 mmol) in hexane (50 ml) for 30 minutes with subsequent recrystallization from hexane (*ca* 25 ml). Yield of 1.829 g (77%) of white solid **16**. Mp 62 - 64 °C.

^1^H NMR (C_6_D_6_, 500 MHz, 295 K) δ: 7.29 (d, ^3^*J* = 7.8 Hz, 4H, Ar*H*^Dipp^); 7.23 – 7.16 (m, 4H, Ar*H*^Dipp^ +Ar*H*^MXDA^); 7.12 (t, ^3^*J* = 7.5 Hz, 1H, Ar*H*^MXDA^); 7.06 (t, ^3^*J* = 7.5 Hz, 2H, Ar*H*^Dipp^); 7.01 (s, 1H, Ar*H*^MXDA^); 6.96 (d, ^3^*J* = 7.6 Hz, 4H, Ar*H*^Dipp^); 4.85 (s, 2H, N*H*^Dipp^); 4.44 (d, ^3^*J* = 5.5 Hz, 4H, C*H*_2_); 3.73 (t, ^3^*J* = 5.6 Hz, 2H, CH_2_-N*H*); 3.49 (m, ^3^*J* = 6.8 Hz, 4H, C*H*^Dipp^); 3.29 (m, ^3^*J* = 6.8 Hz, 4H, C*H*^Dipp^); 1.41 (d, ^3^*J* = 7.0 Hz, 12H, C*H*_3_^Dipp^); 1.31 (d, ^3^*J* = 6.6 Hz, 12H, C*H*_3_^Dipp^); 1.16 (d, ^3^*J* = 6.7 Hz, 12H, C*H*_3_^Dipp^); 0.97 (d, ^3^*J* = 6.6 Hz, 12H, C*H*_3_^Dipp^). ^13^C NMR (C_6_D_6_, 125 MHz, 295 K) δ: 148.6 (Ar_q_^Dipp^); 146.6 (Ar_q_^Gua^); 145.3 (Ar_q_^Dipp^); 141.5 (Ar_q_^Dipp^); 141.2 (Ar_q_^MXDA^); 132.5 (Ar_q_^Dipp^); 129.3 (ArH^Dipp^); 128.7 (ArH^MXDA^); 127.8 (ArH^MXDA^); 126.6 (ArH^MXDA^); 124.5 (ArH^Dipp^); 123.8 (ArH^Dipp^); 123.5 (ArH^Dipp^); 45.5 (*C*H_2_); 29.2 (*C*H^Dipp^); 28.7 (*C*H^Dipp^); 25.2 (*C*H_3_^Dipp^); 24.6 (*C*H_3_^Dipp^); 24.2 (*C*H_3_^Dipp^); 23.1 (*C*H_3_^Dipp^).

MALDI: [M+H]^+^ Calcd. for C_58_H_80_N_6_ 861.65172; found 861.65451.

**Preparation of 17**

**Method A:** *N*,*N*'-bis(2,6-diisopropylphenyl)carbodiimide (1.640 g, 4.52 mmol); *N*'-(2-aminoethyl)ethane-1,2-diamine (0.25 ml, ρ = 0.946 g.cm^-3^, 4.52 mmol); 3 days; recrystallized from Et_2_O (*ca* 30 ml). Yield of 0.991 g (53%) of white crystalline **17**. Single crystals suitable for sc-XRD analysis were obtained by cooling a saturated solution of **17** in Et_2_O to -30 °C. Mp 130.5 - 131.5 °C.

^1^H NMR (C_6_D_6_, 500 MHz, 295 K) δ: 7.30 (d, ^3^*J* = 7.5 Hz, 4H, Ar*H*^Dipp^); 7.18 (t, ^3^*J* = 7.5 Hz, 2H, Ar*H*^Dipp^); 7.08 (t, ^3^*J* = 7.7 Hz, 2H, Ar*H*^Dipp^); 6.98 (d, ^3^*J* = 7.6 Hz, 4H, Ar*H*^Dipp^); 4.79 (s, 2H, N*H*^Dipp^); 3.67 (t, ^3^*J* = 5.2 Hz, 2H, C^Gua^-N*H*-CH_2_^α^); 3.49 (m, ^3^*J* = 6.6 Hz, 4H, C*H*^Dipp^); 3.33 – 3.13 (m, 8H, C*H*^Dipp^ + C*H*_2_^α^); 2.46 (t, ^3^*J* = 5.7 Hz, 2H, CH_2_^α^-C*H*_2_^β^); 1.43 (d, ^3^*J* = 6.8 Hz, 12H, C*H*_3_^Dipp^); 1.40 (d, ^3^*J* = 6.6 Hz, 12H, C*H*_3_^Dipp^); 1.23 (d, ^3^*J* = 6.7 Hz, 12H, C*H*_3_^Dipp^); 0.99 (d, ^3^*J* = 6.4 Hz, 12H, C*H*_3_^Dipp^); 0.49 (br s , 1H, CH_2_^β^-N*H*-CH_2_^β^). ^13^C NMR (C_6_D_6_, 125 MHz, 295 K) δ: 148.5 (Ar_q_^Dipp^); 147.0 (Ar_q_^Gua^); 145.4 (Ar_q_^Dipp^); 141.5 (Ar_q_^Dipp^); 132.7 (Ar_q_^Dipp^); 129.1 (ArH^Dipp^); 124.4 (ArH^Dipp^); 123.8 (ArH^Dipp^); 123.4 (ArH^Dipp^); 49.6 (CH_2_^α^-*C*H_2_^β^); 41.7 (*C*H_2_^α^-CH_2_^β^); 29.1 (*C*H^Dipp^); 28.7 (*C*H^Dipp^); 25.2 (*C*H_3_^Dipp^); 24.5 (*C*H_3_^Dipp^); 24.4 (*C*H_3_^Dipp^); 23.0 (*C*H_3_^Dipp^).

MALDI: [M+H]^+^ Calcd. for C_54_H_81_N_7_ 828.66317; found 828.66363.

**Preparation of 18**

**Method B:** To prevent oxidation of NCN moiety of starting *N,N'*‑dicyclohexylcarbodiimide to corresponding urea, handling and synthetic procedure were carried out under inert argon atmosphere. *N,N'*‑dicyclohexylcarbodiimide (1.000 g, 4.85 mmol); aniline (0.44 ml, ρ = 1.027 g.cm^-3^, 4.85 mmol); HCl (0.81 ml of 3M CPME solution, 4.85 mmol); Et_3_N (0.74 ml, ρ = 0.727 g.cm^-3^, 5.34 mmol). Toluene was evaporated under vacuum, and solid matter was extracted with a mixture of solvents - Et_2_O (80 ml), toluene (100 ml), and THF (70 ml). After, all volatiles were evaporated under vacuum and crude **18** was recrystallized from mixture THF:Et_2_O (1:1 ratio, *ca* 60 ml) to give 1.205 g (83%) of white crystalline **18**. Single crystals suitable for sc-XRD analysis were obtained by slow evaporation of Et_2_O from a saturated solution of **18** at room temperature. Mp 158 - 159 °C.

^1^H NMR (C_6_D_6_, 400 MHz, 295 K) δ: 7.25 (t, ^3^*J* = 7.7 Hz, 2H, Ar*H*^Ph^); 7.20 (dd, ^3^*J* = 8.3 Hz, ^4^*J* = 1.4 Hz, 2H, Ar*H*^Ph^); 6.93 (tt, ^3^*J* = 7.2 Hz, ^4^*J* = 1.3 Hz, 1H, Ar*H*^Ph^); 4.21 (br s, 2H, N*H*); 3.53 (br s, 2H, C*H*^Cy^); 2.00 – 1.85 (m, 4H, C*H*_2_^Cy^); 1.58 – 1.44 (m, 4H, C*H*_2_^Cy^); 1.43 – 1.32 (m, 2H, C*H*_2_^Cy^); 1.21 – 1.06 (m, 4H, C*H*_2_^Cy^); 1.03 – 0.84 (m, 6H, C*H*_2_^Cy^). ^13^C NMR (C_6_D_6_, 100 MHz, 295 K) δ: 150.7 (br s, Ar_q_^Ph^ + Ar_q_^Gua^); 130.0 (ArH^Ph^); 124.1 (ArH^Ph^); 122.2 (ArH^Ph^); 51.1 (*C*H^Cy^); 34.2 (*C*H_2_^Cy^); 26.2 (*C*H_2_^Cy^); 25.6 (*C*H_2_^Cy^). ^1^H NMR (THF-d_8_, 400 MHz, 295 K) δ: 7.19 – 7.07 (m, 2H, Ar*H*^Ph^); 6.84 – 6.71 (m, 3H, Ar*H*^Ph^); 4.63 (br s, 2H, N*H*); 3.55 – 3.44 (m, 2H, C*H*^Cy^); 2.02 – 1.90 (m, 4H, C*H*_2_^Cy^); 1.71 – 1.62 (m, 4H, C*H*_2_^Cy^); 1.61 – 1.51 (m, 2H, C*H*_2_^Cy^); 1.36 – 1.23 (m, 4H, C*H*_2_^Cy^); 1.20 – 1.04 (m, 6H, C*H*_2_^Cy^). ^13^C NMR (THF-d_8_, 125 MHz, 295 K) δ: 151.5 (br s, Ar_q_^Gua^); 151.1 (Ar_q_^Ph^); 129.7 (ArH^Ph^); 123.9 (ArH^Ph^); 121.4 (ArH^Ph^); 51.3 (*C*H^Cy^); 34.6 (*C*H_2_^Cy^); 26.9 (*C*H_2_^Cy^); 25.2 (*C*H_2_^Cy^).

MALDI: [M+H]^+^ Calcd. for C_19_H_29_N_3_ 300.24397; found 300.24377.

**Preparation of 19**

**Method B:** To prevent oxidation of NCN moiety of starting *N,N'*‑dicyclohexylcarbodiimide to corresponding urea, handling and synthetic procedure were carried out under inert argon atmosphere. *N,N'*‑dicyclohexylcarbodiimide (4.000 g, 19.39 mmol); 2-methoxyaniline (2.19 ml, ρ = 1.092 g.cm^-3^, 19.39 mmol); HCl (9.70 ml of 2M Et_2_O solution, 19.39 mmol). Corresponding crude guanidinium chloride **19**^.^HCl was filtered off and recrystallized from Et_2_O (500 ml) to give 6.526 g (92%) of pure off-white powder of **19**^.^HCl (properties and NMR parameters are described below).

A portion of **19**^.^HCl (2.000 g, 5.47 mmol) was transformed to crude **19** using *^t^*BuOK (0.613 g, 5.47 mmol) in THF (150 ml) and recrystallized from Et_2_O (*ca* 40 ml). Yield 1.576 g (87%) of white crystalline **19**. Single crystals suitable for sc-XRD analysis were obtained by slow evaporation of solvent(s) from saturated Et_2_O:hexane (1:1 ratio) solution of **19** at room temperature. Mp 113.5 - 114 °C.

^1^H NMR (C_6_D_6_, 500 MHz, 295 K) δ: 7.22 (d, ^3^*J* = 7.1 Hz, 1H, Ar*H*^OAS^); 7.00 (t, ^3^*J* = 7.3 Hz, 1H, Ar*H*^OAS^); 6.95 (t, ^3^*J* = 7.2 Hz, 1H, Ar*H*^OAS^); 6.80 (d, ^3^*J* = 7.8 Hz, 1H, Ar*H*^OAS^); 3.55 (br s, 4H, C*H*^Cy^ + N*H*); 3.47 (s, 3H, OC*H*_3_); 1.97 (br s, 4H, C*H*_2_^Cy^); 1.59 – 1.44 (m, 4H, C*H*_2_^Cy^); 1.43 – 1.32 (m, 2H, C*H*_2_^Cy^); 1.21 – 1.07 (m, 4H, C*H*_2_^Cy^); 1.00 – 0.79 (m, 6H, C*H*_2_^Cy^). ^13^C NMR (C_6_D_6_, 125 MHz, 295 K) δ: 153.3 (Ar_q_^OAS^-OCH_3_); 149.8 (Ar_q_^Gua^); 141.3 (Ar_q_^OAS^); 125.6 (ArH^OAS^); 122.6 (ArH^OAS^); 122.5 (ArH^OAS^); 113.6 (ArH^OAS^); 55.9 (O*C*H_3_); 50.9 (*C*H^Cy^); 34.4 (*C*H_2_^Cy^); 26.3 (*C*H_2_^Cy^); 25.6 (*C*H_2_^Cy^). ^1^H NMR (THF-d_8_, 500 MHz, 295 K) δ: 6.82 – 6.77 (m, 1H, Ar*H*^OAS^); 6.77 – 6.70 (m, 2H, Ar*H*^OAS^); 6.56 (br s, 1H, Ar*H*^OAS^); 3.98 (br s, 2H, N*H*); 3.68 (s, 3H, OC*H*_3_); 3.45 (br s, 2H, C*H*^Cy^); 2.06 – 1.94 (m, 4H, C*H*_2_^Cy^); 1.71 – 1.61 (m, 4H, C*H*_2_^Cy^); 1.61 – 1.52 (m, 2H, C*H*_2_^Cy^); 1.36 – 1.22 (m, 4H, C*H*_2_^Cy^); 1.19 – 1.09 (m, 2H, C*H*_2_^Cy^); 1.09 – 0.98 (m, 4H, C*H*_2_^Cy^). ^13^C NMR (THF-d_8_, 125 MHz, 295 K) δ: 153.4 (Ar_q_^OAS^-OCH_3_); 150.3 (Ar_q_^Gua^); 142.0 (Ar_q_^OAS^); 125.4 (ArH^OAS^); 122.1 (ArH^OAS^); 121.8 (ArH^OAS^); 113.7 (ArH^OAS^); 56.1 (O*C*H_3_); 51.2 (*C*H^Cy^); 34.8 (*C*H_2_^Cy^); 27.0 (*C*H_2_^Cy^); 26.3 (*C*H_2_^Cy^).

MALDI: [M+H]^+^ Calcd. for C_20_H_31_N_3_O 330.25454; found 330.25422.

**Properties of 19**^.^HCl

Preparation is associated with paragraph **Preparation of 19 Method B** (described above). Single crystals suitable for sc-XRD analysis were obtained by slow evaporation of toluene from saturated solution of **19**^.^HCl at room temperature. Mp 142 - 143.5 °C.

^1^H NMR (C_6_D_6_, 500 MHz, 295 K) δ: 10.53 (br s, 1H, N*H*Cl); 8.57 (br s, 2H, N*H*); 7.43 (d, ^3^*J* = 6.3 Hz, 1H, Ar*H*^OAS^); 6.94 (t, ^3^*J* = 7.7 Hz, 1H, Ar*H*^OAS^); 6.78 (t, ^3^*J* = 7.5 Hz, 1H, Ar*H*^OAS^); 6.49 (d, ^3^*J* = 8.2 Hz, 1H, Ar*H*^OAS^); 4.02 (br s, 2H, C*H*^Cy^); 3.35 (s, 3H, OC*H*_3_); 1.98 (br s, 4H, C*H*_2_^Cy^); 1.75 – 1.54 (br m, 8H, C*H*_2_^Cy^); 1.47 – 1.21 (br m, 6H, C*H*_2_^Cy^); 1.16 – 1.03 (m, 2H, C*H*_2_^Cy^). ^13^C NMR (C_6_D_6_, 125 MHz, 295 K) δ: 156.0 (Ar_q_^Gua^); 154.1 (Ar_q_^OAS^-OCH_3_); 127.8 (Ar_q_^OAS^); 127.7 (ArH^OAS^); 126.9 (ArH^OAS^); 121.5 (ArH^OAS^); 112.4 (ArH^OAS^); 55.8 (O*C*H_3_); 53.0 (*C*H^Cy^); 33.4 (*C*H_2_^Cy^); 26.0 (*C*H_2_^Cy^); 25.5 (*C*H_2_^Cy^).

MALDI: [M+H]^+^ Calcd. for C_20_H_32_ClN_3_O 366.23067; found 330.25451. The result corresponds to the basic form without HCl, which was probably cleaved during ionization. Calcd. for the basic form C_32_H_43_N_3_O is 330.25454.

**Preparation of 20**

**Method B:** *N,N'*‑bis(2,6‑diisopropylphenyl)carbodiimide (1.000 g, 2.76 mmol); aniline (0.25 ml, ρ = 1.027 g.cm^-3^, 2.76 mmol); HCl (0.24 ml of 35% aqueous solution, ρ = 1.180 g.cm^-3^, 2.76 mmol); Et_3_N (0.42 ml, ρ = 0.727 g.cm^-3^, 3.04 mmol); recrystallized from hexane (*ca* 20 ml). Yield of 1.106 g (88%) of white crystalline **20**. Single crystals suitable for sc-XRD analysis were obtained by slow evaporation of hexane from saturated solution of **20** at room temperature. Mp 129 - 130 °C. Analysis, calculated: C 81.71%, H 9.07%, N 9.22%; found: C 82.22±0.07, H 9.10±0.13, N 9.24±0.07.

**Method C:** aniline (0.13 ml, ρ = 1.027 g.cm^-3^, 1.38 mmol). Sub-stoichiometric amounts of 0.1 and 0.5 equivalent of HCl solution were tested in a catalytic guanylation approach to monitor activity in time scales. Details of NMR yield, guanylation progress and reaction time are given in the discussion part of the main text and Figure S8.

NMR spectra of **20** show the presence of two tautomers at THF-d_8_ and C_6_D_6_. Signals of the second(minor) tautomer of **20** at C_6_D_6_ and THF-d_8_ were not assigned (and marked with black dots on NMR spectra – see Figures S44-S47). **NMR spectra @C_6_D_6_ at 295 K for major** **tautomer** - ^1^H NMR (C_6_D_6_, 500 MHz, 295 K) δ: 7.57 (d, 2H, ^3^*J* = 7.9 Hz, Ar*H*^ANL^); 7.30 (d, 2H, ^3^*J* = 7.6 Hz, Ar*H*^Dipp^); 7.19 (t, 1H, ^3^*J* = 7.6 Hz, Ar*H*^Dipp^); 7.13 (t, 1H, ^3^*J* = 7.5 Hz, Ar*H*^Dipp^); 7.07 (t, 2H, ^3^*J* = 7.8 Hz, Ar*H*^ANL^); 7.00 (d, 2H, ^3^*J* = 7.6 Hz, Ar*H*^Dipp^); 6.80 (t, 1H, ^3^*J* = 7.4 Hz, Ar*H*^ANL^); 5.61 (s, 1H, N*H*^ANL^); 4.99 (s, 1H, N*H*^Dipp^); 3.57 (m, 2H, ^3^*J* = 6.7 Hz, C*H*^Dipp^); 3.31 (m, 2H, ^3^*J* = 6.8 Hz, C*H*^Dipp^); 1.40 (d, 6H, ^3^*J* = 6.9 Hz, C*H*_3_^Dipp^); 1.35 (d, 6H, ^3^*J* = 6.7 Hz, C*H*_3_^Dipp^); 1.15 (d, 6H, ^3^*J* = 6.4 Hz, C*H*_3_^Dipp^); 0.98 (d, 6H, ^3^*J* = 6.4 Hz, C*H*_3_^Dipp^). ^13^C NMR (C_6_D_6_, 125 MHz, 295 K) δ: 148.8 (Ar_q_^Dipp^); 144.2 (Ar_q_^Dipp^); 143.7 (Ar_q_^Gau^); 141.1 (Ar_q_^Dipp^); 141.0 (Ar_q_^ANL^); 132.0 (Ar_q_^Dipp^); 129.6 (ArH^Dipp^); 129.4 (ArH^ANL^); 124.7 (ArH^Dipp^); 124.0 (ArH^Dipp^); 123.9 (ArH^Dipp^); 122.4 (ArH^ANL^); 119.1 (ArH^ANL^); 29.2 (*C*H^Dipp^); 28.8 (*C*H^Dipp^); 25.3 (*C*H_3_^Dipp^); 24.4 (2x *C*H_3_^Dipp^); 23.0 (*C*H_3_^Dipp^). **NMR spectra @THF-d_8_ at 295 K for major tautomer** - ^1^H NMR (THF-d_8_, 500 MHz, 295 K) δ: 7.59 (d, 2H, ^3^*J* = 7.7 Hz, Ar*H*^ANL^); 7.33 (t, 1H, ^3^*J* = 7.4 Hz, Ar*H*^Dipp^); 7.25 (d, 2H, ^3^*J* = 7.5 Hz, Ar*H*^Dipp^); 7.16 - 7.08 (br m, 4H, incld: triplet with ^3^*J* = 7.9 Hz for 2H at 7.12 ppm for Ar*H*^ANL^ + doublet with ^3^*J* = 7.5 Hz for 2H at 7.11 ppm for Ar*H*^Dipp^); 6.93 (t, 1H, ^3^*J* = 7.5 Hz, Ar*H*^Dipp^); 6.84 (t, 1H, ^3^*J* = 7.1 Hz, Ar*H*^ANL^); 5.97 (s, 1H, N*H*^Dipp^); 5.72 (s, 1H, N*H*^ANL^); 3.48 – 3.37 (br m, 4H, two C*H*^Dipp^ multiplets); 1.34 (d, 6H, ^3^*J* = 6.7 Hz, C*H*_3_^Dipp^); 1.25 (d, 6H, ^3^*J* = 6.3 Hz, C*H*_3_^Dipp^); 1.16 (d, 6H, ^3^*J* = 6.6 Hz, C*H*_3_^Dipp^); 1.13 (d, 6H, ^3^*J* = 6.4 Hz, C*H*_3_^Dipp^). ^13^C NMR (THF-d_8_, 125 MHz, 295 K) δ: 149.4 (Ar_q_^Dipp^); 145.0 (Ar_q_^Dipp^); 144.3 (Ar_q_^Gua^); 141.9 (Ar_q_^ANL^); 141.1 (Ar_q_^Dipp^); 133.4 (Ar_q_^Dipp^); 129.7 (ArH^Dipp^); 129.2 (ArH^ANL^); 124.9 (ArH^Dipp^); 123.6 (ArH^Dipp^); 123.1 (ArH^Dipp^); 122.2 (ArH^ANL^); 119.7 (ArH^ANL^); 29.4 (*C*H^Dipp^); 29.2 (*C*H^Dipp^); 25.2 (*C*H_3_^Dipp^); 24.5 (*C*H_3_^Dipp^); 24.4 (*C*H_3_^Dipp^); 23.0 (*C*H_3_^Dipp^).

MALDI: [M+H]^+^ Calcd. for C_31_H_41_N_3_ 456.33787; found 456.33792.

**Preparation of 21**

**Method B:** *N,N'*‑bis(2,6‑diisopropylphenyl)carbodiimide (1.562 g, 4.31 mmol); 2-methoxyaniline (0.49 ml, ρ = 1.092 g.cm^-3^, 4.31 mmol); HCl (0.38 ml of 35% aqueous solution, ρ = 1.180 g.cm^-3^, 4.31 mmol). Corresponding crude guanidinium chloride **21**^.^HCl was filtered off and recrystallized from THF (*ca* 20 ml) to give 2.034 g (89%) of pure white powder of **21**^.^HCl (properties and NMR parameters are described below).

A portion of **21**^.^HCl (0.514 g, 0.98 mmol) was transformed to crude **21** using Et_3_N (0.15 ml, ρ = 0.727 g.cm^-3^, 1.08 mmol) in hexane (20 ml) for 30 minutes and recrystallized from hexane (*ca* 15 ml). Yield 0.369 g (77%) of white crystalline **21**. Single crystals suitable for sc-XRD analysis were obtained by slow evaporation of hexane from saturated solution of **21** at room temperature. Mp 163.5 - 165 °C. Analysis, calculated: C 79.13%, H 8.92%, N 8.65%; found: C 79.45±0.07, H 9.01±0.07, N 8.45±0.02.

**Method C:** 2-methoxyaniline (0.16 ml, ρ = 1.092 g.cm^-3^, 1.38 mmol). Sub-stoichiometric amounts of 0.01, 0.05, 0.1, 0.3, 0.5 and 0.7 equivalent of HCl solution were tested in a catalytic guanylation approach to monitor activity in time scales. Details of NMR yields, guanylation progress and reaction time are given in the discussion part of the main text and Figure S9.

^1^H NMR (C_6_D_6_, 500 MHz, 295 K) δ: 9.39 (d, ^3^*J* = 7.9 Hz, 1H, Ar*H*^OAS^); 7.32 (d, ^3^*J* = 7.6 Hz, 2H, Ar*H*^Dipp^); 7.20 (t, ^3^*J* = 7.6 Hz, 1H, Ar*H*^Dipp^); 7.17 (t, ^3^*J* = 7.8 Hz, 1H, Ar*H*^Dipp^); 7.05 (d, ^3^*J* = 7.8 Hz, 2H, Ar*H*^Dipp^); 7.03 (t, ^3^*J* = 7.6 Hz, 1H, Ar*H*^OAS^); 6.77 (td, ^3^*J* = 7.8 Hz, ^4^*J* = 1.0 Hz, 1H, Ar*H*^OAS^); 6.71 (s, 1H, N*H*^OAS^); 6.39 (m, ^3^*J* = 8.0 Hz, 1H, Ar*H*^OAS^); 5.07 (s, 1H, N*H*^Dipp^); 3.63 (m, ^3^*J* = 6.8 Hz, 2H, C*H*^Dipp^); 3.38 (m, ^3^*J* = 6.7 Hz, 2H, C*H*^Dipp^); 2.99 (s, 3H, OC*H*_3_); 1.42 (d, ^3^*J* = 6.8 Hz, 6H, C*H*_3_^Dipp^); 1.37 (d, ^3^*J* = 6.7 Hz, 6H, C*H*_3_^Dipp^); 1.19 (d, ^3^*J* = 6.1 Hz, 6H, C*H*_3_^Dipp^); 1.00 (d, ^3^*J* = 6.0 Hz, 6H, C*H*_3_^Dipp^). ^13^C NMR (C_6_D_6_, 125 MHz, 295 K) δ: 148.9 (Ar_q_^Dipp^); 147.9 (Ar_q_^OAS^-OCH_3_); 144.4 (Ar_q_^Dipp^); 144.0 (Ar_q_^Gua^); 141.1 (Ar_q_^Dipp^); 132.3 (Ar_q_^Dipp^); 131.0 (Ar_q_^OAS^-NH); 129.3 (ArH^Dipp^); 124.5 (ArH^Dipp^); 124.0 (ArH^Dipp^); 123.9 (ArH^Dipp^); 122.2 (ArH^OAS^); 121.4 (ArH^OAS^); 119.1 (ArH^OAS^); 110.2 (ArH^OAS^); 55.4 (O*C*H_3_); 29.2 (*C*H^Dipp^); 28.9 (*C*H^Dipp^); 25.4 (*C*H_3_^Dipp^); 24.6 (*C*H_3_^Dipp^); 24.1 (*C*H_3_^Dipp^); 23.0 (*C*H_3_^Dipp^). ^1^H NMR (THF-d_8_, 500 MHz, 295 K) δ: 8.84 (d, ^3^*J* = 7.9 Hz, 1H, Ar*H*^OAS^); 7.33 (t, ^3^*J* = 7.6 Hz, 1H, Ar*H*^Dipp^); 7.24 (d, ^3^*J* = 7.7 Hz, 2H, Ar*H*^Dipp^); 7.11 (d, ^3^*J* = 7.6 Hz, 2H, Ar*H*^Dipp^); 6.93 (t, ^3^*J* = 7.6 Hz, 1H, Ar*H*^Dipp^); 6.84-6.78 (m, 1H, Ar*H*^OAS^); 6.78-6.71 (m, 2H, Ar*H*^OAS^); 6.48 (s, 1H, N*H*^OAS^); 5.99 (s, 1H, N*H*^Dipp^); 3.53 (s, 3H, OC*H*_3_); 3.45 – 3.35 (m, 4H, 2x C*H*^Dipp^); 1.34 (d, ^3^*J* = 6.9 Hz, 6H, C*H*_3_^Dipp^); 1.20 (d, ^3^*J* = 6.8 Hz, 6H, C*H*_3_^Dipp^); 1.16 (d, ^3^*J* = 6.7 Hz, 6H, C*H*_3_^Dipp^); 1.11 (d, ^3^*J* = 6.8 Hz, 6H, C*H*_3_^Dipp^). ^13^C NMR (THF-d_8_, 125 MHz, 295 K) δ: 149.5 (Ar_q_^Dipp^); 148.4 (Ar_q_^OAS^-OCH_3_); 145.1 (Ar_q_^Dipp^); 144.4 (Ar_q_^Gua^); 141.1 (Ar_q_^Dipp^); 133.5 (Ar_q_^Dipp^); 131.6 (Ar_q_^OAS^-NH); 129.5 (ArH^Dipp^); 124.7 (ArH^Dipp^); 123.6 (ArH^Dipp^); 123.1 (ArH^Dipp^); 121.8 (ArH^OAS^); 121.0 (ArH^OAS^); 118.7 (ArH^OAS^); 110.4 (ArH^OAS^); 56.0 (O*C*H_3_); 29.3 (*C*H^Dipp^); 29.2 (*C*H^Dipp^); 25.2 (*C*H_3_^Dipp^); 24.6 (*C*H_3_^Dipp^); 24.2 (*C*H_3_^Dipp^); 23.0 (*C*H_3_^Dipp^).

MALDI: [M+H]^+^ Calcd. for C_32_H_43_N_3_O 486.34844; found 486.34800.

**Properties of 21**^.^HCl

Preparation is associated with paragraph **Preparation of 21 Method B** (described above). Single crystals suitable for sc-XRD analysis were obtained by slow evaporation of THF from saturated solution of **21**^.^HCl at room temperature. Mp 238 - 239 °C. Analysis, calculated: C 73.61%, H 8.49%, N 8.05%, Cl 6,79%; found: C 73.70±0.06, H 8.67±0.03, N 7.95±0.04, Cl 6.74±0.32.

^1^H NMR (C_6_D_6_, 500 MHz, 295 K) δ: 13.14 (s, 2H, N*H*^Dipp^); 7.01 (t, ^3^*J* = 7.6 Hz, 2H, Ar*H*^Dipp^); 6.90 (d, ^3^*J* = 7.7 Hz, 4H, Ar*H*^Dipp^); 6.51-6.43 (m, 2H, Ar*H*^OAS^); 6.14 (t, ^3^*J* = 7.5 Hz, 1H, Ar*H*^OAS^); 5.87 (d, ^3^*J* = 8.1 Hz, 1H, Ar*H*^OAS^); 5.59 (s, 1H, N*H*^OAS^); 3.55 (m, ^3^*J* = 6.8 Hz, 4H, C*H*^Dipp^); 2.92 (s, 3H, OC*H*_3_); 1.42 (d, ^3^*J* = 6.8 Hz, 12H, C*H*_3_^Dipp^); 1.18 (d, ^3^*J* = 6.9 Hz, 12H, C*H*_3_^Dipp^). ^13^C NMR (C_6_D_6_, 125 MHz, 295 K) δ: 155.5 (Ar_q_^Gua^); 152.9 (Ar_q_^OAS^-OCH_3_); 147.5 (Ar_q_^Dipp^); 130.6 (Ar_q_^Dipp^); 129.7 (ArH^Dipp^); 128.4 (ArH^OAS^); 127.5 (ArH^OAS^); 124.3 (ArH^Dipp^); 122.8 (Ar_q_^OAS^-NH); 120.2 (ArH^OAS^); 110.5 (ArH^OAS^); 54.9 (O*C*H_3_); 29.6 (*C*H^Dipp^); 25.9 (*C*H_3_^Dipp^); 22.6 (*C*H_3_^Dipp^). ^1^H NMR (THF-d_8_, 500 MHz, 295 K) δ: 12.20 (s, 2H, N*H*^Dipp^); 7.37 (s, 1H, N*H*^OAS^); 7.15 (t, ^3^*J* = 7.5 Hz, 2H, Ar*H*^Dipp^); 7.07 (d, ^3^*J* = 7.7 Hz, 4H, Ar*H*^Dipp^); 6.90 (t, ^3^*J* = 7.9 Hz, 1H, Ar*H*^OAS^); 6.68 (d, ^3^*J* = 8.3 Hz, 1H, Ar*H*^OAS^); 6.62 (d, ^3^*J* = 7.8 Hz, 1H, Ar*H*^OAS^); 6.40 (t, ^3^*J* = 7.7 Hz, 1H, Ar*H*^OAS^); 3.73 (s, 3H, OC*H*_3_); 3.47 (m, ^3^*J* = 6.8 Hz, 4H, C*H*^Dipp^); 1.35 (d, ^3^*J* = 7.0 Hz, 12H, C*H*_3_^Dipp^); 1.33 (d, ^3^*J* = 6.8 Hz, 12H, C*H*_3_^Dipp^). ^13^C NMR (THF-d_8_, 125 MHz, 295 K) δ: 157.0 (Ar_q_^Gua^); 155.4 (Ar_q_^OAS^-OCH_3_); 148.0 (Ar_q_^Dipp^); 131.6 (Ar_q_^Dipp^); 129.9 (ArH^OAS^); 129.6 (ArH^Dipp^ + ArH^OAS^); 124.6 (Ar_q_^OAS^-NH); 124.2 (ArH^Dipp^); 120.7 (ArH^OAS^); 111.6 (ArH^OAS^); 55.8 (O*C*H_3_); 29.8 (*C*H^Dipp^); 26.1 (*C*H_3_^Dipp^); 22.6 (*C*H_3_^Dipp^). ^1^H NMR (DMSO-d_6_, 500 MHz, 295 K) δ: 8.79 (s, 1H, N*H*^Dipp^); 8.69 (s, 1H, N*H*^Dipp^); 8.59 (s, 1H, N*H*^OAS^); 7.47 – 7.36 (br m, 3H, Ar*H*^Dipp^ + Ar*H*^OAS^); 7.30 (d, ^3^*J* = 7.7 Hz, 2H, Ar*H*^Dipp^); 7.27 (d, ^3^*J* = 7.7 Hz, 4H, Ar*H*^Dipp^); 7.21 (d, ^3^*J* = 8.2 Hz, 1H, Ar*H*^OAS^); 7.10 (d, ^3^*J* = 6.9 Hz, 1H, Ar*H*^OAS^); 7.04 (t, ^3^*J* = 7.5 Hz, 1H, Ar*H*^OAS^); 3.88 (s, 3H, OC*H*_3_); 3.26 (m, ^3^*J* = 6.8 Hz, 2H, C*H*^Dipp^); 3.20 (m, ^3^*J* = 6.8 Hz, 2H, C*H*^Dipp^); 1.38 (d, ^3^*J* = 6.8 Hz, 6H, C*H*_3_^Dipp^); 1.34 (d, ^3^*J* = 6.8 Hz, 6H, C*H*_3_^Dipp^); 1.15 (d, ^3^*J* = 6.3 Hz, 6H, C*H*_3_^Dipp^); 1.14 (d, ^3^*J* = 6.3 Hz, 6H, C*H*_3_^Dipp^). ^13^C NMR (DMSO-d_6_, 125 MHz, 295 K) δ: 155.8 (Ar_q_^OAS^-OCH_3_); 153.5 (Ar_q_^Gua^); 147.4 (Ar_q_^Dipp^); 146.9 (Ar_q_^Dipp^); 130.3 (ArH^OAS^); 129.8 (ArH^OAS^); 129.6 (Ar_q_^Dipp^); 129.4 (Ar_q_^Dipp^); 128.5 (Ar_q_^Dipp^); 128.4 (Ar_q_^Dipp^); 124.2 (ArH^Dipp^); 123.9 (ArH^Dipp^); 122.2 (Ar_q_^OAS^-NH); 120.8 (ArH^OAS^); 112.4 (ArH^OAS^); 55.6 (O*C*H_3_); 28.1 (*C*H^Dipp^); 27.8 (*C*H^Dipp^); 24.7 (*C*H_3_^Dipp^); 24.1 (*C*H_3_^Dipp^); 23.4 (*C*H_3_^Dipp^); 22.5 (*C*H_3_^Dipp^).

MALDI: [M+H]^+^ Calcd. for C_32_H_44_ClN_3_O 522.32512; found 486.34804. The result corresponds to the basic form without HCl, which was probably cleaved during ionization. Calcd. for the basic form C_32_H_43_N_3_O is 486.34844.

**Preparation of 22**

**Method B:** *N,N'*‑bis(2,6‑diisopropylphenyl)carbodiimide (1.979 g, 5.46 mmol); 3-methoxyaniline (0.61 ml, ρ = 1.096 g.cm^-3^, 5.46 mmol); HCl (0.49 ml of 35% aqueous solution, ρ = 1.180 g.cm^-3^, 5.46 mmol); Et_3_N (0.84 ml, ρ = 0.727 g.cm^-3^, 6.01 mmol); recrystallized from hexane (2x *ca* 50 ml) to give 1.905 g (72%) of off-white crystalline **22**. Single crystals suitable for sc-XRD analysis were obtained by slow evaporation of hexane from saturated solution of **22** at room temperature. Mp 140 - 141 °C. Analysis, calculated: C 79.13%, H 8.92%, N 8.65%; found: C 78.78±0.15, H 8.76±0.01, N 8.32±0.01.

**Method C:** 3-methoxyaniline (0.16 ml, ρ = 1.096 g.cm^-3^, 1.38 mmol). Sub-stoichiometric amounts of 0.1 and 0.5 equivalent of HCl solution were tested in a catalytic guanylation approach to monitor activity in time scales. Details of NMR yields, guanylation progress and reaction time are given in the discussion part of the main text and Figure S10.

NMR spectra of **22** show the presence of two tautomers at THF-d_8_ and C_6_D_6_. Signals of the second(minor) tautomer of **22** at C_6_D_6_ and THF-d_8_ were not assigned (and marked with black dots on NMR spectra – see Figures S48-S51). **NMR spectra @C_6_D_6_ at 295 K for major tautomer** - ^1^H NMR (C_6_D_6_, 500 MHz, 295 K) δ: 8.23 (s, 1H, Ar*H*^MAS^); 7.30 (d, ^3^*J* = 7.5 Hz, 2H, Ar*H*^Dipp^); 7.19 (t, ^3^*J* = 7.5 Hz, 1H, Ar*H*^Dipp^); 7.12 (t, ^3^*J* = 7.6 Hz, 1H, Ar*H*^Dipp^); 7.00 (d, ^3^*J* = 7.5 Hz, 2H, Ar*H*^Dipp^); 6.91 (t, ^3^*J* = 8.1 Hz, 1H, Ar*H*^MAS^); 6.52 (d, ^3^*J* = 7.1 Hz, 1H, Ar*H*^MAS^); 6.48 (d, ^3^*J* = 7.8 Hz, 1H, Ar*H*^MAS^); 5.70 (s, 1H, N*H*^MAS^); 5.00 (s, 1H, N*H*^Dipp^); 3.57 (m, ^3^*J* = 6.7 Hz, 2H, C*H*^Dipp^); 3.39 (s, 3H, OC*H*_3_); 3.30 (m, ^3^*J* = 6.6 Hz, 2H, C*H*^Dipp^); 1.40 (d, ^3^*J* = 6.7 Hz, 6H, C*H*_3_^Dipp^); 1.35 (d, ^3^*J* = 6.5 Hz, 6H, C*H*_3_^Dipp^); 1.16 (d, ^3^*J* = 5.9 Hz, 6H, C*H*_3_^Dipp^); 0.98 (d, ^3^*J* = 5.9 Hz, 6H, C*H*_3_^Dipp^). ^13^C NMR (C_6_D_6_, 125 MHz, 295 K) δ: 161.4 (Ar_q_^MAS^-OCH_3_); 148.7 (Ar_q_^Dipp^); 144.2 (Ar_q_^Dipp^); 143.7 (Ar_q_^Gua^); 142.3 (Ar_q_^MAS^-NH); 141.1 (Ar_q_^Dipp^); 131.9 (Ar_q_^Dipp^); 129.9 (ArH^MAS^); 129.77 (ArH^Dipp^); 124.8 (ArH^Dipp^); 124.0 (ArH^Dipp^); 123.9 (ArH^Dipp^); 111.0 (ArH^MAS^); 108.7 (ArH^MAS^); 105.1 (ArH^MAS^); 55.2 (O*C*H_3_); 29.3 (*C*H^Dipp^); 28.8 (*C*H^Dipp^); 25.4 (*C*H_3_^Dipp^); 24.4 (2x *C*H_3_^Dipp^); 22.8 (*C*H_3_^Dipp^). **NMR spectra @THF-d_8_ at 295 K for major tautomer** - ^1^H NMR (THF-d_8_, 500 MHz, 295 K) δ: 7.84 (s, 1H, Ar*H*^MAS^); 7.33 (t, ^3^*J* = 7.2 Hz, 1H, Ar*H*^Dipp^); 7.24 (d, ^3^*J* = 7.3 Hz, 2H, Ar*H*^Dipp^); 7.11 (d, ^3^*J* = 7.3 Hz, 2H, Ar*H*^Dipp^); 6.99 – 6.90 (m, 2H, Ar*H*^Dipp^ ^+^ Ar*H*^MAS^); 6.70 (d, ^3^*J* = 7.5 Hz, 1H, Ar*H*^MAS^); 6.41 (d, ^3^*J* = 7.4 Hz, 1H, Ar*H*^MAS^); 5.97 (s, 1H, N*H*^Dipp^); 5.73 (s, 1H, N*H*^MAS^); 3.67 (s, 3H, OC*H*_3_); 3.46 – 3.34 (br m, 4H, C*H*^Dipp^); 1.34 (d, ^3^*J* = 6.3 Hz, 6H, C*H*_3_^Dipp^); 1.24 (d, ^3^*J* = 5.9 Hz, 6H, C*H*_3_^Dipp^); 1.17 (d, ^3^*J* = 6.4 Hz, 6H, C*H*_3_^Dipp^); 1.12 (d, ^3^*J* = 5.8 Hz, 6H, C*H*_3_^Dipp^). ^13^C NMR (THF-d_8_, 125 MHz, 295 K) δ: 161.4 (Ar_q_^MAS^-OCH_3_); 149.4 (Ar_q_^Dipp^); 144.9 (Ar_q_^Dipp^); 144.3 (Ar_q_^Gua^); 143.1 (Ar_q_^MAS^-NH); 141.1 (Ar_q_^Dipp^); 133.3 (Ar_q_^Dipp^); 129.7 (ArH^Dipp^); 129.6 (ArH^MAS^); 125.0 (ArH^Dipp^); 123.6 (ArH^Dipp^); 123.2 (ArH^Dipp^); 111.4 (ArH^MAS^); 108.2 (ArH^MAS^); 105.2 (ArH^MAS^); 55.4 (O*C*H_3_); 29.4 (*C*H^Dipp^); 29.2 (*C*H^Dipp^); 25.3 (*C*H_3_^Dipp^); 24.4 (2x *C*H_3_^Dipp^); 22.9 (*C*H_3_^Dipp^).

MALDI: [M+H]^+^ Calcd. for C_32_H_43_N_3_O 486.34844; found 486.34789.

**Preparation of 23**

**Method B:** *N,N'*‑bis(2,6‑diisopropylphenyl)carbodiimide (1.395 g, 3.85 mmol); 4-methoxyaniline (0.474 g, 3.85 mmol); HCl (0.34 ml of 35% aqueous solution, ρ = 1.180 g.cm^-3^, 3.85 mmol). Corresponding crude guanidinium chloride **23^.^**HCl was filtered off and recrystallized from Et_2_O (*ca* 100 ml) to give 1.808 g (90%) of pure off-white powder of **23**^.^HCl (properties and NMR parameters are described below).

A portion of **23**^.^HCl (0.872 g, 1.67 mmol) was transformed to crude **23** using Et_3_N (0.26 ml, ρ = 0.727 g.cm^-3^, 1.84 mmol) in hexane (30 ml) for 30 minutes and recrystallized from hexane (*ca* 25 ml). Yield 0.589 g (73%) of off-white crystalline **23**. Single crystals suitable for sc-XRD analysis were obtained by slow evaporation of hexane from saturated solution of **23** at room temperature. Mp 134.5 - 136 °C. Analysis, calculated: C 79.13%, H 8.92%, N 8.65%; found: C 79.04±0.23, H 9.16±0.07, N 8.48±0.07.

**Method C:** 4-methoxyaniline (0.170 g, 1.38 mmol). Sub-stoichiometric amounts of 0.5 equivalent of HCl solution were tested in a catalytic guanylation approach to monitor activity in time scales. Details of NMR yields, guanylation progress and reaction time are concluded in the discussion part of the main text and Figure S11 left.

NMR spectra of **23** show the presence of two tautomers at THF-d_8_ and C_6_D_6_. Signals of the second (minor) tautomer of **23** at C_6_D_6_ and THF-d_8_ were not assigned (and marked with black dots on NMR spectra – see Figures S52-S55). **NMR spectra @C_6_D_6_ at 295 K for major tautomer** - ^1^H NMR (C_6_D_6_, 500 MHz, 295 K) δ: 7.45 (d, ^3^*J* = 8.6 Hz, 2H, Ar*H*^PAS^); 7.31 (d, ^3^*J* = 7.5 Hz, 2H, Ar*H*^Dipp^); 7.19 (t, ^3^*J* = 7.5 Hz, 1H, Ar*H*^Dipp^); 7.13 (t, ^3^*J* = 7.8 Hz, 1H, Ar*H*^Dipp^); 7.02 (d, ^3^*J* = 7.6 Hz, 2H, Ar*H*^Dipp^); 6.71 (d, ^3^*J* = 8.6 Hz, 2H, Ar*H*^PAS^); 5.45 (s, 1H, N*H*^PAS^); 4.99 (s, 1H, N*H*^Dipp^); 3.59 (m, ^3^*J* = 6.8 Hz, 2H, C*H*^Dipp^); 3.34 (m, ^3^*J* = 6.7 Hz, 2H, C*H*^Dipp^); 3.26 (s, 3H, OC*H*_3_); 1.42 (d, ^3^*J* = 6.8 Hz, 6H, C*H*_3_^Dipp^); 1.37 (d, ^3^*J* = 6.5 Hz, 6H, C*H*_3_^Dipp^); 1.20 (d, ^3^*J* = 6.4 Hz, 6H, C*H*_3_^Dipp^); 1.00 (d, ^3^*J* = 6.5 Hz, 6H, C*H*_3_^Dipp^). ^13^C NMR (C_6_D_6_, 125 MHz, 295 K) δ: 155.9 (Ar_q_^PAS^-OCH_3_); 148.7 (Ar_q_^Dipp^); 144.6 (Ar_q_^Dipp^); 144.2 (Ar_q_^Gua^); 141.2 (Ar_q_^Dipp^); 134.2 (Ar_q_^PAS^-NH); 132.2 (Ar_q_^Dipp^); 129.6 (ArH^Dipp^); 124.7 (ArH^Dipp^); 124.0 (ArH^Dipp^); 123.8 (ArH^Dipp^); 121.3 (ArH^PAS^); 114.6 (ArH^PAS^); 55.3 (O*C*H_3_); 29.2 (*C*H^Dipp^); 28.8 (*C*H^Dipp^); 25.3 (*C*H_3_^Dipp^); 24.4 (2x *C*H_3_^Dipp^); 23.0 (*C*H_3_^Dipp^). **NMR spectra @THF-d_8_ at 295 K for major tautomer** - ^1^H NMR (THF-d_8_, 500 MHz, 295 K) δ: 7.47 (d, ^3^*J* = 8.3 Hz, 2H, Ar*H*^PAS^); 7.31 (t, ^3^*J* = 7.4 Hz, 1H, Ar*H*^Dipp^); 7.23 (d, ^3^*J* = 7.4 Hz, 2H, Ar*H*^Dipp^); 7.08 (d, ^3^*J* = 7.4 Hz, 2H, Ar*H*^Dipp^); 6.90 (t, ^3^*J* = 7.1 Hz, 1H, Ar*H*^Dipp^); 6.71 (d, ^3^*J* = 8.3 Hz, 2H, Ar*H*^PAS^); 5.89 (s, 1H, N*H*^Dipp^); 5.61 (s, 1H, N*H*^PAS^); 3.68 (s, 3H, OC*H*_3_); 3.43 (m, ^3^*J* = 6.7 Hz, 2H, C*H*^Dipp^); 3.38 (m, ^3^*J* = 6.4 Hz, 2H, C*H*^Dipp^); 1.33 (d, ^3^*J* = 6.6 Hz, 6H, C*H*_3_^Dipp^); 1.26 (d, ^3^*J* = 6.2 Hz, 6H, C*H*_3_^Dipp^); 1.17-1.09 (m, 2x6H, C*H*_3_^Dipp^). ^13^C NMR (THF-d_8_, 125 MHz, 295 K) δ: 156.1 (Ar_q_^PAS^-OCH_3_); 149.4 (Ar_q_^Dipp^); 145.4 (Ar_q_^Dipp^); 144.8 (Ar_q_^Gua^); 141.2 (Ar_q_^Dipp^); 135.1 (Ar_q_^PAS^-NH); 133.6 (Ar_q_^Dipp^); 129.5 (ArH^Dipp^); 124.8 (ArH^Dipp^); 123.5 (ArH^Dipp^); 122.9 (ArH^Dipp^); 122.0 (ArH^PAS^); 114.3 (ArH^PAS^); 55.6 (O*C*H_3_); 29.3 (*C*H^Dipp^); 29.2 (*C*H^Dipp^); 25.1 (*C*H_3_^Dipp^); 24.5 (*C*H_3_^Dipp^); 24.4 (*C*H_3_^Dipp^); 23.0 (*C*H_3_^Dipp^).

MALDI: [M+H]^+^ Calcd. for C_32_H_43_N_3_O 486.34844; found 486.34778.

**Properties of 23**^.^HCl

Preparation is associated with paragraph **Preparation of 23 Method B** (described above). Single crystals suitable for sc-XRD analysis were obtained by slow evaporation of Et_2_O from saturated solution of **23**^.^HCl at room temperature. Mp 256 - 258 °C. Analysis, calculated: C 73.61%, H 8.49%, N 8.05%, Cl 6,79%; found: C 74.15±0.18, H 8.46±0.08, N 7.75±0.14, Cl 6.94±0.25.

^1^H NMR (C_6_D_6_, 500 MHz, 295 K) δ: 13.05 (s, 2H, N*H*^Dipp^); 7.00 (t, ^3^*J* = 7.5 Hz, 2H, Ar*H*^Dipp^); 6.88 (d, ^3^*J* = 7.7 Hz, 4H, Ar*H*^Dipp^); 6.30 (d, ^3^*J* = 8.7 Hz, 2H, Ar*H*^PAS^); 6.15 (d, ^3^*J* = 8.8 Hz, 2H, Ar*H*^PAS^); 5.54 (s, 1H, N*H*^PAS^); 3.46 (m, ^3^*J* = 6.8 Hz, 4H, C*H*^Dipp^); 3.05 (s, 3H, OC*H*_3_); 1.40 (d, ^3^*J* = 6.8 Hz, 12H, C*H*_3_^Dipp^); 1.19 (d, ^3^*J* = 6.9 Hz, 12H, C*H*_3_^Dipp^). ^13^C NMR (C_6_D_6_, 125 MHz, 295 K) δ: 159.4 (Ar_q_^PAS^-OCH_3_); 156.4 (Ar_q_^Gua^); 147.4 (Ar_q_^Dipp^); 130.5 (Ar_q_^Dipp^); 129.8 (ArH^Dipp^); 129.6 (ArH^PAS^); 126.6 (Ar_q_^PAS^-NH); 124.4 (ArH^Dipp^); 114.0 (ArH^PAS^); 55.3 (O*C*H_3_); 29.8 (*C*H^Dipp^); 25.8 (*C*H_3_^Dipp^); 22.7 (*C*H_3_^Dipp^). ^1^H NMR (THF-d_8_, 500 MHz, 295 K) δ: 12.18 (s, 2H, N*H*^Dipp^); 7.85 (s, 1H, N*H*^PAS^); 7.17 (br s, 2H, Ar*H*^Dipp^); 7.09 (br s, 4H, Ar*H*^Dipp^); 6.65 (d, ^3^*J* = 8.8 Hz, 2H, Ar*H*^PAS^); 6.47 (d, ^3^*J* = 8.9 Hz, 2H, Ar*H*^PAS^); 3.59 (s, 3H, OC*H*_3_); 3.37 (m, ^3^*J* = 6.3 Hz, 4H, C*H*^Dipp^); 1.35 (d, ^3^*J* = 7.2 Hz, 12H, C*H*_3_^Dipp^); 1.33 (d, ^3^*J* = 7.2 Hz, 12H, C*H*_3_^Dipp^). ^13^C NMR (THF-d_8_, 125 MHz, 295 K) δ: 159.7 (Ar_q_^PAS^-OCH_3_); 157.2 (Ar_q_^Gua^); 147.6 (Ar_q_^Dipp^); 131.5 (Ar_q_^Dipp^); 129.7 (ArH^Dipp^); 129.1 (ArH^PAS^); 128.5 (Ar_q_^PAS^-NH); 124.4 (ArH^Dipp^); 114.5 (ArH^PAS^); 55.7 (O*C*H_3_); 30.1 (*C*H^Dipp^); 25.9 (*C*H_3_^Dipp^); 22.7 (*C*H_3_^Dipp^).

MALDI: [M+H]^+^ Calcd. for C_32_H_44_ClN_3_O 522.32512; found 486.34791. The result corresponds to the basic form without HCl, which was probably cleaved during ionization. Calcd. for the basic form C_32_H_43_N_3_O is 486.34844.

**Preparation of 24**

**Method B:** *N,N'*‑bis(2,6‑diisopropylphenyl)carbodiimide (1.000 g, 2.76 mmol); 4-fluoroaniline (0.26 ml, ρ = 1.173 g.cm^-3^, 2.76 mmol); HCl (0.25 ml of 35% aqueous solution, ρ = 1.180 g.cm^-3^, 2.76 mmol); Et_3_N (0.42 ml, ρ = 0.727 g.cm^-3^, 3.03 mmol); recrystallized from hexane (*ca* 25 ml) to give 1.004 g (77%) of white crystalline **24**. Single crystals suitable for sc-XRD analysis were obtained by slow evaporation of hexane from saturated solution of **24** at room temperature. Mp 147 - 149 °C. Analysis, calculated: C 78.61%, H 8.51%, N 8.87%; found: C 79.26±0.13, H 8.41±0.08, N 8.88±0.04.

**Method C:** 4-fluoroaniline (0.13 ml, 1.38 mmol, ρ = 1.180 g.cm^-3^). Sub-stoichiometric amounts of 0.5 equivalent of HCl solution were tested in a catalytic guanylation approach to monitor cationic H-initiator activity in time scales. Details of NMR yields, guanylation progress and reaction time are given in the discussion part of the main text and Figure S11 right.

NMR spectra of **24** show the presence of two tautomers at THF-d_8_ and C_6_D_6_. Signals of the second(minor) tautomer of **24** at C_6_D_6_ and THF-d_8_ were not assigned (and marked with black dots on NMR spectra – see Figures S56-S59). **NMR spectra @C_6_D_6_ at 295 K for major tautomer** - ^1^H NMR (C_6_D_6_, 500 MHz, 295 K) δ: 7.34 – 7.29 (m, 2H, Ar*H*^FAN^); 7.29 (d, ^3^*J* = 7.6 Hz, 2H, Ar*H*^Dipp^); 7.19 (t, ^3^*J* = 7.8 Hz, 1H, Ar*H*^Dipp^); 7.14 (t, ^3^*J* = 7.7 Hz, 1H, Ar*H*^Dipp^); 7.01 (d, ^3^*J* = 7.7 Hz, 2H, Ar*H*^Dipp^); 6.72 (t, ^3^*J* = 8.6 Hz, 2H, Ar*H*^FAN^); 5.45 (s, 1H, N*H*^FAN^); 4.96 (s, 1H, N*H*^Dipp^); 3.51 (m, ^3^*J* = 6.8 Hz, 2H, C*H*^Dipp^); 3.27 (m, ^3^*J* = 6.8 Hz, 2H, C*H*^Dipp^); 1.40 (d, ^3^*J* = 7.0 Hz, 6H, C*H*_3_^Dipp^); 1.32 (d, ^3^*J* = 6.7 Hz, 6H, C*H*_3_^Dipp^); 1.15 (d, ^3^*J* = 6.7 Hz, 6H, C*H*_3_^Dipp^); 0.98 (d, ^3^*J* = 6.7 Hz, 6H, C*H*_3_^Dipp^). ^13^C NMR (C_6_D_6_, 125 MHz, 295 K) δ: 158.9 (d, ^1^*J*_C,F_ = 240.7 Hz, Ar_q_^FAN^-F); 148.7 (Ar_q_^Dipp^); 144.1 (Ar_q_^Dipp^); 143.8 (Ar_q_^Gua^); 141.1 (Ar_q_^Dipp^); 136.9 (d, ^4^*J*_C,F_ = 2.1 Hz, Ar_q_^FAN^-NH); 131.8 (Ar_q_^Dipp^); 129.7 (ArH^Dipp^); 124.7 (ArH^Dipp^); 124.0 (2x ArH^Dipp^); 120.8 (d, ^3^*J*_C,F_ = 7.3 Hz, ArH^FAN^); 115.8 (d, ^2^*J*_C,F_ = 22.3 Hz, ArH^FAN^); 29.2 (*C*H^Dipp^); 28.8 (*C*H^Dipp^); 25.3 (*C*H_3_^Dipp^); 24.4 (*C*H_3_^Dipp^); 24.3 (*C*H_3_^Dipp^); 22.9 (*C*H_3_^Dipp^). **NMR spectra @THF-d_8_ at 295 K for major tautomer** - ^1^H NMR (THF-d_8_, 500 MHz, 295 K) δ: 7.67 – 7.56 (m, 2H, Ar*H*^FAN^); 7.32 (t, ^3^*J* = 7.4 Hz, 1H, Ar*H*^Dipp^); 7.24 (d, ^3^*J* = 7.5 Hz, 2H, Ar*H*^Dipp^); 7.11 (d, ^3^*J* = 7.5 Hz, 2H, Ar*H*^Dipp^); 6.93 (t, ^3^*J* = 7.6 Hz, 1H, Ar*H*^Dipp^); 6.89 (d, ^3^*J* = 8.4 Hz, 2H, Ar*H*^FAN^); 5.99 (s, 1H, N*H*^Dipp^); 5.79 (s, 1H, N*H*^FAN^); 3.42 (m, ^3^*J* = 6.7 Hz, 2H, C*H*^Dipp^); 3.36 (m, ^3^*J* = 6.8 Hz, 2H, C*H*^Dipp^); 1.34 (d, ^3^*J* = 6.7 Hz, 6H, C*H*_3_^Dipp^); 1.26 (d, ^3^*J* = 6.4 Hz, 6H, C*H*_3_^Dipp^); 1.18-1.10 (m, 2x6H, C*H*_3_^Dipp^). ^13^C NMR (THF-d_8_, 125 MHz, 295 K) δ: 159.1 (d, ^1^*J*_C,F_ = 239.5 Hz, Ar_q_^FAN^-F); 149.3 (Ar_q_^Dipp^); 145.0 (Ar_q_^Dipp^); 144.6 (Ar_q_^Gua^); 141.2 (Ar_q_^Dipp^); 138.2 (Ar_q_^FAN^-NH); 133.4 (Ar_q_^Dipp^); 129.7 (ArH^Dipp^); 124.9 (ArH^Dipp^); 123.6 (ArH^Dipp^); 123.2 (ArH^Dipp^); 121.8 (d, ^3^*J*_C,F_ = 6.8 Hz, ArH^FAN^); 115.4 (d, ^2^*J*_C,F_ = 22.1 Hz, ArH^FAN^); 29.4 (*C*H^Dipp^); 29.2 (*C*H^Dipp^); 25.1 (*C*H_3_^Dipp^); 24.4 (*C*H_3_^Dipp^); 24.4 (*C*H_3_^Dipp^); 23.0 (*C*H_3_^Dipp^).

MALDI: [M+H]^+^ Calcd. for C_31_H_40_FN_3_ 474.32845; found 474.32895.

**Preparation of 25**

**Method B:** To prevent oxidation of NCN moiety of starting *N,N'*‑bis(2,6‑diisopropylphenyl)carbodiimide to corresponding urea, handling and synthetic procedure were carried out under inert argon atmosphere. *N,N'*‑bis(2,6‑diisopropylphenyl)carbodiimide (1.000 g, 2.76 mmol); 2-nitroaniline (0.381 g, 2.76 mmol); HCl (0.24 ml of 35% aqueous solution, ρ = 1.180 g.cm^-3^, 2.76 mmol); Et_3_N (0.42 ml, ρ = 0.727 g.cm^-3^, 3.03 mmol); washed by Et_2_O (80 ml); suspension in hexane (30 ml). Crystallized from hexane (*ca* 10 ml) to give 0.290 g (21%) of yellow crystalline **25**. Single crystals suitable for sc-XRD analysis were obtained by cooling of saturated solution of **25** in Et_2_O to +7 °C. Mp 196.5 – 197.5 °C. Analysis, calculated: C 74.37%, H 8.05%, N 11.19%; found: C 74.37±0.05, H 8.33±0.08, N 10.96±0.01.

NMR spectra of **25** show the presence of two tautomers at THF-d_8_. Signals of the second(minor) tautomer of **25** at THF-d_8_ were not assigned (and marked with black dots on NMR spectra – see Figures S60-S61). **NMR spectra @C_6_D_6_ at 295 K** - ^1^H NMR (C_6_D_6_, 500 MHz, 295 K) δ: 9.82 (d, ^3^*J* = 8.7 Hz, 1H, Ar*H*^ONO^); 9.77 (s, 1H, N*H*^ONO^); 7.80 (dd, ^3^*J* = 8.4 Hz, ^4^*J* = 1.6 Hz, 1H, Ar*H*^ONO^); 7.30 (d, ^3^*J* = 7.7 Hz, 2H, Ar*H*^Dipp^); 7.24 (t, ^3^*J* = 7.8 Hz, 1H, Ar*H*^Dipp^); 7.19 (t, ^3^*J* = 7.4 Hz, 1H, Ar*H*^Dipp^); 7.09 (d, ^3^*J* = 7.8 Hz, 2H, Ar*H*^Dipp^); 7.07 (td as m, ^3^*J* = 7.9 Hz, ^4^*J* = 1.5 Hz, 1H, Ar*H*^ONO^); 6.27 (td as m, ^3^*J* = 7.8 Hz, ^4^*J* = 1.3 Hz, 1H, Ar*H*^ONO^); 5.12 (s, 1H, N*H*^Dipp^); 3.51 (m, ^3^*J* = 6.9 Hz, 2H, C*H*^Dipp^); 3.26 (m, ^3^*J* = 6.9 Hz, 2H, C*H*^Dipp^); 1.38 (d, ^3^*J* = 6.9 Hz, 6H, C*H*_3_^Dipp^); 1.35 (d, ^3^*J* = 6.9 Hz, 6H, C*H*_3_^Dipp^); 1.14 (d, ^3^*J* = 6.9 Hz, 6H, C*H*_3_^Dipp^); 0.98 (d, ^3^*J* = 6.9 Hz, 6H, C*H*_3_^Dipp^). **NMR spectra @THF-d_8_ at 295 K for major tautomer** - ^1^H NMR (THF-d_8_, 500 MHz, 295 K) δ: 9.63 (d, ^3^*J* = 8.7 Hz, 1H, Ar*H*^ONO^); 9.45 (s, 1H, N*H*^ONO^); 8.09 (dd, ^3^*J* = 8.5 Hz, ^4^*J* = 1.2 Hz, 1H, Ar*H*^ONO^); 7.57 (t, ^3^*J* = 8.1 Hz, 1H, Ar*H*^ONO^); 7.36 (t, ^3^*J* = 7.7 Hz, 1H, Ar*H*^Dipp^); 7.25 (d, ^3^*J* = 7.7 Hz, 2H, Ar*H*^Dipp^); 7.15 (d, ^3^*J* = 7.7 Hz, 2H, Ar*H*^Dipp^); 6.99 (t, ^3^*J* = 7.6 Hz, 1H, Ar*H*^Dipp^); 6.96 (t, ^3^*J* = 7.9 Hz, 1H, Ar*H*^ONO^); 6.37 (s, 1H, N*H*^Dipp^); 3.41 – 3.29 (m, 4H, 2x C*H*^Dipp^); 1.36 (d, ^3^*J* = 7.0 Hz, 6H, C*H*_3_^Dipp^); 1.20 – 1.14 (m, 12H, C*H*_3_^Dipp^); 1.13 (d, ^3^*J* = 6.9 Hz, 6H, C*H*_3_^Dipp^). ^13^C NMR (THF-d_8_, 125 MHz, 295 K) δ: 149.1 (Ar_q_^Dipp^); 144.8 (Ar_q_^Gua^); 144.1 (Ar_q_^Dipp^); 140.7 (Ar_q_^Dipp^); 139.2 (Ar_q_^ONO^-NH); 136.4 (ArH^ONO^); 135.8 (Ar_q_^ONO^-N0_2_); 132.7 (Ar_q_^Dipp^); 130.2 (ArH^Dipp^); 126.5 (ArH^ONO^); 125.1 (ArH^Dipp^); 123.8 (2x ArH^Dipp^); 121.8 (ArH^ONO^); 120.8 (ArH^ONO^); 29.5 (*C*H^Dipp^); 29.3 (*C*H^Dipp^); 25.2 (*C*H_3_^Dipp^); 24.6 (*C*H_3_^Dipp^); 24.1 (*C*H_3_^Dipp^); 22.9 (*C*H_3_^Dipp^).

MALDI: [M+H]^+^ Calcd. for C_31_H_40_N_4_O_2_ 501.32295; found 501.32454.

**Preparation of 26**

**Method B:** *N,N'*‑bis(2,6‑diisopropylphenyl)carbodiimide (1.347 g, 3.72 mmol); 3-nitroaniline (0.513 g, 3.72 mmol); HCl (0.33 ml of 35% aqueous solution, ρ = 1.180 g.cm^-3^, 3.72 mmol); Et_3_N (0.57 ml, ρ = 0.727 g.cm^-3^, 4.09 mmol); recrystallized from hexane (*ca* 40 ml) to give 1.509 g (81%) of pale yellow crystalline **26**. Single crystals suitable for sc-XRD analysis were obtained by slow evaporation of hexane from saturated solution of **26** at room temperature. Mp 166 - 167 °C. Analysis, calculated: C 74.37%, H 8.05%, N 11.19%; found: C 74.59±0.06, H 7.99±0.15, N 11.07±0.03.

**Method C:** 4-nitroaniline (0.191 g, 1.38 mmol). Sub-stoichiometric amounts of 0.1 and 0.5 equivalent of HCl solution were tested in a catalytic guanylation approach to monitor cationic H-initiator activity in time scales. Details of NMR yields, guanylation progress and reaction time are given in the discussion part of the main text and Figure S12.

NMR spectra of **26** show the presence of two tautomers at THF-d_8_, C_6_D_6,_ and Tol-d_8_ (Figures S62-S66). For measurement @THF-d_8_ at 295 K (Figures S64-S65) as well as 333 K the major one is *ca* 82 mol % and the second minor *ca* 18 mol %, however at 280 K is molar ratio is about 81:19, and for 173 K about 80:20. Chemical shift values of **26** @THF-d_8_ at 333 K, 295 K, 280 K, and 173 K (Figures S5-S6) differ for major and minor tautomer in all VT ^1^H NMR spectra. **NMR spectra @THF-d_8_ at 295 K for major tautomer** - ^1^H NMR (THF-d_8_, 500 MHz, 295 K) δ: 8.85 (dd as t, ^4^*J* = 2.1 Hz, 1H, Ar*H*^MNO^); 8.00 (d, ^3^*J* = 8.2 Hz, 1H, Ar*H*^MNO^); 7.74 (d, ^3^*J* = 8.2 Hz, 1H, Ar*H*^MNO^); 7.37 (t, ^3^*J* = 8.2 Hz, 1H, Ar*H*^MNO^); 7.34 (t, ^3^*J* = 7.5 Hz, 1H, Ar*H*^Dipp^); 7.26 (d, ^3^*J* = 7.7 Hz, 2H, Ar*H*^Dipp^); 7.14 (d, ^3^*J* = 7.7 Hz, 2H, Ar*H*^Dipp^); 6.97 (t, ^3^*J* = 7.6 Hz, 1H, Ar*H*^Dipp^); 6.35 (s, 1H, N*H*^MNO^); 6.18 (s, 1H, N*H*^Dipp^); 3.40 (m, ^3^*J* = 6.9 Hz, 2H, C*H*^Dipp^); 3.36 (m, ^3^*J* = 6.9 Hz, 2H, C*H*^Dipp^); 1.36 (d, ^3^*J* = 7.0 Hz, 6H, C*H*_3_^Dipp^); 1.27 (d, ^3^*J* = 6.9 Hz, 6H, C*H*_3_^Dipp^); 1.18 (d, ^3^*J* = 6.7 Hz, 6H, C*H*_3_^Dipp^); 1.14 (d, ^3^*J* = 6.8 Hz, 6H, C*H*_3_^Dipp^). ^13^C NMR (THF-d_8_, 125 MHz, 295 K) δ: 149.7 (Ar_q_^MNO^-NO_2_); 149.2 (Ar_q_^Dipp^); 144.4 (Ar_q_^Gua^); 144.4 (Ar_q_^Dipp^); 143.3 (Ar_q_^MNO^-NH); 141.1 (Ar_q_^Dipp^); 133.0 (Ar_q_^Dipp^); 129.9 (ArH^Dipp^); 129.8 (ArH^MNO^); 125.7 (ArH^MNO^); 125.0 (ArH^Dipp^); 123.7 (ArH^Dipp^); 123.6 (ArH^Dipp^); 116.7 (ArH^MNO^); 114.7 (ArH^MNO^); 29.5 (*C*H^Dipp^); 29.3 (*C*H^Dipp^); 25.3 (*C*H_3_^Dipp^); 24.4 (*C*H_3_^Dipp^); 24.4 (*C*H_3_^Dipp^); 22.9 (*C*H_3_^Dipp^). ^15^N NMR (THF-d_8_, 51 MHz, 295 K) δ: -10 (*N*O_2_); -200 (C=*N*); -286 (MNO-*N*H); -299 (Dipp-*N*H). **NMR spectra @THF-d_8_ at 295 K for minor tautomer** - ^1^H NMR (THF-d_8_, 500 MHz, 295 K) δ: 7.77 (dd as t, ^4^*J* = 2.2 Hz, 0.22H, Ar*H*^MNO^); 7.64 (d, ^3^*J* = 8.1 Hz, 0.22H, Ar*H*^MNO^); 7.36 (m, 0.22H, Ar*H*^Dipp^); 7.34 (m, 0.22H, Ar*H*^MNO^); 7.28 (m, 0.44H, Ar*H*^Dipp^); 7.27 (m, 0.22H, Ar*H*^Dipp^); 7.21 (m, ^3^*J* = 7.8 Hz, 0.22H, Ar*H*^MNO^); 7.09 (m, 0.44H, Ar*H*^Dipp^); 6.41 (s, 0.22H, N*H*^MNO^); 5.89 (s, 0.22H, N*H*^Dipp^); 3.62 (m, 0.44H, C*H*^Dipp^); 3.38 (m, 0.44H, C*H*^Dipp^); 1.44 (d, 1.32H, C*H*_3_^Dipp^); 1.33 (d, 1.32H, C*H*_3_^Dipp^); 1.21 (d, 1.32H, C*H*_3_^Dipp^); 1.16 (d, 1.32H, C*H*_3_^Dipp^). ^13^C NMR (THF-d_8_, 125 MHz, 295 K) δ: 154.2 (Ar_q_^MNO^-NO_2_); 150.7 (Ar_q_^Gua^); 150.4 (Ar_q_^MNO^-NH); 149.7 (Ar_q_^Dipp^); 148.0 (Ar_q_^Dipp^); 134.9 (Ar_q_^Dipp^); 133.5 (Ar_q_^Dipp^); 130.6 (ArH^MNO^); 130.4 (ArH^MNO^); 129.8 (ArH^Dipp^); 128.1 (ArH^Dipp^); 124.7 (ArH^Dipp^); 123.7 (ArH^Dipp^); 117.9 (ArH^MNO^); 115.5 (ArH^MNO^); 29.7 (*C*H^Dipp^); 29.4 (*C*H^Dipp^); 25.4 (*C*H_3_^Dipp^); 25.1 (*C*H_3_^Dipp^); 24.2 (*C*H_3_^Dipp^); 23.3 (*C*H_3_^Dipp^). For ^15^N NMR @THF-d_8_ at 295 K, no signals of minor tautomer have been detected.

Chemical shift values and the molar ratio (91:9) of both tautomers of **26** @C_6_D_6_ at 333 K, 313 K, and 295 K - Figure S4) remain virtually the same for all VT ^1^H measurements. Signals of the second (minor) tautomer at C_6_D_6_ were not assigned (and marked with black dots on NMR spectra – see Figures S62-S63). **NMR spectra @C_6_D_6_ at 295 K for major tautomer** - ^1^H NMR (C_6_D_6_, 500 MHz, 295 K) δ: 9.01 (s, 1H, Ar*H*^MNO^); 7.49 (d, ^3^*J* = 7.7 Hz, 1H, Ar*H*^MNO^); 7.28 (d, ^3^*J* = 7.5 Hz, 2H, Ar*H*^Dipp^); 7.19 (t, ^3^*J* = 7.5 Hz, 1H, Ar*H*^Dipp^); 7.13 (t, ^3^*J* = 7.7 Hz, 1H, Ar*H*^Dipp^); 7.00 (d, ^3^*J* = 7.6 Hz, 2H, Ar*H*^Dipp^); 6.94 (d, ^3^*J* = 7.8 Hz, 1H, Ar*H*^MNO^); 6.61 (t, ^3^*J* = 8.1 Hz, 1H, Ar*H*^MNO^); 5.67 (s, 1H, N*H*^MNO^); 5.03 (s, 1H, N*H*^Dipp^); 3.46 (m, ^3^*J* = 6.8 Hz, 2H, C*H*^Dipp^); 3.22 (m, ^3^*J* = 6.7 Hz, 2H, C*H*^Dipp^); 1.37 (d, ^3^*J* = 7.0 Hz, 6H, C*H*_3_^Dipp^); 1.34 (d, ^3^*J* = 6.7 Hz, 6H, C*H*_3_^Dipp^); 1.14 (d, ^3^*J* = 6.4 Hz, 6H, C*H*_3_^Dipp^); 0.97 (d, ^3^*J* = 6.4 Hz, 6H, C*H*_3_^Dipp^). ^13^C NMR (C_6_D_6_, 125 MHz, 295 K) δ: 149.6 (Ar_q_^MNO^-NO_2_); 148.6 (Ar_q_^Dipp^); 143.4 (Ar_q_^Dipp^); 143.4 (Ar_q_^Gua^); 141.5 (Ar_q_^MNO^-NH); 140.9 (Ar_q_^Dipp^); 131.4 (Ar_q_^Dipp^); 129.9 (ArH^Dipp^); 129.5 (ArH^MNO^); 124.9 (ArH^Dipp^); 124.4 (ArH^Dipp^); 124.2 (ArH^MNO^); 124.1 (ArH^Dipp^); 117.1 (ArH^MNO^); 114.2 (ArH^MNO^); 29.4 (*C*H^Dipp^); 28.8 (*C*H^Dipp^); 25.4 (*C*H_3_^Dipp^); 24.4 (*C*H_3_^Dipp^); 24.2 (*C*H_3_^Dipp^); 22.8 (*C*H_3_^Dipp^).

For measurement @Tol-d_8_ at 373 K is a molar ratio about 85:15 major. With decreasing temperature, the amount of minor tautomer decreases – at 295 K is a ratio of 91:9, and at 183 K >99:<1. Chemical shift values of **26** @Tol-d_8_ at 373 K, 295 K, and 183 K (Figure S7 and Figure 5 in main text) differ for major and minor tautomer in all VT ^1^H NMR spectra. Signals of the second(minor) tautomer at Tol-d_8_ were not assigned (and marked with black dots on NMR spectra – see Figure S66). **NMR spectra @Tol-d_8_ at 295 K for major tautomer** - ^1^H NMR (Tol-d_8_, 500 MHz, 295 K) δ: 8.83 (dd as t, ^4^*J* = 2.1 Hz 1H, Ar*H*^MNO^); 7.4d (dd, ^3^*J* = 8.2 Hz, ^4^*J* = 1.5 Hz, 1H, Ar*H*^MNO^); 7.22 (d, ^3^*J* = 7.6 Hz, 2H, Ar*H*^Dipp^); 7.16 – 7.09 (m, 2H, 2x Ar*H*^Dipp^); 7.07 (d, ^3^*J* = 7.7 Hz, 1H, Ar*H*^MNO^); 6.98 (d, ^3^*J* = 7.7 Hz, 2H, Ar*H*^Dipp^); 6.63 (t, ^3^*J* = 8.2 Hz, 1H, Ar*H*^MNO^); 5.63 (s, 1H, N*H*^MNO^); 5.02 (s, 1H, N*H*^Dipp^); 3.41 (m, ^3^*J* = 6.8 Hz, 2H, C*H*^Dipp^); 3.21 (m, ^3^*J* = 6.8 Hz, 2H, C*H*^Dipp^); 1.35 (d, ^3^*J* = 7.0 Hz, 6H, C*H*_3_^Dipp^); 1.29 (d, ^3^*J* = 6.7 Hz, 6H, C*H*_3_^Dipp^); 1.14 (d, ^3^*J* = 6.7 Hz, 6H, C*H*_3_^Dipp^); 0.95 (d, ^3^*J* = 6.6 Hz, 6H, C*H*_3_^Dipp^).

MALDI: [M+H]^+^ Calcd. for C_31_H_40_N_4_O_2_ 501.32295; found 501.32413.

**Preparation of 27**

**Method B:** *N,N'*‑bis(2,6‑diisopropylphenyl)carbodiimide (1.000 g, 2.76 mmol); 4-nitroaniline (0.381 g, 2.76 mmol); HCl (0.25 ml of 35% aqueous solution, ρ = 1.180 g.cm^-3^, 2.76 mmol); Et_3_N (0.42 ml, ρ = 0.727 g.cm^-3^, 3.03 mmol); recrystallized from hexane (*ca* 40 ml) to give 0.874 g (63%) of pale yellow crystalline **27**. Single crystals suitable for sc-XRD analysis were obtained by slow evaporation of solvent(s) from saturated Et_2_O:hexane (1:1 ratio) solution of **27** at room temperature. Mp 185 – 185.5 °C. Analysis, calculated: C 74.37%, H 8.05%, N 11.19%; found: C 74.60±0.02, H 8.22±0.03, N 11.16±0.01.

**Method C:** 4-nitroaniline (0.191 g, 1.38 mmol). Sub-stoichiometric amounts of 0.5 equivalent of HCl solution were tested in a catalytic guanylation approach to monitor activity in time scales. Details of NMR yields, guanylation progress and reaction time are given in the discussion part of the main text and Figure S13.

NMR spectra of **27** show the presence of two tautomers at THF-d_8_ and C_6_D_6_. Signals of the second(minor) tautomer of **27** at C_6_D_6_ and THF-d_8_ were not assigned (and marked with black dots on NMR spectra – see Figures S67-S70). **NMR spectra @C_6_D_6_ at 295 K for major tautomer** - ^1^H NMR (C_6_D_6_, 500 MHz, 295 K) δ: 7.82 (d, ^3^*J* = 9.0 Hz, 2H, Ar*H*^PNO^); 7.28 (d, ^3^*J* = 7.6 Hz, 2H, Ar*H*^Dipp^); 7.21-7.16 (m, 3H, Ar*H*^Dipp^ + Ar*H*^PNO^); 7.13 (t, ^3^*J* = 7.7 Hz, 1H, Ar*H*^Dipp^); 6.99 (d, ^3^*J* = 7.7 Hz, 2H, Ar*H*^Dipp^); 5.74 (s, 1H, N*H*^PNO^); 5.00 (s, 1H, N*H*^Dipp^); 3.40 (m, ^3^*J* = 6.8 Hz, 2H, C*H*^Dipp^); 3.16 (m, ^3^*J* = 6.7 Hz, 2H, C*H*^Dipp^); 1.37 (d, ^3^*J* = 6.9 Hz, 6H, C*H*_3_^Dipp^); 1.31 (d, ^3^*J* = 6.7 Hz, 6H, C*H*_3_^Dipp^); 1.09 (d, ^3^*J* = 6.6 Hz, 6H, C*H*_3_^Dipp^); 0.96 (d, ^3^*J* = 6.6 Hz, 6H, C*H*_3_^Dipp^). ^13^C NMR (C_6_D_6_, 125 MHz, 295 K) δ: 148.6 (br s, Ar_q_^Dipp^); 146.0 (br s, Ar_q_^PNO^-NH); 143.0 (br s, Ar_q_^Gua^ + Ar_q_^Dipp^); 142.7 (Ar_q_^PNO^-N0_2_); 140.7 (br s, Ar_q_^Dipp^); 131.2 (br s, Ar_q_^Dipp^); 130.0 (br s, ArH^Dipp^); 125.5 (br s, ArH^PNO^); 124.9 (br s, ArH^Dipp^); 124.5 (br s, ArH^Dipp^); 124.2 (br s, ArH^Dipp^); 117.8 (br s, ArH^PNO^); 29.3 (*C*H^Dipp^); 28.9 (br s, *C*H^Dipp^); 25.3 (*C*H_3_^Dipp^); 24.4 (br s, *C*H_3_^Dipp^); 24.2 (br s, *C*H_3_^Dipp^); 22.8 (*C*H_3_^Dipp^). **NMR spectra @THF-d_8_ at 295 K for major tautomer** - ^1^H NMR (THF-d_8_, 500 MHz, 295 K) δ: 8.08 (d, ^3^*J* = 9.1 Hz, 2H, Ar*H*^PNO^); 7.92 (d, ^3^*J* = 9.0 Hz, 2H, Ar*H*^PNO^); 7.36 (t, ^3^*J* = 7.7 Hz, 1H, Ar*H*^Dipp^); 7.27 (d, ^3^*J* = 7.7 Hz, 2H, Ar*H*^Dipp^); 7.14 (d, ^3^*J* = 7.6 Hz, 2H, Ar*H*^Dipp^); 6.98 (t, ^3^*J* = 7.7 Hz, 1H, Ar*H*^Dipp^); 6.40 (s, 1H, N*H*^PNO^); 6.29 (s, 1H, N*H*^Dipp^); 3.42 – 3.27 (br m, 4H, 2x C*H*^Dipp^); 1.35 (d, ^3^*J* = 6.8 Hz, 6H, C*H*_3_^Dipp^); 1.23 (d, ^3^*J* = 6.8 Hz, 6H, C*H*_3_^Dipp^); 1.17 (d, ^3^*J* = 6.7 Hz, 6H, C*H*_3_^Dipp^); 1.13 (d, ^3^*J* = 6.7 Hz, 6H, C*H*_3_^Dipp^). ^13^C NMR (THF-d_8_, 125 MHz, 295 K) δ: 149.3 (Ar_q_^Dipp^); 147.9 (Ar_q_^PNO^-NH); 144.0 (Ar_q_^Dipp^); 144.0 (Ar_q_^Gua^); 142.6 (Ar_q_^PNO^-N0_2_); 140.9 (Ar_q_^Dipp^); 132.8 (Ar_q_^Dipp^); 130.0 (ArH^Dipp^); 125.4 (ArH^PNO^); 125.1 (ArH^Dipp^); 123.8 (2x ArH^Dipp^); 118.8 (ArH^PNO^); 29.5 (*C*H^Dipp^); 29.3 (*C*H^Dipp^); 25.2 (*C*H_3_^Dipp^); 24.4 (*C*H_3_^Dipp^); 24.3 (*C*H_3_^Dipp^); 22.9 (*C*H_3_^Dipp^).

MALDI: [M+H]^+^ Calcd. for C_31_H_40_N_4_O_2_ 501.32295; found 501.32461.

**Preparation of 28**

**Method B:** *N,N'*‑bis(2,6‑dimethylphenyl)carbodiimide (1.000 g, 3.99 mmol); 2-methoxyaniline (0.45 ml, ρ = 1.092 g.cm^-3^, 3.99 mmol); HCl (0.36 ml of 35% aqueous solution, ρ = 1.180 g.cm^-3^, 3.99 mmol); Et_3_N (0.61 ml, ρ = 0.727 g.cm^-3^, 4.39 mmol); recrystallized from the mixture of hexane:Et_2_O (1:1 ratio, *ca* 20 ml) to give 0.971 g (65%) of white crystalline **28**. Single crystals suitable for sc-XRD analysis were obtained by slow evaporation of hexane from saturated solution of **28** at room temperature. Mp 169 – 170.5 °C.

^1^H NMR (C_6_D_6_, 500 MHz, 295 K) δ: 9.38 (d, ^3^*J* = 7.8 Hz, 1H, Ar*H*^OAS^); 7.20 (d, ^3^*J* = 7.4 Hz, 2H, Ar*H*^Dmp^); 7.02 (t, ^3^*J* = 7.5 Hz, 2H, Ar*H*^Dmp^); 6.94 (t, ^3^*J* = 7.4 Hz, 1H, Ar*H*^OAS^); 6.84 (d, ^3^*J* = 7.4 Hz, 2H, Ar*H*^Dmp^); 6.79 (dt, ^3^*J* = 7.8 Hz, ^4^*J* = 1.1 Hz, 1H, Ar*H*^OAS^); 6.69 (s, 1H, N*H*^OAS^); 6.40 (d, ^3^*J* = 8.1 Hz, 1H, Ar*H*^OAS^); 4.59 (s, 1H, N*H*^Dmp^); 3.00 (s, 3H, OC*H*_3_); 2.43 (s, 6H, C*H*_3_^Dmp^); 2.00 (s, 6H, C*H*_3_^Dmp^). ^13^C NMR (C_6_D_6_, 125 MHz, 295 K) δ: 148.1 (Ar_q_^OAS^-OCH_3_); 147.4 (Ar_q_^Dmp^); 143.6 (Ar_q_^Gua^); 138.3 (Ar_q_^Dmp^); 135.4 (Ar_q_^Dmp^); 131.2 (Ar_q_^OAS^); 130.9 (Ar_q_^Dmp^); 129.1 (2x ArH^Dmp^); 128.0 (ArH^OAS^); 122.8 (ArH^Dmp^); 122.0 (ArH^Dmp^); 121.4 (ArH^OAS^); 119.8 (ArH^OAS^); 110.2 (ArH^OAS^); 55.6 (O*C*H_3_); 19.2 (*C*H_3_^Dmp^); 18.9 (*C*H_3_^Dmp^).

MALDI: [M+H]^+^ Calcd. for C_24_H_27_N_3_O 374.22324; found 374.22371.

**Preparation of 29**

**Method B:** *N,N'*‑bis(2,6‑diisopropylphenyl)carbodiimide (3.267 g, 9.01 mmol); diphenylamine (1.525 g, 9.01 mmol); HCl (0.80 ml of 35% aqueous solution, ρ = 1.180 g.cm^-3^, 9.01 mmol); Et_3_N (1.38 ml, ρ = 0.727 g.cm^-3^, 9.91 mmol); recrystallized from hexane (*ca* 250 ml). Yield of 3.544 g (74%) of white crystalline **29**. Single crystals suitable for sc-XRD analysis were obtained by slow evaporation of hexane from saturated solution of **29** at room temperature. Mp 171 - 172 °C. Analysis, calculated: C 83.57%, H 8.53%, N 7.90%; found: C 83.93±0.12, H 8.67±0.08, N 7.62±0.11.

^1^H NMR spectra of **29** at Tol-d_8_ during VT measurements show the presence of two tautomers. Signals of the second(very minor) tautomer of **29** at Tol-d_8_ were not assigned (and marked with black dots on NMR spectra – see Figures S71-S72). **NMR spectra @C_6_D_6_ at 295 K** - ^1^H NMR (C_6_D_6_, 500 MHz, 295 K) δ: 7.22 (br s, 2H, Ar*H*^Dipp^); 7.15-7.12 (m, 4H, Ar*H*^Ph2^); 7.09 (br s, 1H, Ar*H*^Dipp^); 6.98 (t, 4H, ^3^*J* = 7.8 Hz, Ar*H*^Ph2^); 6.91 (br s, 1H, Ar*H*^Dipp^); 6.80 (t, 2H, ^3^*J* = 7.4 Hz, Ar*H*^Ph2^); 6.76 (br s, 2H, Ar*H*^Dipp^); 5.47 (br s, 1H, N*H*^Dipp^); 3.65 (br s, 2H, C*H*^Dipp^); 3.40 (br s, 2H, C*H*^Dipp^); 1.41 (br s, 6H, C*H*_3_^Dipp^); 1.31 (d, 6H, ^3^*J* = 6.6 Hz, C*H*_3_^Dipp^); 1.10 (br s, 6H, C*H*_3_^Dipp^); 0.87 (br s, 6H, C*H*_3_^Dipp^). **NMR spectra @Tol-d_8_ at 295 K** - ^1^H NMR (Tol-d_8_, 500 MHz, 295 K) δ: 7.19 (br s, 2H, Ar*H*^Dipp^); 7.10 (s, 4H, Ar*H*^Ph2^); 7.05 (br s, 1H, Ar*H*^Dipp^); 7.01-6.95 (m, 4H, Ar*H*^Ph2^); 6.91 (br s, 1H, Ar*H*^Dipp^); 6.80 (t, 2H, ^3^*J* = 7.3 Hz, Ar*H*^Ph2^); 6.76 (br s, 2H, Ar*H*^Dipp^); 5.42 (br s, 1H, N*H*^Dipp^); 3.61 (br s, 2H, C*H*^Dipp^); 3.38 (br s, 2H, C*H*^Dipp^); 1.42 (br s, 6H, C*H*_3_^Dipp^); 1.30 (d, 6H, ^3^*J* = 6.6 Hz, C*H*_3_^Dipp^); 1.10 (br s, 6H, C*H*_3_^Dipp^); 0.87 (br s, 6H, C*H*_3_^Dipp^). **NMR spectra @Tol-d_8_ at 273 K for major tautomer** - ^1^H NMR (Tol-d_8_, 500 MHz, 273 K) δ: 7.25 (d, 2H, ^3^*J* = 6.7 Hz, Ar*H*^Dipp^); 7.12 (s, 4H, Ar*H*^Ph2^); 7.10 (br s, 1H, Ar*H*^Dipp^); 6.98 (s, 4H, Ar*H*^Ph2^); 6.88 (br s, 1H, Ar*H*^Dipp^); 6.80 (t, 2H, ^3^*J* = 7.2 Hz, Ar*H*^Ph2^); 6.72 (d, 2H, ^3^*J* = 6.8 Hz, Ar*H*^Dipp^); 5.45 (s, 1H, N*H*^Dipp^); 3.67 (s, 2H, C*H*^Dipp^); 3.34 (s, 2H, C*H*^Dipp^); 1.47 (d, 6H, ^3^*J* = 5.3 Hz, C*H*_3_^Dipp^); 1.33 (d, 6H, ^3^*J* = 5.7 Hz, C*H*_3_^Dipp^); 1.11 (d, 6H, ^3^*J* = 4.7 Hz, C*H*_3_^Dipp^); 0.83 (s, 6H, C*H*_3_^Dipp^). **NMR spectra @Tol-d_8_ at 240 K for major tautomer** - ^1^H NMR (Tol-d_8_, 500 MHz, 240 K) δ: 7.28 (d, 2H, ^3^*J* = 7.6 Hz, Ar*H*^Dipp^); 7.16-7.11 (m, 5H, Ar*H*^Ph2^+Ar*H*^Dipp^); 6.99-6.94 (m, 4H, Ar*H*^Ph2^); 6.89 (t, 1H, ^3^*J* = 7.7 Hz, Ar*H*^Dipp^); 6.81 (t, 2H, ^3^*J* = 7.3 Hz, Ar*H*^Ph2^); 6.71 (d, 2H, ^3^*J* = 7.7 Hz, Ar*H*^Dipp^); 5.47 (s, 1H, N*H*^Dipp^); 3.71 (m, 2H, ^3^*J* = 6.6 Hz, C*H*^Dipp^); 3.35 (m, 2H, ^3^*J* = 6.6 Hz, C*H*^Dipp^); 1.49 (d, 6H, ^3^*J* = 6.8 Hz, C*H*_3_^Dipp^); 1.38 (d, 6H, ^3^*J* = 6.6 Hz, C*H*_3_^Dipp^); 1.12 (d, 6H, ^3^*J* = 6.7 Hz, C*H*_3_^Dipp^); 0.83 (s, 6H, ^3^*J* = 6.7 Hz, C*H*_3_^Dipp^).^13^C NMR (Tol-d_8_, 125 MHz, 240 K) δ: 149.6 (Ar_q_^Gua^); 145.6 (Ar_q_^Ph2^); 144.8 (Ar_q_^Dipp^); 144.5 (Ar_q_^Dipp^); 139.2 (Ar_q_^Dipp^); 133.4 (Ar_q_^Dipp^); 128.7 (ArH^Ph2^); 127.0 (ArH^Dipp^); 125.6 (ArH^Ph2^); 124.5 (ArH^Ph2^); 123.6 (ArH^Dipp^); 123.6 (ArH^Dipp^); 122.9 (ArH^Dipp^);29.3 (*C*H^Dipp^); 28.6 (*C*H^Dipp^); 25.6 (*C*H_3_^Dipp^); 24.4 (*C*H_3_^Dipp^); 22.2 (*C*H_3_^Dipp^); 22.1 (*C*H_3_^Dipp^).

IC-MS: [M+H]^+^ Calcd. for C_37_H_45_N_3_ 532.36; found 532.4.

**Crystallography**

Full-set of diffraction data for **2**, **4**–**8**, **10**, **12**–**14** and **17** (see Tables S4-S13 and Table S15) were obtained at 150K using Oxford Cryostream low-temperature device on a Nonius KappaCCD diffractometer with MoK_α_ radiation (λ = 0.71073 Å), a graphite monochromator, and the φ and χ scan mode. Data reductions were performed with DENZO-SMN^[[8]](#footnote-8)^. The absorption was corrected by multi-scan method – SADABS or by integration methods.^[[9]](#footnote-9)^ Structures were solved by direct methods (Sir92)^[[10]](#footnote-10)^ and refined by full matrix least-square based on *F^2^* (SHELXL97)^[[11]](#footnote-11)^.

Full-set of diffraction data for **1**, **15**, **18**–**29**, **19**^.^HCl, **21**^.^HCl and **23**^.^HCl (see Table S3, Table S14 and Tables S16-S30) were collected at 150(2)K with a Bruker D8-Venture diffractometer equipped with Cu (Cu/Kα radiation; λ = 1.54178 Å) or Mo (Mo/Kα radiation; λ = 0.71073 Å) microfocus X-ray (IμS) sources, Photon CMOS detector and Oxford Cryosystems cooling device was used for data collection.

The frames were integrated with the Bruker SAINT software package using a narrow frame algorithm. Data were corrected for absorption effects using the Multi-Scan method (SADABS). Obtained data were treated by XT-version 2014/5 and SHELXL-2017/1 software implemented in APEX3 v2016.5-0 (Bruker AXS) system.^[[12]](#footnote-12)^

Hydrogen atoms were mostly localized on a difference Fourier map, however, to ensure uniformity of treatment of crystal, all hydrogen were recalculated into idealized positions (riding model) and assigned temperature factors H_iso_(H) = 1.2 U_eq_ (pivot atom) or of 1.5U_eq_ (methyl). H atoms in methyl, methylene, moieties and hydrogen atoms in aromatic rings were placed with C-H distances of 0.96, 0.97, and 0.93Å. Hydrogen atoms of N-H groups were refined freely or with fixed distances of 0.92Å. Disordered parts of isopropyl and coordinated THF molecules in **1** were treated by standard methods.

Crystallographic data for structural analysis have been deposited with the Cambridge Crystallographic Data Centre, CCDC no. 2120564-2120577, 2120580-2120589, 2382648-2382649, 2442574 2524367 for **1**–**2**, **4**–**8**, **10**, **12**–**15**, **17**–**29**, **19**^.^HCl, **21**^.^HCl and **23**^.^HCl . Copies of this information may be obtained free of charge from The Director, CCDC, 12 Union Road, Cambridge CB2 1EY, UK (fax: +44-1223-336033; e-mail: deposit@ccdc.cam.ac.uk or www: http://www.ccdc.cam.ac.uk).

**Table S3**. Crystal data and structure refinement for **1**.

| Crystal data | |
| --- | --- |
| Chemical formula | C_14_H_23_N_3_ |
| *M*_r_ | 233.35 |
| Crystal system, space group | Trigonal, *R*-3:*H* |
| Temperature (K) | 150 |
| *a*, *c* (Å) | 31.7688(11), 7.4694(3) |
| *V* (Å^3^) | 6528.6(5) |
| *Z* | 18 |
| Radiation type | Mo*K*α |
| µ (mm^−1^) | 0.07 |
| Crystal size (mm) | 0.59 × 0.59 × 0.32 |
| Data collection | |
| Diffractometer | Bruker D8 - Venture |
| Absorption correction | Multi-scan  *SADABS2016*/2 - Bruker AXS area detector scaling and absorption correction |
| *T*_min_, *T*_max_ | 0.667, 0.746 |
| No. of measured, independent and observed [*I* > 2σ(*I*)] reflections | 22011, 3324, 2542 |
| *R*_int_ | 0.058 |
| (sin θ/λ)_max_ (Å^−1^) | 0.650 |
| Refinement | |
| *R*[*F*^2^ > 2σ(*F*^2^)], *wR*(*F*^2^), *S* | 0.049, 0.127, 1.04 |
| No. of reflections | 3324 |
| No. of parameters | 166 |
| No. of restraints | 114 |
| H-atom treatment | H-atoms treated by a mixture of independent and constrained refinement |
| Δρ_max_, Δρ_min_ (e Å^−3^) | 0.18, −0.24 |

Computer programs: Bruker Instrument Service vV6.2.3, *APEX3* v2016.5-0 (Bruker AXS), *SAINT* V8.37A (Bruker AXS Inc., 2015), XT, VERSION 2014/5, *SHELXL2014*/7 (Sheldrick, 2014), *PLATON* (Spek, 2009).

Hydrogen-bond geometry (Å, °)

| *D*—H···*A* | *D*—H | H···*A* | *D*···*A* | *D*—H···*A* |
| --- | --- | --- | --- | --- |
| N3—H3···N2^i^ | 0.878(18) | 2.178(18) | 3.0442(16) | 169.2(15) |

Symmetry code: (i) x−y+1/3, x−1/3, −z+2/3.

**Table S4.** Crystal data and structure refinement for **2**.

| Crystal data | |
| --- | --- |
| Chemical formula | C_29_H_45_N_3_ |
| *M*_r_ | 435.68 |
| Crystal system, space group | Monoclinic, *P*2_1_/*c* |
| Temperature (K) | 150 |
| *a*, *b*, *c* (Å) | 15.6690(12), 10.8960(7), 19.6221(14) |
| β (°) | 125.408(7) |
| *V* (Å^3^) | 2730.5(4) |
| *Z* | 4 |
| Radiation type | Mo*K*α |
| µ (mm^−1^) | 0.06 |
| Crystal size (mm) | 0.55 × 0.41 × 0.39 |
| Data collection | |
| Diffractometer | Bruker Nonius KappaCCD area detector |
| Absorption correction | Integration  Gaussian integration (Coppens, 1970) |
| *T*_min_, *T*_max_ | 0.978, 0.988 |
| No. of measured, independent and observed [*I* > 2σ(*I*)] reflections | 17392, 5688, 3884 |
| *R*_int_ | 0.043 |
| (sin θ/λ)_max_ (Å^−1^) | 0.633 |
| Refinement | |
| *R*[*F*^2^ > 2σ(*F*^2^)], *wR*(*F*^2^), *S* | 0.058, 0.140, 1.22 |
| No. of reflections | 5688 |
| No. of parameters | 289 |
| No. of restraints | 0 |
| H-atom treatment | H-atoms treated by a mixture of independent and constrained refinement |
| Δρ_max_, Δρ_min_ (e Å^−3^) | 0.31, −0.26 |

Computer programs: *COLLECT* (Hooft, 1998) and *DENZO* (Otwinowski & Minor, 1997), *COLLECT* and *DENZO*, *SIR92* (Altomare *et al*., 1994), *SHELXL97* (Sheldrick, 2008), *PLATON* (Spek, 2003).

**Table S5.** Crystal data and structure refinement for **4**.

| Crystal data | |
| --- | --- |
| Chemical formula | C_32_H_43_N_3_ |
| *M*_r_ | 469.69 |
| Crystal system, space group | Monoclinic, *C*2/*c* |
| Temperature (K) | 150 |
| *a*, *b*, *c* (Å) | 24.3102(4), 15.8240(2), 19.1463(5) |
| β (°) | 128.232(3) |
| *V* (Å^3^) | 5785.5(3) |
| *Z* | 8 |
| Radiation type | Mo*K*α |
| µ (mm^−1^) | 0.06 |
| Crystal size (mm) | 0.59 × 0.42 × 0.38 |
| Data collection | |
| Diffractometer | Bruker Nonius KappaCCD area detector |
| Absorption correction | Integration  Gaussian integration (Coppens, 1970) |
| *T*_min_, *T*_max_ | 0.976, 0.985 |
| No. of measured, independent and observed [*I* > 2σ(*I*)] reflections | 28914, 6276, 4229 |
| *R*_int_ | 0.080 |
| (sin θ/λ)_max_ (Å^−1^) | 0.639 |
| Refinement | |
| *R*[*F*^2^ > 2σ(*F*^2^)], *wR*(*F*^2^), *S* | 0.061, 0.151, 1.21 |
| No. of reflections | 6276 |
| No. of parameters | 316 |
| No. of restraints | 0 |
| H-atom treatment | H-atoms treated by a mixture of independent and constrained refinement |
| Δρ_max_, Δρ_min_ (e Å^−3^) | 0.25, −0.25 |

Computer programs: *COLLECT* (Hooft, 1998) and *DENZO* (Otwinowski & Minor, 1997), *COLLECT* and *DENZO*, *SIR92* (Altomare *et al*., 1994), *SHELXL97* (Sheldrick, 2008), *PLATON* (Spek, 2003).

**Table S6.** Crystal data and structure refinement for **5**.

| Crystal data | |
| --- | --- |
| Chemical formula | C_33_H_45_N_3_ |
| *M*_r_ | 483.72 |
| Crystal system, space group | Triclinic, *P*-1 |
| Temperature (K) | 150 |
| *a*, *b*, *c* (Å) | 9.0930(7), 9.3500(4), 18.9961(11) |
| α, β, γ (°) | 78.306(4), 83.689(5), 65.985(4) |
| *V* (Å^3^) | 1443.94(16) |
| *Z* | 2 |
| Radiation type | Mo*K*α |
| µ (mm^−1^) | 0.07 |
| Crystal size (mm) | 0.36 × 0.22 × 0.14 |
| Data collection | |
| Diffractometer | Bruker Nonius KappaCCD area detector |
| Absorption correction | Integration  Gaussian integration (Coppens, 1970) |
| *T*_min_, *T*_max_ | 0.984, 0.993 |
| No. of measured, independent and observed [*I* > 2σ(*I*)] reflections | 17020, 5178, 3383 |
| *R*_int_ | 0.120 |
| (sin θ/λ)_max_ (Å^−1^) | 0.601 |
| Refinement | |
| *R*[*F*^2^ > 2σ(*F*^2^)], *wR*(*F*^2^), *S* | 0.071, 0.164, 1.17 |
| No. of reflections | 5178 |
| No. of parameters | 325 |
| No. of restraints | 0 |
| H-atom treatment | H-atoms treated by a mixture of independent and constrained refinement |
| Δρ_max_, Δρ_min_ (e Å^−3^) | 0.20, −0.23 |

Computer programs: *COLLECT* (Hooft, 1998) and *DENZO* (Otwinowski & Minor, 1997), *COLLECT* and *DENZO*, *SIR92* (Altomare *et al*., 1994), *SHELXL97* (Sheldrick, 2008), *PLATON*

**Table S7.** Crystal data and structure refinement for **6**.

| Crystal data | |
| --- | --- |
| Chemical formula | C_31_H_42_N_4_ |
| *M*_r_ | 470.69 |
| Crystal system, space group | Triclinic, *P*-1 |
| Temperature (K) | 150 |
| *a*, *b*, *c* (Å) | 9.1091(6), 16.6279(19), 21.2851(15) |
| α, β, γ (°) | 67.582(6), 89.544(6), 74.530(7) |
| *V* (Å^3^) | 2856.3(4) |
| *Z* | 4 |
| Radiation type | Mo*K*α |
| µ (mm^−1^) | 0.07 |
| Crystal size (mm) | 0.56 × 0.42 × 0.36 |
| Data collection | |
| Diffractometer | Bruker Nonius KappaCCD area detector |
| Absorption correction | Integration  Gaussian integration (Coppens, 1970) |
| *T*_min_, *T*_max_ | 0.973, 0.987 |
| No. of measured, independent and observed [*I* > 2σ(*I*)] reflections | 52587, 11954, 7782 |
| *R*_int_ | 0.070 |
| (sin θ/λ)_max_ (Å^−1^) | 0.634 |
| Refinement | |
| *R*[*F*^2^ > 2σ(*F*^2^)], *wR*(*F*^2^), *S* | 0.059, 0.153, 1.17 |
| No. of reflections | 11954 |
| No. of parameters | 631 |
| H-atom treatment | H-atoms parameters constrained |
| Δρ_max_, Δρ_min_ (e Å^−3^) | 0.52, −0.34 |

Computer programs: *COLLECT* (Hooft, 1998) and *DENZO* (Otwinowski & Minor, 1997), *COLLECT* and *DENZO*, *SIR92* (Altomare *et al*., 1994), *SHELXL97* (Sheldrick, 2008), *PLATON* (Spek, 2003).

Hydrogen-bond geometry (Å, °)

| *D*—H···*A* | *D*—H | H···*A* | *D*···*A* | *D*—H···*A* |
| --- | --- | --- | --- | --- |
| N101—H101···N102^i^ | 0.98 | 2.51 | 3.227(3) | 129 |

Symmetry code: (i) −x, −y+1, −z+1.

**Table S8.** Crystal data and structure refinement for **7**.

| Crystal data | |
| --- | --- |
| Chemical formula | C_32_H_44_N_4_ |
| *M*_r_ | 484.71 |
| Crystal system, space group | Triclinic, *P*-1 |
| Temperature (K) | 150 |
| *a*, *b*, *c* (Å) | 10.7330(11), 12.5561(13), 22.4081(16) |
| α, β, γ (°) | 94.656(6), 96.530(8), 100.603(9) |
| *V* (Å^3^) | 2932.8(5) |
| *Z* | 4 |
| Radiation type | Mo*K*α |
| µ (mm^−1^) | 0.07 |
| Crystal size (mm) | 0.46 × 0.38 × 0.24 |
| Data collection | |
| Diffractometer | Bruker Nonius KappaCCD area detector |
| Absorption correction | Integration  Gaussian integration (Coppens, 1970) |
| *T*_min_, *T*_max_ | 0.979, 0.987 |
| No. of measured, independent and observed [*I* > 2σ(*I*)] reflections | 47293, 11963, 8714 |
| *R*_int_ | 0.059 |
| (sin θ/λ)_max_ (Å^−1^) | 0.628 |
| Refinement | |
| *R*[*F*^2^ > 2σ(*F*^2^)], *wR*(*F*^2^), *S* | 0.066, 0.175, 1.17 |
| No. of reflections | 11963 |
| No. of parameters | 649 |
| No. of restraints | 0 |
| H-atom treatment | H-atoms treated by a mixture of independent and constrained refinement |
| Δρ_max_, Δρ_min_ (e Å^−3^) | 0.34, −0.28 |

Computer programs: *COLLECT* (Hooft, 1998) and *DENZO* (Otwinowski & Minor, 1997), *COLLECT* and *DENZO*, *SIR92* (Altomare *et al*., 1994), *SHELXL97* (Sheldrick, 2008), *PLATON* (Spek, 2003).

**Table S9.** Crystal data and structure refinement for **8**.

| Crystal data | |
| --- | --- |
| Chemical formula | C_32_H_44_N_4_ |
| *M*_r_ | 484.71 |
| Crystal system, space group | Monoclinic, *C*2/*c* |
| Temperature (K) | 150 |
| *a*, *b*, *c* (Å) | 28.1712(5), 8.9483(5), 22.7152(4) |
| β (°) | 97.121(3) |
| *V* (Å^3^) | 5682.0(3) |
| *Z* | 8 |
| Radiation type | Mo*K*α |
| µ (mm^−1^) | 0.07 |
| Crystal size (mm) | 0.38 × 0.37 × 0.22 |
| Data collection | |
| Diffractometer | Bruker Nonius KappaCCD area detector |
| Absorption correction | Integration  Gaussian integration (Coppens, 1970) |
| *T*_min_, *T*_max_ | 0.981, 0.988 |
| No. of measured, independent and observed [*I* > 2σ(*I*)] reflections | 40034, 6447, 5027 |
| *R*_int_ | 0.032 |
| (sin θ/λ)_max_ (Å^−1^) | 0.650 |
| Refinement | |
| *R*[*F*^2^ > 2σ(*F*^2^)], *wR*(*F*^2^), *S* | 0.047, 0.125, 1.10 |
| No. of reflections | 6447 |
| No. of parameters | 325 |
| No. of restraints | 0 |
| H-atom treatment | H-atoms treated by a mixture of independent and constrained refinement |
| Δρ_max_, Δρ_min_ (e Å^−3^) | 0.21, −0.27 |

Computer programs: *COLLECT* (Hooft, 1998) and *DENZO* (Otwinowski & Minor, 1997), *COLLECT* and *DENZO*, *SIR92* (Altomare *et al*., 1994), *SHELXL97* (Sheldrick, 2008), *PLATON* (Spek, 2003).

Hydrogen-bond geometry (Å, °)

| *D*—H···*A* | *D*—H | H···*A* | *D*···*A* | *D*—H···*A* |
| --- | --- | --- | --- | --- |
| N3—H3···N4^i^ | 0.81 | 2.37 | 3.1693(17) | 166 |

Symmetry code: (i) −*x*+1, *y*, −*z*+3/2.

**Table S10.** Crystal data and structure refinement for **10**.

| Crystal data | |
| --- | --- |
| Chemical formula | C_34_H_47_N_3_O |
| *M*_r_ | 513.74 |
| Crystal system, space group | Orthorhombic, *P*2_1_2_1_2_1_ |
| Temperature (K) | 150 |
| *a*, *b*, *c* (Å) | 10.6851(11), 16.1000(16), 18.3610(18) |
| *V* (Å^3^) | 3158.6(5) |
| *Z* | 4 |
| Radiation type | Mo*K*α |
| µ (mm^−1^) | 0.07 |
| Crystal size (mm) | 0.59 × 0.42 × 0.34 |
| Data collection | |
| Diffractometer | Bruker Nonius KappaCCD area detector |
| Absorption correction | Integration  Gaussian integration (Coppens, 1970) |
| *T*_min_, *T*_max_ | 0.973, 0.982 |
| No. of measured, independent and observed [*I* > 2σ(*I*)] reflections | 28609, 6753, 5501 |
| *R*_int_ | 0.045 |
| (sin θ/λ)_max_ (Å^−1^) | 0.639 |
| Refinement | |
| *R*[*F*^2^ > 2σ(*F*^2^)], *wR*(*F*^2^), *S* | 0.049, 0.117, 1.22 |
| No. of reflections | 6753 |
| No. of parameters | 344 |
| No. of restraints | 0 |
| H-atom treatment | H-atoms treated by a mixture of independent and constrained refinement |
| Δρ_max_, Δρ_min_ (e Å^−3^) | 0.23, −0.21 |
| Absolute structure | Flack x determined using 2009 quotients [(I+)-(I-)]/[(I+)+(I-)] (Parsons, Flack and Wagner, Acta Cryst. B69 (2013) 249-259). |

Computer programs: *COLLECT* (Hooft, 1998) and *DENZO* (Otwinowski & Minor, 1997), *COLLECT* and *DENZO*, *SIR92* (Altomare *et al*., 1994), *SHELXL2017/*1 (Sheldrick, 2017), *PLATON* (Spek, 2003), *SHELXL97* (Sheldrick, 2008).

Hydrogen-bond geometry (Å, °)

| *D*—H···*A* | *D*—H | H···*A* | *D*···*A* | *D*—H···*A* |
| --- | --- | --- | --- | --- |
| N2—H2···O1^i^ | 0.972(2) | 2.302(2) | 2.971(3) | 125.26(13) |

Symmetry code: (i) −*x*+2, *y*−1/2, −*z*+1/2.

**Table S11.** Crystal data and structure refinement for **12**.

| Crystal data | |
| --- | --- |
| Chemical formula | C_30_H_48_N_4_ |
| *M*_r_ | 464.72 |
| Crystal system, space group | Monoclinic, *P*2_1_/*c* |
| Temperature (K) | 150 |
| *a*, *b*, *c* (Å) | 11.723(1), 16.4900(12), 18.9630(9) |
| β (°) | 126.754(6) |
| *V* (Å^3^) | 2937.1(4) |
| *Z* | 4 |
| Radiation type | Mo*K*α |
| µ (mm^−1^) | 0.06 |
| Crystal size (mm) | 0.40 × 0.32 × 0.31 |
| Data collection | |
| Diffractometer | Bruker Nonius KappaCCD area detector |
| Absorption correction | Integration  Gaussian integration (Coppens, 1970) |
| *T*_min_, *T*_max_ | 0.979, 0.987 |
| No. of measured, independent and observed [*I* > 2σ(*I*)] reflections | 22886, 5737, 3774 |
| *R*_int_ | 0.083 |
| (sin θ/λ)_max_ (Å^−1^) | 0.617 |
| Refinement | |
| *R*[*F*^2^ > 2σ(*F*^2^)], *wR*(*F*^2^), *S* | 0.072, 0.201, 1.02 |
| No. of reflections | 5737 |
| No. of parameters | 312 |
| No. of restraints | 0 |
| H-atom treatment | H-atom parameters constrained |
| Δρ_max_, Δρ_min_ (e Å^−3^) | 0.63, −0.32 |

Computer programs: *COLLECT* (Hooft, 1998) and *DENZO* (Otwinowski & Minor, 1997), *COLLECT* and *DENZO*, *SIR92* (Altomare *et al*., 1994), *SHELXL2017/*1 (Sheldrick, 2017), *PLATON* (Spek, 2003), *SHELXL97* (Sheldrick, 2008).

Hydrogen-bond geometry (Å, °)

| *D*—H···*A* | *D*—H | H···*A* | *D*···*A* | *D*—H···*A* |
| --- | --- | --- | --- | --- |
| N3—H3···N4 | 0.92 | 2.13 | 2.856(3) | 135 |

**Table S12.** Crystal data and structure refinement for **13**.

| Crystal data | |
| --- | --- |
| Chemical formula | C_34_H_48_N_4_ |
| *M*_r_ | 512.76 |
| Crystal system, space group | Triclinic, *P*-1 |
| Temperature (K) | 150 |
| *a*, *b*, *c* (Å) | 8.400(3), 9.5430(18), 10.979(4) |
| α, β, γ (°) | 87.30(2), 76.52(3), 65.93(2) |
| *V* (Å^3^) | 780.3(4) |
| *Z* | 1 |
| Radiation type | Mo*K*α |
| µ (mm^−1^) | 0.06 |
| Crystal size (mm) | 0.59 × 0.43 × 0.05 |
| Data collection | |
| Diffractometer | Bruker Nonius KappaCCD area detector |
| Absorption correction | Integration  Gaussian integration (Coppens, 1970) |
| *T*_min_, *T*_max_ | 0.981, 0.997 |
| No. of measured, independent and observed [*I* > 2σ(*I*)] reflections | 14231, 6313, 5520 |
| *R*_int_ | 0.068 |
| (sin θ/λ)_max_ (Å^−1^) | 0.639 |
| Refinement | |
| *R*[*F*^2^ > 2σ(*F*^2^)], *wR*(*F*^2^), *S* | 0.053, 0.138, 1.13 |
| No. of reflections | 6313 |
| No. of parameters | 343 |
| No. of restraints | 3 |
| H-atom treatment | H-atom parameters constrained |
| Δρ_max_, Δρ_min_ (e Å^−3^) | 0.21, −0.28 |
| Absolute structure | Flack x determined using 2229 quotients [(I+)-(I-)]/[(I+)+(I-)] (Parsons, Flack and Wagner, Acta Cryst. B69 (2013) 249-259). |

Computer programs: *COLLECT* (Hooft, 1998) and *DENZO* (Otwinowski & Minor, 1997), *COLLECT* and *DENZO*, *SIR92* (Altomare *et al*., 1994), *SHELXL97* (Sheldrick, 2008), *PLATON* (Spek, 2003).

Hydrogen-bond geometry (Å, °)

| *D*—H···*A* | *D*—H | H···*A* | *D*···*A* | *D*—H···*A* |
| --- | --- | --- | --- | --- |
| N1—H1···N2 | 0.88 | 2.16 | 2.888(4) | 140 |

**Table S13.** Crystal data and structure refinement for **14**.

| Crystal data | |
| --- | --- |
| Chemical formula | C_39_H_50_N_4_ |
| *M*_r_ | 574.83 |
| Crystal system, space group | Monoclinic, *P*2_1_/*c* |
| Temperature (K) | 150 |
| *a*, *b*, *c* (Å) | 11.174(1), 18.8980(12), 16.2880(12) |
| β (°) | 103.827(7) |
| *V* (Å^3^) | 3339.8(5) |
| *Z* | 4 |
| Radiation type | Mo*K*α |
| µ (mm^−1^) | 0.07 |
| Crystal size (mm) | 0.59 × 0.25 × 0.10 |
| Data collection | |
| Diffractometer | Bruker Nonius KappaCCD area detector |
| Absorption correction | Integration  Gaussian integration (Coppens, 1970) |
| *T*_min_, *T*_max_ | 0.973, 0.995 |
| No. of measured, independent and observed [*I* > 2σ(*I*)] reflections | 20853, 6166, 4182 |
| *R*_int_ | 0.075 |
| (sin θ/λ)_max_ (Å^−1^) | 0.608 |
| Refinement | |
| *R*[*F*^2^ > 2σ(*F*^2^)], *wR*(*F*^2^), *S* | 0.064, 0.153, 1.19 |
| No. of reflections | 6166 |
| No. of parameters | 391 |
| No. of restraints | 1 |
| H-atom treatment | H-atom parameters constrained |
| Δρ_max_, Δρ_min_ (e Å^−3^) | 0.26, −0.25 |

Computer programs: *COLLECT* (Hooft, 1998) and *DENZO* (Otwinowski & Minor, 1997), *COLLECT* and *DENZO*, *SIR92* (Altomare *et al*., 1994), *SHELXL2017/*1 (Sheldrick, 2017), *PLATON* (Spek, 2003), *SHELXL97* (Sheldrick, 2008).

Hydrogen-bond geometry (Å, °)

| *D*—H···*A* | *D*—H | H···*A* | *D*···*A* | *D*—H···*A* |
| --- | --- | --- | --- | --- |
| N2—H2···N1^i^ | 0.99 | 2.11 | 3.054(3) | 159 |

Symmetry code: (i) x, −y+1/2, z−1/2.

**Table S14.** Crystal data and structure refinement for **15**.

| Crystal data | |
| --- | --- |
| Chemical formula | C_64_H_84_N_6_ |
| *M*_r_ | 937.37 |
| Crystal system, space group | Monoclinic, *C*2/*c* |
| Temperature (K) | 150 |
| *a*, *b*, *c* (Å) | 21.283(14), 10.852(7), 26.792(12) |
| β (°) | 105.112(16) |
| *V* (Å^3^) | 5974(6) |
| *Z* | 4 |
| Radiation type | Mo*K*α |
| µ (mm^−1^) | 0.06 |
| Crystal size (mm) | 0.34 × 0.27 × 0.25 |
| Data collection | |
| Diffractometer | Bruker D8 - Venture |
| Absorption correction | Multi-scan  *SADABS2016*/2 - Bruker AXS area detector scaling and absorption correction |
| *T*_min_, *T*_max_ | 0.698, 0.746 |
| No. of measured, independent and observed [*I* > 2σ(*I*)] reflections | 75335, 6869, 5268 |
| *R*_int_ | 0.077 |
| (sin θ/λ)_max_ (Å^−1^) | 0.651 |
| Refinement | |
| *R*[*F*^2^ > 2σ(*F*^2^)], *wR*(*F*^2^), *S* | 0.063, 0.177, 1.06 |
| No. of reflections | 6869 |
| No. of parameters | 333 |
| No. of restraints | 264 |
| H-atom treatment | H-atoms treated by a mixture of independent and constrained refinement |
| Δρ_max_, Δρ_min_ (e Å^−3^) | 0.45, −0.34 |

Computer programs: Bruker Instrument Service vV6.2.3, *APEX3* v2016.5-0 (Bruker AXS), *SAINT* V8.37A (Bruker AXS Inc., 2015), XT, VERSION 2014/5, *SHELXL2014*/7 (Sheldrick, 2014), *PLATON* (Spek, 2009).

Symmetry code: (i) −x+1, y, −z+3/2

**Table S15.** Crystal data and structure refinement for **17**.

| Crystal data | |
| --- | --- |
| Chemical formula | C_81_H_120_N_10.50_ |
| *M*_r_ | 1240.88 |
| Crystal system, space group | Triclinic, *P-*1 |
| Temperature (K) | 150 |
| *a*, *b*, *c* (Å) | 11.261(5), 14.355(5), 24.471(8) |
| α, β, γ (°) | 87.92(3), 81.13(3), 82.16(5) |
| *V* (Å^3^) | 3871(2) |
| *Z* | 2 |
| Radiation type | Mo*K*α |
| µ (mm^−1^) | 0.06 |
| Crystal size (mm) | 0.26 × 0.18 × 0.16 |
| Data collection | |
| Diffractometer | Bruker Nonius KappaCCD area detector |
| Absorption correction | - |
| No. of measured, independent and observed [*I* > 2σ(*I*)] reflections | 51709, 26828, 17382 |
| *R*_int_ | 0.060 |
| (sin θ/λ)_max_ (Å^−1^) | 0.612 |
| Refinement | |
| *R*[*F*^2^ > 2σ(*F*^2^)], *wR*(*F*^2^), *S* | 0.098, 0.320, 1.10 |
| No. of reflections | 26828 |
| No. of parameters | 1649 |
| No. of restraints | 117 |
| H-atom treatment | H-atoms treated by a mixture of independent and constrained refinement |
| Δρ_max_, Δρ_min_ (e Å^−3^) | 1.40, −0.43 |
| Absolute structure | Flack H D (1983), Acta Cryst. A39, 876-881 |
| Absolute structure parameter | −5(4) |

Computer programs: *COLLECT* (Hooft, 1998) and *DENZO* (Otwinowski & Minor, 1997), *COLLECT* and *DENZO*, *SIR92* (Altomare *et al.*, 1994), *SHELXL97* (Sheldrick, 2008), *PLATON* (Spek, 2003).

**Table S16.** Crystal data and structure refinement for **18**.

| Crystal data | |
| --- | --- |
| Chemical formula | C_19_H_29_N_3_ |
| *M*_r_ | 299.45 |
| Crystal system, space group | Monoclinic, *P*2_1_/*n* |
| Temperature (K) | 150 |
| *a*, *b*, *c* (Å) | 11.7016(5), 16.9033(6), 17.8026(7) |
| β (°) | 94.192(2) |
| *V* (Å^3^) | 3511.9(2) |
| *Z* | 8 |
| Radiation type | Mo*K*α |
| µ (mm^−1^) | 0.07 |
| Crystal size (mm) | 0.56 × 0.48 × 0.37 |
| Data collection | |
| Diffractometer | Bruker D8 - Venture |
| Absorption correction | Multi-scan  *SADABS2016*/2 - Bruker AXS area detector scaling and absorption correction |
| *T*_min_, *T*_max_ | 0.679, 0.746 |
| No. of measured, independent and observed [*I* > 2σ(*I*)] reflections | 51864, 8089, 6006 |
| *R*_int_ | 0.048 |
| (sin θ/λ)_max_ (Å^−1^) | 0.652 |
| Refinement | |
| *R*[*F*^2^ > 2σ(*F*^2^)], *wR*(*F*^2^), *S* | 0.050, 0.126, 1.04 |
| No. of reflections | 8089 |
| No. of parameters | 414 |
| No. of restraints | 324 |
| H-atom treatment | H-atoms treated by a mixture of independent and constrained refinement |
| Δρ_max_, Δρ_min_ (e Å^−3^) | 0.33, −0.21 |

Computer programs: Bruker Instrument Service vV6.2.3, *APEX3* v2016.5-0 (Bruker AXS), *SAINT* V8.37A (Bruker AXS Inc., 2015), XT, VERSION 2014/5, *SHELXL2014*/7 (Sheldrick, 2014), *PLATON* (Spek, 2009).

Hydrogen-bond geometry (Å, °)

| *D*—H···*A* | *D*—H | H···*A* | *D*···*A* | *D*—H···*A* |
| --- | --- | --- | --- | --- |
| N1′—H1′···N6′ | 0.846(19) | 2.227(19) | 3.0366(18) | 160.4(16) |
| N2′—H2′···N6′ | 0.87(2) | 2.36(2) | 3.1644(17) | 153.6(16) |
| N4′—H4′···N3′^i^ | 0.91(2) | 2.40(2) | 3.2387(19) | 153.4(17) |
| N5′—H5′···N3′^i^ | 0.87(2) | 2.20(2) | 3.0472(18) | 167.0(18) |

Symmetry code: (i) −x+1/2, y−1/2, −z+3/2

**Table S17.** Crystal data and structure refinement for **19**.

| Crystal data | |
| --- | --- |
| Chemical formula | C_20_H_31_N_3_O |
| *M*_r_ | 329.48 |
| Crystal system, space group | Tetragonal, *P*4_3_2_1_2 |
| Temperature (K) | 150 |
| *a*, *c* (Å) | 15.437(3), 16.690(3) |
| *V* (Å^3^) | 3977.2(17) |
| *Z* | 8 |
| Radiation type | Cu*K*α |
| µ (mm^−1^) | 0.53 |
| Crystal size (mm) | 0.59 × 0.28 × 0.17 |
| Data collection | |
| Diffractometer | Bruker D8 - Venture |
| Absorption correction | Multi-scan  *SADABS2016*/2 - Bruker AXS area detector scaling and absorption correction |
| *T*_min_, *T*_max_ | 0.583, 0.754 |
| No. of measured, independent and observed [*I* > 2σ(*I*)] reflections | 38949, 3912, 3464 |
| *R*_int_ | 0.088 |
| (sin θ/λ)_max_ (Å^−1^) | 0.618 |
| Refinement | |
| *R*[*F*^2^ > 2σ(*F*^2^)], *wR*(*F*^2^), *S* | 0.060, 0.115, 1.18 |
| No. of reflections | 3912 |
| No. of parameters | 243 |
| No. of restraints | 249 |
| H-atom treatment | H-atoms treated by a mixture of independent and constrained refinement |
| Δρ_max_, Δρ_min_ (e Å^−3^) | 0.14, −0.21 |
| Absolute structure | Refined as a perfect inversion twin. |
| Absolute structure parameter | 0.5 |

Computer programs: Bruker Instrument Service vV6.2.3, *APEX3* v2016.5-0 (Bruker AXS), *SAINT* V8.37A (Bruker AXS Inc., 2015), XT, VERSION 2014/5, *SHELXL2014*/7 (Sheldrick, 2014), *PLATON* (Spek, 2009).

**Table S18.** Crystal data and structure refinement for **19**^.^HCl.

| Crystal data | |
| --- | --- |
| Chemical formula | C_20_H_32_N_3_O·Cl |
| *M*_r_ | 365.93 |
| Crystal system, space group | Triclinic, *P-*1 |
| Temperature (K) | 150 |
| *a*, *b*, *c* (Å) | 7.5235(2), 11.3503(4), 11.9666(4) |
| α, β, γ (°) | 95.860(2), 92.796(2), 103.255(2) |
| *V* (Å^3^) | 986.70(6) |
| *Z* | 2 |
| Radiation type | Mo*K*α |
| µ (mm^−1^) | 0.21 |
| Crystal size (mm) | 0.44 × 0.17 × 0.16 |
| Data collection | |
| Diffractometer | Bruker D8 - Venture |
| Absorption correction | Multi-scan  *SADABS2016*/2 - Bruker AXS area detector scaling and absorption correction |
| *T*_min_, *T*_max_ | 0.644, 0.746 |
| No. of measured, independent and observed [*I* > 2σ(*I*)] reflections | 27843, 4546, 3608 |
| *R*_int_ | 0.057 |
| (sin θ/λ)_max_ (Å^−1^) | 0.650 |
| Refinement | |
| *R*[*F*^2^ > 2σ(*F*^2^)], *wR*(*F*^2^), *S* | 0.039, 0.092, 1.02 |
| No. of reflections | 4546 |
| No. of parameters | 239 |
| No. of restraints | 177 |
| H-atom treatment | H-atoms treated by a mixture of independent and constrained refinement |
| Δρ_max_, Δρ_min_ (e Å^−3^) | 0.26, −0.23 |

Computer programs: Bruker Instrument Service vV6.2.3, *APEX3* v2016.5-0 (Bruker AXS), *SAINT* V8.37A (Bruker AXS Inc., 2015), XT, VERSION 2014/5, *SHELXL2014/7* (Sheldrick, 2014), *PLATON* (Spek, 2009).

Hydrogen-bond geometry (Å, °)

| *D*—H···*A* | *D*—H | H···*A* | *D*···*A* | *D*—H···*A* |
| --- | --- | --- | --- | --- |
| N1—H1···Cl1 | 0.875(18) | 2.309(19) | 3.1676(13) | 167.1(15) |
| N2—H2···Cl1^i^ | 0.81(2) | 2.71(2) | 3.4059(14) | 143.7(18) |
| N3—H3···Cl1^i^ | 0.892(19) | 2.293(19) | 3.1735(12) | 169.2(16) |

Symmetry code: (i) x+1, y, z

**Table S19.** Crystal data and structure refinement for **20**.

| Crystal data | |
| --- | --- |
| Chemical formula | C_31_H_41_N_3_ |
| *M*_r_ | 455.67 |
| Crystal system, space group | Triclinic, *P-*1 |
| Temperature (K) | 150 |
| *a*, *b*, *c* (Å) | 11.1923(10), 15.4958(12), 17.6626(15) |
| α, β, γ (°) | 66.829(3), 84.901(4), 76.368(3) |
| *V* (Å^3^) | 2736.8(4) |
| *Z* | 4 |
| Radiation type | Mo*K*α |
| µ (mm^−1^) | 0.06 |
| Crystal size (mm) | 0.59 × 0.36 × 0.31 |
| Data collection | |
| Diffractometer | Bruker D8 - Venture |
| Absorption correction | Multi-scan  *SADABS2016*/2 - Bruker AXS area detector scaling and absorption correction |
| *T*_min_, *T*_max_ | 0.684, 0.746 |
| No. of measured, independent and observed [*I* > 2σ(*I*)] reflections | 93075, 14713, 8682 |
| *R*_int_ | 0.103 |
| (sin θ/λ)_max_ (Å^−1^) | 0.687 |
| Refinement | |
| *R*[*F*^2^ > 2σ(*F*^2^)], *wR*(*F*^2^), *S* | 0.068, 0.188, 1.03 |
| No. of reflections | 14713 |
| No. of parameters | 663 |
| No. of restraints | 546 |
| H-atom treatment | H-atoms treated by a mixture of independent and constrained refinement |
| Δρ_max_, Δρ_min_ (e Å^−3^) | 0.41, −0.44 |

Computer programs: Bruker Instrument Service vV6.2.3, *APEX3* v2016.5-0 (Bruker AXS), *SAINT* V8.37A (Bruker AXS Inc., 2015), XT, VERSION 2014/5, *SHELXL2014/7* (Sheldrick, 2014), *PLATON* (Spek, 2009).

**Table S20.** Crystal data and structure refinement for **21**.

| Crystal data | |
| --- | --- |
| Chemical formula | C_32_H_43_N_3_O |
| *M*_r_ | 485.69 |
| Crystal system, space group | Triclinic, *P*-1 |
| Temperature (K) | 150 |
| *a*, *b*, *c* (Å) | 8.729(2), 11.450(4), 15.790(4) |
| α, β, γ (°) | 103.739(13), 101.607(8), 105.737(12) |
| *V* (Å^3^) | 1414.7(6) |
| *Z* | 2 |
| Radiation type | Mo*K*α |
| µ (mm^−1^) | 0.07 |
| Crystal size (mm) | 0.26 × 0.18 × 0.12 |
| Data collection | |
| Diffractometer | Bruker D8 - Venture |
| Absorption correction | Multi-scan  *SADABS2016*/2 - Bruker AXS area detector scaling and absorption correction |
| *T*_min_, *T*_max_ | 0.695, 0.746 |
| No. of measured, independent and observed [*I* > 2σ(*I*)] reflections | 31497, 6493, 4047 |
| *R*_int_ | 0.099 |
| (sin θ/λ)_max_ (Å^−1^) | 0.652 |
| Refinement | |
| *R*[*F*^2^ > 2σ(*F*^2^)], *wR*(*F*^2^), *S* | 0.060, 0.129, 1.04 |
| No. of reflections | 6493 |
| No. of parameters | 342 |
| No. of restraints | 0 |
| H-atom treatment | H-atoms treated by a mixture of independent and constrained refinement |
| Δρ_max_, Δρ_min_ (e Å^−3^) | 0.20, −0.26 |

Computer programs: Bruker Instrument Service vV6.2.3, *APEX3* v2016.5-0 (Bruker AXS), *SAINT* V8.37A (Bruker AXS Inc., 2015), XT, VERSION 2014/5, *SHELXL2014*/7 (Sheldrick, 2014), *PLATON* (Spek, 2009).

Hydrogen-bond geometry (Å, °)

| *D*—H···*A* | *D*—H | H···*A* | *D*···*A* | *D*—H···*A* |
| --- | --- | --- | --- | --- |
| N1—H1···O1 | 0.90(2) | 2.07(2) | 2.564(2) | 113.0(16) |

**Table S21.** Crystal data and structure refinement for **21**^.^HCl.

| Crystal data | |
| --- | --- |
| Chemical formula | C_32_H_44_N_3_O·Cl |
| *M*_r_ | 522.15 |
| Crystal system, space group | Orthorhombic, *Pna*2_1_ |
| Temperature (K) | 150 |
| *a*, *b*, *c* (Å) | 18.2350(8), 15.0592(7), 10.9577(6) |
| *V* (Å^3^) | 3009.0(3) |
| *Z* | 4 |
| Radiation type | Mo*K*α |
| µ (mm^−1^) | 0.16 |
| Crystal size (mm) | 0.41 × 0.22 × 0.18 |
| Data collection | |
| Diffractometer | Bruker D8 - Venture |
| Absorption correction | Multi-scan  *SADABS2016*/2 - Bruker AXS area detector scaling and absorption correction |
| *T*_min_, *T*_max_ | 0.653, 0.746 |
| No. of measured, independent and observed [*I* > 2σ(*I*)] reflections | 28323, 6858, 6221 |
| *R*_int_ | 0.043 |
| (sin θ/λ)_max_ (Å^−1^) | 0.650 |
| Refinement | |
| *R*[*F*^2^ > 2σ(*F*^2^)], *wR*(*F*^2^), *S* | 0.041, 0.097, 1.03 |
| No. of reflections | 6858 |
| No. of parameters | 355 |
| No. of restraints | 274 |
| H-atom treatment | H-atoms treated by a mixture of independent and constrained refinement |
| Δρ_max_, Δρ_min_ (e Å^−3^) | 0.44, −0.30 |
| Absolute structure | Flack x determined using 2630 quotients [(I+)-(I-)]/[(I+)+(I-)] (Parsons, Flack and Wagner, Acta Cryst. B69 (2013) 249-259). |
| Absolute structure parameter | −0.01(2) |

Computer programs: Bruker Instrument Service vV6.2.3, *APEX3* v2016.5-0 (Bruker AXS), *SAINT* V8.37A (Bruker AXS Inc., 2015), XT, VERSION 2014/5, *SHELXL2014*/7 (Sheldrick, 2014), *PLATON* (Spek, 2009).

Hydrogen-bond geometry (Å, °)

| *D*—H···*A* | *D*—H | H···*A* | *D*···*A* | *D*—H···*A* |
| --- | --- | --- | --- | --- |
| N1—H1···Cl1 | 0.93(3) | 2.17(3) | 3.068(2) | 163(3) |
| N2—H2···Cl1 | 0.89(3) | 2.29(3) | 3.140(2) | 161(3) |

**Table S22.** Crystal data and structure refinement for **22**.

| Crystal data | |
| --- | --- |
| Chemical formula | C_32_H_43_N_3_O |
| *M*_r_ | 485.69 |
| Crystal system, space group | Monoclinic, *Cc* |
| Temperature (K) | 150 |
| *a*, *b*, *c* (Å) | 17.717(3), 16.691(2), 9.7428(11) |
| β (°) | 94.336(4) |
| *V* (Å^3^) | 2872.8(7) |
| *Z* | 4 |
| Radiation type | Mo*K*α |
| µ (mm^−1^) | 0.07 |
| Crystal size (mm) | 0.59 × 0.21 × 0.10 |
| Data collection | |
| Diffractometer | Bruker D8 - Venture |
| Absorption correction | Multi-scan  *SADABS2016*/2 - Bruker AXS area detector scaling and absorption correction |
| *T*_min_, *T*_max_ | 0.697, 0.746 |
| No. of measured, independent and observed [*I* > 2σ(*I*)] reflections | 38938, 6514, 4144 |
| *R*_int_ | 0.166 |
| (sin θ/λ)_max_ (Å^−1^) | 0.650 |
| Refinement | |
| *R*[*F*^2^ > 2σ(*F*^2^)], *wR*(*F*^2^), *S* | 0.065, 0.115, 1.06 |
| No. of reflections | 6514 |
| No. of parameters | 355 |
| No. of restraints | 294 |
| H-atom treatment | H-atoms treated by a mixture of independent and constrained refinement |
| Δρ_max_, Δρ_min_ (e Å^−3^) | 0.02, −0.21 |
| Absolute structure | Flack x determined using 1457 quotients [(I+)-(I-)]/[(I+)+(I-)] (Parsons, Flack and Wagner, Acta Cryst. B69 (2013) 249-259). |
| Absolute structure parameter | 0.1(9) |

Computer programs: Bruker Instrument Service vV6.2.3, *APEX3* v2016.5-0 (Bruker AXS), *SAINT* V8.37A (Bruker AXS Inc., 2015), XT, VERSION 2014/5, *SHELXL2014*/7 (Sheldrick, 2014), *PLATON* (Spek, 2009).

**Table S23.** Crystal data and structure refinement for **23**.

| Crystal data | |
| --- | --- |
| Chemical formula | C_32_H_43_N_3_O |
| *M*_r_ | 485.69 |
| Crystal system, space group | Orthorhombic, *Pbca* |
| Temperature (K) | 150 |
| *a*, *b*, *c* (Å) | 9.9843(8), 20.5796(13), 27.1817(15) |
| *V* (Å^3^) | 5585.1(6) |
| *Z* | 8 |
| Radiation type | Mo*K*α |
| µ (mm^−1^) | 0.07 |
| Crystal size (mm) | 0.26 × 0.22 × 0.12 |
| Data collection | |
| Diffractometer | Bruker D8 - Venture |
| Absorption correction | Multi-scan  *SADABS2016*/2 - Bruker AXS area detector scaling and absorption correction |
| *T*_min_, *T*_max_ | 0.687, 0.745 |
| No. of measured, independent and observed [*I* > 2σ(*I*)] reflections | 39648, 5175, 3717 |
| *R*_int_ | 0.097 |
| (sin θ/λ)_max_ (Å^−1^) | 0.606 |
| Refinement | |
| *R*[*F*^2^ > 2σ(*F*^2^)], *wR*(*F*^2^), *S* | 0.053, 0.126, 1.07 |
| No. of reflections | 5175 |
| No. of parameters | 351 |
| No. of restraints | 4 |
| H-atom treatment | H-atoms treated by a mixture of independent and constrained refinement |
| Δρ_max_, Δρ_min_ (e Å^−3^) | 0.20, −0.22 |

Computer programs: Bruker Instrument Service vV6.2.3, *APEX3* v2016.5-0 (Bruker AXS), *SAINT* V8.37A (Bruker AXS Inc., 2015), XT, VERSION 2014/5, *SHELXL2014*/7 (Sheldrick, 2014), *PLATON* (Spek, 2009).

**Table S24.** Crystal data and structure refinement for **23**^.^HCl.

| Crystal data | |
| --- | --- |
| Chemical formula | C_32_H_44_N_3_O·Cl |
| *M*_r_ | 522.15 |
| Crystal system, space group | Monoclinic, *Cc* |
| Temperature (K) | 150 |
| *a*, *b*, *c* (Å) | 10.2853(4), 22.7911(8), 13.7450(4) |
| β (°) | 101.804(1) |
| *V* (Å^3^) | 3153.87(19) |
| *Z* | 4 |
| Radiation type | Mo*K*α |
| µ (mm^−1^) | 0.15 |
| Crystal size (mm) | 0.59 × 0.38 × 0.18 |
| Data collection | |
| Diffractometer | Bruker D8 - Venture |
| Absorption correction | Multi-scan  *SADABS2016*/2 - Bruker AXS area detector scaling and absorption correction |
| *T*_min_, *T*_max_ | 0.698, 0.746 |
| No. of measured, independent and observed [*I* > 2σ(*I*)] reflections | 50911, 7172, 6273 |
| *R*_int_ | 0.060 |
| (sin θ/λ)_max_ (Å^−1^) | 0.650 |
| Refinement | |
| *R*[*F*^2^ > 2σ(*F*^2^)], *wR*(*F*^2^), *S* | 0.038, 0.092, 1.04 |
| No. of reflections | 7172 |
| No. of parameters | 351 |
| No. of restraints | 275 |
| H-atom treatment | H-atoms treated by a mixture of independent and constrained refinement |
| Δρ_max_, Δρ_min_ (e Å^−3^) | 0.18, −0.21 |
| Absolute structure | Flack x determined using 2663 quotients [(I+)-(I-)]/[(I+)+(I-)] (Parsons, Flack and Wagner, Acta Cryst. B69 (2013) 249-259). |
| Absolute structure parameter | 0.010(16) |

Computer programs: Bruker Instrument Service vV6.2.3, *APEX3* v2016.5-0 (Bruker AXS), *SAINT* V8.37A (Bruker AXS Inc., 2015), XT, VERSION 2014/5, *SHELXL2014*/7 (Sheldrick, 2014), *PLATON* (Spek, 2009).

Hydrogen-bond geometry (Å, °)

| *D*—H···*A* | *D*—H | H···*A* | *D*···*A* | *D*—H···*A* |
| --- | --- | --- | --- | --- |
| N1—H1···Cl1^i^ |  | 2.33(3) | 3.138(2) | 161(2) |
| N2—H2···Cl1^i^ | 0.93(3) | 2.23(3) | 3.130(2) | 162(2) |
| N3—H3···Cl1 | 0.88 | 2.25 | 3.0938(19) | 160 |

Symmetry code: (i) x, −y+1, z−1/2

**Table S25.** Crystal data and structure refinement for **24**.

| Crystal data | |
| --- | --- |
| Chemical formula | C_31_H_40_FN_3_ |
| *M*_r_ | 473.66 |
| Crystal system, space group | Orthorhombic, *Pna*2_1_ |
| Temperature (K) | 150 |
| *a*, *b*, *c* (Å) | 15.4103(9), 10.4739(7), 17.7507(11) |
| *V* (Å^3^) | 2865.1(3) |
| *Z* | 4 |
| Radiation type | Mo*K*α |
| µ (mm^−1^) | 0.07 |
| Crystal size (mm) | 0.59 × 0.44 × 0.37 |
| Data collection | |
| Diffractometer | Bruker D8 - Venture |
| Absorption correction | Multi-scan  *SADABS2016*/2 - Bruker AXS area detector scaling and absorption correction |
| *T*_min_, *T*_max_ | 0.679, 0.746 |
| No. of measured, independent and observed [*I* > 2σ(*I*)] reflections | 28907, 7622, 6200 |
| *R*_int_ | 0.039 |
| (sin θ/λ)_max_ (Å^−1^) | 0.696 |
| Refinement | |
| *R*[*F*^2^ > 2σ(*F*^2^)], *wR*(*F*^2^), *S* | 0.052, 0.123, 1.06 |
| No. of reflections | 7622 |
| No. of parameters | 359 |
| No. of restraints | 335 |
| H-atom treatment | H-atoms treated by a mixture of independent and constrained refinement |
| Δρ_max_, Δρ_min_ (e Å^−3^) | 0.20, −0.25 |
| Absolute structure | Flack x determined using 2381 quotients [(I+)-(I-)]/[(I+)+(I-)] (Parsons, Flack and Wagner, Acta Cryst. B69 (2013) 249-259). |
| Absolute structure parameter | 0.5(3) |

Computer programs: Bruker Instrument Service vV6.2.3, *APEX3* v2016.5-0 (Bruker AXS), *SAINT* V8.37A (Bruker AXS Inc., 2015), XT, VERSION 2014/5, *SHELXL2014*/7 (Sheldrick, 2014), *PLATON* (Spek, 2009).

Hydrogen-bond geometry (Å, °)

| *D*—H···*A* | *D*—H | H···*A* | *D*···*A* | *D*—H···*A* |
| --- | --- | --- | --- | --- |
| N2—H2···F1a^i^ | 0.88 | 2.10 | 2.874(6) | 147 |
| N2—H2···F1*^A^*b^i^ | 0.88 | 2.04 | 2.816(9) | 146 |

Symmetry code: (i) x, y−1, z

**Table S26.** Crystal data and structure refinement for **25**.

| Crystal data | |
| --- | --- |
| Chemical formula | C_31_H_40_N_4_O_2_ |
| *M*_r_ | 500.67 |
| Crystal system, space group | Triclinic, *P*-1 |
| Temperature (K) | 150 |
| *a*, *b*, *c* (Å) | 8.8345(2), 11.0889(2), 15.8031(3) |
| α, β, γ (°) | 101.605(1), 102.594(1), 106.472(1) |
| *V* (Å^3^) | 1390.51(5) |
| *Z* | 2 |
| Radiation type | Mo*K*α |
| µ (mm^−1^) | 0.08 |
| Crystal size (mm) | 0.32 × 0.20 × 0.18 |
| Data collection | |
| Diffractometer | Bruker D8 - Venture |
| Absorption correction | Multi-scan  *SADABS2016*/2 - Bruker AXS area detector scaling and absorption correction |
| *T*_min_, *T*_max_ | 0.711, 0.746 |
| No. of measured, independent and observed [*I* > 2σ(*I*)] reflections | 69304, 6828, 5913 |
| *R*_int_ | 0.047 |
| (sin θ/λ)_max_ (Å^−1^) | 0.667 |
| Refinement | |
| *R*[*F*^2^ > 2σ(*F*^2^)], *wR*(*F*^2^), *S* | 0.040, 0.105, 1.05 |
| No. of reflections | 6828 |
| No. of parameters | 348 |
| No. of restraints | 282 |
| H-atom treatment | H-atoms treated by a mixture of independent and constrained refinement |
| Δρ_max_, Δρ_min_ (e Å^−3^) | 0.30, −0.26 |

Computer programs: Bruker Instrument Service vV6.2.3, *APEX4* v2022.10-0 (Bruker AXS), *SAINT* V8.37A (Bruker AXS Inc., 2015), XT, VERSION 2014/5, *SHELXL2019*/1 (Sheldrick, 2019), *PLATON* (Spek, 2009).

Hydrogen-bond geometry (Å, °)

| *D*—H···*A* | *D*—H | H···*A* | *D*···*A* | *D*—H···*A* |
| --- | --- | --- | --- | --- |
| N3—H3···O1 | 0.903(14) | 1.908(14) | 2.6237(11) | 134.8(12) |

**Table S27.** Crystal data and structure refinement for **26**.

| Crystal data | |
| --- | --- |
| Chemical formula | C_31_H_40_N_4_O_2_ |
| *M*_r_ | 500.67 |
| Crystal system, space group | Orthorhombic, *Pbca* |
| Temperature (K) | 150 |
| *a*, *b*, *c* (Å) | 9.9301(4), 20.3878(7), 27.3938(10) |
| *V* (Å^3^) | 5546.0(4) |
| *Z* | 8 |
| Radiation type | Mo*K*α |
| µ (mm^−1^) | 0.08 |
| Crystal size (mm) | 0.59 × 0.59 × 0.24 |
| Data collection | |
| Diffractometer | Bruker D8 - Venture |
| Absorption correction | Multi-scan  *SADABS2016*/2 - Bruker AXS area detector scaling and absorption correction |
| *T*_min_, *T*_max_ | 0.692, 0.746 |
| No. of measured, independent and observed [*I* > 2σ(*I*)] reflections | 53339, 8681, 4774 |
| *R*_int_ | 0.105 |
| (sin θ/λ)_max_ (Å^−1^) | 0.750 |
| Refinement | |
| *R*[*F*^2^ > 2σ(*F*^2^)], *wR*(*F*^2^), *S* | 0.077, 0.148, 1.05 |
| No. of reflections | 8681 |
| No. of parameters | 350 |
| No. of restraints | 282 |
| H-atom treatment | H-atoms treated by a mixture of independent and constrained refinement |
| Δρ_max_, Δρ_min_ (e Å^−3^) | 0.24, −0.32 |

Computer programs: Bruker Instrument Service vV6.2.3, *APEX3* v2016.5-0 (Bruker AXS), *SAINT* V8.37A (Bruker AXS Inc., 2015), XT, VERSION 2014/5, *SHELXL2014*/7 (Sheldrick, 2014), *PLATON* (Spek, 2009).

Hydrogen-bond geometry (Å, °)

| *D*—H···*A* | *D*—H | H···*A* | *D*···*A* | *D*—H···*A* |
| --- | --- | --- | --- | --- |
| N3—H3···O1^i^ | 0.82(2) | 2.65(2) | 3.201(2) | 126.5(17) |
| N3—H3···O2^i^ | 0.82(2) | 2.63(2) | 3.403(2) | 158.3(19) |

Symmetry code: (i) x+1/2, −y+3/2, −z+1

**Table S28.** Crystal data and structure refinement for **27**.

| Crystal data | |
| --- | --- |
| Chemical formula | C_31_H_40_N_4_O_2_ |
| *M*_r_ | 500.67 |
| Crystal system, space group | Monoclinic, *P*2_1_/*n* |
| Temperature (K) | 150 |
| *a*, *b*, *c* (Å) | 16.8907(15), 9.4712(7), 18.7409(14) |
| β (°) | 108.122(3) |
| *V* (Å^3^) | 2849.4(4) |
| *Z* | 4 |
| Radiation type | Mo*K*α |
| µ (mm^−1^) | 0.07 |
| Crystal size (mm) | 0.59 × 0.59 × 0.53 |
| Data collection | |
| Diffractometer | Bruker D8 - Venture |
| Absorption correction | Multi-scan  *SADABS2016*/2 - Bruker AXS area detector scaling and absorption correction |
| *T*_min_, *T*_max_ | 0.665, 0.746 |
| No. of measured, independent and observed [*I* > 2σ(*I*)] reflections | 79727, 6564, 4523 |
| *R*_int_ | 0.119 |
| (sin θ/λ)_max_ (Å^−1^) | 0.652 |
| Refinement | |
| *R*[*F*^2^ > 2σ(*F*^2^)], *wR*(*F*^2^), *S* | 0.067, 0.167, 1.08 |
| No. of reflections | 6564 |
| No. of parameters | 350 |
| No. of restraints | 282 |
| H-atom treatment | H-atoms treated by a mixture of independent and constrained refinement |
| Δρ_max_, Δρ_min_ (e Å^−3^) | 0.62, −0.39 |

Computer programs: Bruker Instrument Service vV6.2.3, *APEX3* v2016.5-0 (Bruker AXS), *SAINT* V8.37A (Bruker AXS Inc., 2015), XT, VERSION 2014/5, *SHELXL2014*/7 (Sheldrick, 2014), *PLATON* (Spek, 2009).

Hydrogen-bond geometry (Å, °)

| *D*—H···*A* | *D*—H | H···*A* | *D*···*A* | *D*—H···*A* |
| --- | --- | --- | --- | --- |
| N2—H2···O2^i^ | 0.87(2) | 2.32(2) | 2.931(2) | 127.7(18) |

Symmetry code: (i) x+1/2, −y+3/2, z+1/2

**Table S29.** Crystal data and structure refinement for **28**.

| Crystal data | |
| --- | --- |
| Chemical formula |  |
| *M*_r_ | 373.48 |
| Crystal system, space group | Hexagonal, *P*6_1_ |
| Temperature (K) | 150 |
| *a*, *c* (Å) | 21.6174(8), 7.9213(4) |
| *V* (Å^3^) | 3205.8(3) |
| *Z* | 6 |
| Radiation type | Mo*K*α |
| µ (mm^−1^) | 0.07 |
| Crystal size (mm) | 0.59 × 0.15 × 0.10 |
| Data collection | |
| Diffractometer | Bruker D8 - Venture |
| Absorption correction | Multi-scan  *SADABS2016*/2 - Bruker AXS area detector scaling and absorption correction |
| *T*_min_, *T*_max_ | 0.681, 0.746 |
| No. of measured, independent and observed [*I* > 2σ(*I*)] reflections | 32352, 4826, 4072 |
| *R*_int_ | 0.064 |
| (sin θ/λ)_max_ (Å^−1^) | 0.650 |
| Refinement | |
| *R*[*F*^2^ > 2σ(*F*^2^)], *wR*(*F*^2^), *S* | 0.051, 0.109, 1.16 |
| No. of reflections | 4826 |
| No. of parameters | 266 |
| No. of restraints | 214 |
| H-atom treatment | H-atoms treated by a mixture of independent and constrained refinement |
| Δρ_max_, Δρ_min_ (e Å^−3^) | 0.16, −0.22 |
| Absolute structure | Flack x determined using 1446 quotients [(I+)-(I-)]/[(I+)+(I-)] (Parsons, Flack and Wagner, Acta Cryst. B69 (2013) 249-259). |
| Absolute structure parameter | −0.1(6) |

Computer programs: *SHELXT* 2014/5 (Sheldrick, 2014), *SHELXL2017*/1 (Sheldrick, 2017).

**Table S30.** Crystal data and structure refinement for **29**.

| Crystal data | |
| --- | --- |
| Chemical formula | C_37_H_45_N_3_ |
| *M*_r_ | 531.76 |
| Crystal system, space group | Triclinic, *P-*1 |
| Temperature (K) | 150 |
| *a*, *b*, *c* (Å) | 10.6219(2), 10.7814(3), 15.1144(3) |
| α, β, γ (°) | 78.462(1), 88.123(1), 69.129(1) |
| *V* (Å^3^) | 1583.23(6) |
| *Z* | 2 |
| Radiation type | Mo*K*α |
| µ (mm^−1^) | 0.07 |
| Crystal size (mm) | 0.47 × 0.29 × 0.15 |
| Data collection | |
| Diffractometer | Bruker D8 - Venture |
| Absorption correction | Multi-scan  *SADABS2016*/2 - Bruker AXS area detector scaling and absorption correction |
| *T*_min_, *T*_max_ | 0.716, 0.746 |
| No. of measured, independent and observed [*I* > 2σ(*I*)] reflections | 73141, 7844, 6688 |
| *R*_int_ | 0.046 |
| (sin θ/λ)_max_ (Å^−1^) | 0.667 |
| Refinement | |
| *R*[*F*^2^ > 2σ(*F*^2^)], *wR*(*F*^2^), *S* | 0.046, 0.125, 1.04 |
| No. of reflections | 7844 |
| No. of parameters | 372 |
| No. of restraints | 309 |
| H-atom treatment | H atoms treated by a mixture of independent and constrained refinement |
| Δρ_max_, Δρ_min_ (e Å^−3^) | 0.28, −0.33 |

Computer programs: Bruker Instrument Service vV6.2.3, *APEX3* v2016.5-0 (Bruker AXS), *SAINT* V8.37A (Bruker AXS Inc., 2015), XT, VERSION 2014/5, *SHELXL2019*/1 (Sheldrick, 2019), *PLATON* (Spek, 2009).


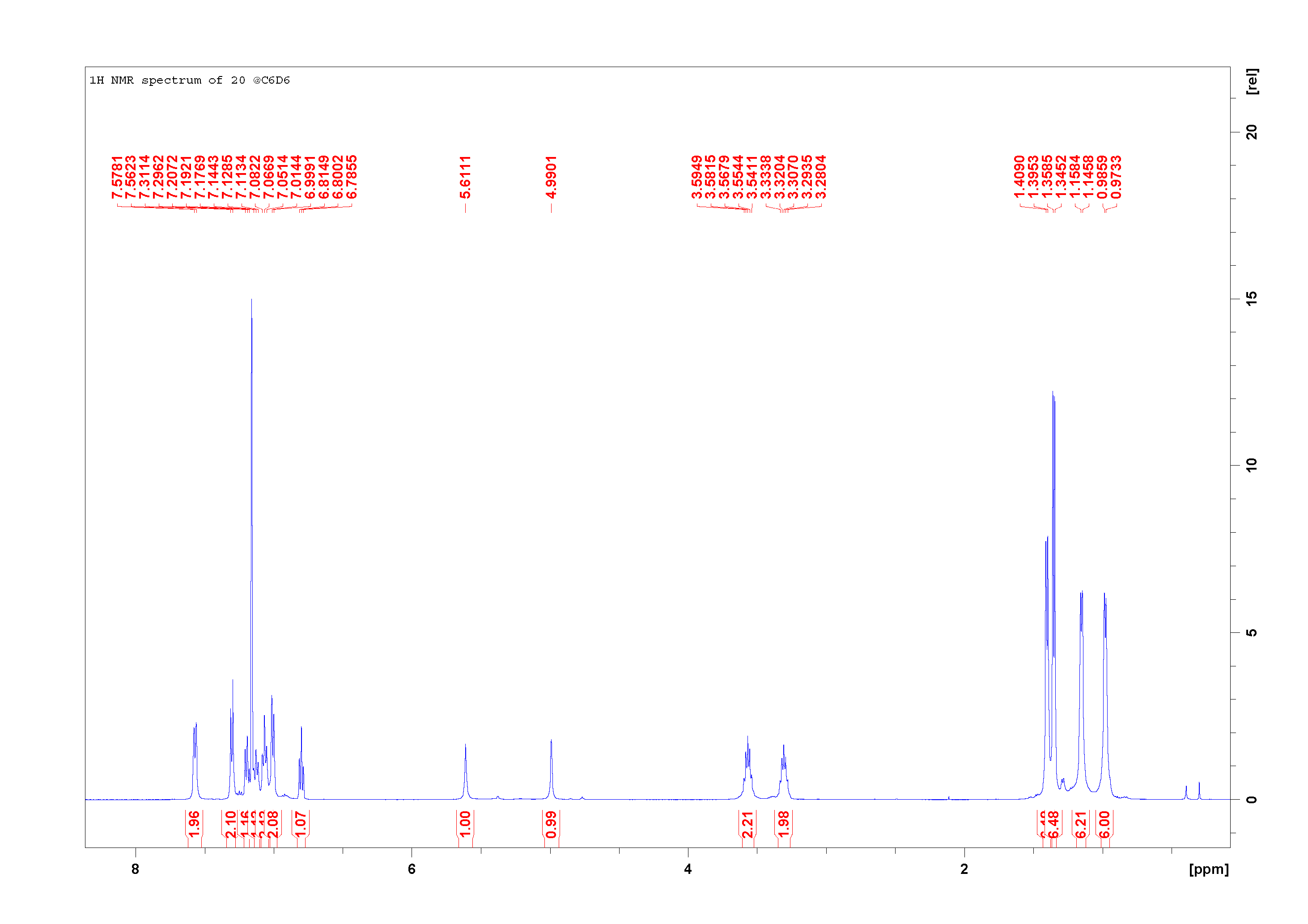


**Figure S43**. ^1^H NMR spectrum of **20** @C_6_D_6_, 295 K. Signals corresponding to the minor form are marked with black dots.


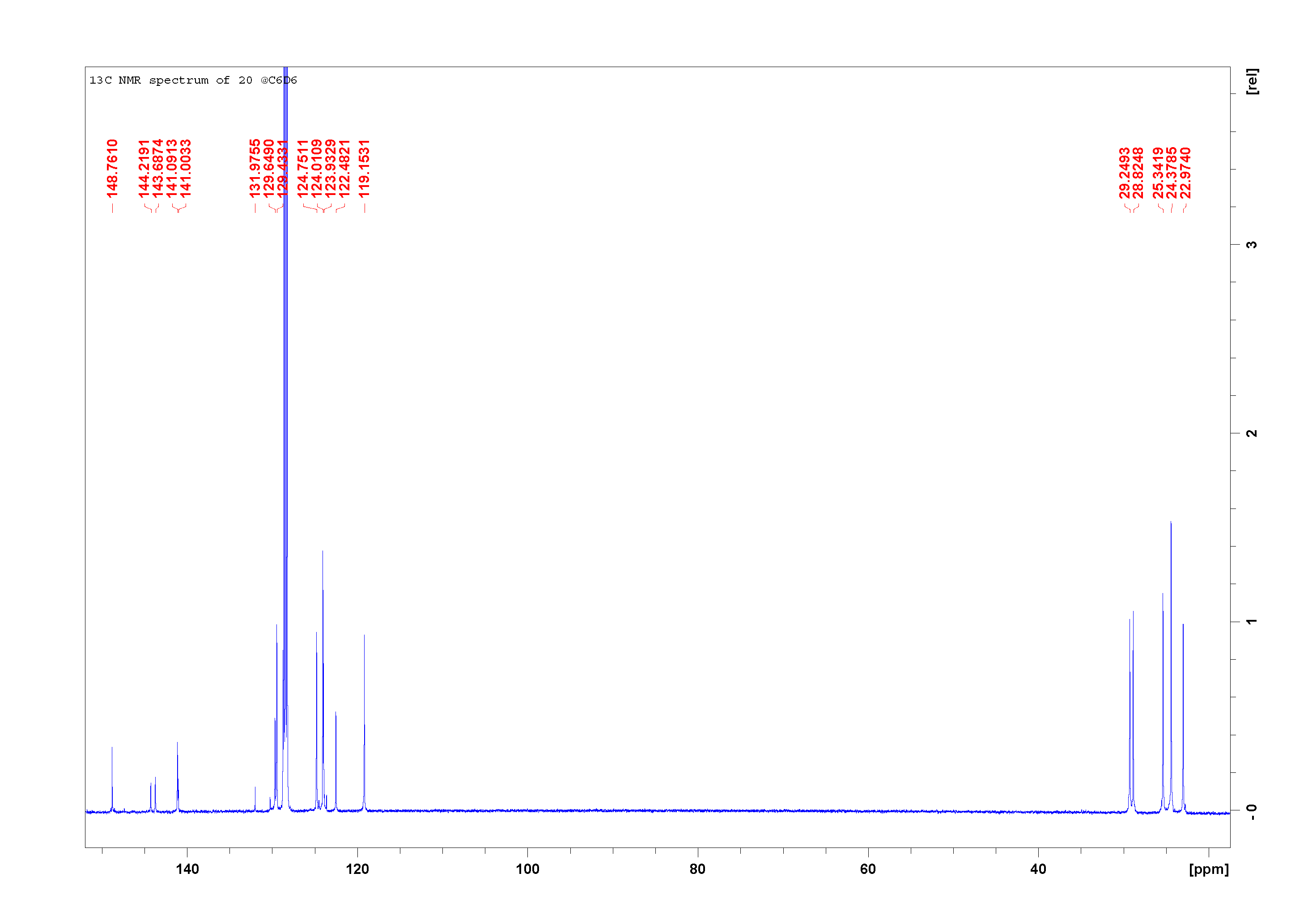


**Figure S44**. ^13^C NMR spectrum of **20** @C_6_D_6_, 295 K. Signals corresponding to the minor form are marked with black dots.


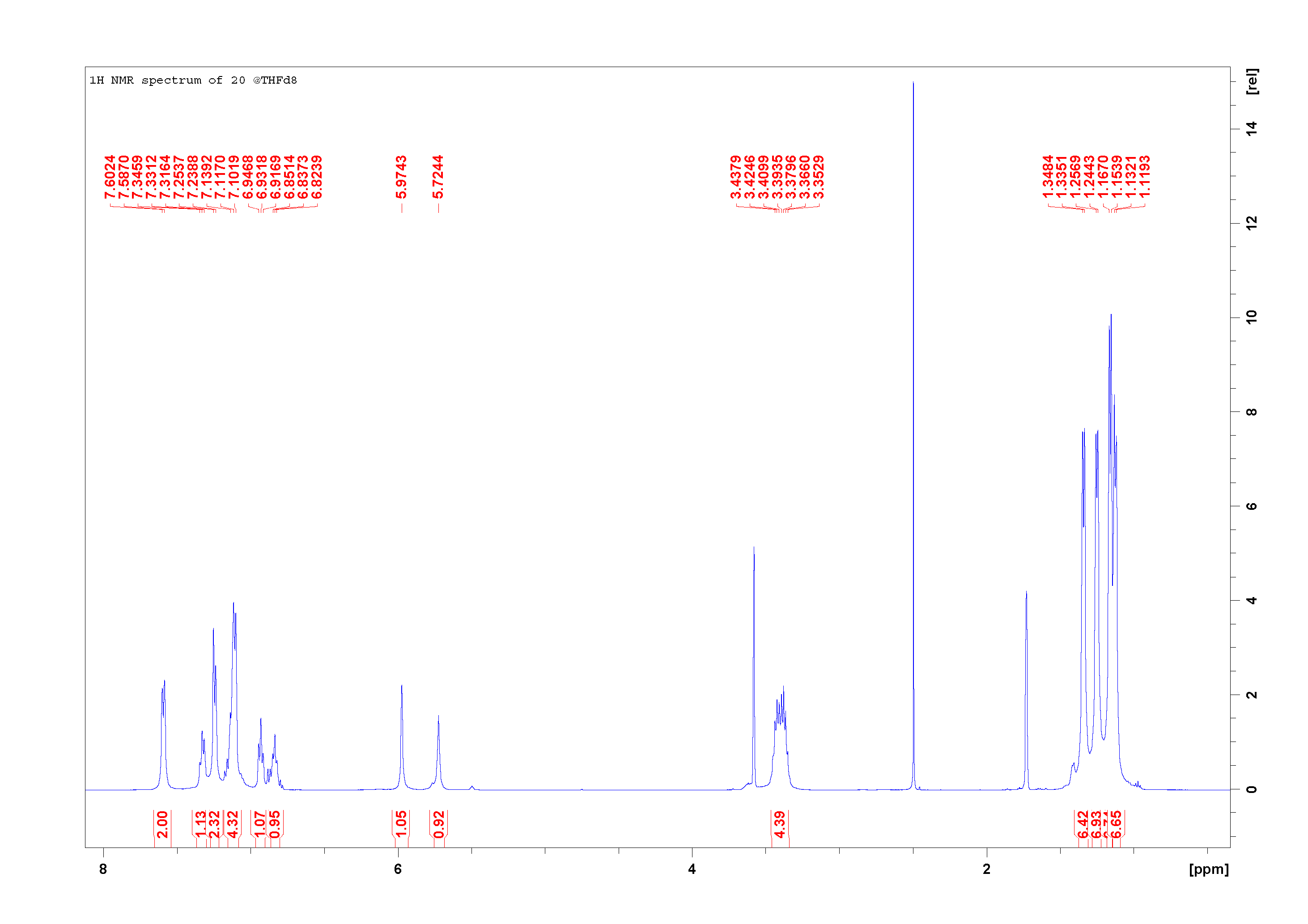


**Figure S45**. ^1^H NMR spectrum of **20** @THF-d_8_, 295 K. Signals corresponding to the minor form are marked with black dots.


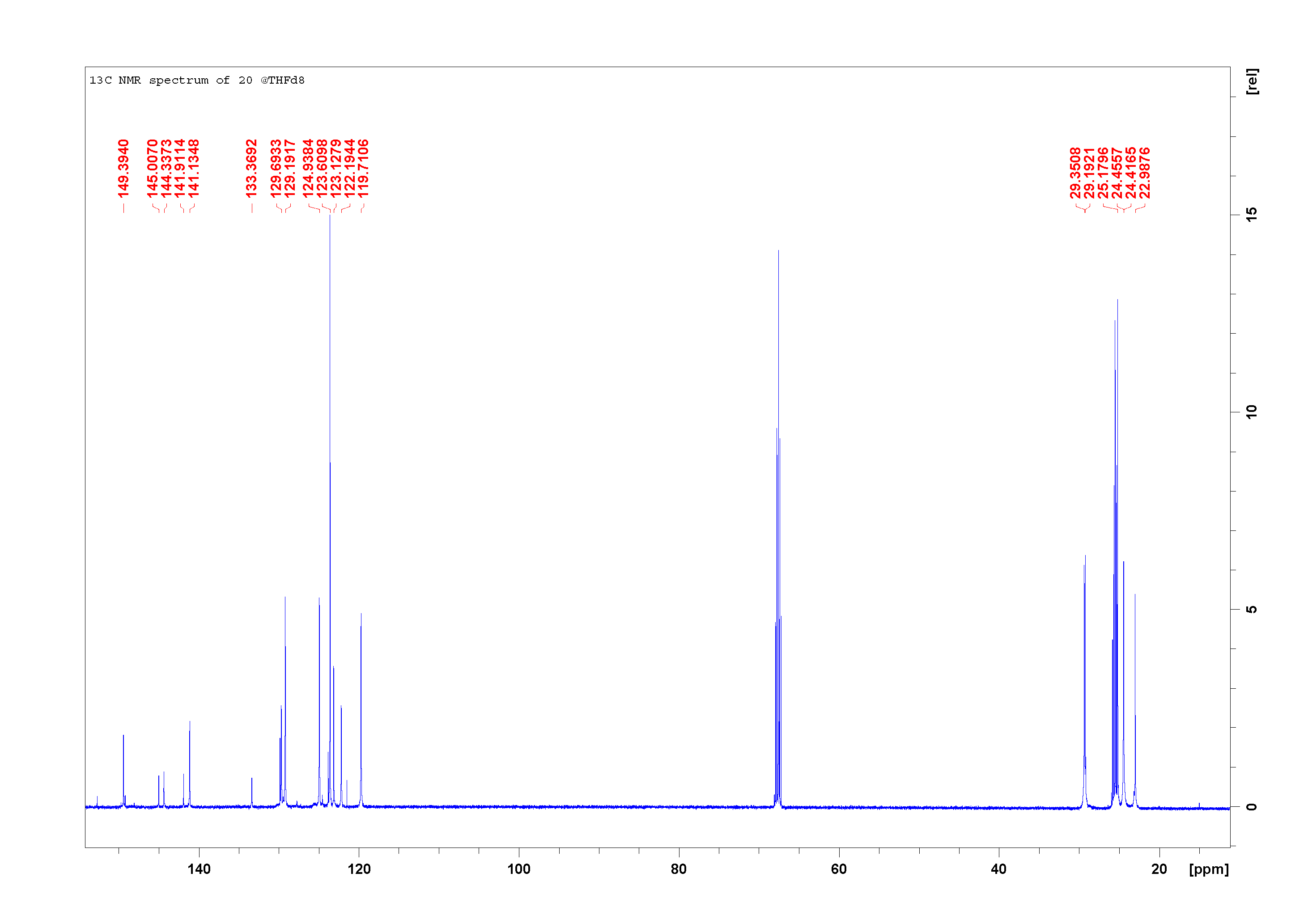


**Figure S46**. ^13^C NMR spectrum of **20** @THF-d_8_, 295 K. Signals corresponding to the minor form are marked with black dots.


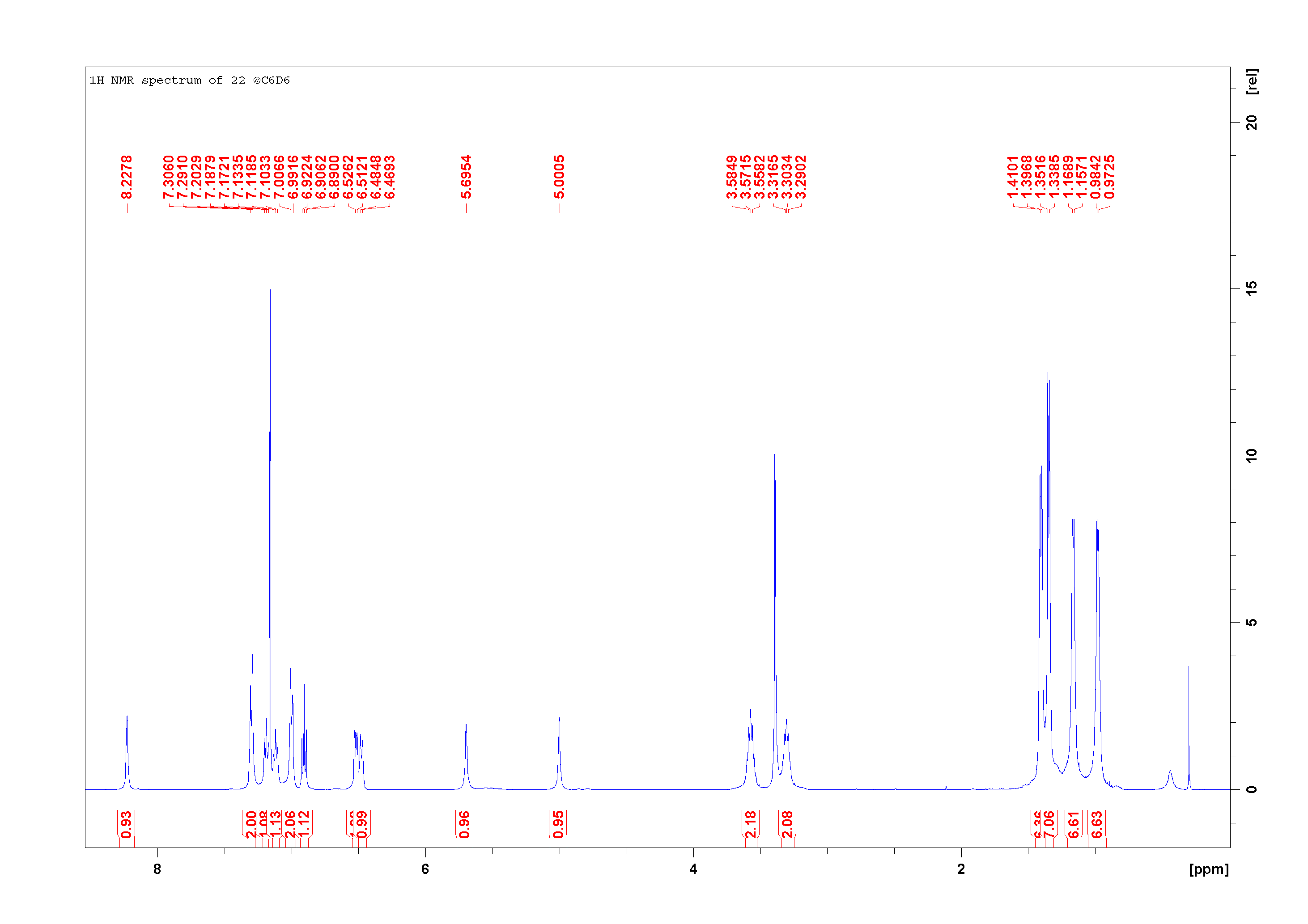


**Figure S47**. ^1^H NMR spectrum of **22** @C_6_D_6_, 295 K. Signals corresponding to the minor form are marked with black dots.


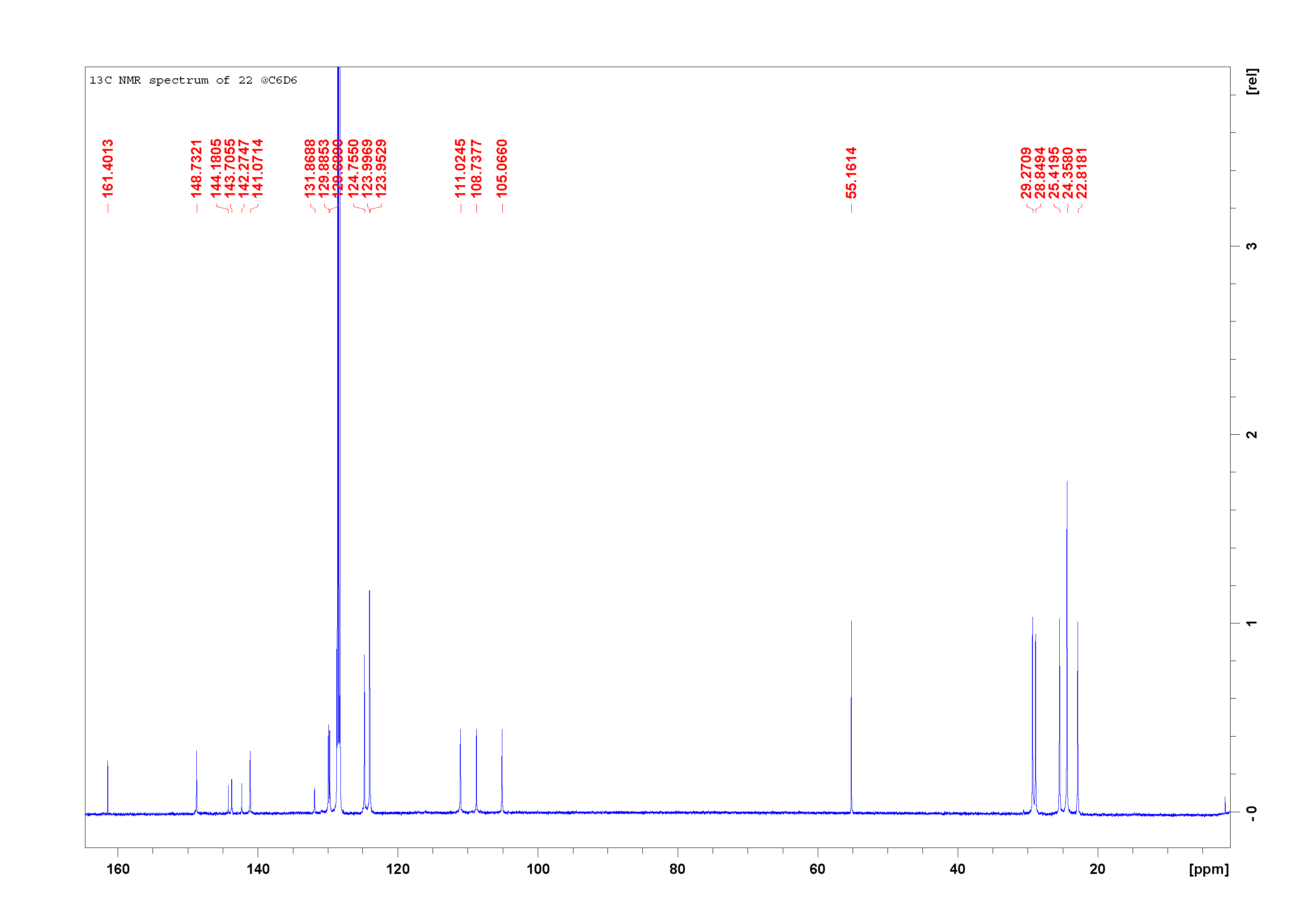


**Figure S48**. ^13^C NMR spectrum of **22** @C_6_D_6_, 295 K. Signals corresponding to the minor form are marked with black dots.


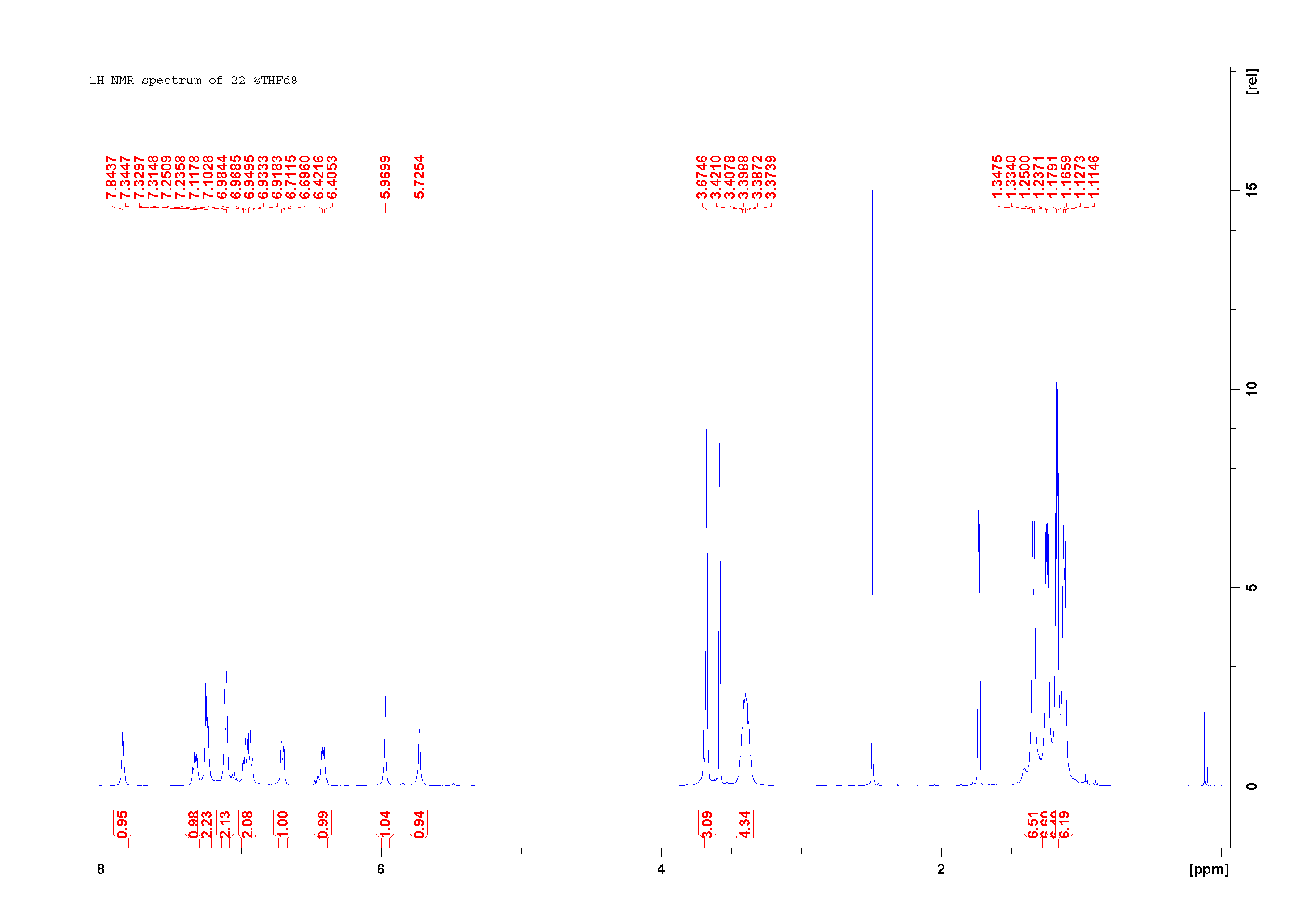


**Figure S49**. ^1^H NMR spectrum of **22** @THF-d_8_, 295 K. Signals corresponding to the minor form are marked with black dots.


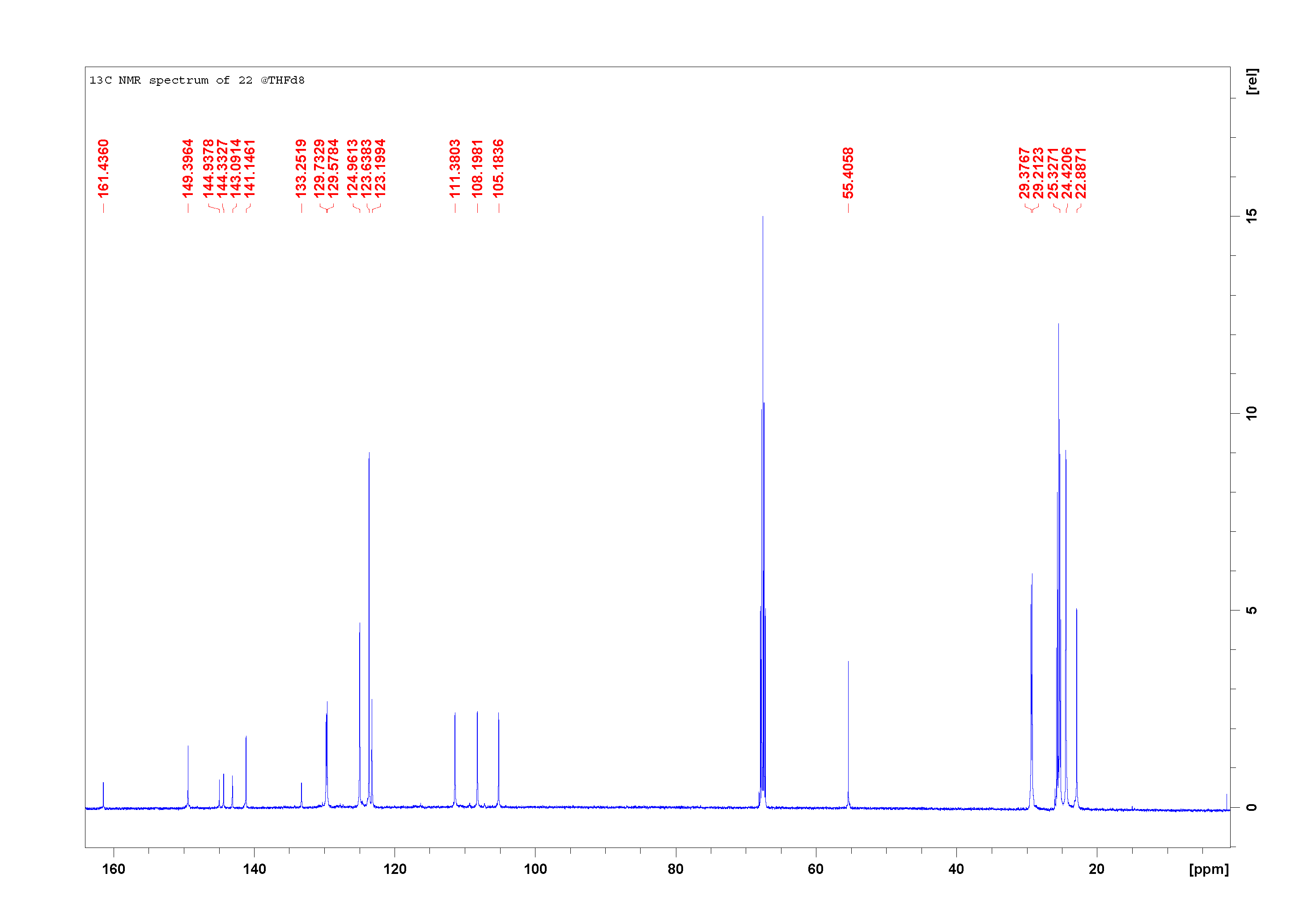


**Figure S50**. ^13^C NMR spectrum of **22** @THF-d_8_, 295 K. Signals corresponding to the minor form are marked with black dots.


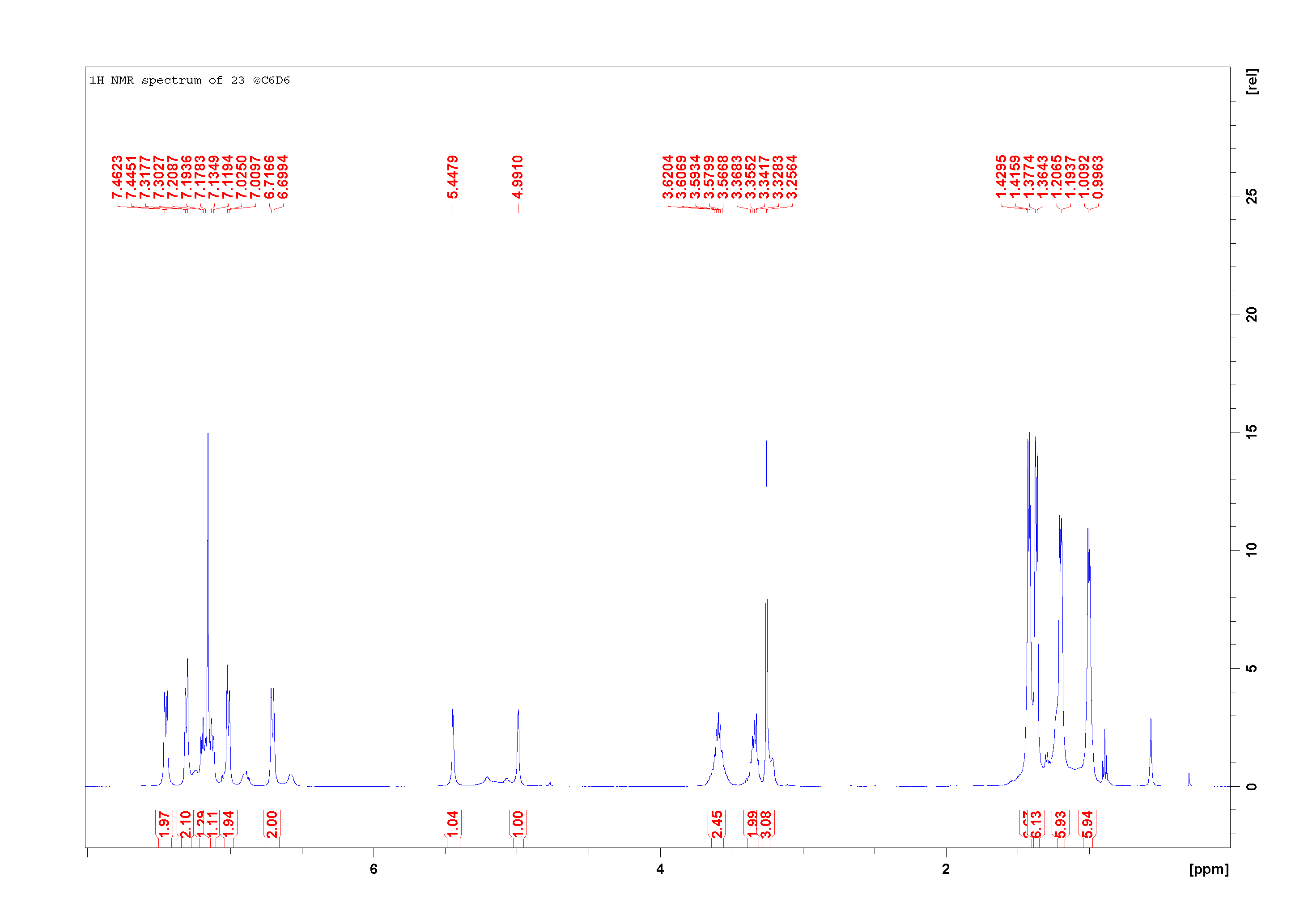


**Figure S51**. ^1^H NMR spectrum of **23** @C_6_D_6_, 295 K. Signals corresponding to the minor form are marked with black dots.


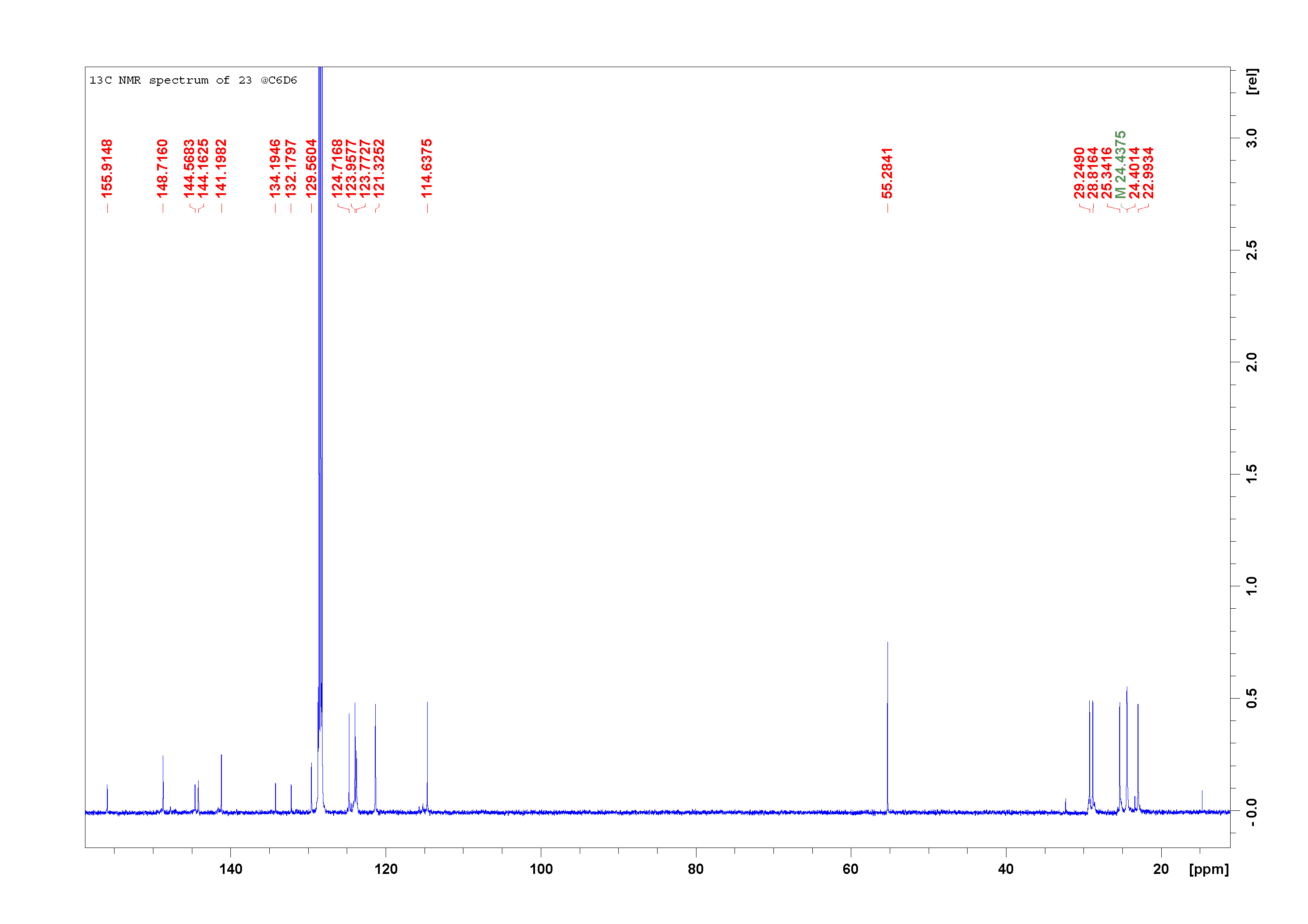


**Figure S52**. ^13^C NMR spectrum of **23** @C_6_D_6_, 295 K. Signals corresponding to the minor form are marked with black dots.


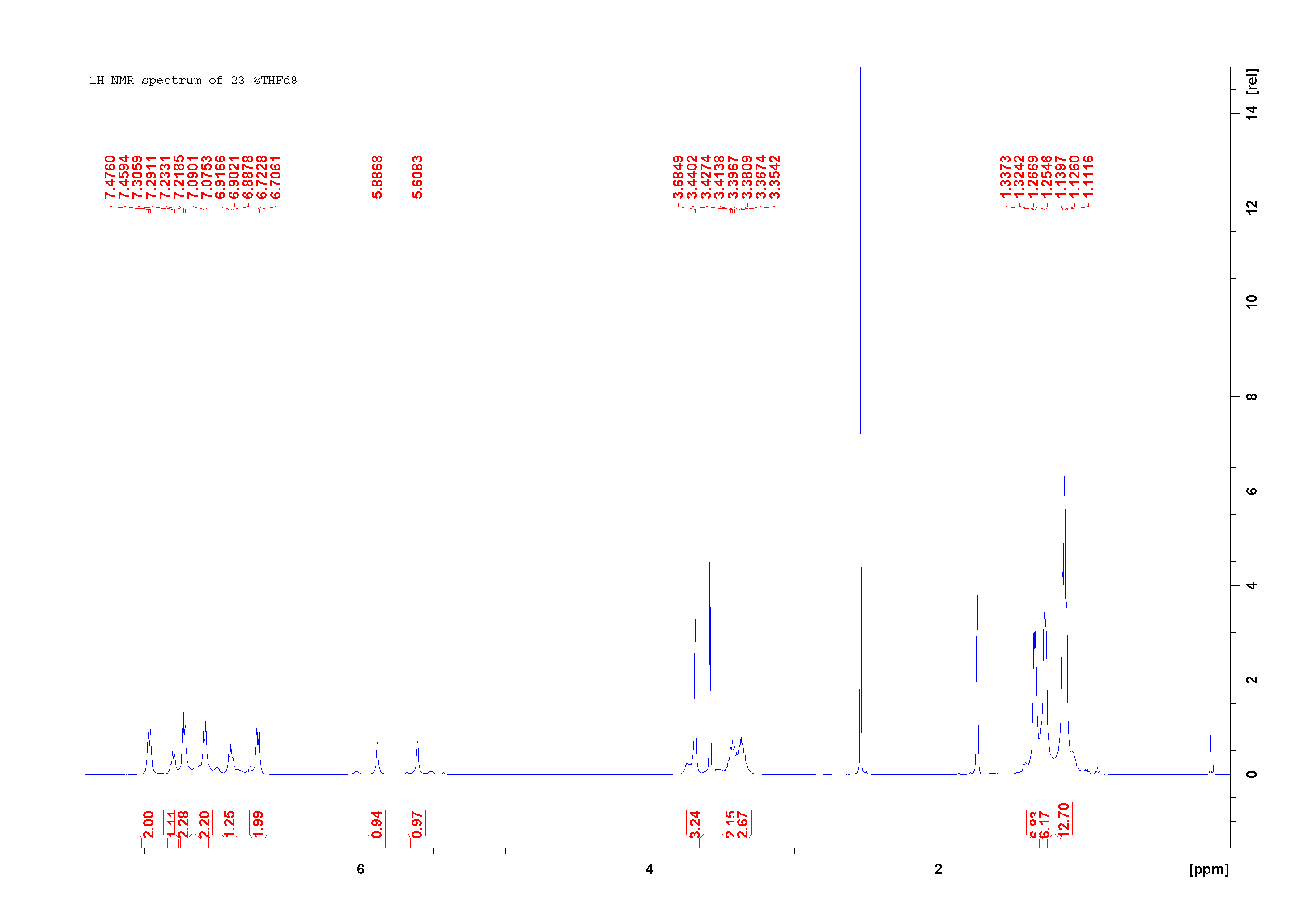


**Figure S53**. ^1^H NMR spectrum of **23** @THF-d_8_, 295 K. Signals corresponding to the minor form are marked with black dots.


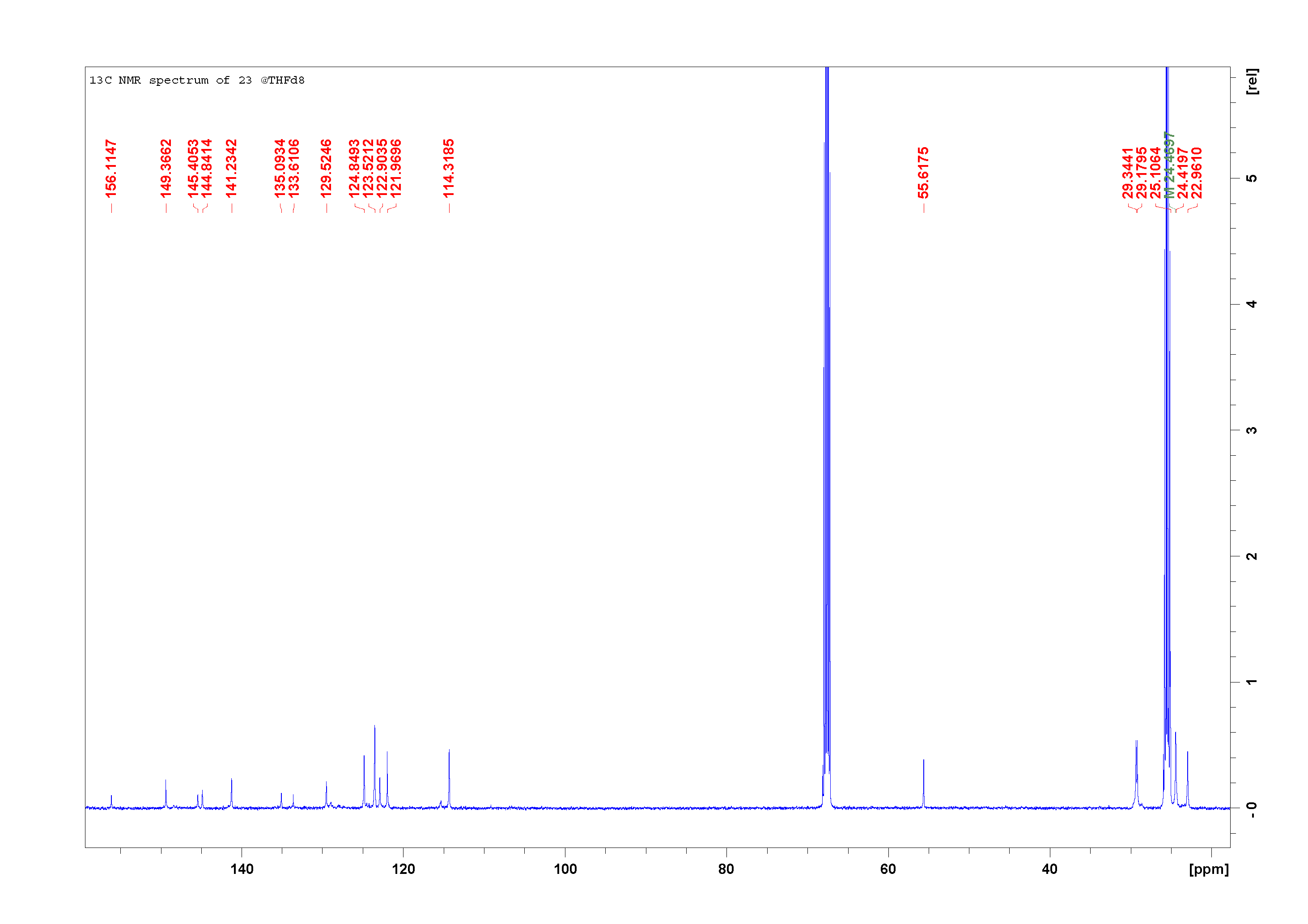


**Figure S54**. ^13^C NMR spectrum of **23** @THF-d_8_, 295 K. Signals corresponding to the minor form are marked with black dots.


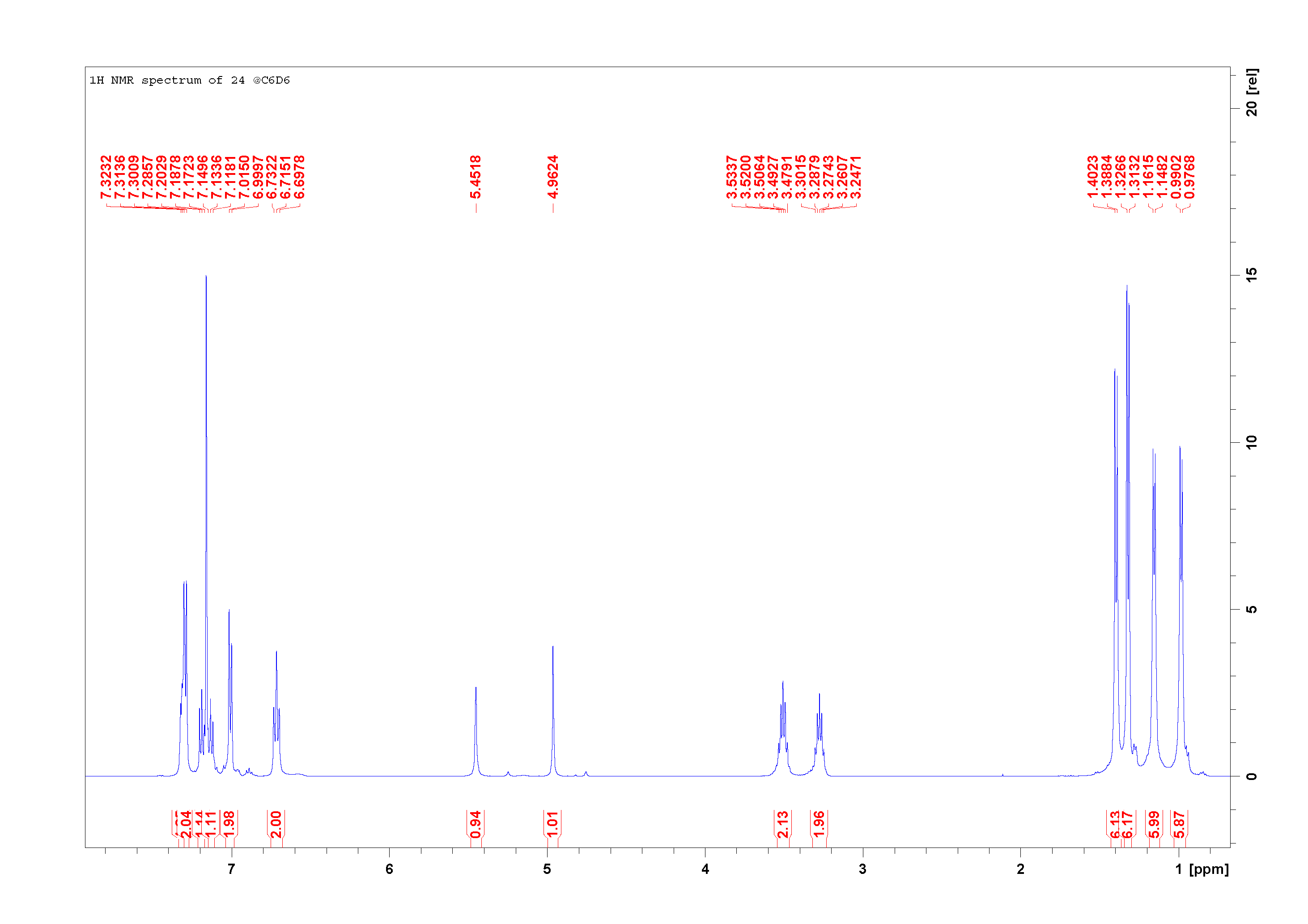


**Figure S55**. ^1^H NMR spectrum of **24** @C_6_D_6_, 295 K. Signals corresponding to the minor form are marked with black dots.


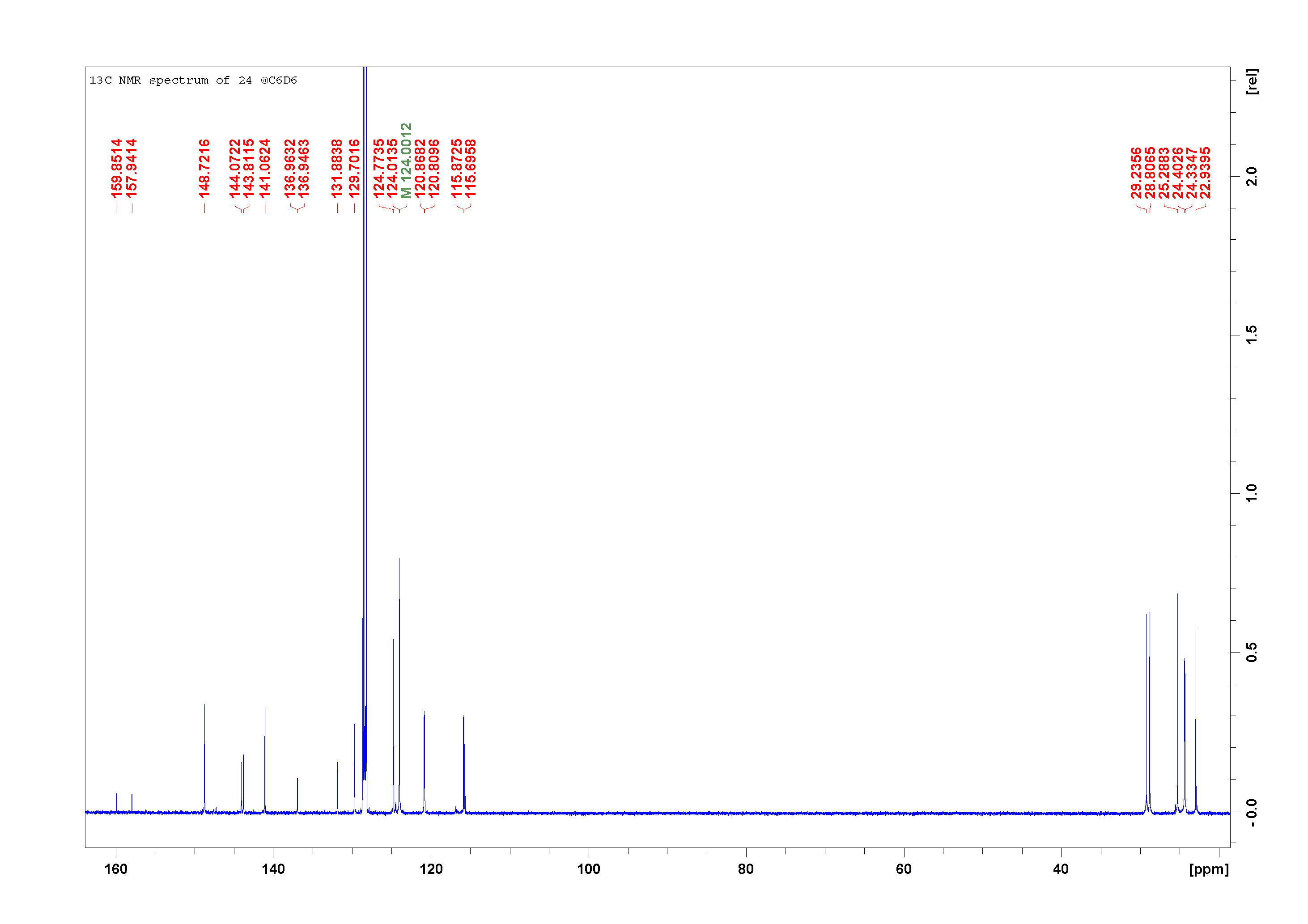


**Figure S56**. ^13^C NMR spectrum of **24** @C_6_D_6_, 295 K. Signals corresponding to the minor form are marked with black dots.


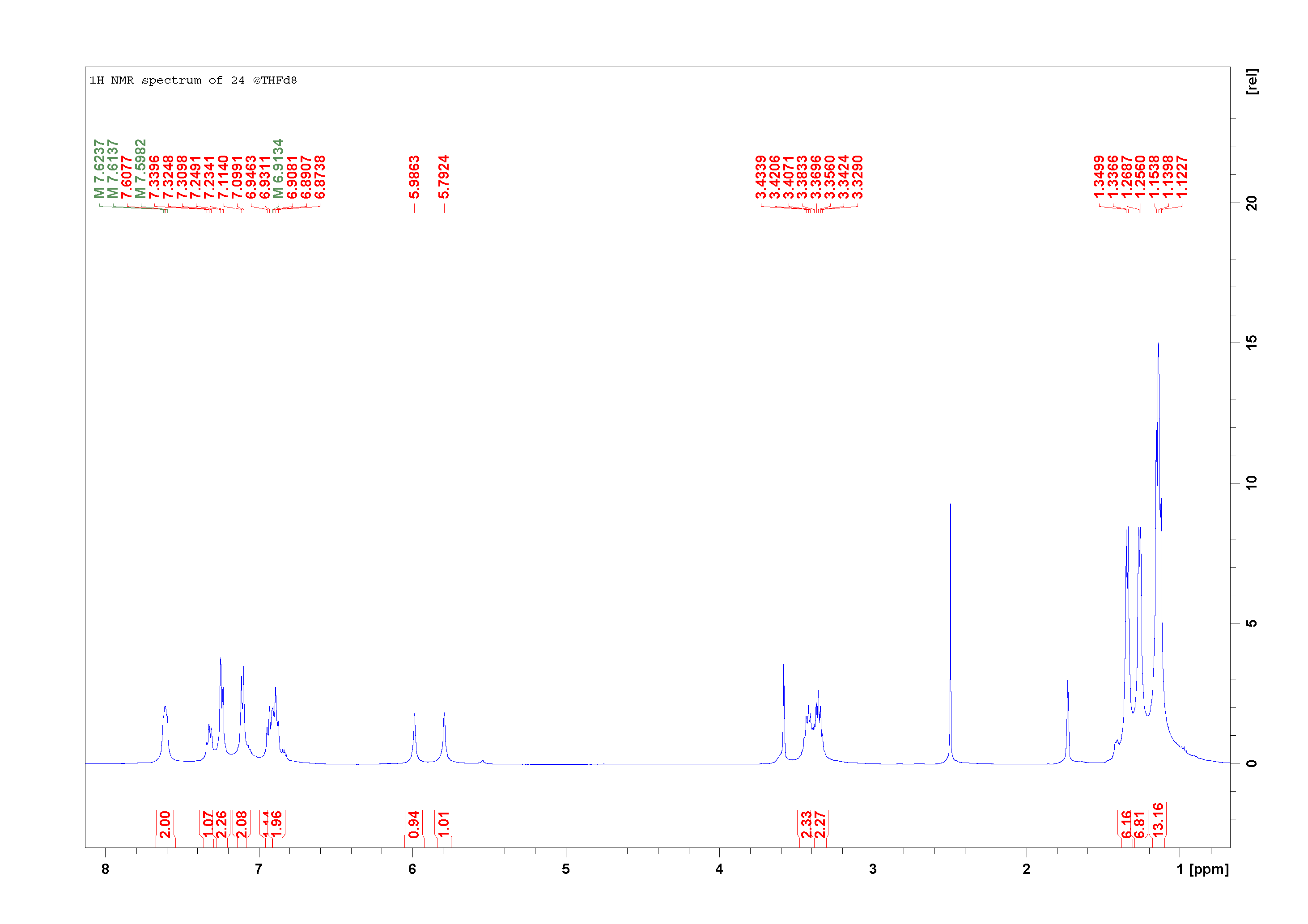


**Figure S57**. ^1^H NMR spectrum of **24** @THF-d_8_, 295 K. Signals corresponding to the minor form are marked with black dots.


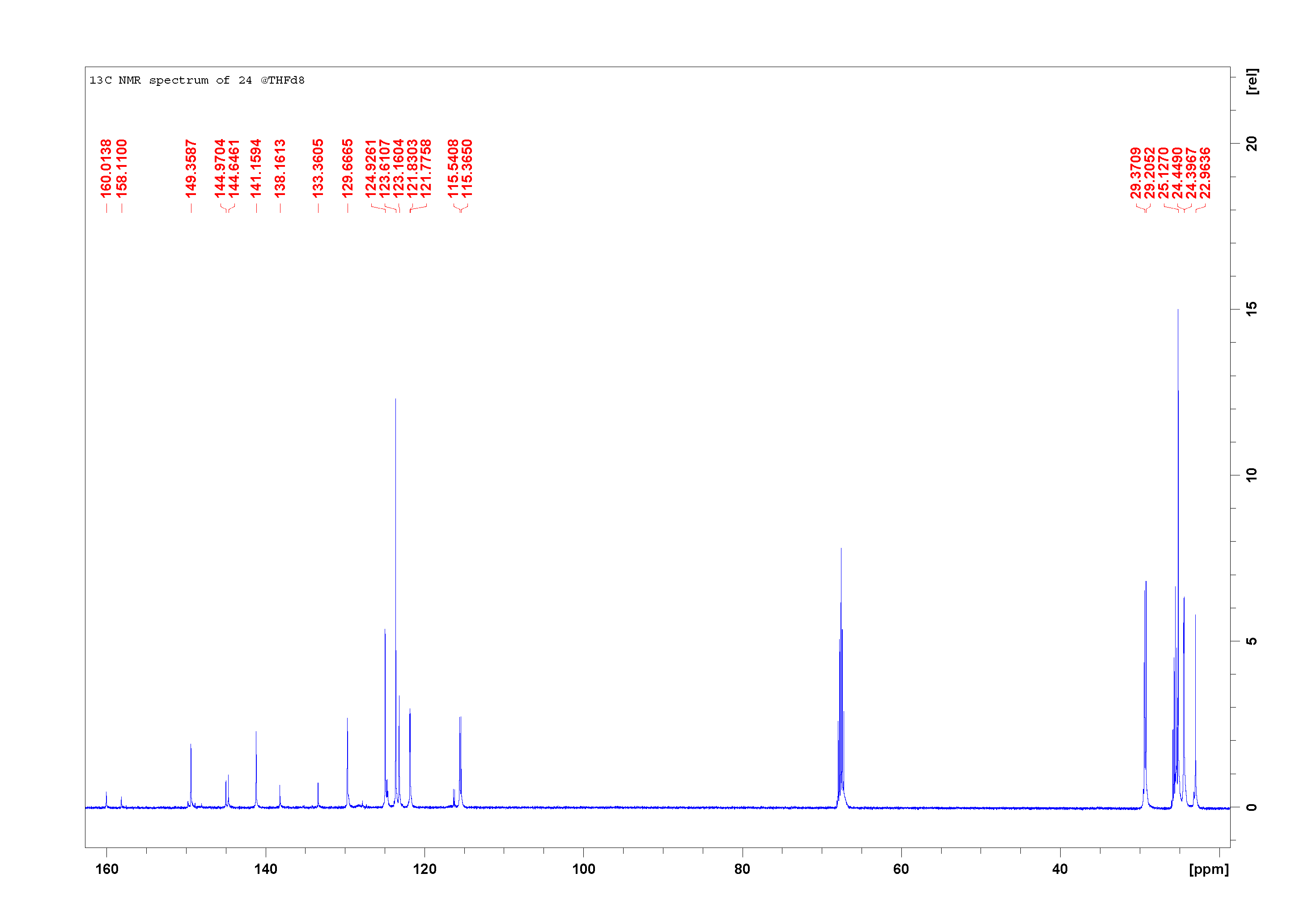


**Figure S58**. ^13^C NMR spectrum of **24** @THF-d_8_, 295 K. Signals corresponding to the minor form are marked with black dots.


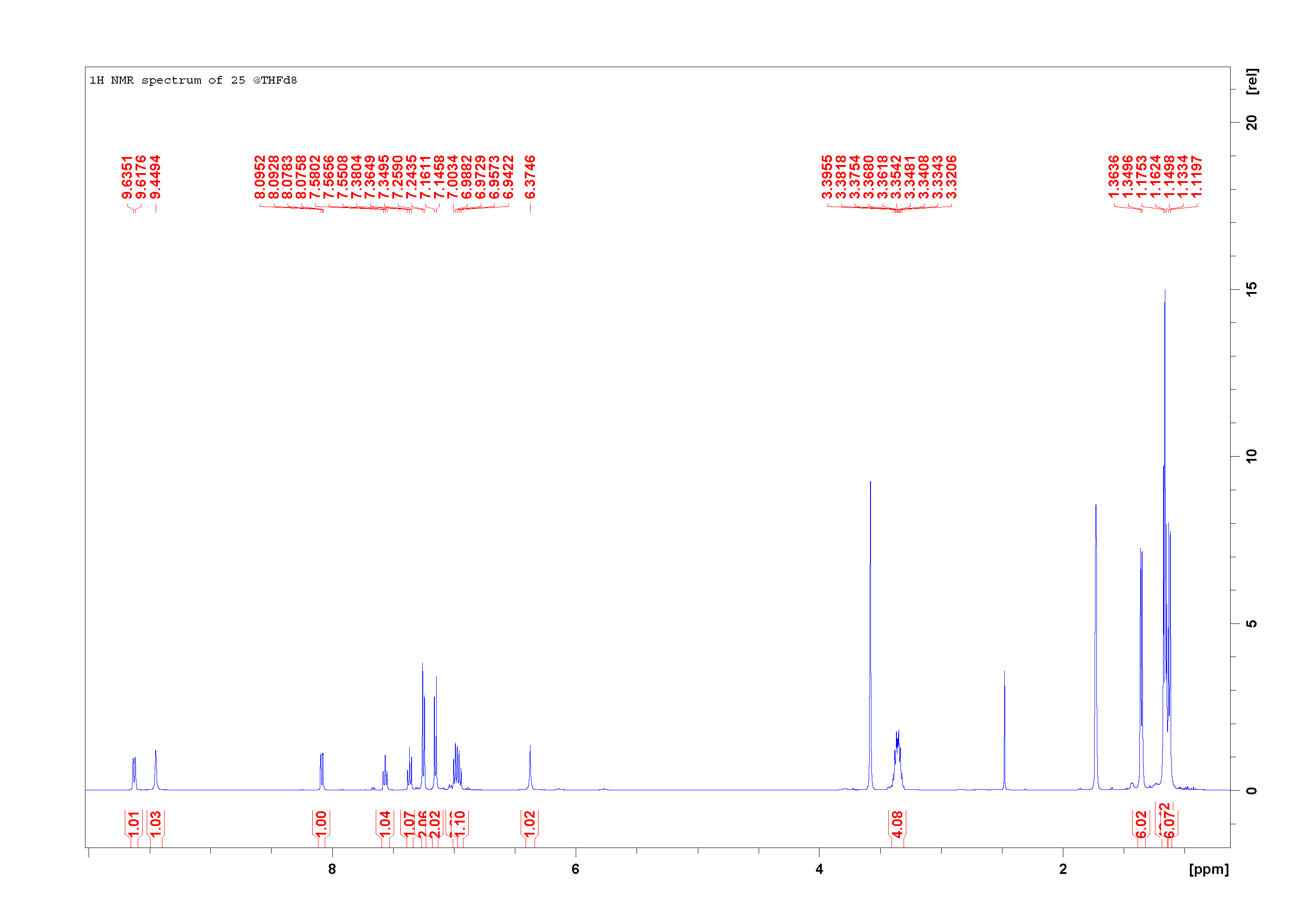


**Figure S59**. ^1^H NMR spectrum of **25** @THF-d_8_, 295 K. Signals corresponding to the minor form are marked with black dots.


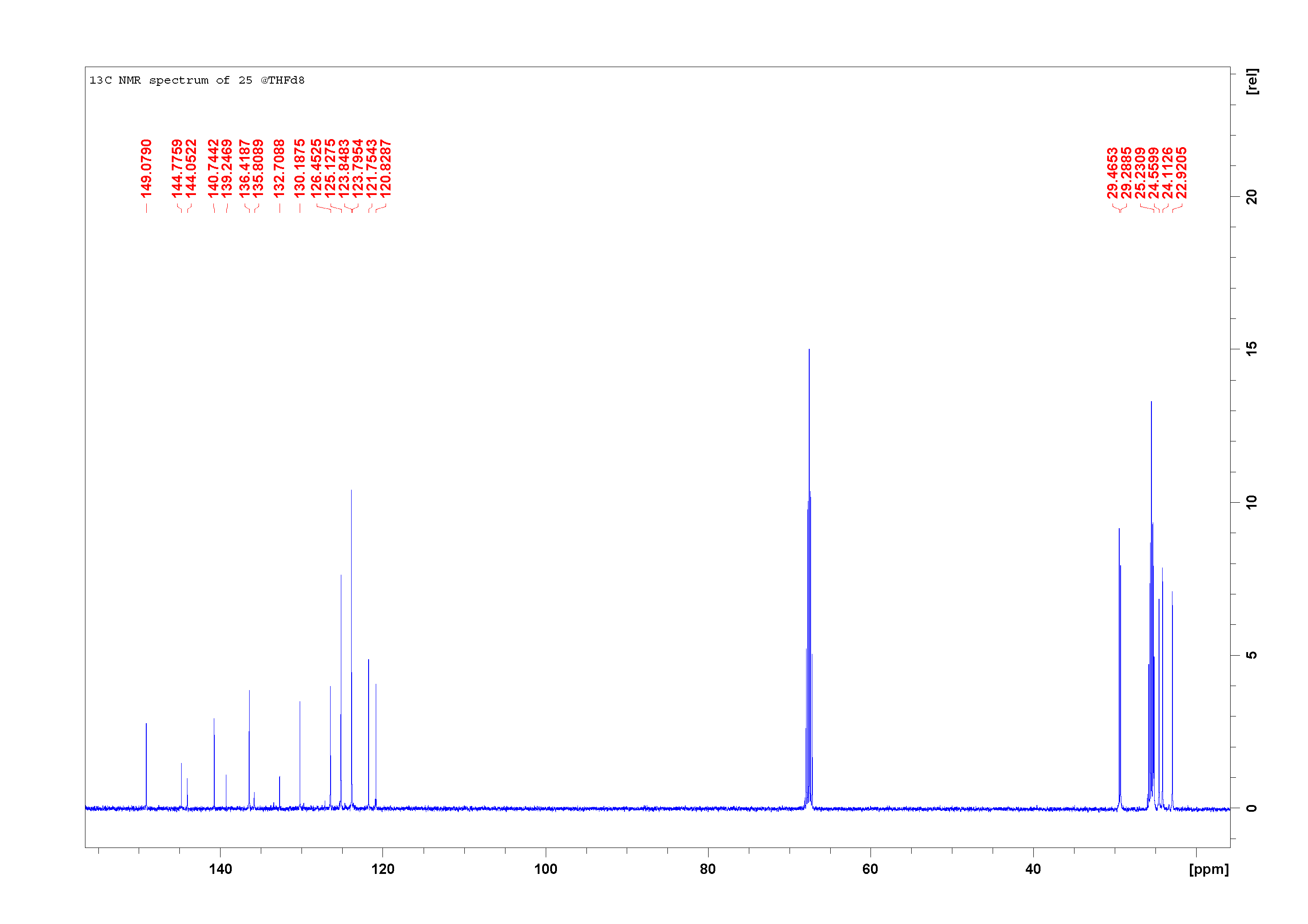


**Figure S60**. ^13^C NMR spectrum of **25** @THF-d_8_, 295 K. Signals corresponding to the minor form are marked with black dots.


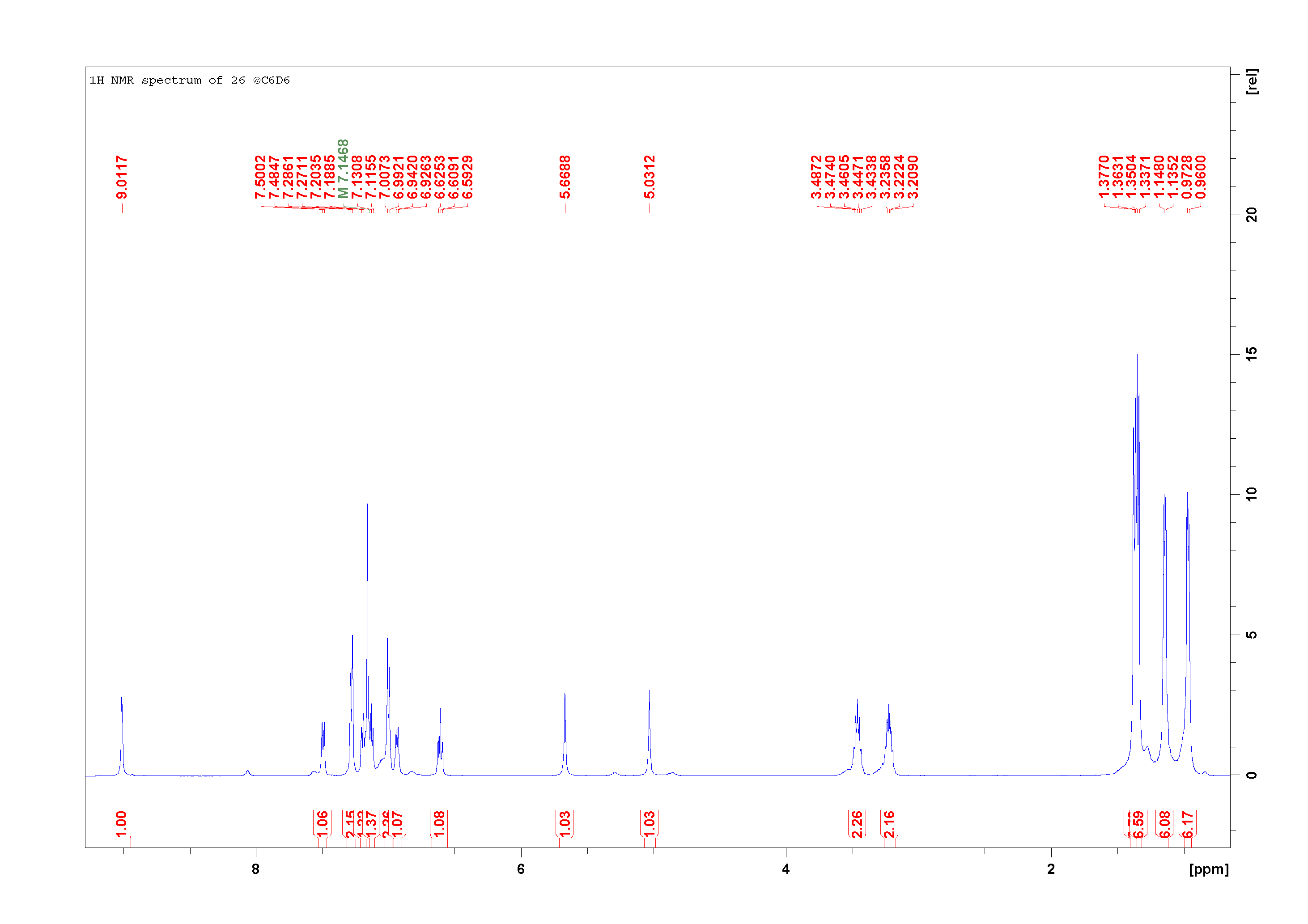


**Figure S61**. ^1^H NMR spectrum of **26** @C_6_D_6_, 295 K. Signals corresponding to the minor form are marked with black dots.


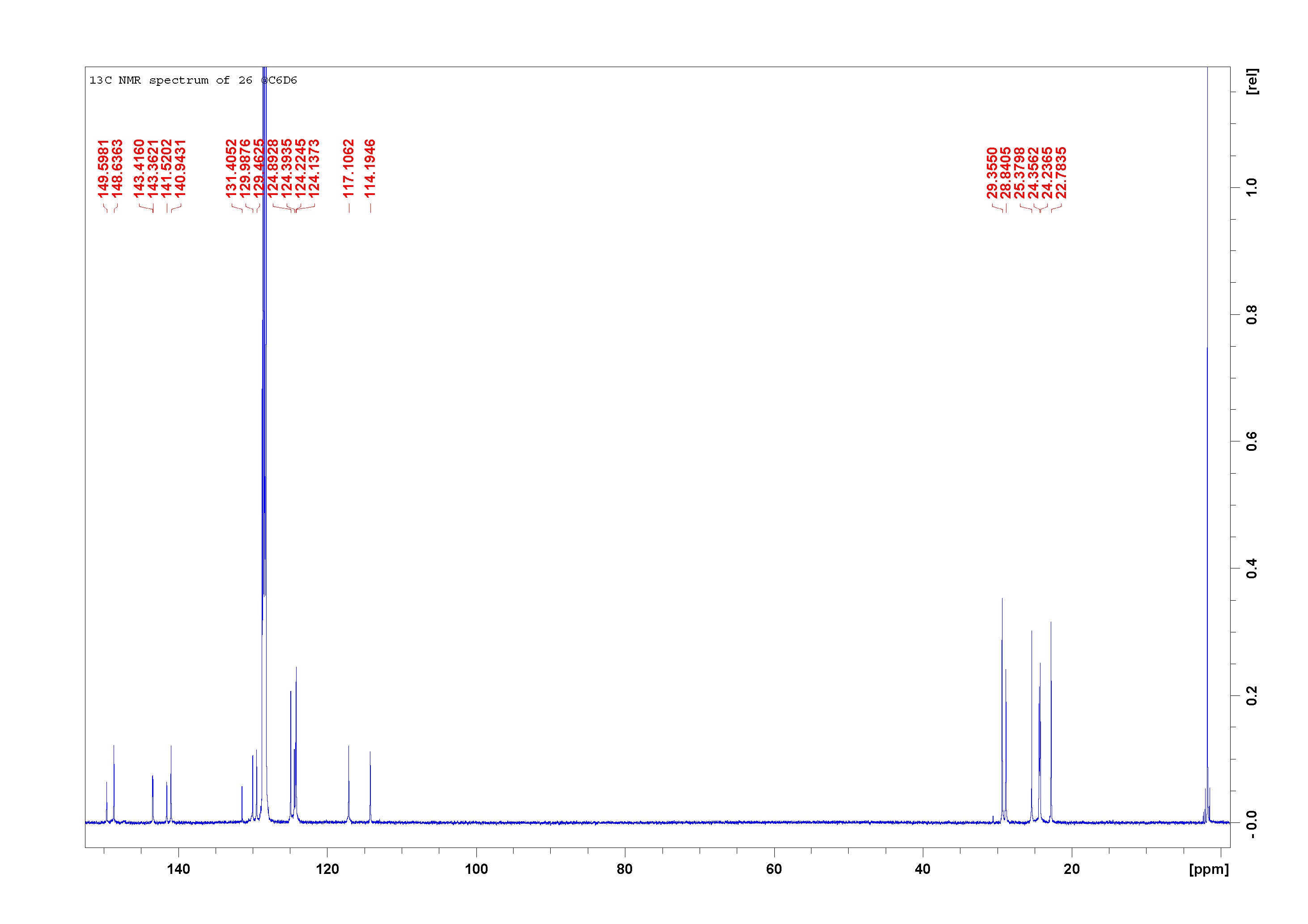


**Figure S62**. ^13^C NMR spectrum of **26** @C_6_D_6_, 295 K. Signals corresponding to the minor form are marked with black dots.


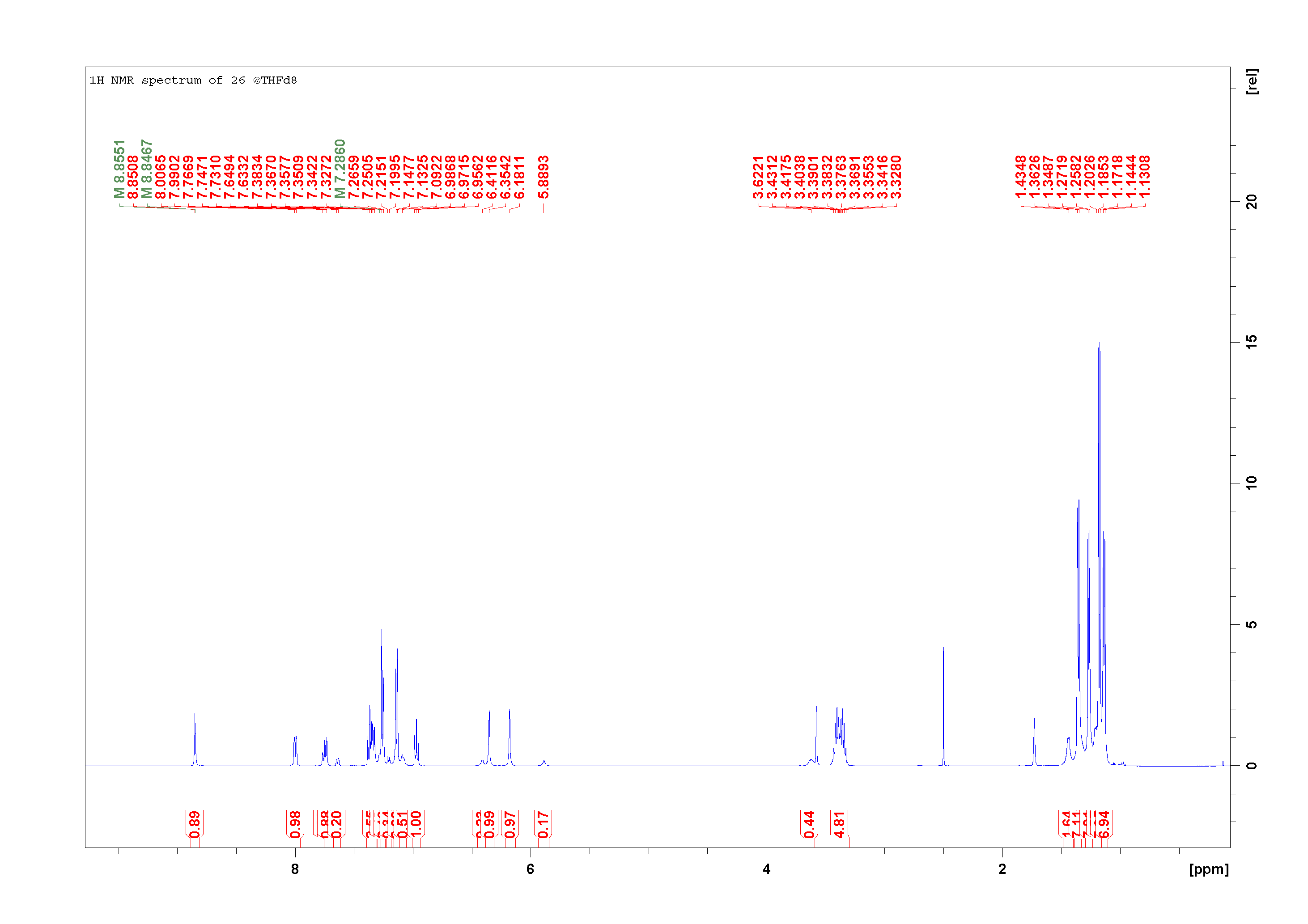


**Figure S63**. ^1^H NMR spectrum of **26** @THF-d_8_, 295 K. Signals corresponding to the minor form are marked with black dots.


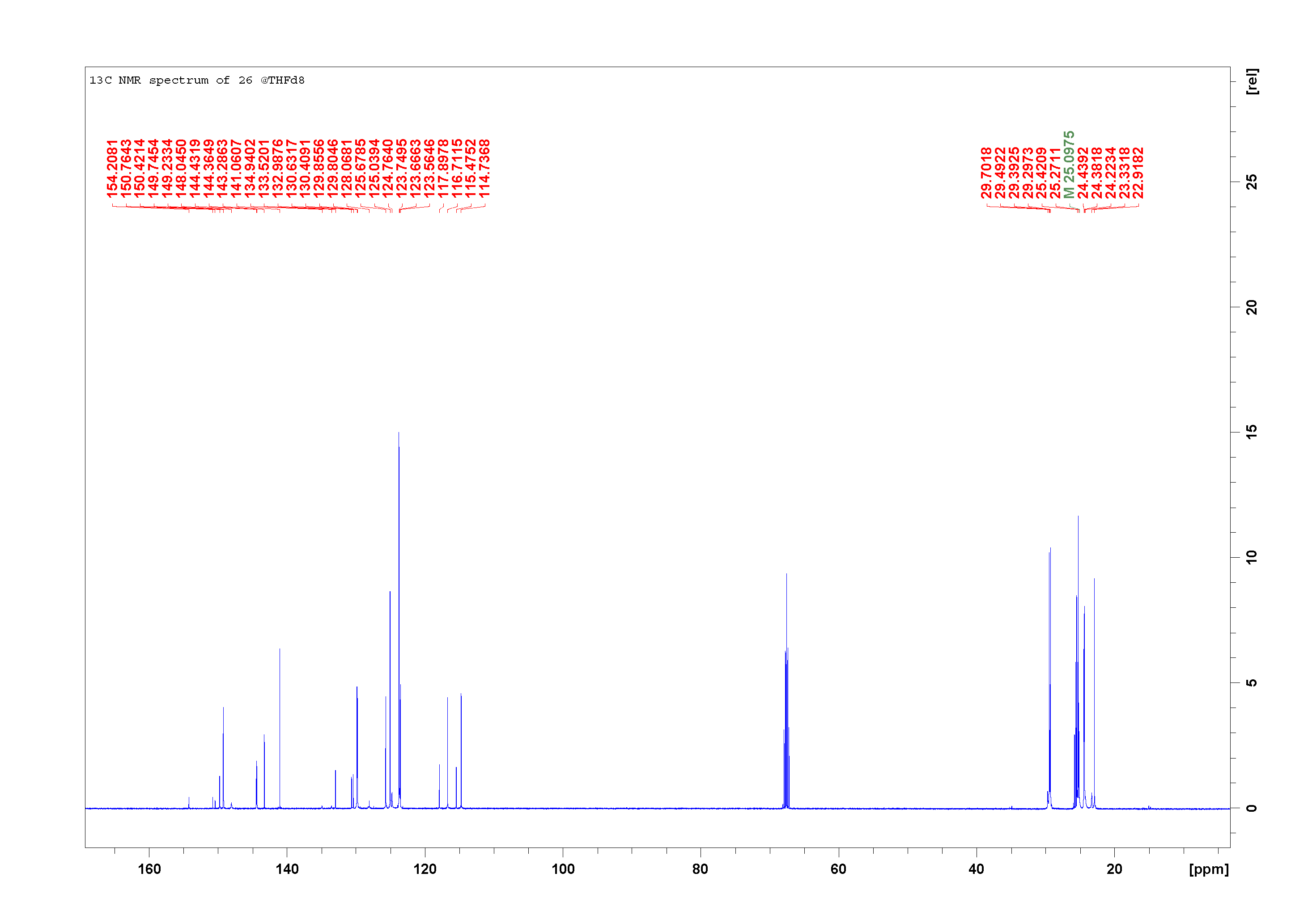


**Figure S64**. ^13^C NMR spectrum of **26** @THF-d_8_, 295 K. Signals corresponding to the minor form are marked with black dots.


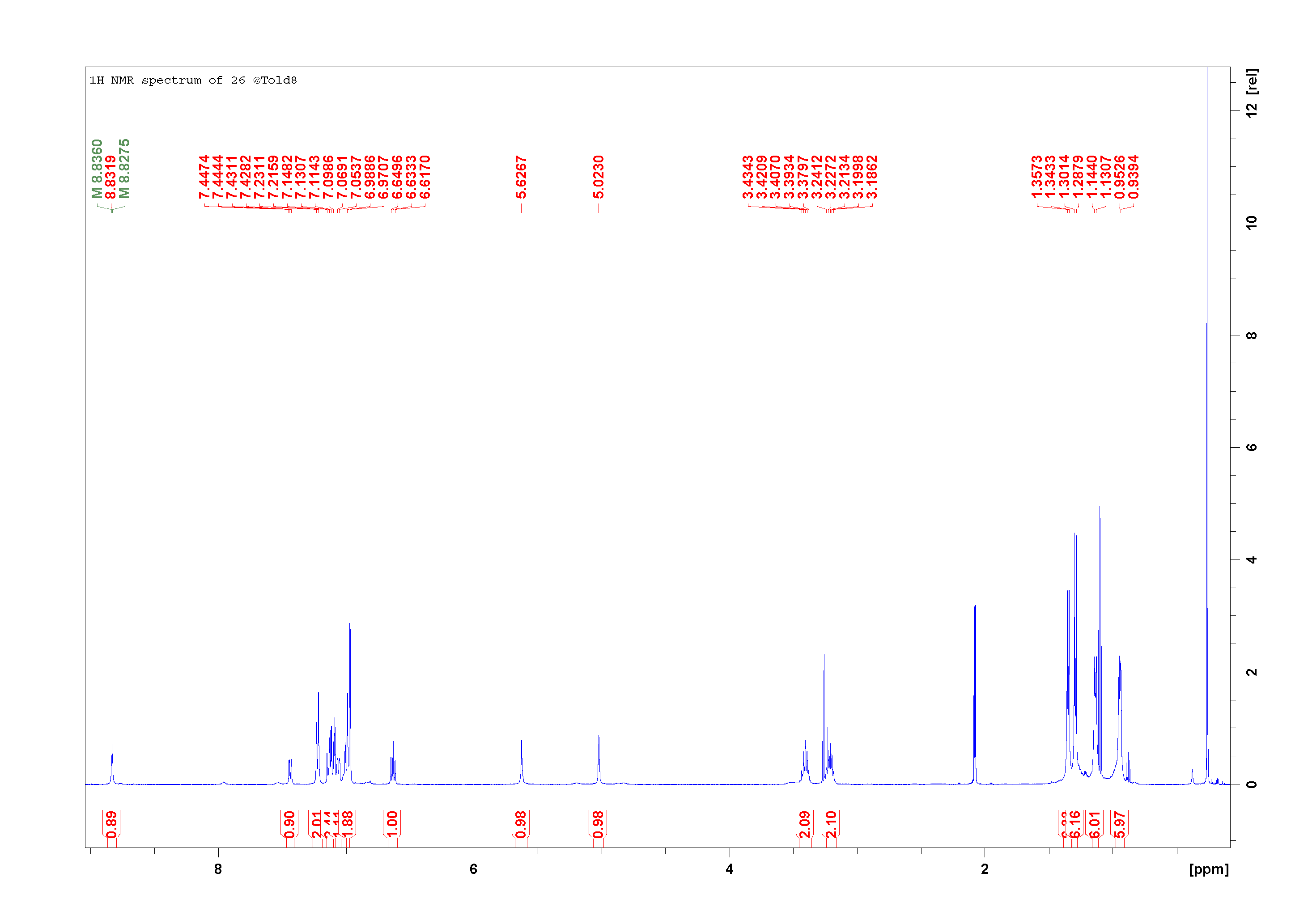


**Figure S65**. ^1^H NMR spectrum of **26** @Tol-d_8_, 295 K. Signals corresponding to the minor form are marked with black dots.


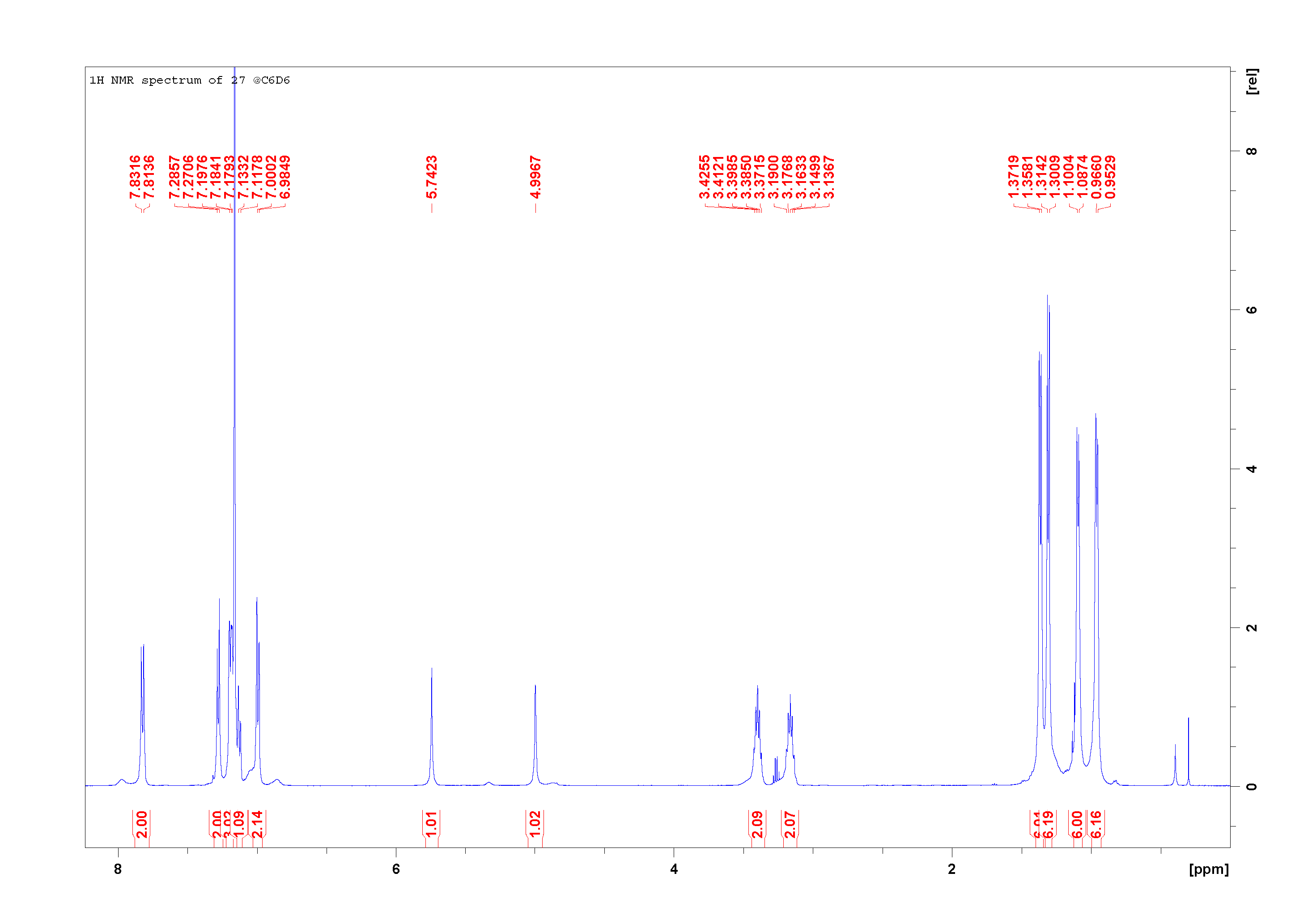


**Figure S66**. ^1^H NMR spectrum of **27** @C_6_D_6_, 295 K. Signals corresponding to the minor form are marked with black dots.


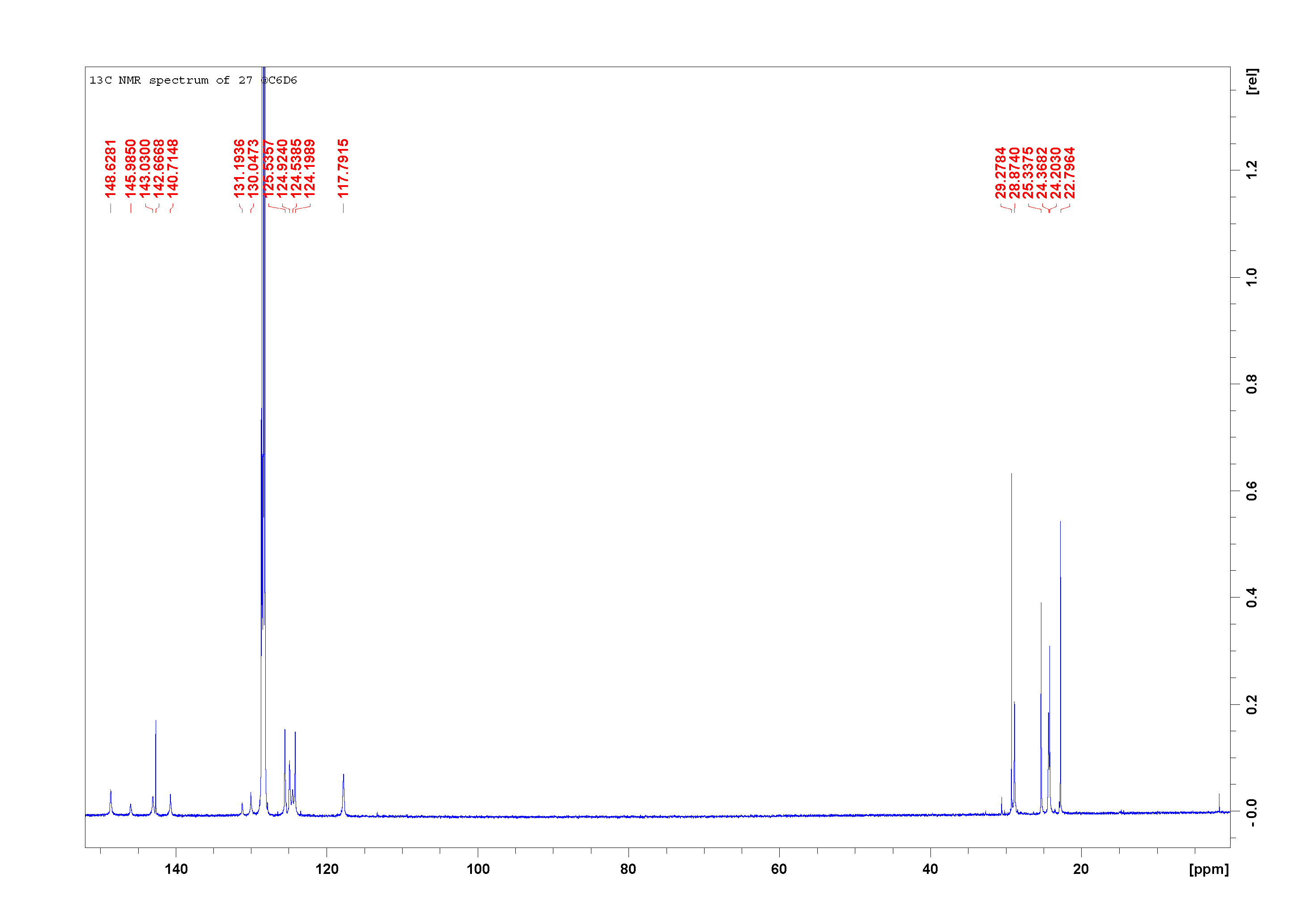


**Figure S67**. ^13^C NMR spectrum of **27** @C_6_D_6_, 295 K. Signals corresponding to the minor form are marked with black dots.


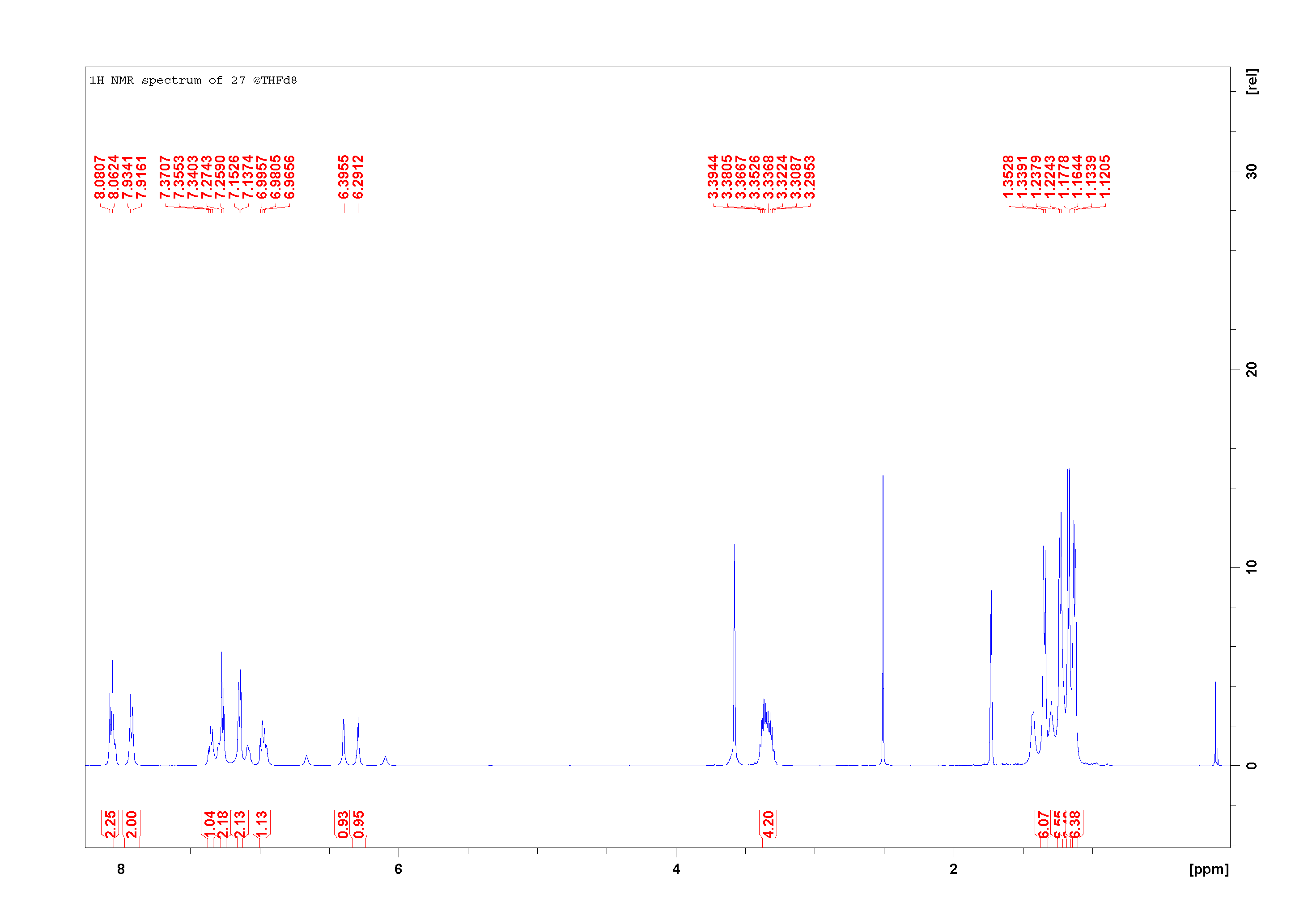


**Figure S68**. ^1^H NMR spectrum of **27** @THF-d_8_, 295 K. Signals corresponding to the minor form are marked with black dots.


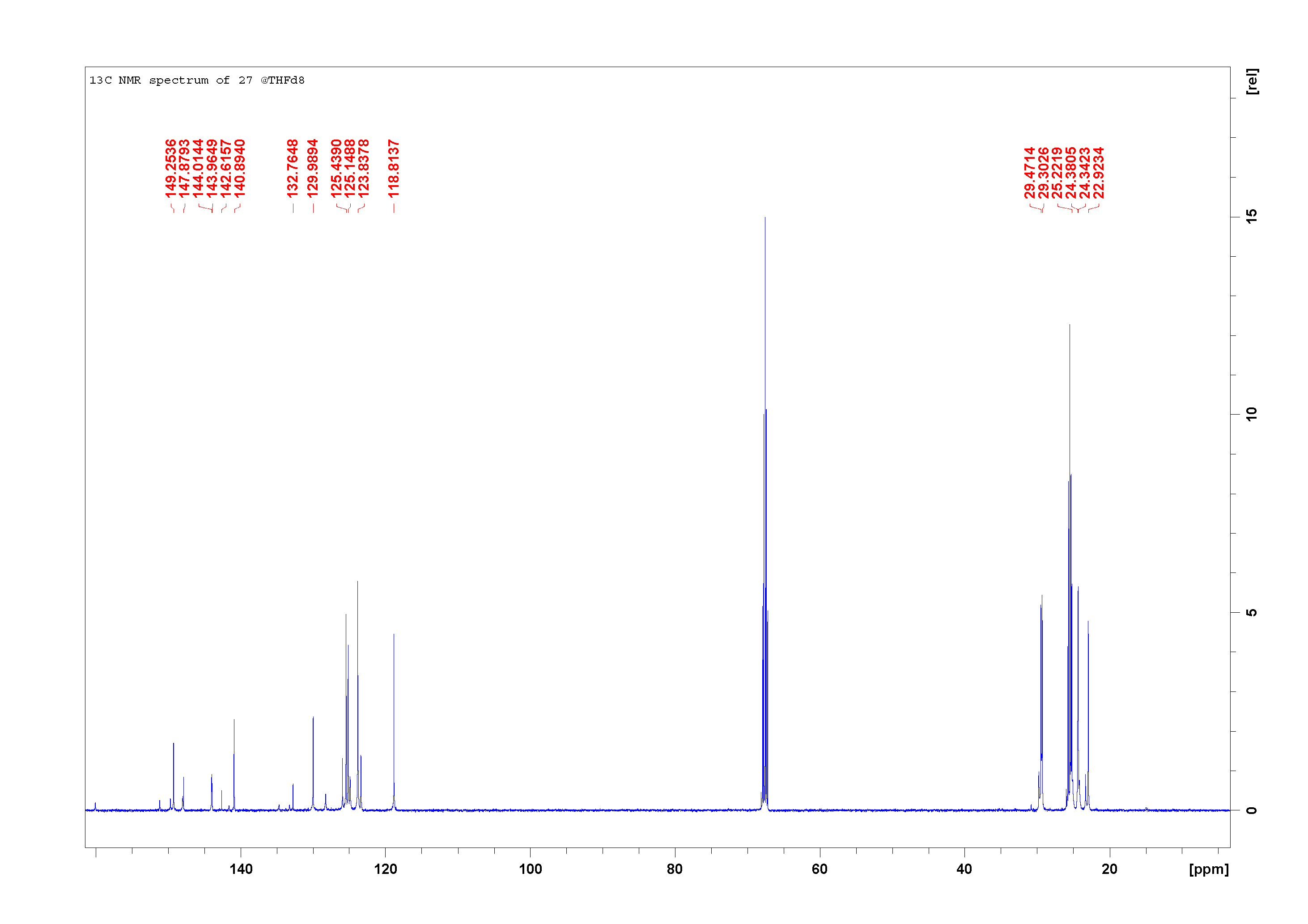


**Figure S69**. ^13^C NMR spectrum of **27** @THF-d_8_, 295 K. Signals corresponding to the minor form are marked with black dots.


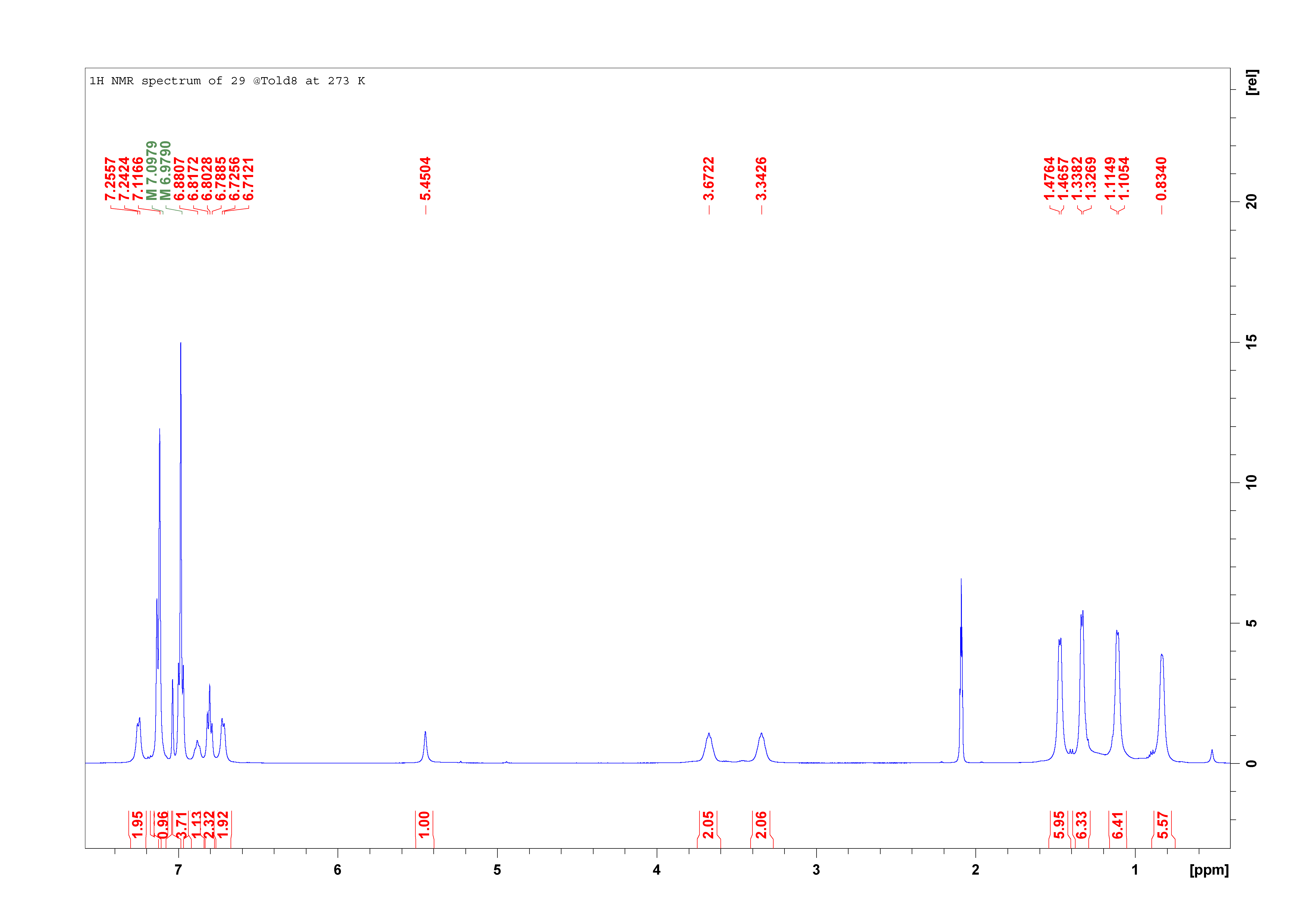


**Figure S70**. ^1^H NMR spectrum of **29** @Tol-d_8_, 273 K. Signals corresponding to the minor form are marked with black dots.


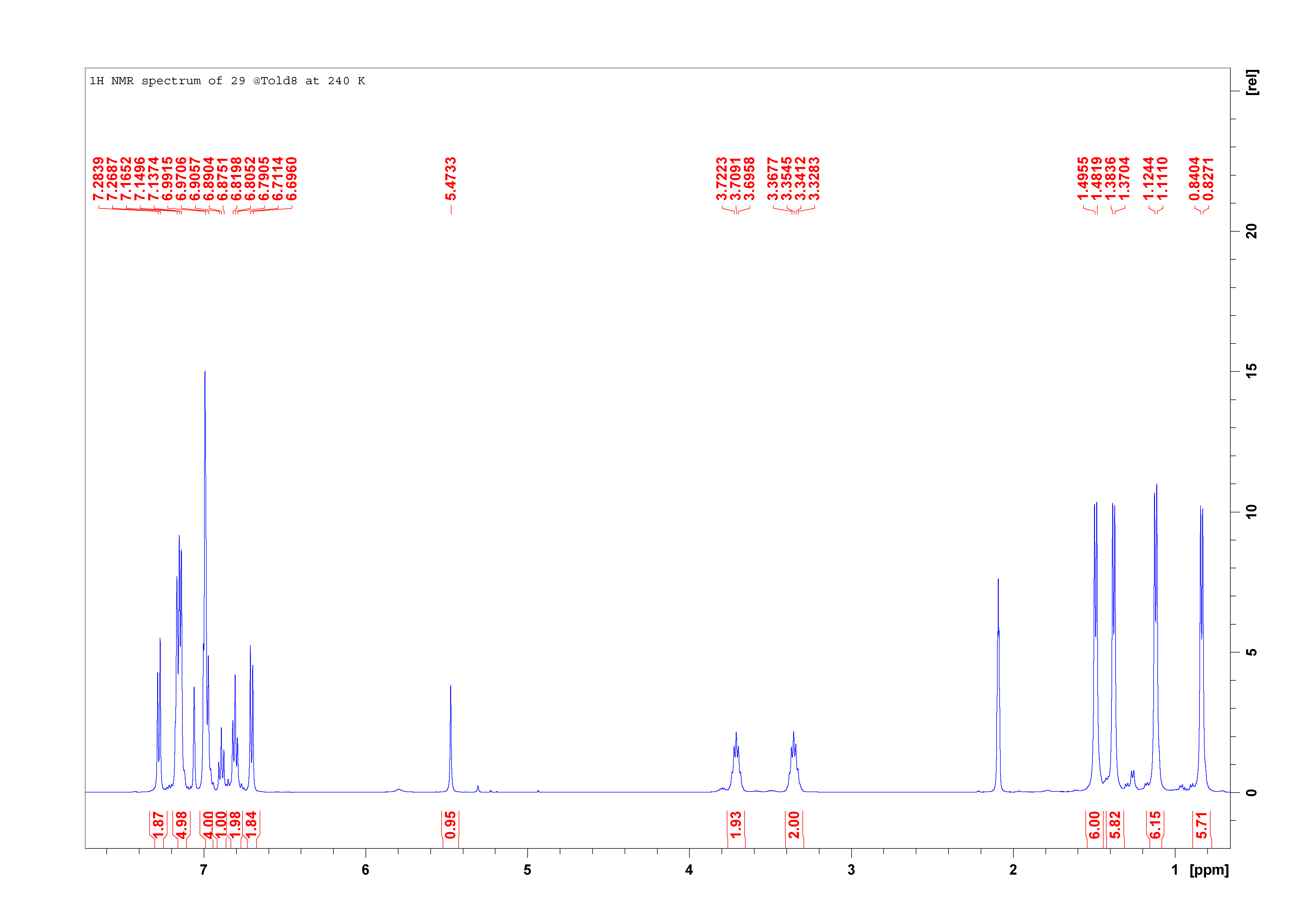


**Figure S71**. ^1^H NMR spectrum of **29** @Tol-d_8_, 240 K. Signals corresponding to the minor form are marked with black dots.

1. NMR spectra of all compounds are given in separate pdf file available in SI. [↑](#footnote-ref-1)
2. Gaussian 16, Revision C.01, Frisch, M. J.; Trucks, G. W.; Schlegel, H. B.; Scuseria, G. E.; Robb, M. A.; Cheeseman, J. R.; Scalmani, G.; Barone, V.; Petersson, G. A.; Nakatsuji, H.; Li, X.; Caricato, M.; Marenich, A. V.; Bloino, J.; Janesko, B. G.; Gomperts, R.; Mennucci, B.; Hratchian, H. P.; Ortiz, J. V.; Izmaylov, A. F.; Sonnenberg, J. L.; Williams-Young, D.; Ding, F.; Lipparini, F.; Egidi, F.; Goings, J.; Peng, B.; Petrone, A.; Henderson, T.; Ranasinghe, D.; Zakrzewski, V. G.; Gao, J.; Rega, N.; Zheng, G.; Liang, W.; Hada, M.; Ehara, M.; Toyota, K.; Fukuda, R.; Hasegawa, J.; Ishida, M.; Nakajima, T.; Honda, Y.; Kitao, O.; Nakai, H.; Vreven, T.; Throssell, K.; Montgomery, J. A., Jr.; Peralta, J. E.; Ogliaro, F.; Bearpark, M. J.; Heyd, J. J.; Brothers, E. N.; Kudin, K. N.; Staroverov, V. N.; Keith, T. A.; Kobayashi, R.; Normand, J.; Raghavachari, K.; Rendell, A. P.; Burant, J. C.; Iyengar, S. S.; Tomasi, J.; Cossi, M.; Millam, J. M.; Klene, M.; Adamo, C.; Cammi, R.; Ochterski, J. W.; Martin, R. L.; Morokuma, K.; Farkas, O.; Foresman, J. B.; Fox, D. J. Gaussian, Inc., Wallingford CT, **2016**. [↑](#footnote-ref-2)
3. Becke, A. D. Density‐functional thermochemistry. III. The role of exact exchange. *J. Chem. Phys.* **1993**, *98*, 5648-5652. https://doi.org/10.1063/1.464913. [↑](#footnote-ref-3)
4. Dunning, T. H. Gaussian basis sets for use in correlated molecular calculations. I. The atoms boron through neon and hydrogen. *J. Chem. Phys.* **1989**, *90*, 1007-1023. https://doi.org/10.1063/1.456153. [↑](#footnote-ref-4)
5. Tomasi, J.; Mennucci, B.; Cammi, R. Quantum Mechanical Continuum Solvation Models. *Chem. Rev.* **2005**, *105*, 2999-3094. https://doi.org/10.1021/cr9904009. [↑](#footnote-ref-5)
6. Grimme, S.; Antony, J.; Ehrlich, S.; Krieg, H. A consistent and accurate ab initio parametrization of density functional dispersion correction (DFT-D) for the 94 elements H-Pu. *J. Chem. Phys.* **2010**, *132*, 154104. https://doi.org/10.1063/1.3382344. [↑](#footnote-ref-6)
7. Patt, S. L.; Shoolery, J. N. Attached proton test for carbon-13 NMR *J. Magn. Reson.* **1982**, *46*, 535-539. https://doi.org/10.1016/0022-2364(82)90105-6 [↑](#footnote-ref-7)
8. Otwinowski, Z.; Minor, W. Processing of X-ray diffraction data collected in oscillation mode. *Methods Enzymol* **1997**, *276*, 307-326. https://doi.org/10.1016/S0076-6879(97)76066-X. [↑](#footnote-ref-8)
9. P. Coppens In: F.R. Ahmed, S.R. Hall, C.P. Huber Editors, Crystallographic Computing, **1970**, pp. 255 – 270, Copenhagen, Munksgaard. [↑](#footnote-ref-9)
10. Altomare, A.; Cascarano, G.; Giacovazzo, C.; Guagliardi, A. Early finding of preferred orientation: a new method. *J. Appl. Cryst.* **1994**, *27*, 1045-1050. https://doi.org/10.1107/S002188989400422X. [↑](#footnote-ref-10)
11. Sheldrick, G. M. SHELXL-97, University of Göttingen: Göttingen, **2008**. [↑](#footnote-ref-11)
12. Sheldrick, G. M. Crystal structure refinement with SHELXL. *Acta Cryst. C* **2015**, *71*, 3-8. https://doi.org/10.1107/S2053229614024218. [↑](#footnote-ref-12)
