# Supplementary material for: Comprehensive Reinvestigation of Carbodiimide Guanylation: HCl-Initiated Access to Tri- and Tetrasubstituted Guanidines
Source: ACS Omega. 2026 Apr 16;11(16):24075–85. doi: 10.1021/acsomega.5c12436 (PMC13129877; doi:10.1021/acsomega.5c12436)
Supplement: Supplementary file 6 [file ao5c12436_si_006.pdf]

Comprehensive Reinvestigation of Carbodiimide Guanylation: HCl-initated Access to Tri- and Tetrasubstituted Guanidines

Lukáš Vlk,[a] Karel Pauk,[b] Maksim A. Samsonov,[a] Zdeňka Růžičková, [a] Tomáš Chlupatý,[a,*] and Aleš Růžička[a,*]

[a]*University of Pardubice, Studentská 573, CZ-532 10, Pardubice, Czech Republic.*

[b]*Institute of Organic Chemistry and Technology, Faculty of Chemical Technology, University of Pardubice, Studentská 573, CZ-532 10, Pardubice, Czech Republic.*

*Tomáš Chlupatý: tomas.chlupaty@upce.cz; Aleš Růžička: – ales.ruzicka@upce.cz

Table of contents

NMR spectra of 1-29 – Figures S72-S329..Pages S2-S259

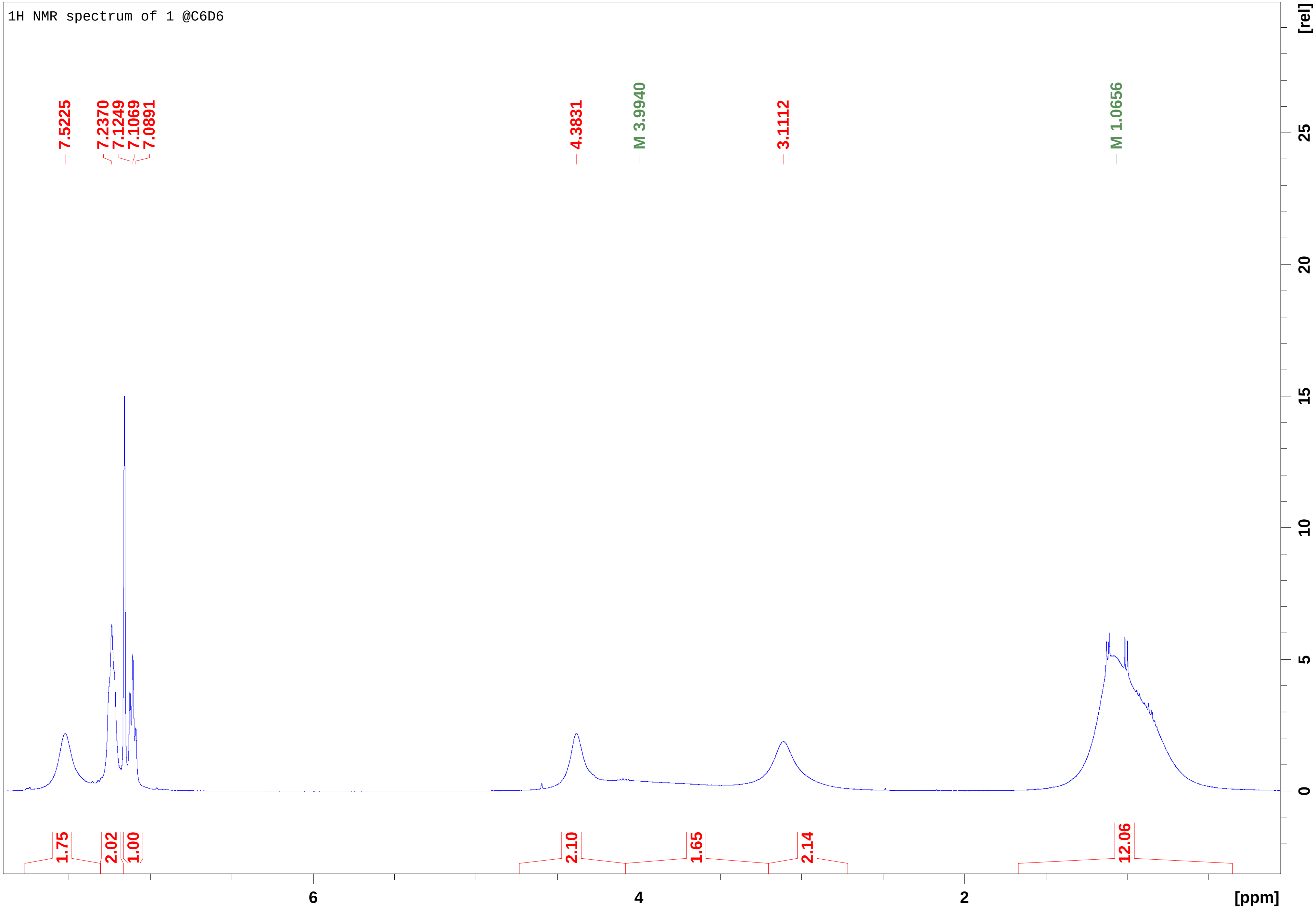

Figure S72. 1H NMR spectrum of 1 in C6D6

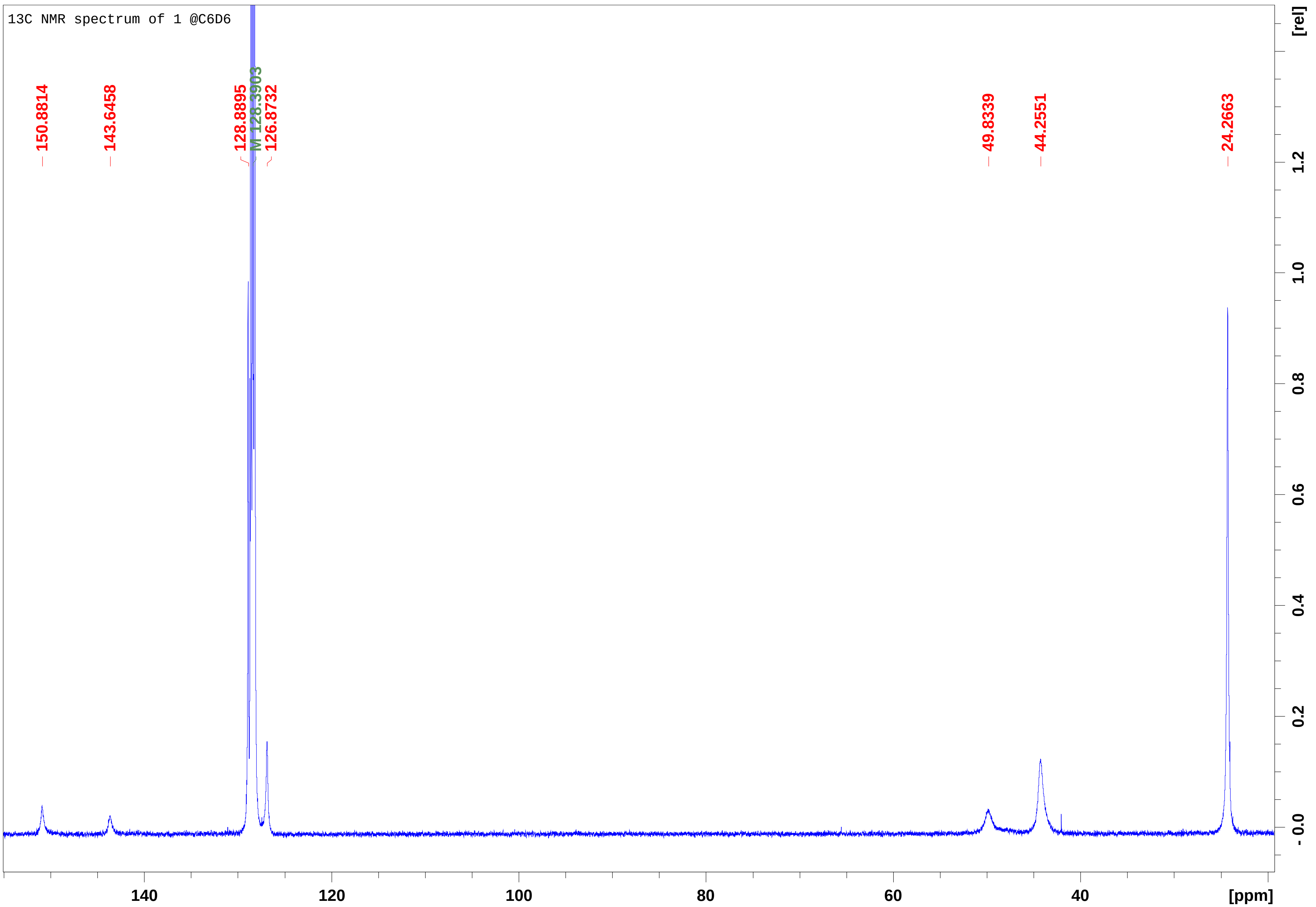

Figure S73. 13C NMR spectrum of 1 in C6D6

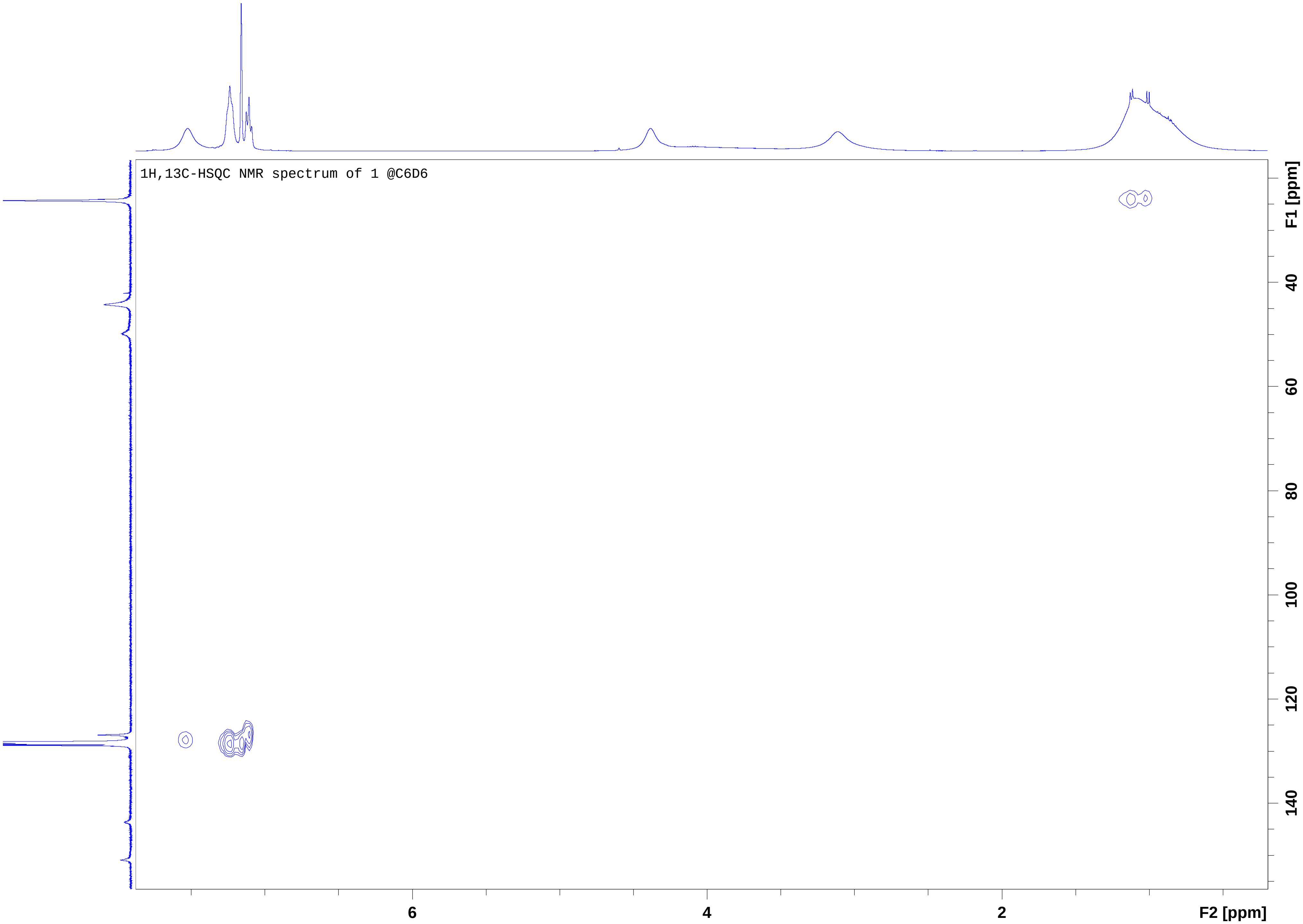

Figure S74. 1H NMR spectrum of 1 in C6D6

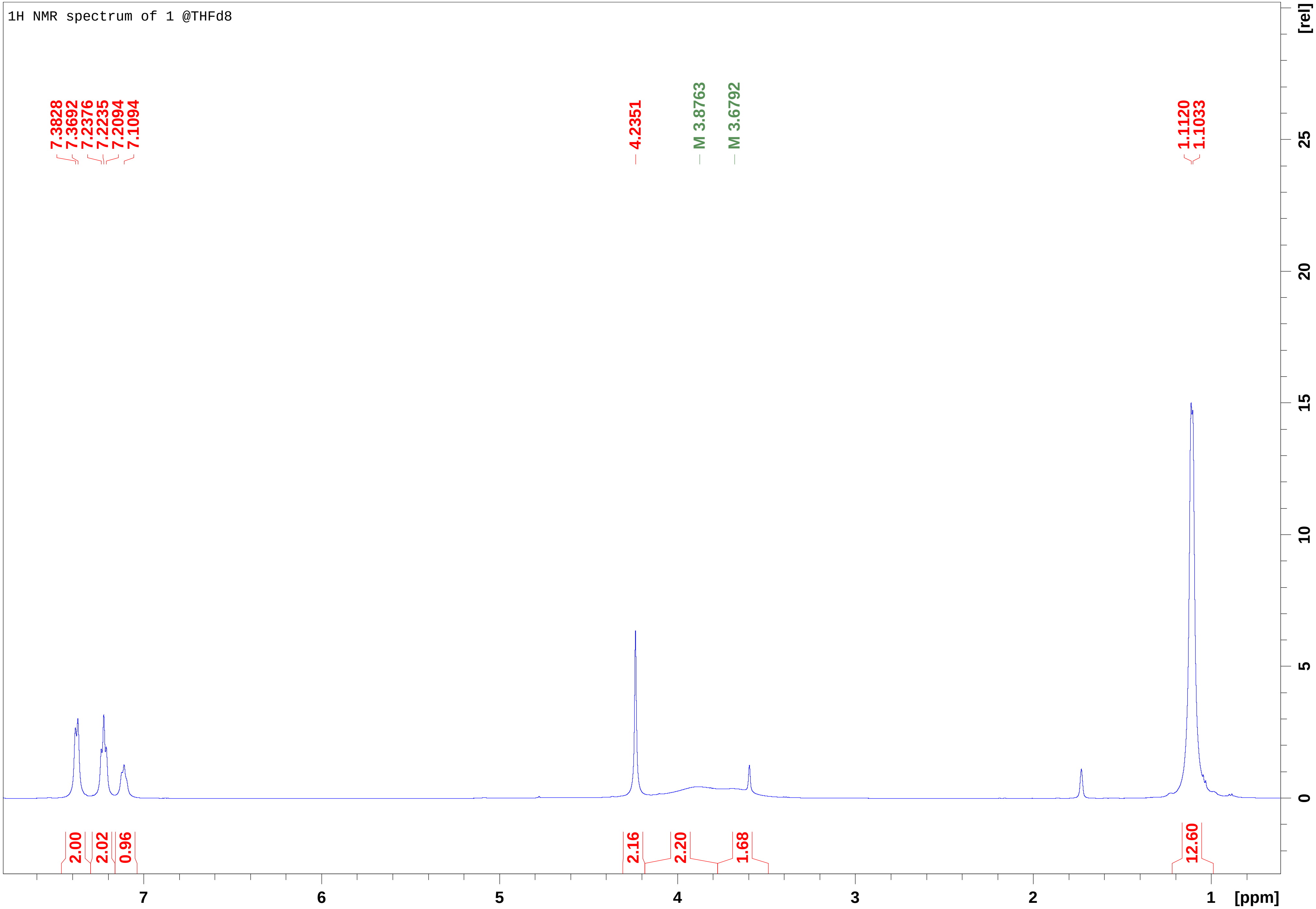

Figure S75. 1H NMR spectrum of 1 in THF-d8

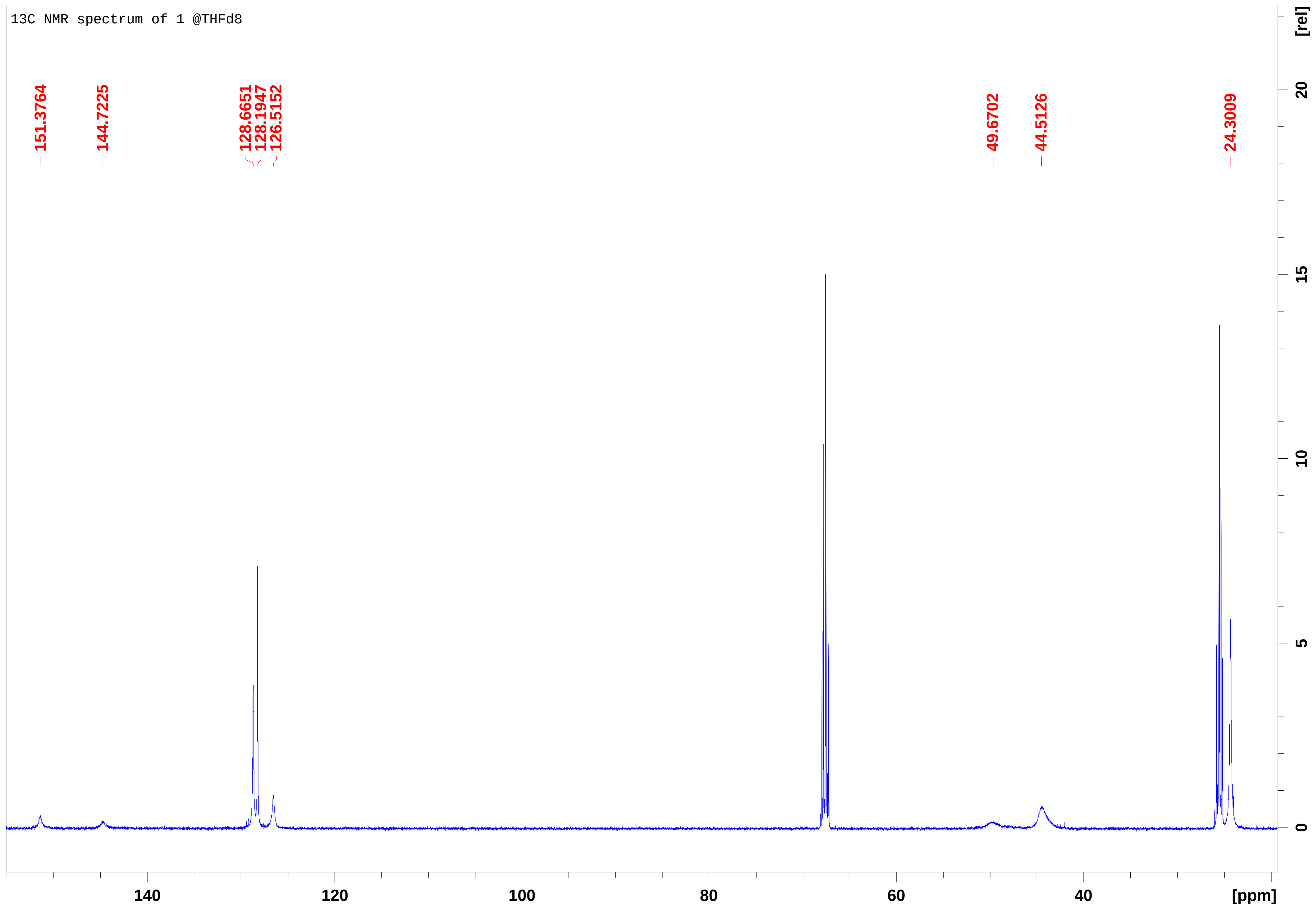

Figure S76. 13C NMR spectrum of 1 in THF-d8

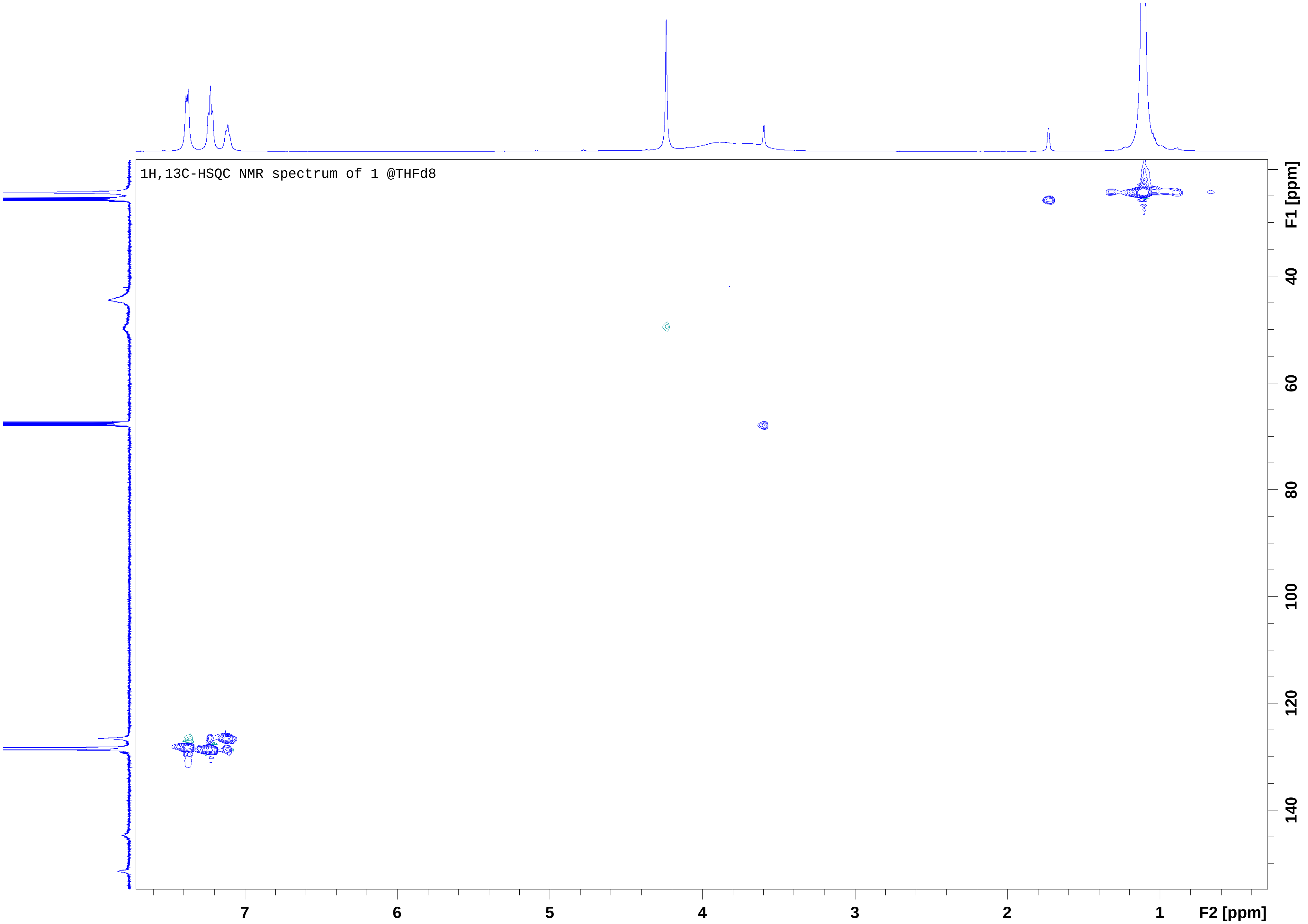

Figure S77. 1H, 13C-HSQC NMR spectrum of 1 in THF-d8

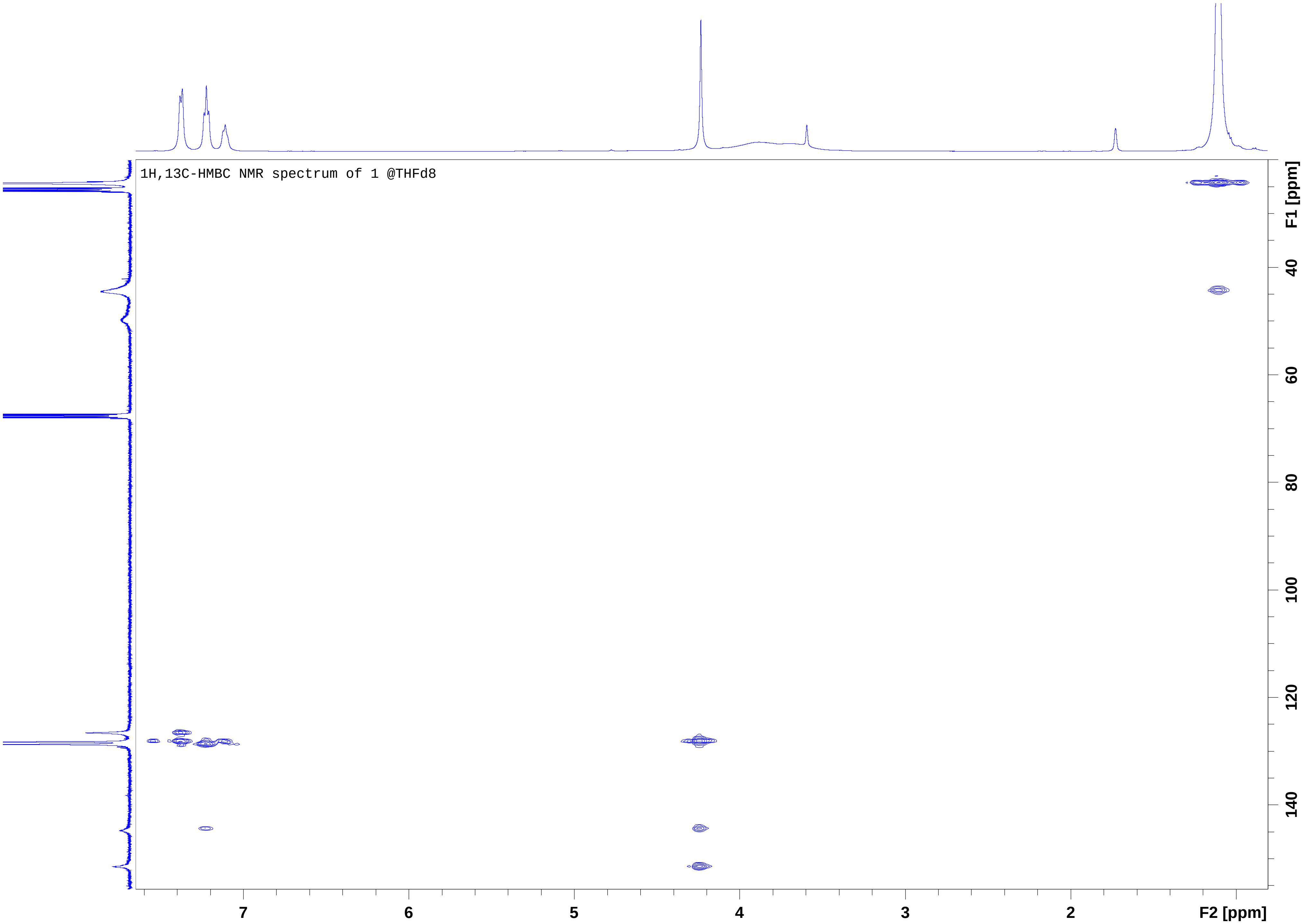

Figure S78. 1H, 13C-HMBC NMR spectrum of 1 in THF-d8

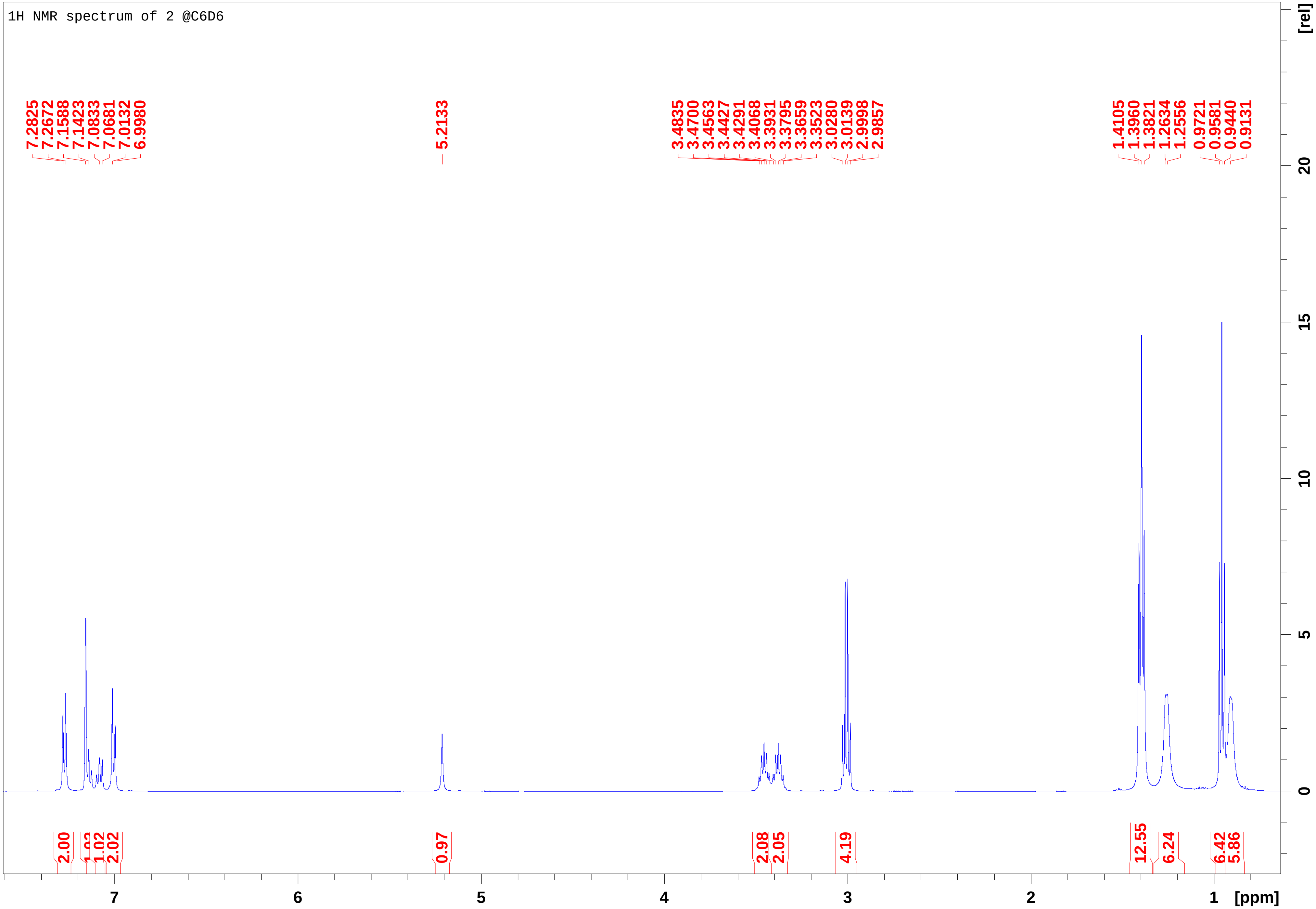

Figure S79. 1H NMR spectrum of 2 in C6D6

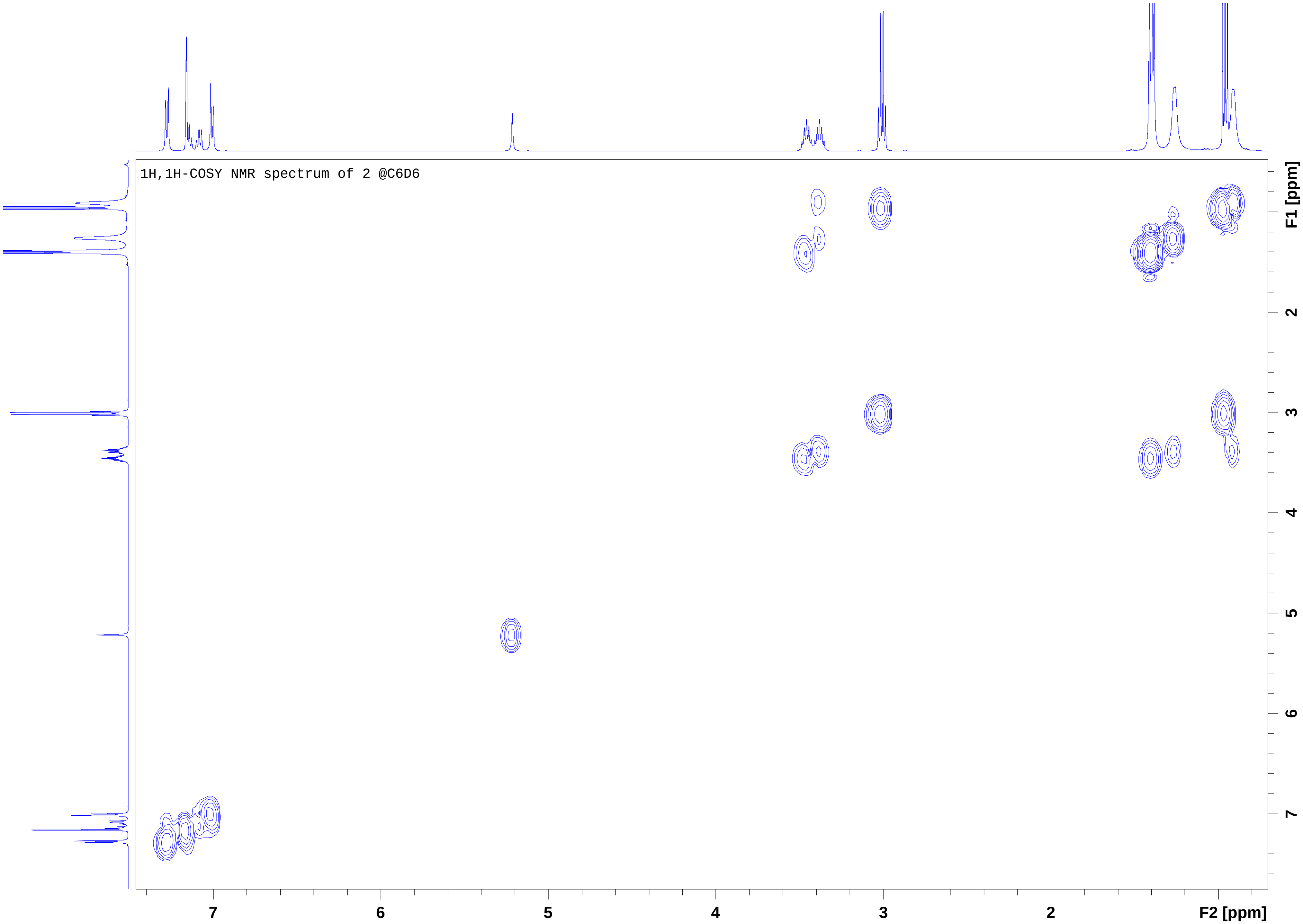

Figure S80. 1H,1H-COSY NMR spectrum of 2 in C_6D_6

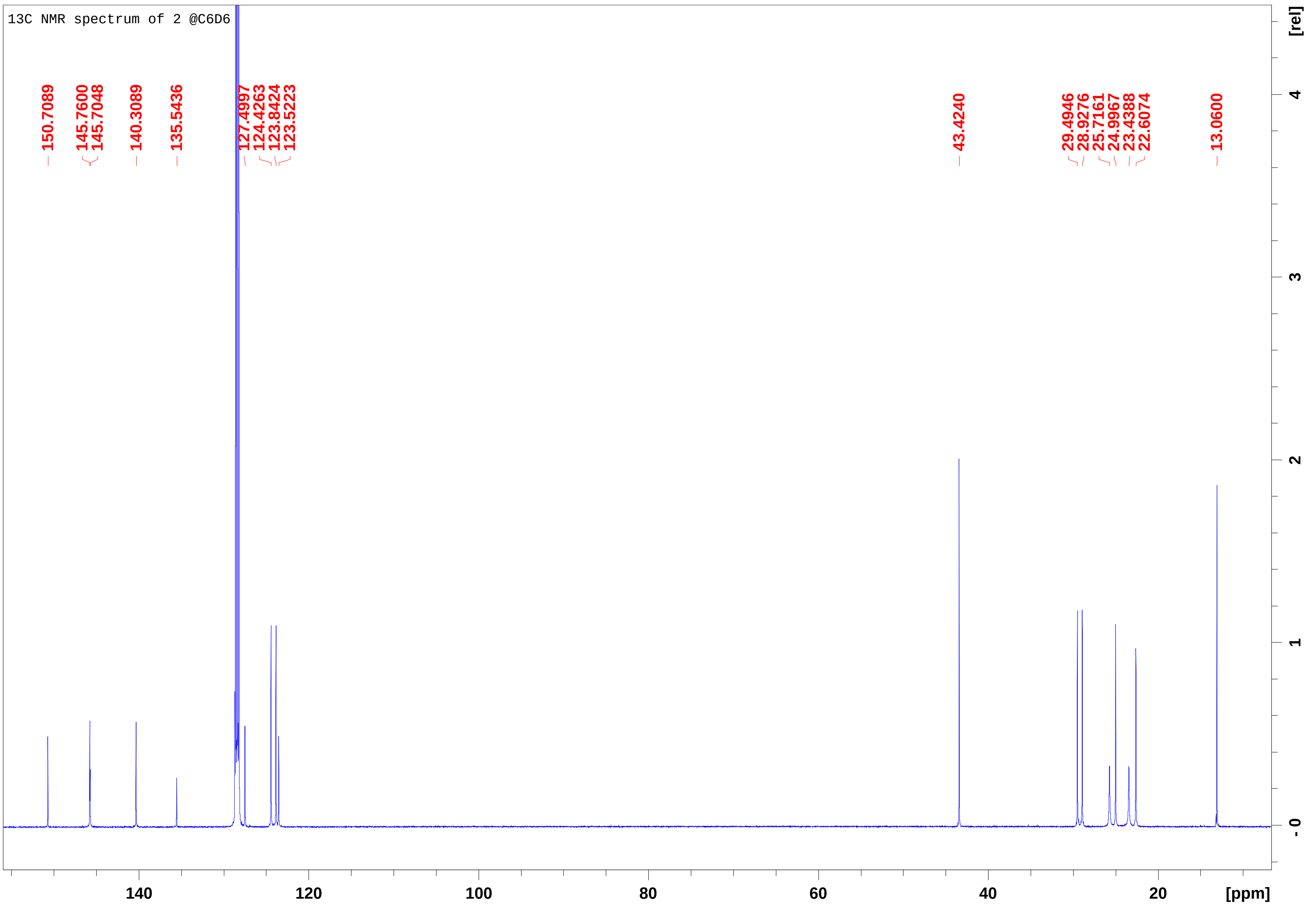

Figure S81. 13C NMR spectrum of 2 in C6D6

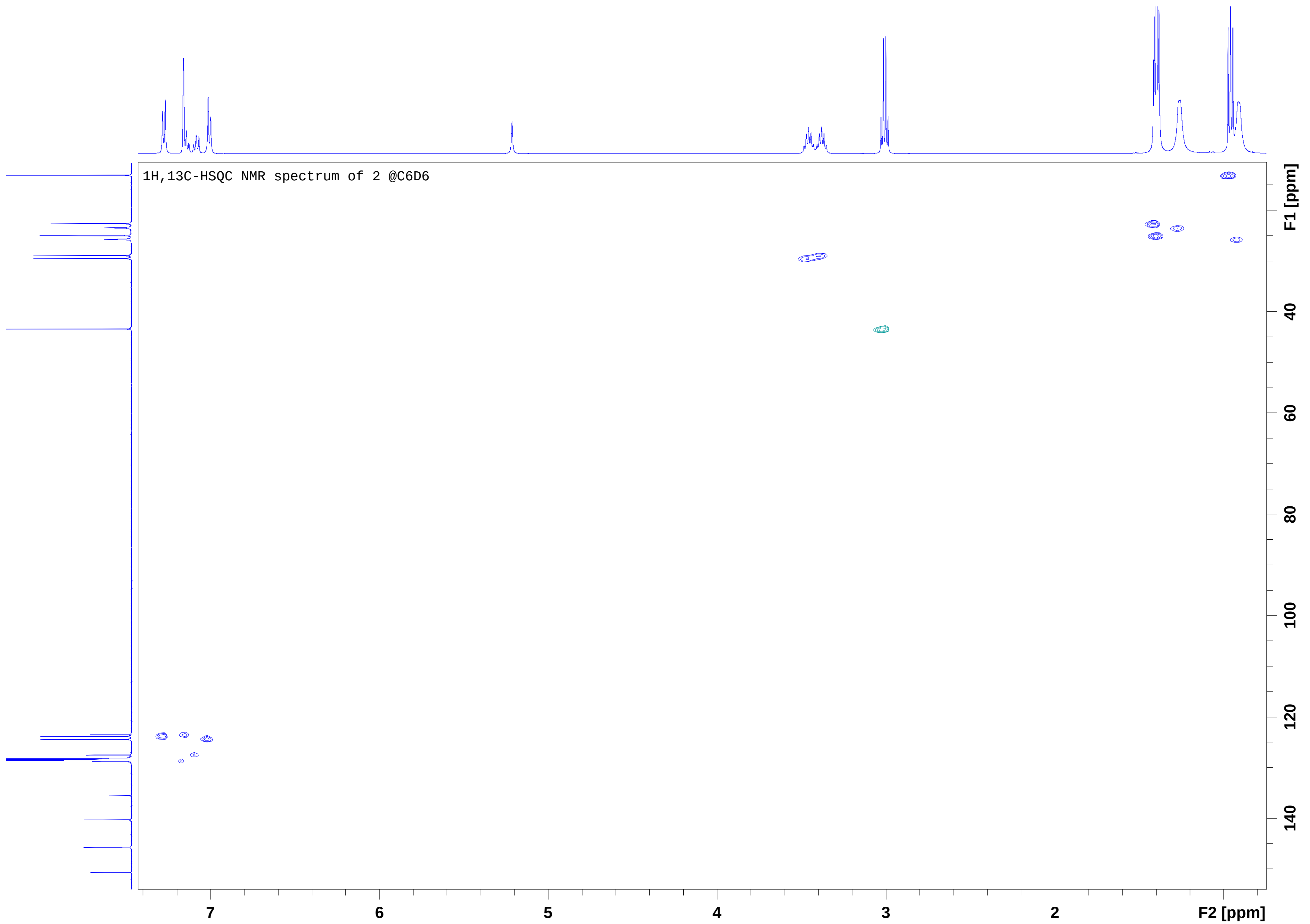

Figure S82. 1H,13C-HSQC NMR spectrum of 2 in C6D6

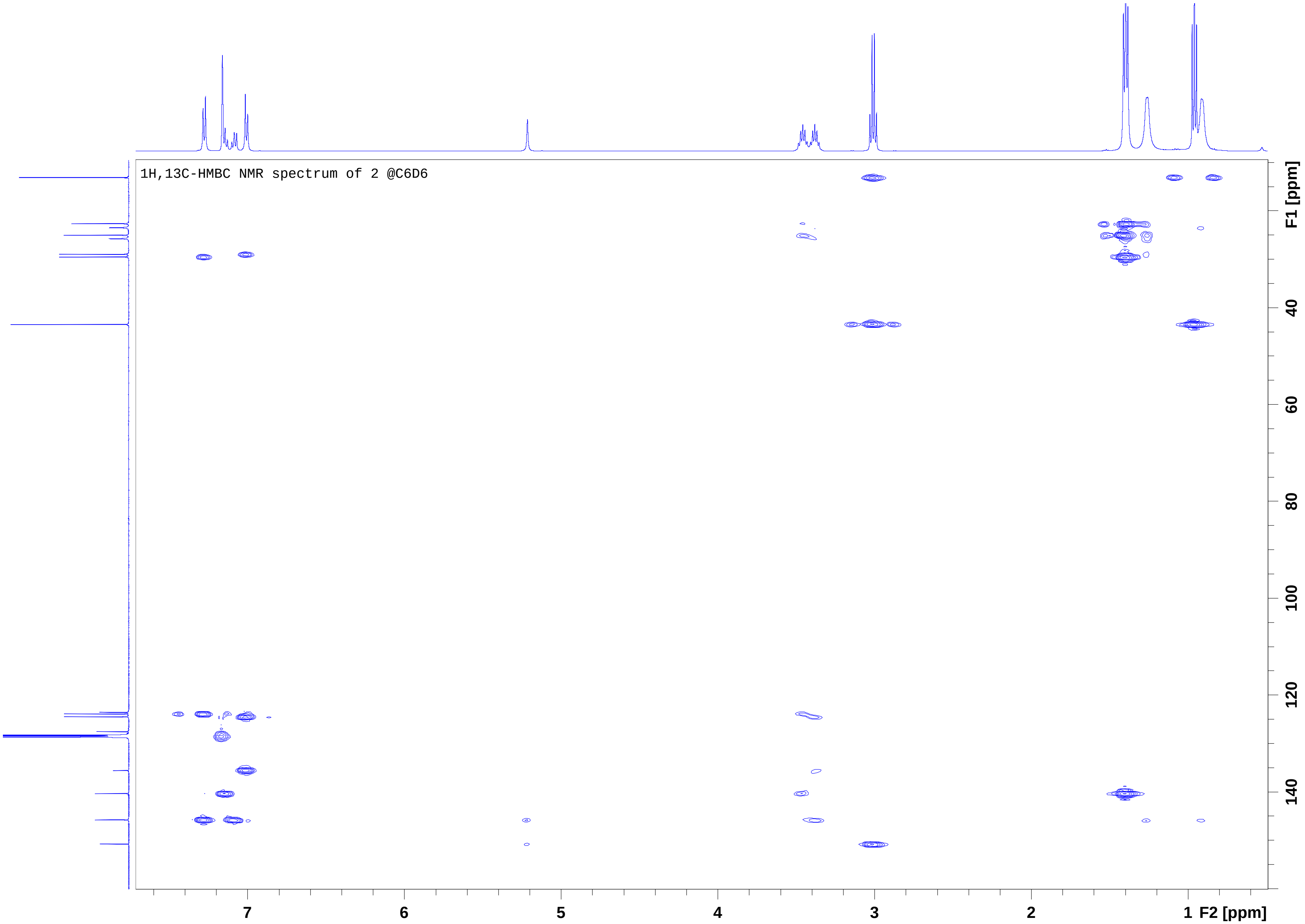

Figure S83. 1H,13C-HMBC NMR spectrum of 2 in C6D6

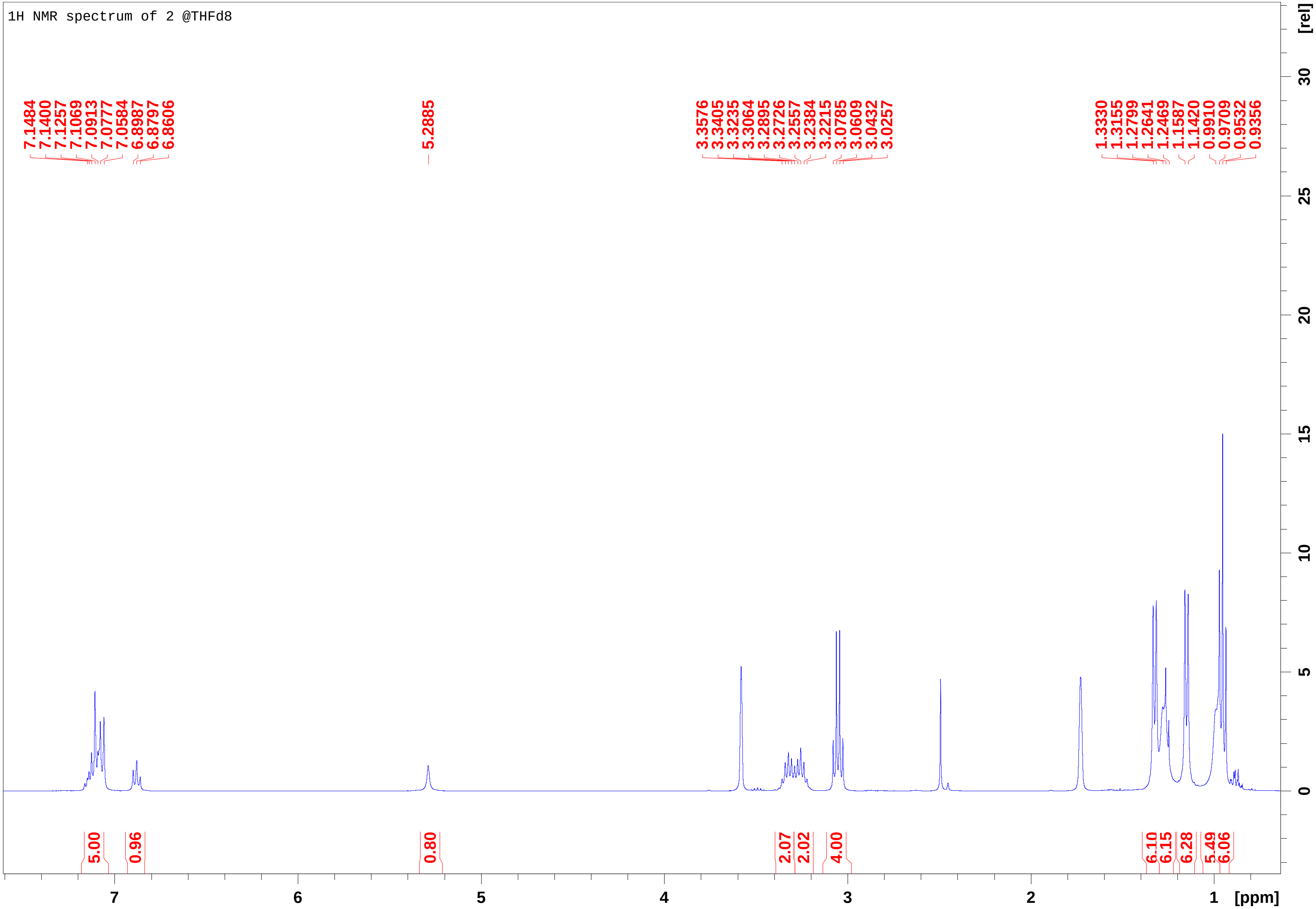

Figure S84. 1H NMR spectrum of 2 in THF-d8

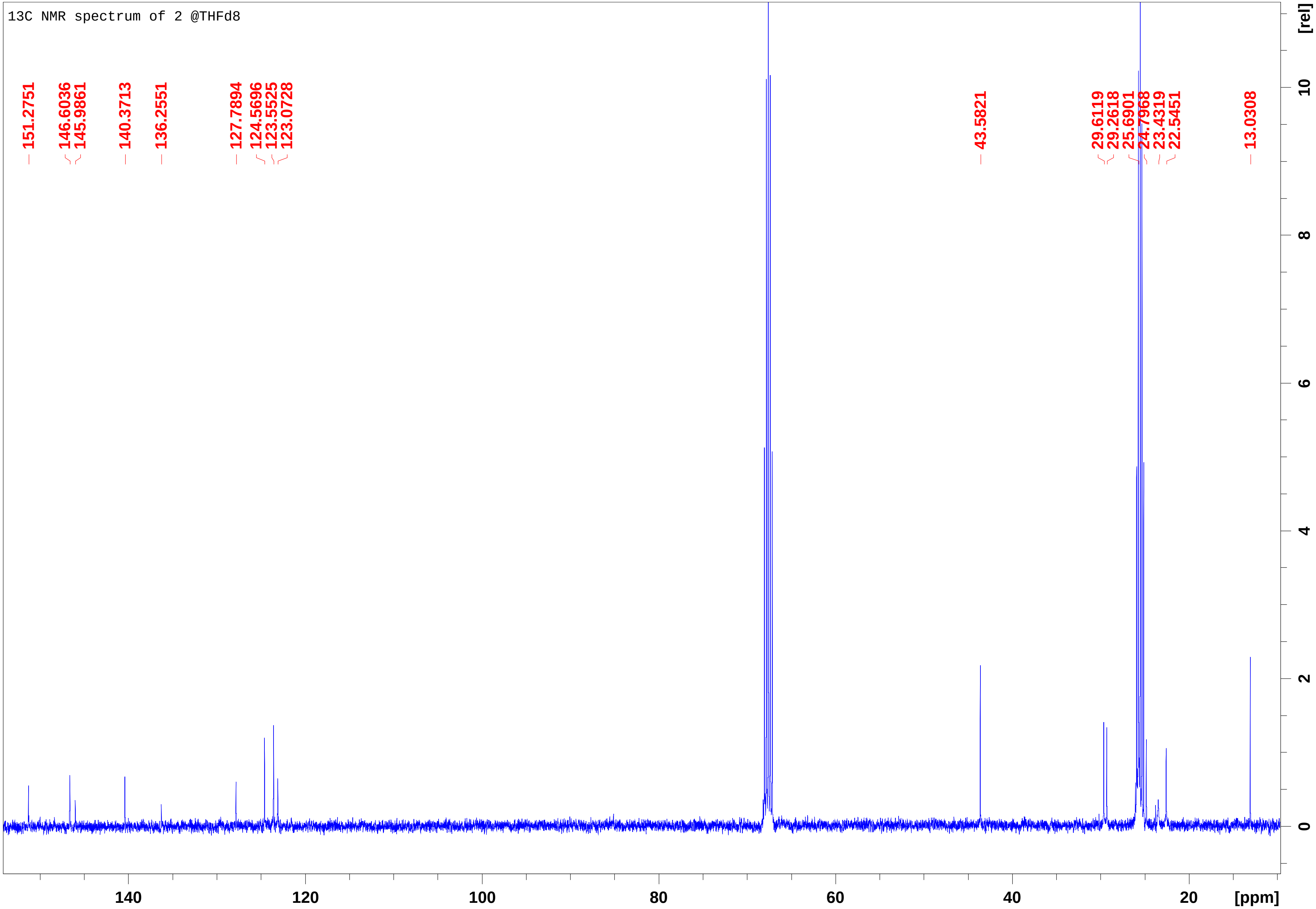

Figure S85. 13C NMR spectrum of 2 in THF-d8

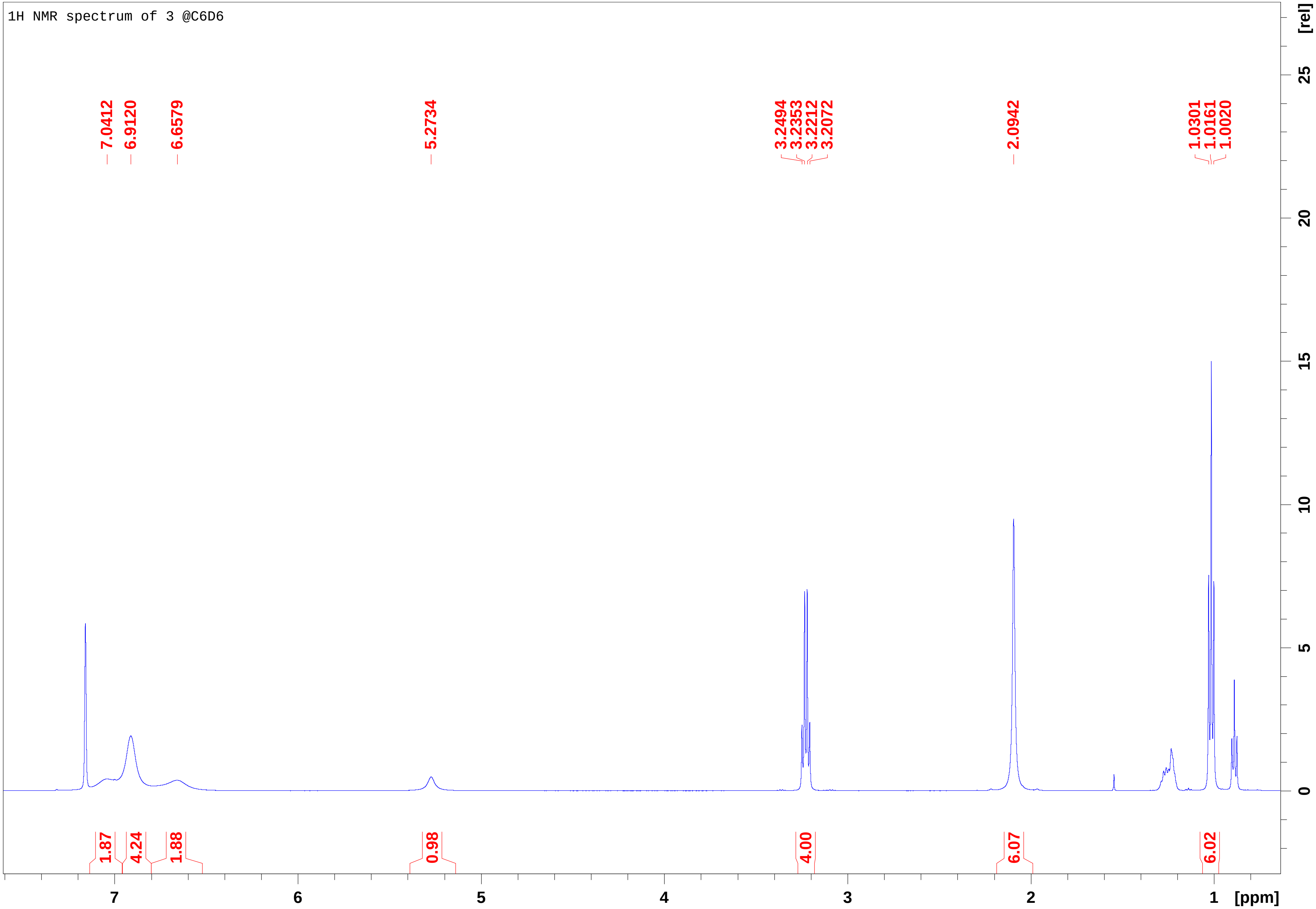

Figure S86. 1H NMR spectrum of 3 in C6D6

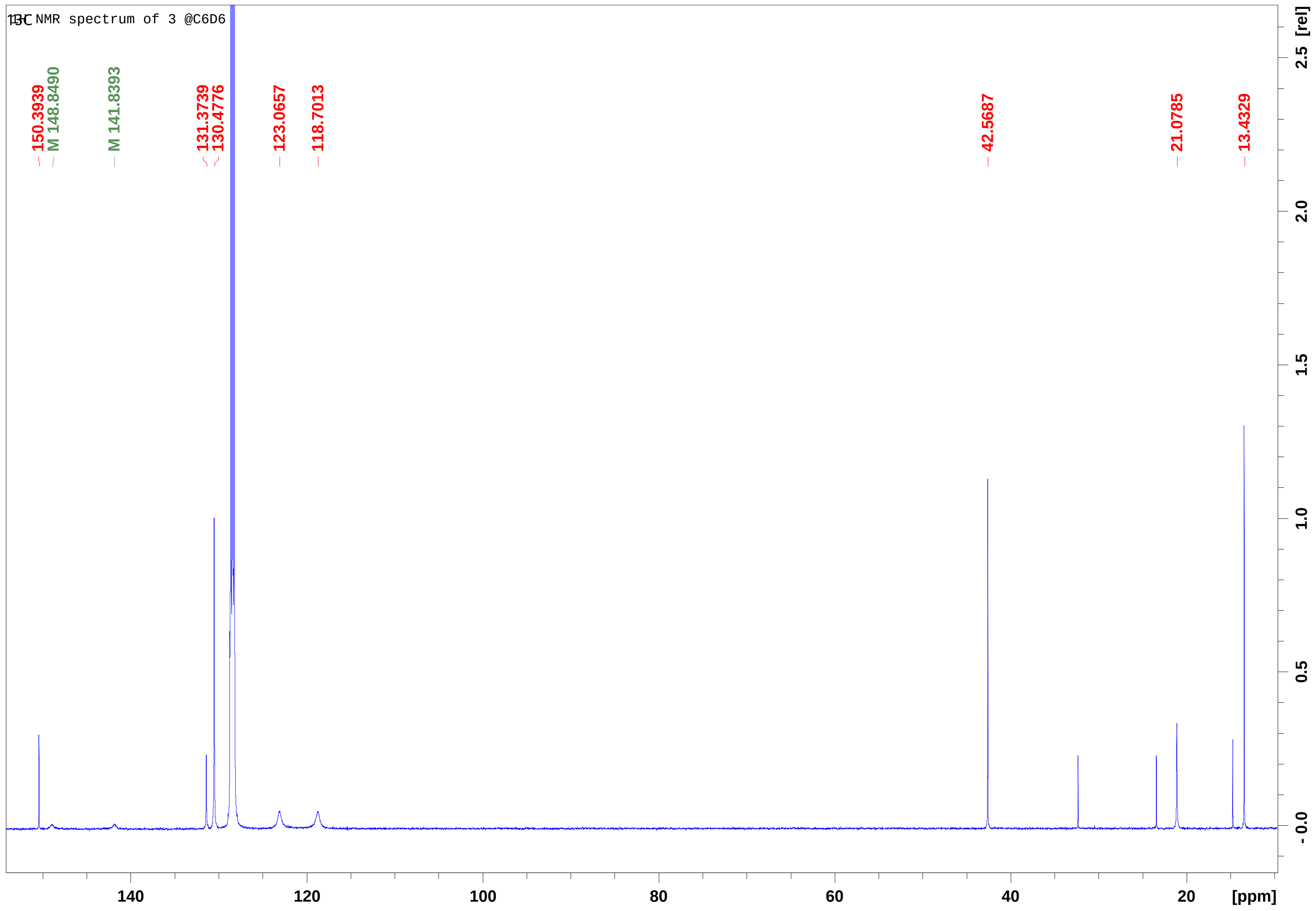

Figure S87. 13C NMR spectrum of 3 in C6D6

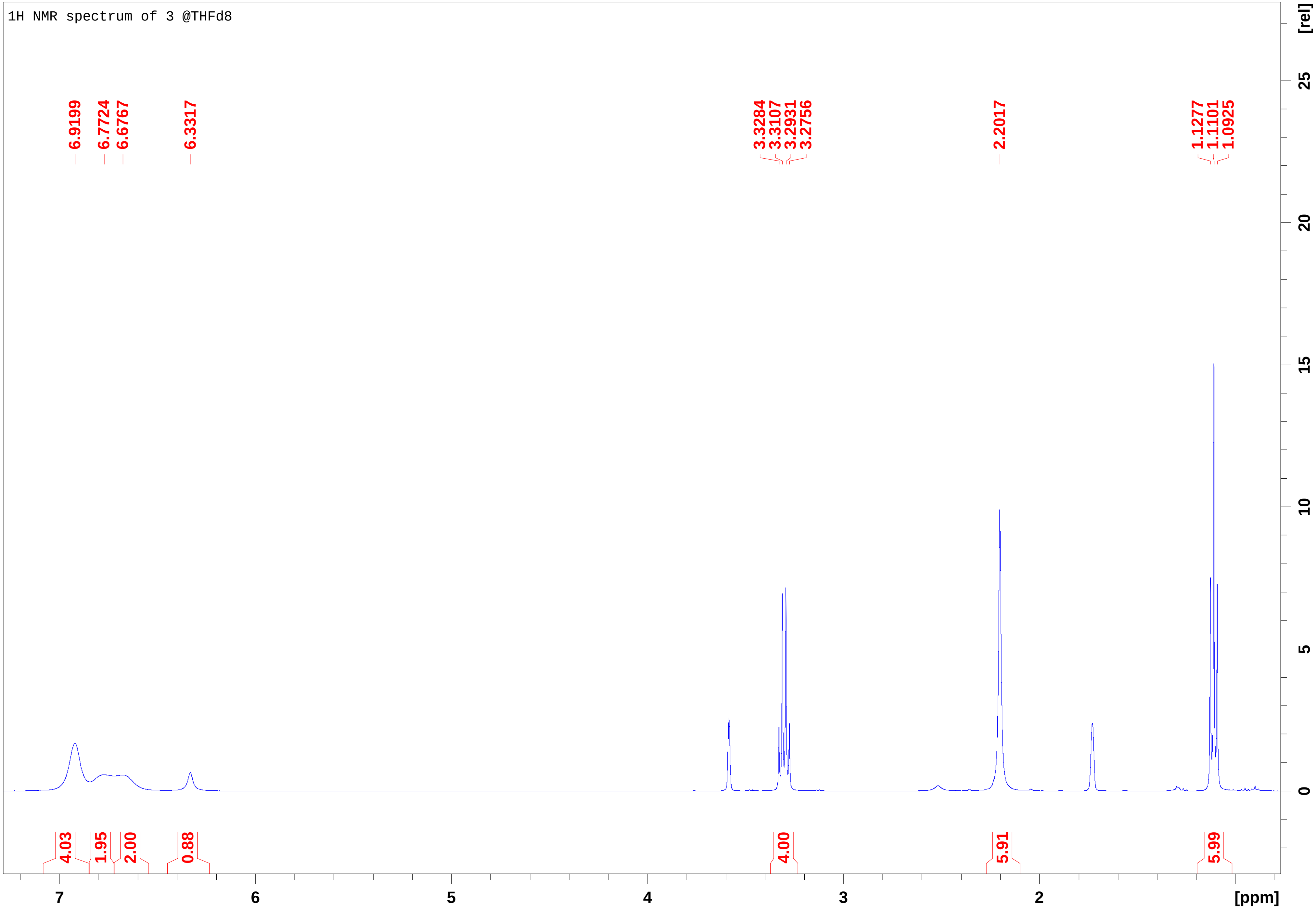

Figure S88. 1H NMR spectrum of 3 in THF-d8

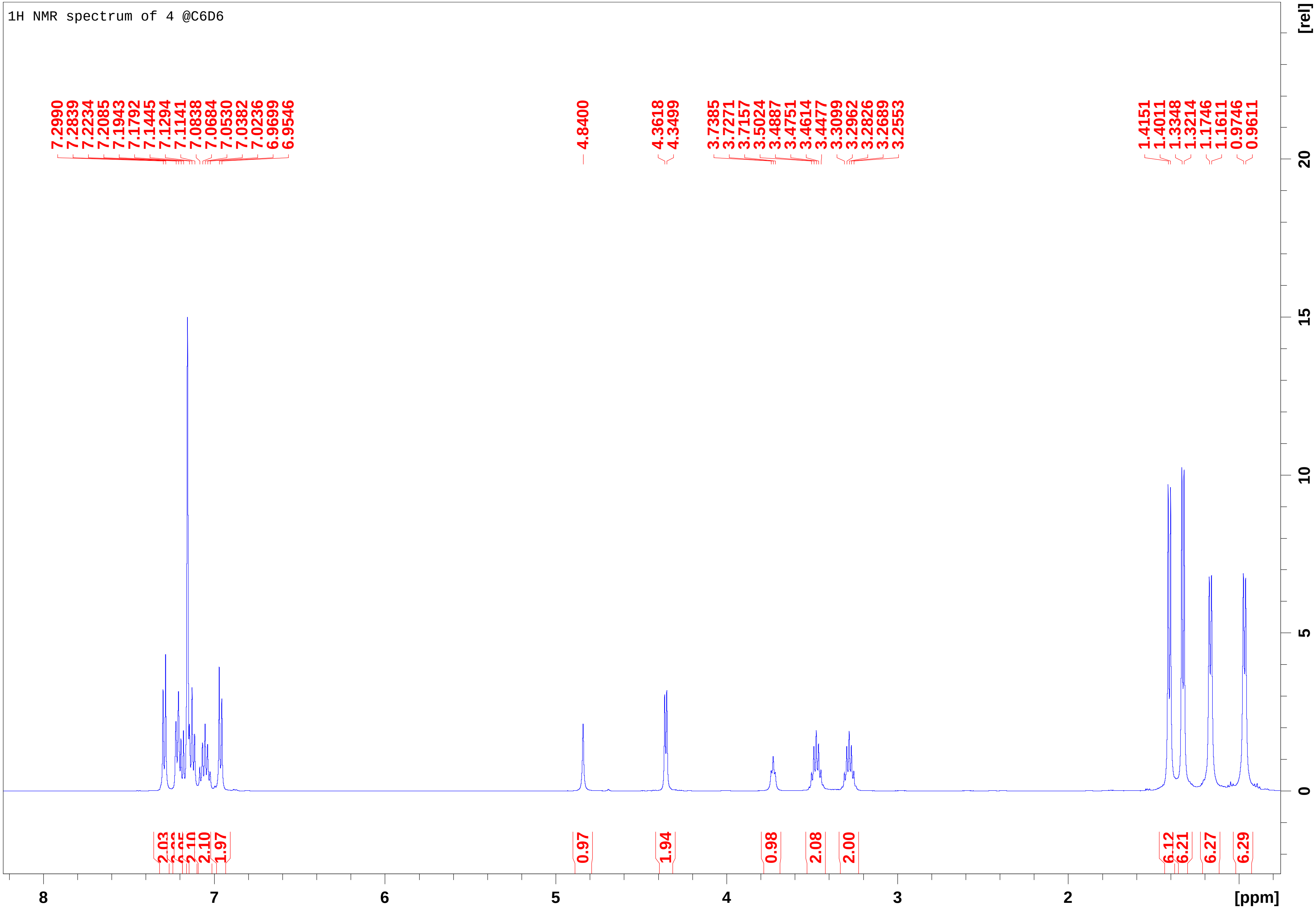

Figure S89. 1H NMR spectrum of 4 in C6D6

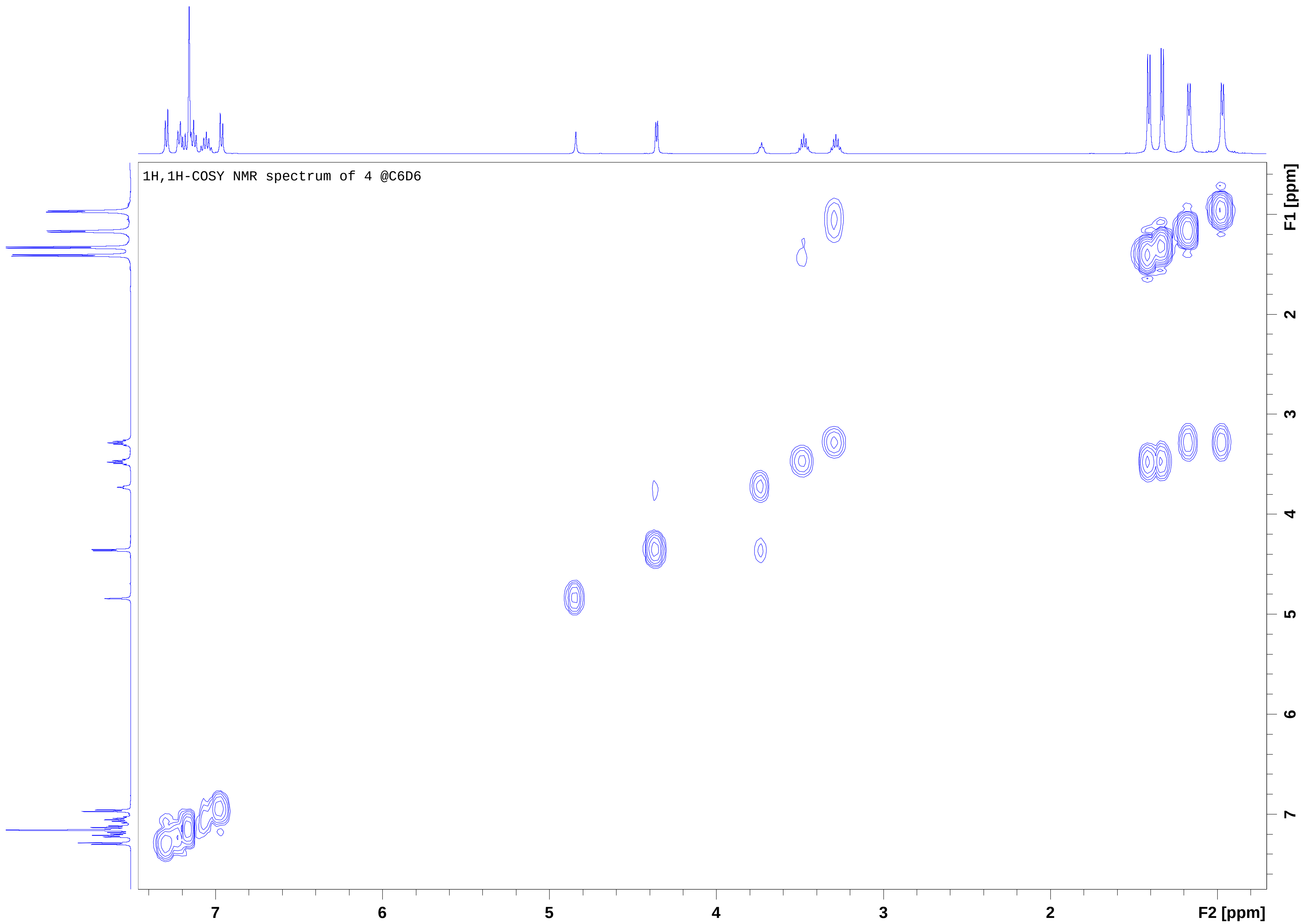

Figure S90. 1H,1H-COSY NMR spectrum of 4 in C6D6

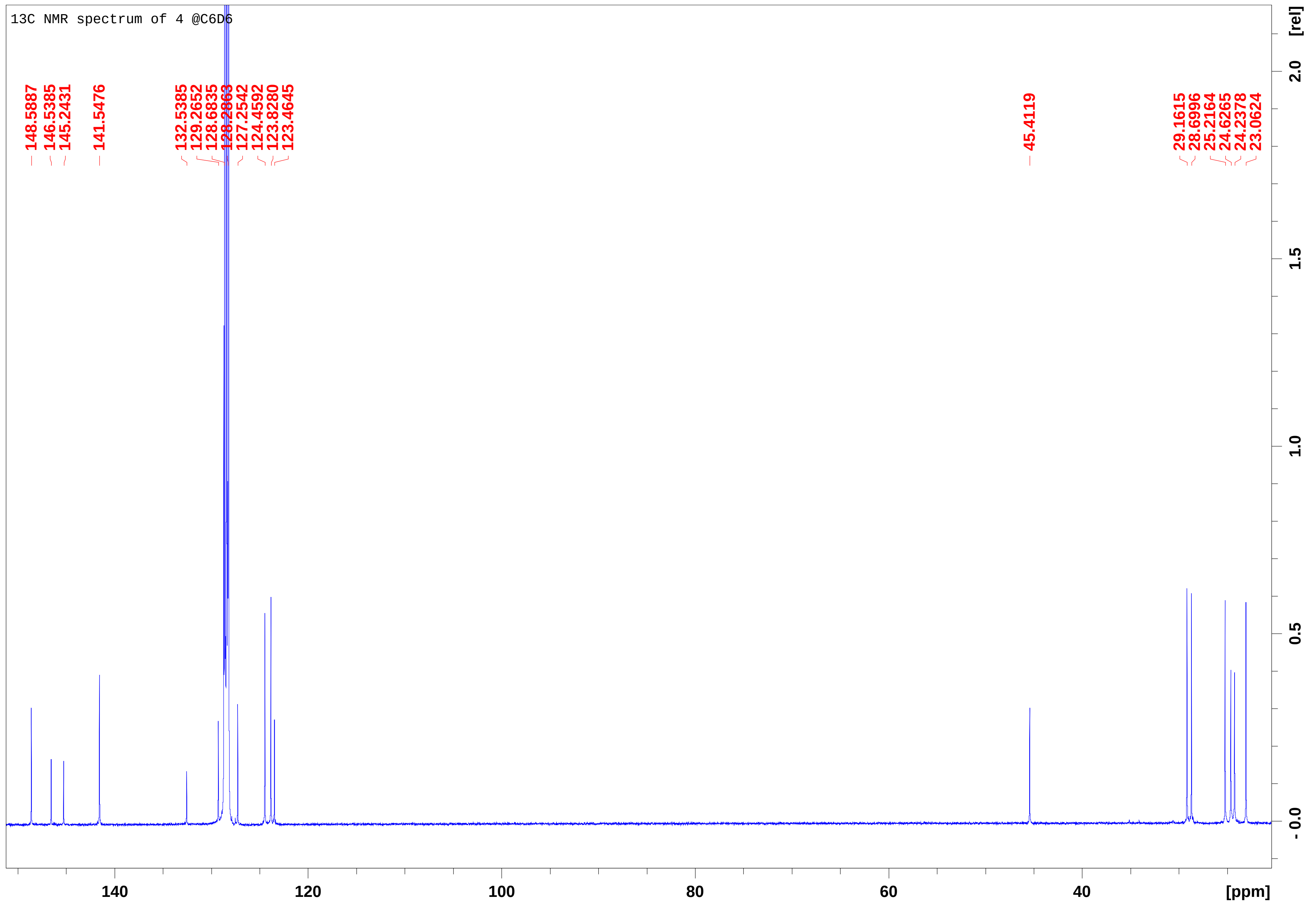

Figure S91. 13C NMR spectrum of 4 in C6D6

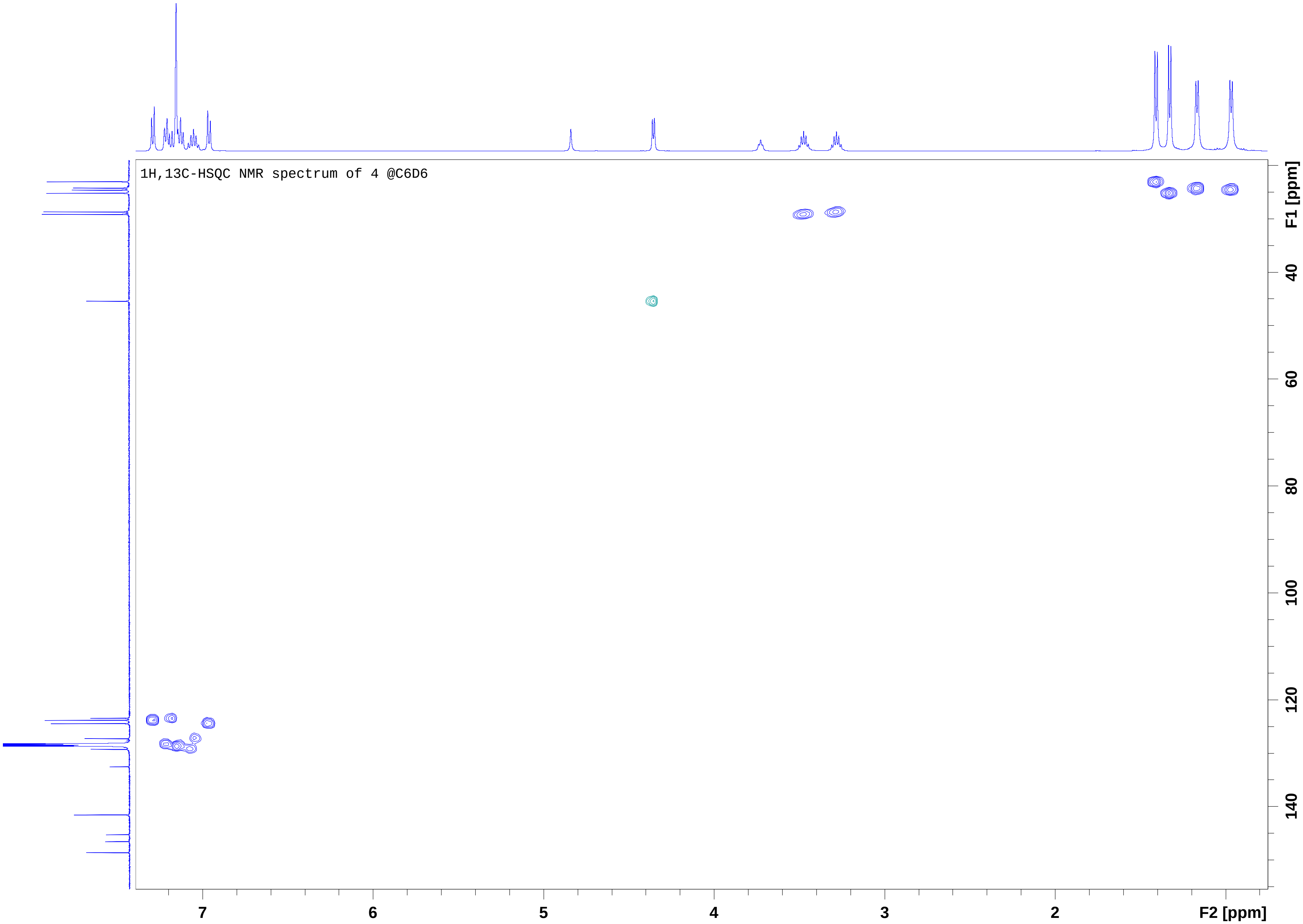

Figure S92. 1H,13C-HSQC NMR spectrum of 4 in C6D6

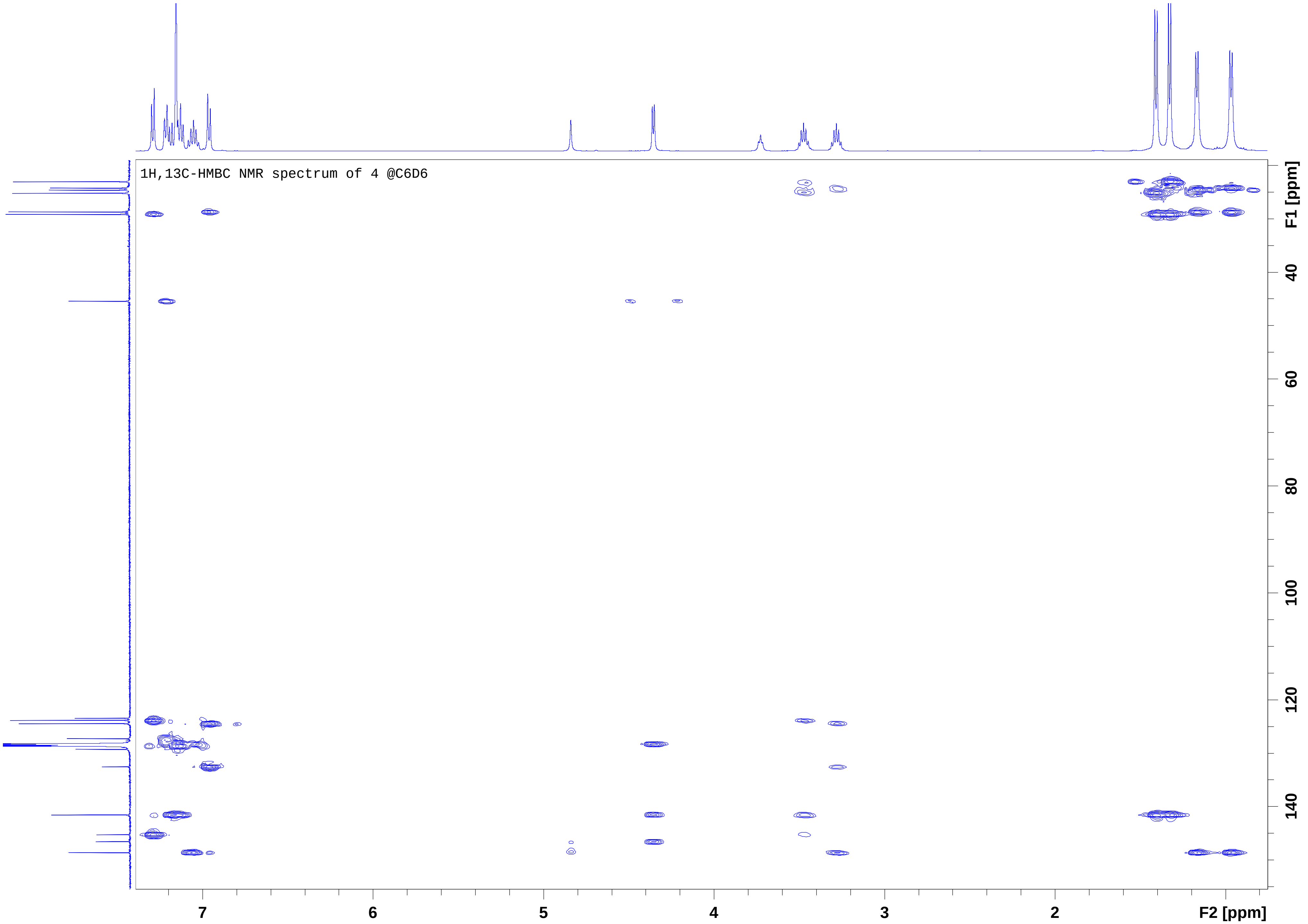

Figure S93. 1H,13C-HMBC NMR spectrum of 4 in C6D6

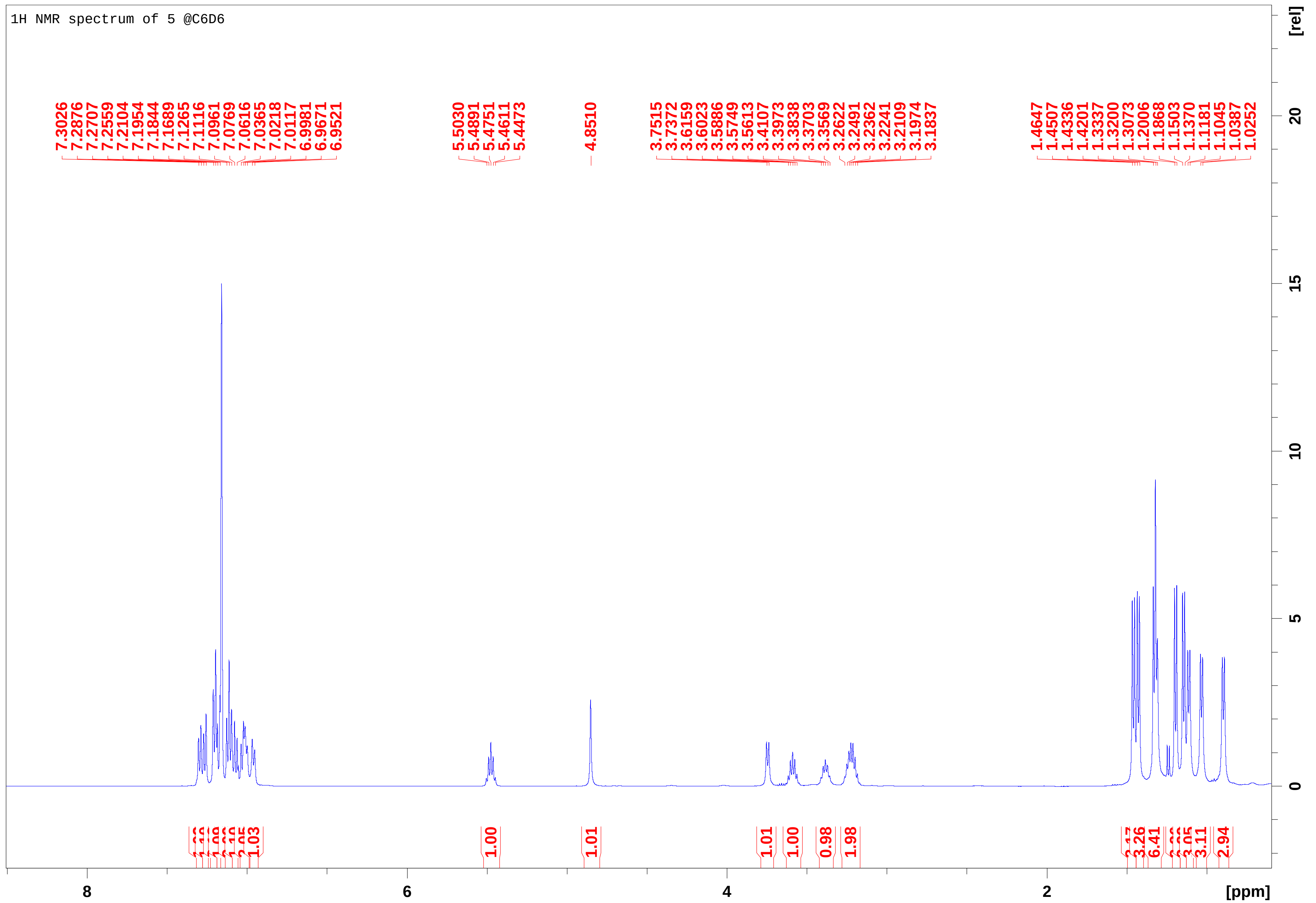

Figure S94. 1H NMR spectrum of 5 in C6D6

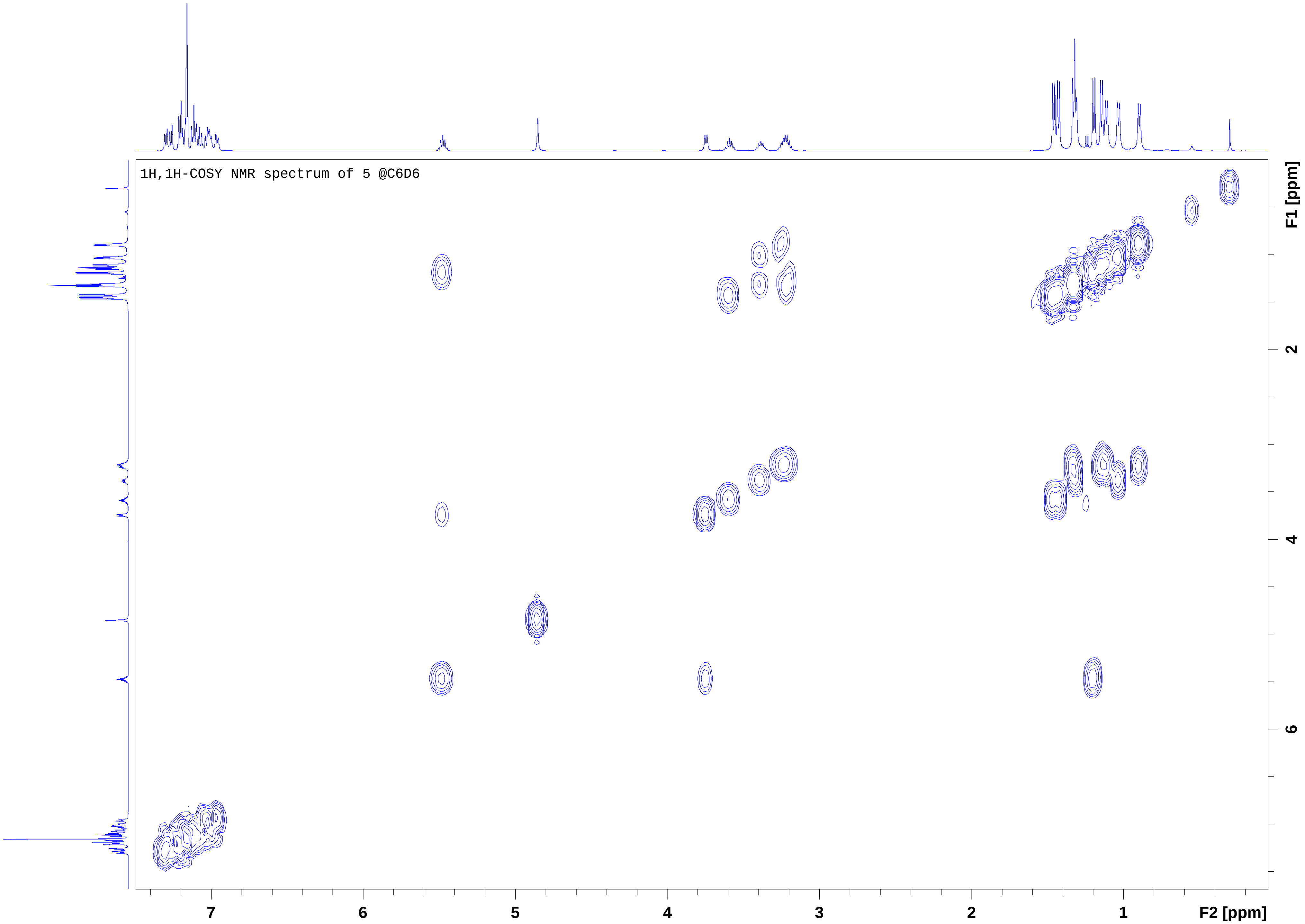

Figure S95. 1H,1H-COSY NMR spectrum of 5 in C6D6

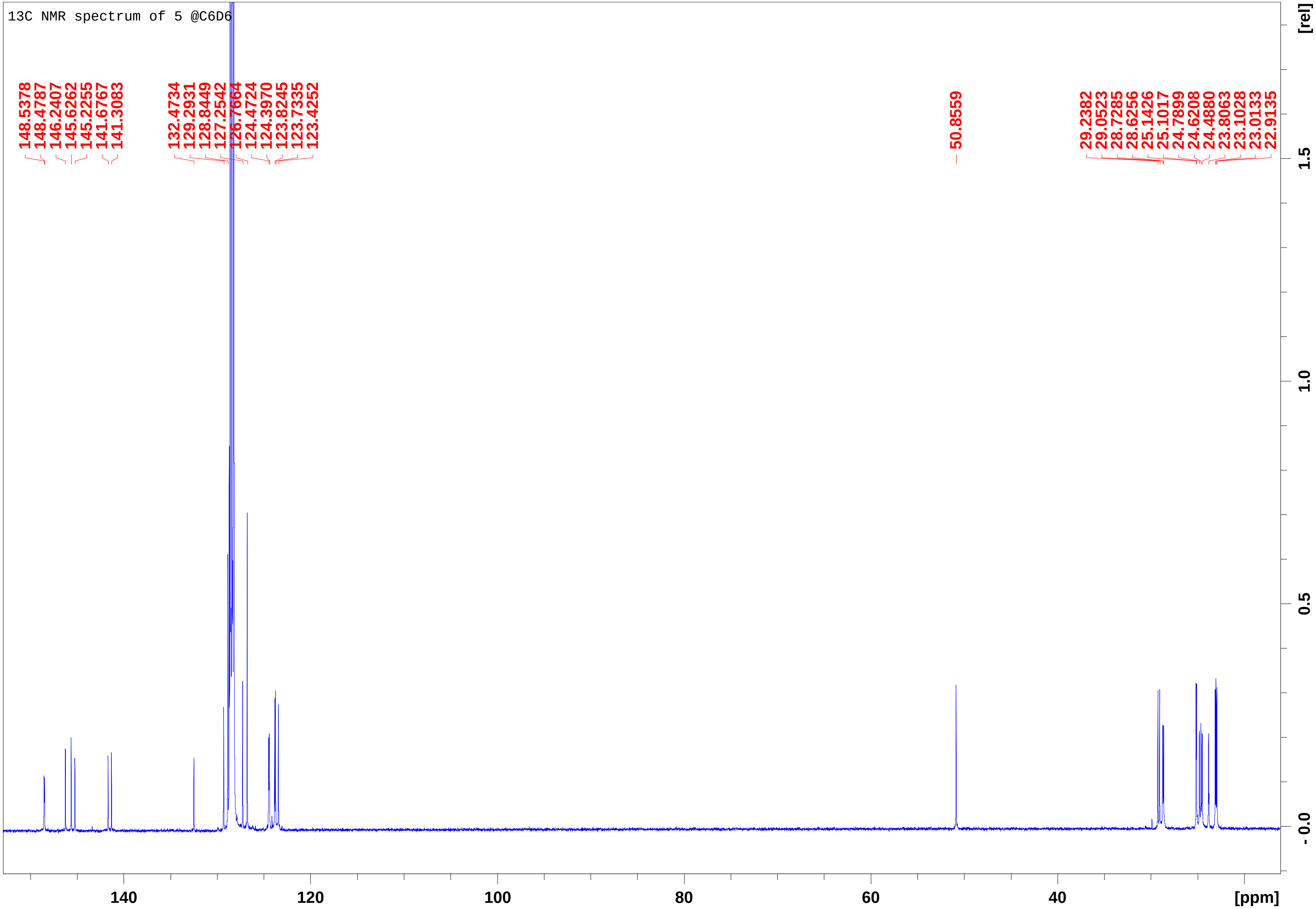

Figure S96. 13C NMR spectrum of 5 in C6D6

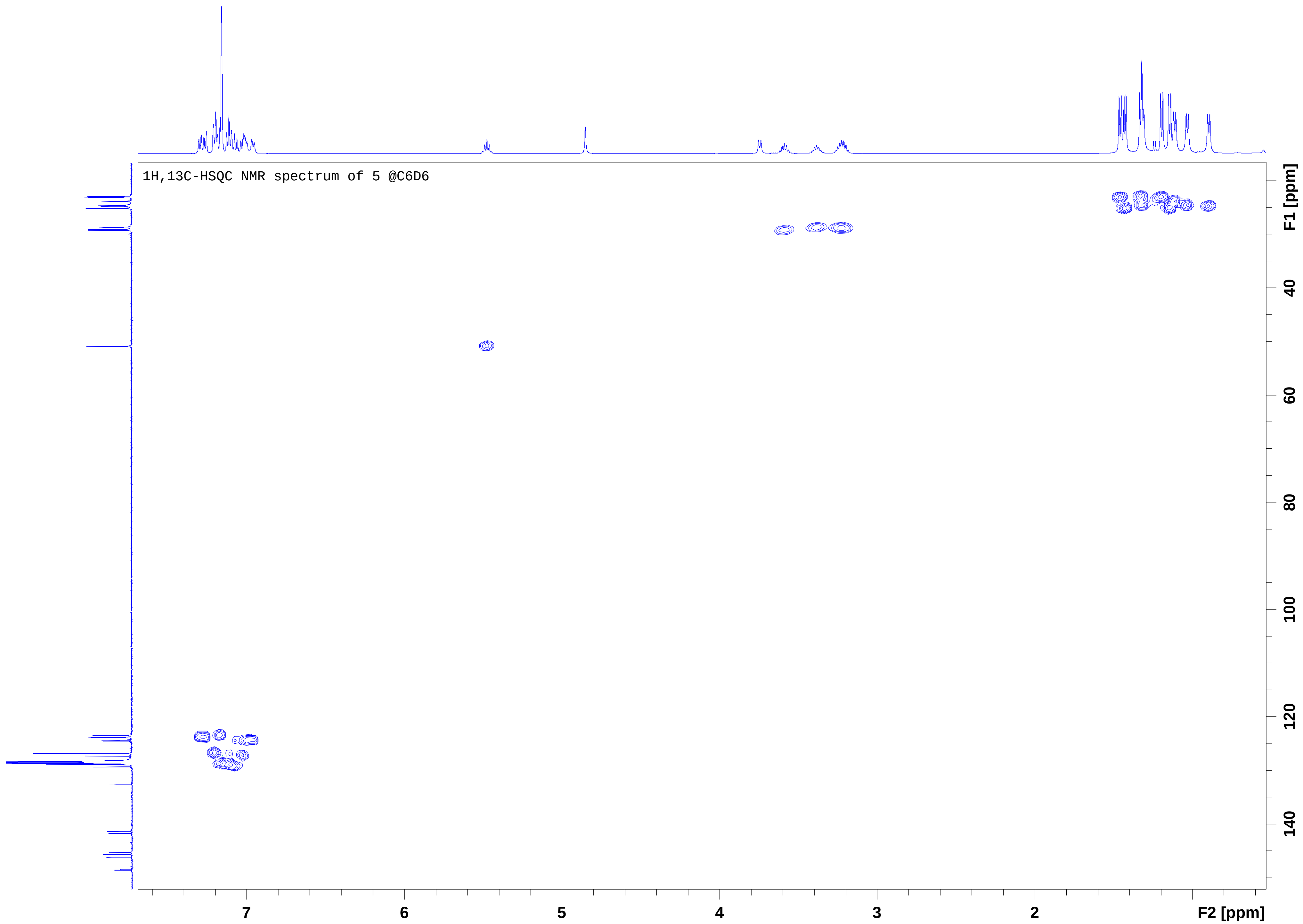

Figure S97. 1H,13C-HSQC NMR spectrum of 5 in C6D6

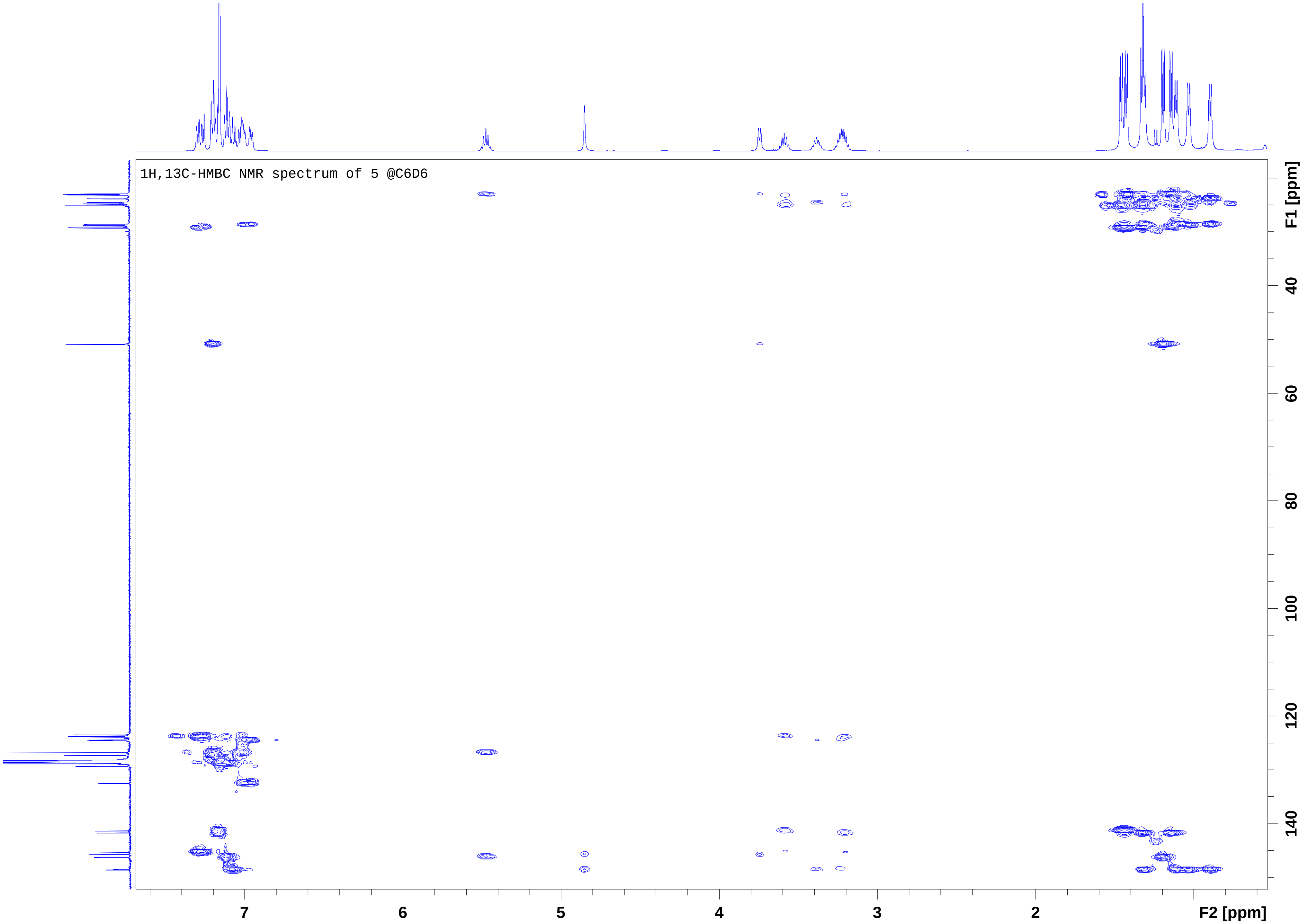

Figure S98. 1H,13C-HMBC NMR spectrum of 5 in C6D6

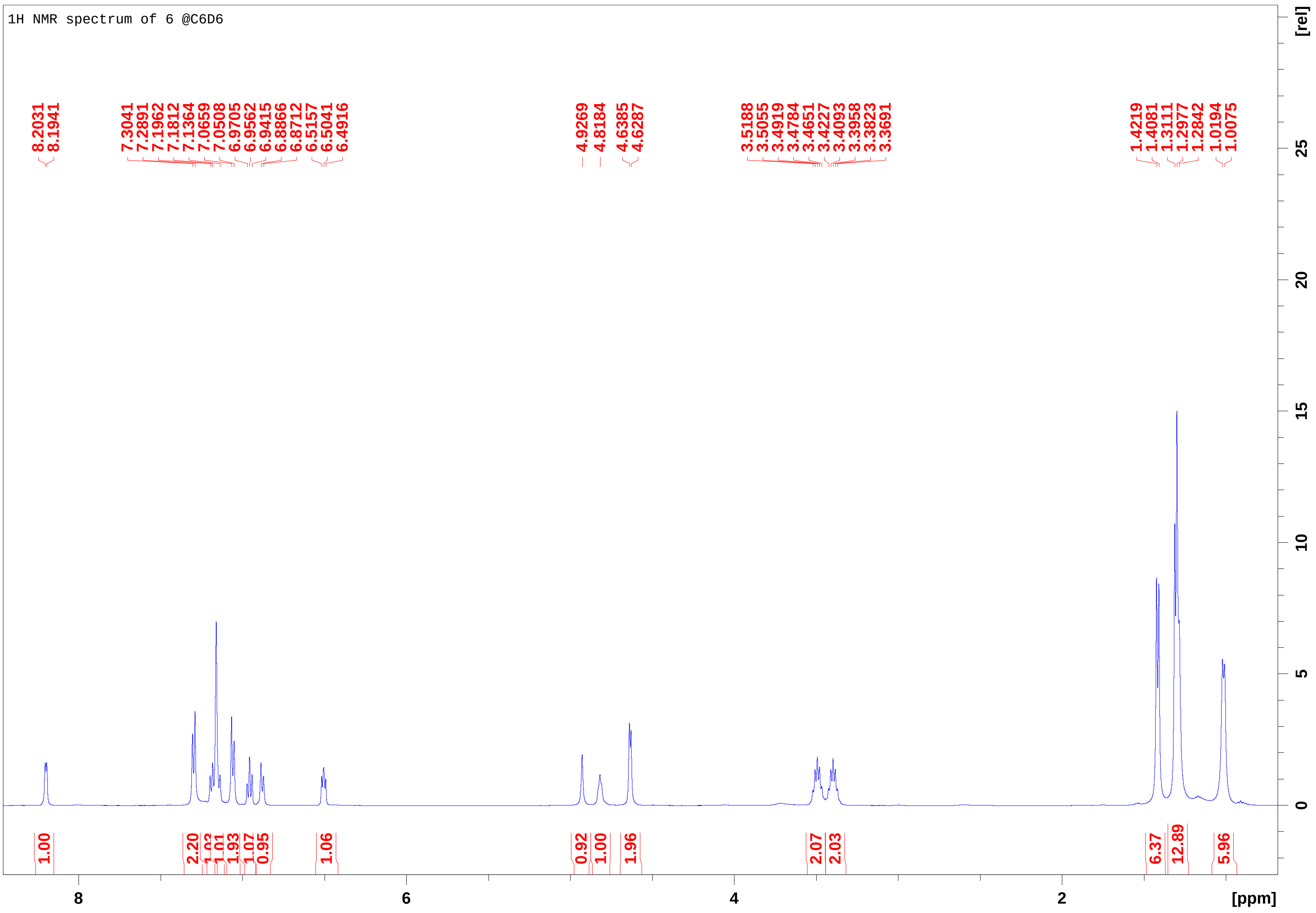

Figure S99. 1H NMR spectrum of 6 in C6D6

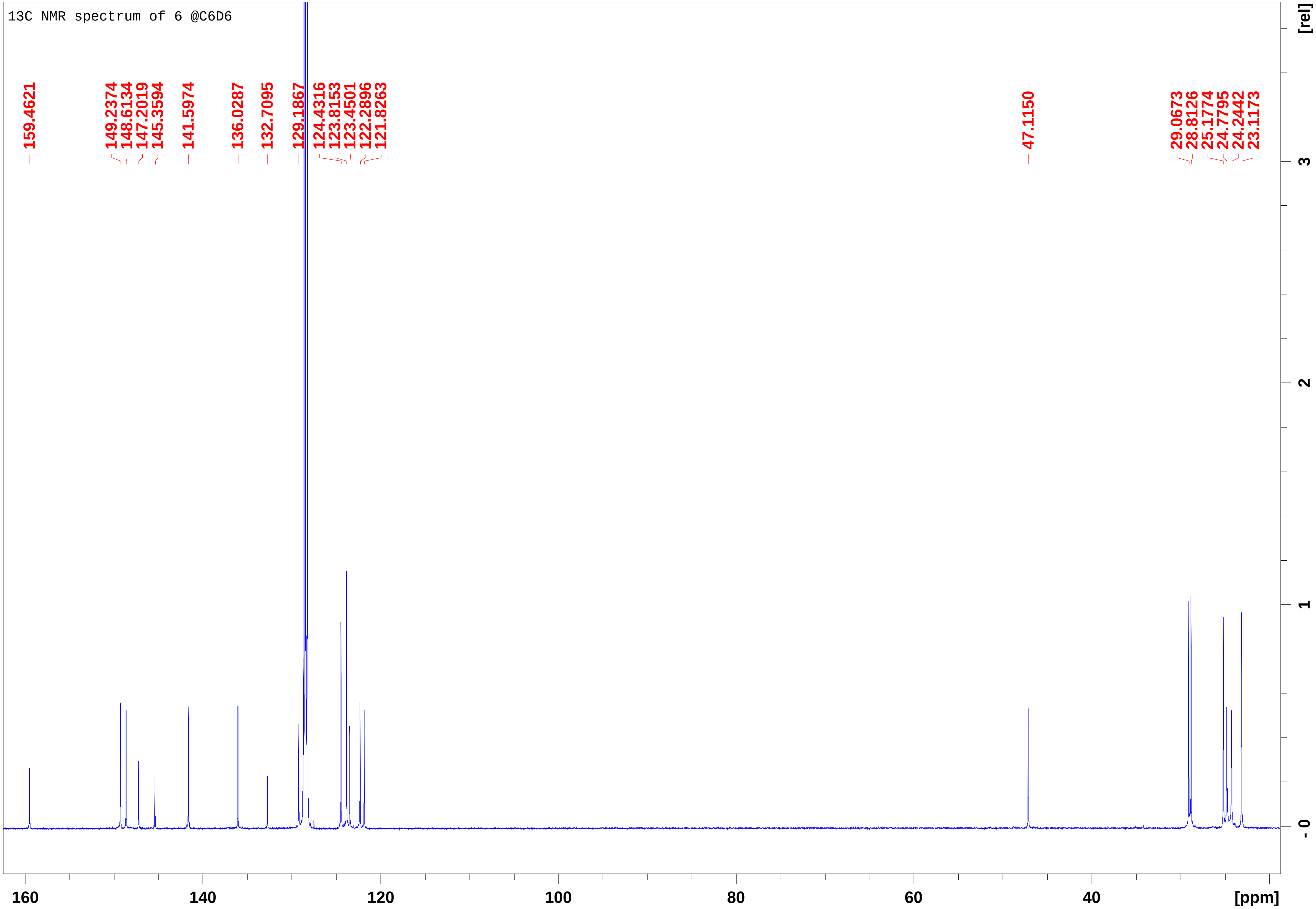

Figure S100. 13C NMR spectrum of 6 in C6D6

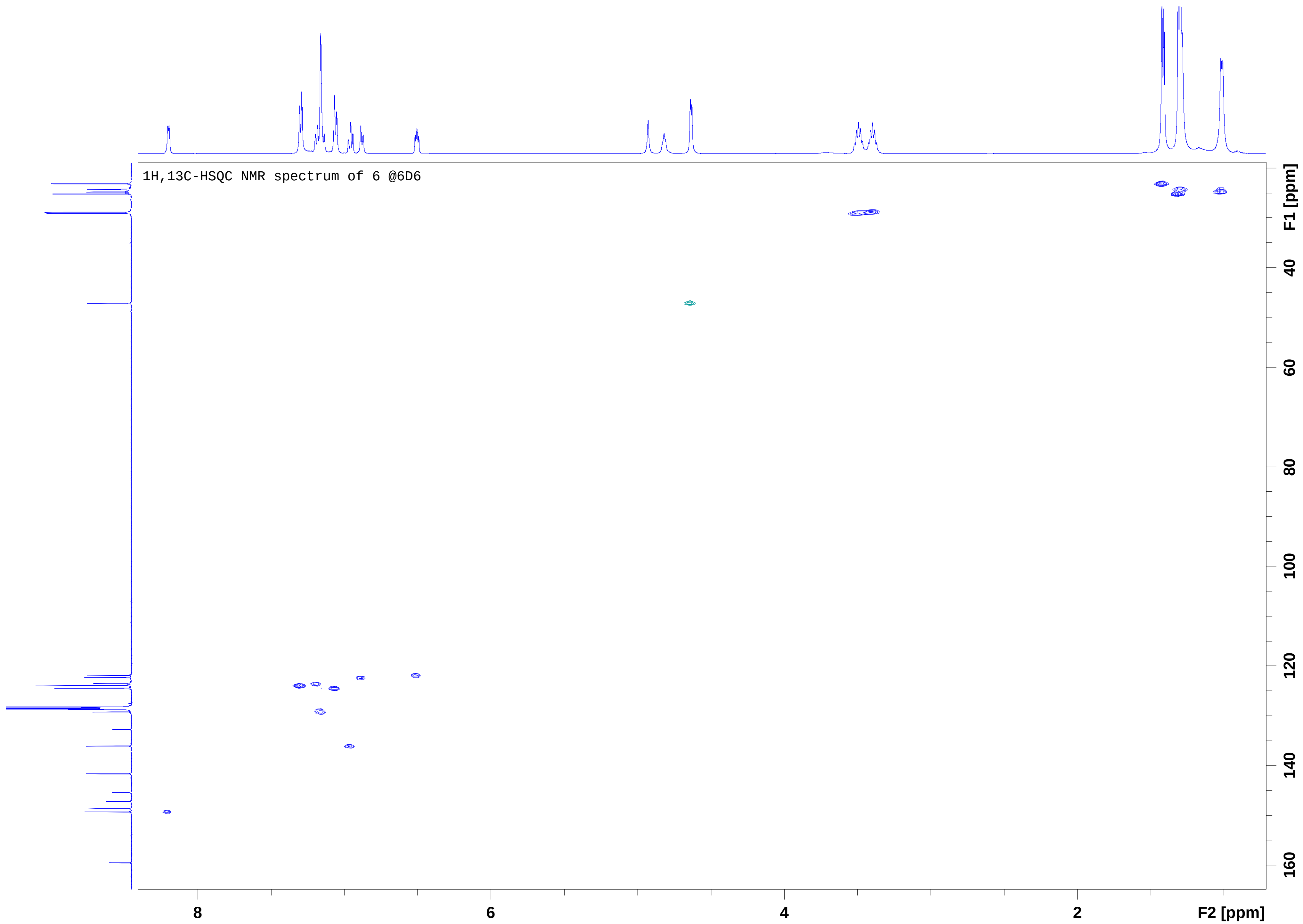

Figure S101. 1H,13C-HSQC NMR spectrum of 6 in C6D6

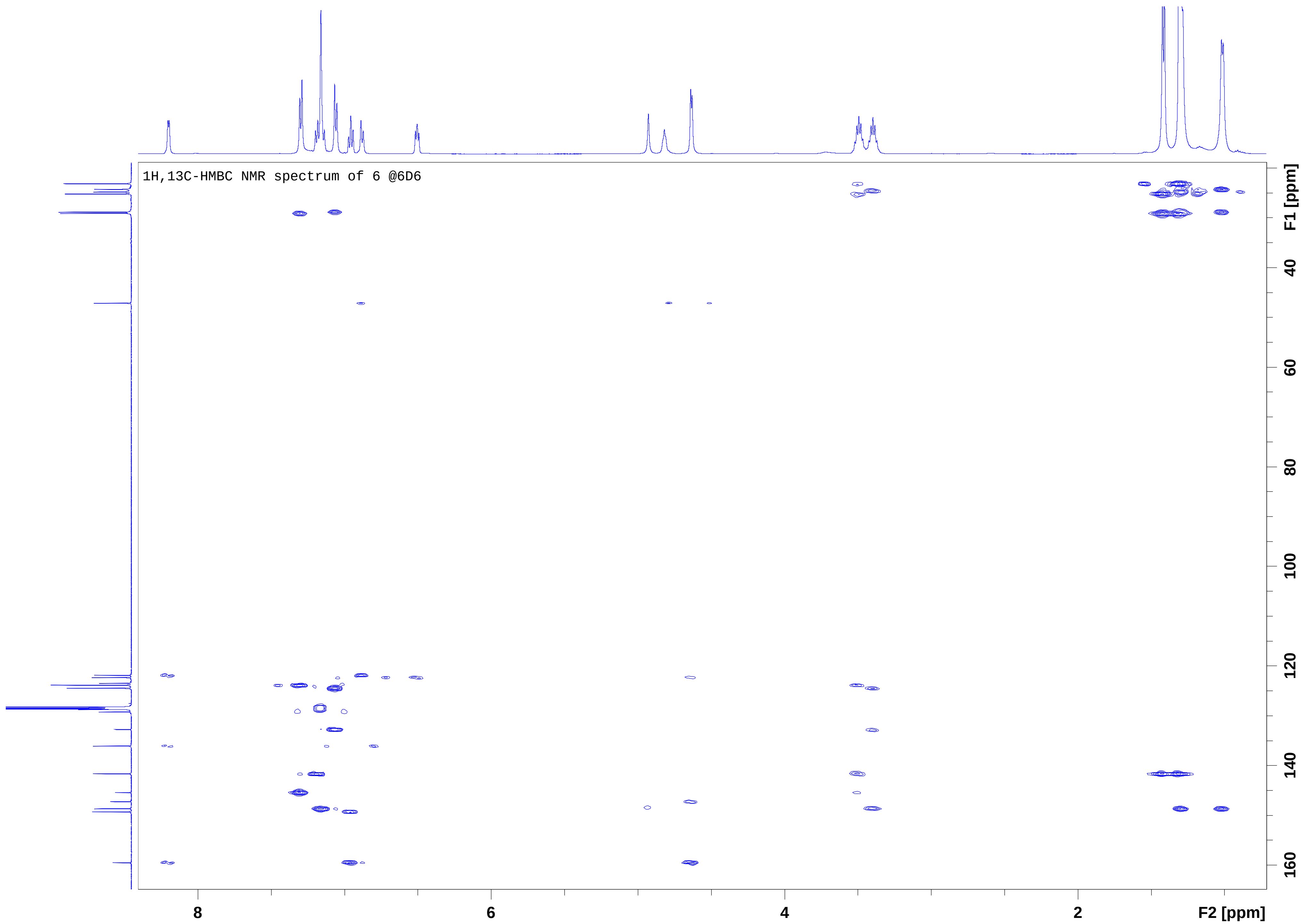

Figure S102. 1H,13C-HMBC NMR spectrum of 6 in C6D6

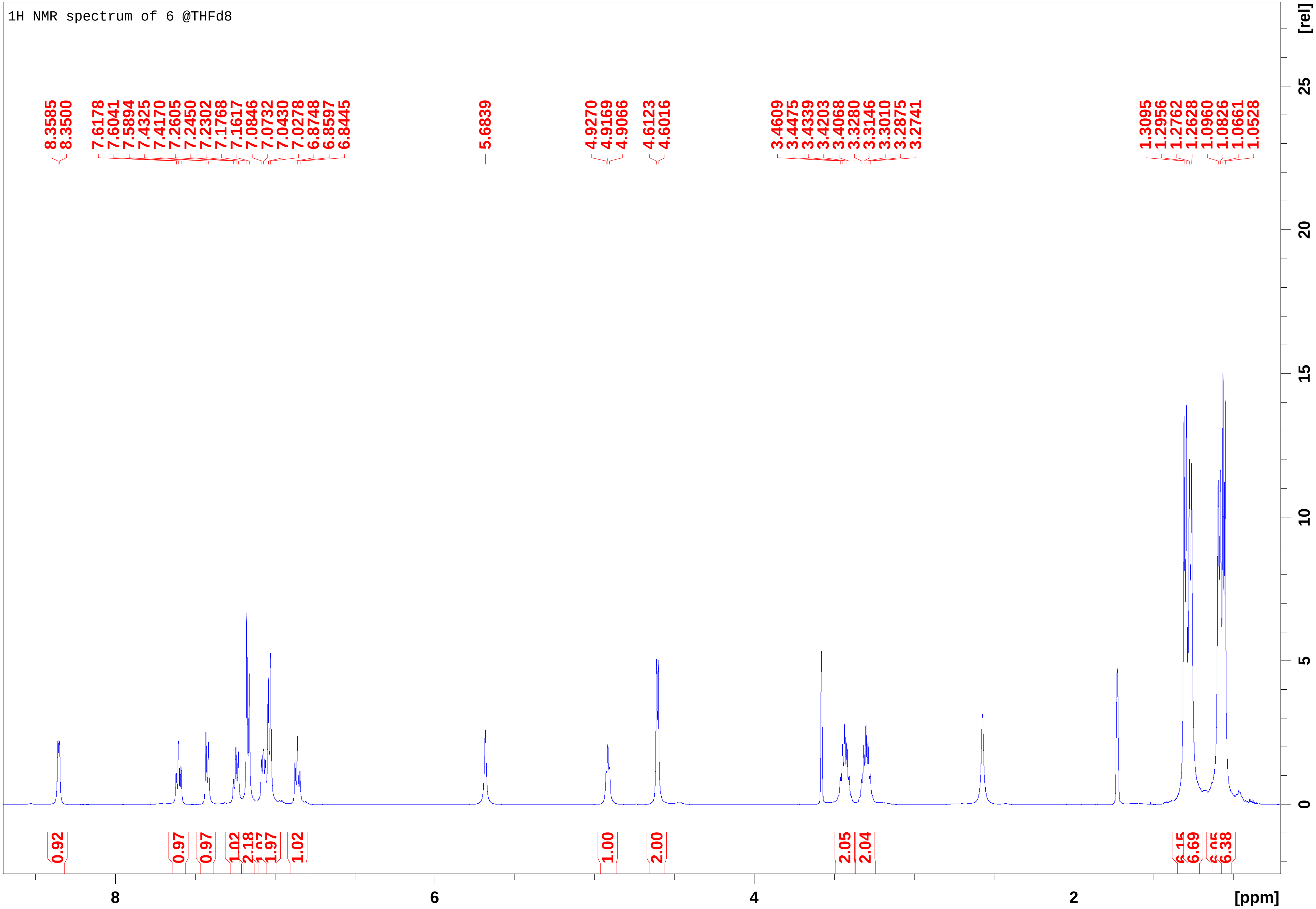

Figure S103. 1H NMR spectrum of 6 in THF-d8

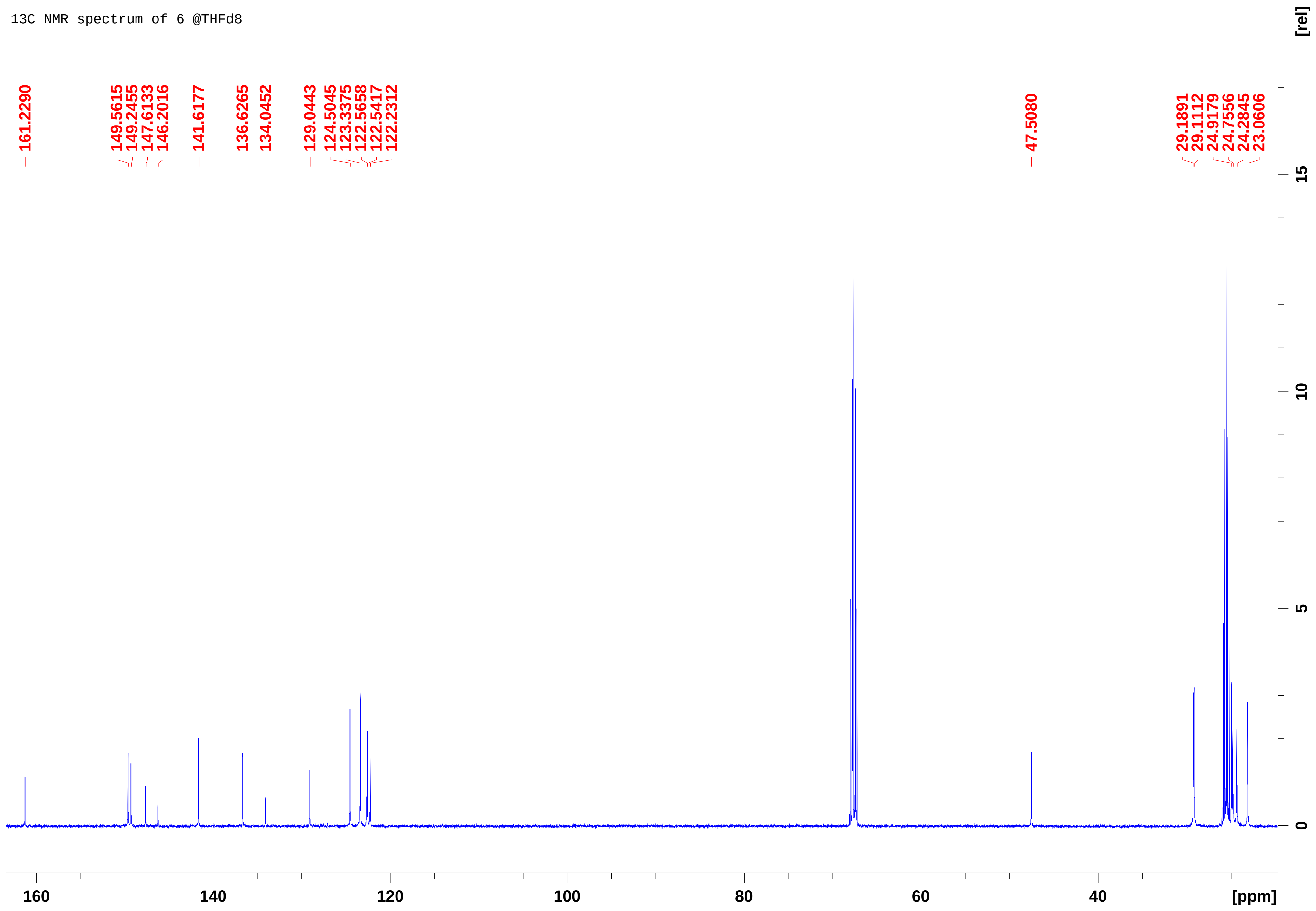

Figure S104. 13C NMR spectrum of 6 in THF-d8

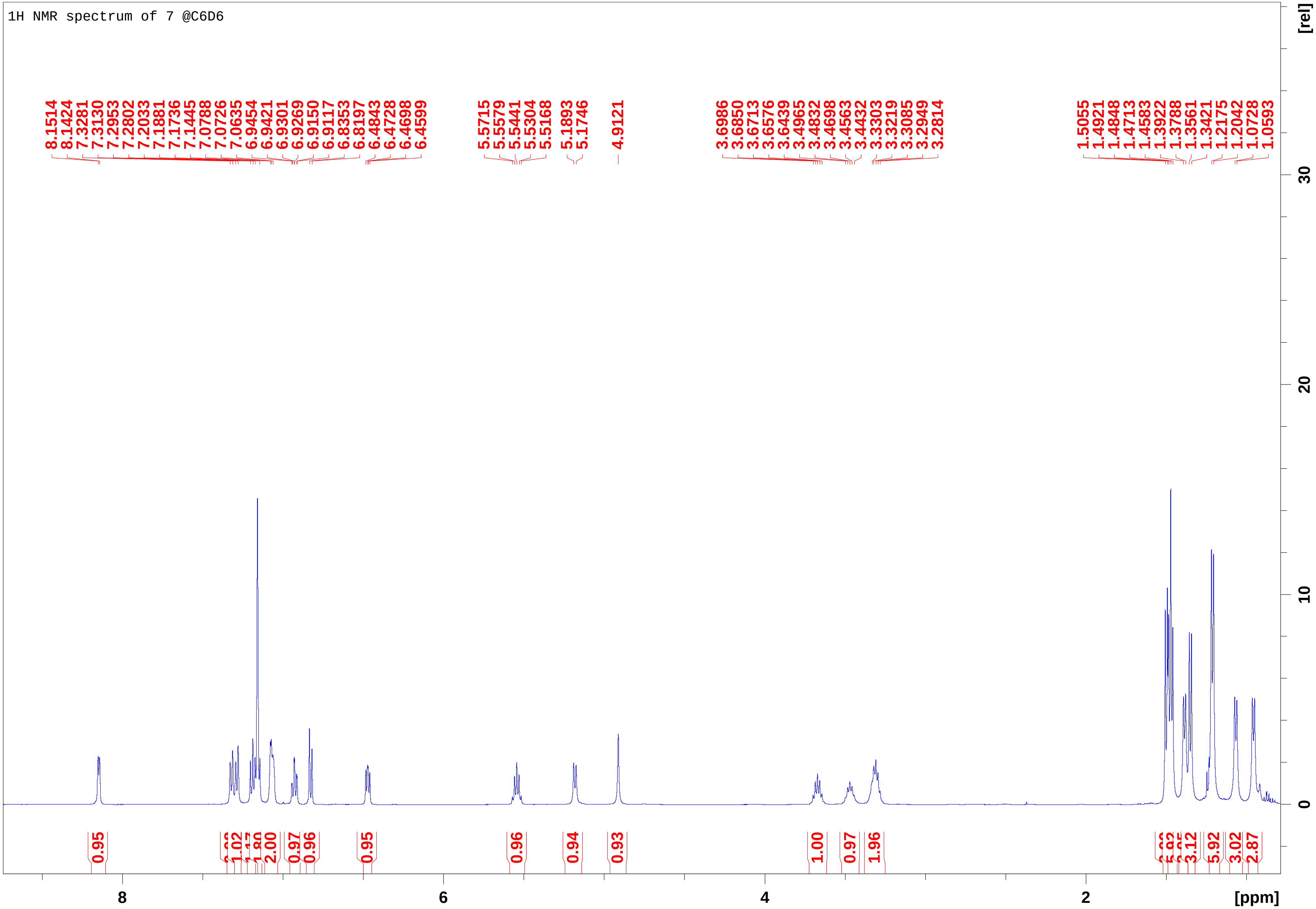

Figure S105. 1H NMR spectrum of 7 in C6D6

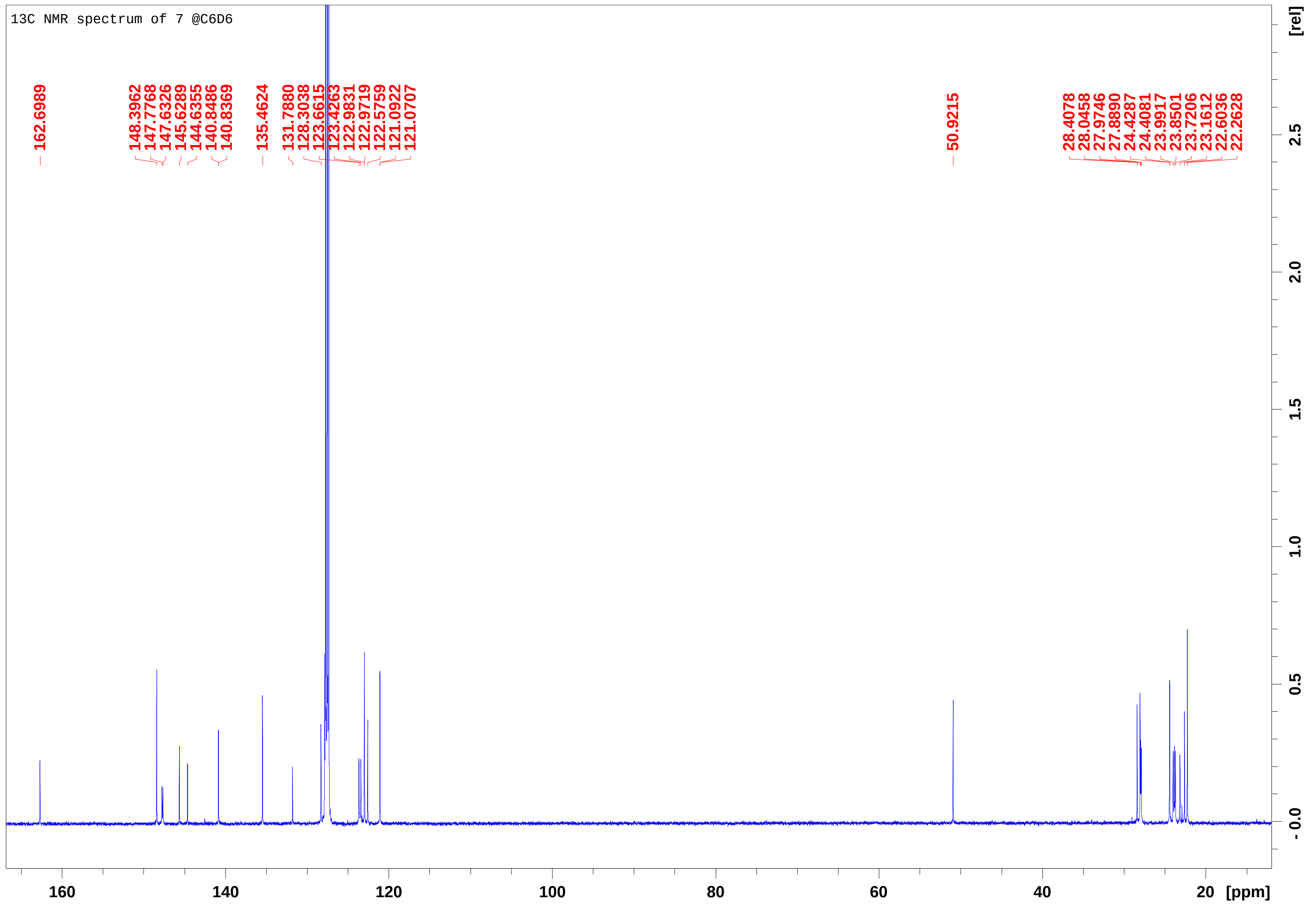

Figure S106. 13C NMR spectrum of 7 in C6D6

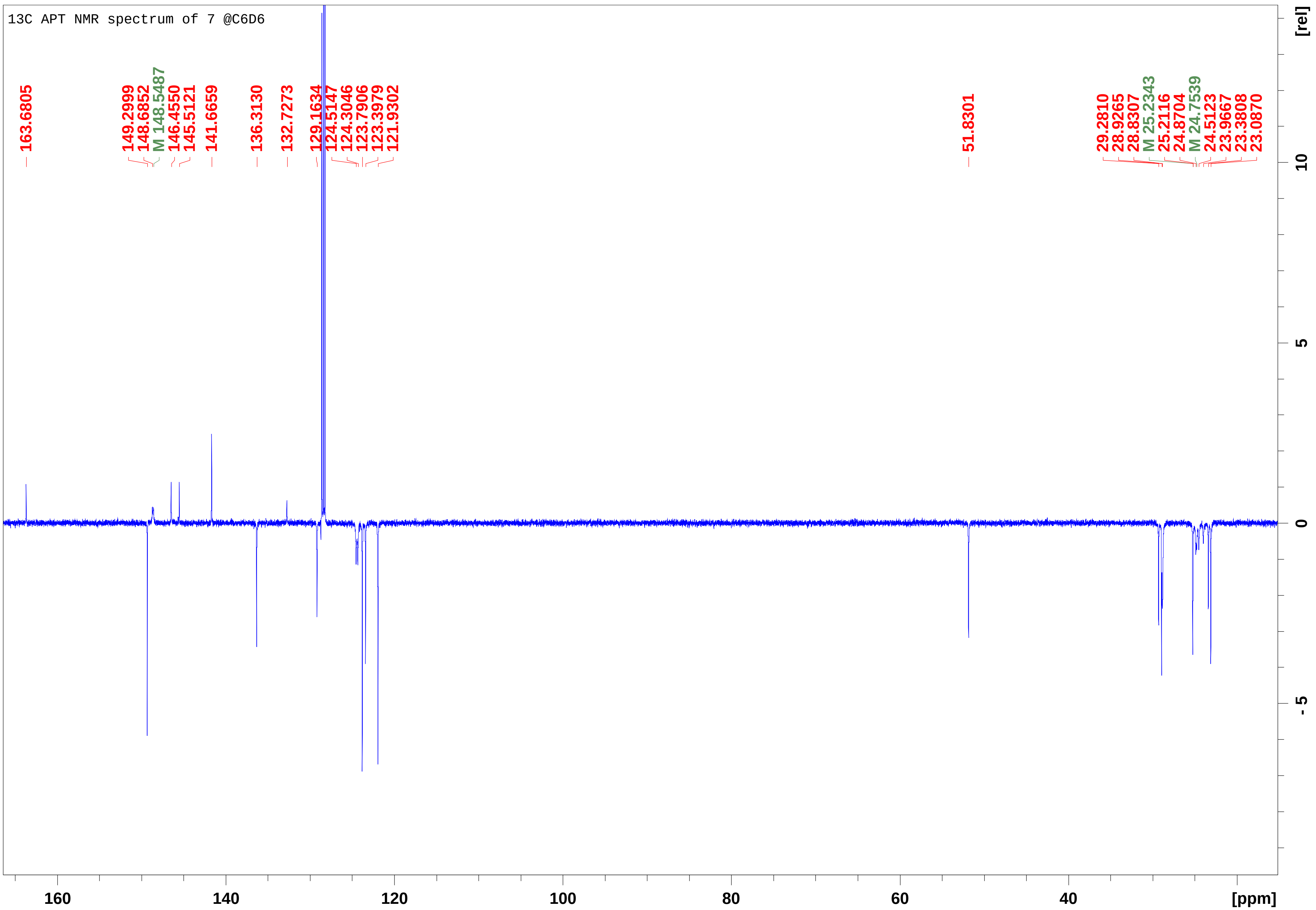

Figure S107. 13C APT NMR spectrum of 7 in C6D6

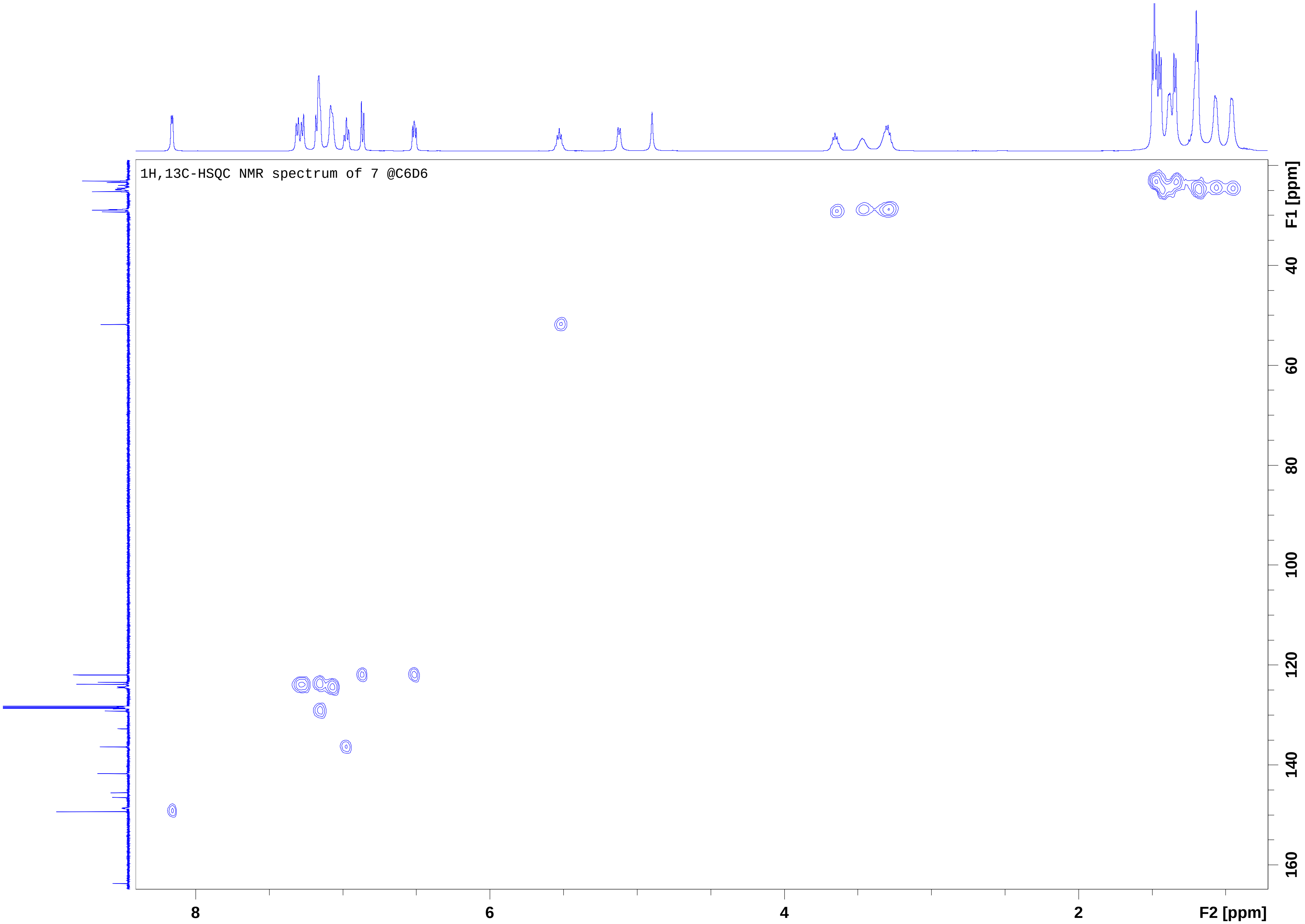

Figure S108. 1H,13C-HSQC NMR spectrum of 7 in C6D6

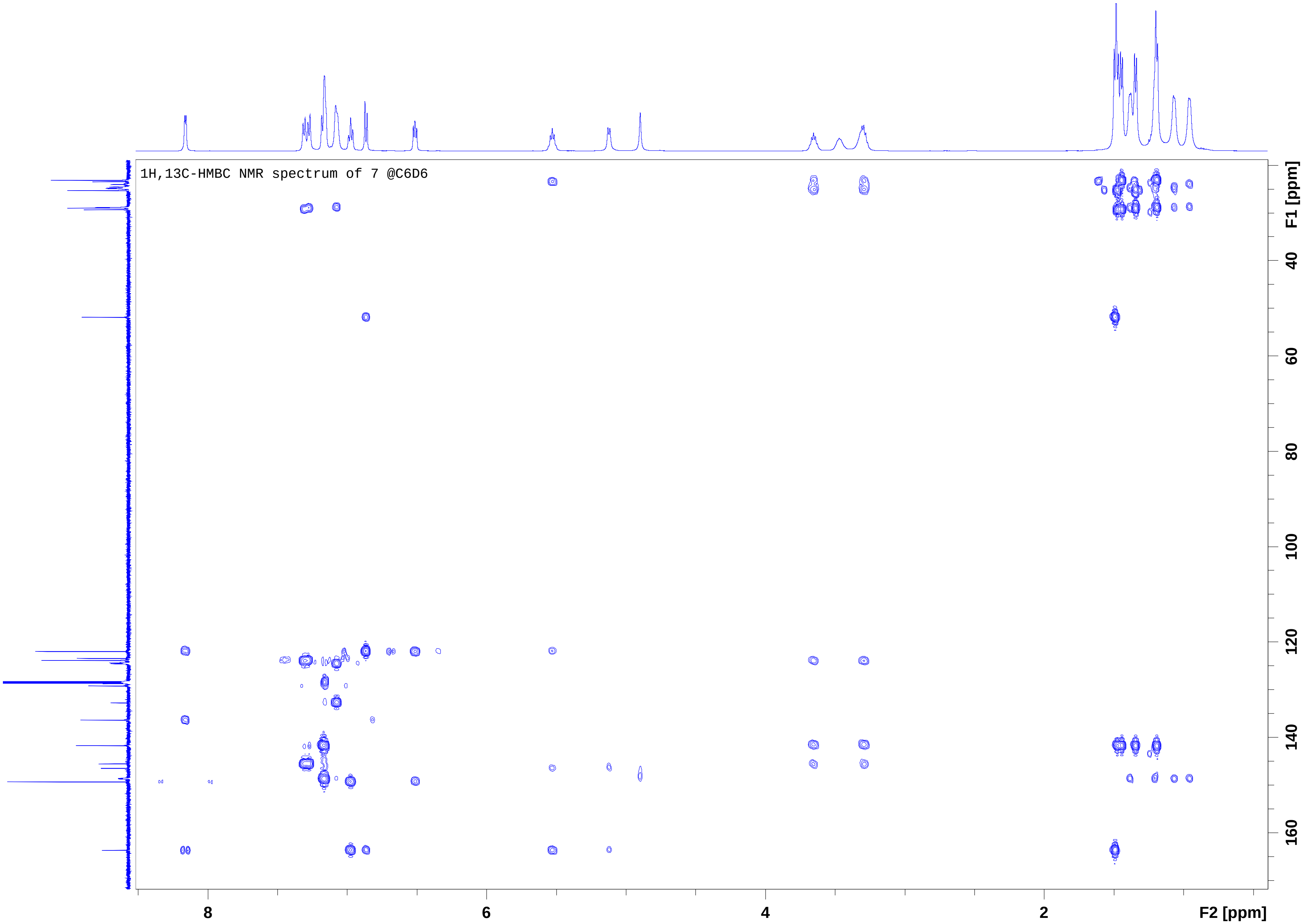

Figure S109. 1H,13C-HMBC NMR spectrum of 7 in C6D6

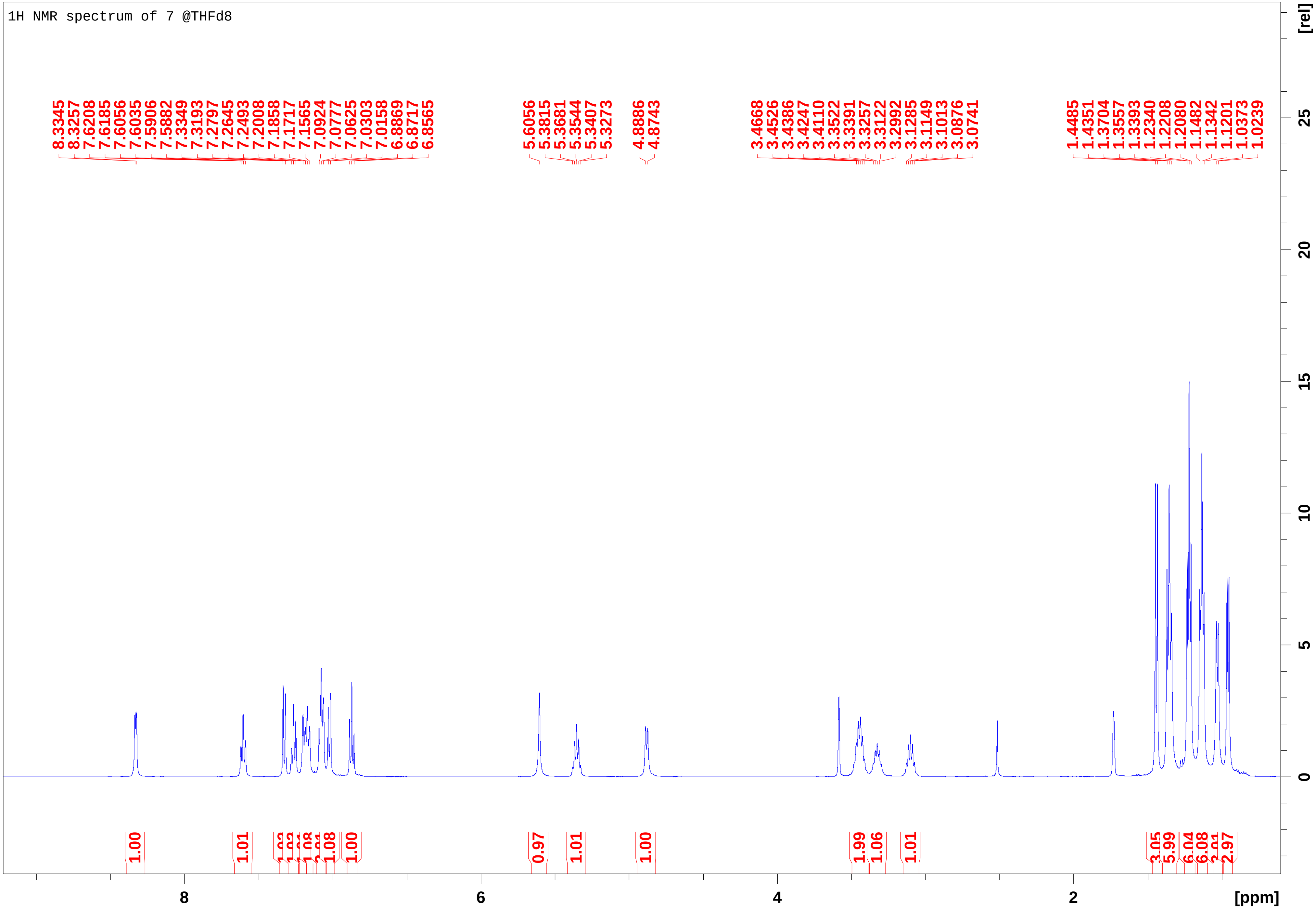

Figure S110. 1H NMR spectrum of 7 in THF-d8

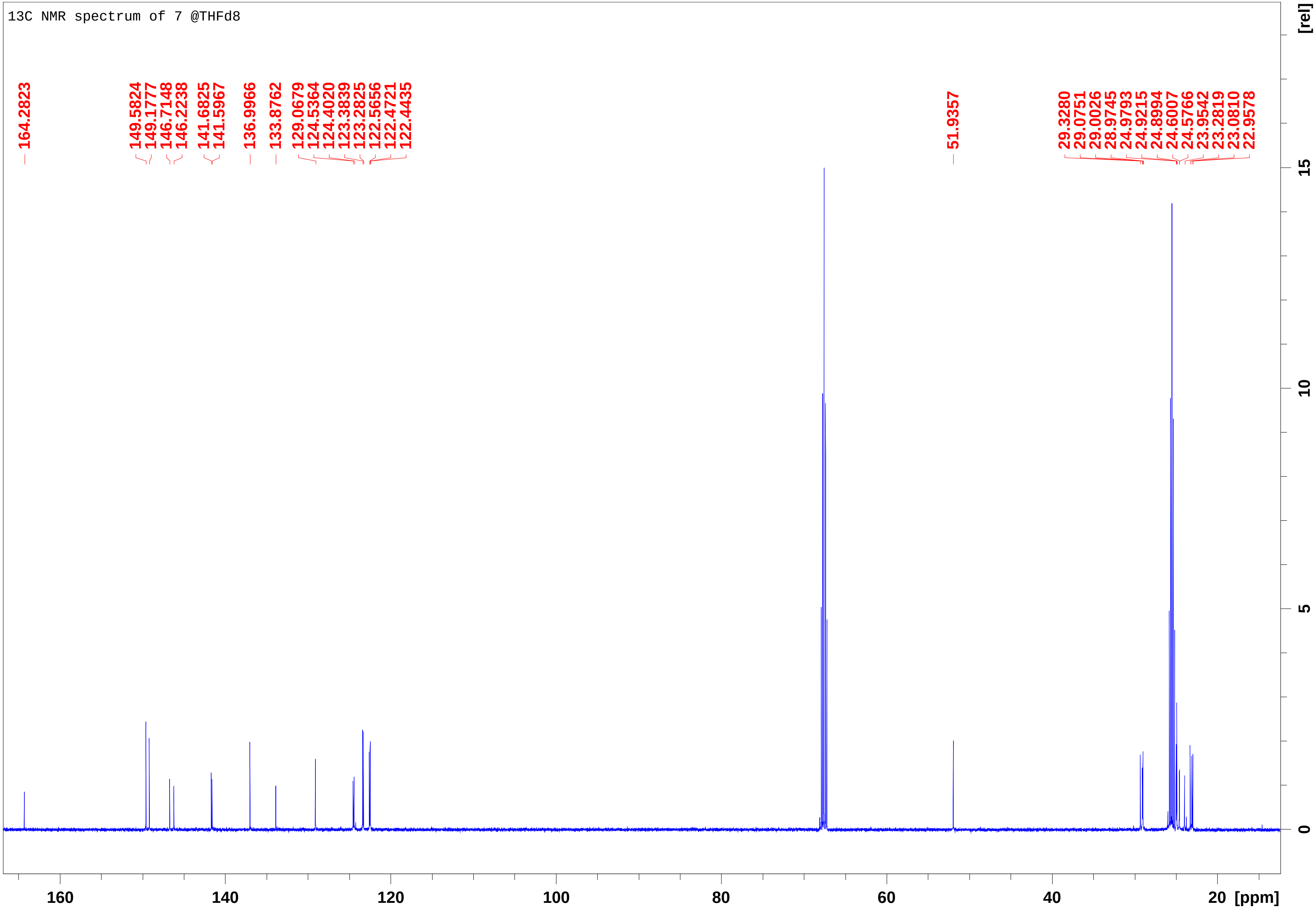

Figure S111. 13C NMR spectrum of 7 in THF-d8

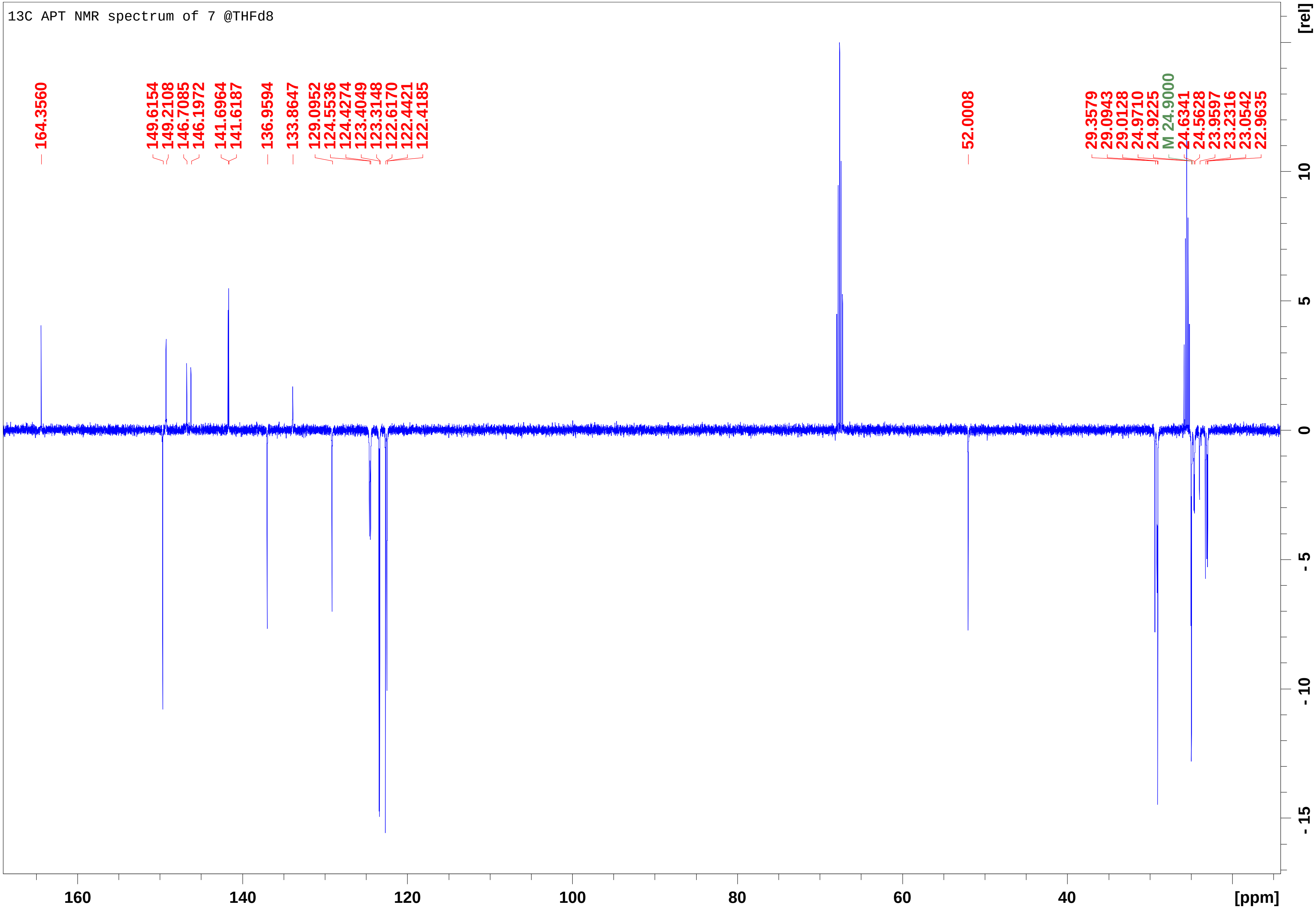

Figure S112. 13C APT NMR spectrum of 7 in THF-d8

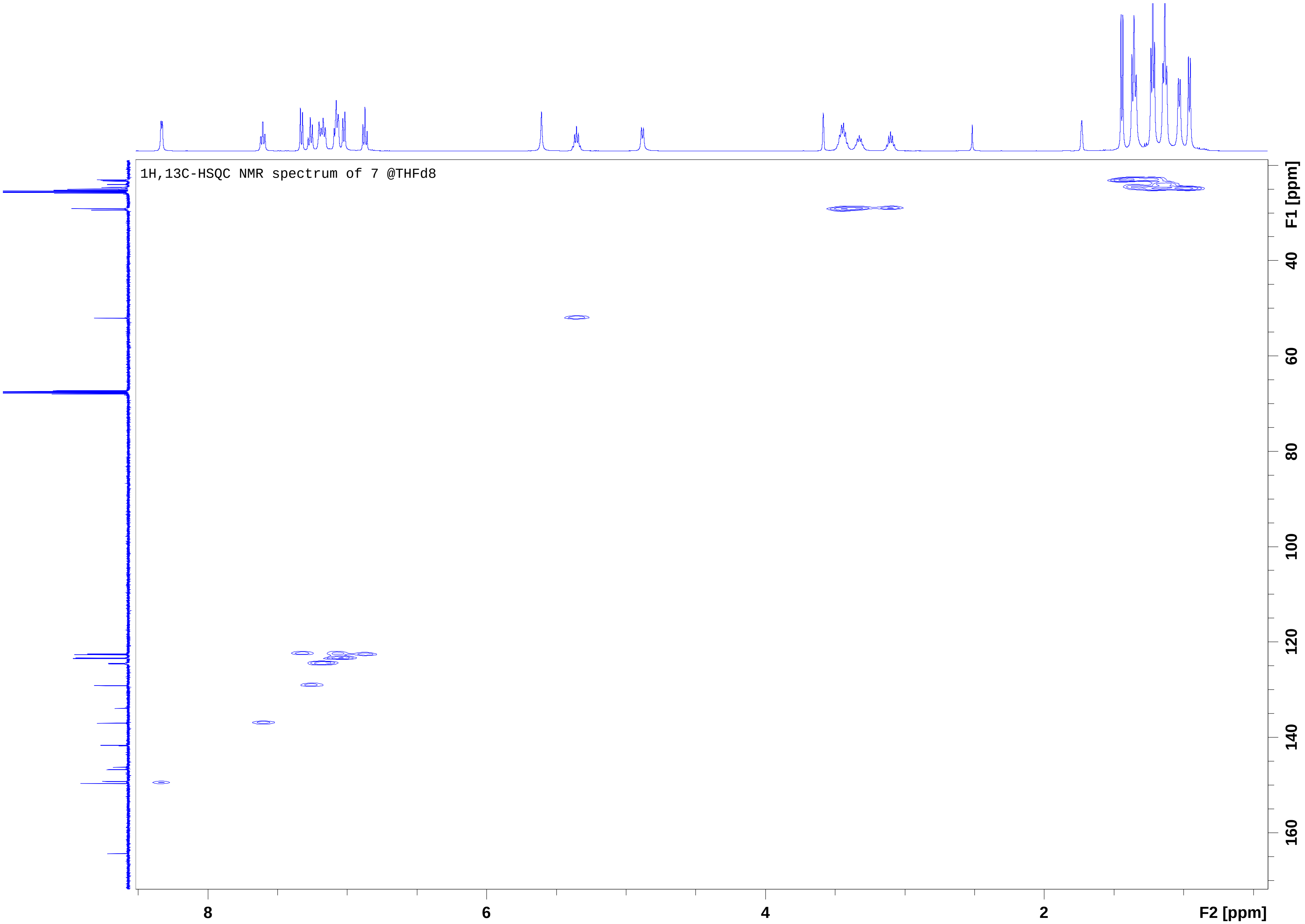

Figure S113. 1H,13C-HSQC NMR spectrum of 7 in THF-d8

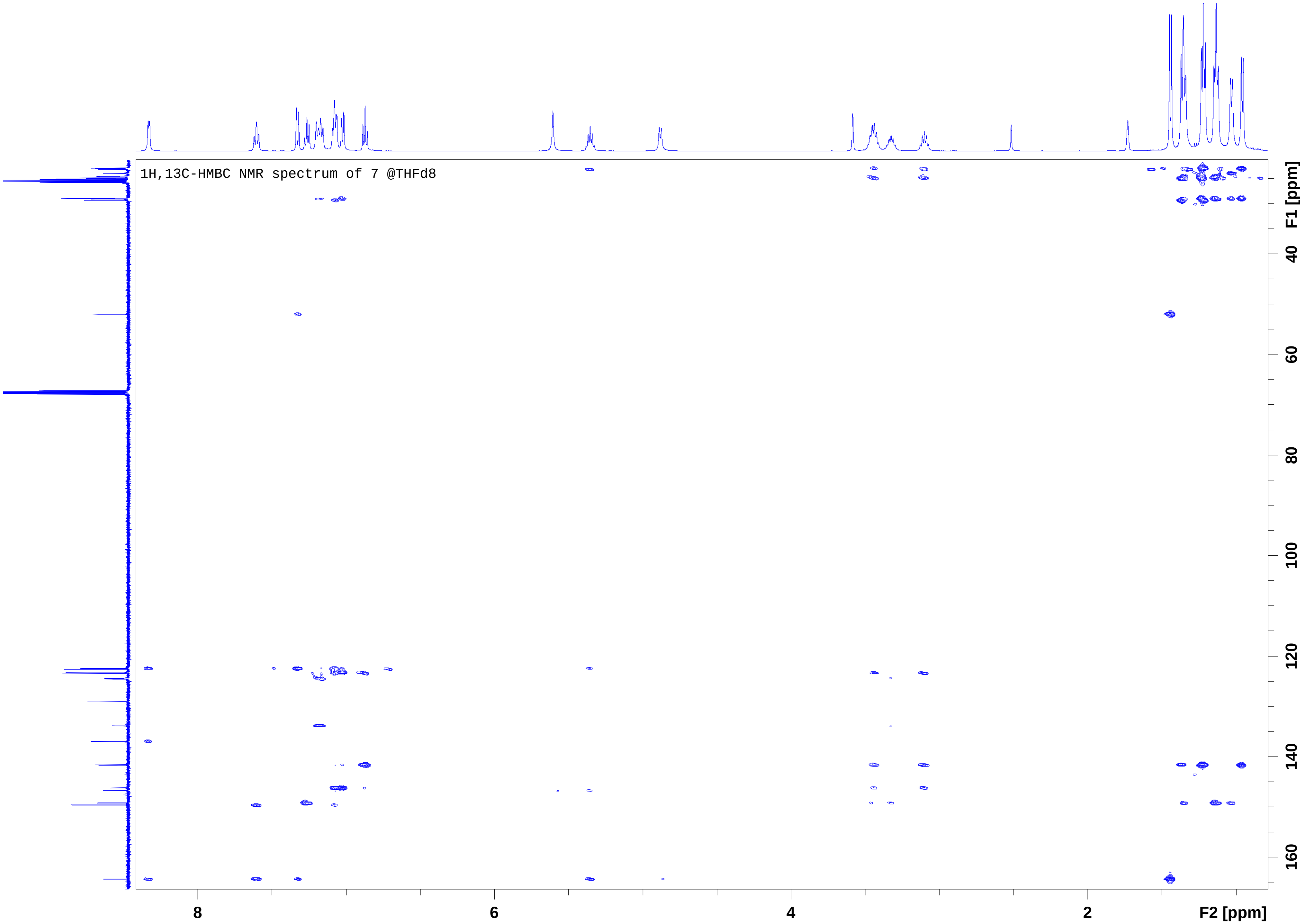

Figure S114. 1H,13C-HMBC NMR spectrum of 7 in THF-d8

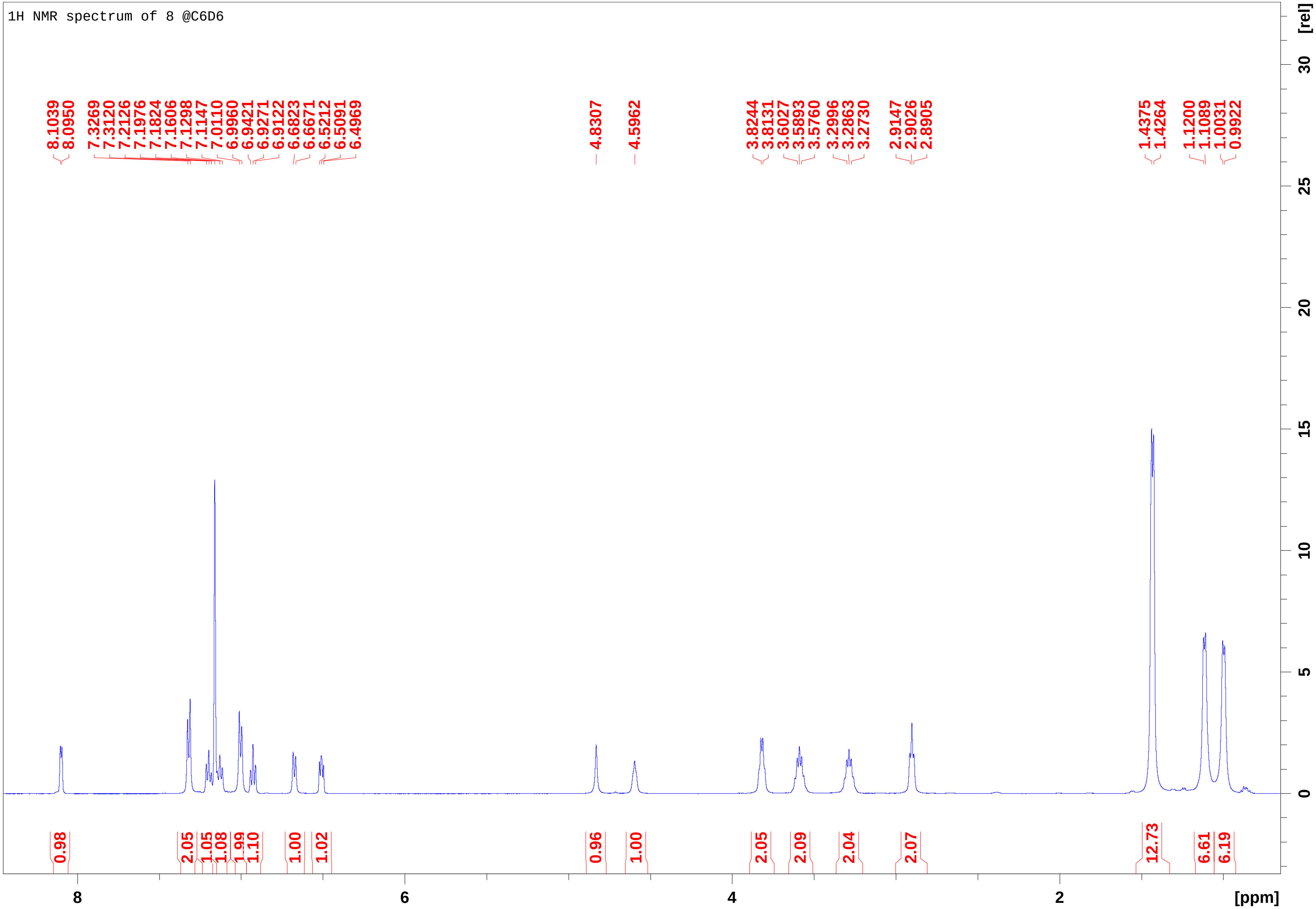

Figure S115. 1H NMR spectrum of 8 in C6D6

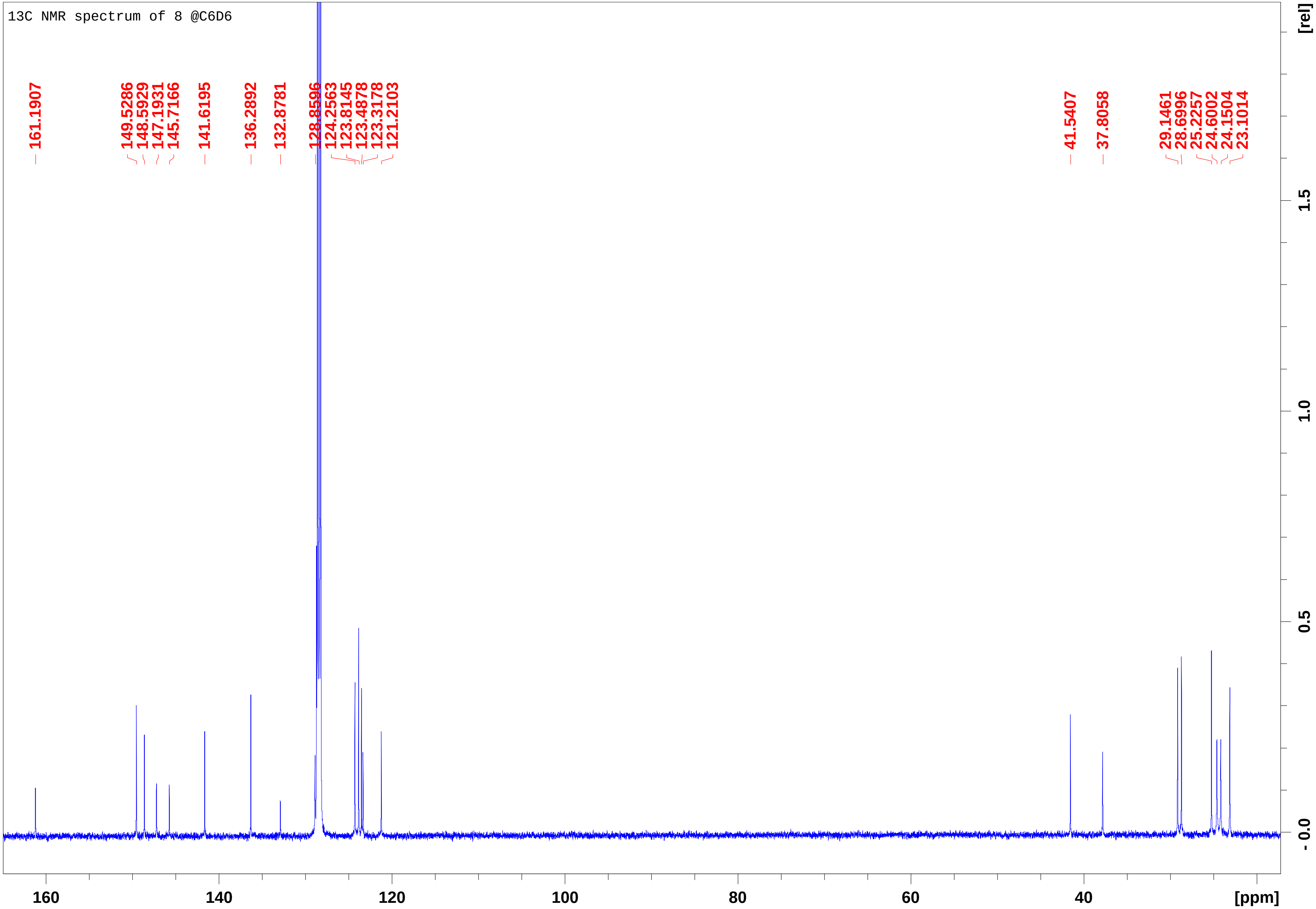

Figure S116. 13C NMR spectrum of 8 in C6D6

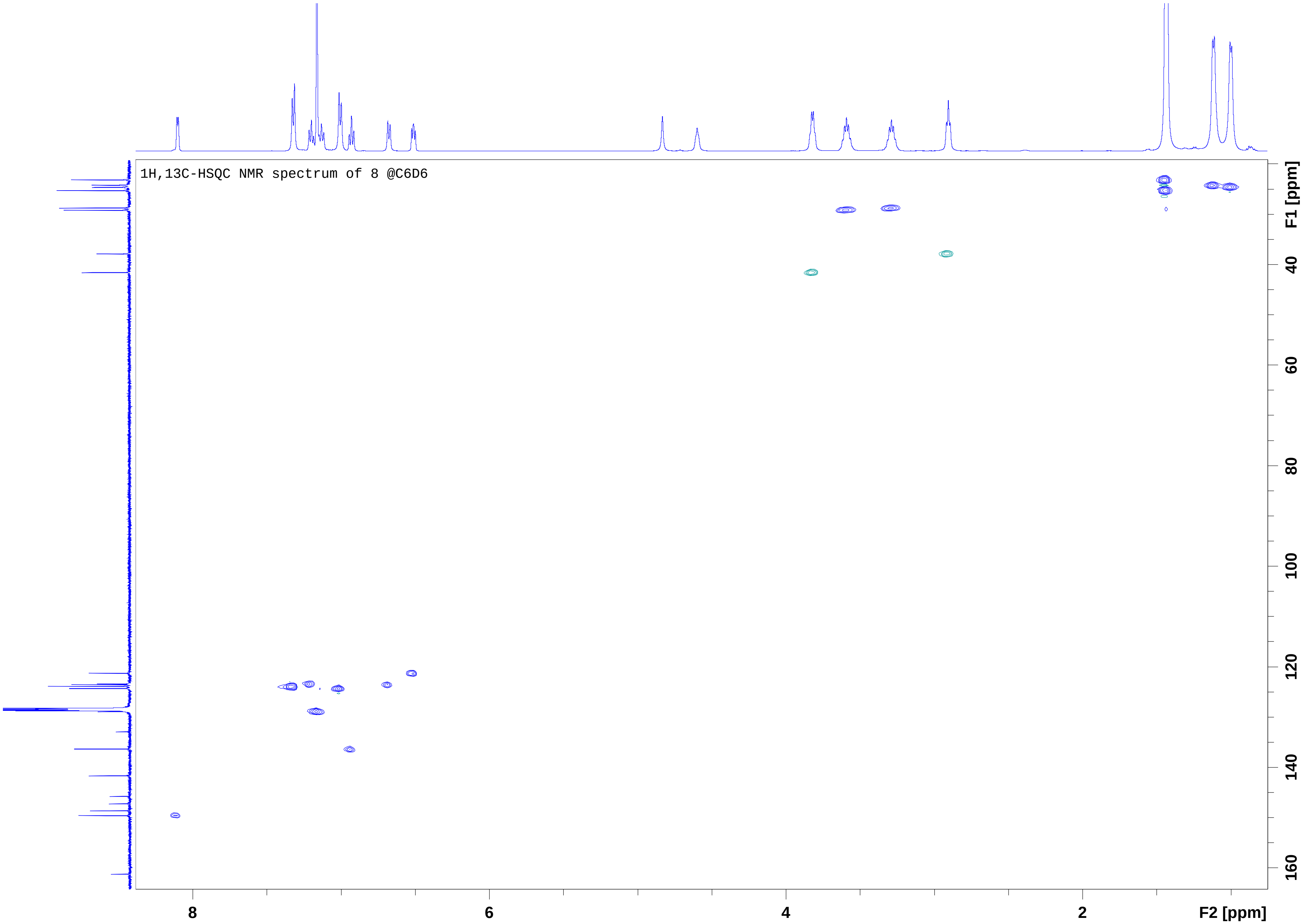

Figure S117. 1H,13C-HSQC NMR spectrum of 8 in C6D6

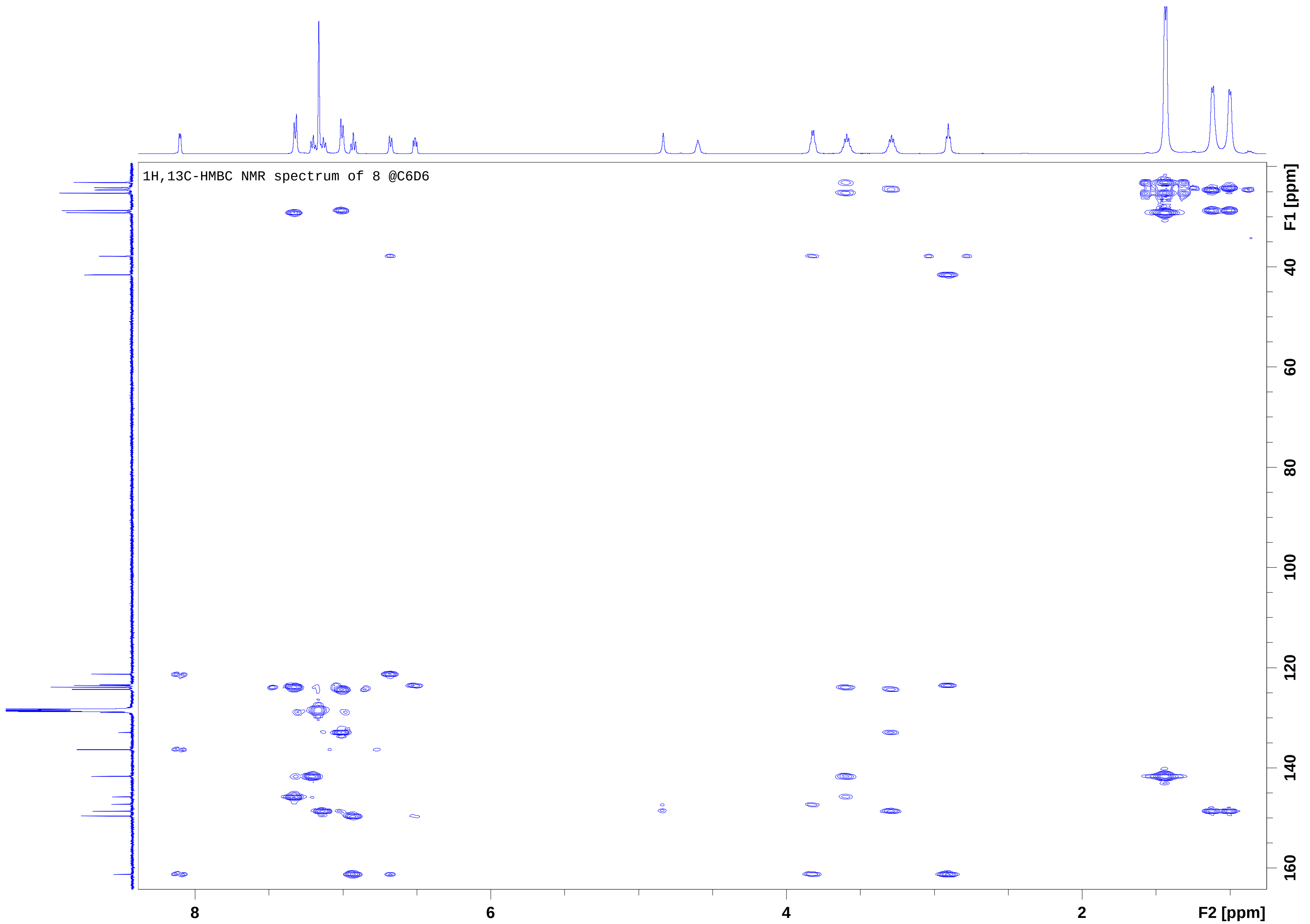

Figure S118. 1H,13C-HMBC NMR spectrum of 8 in C6D6

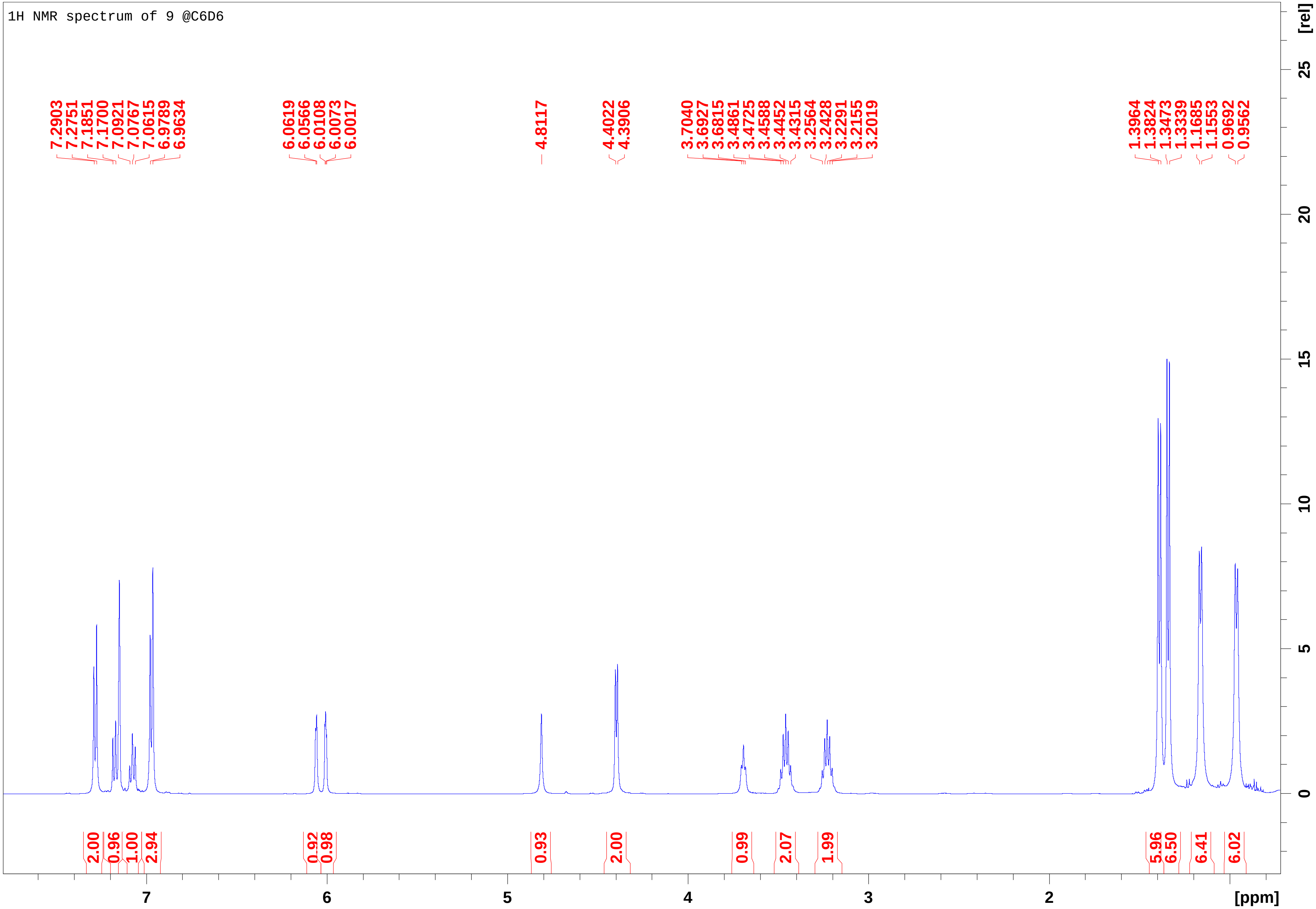

Figure S119. 1H NMR spectrum of 9 in C6D6

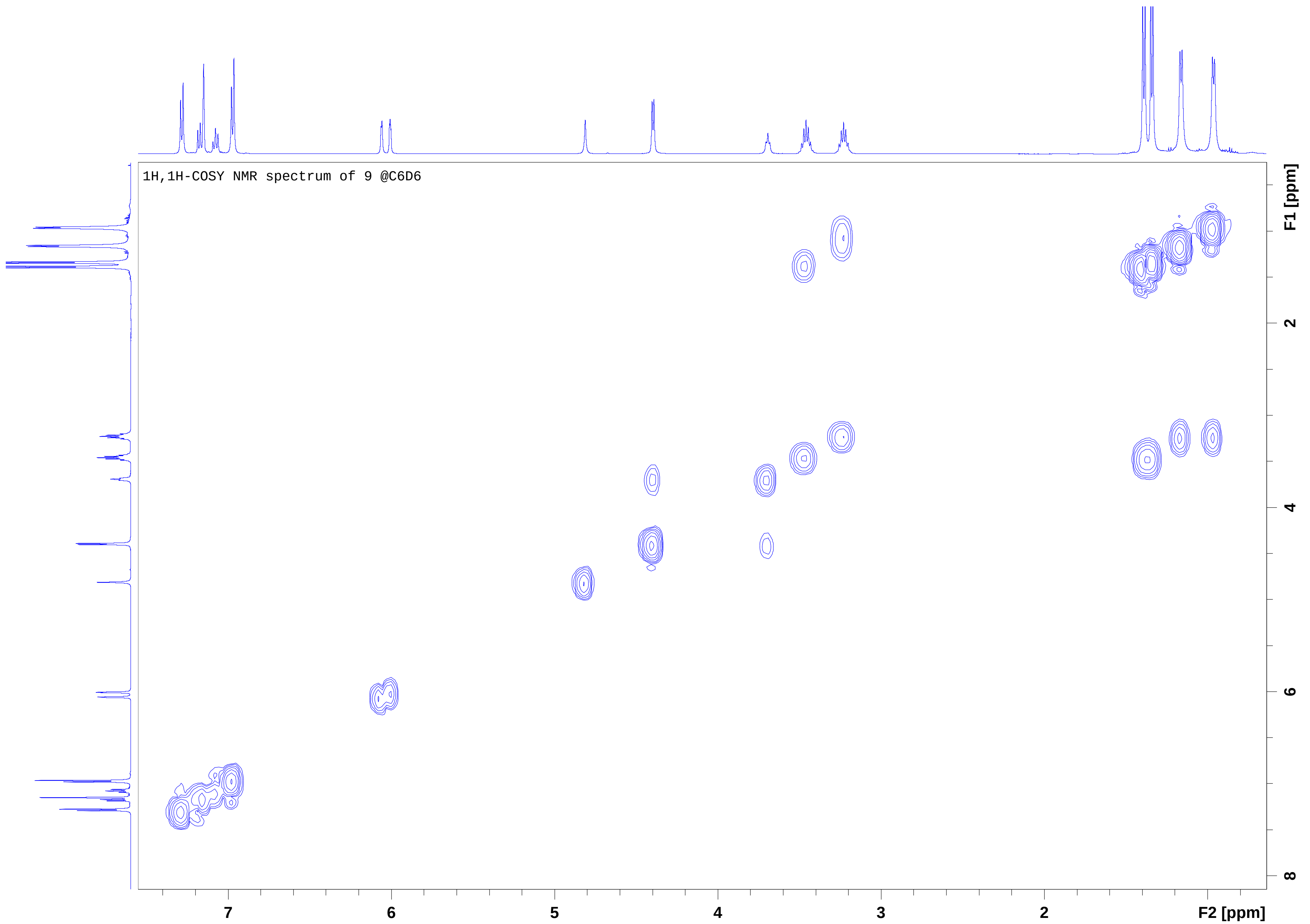

Figure S120. 1H,1H-COSY NMR spectrum of 9 in C6D6

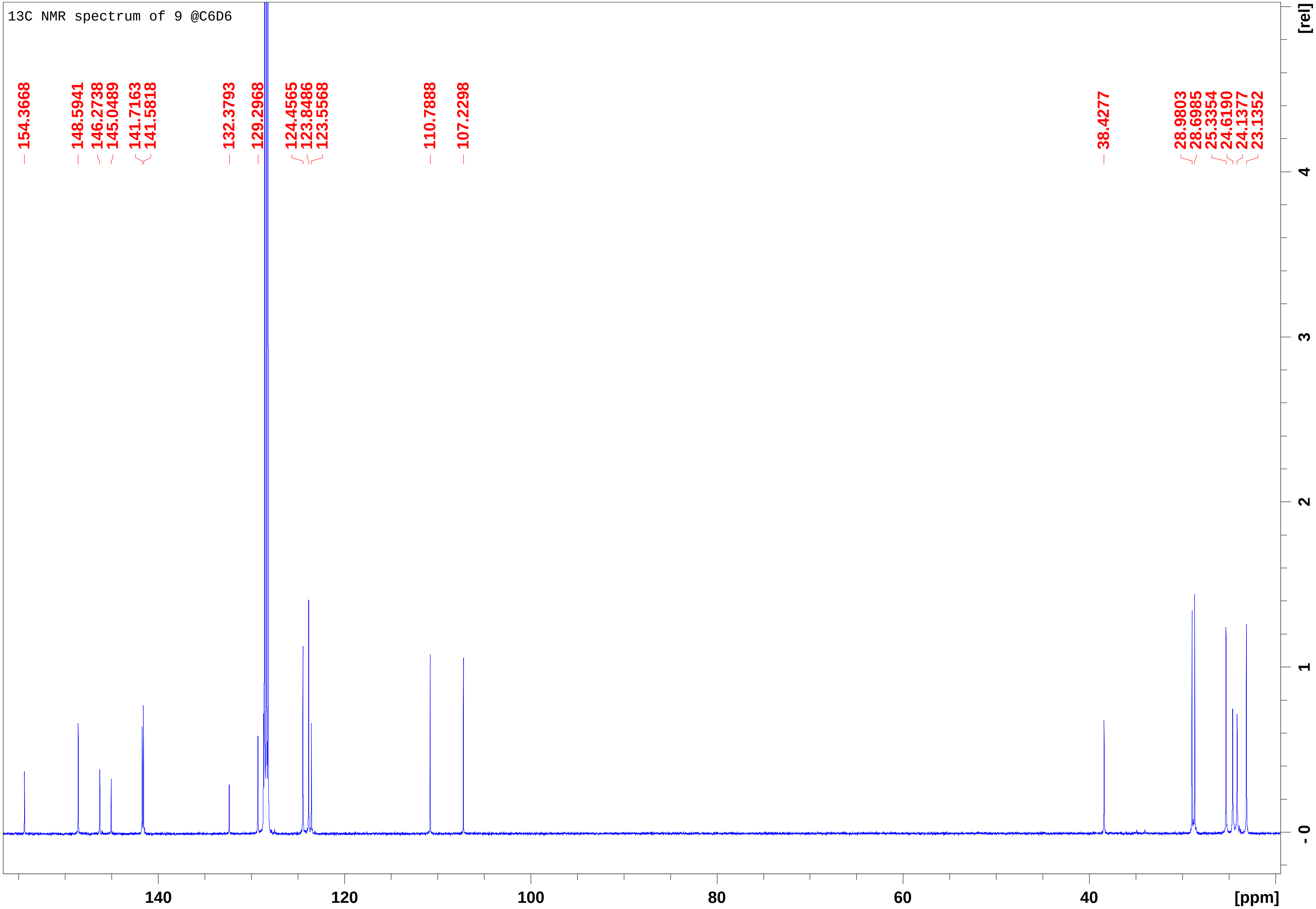

Figure S121. 13C NMR spectrum of 9 in C6D6

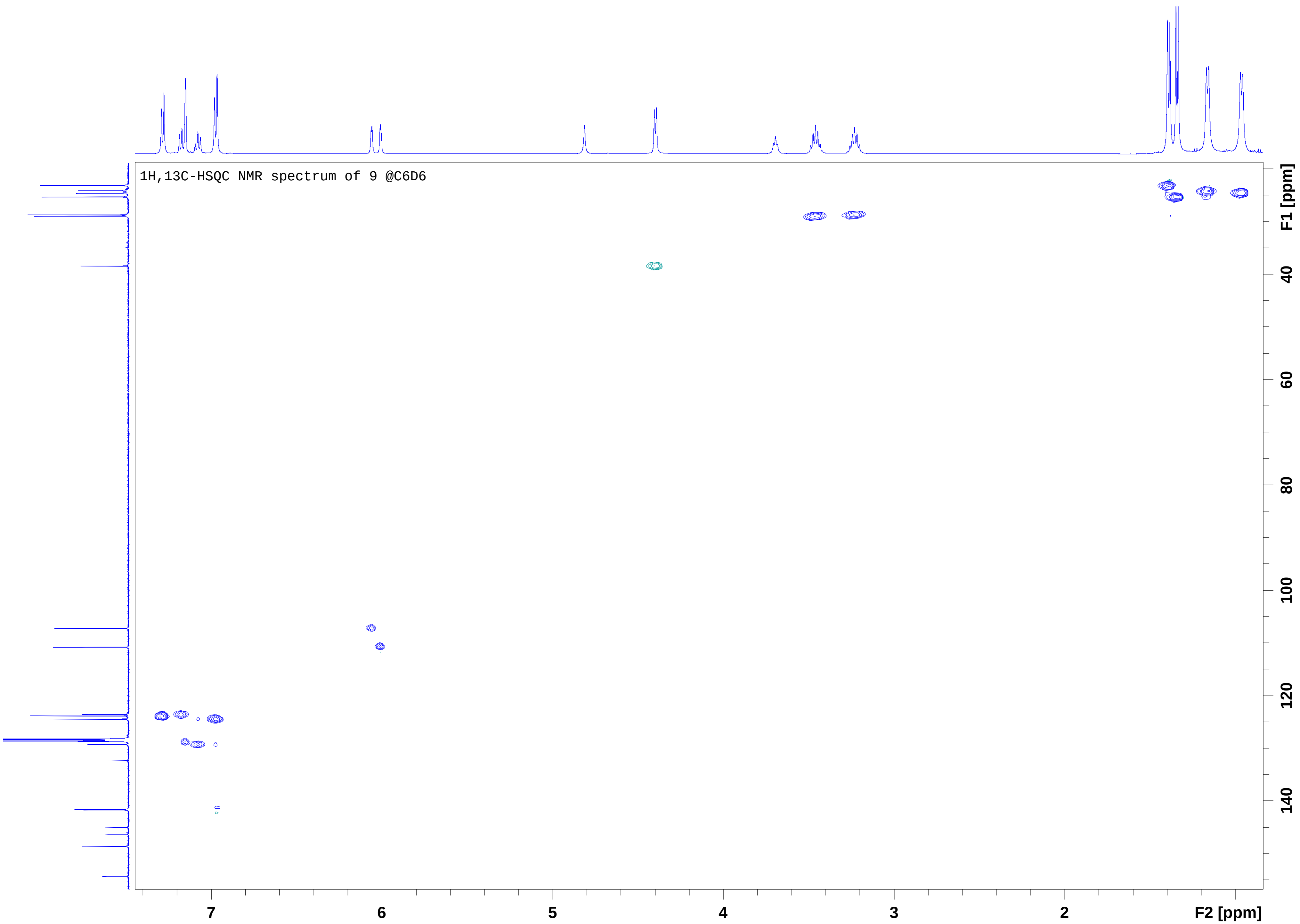

Figure S122. 1H,13C-HSQC NMR spectrum of 9 in C6D6

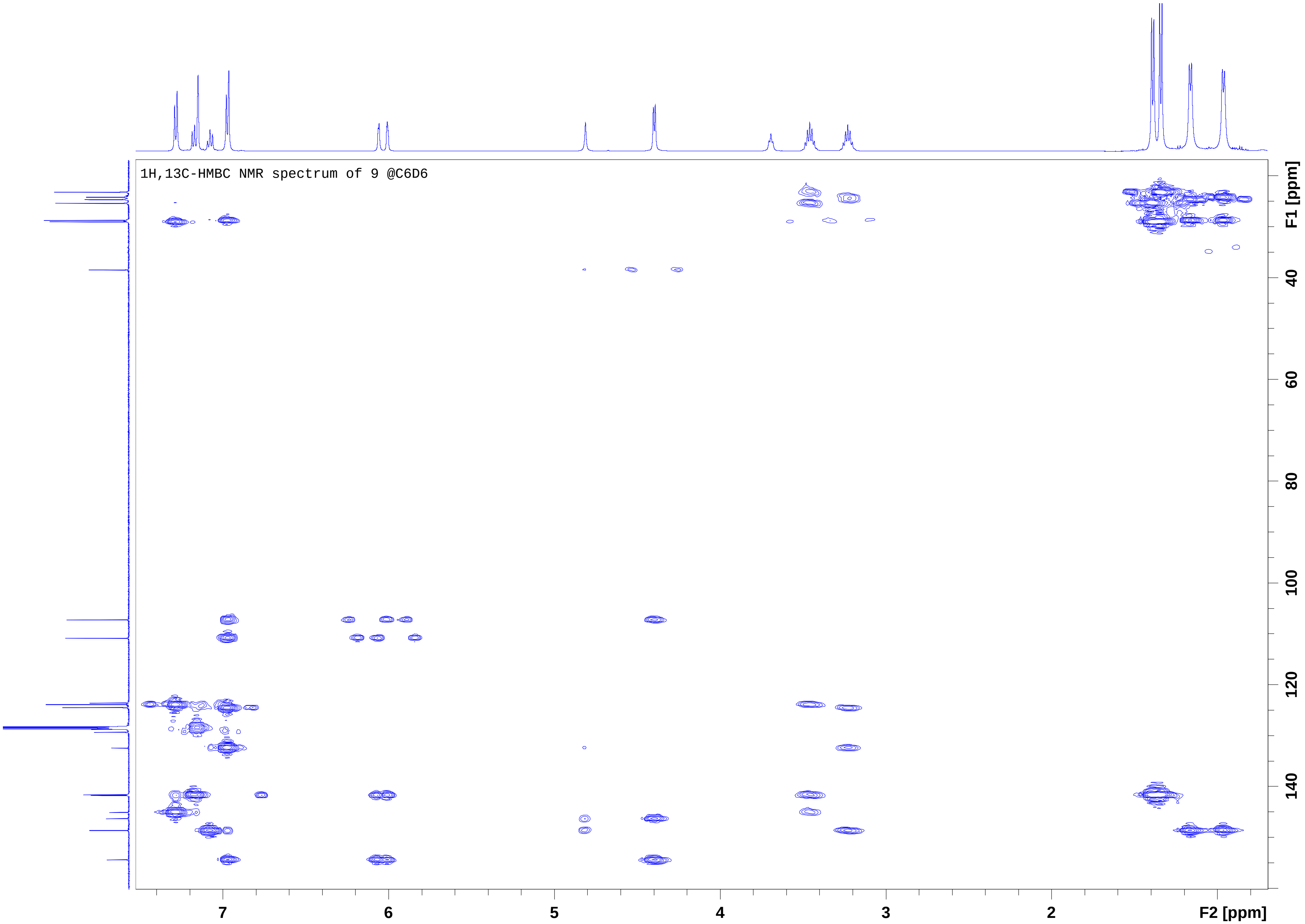

Figure S123. 1H,13C-HMBC NMR spectrum of 9 in C6D6

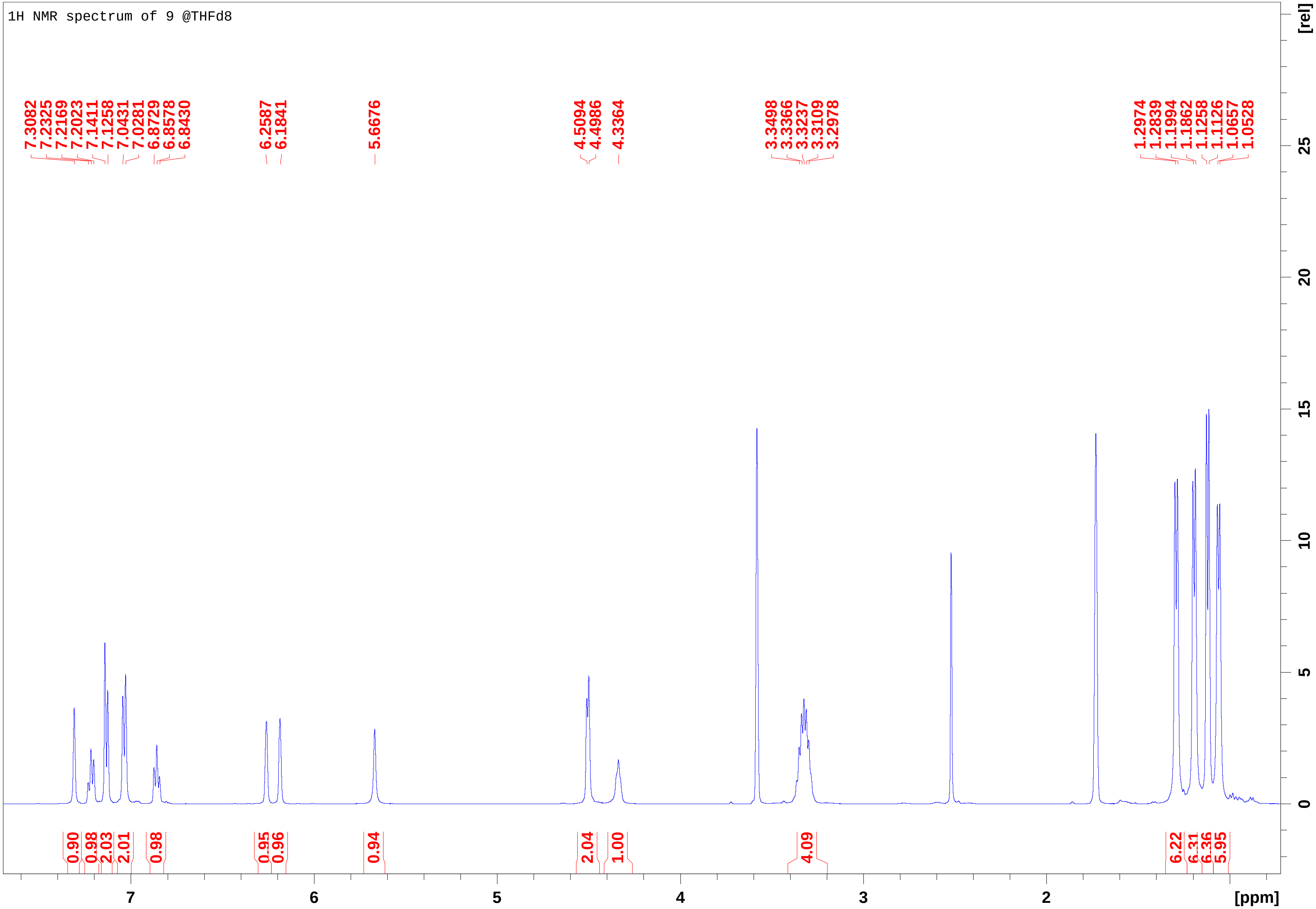

Figure S124. 1H NMR spectrum of 9 in THF-d8

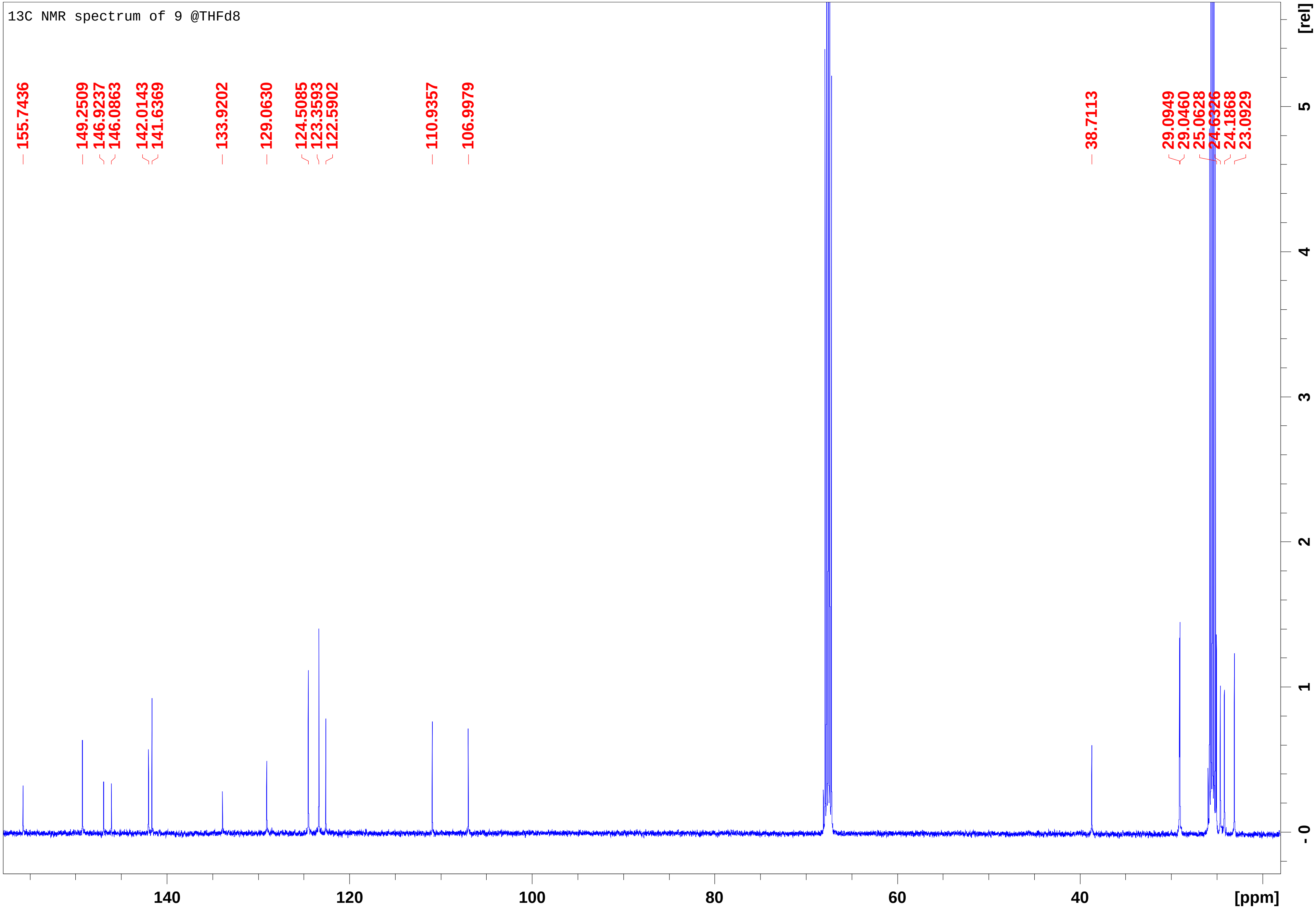

Figure S125. 13C NMR spectrum of 9 in THF-d8

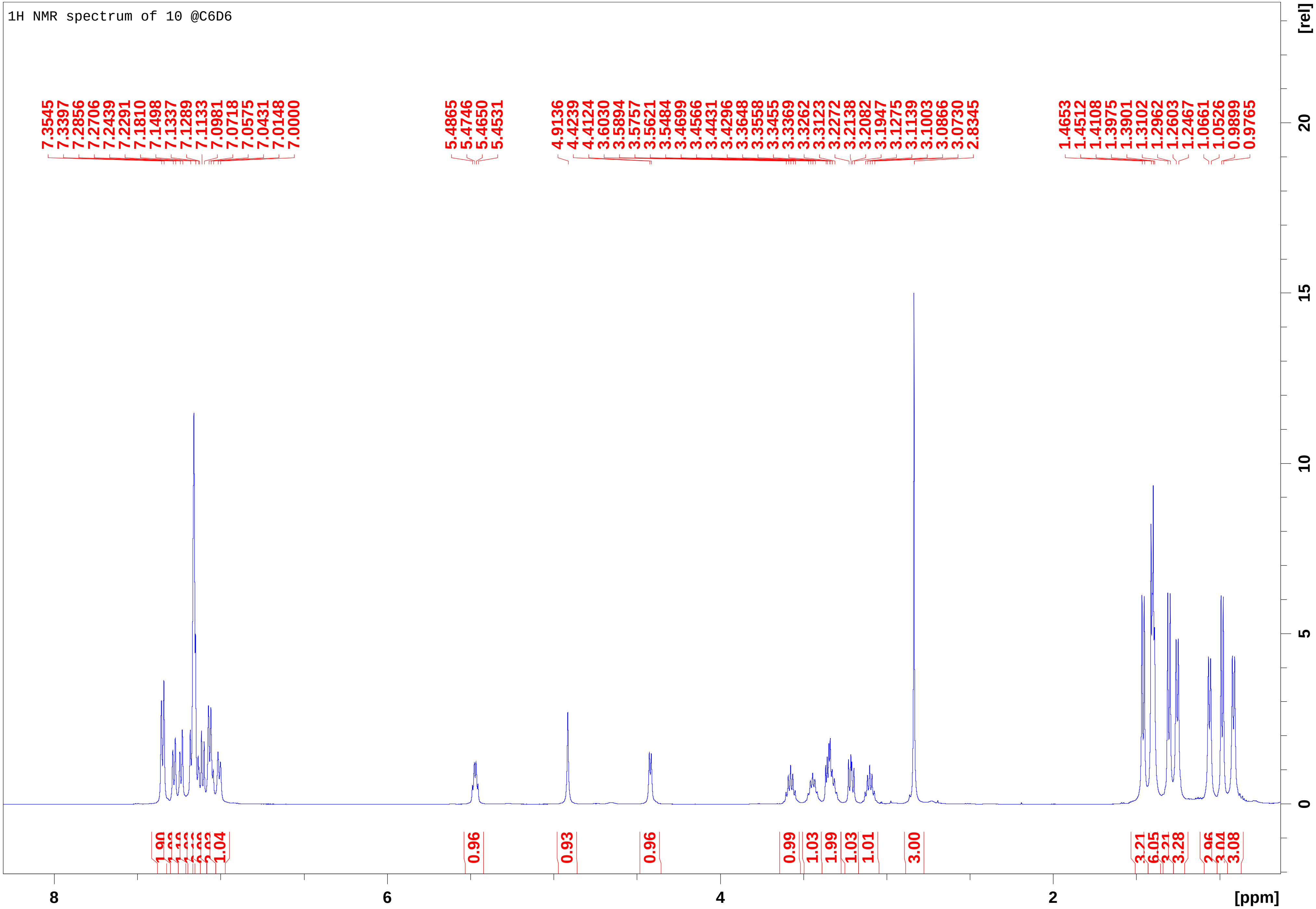

Figure S126. 1H NMR spectrum of 10 in C6D6

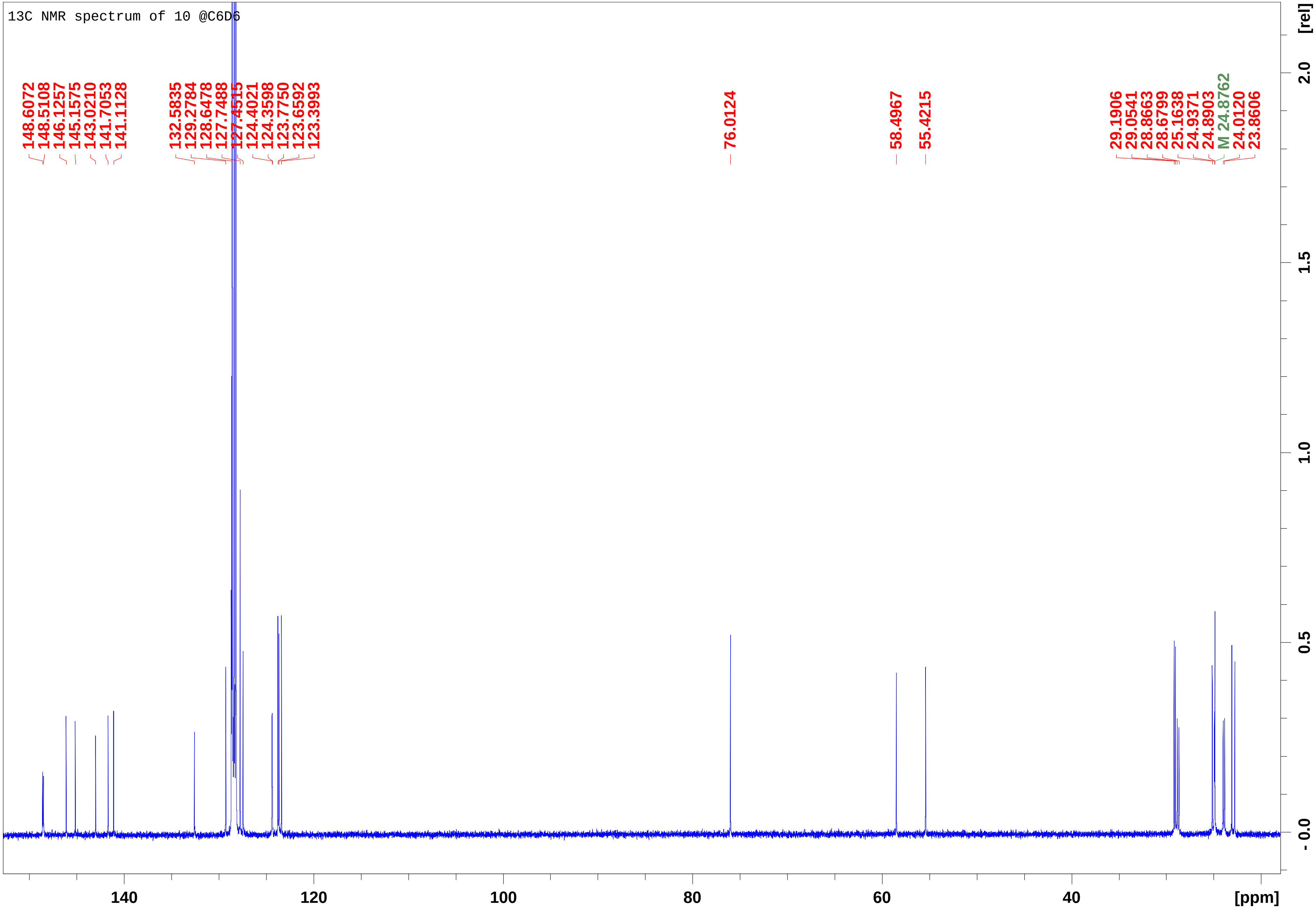

Figure S127. 13C NMR spectrum of 10 in C6D6

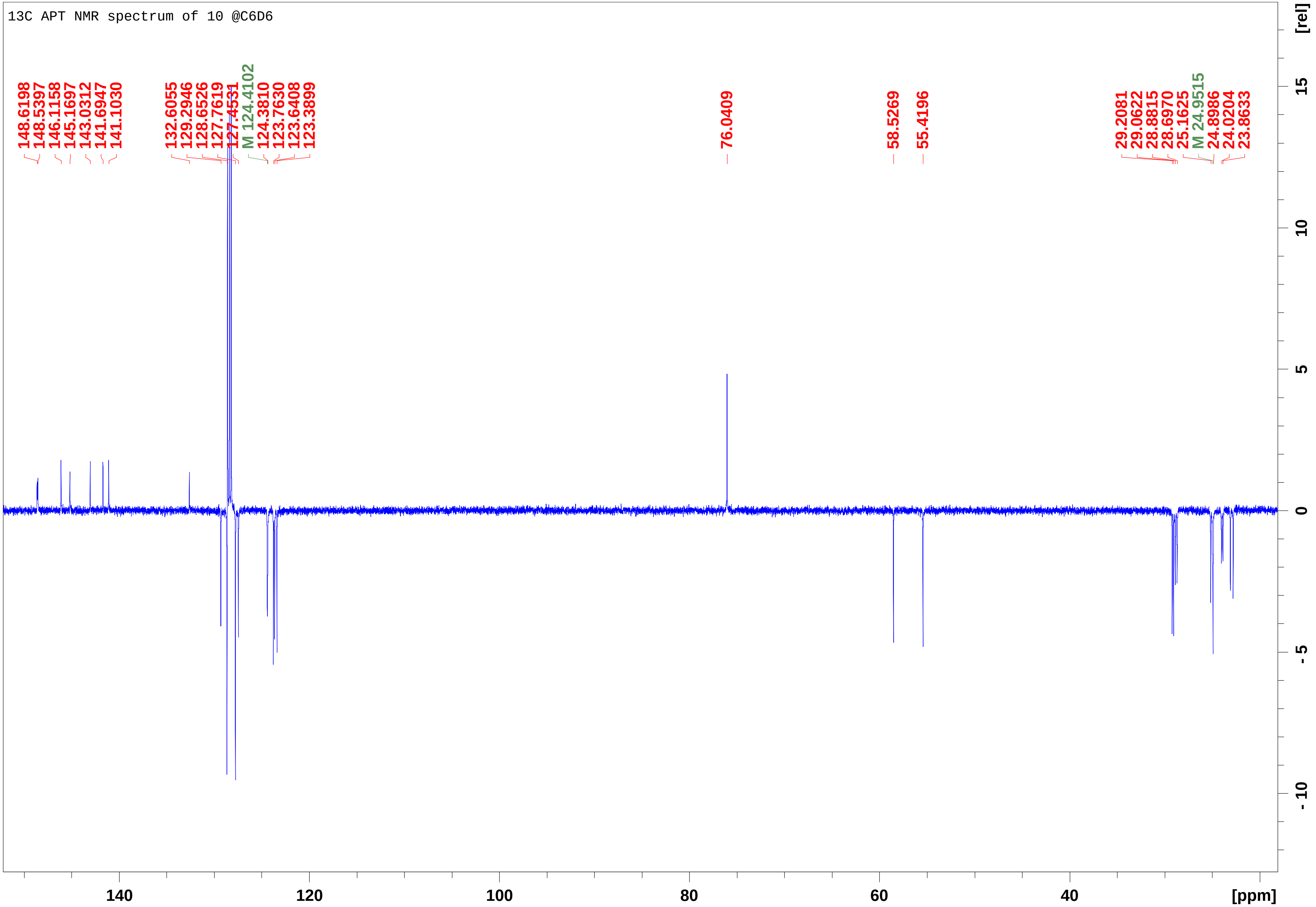

Figure S128. 13C APT NMR spectrum of 10 in C6D6

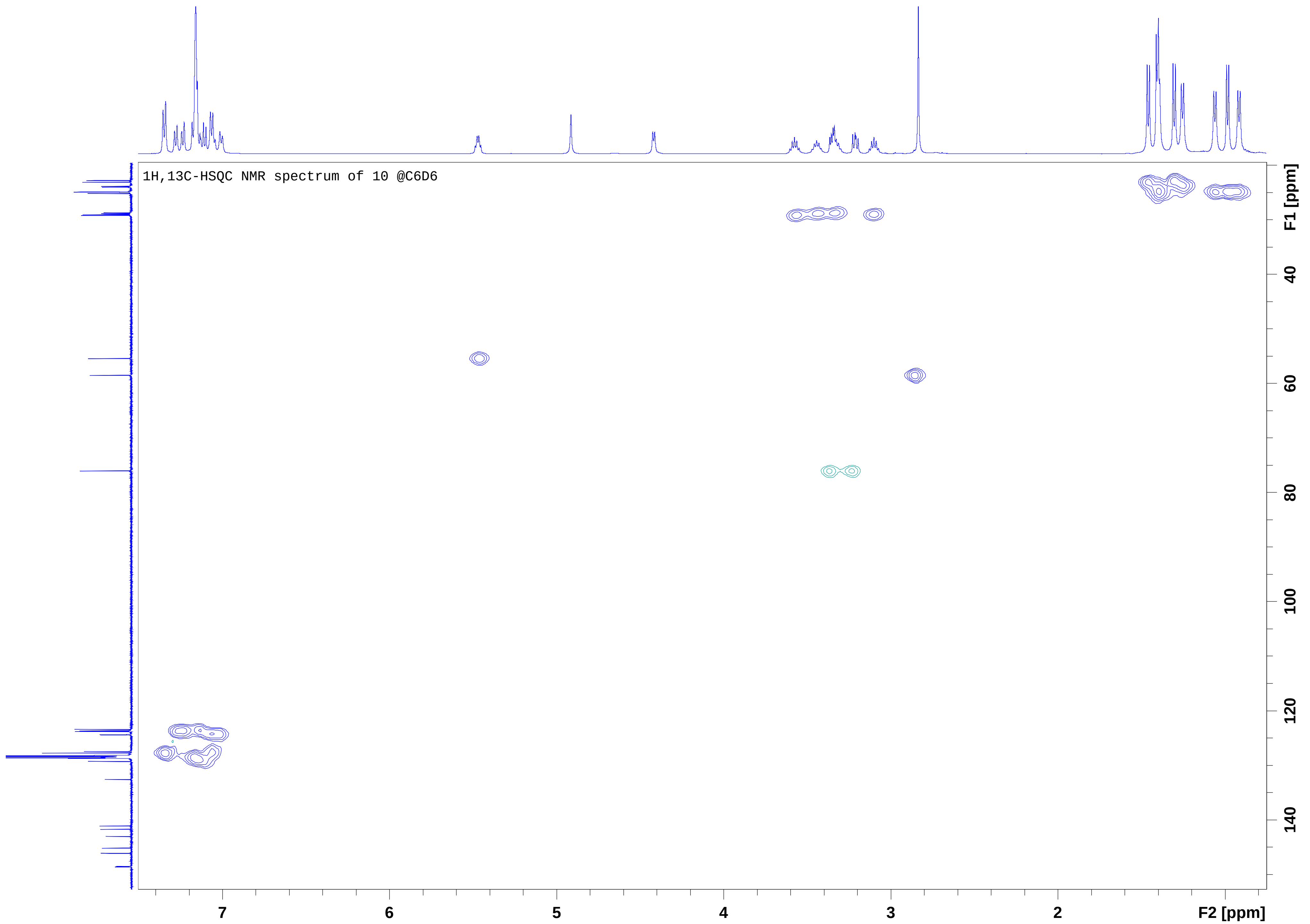

Figure S129. 1H,13C-HSQC NMR spectrum of 10 in C6D6

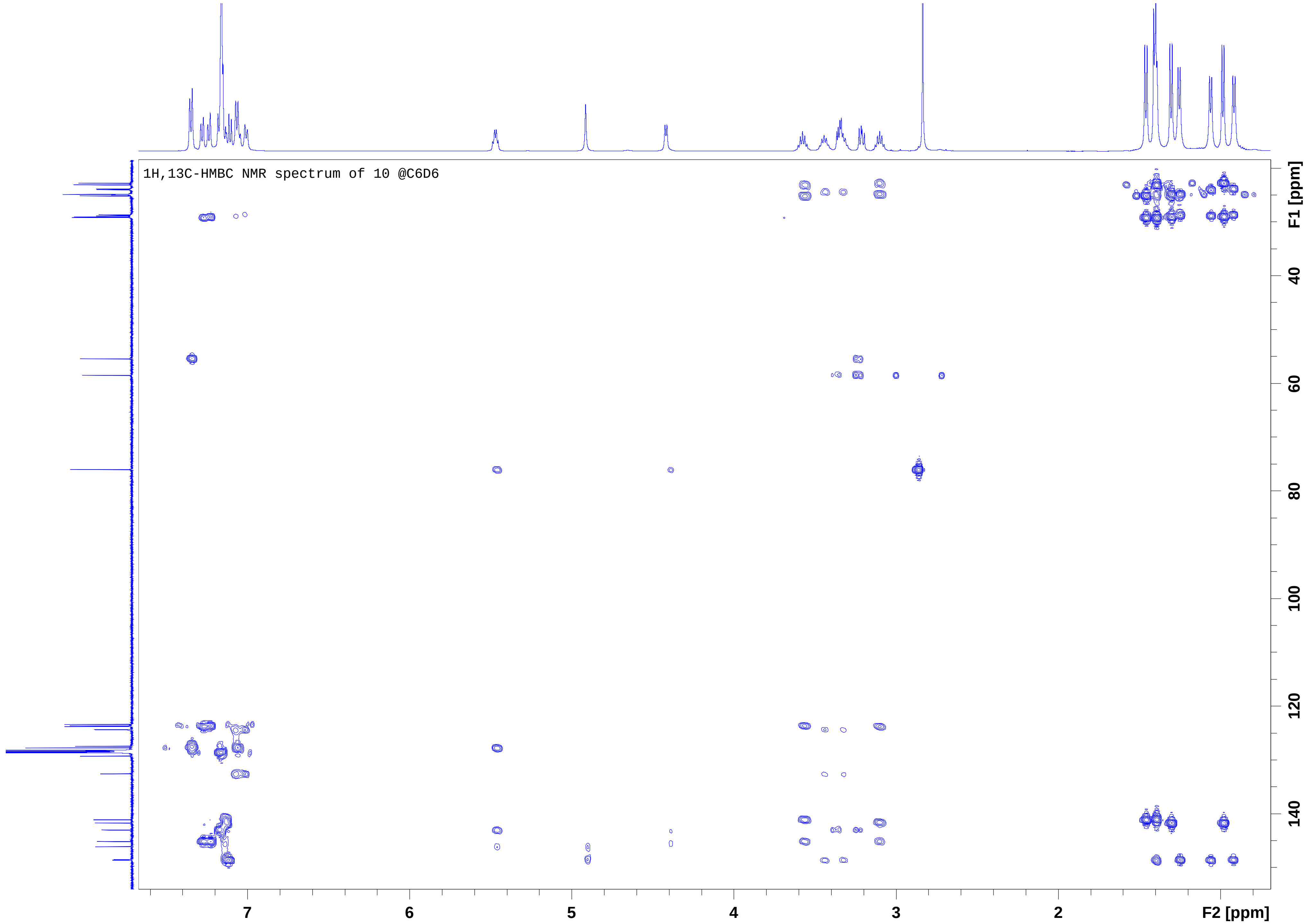

Figure S130. 1H,13C-HMBC NMR spectrum of 10 in C6D6

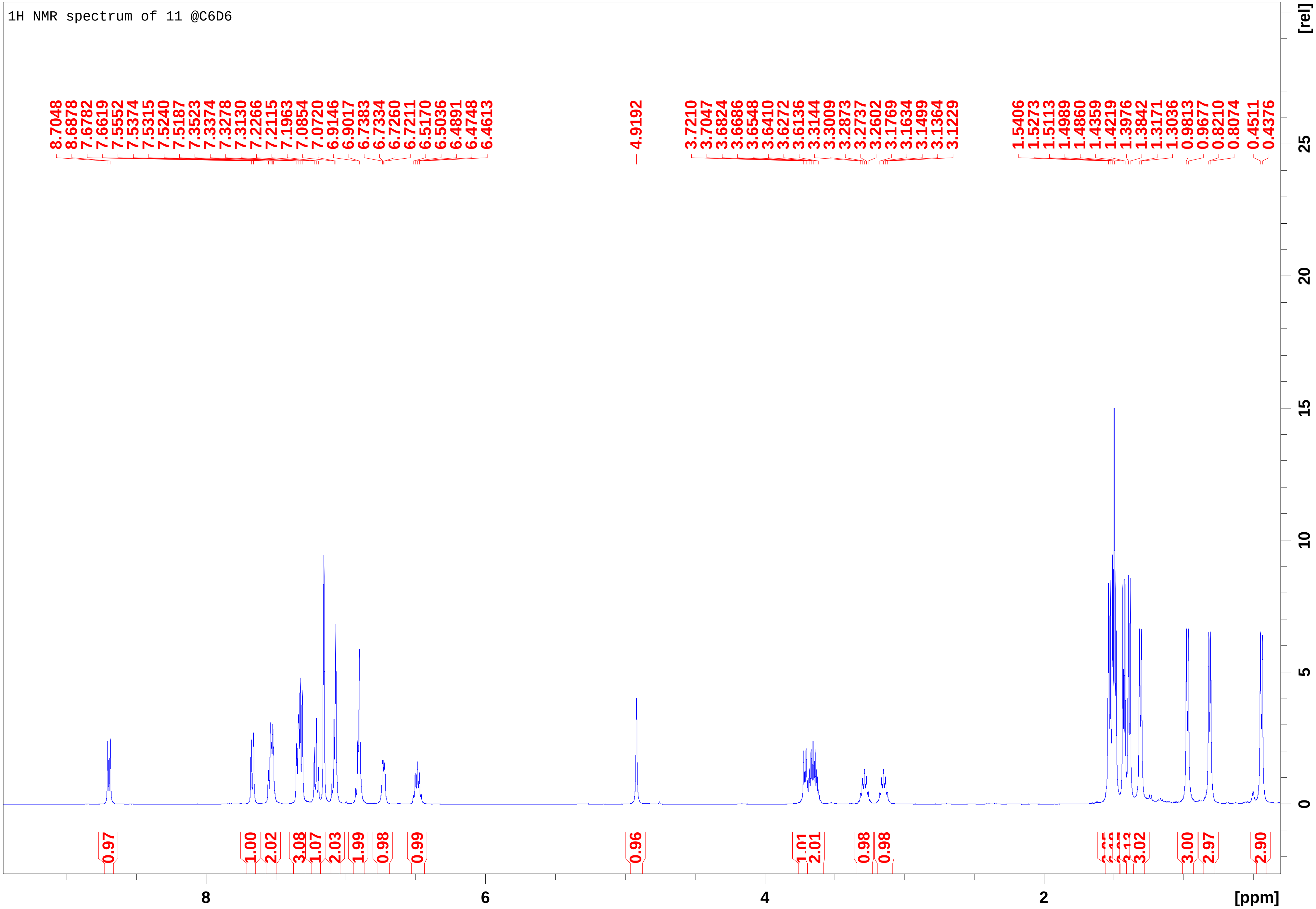

Figure S131. 1H NMR spectrum of 11 in C6D6

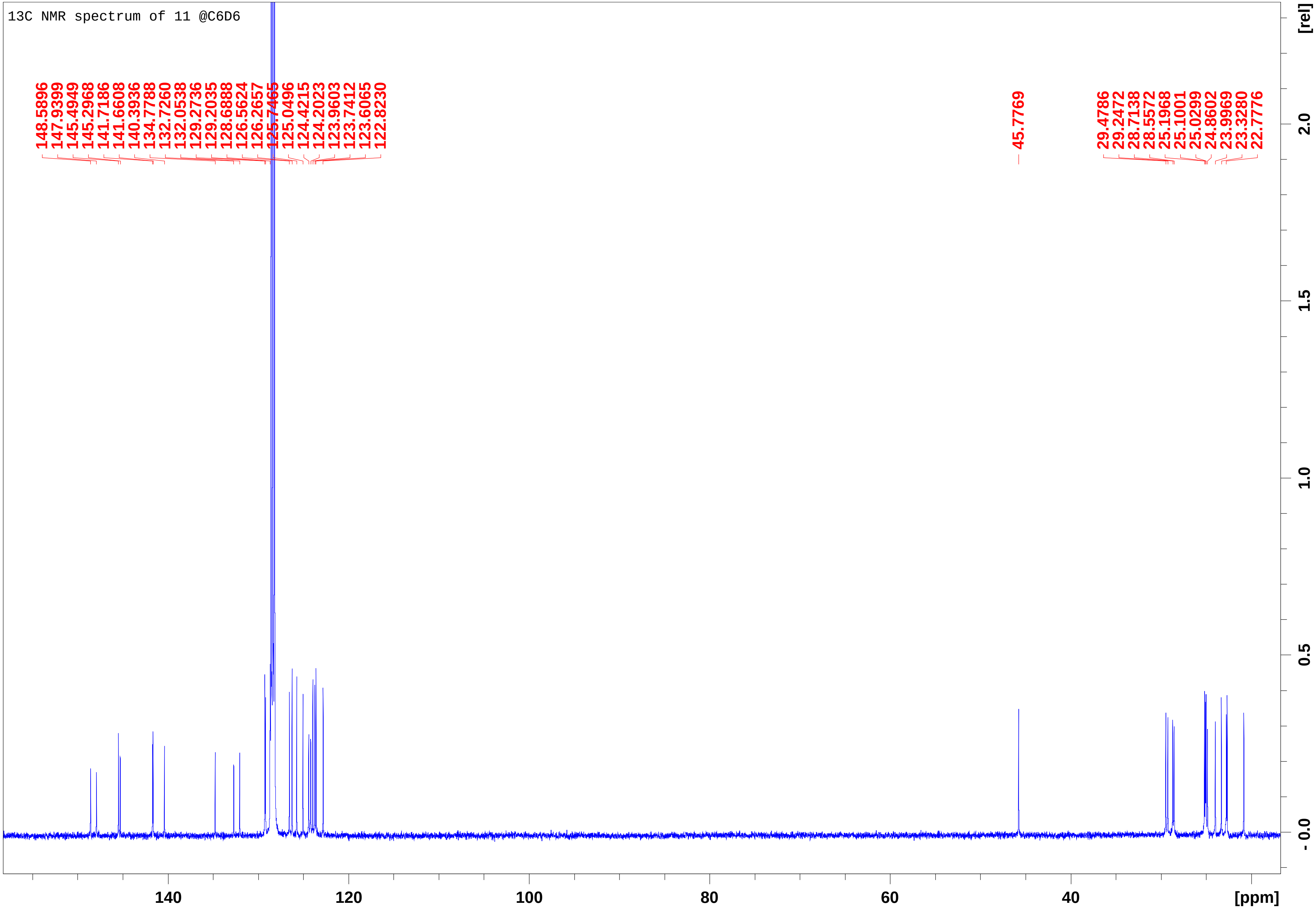

Figure S132. 13C NMR spectrum of 11 in C6D6

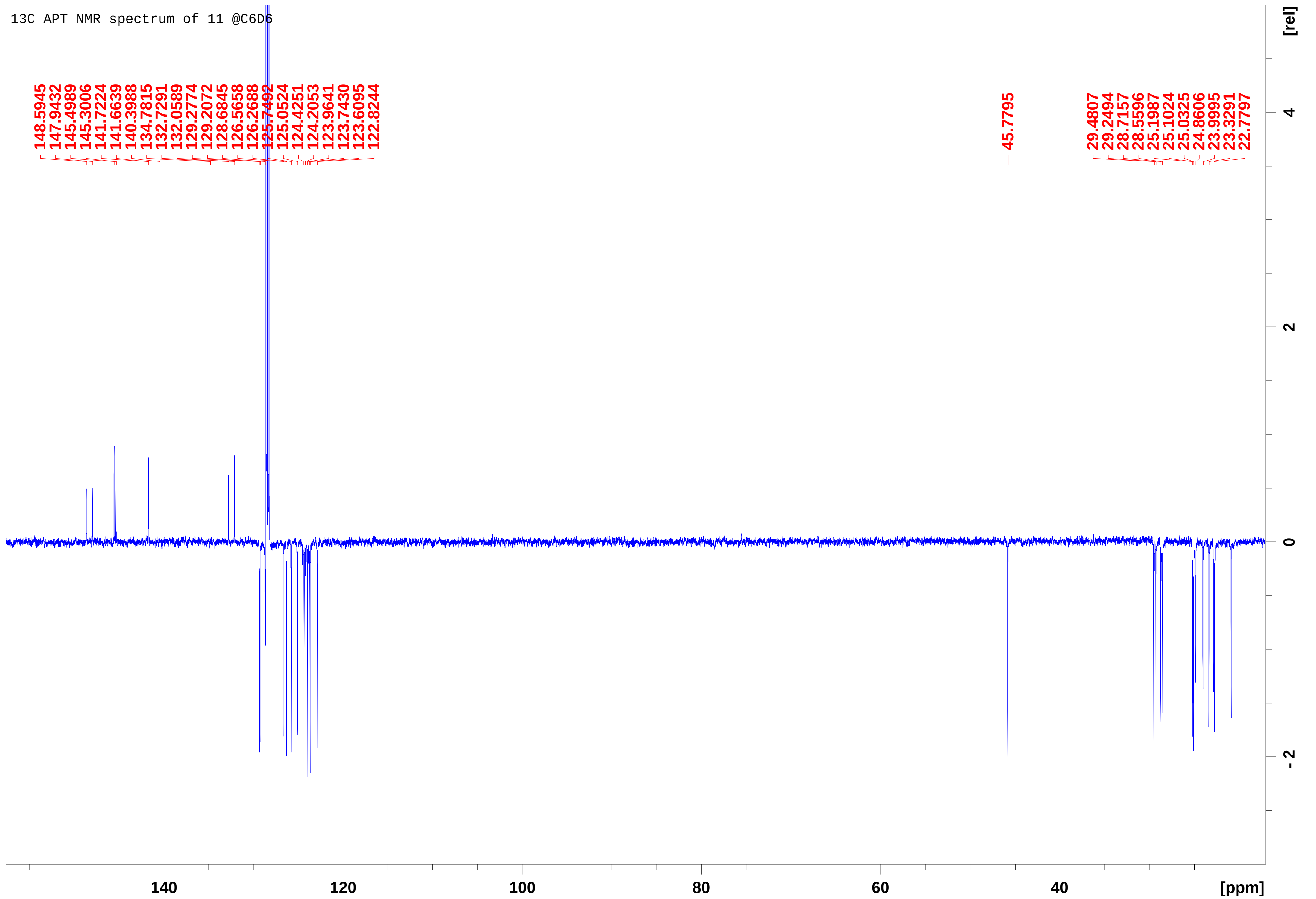

Figure S133. 13C APT NMR spectrum of 11 in C6D6

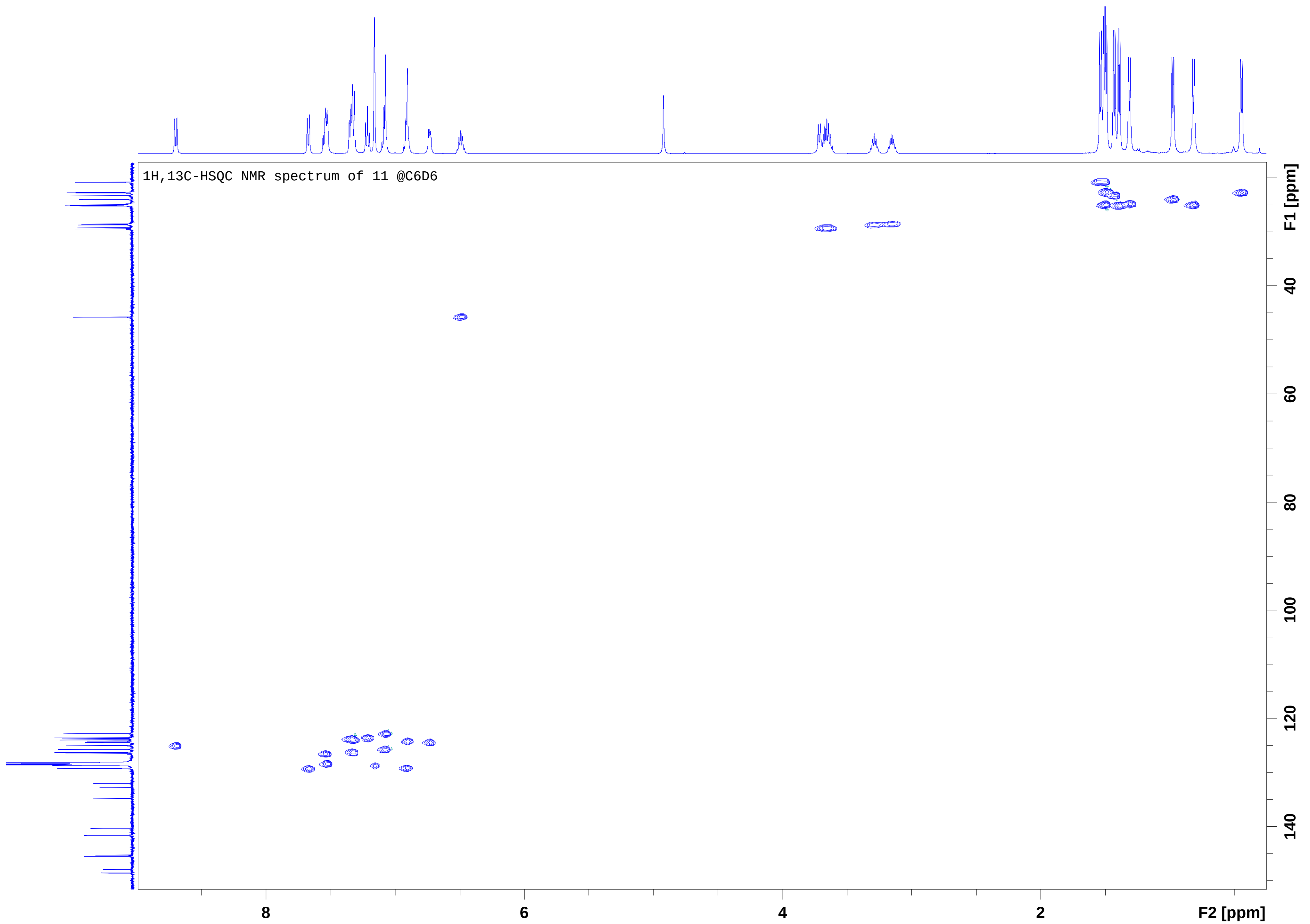

Figure S134. 1H,13C-HSQC NMR spectrum of 11 in C6D6

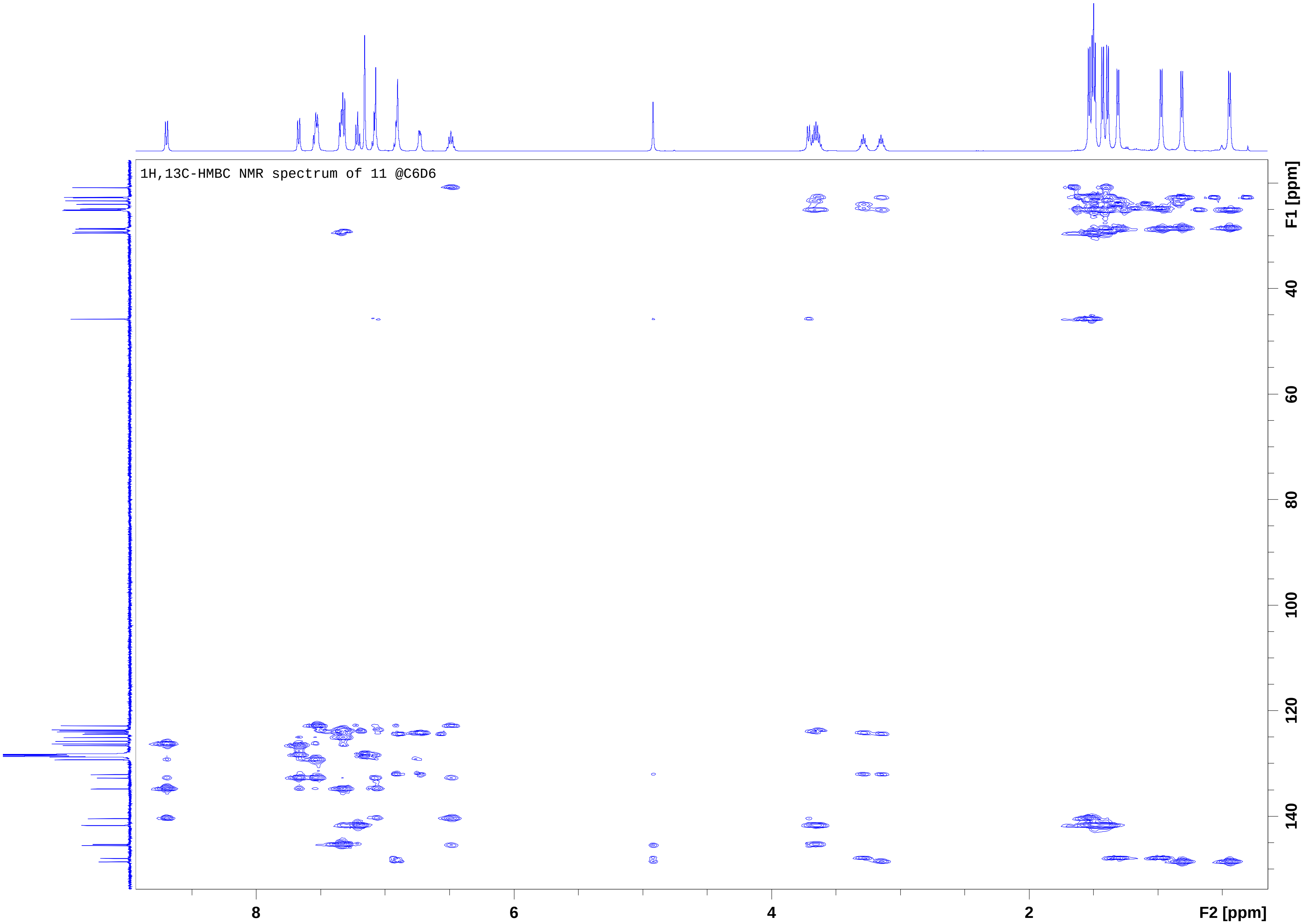

Figure S135. 1H,13C-HMBC NMR spectrum of 11 in C6D6

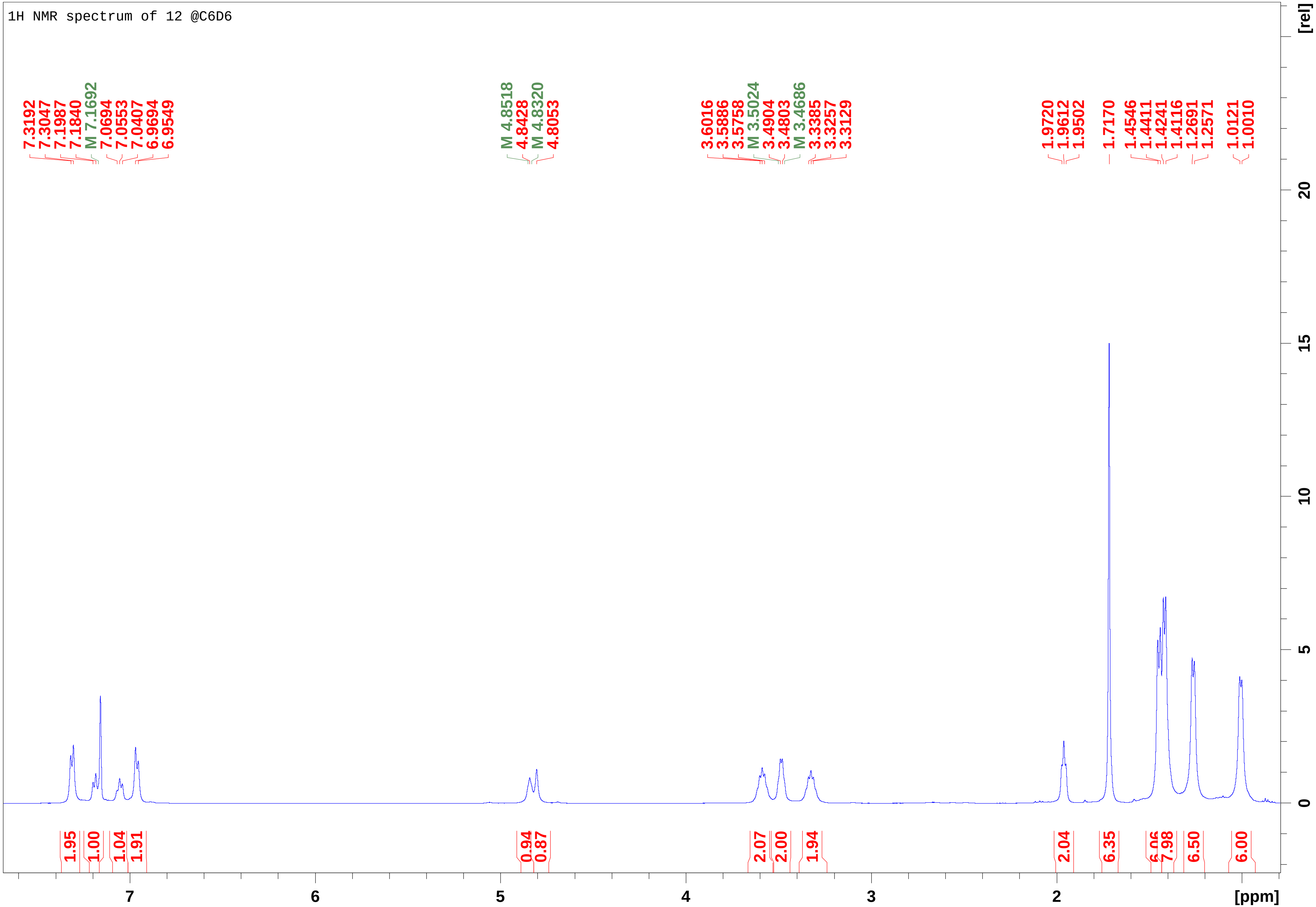

Figure S136. 1H NMR spectrum of 12 in C6D6

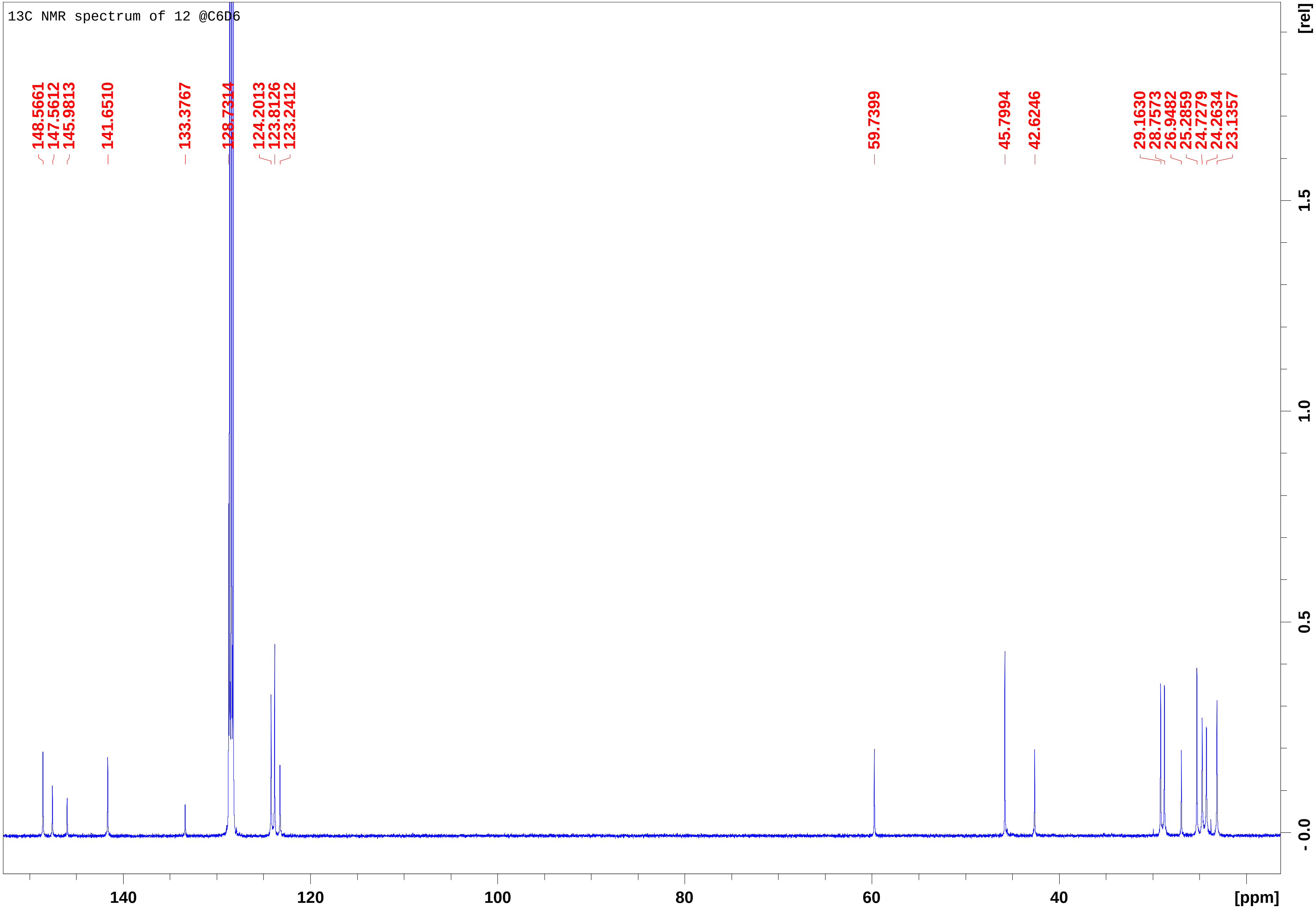

Figure S137. 13C NMR spectrum of 12 in C6D6

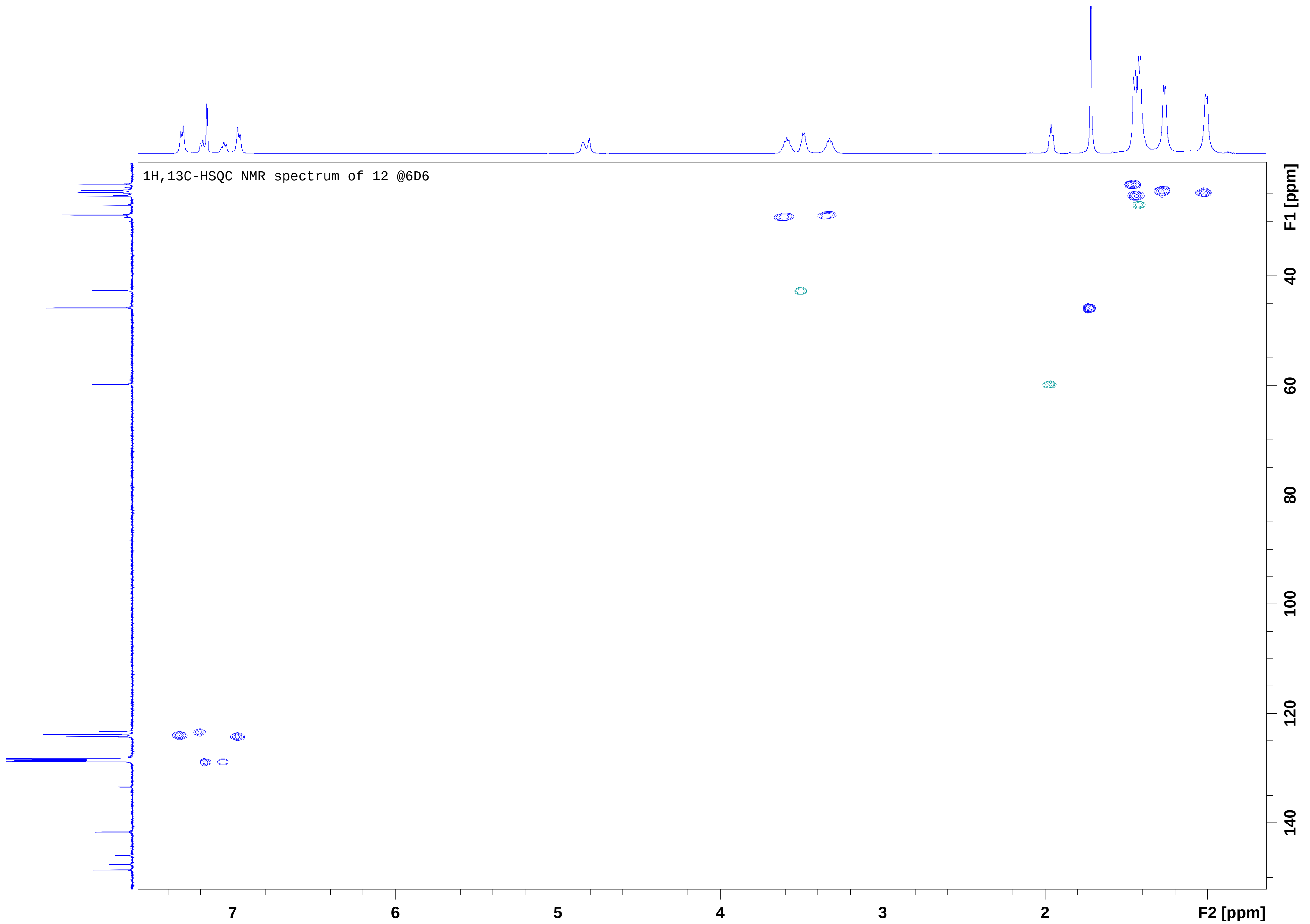

Figure S138. 1H,13C-HSQC NMR spectrum of 12 in C6D6

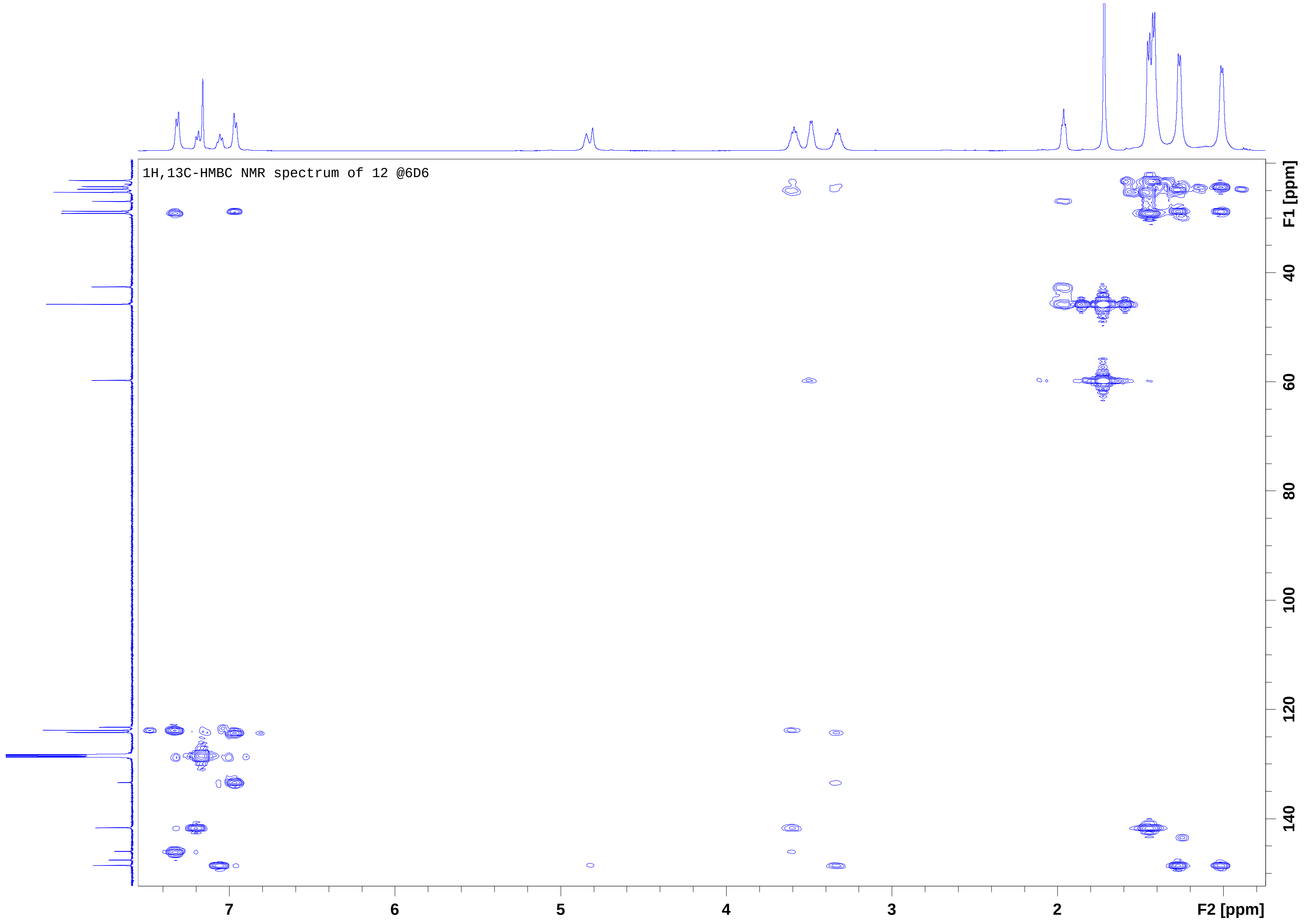

Figure S139. 1H,13C-HMBC NMR spectrum of 12 in C6D6

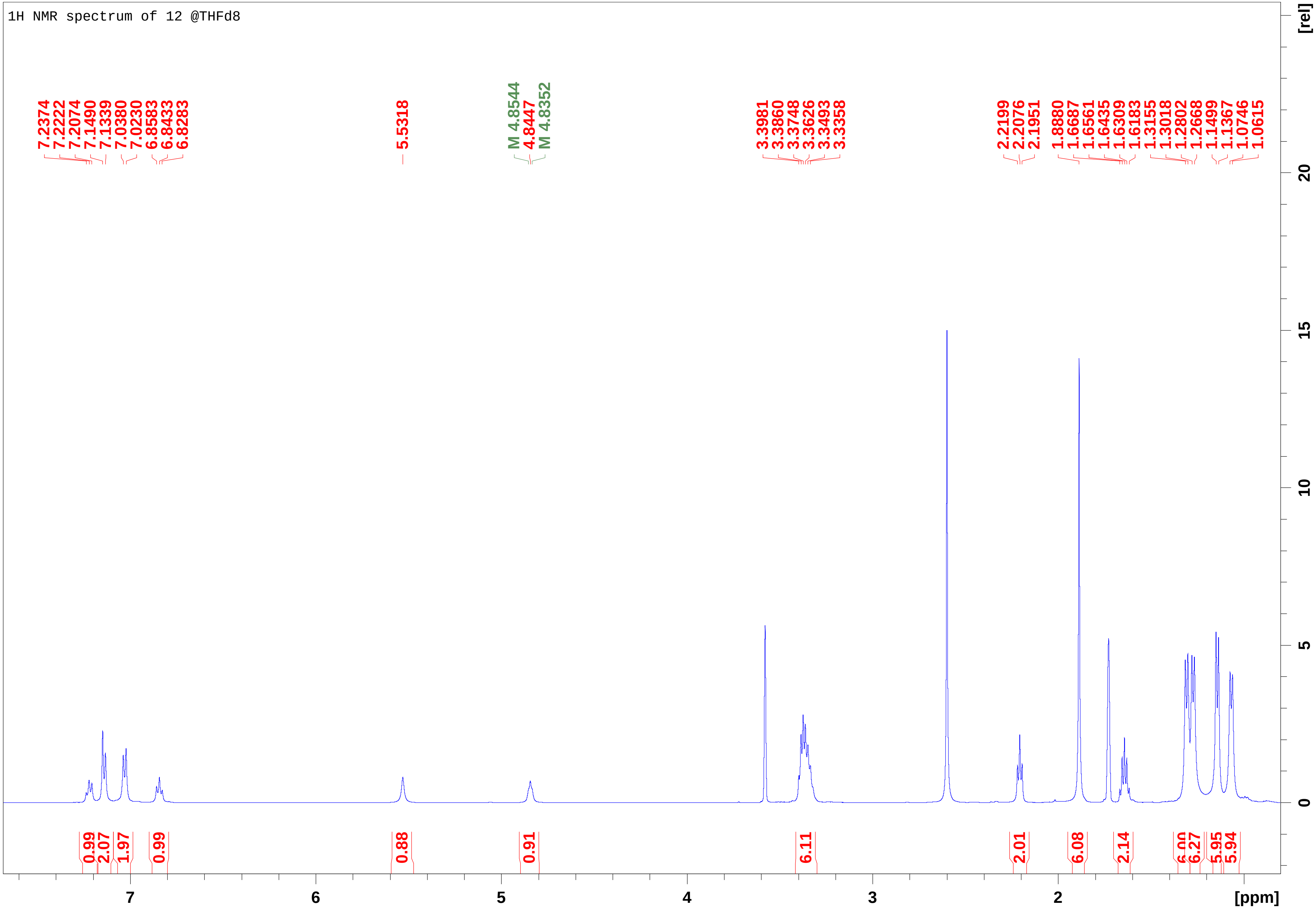

Figure S140. 1H NMR spectrum of 12 in THF-d8

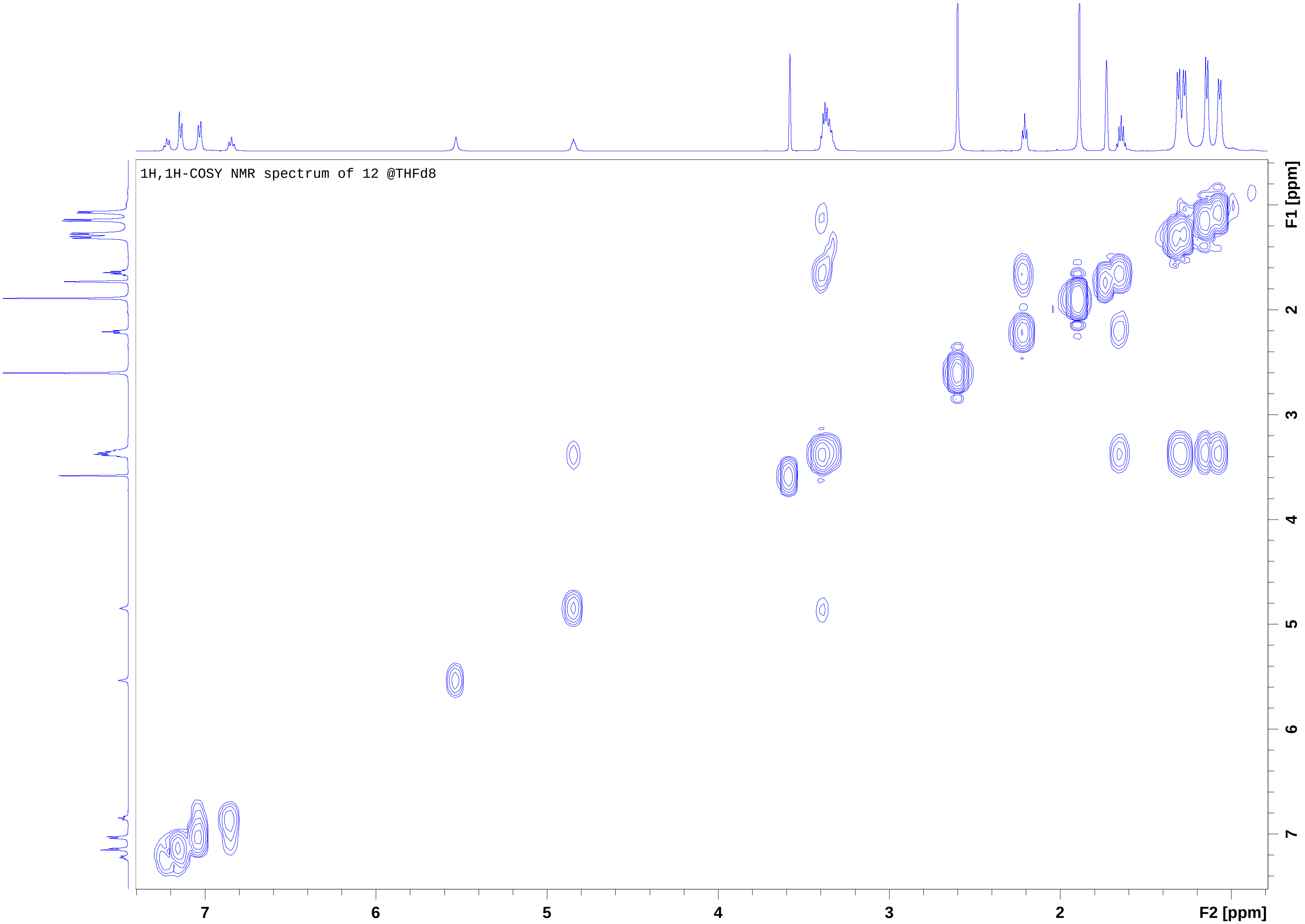

Figure S141. 1H,1H-COSY NMR spectrum of 12 in THF-d8

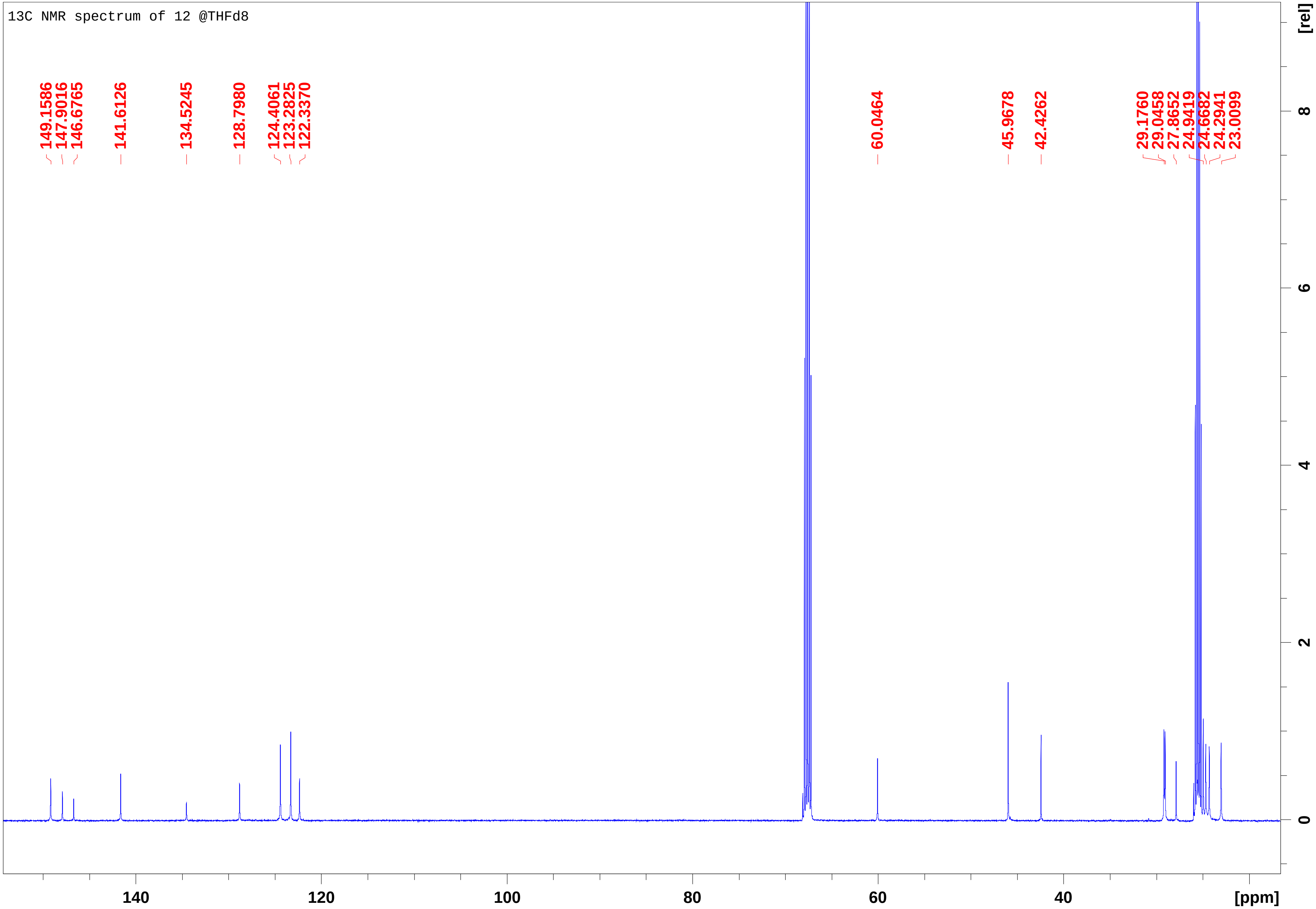

Figure S142. 13C NMR spectrum of 12 in THF-d8

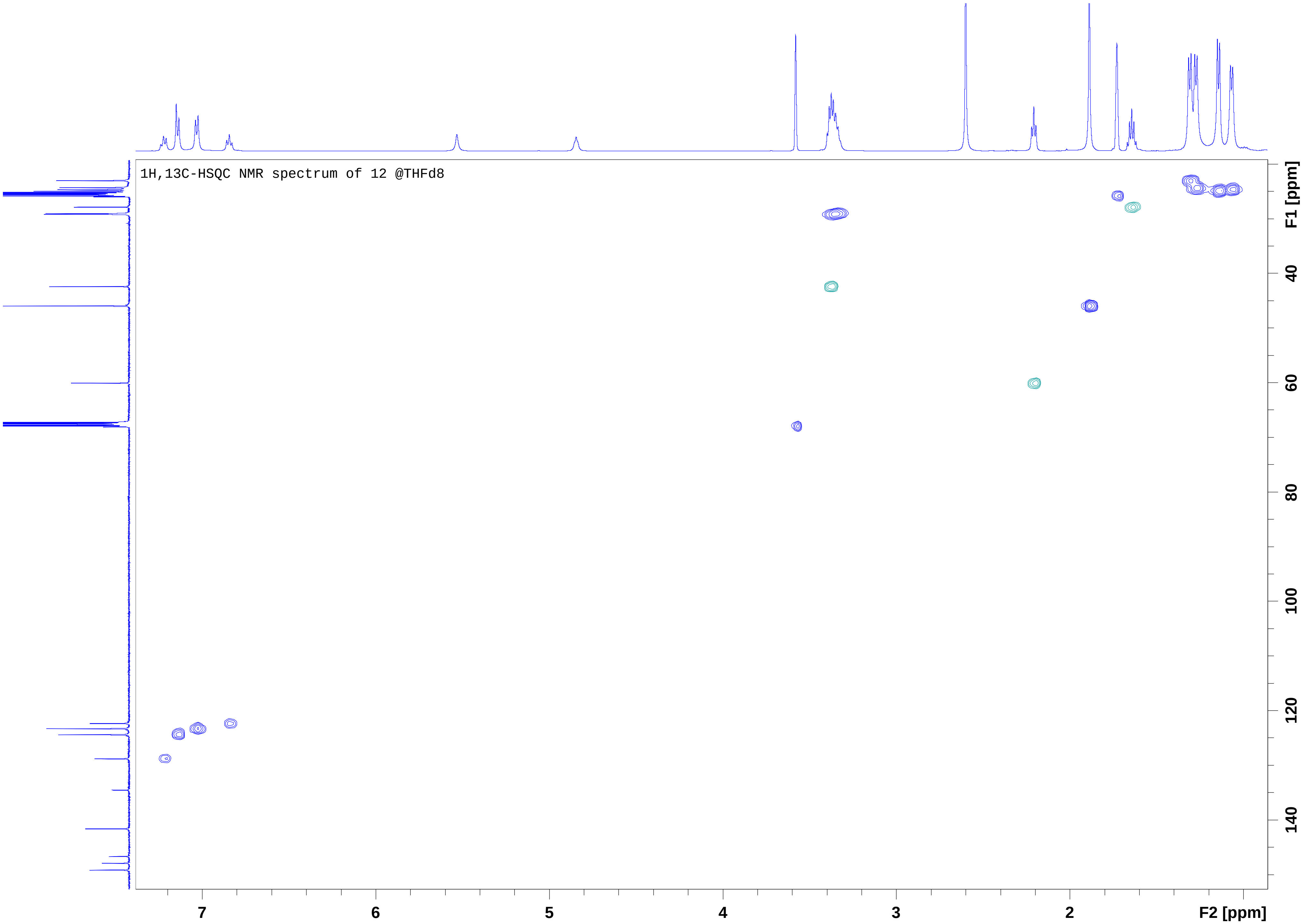

Figure S143. 1H,13C-HSQC NMR spectrum of 12 in THF-d8

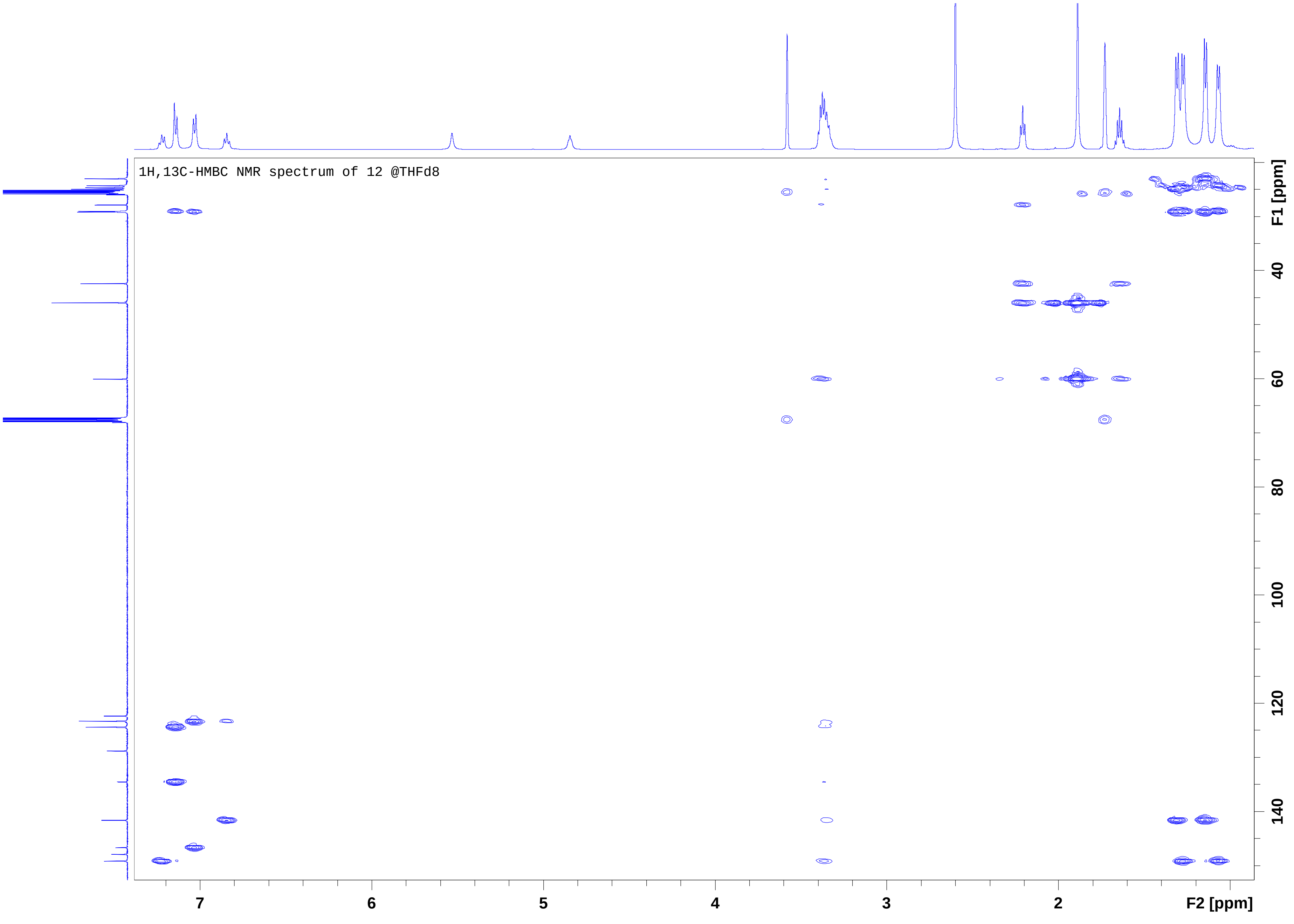

Figure S144. 1H,13C-HMBC NMR spectrum of 12 in THF-d8

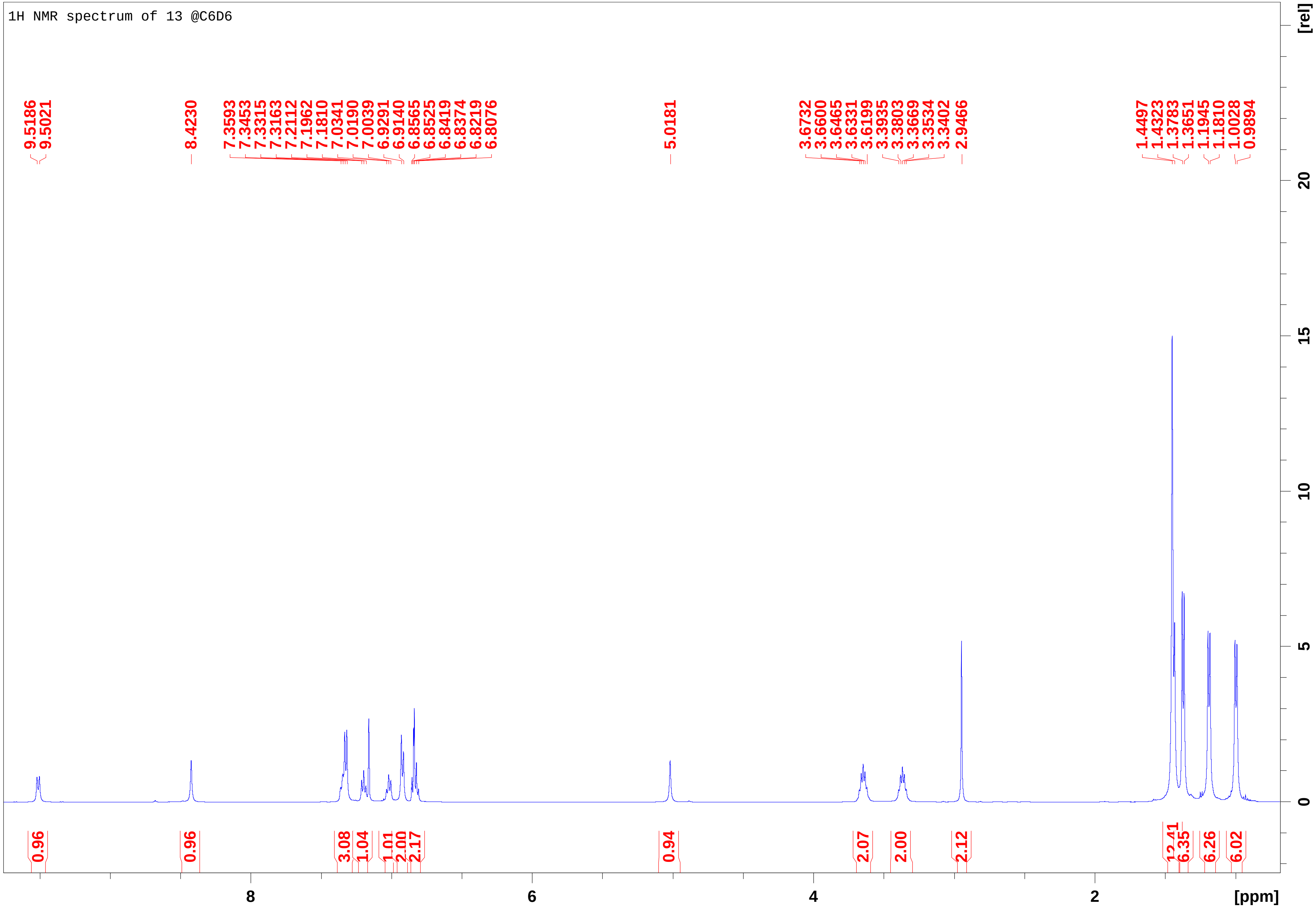

Figure S145. 1H NMR spectrum of 13 in C6D6

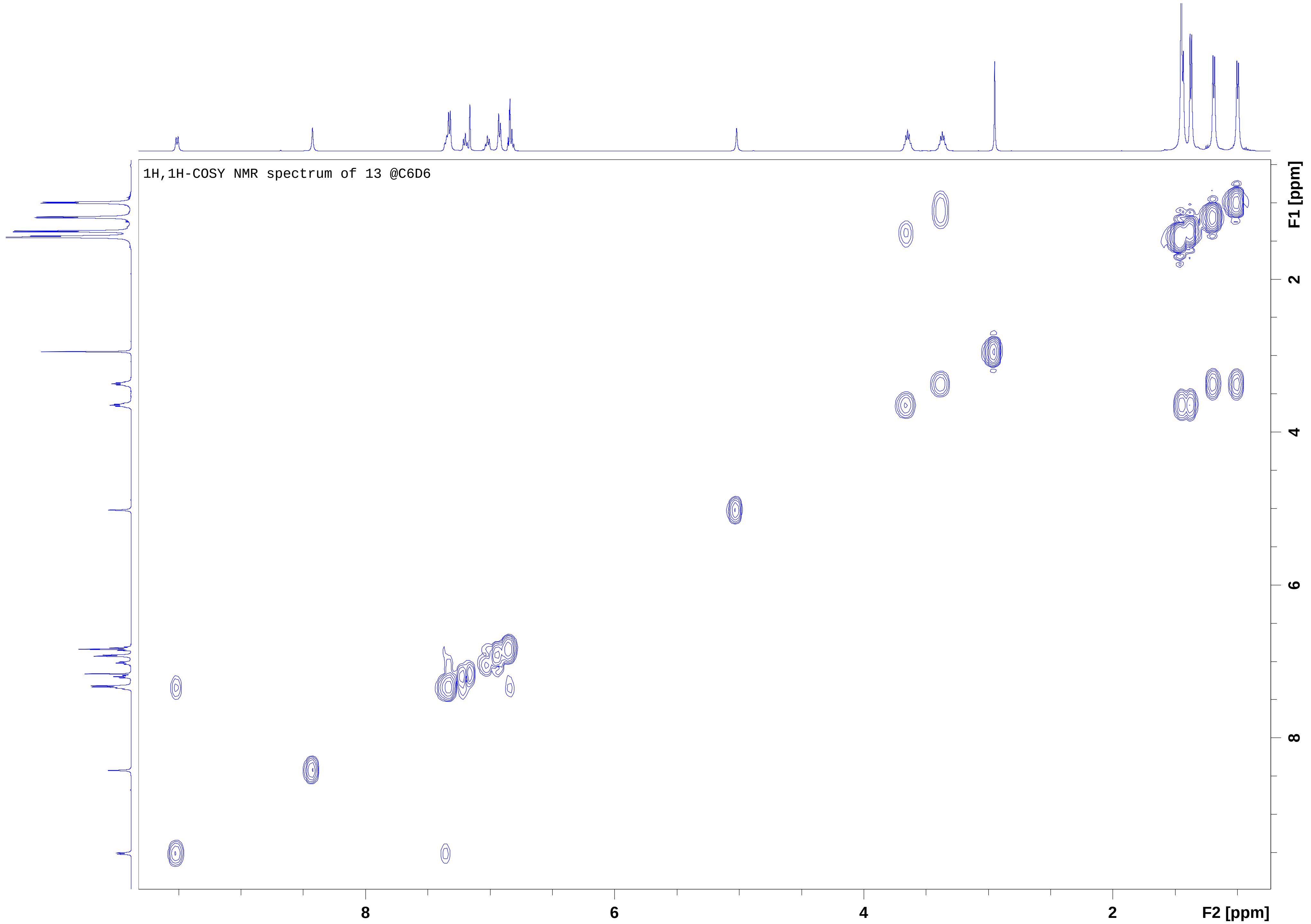

Figure S146. 1H,1H-COSY NMR spectrum of 13 in C6D6

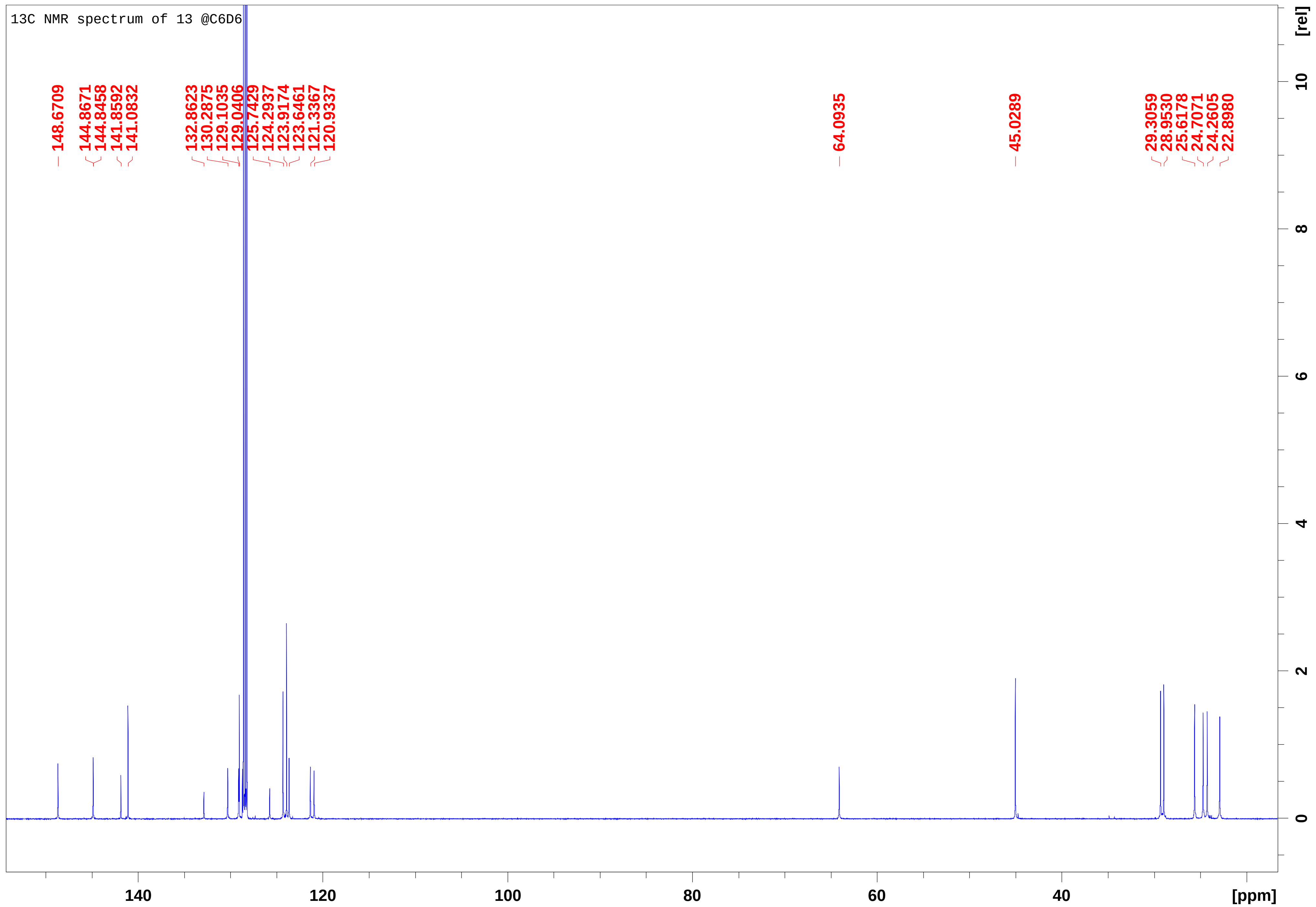

Figure S147. 13C NMR spectrum of 13 in C6D6

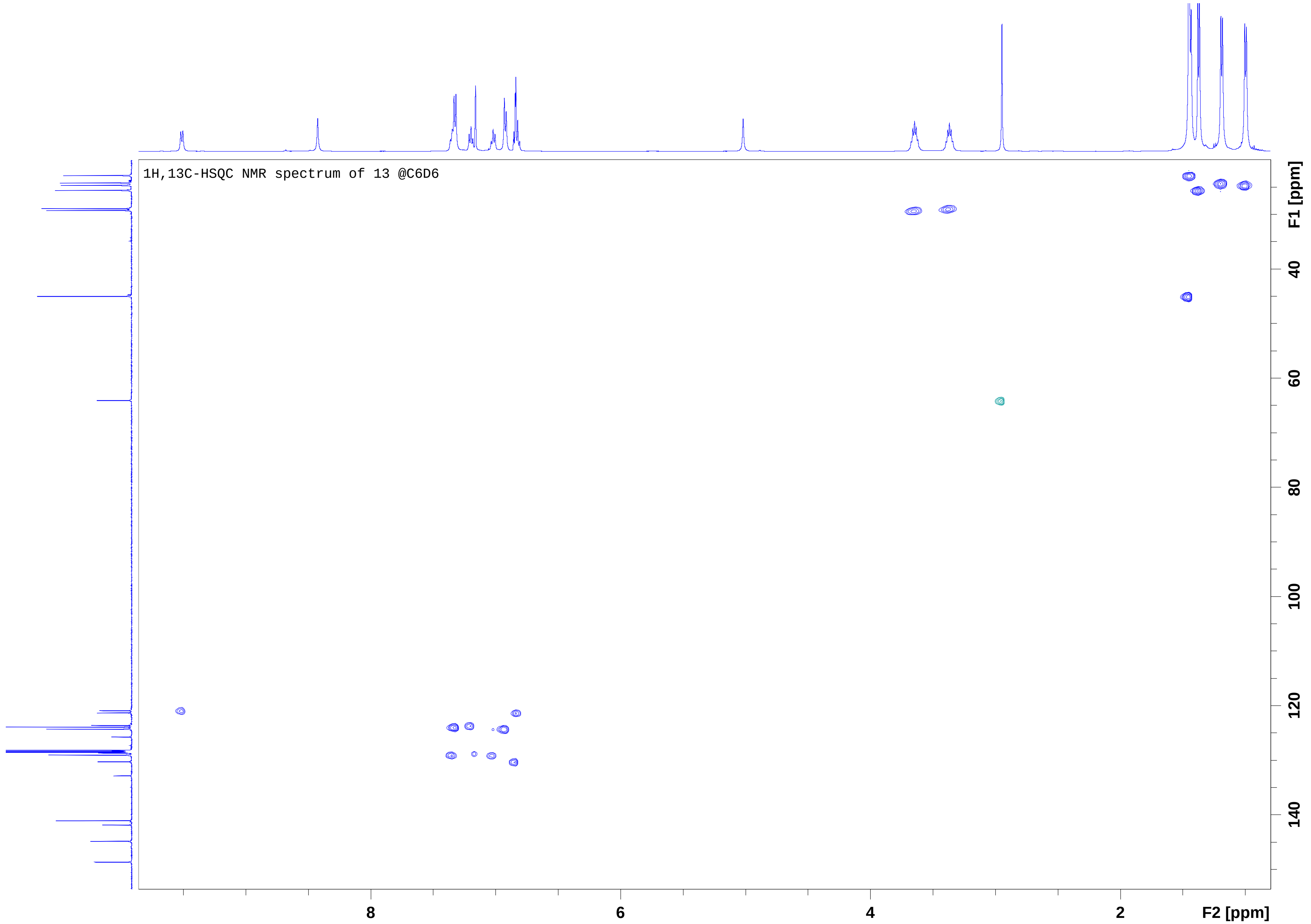

Figure S148. 1H,13C-HSQC NMR spectrum of 13 in C6D6

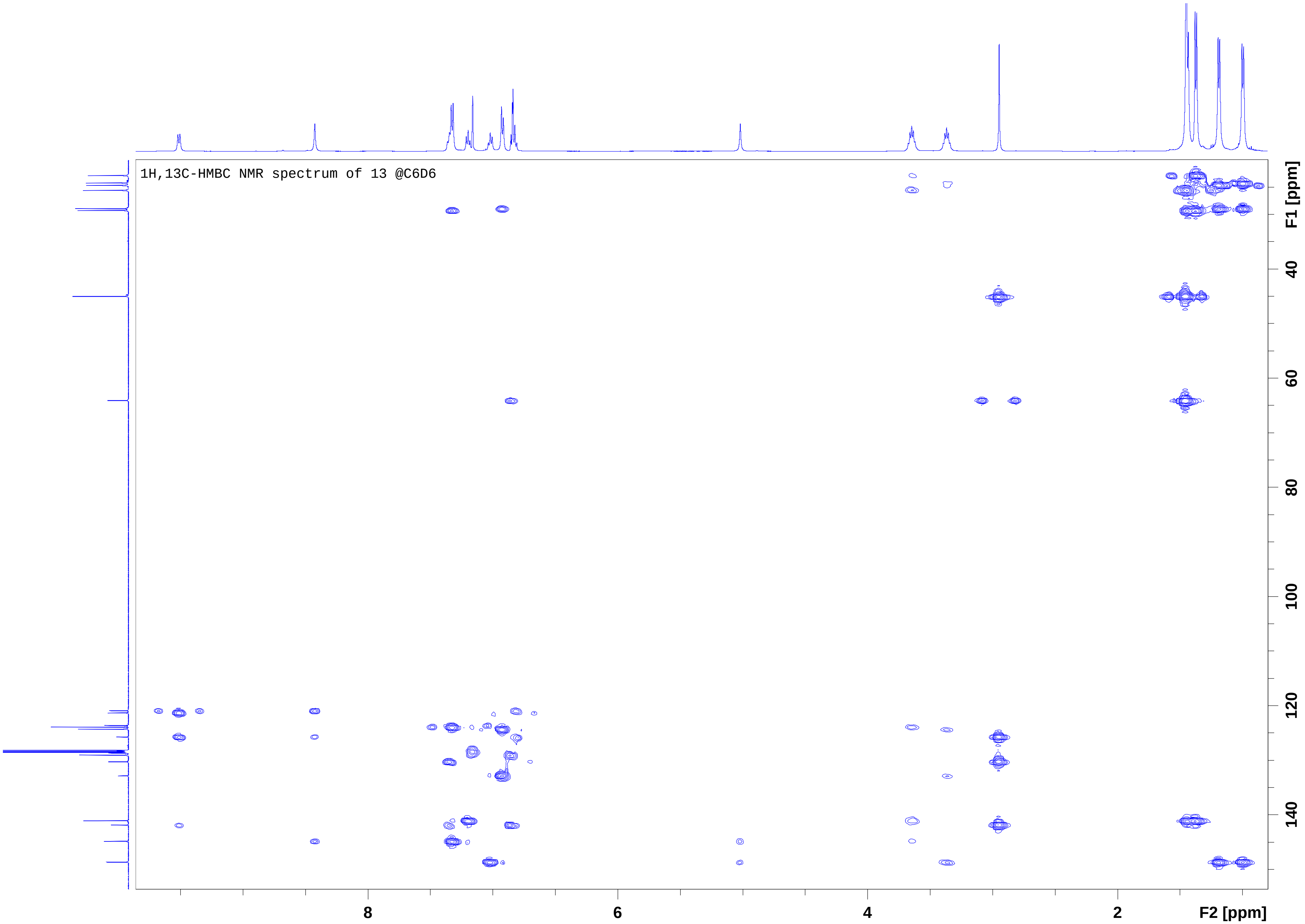

Figure S149. 1H,13C-HMBC NMR spectrum of 13 in C6D6

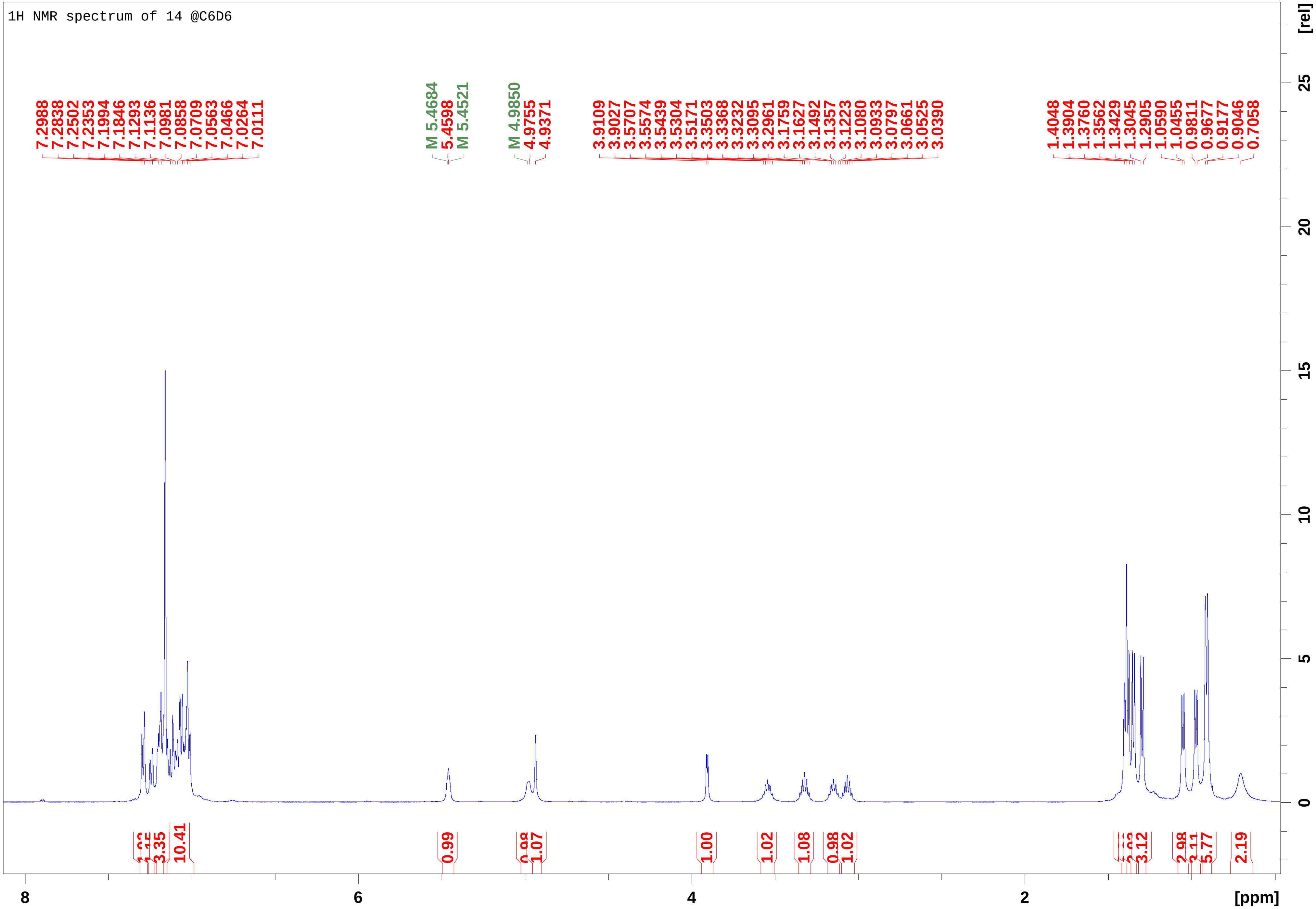

Figure S150. 1H NMR spectrum of 14 in C6D6

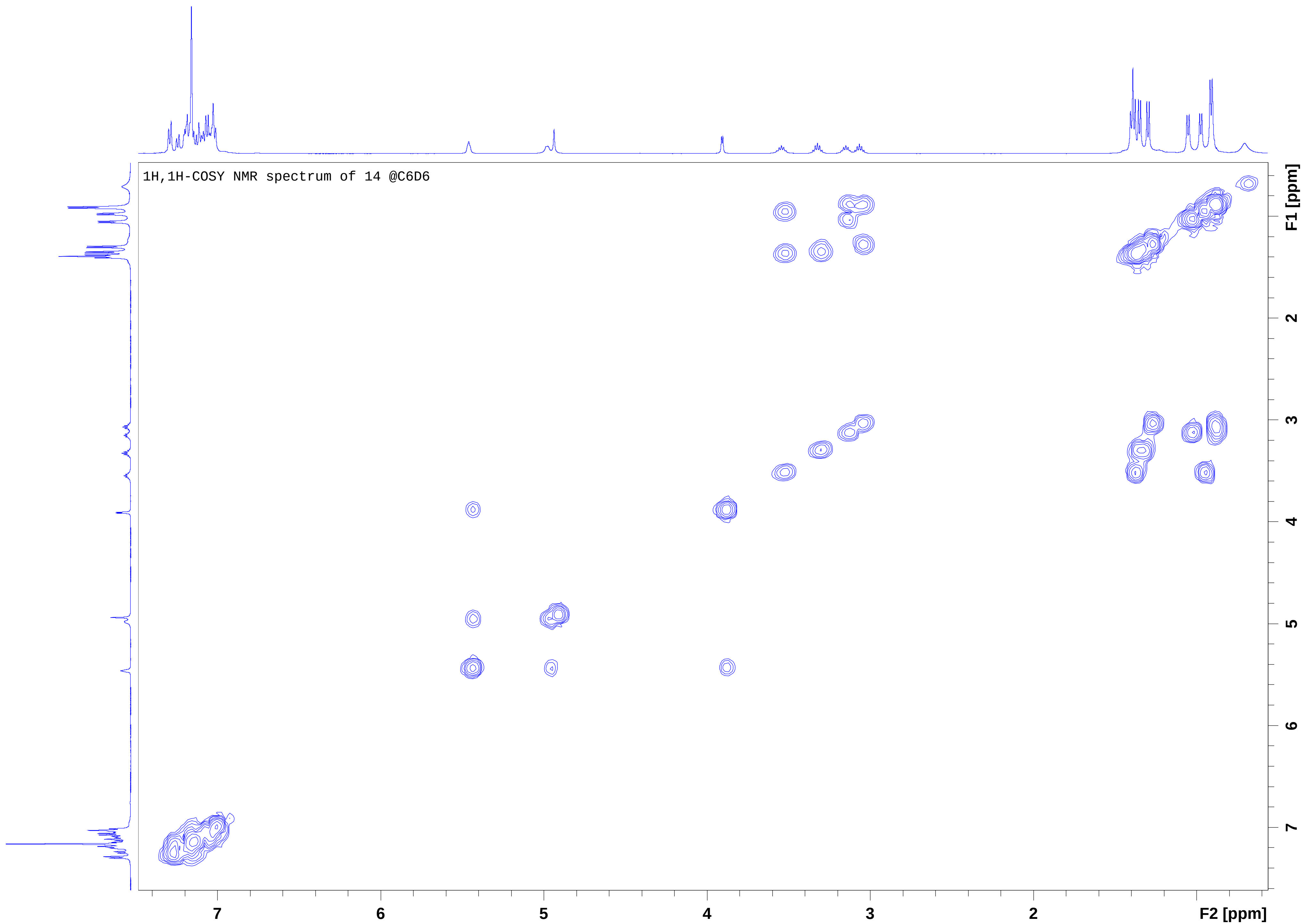

Figure S151. 1H,1H-COSY NMR spectrum of 14 in C6D6

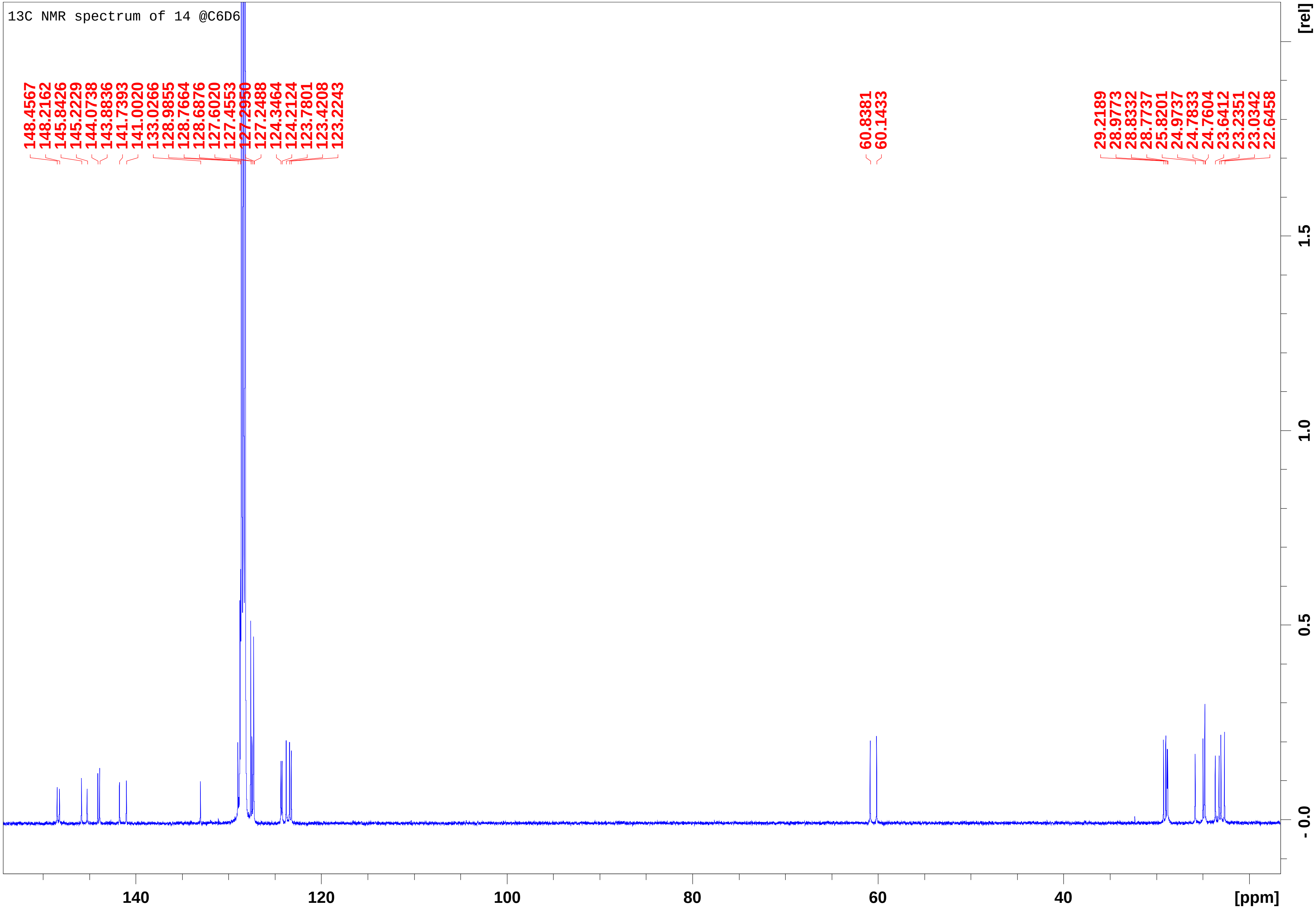

Figure S152. 13C NMR spectrum of 14 in C6D6

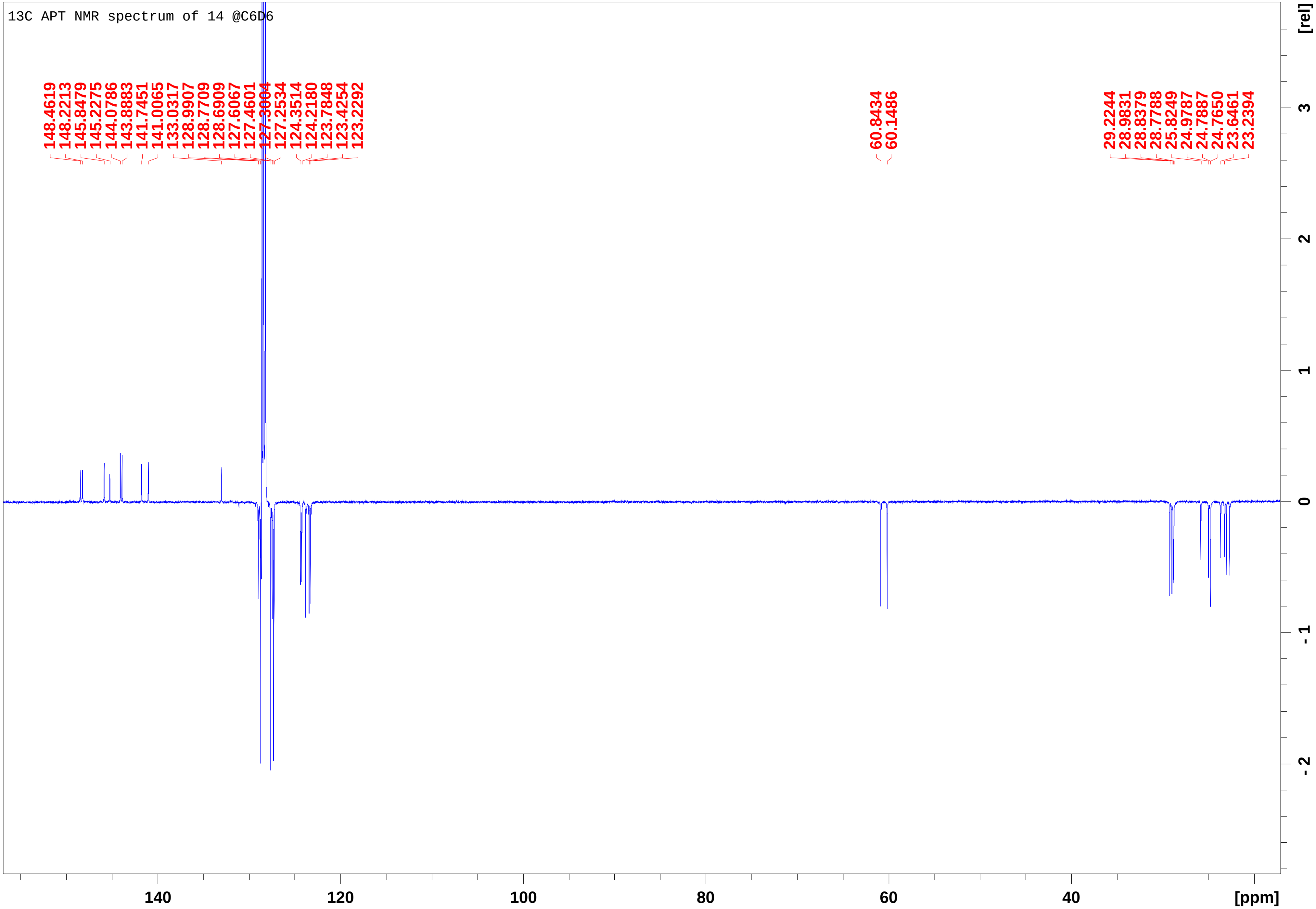

Figure S153. 13C APT NMR spectrum of 14 in C6D6

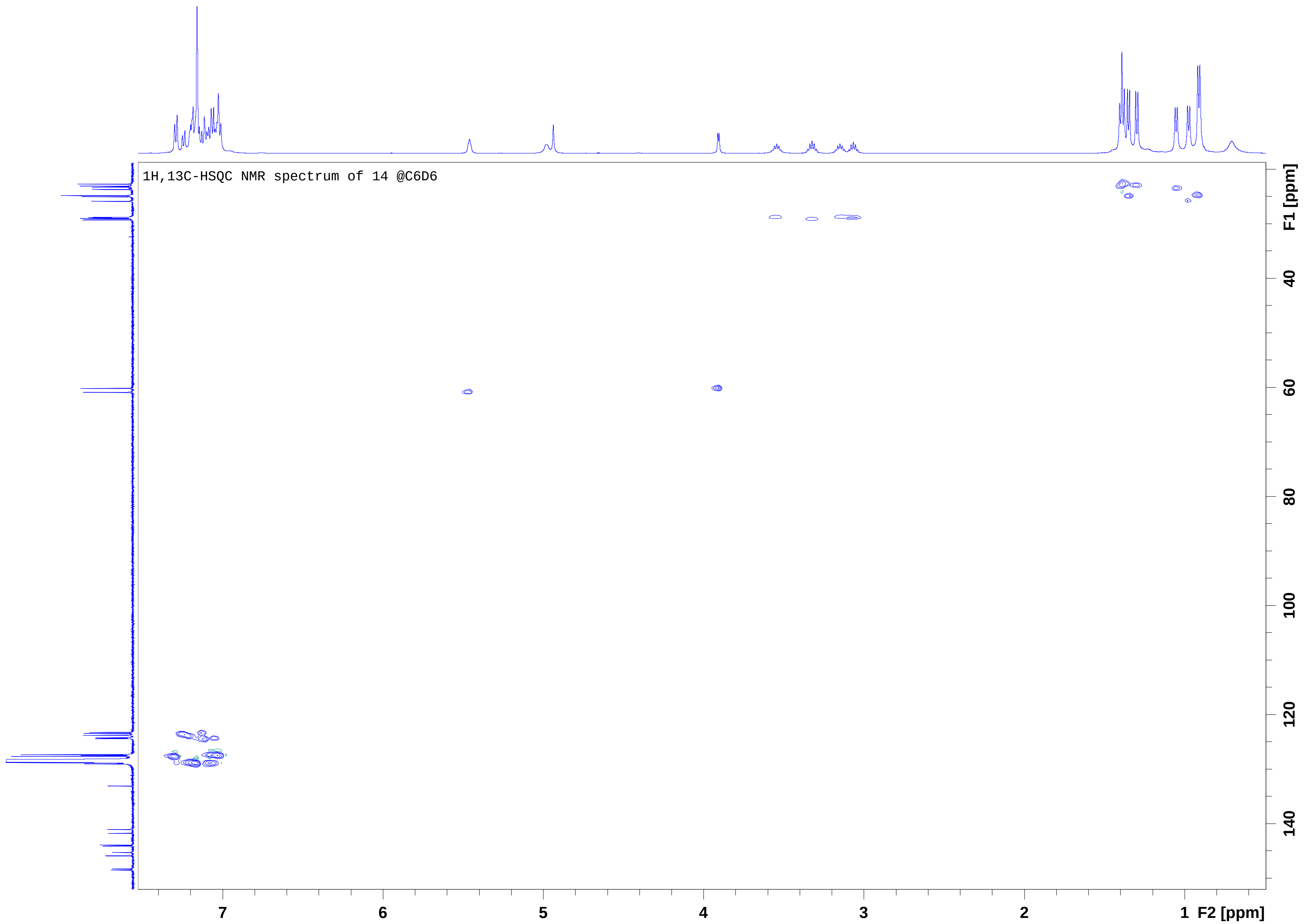

Figure S154. 1H,13C-HSQC NMR spectrum of 14 in C6D6

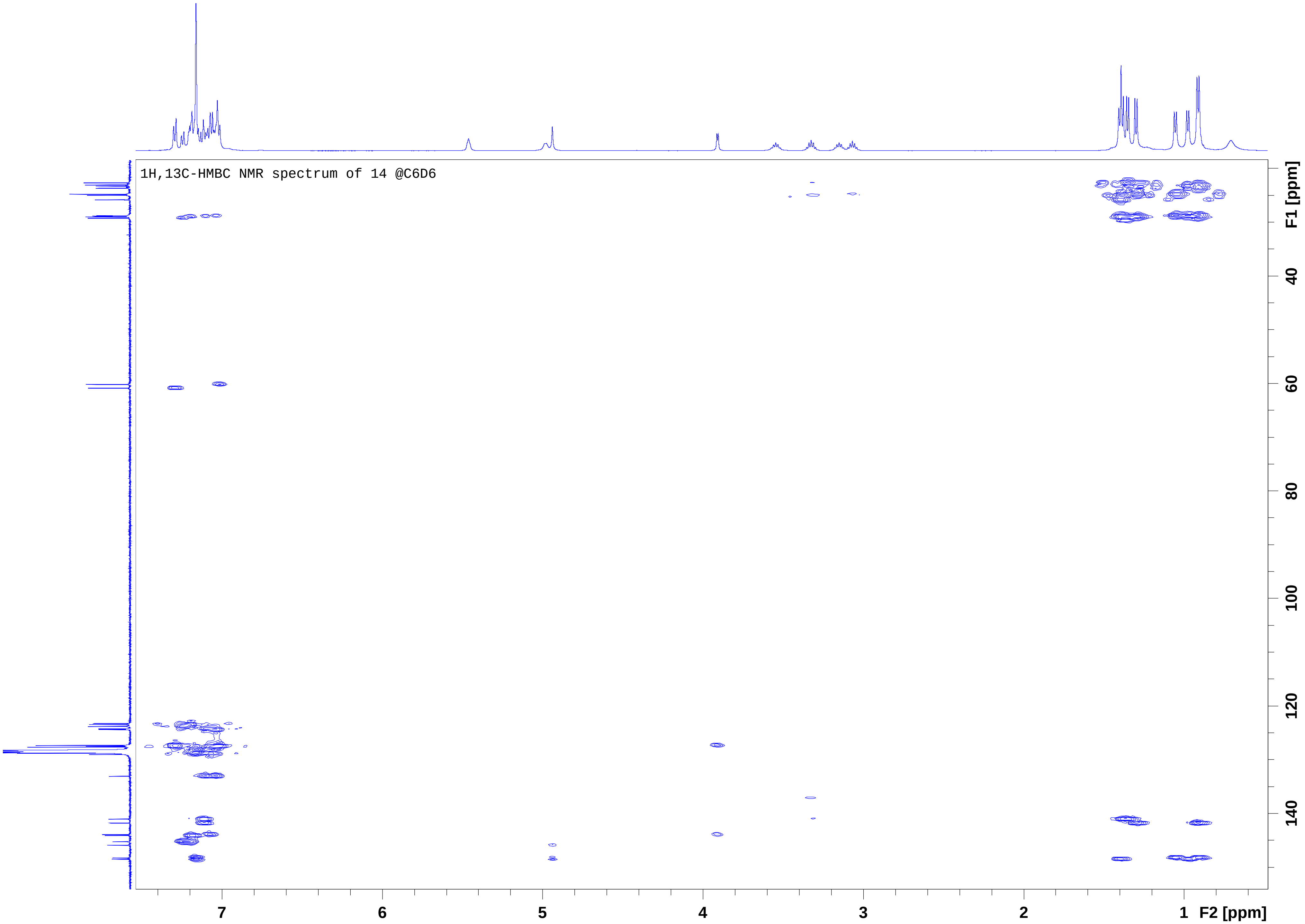

Figure S155. 1H,13C-HMBC NMR spectrum of 14 in C6D6

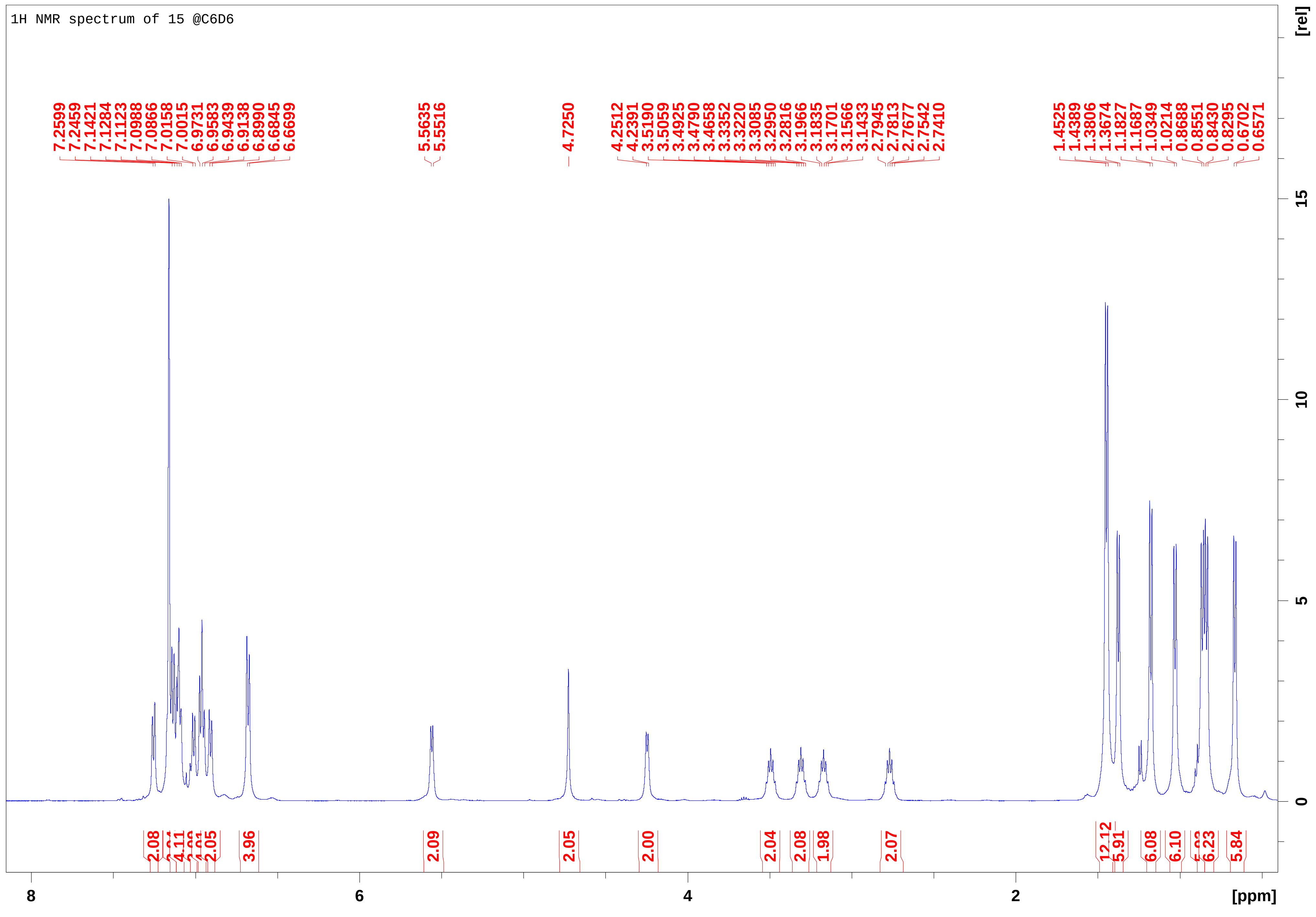

Figure S156. 1H NMR spectrum of 15 in C6D6

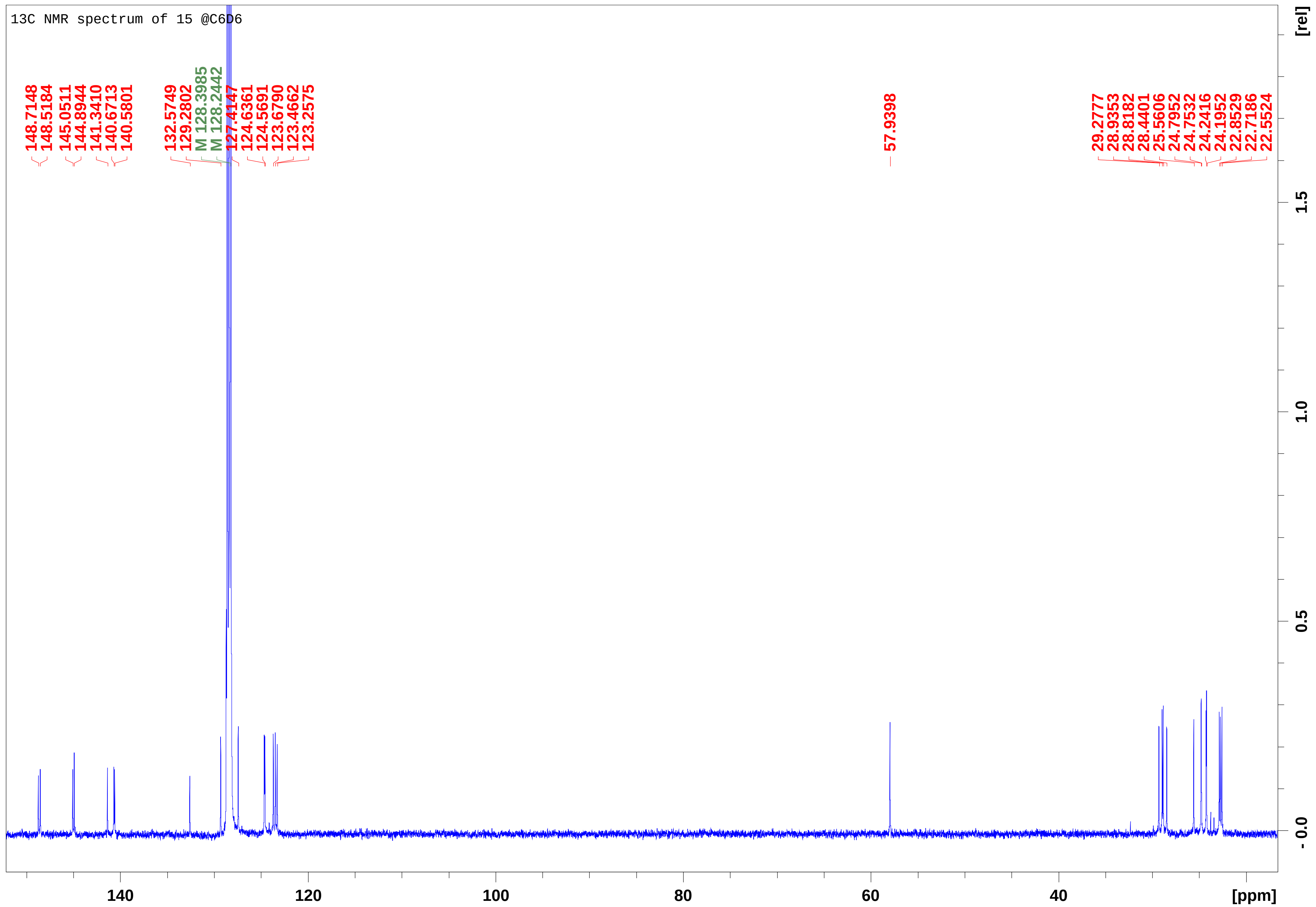

Figure S157. 13C NMR spectrum of 15 in C6D6

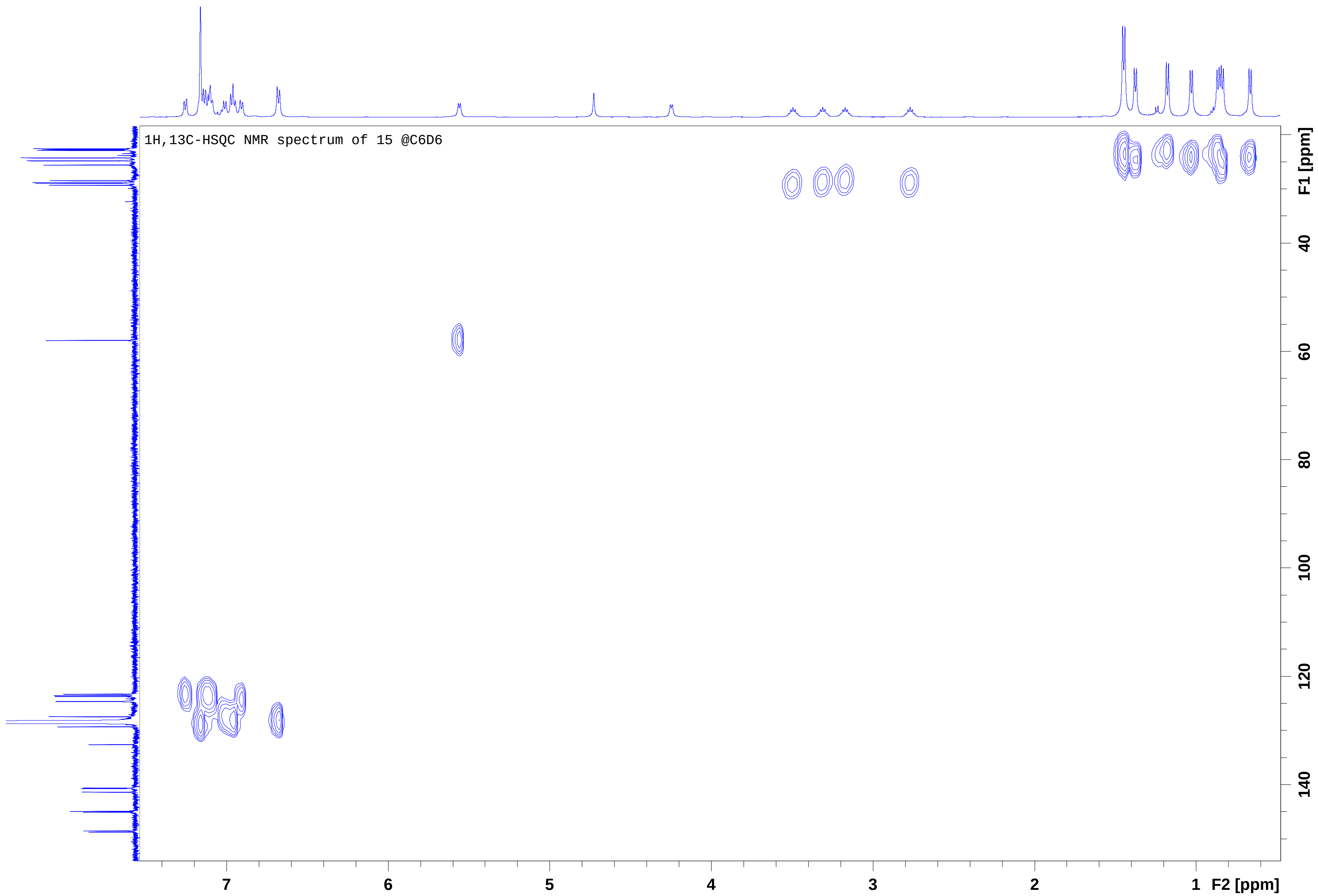

Figure S158. 1H,13C-HSQC NMR spectrum of 15 in C6D6

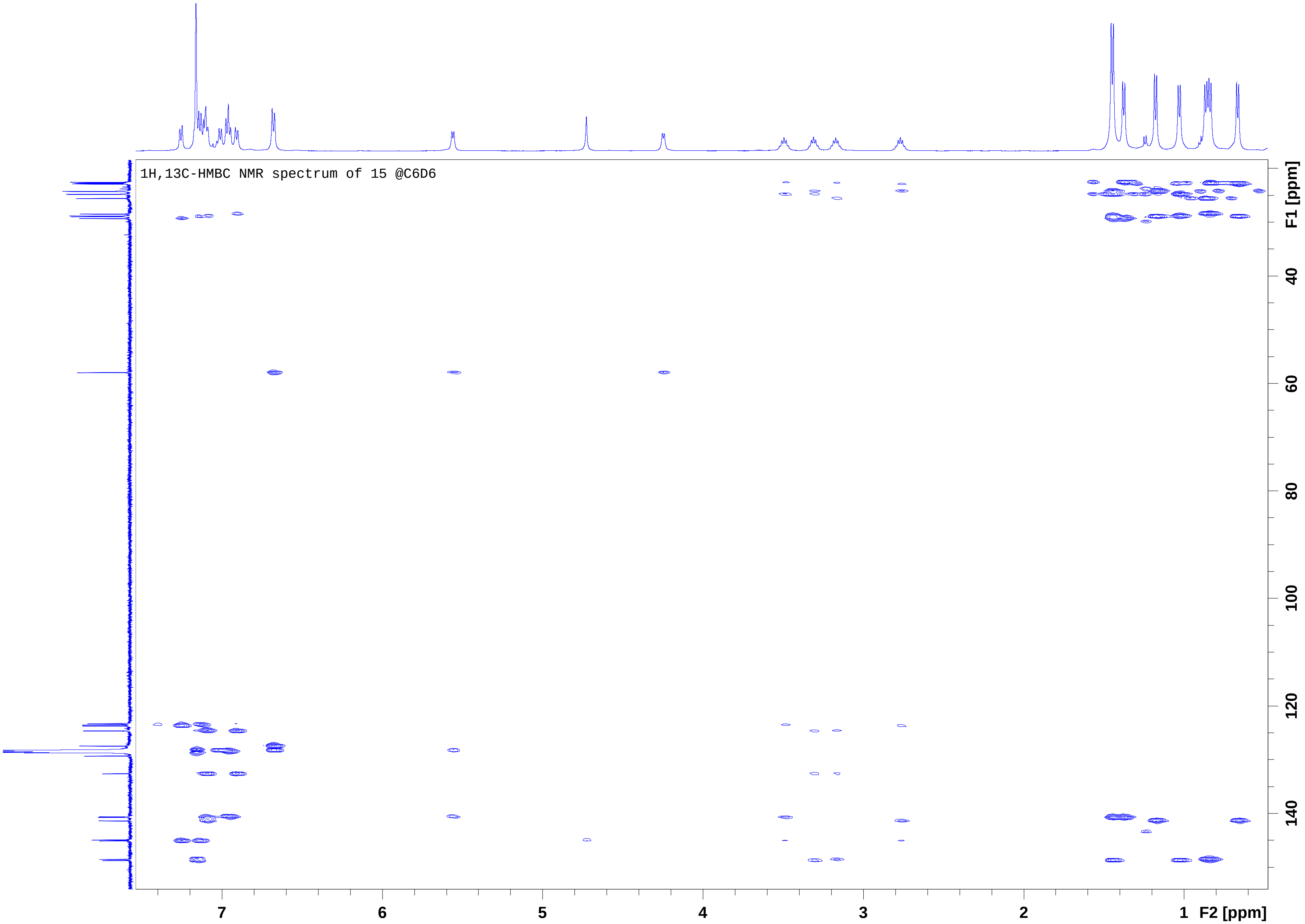

Figure S159. 1H,13C-HMBC NMR spectrum of 15 in C6D6

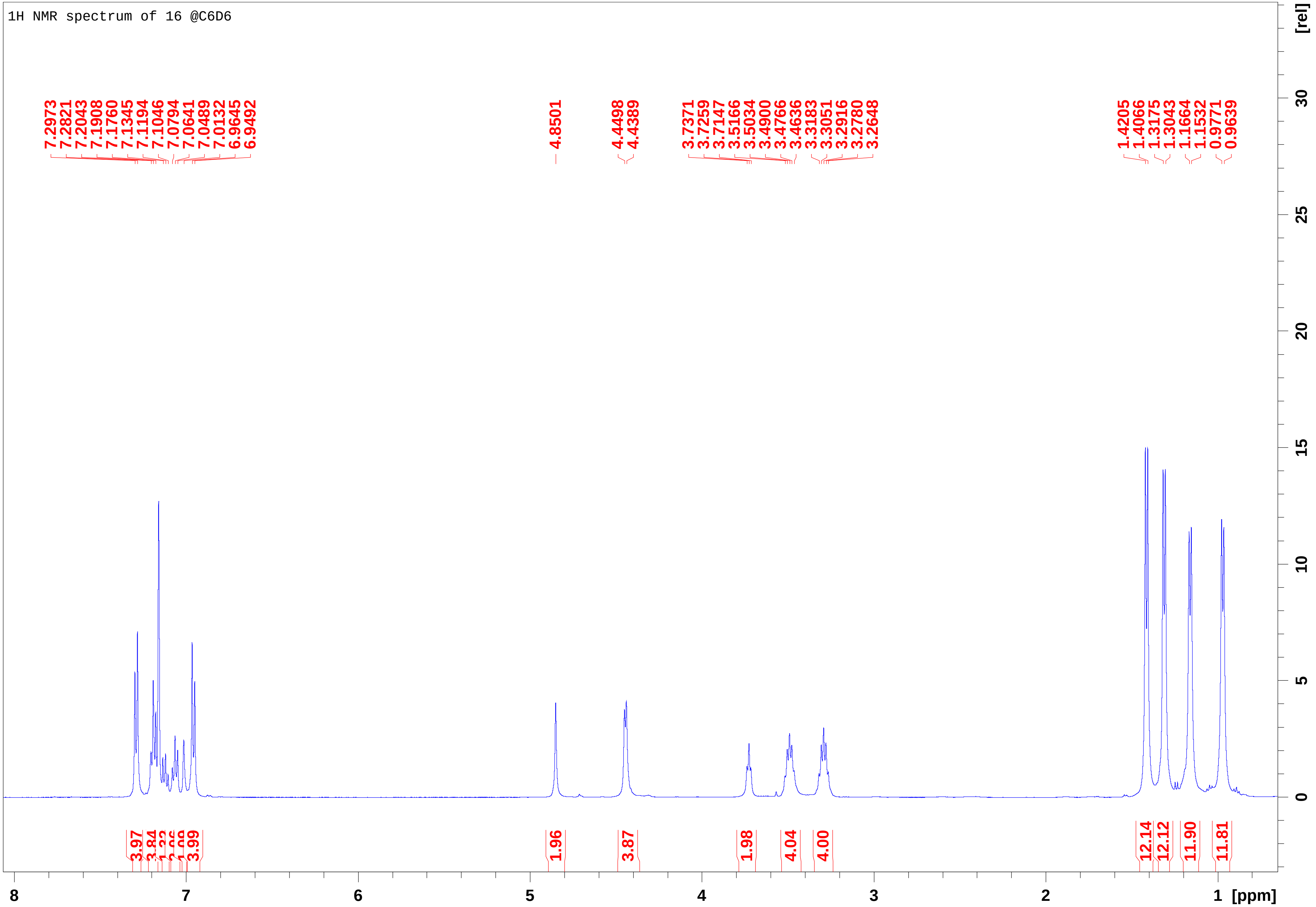

Figure S160. 1H NMR spectrum of 16 in C6D6

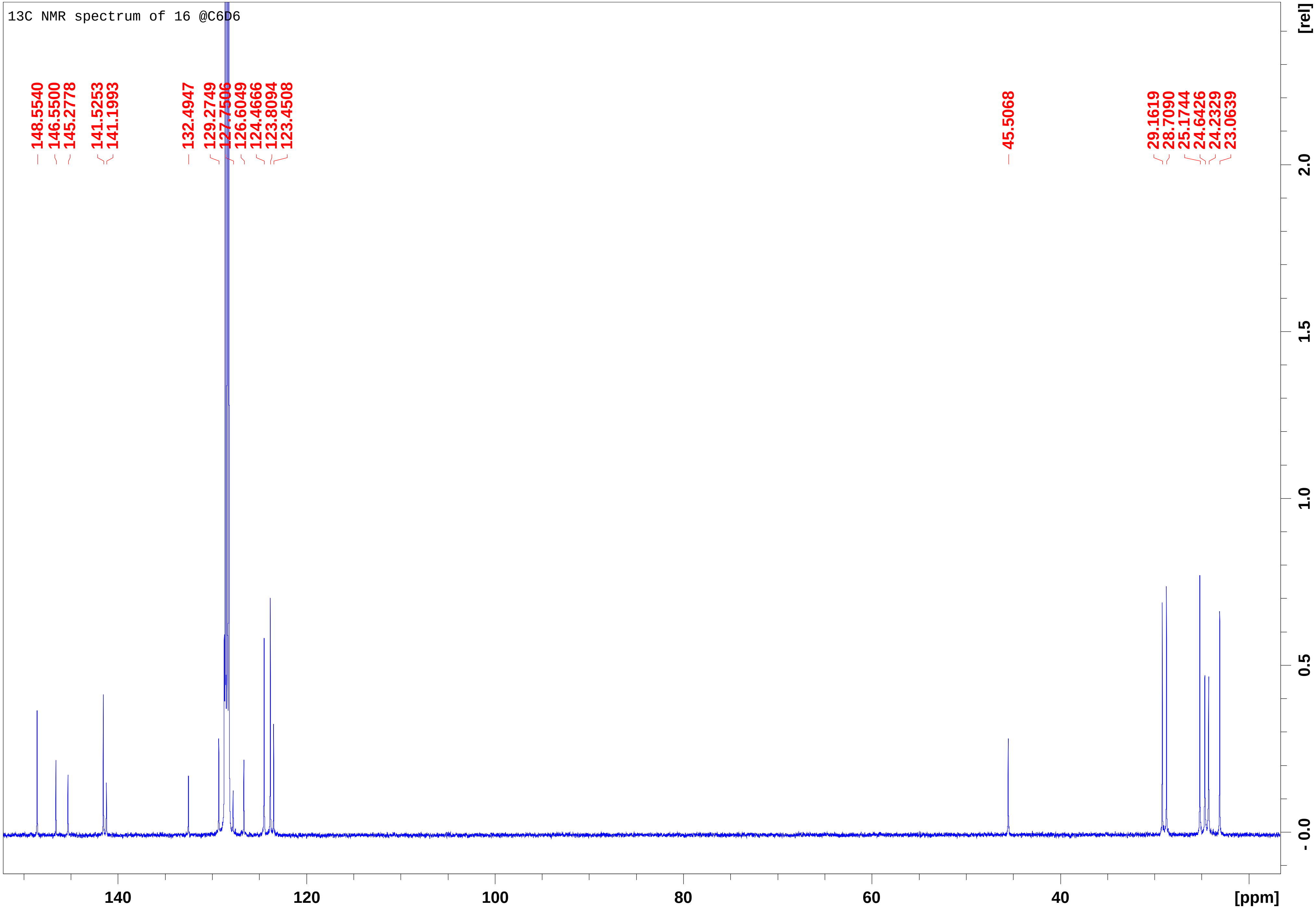

Figure S161. 13C NMR spectrum of 16 in C6D6

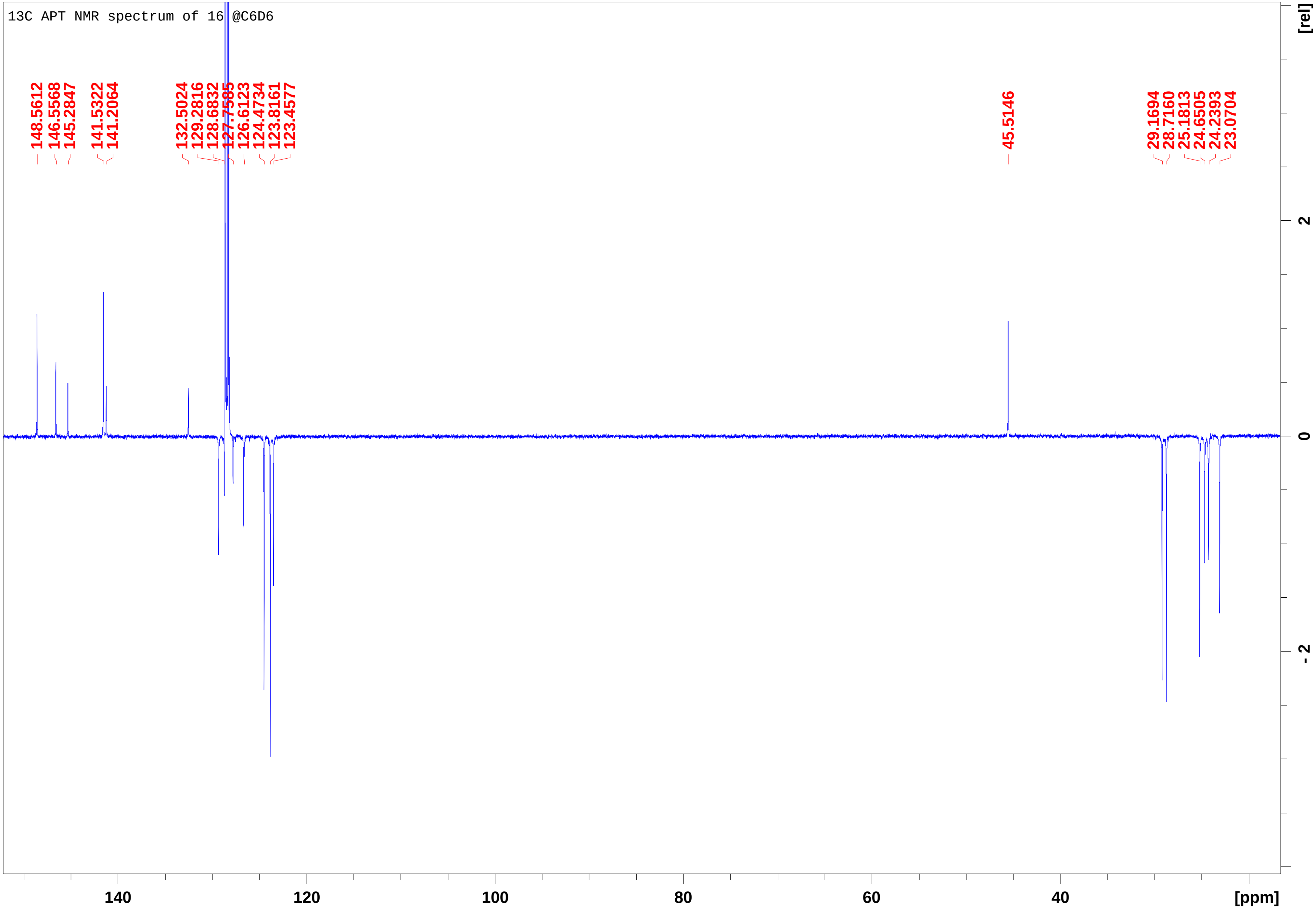

Figure S162. 13C APT NMR spectrum of 16 in C6D6

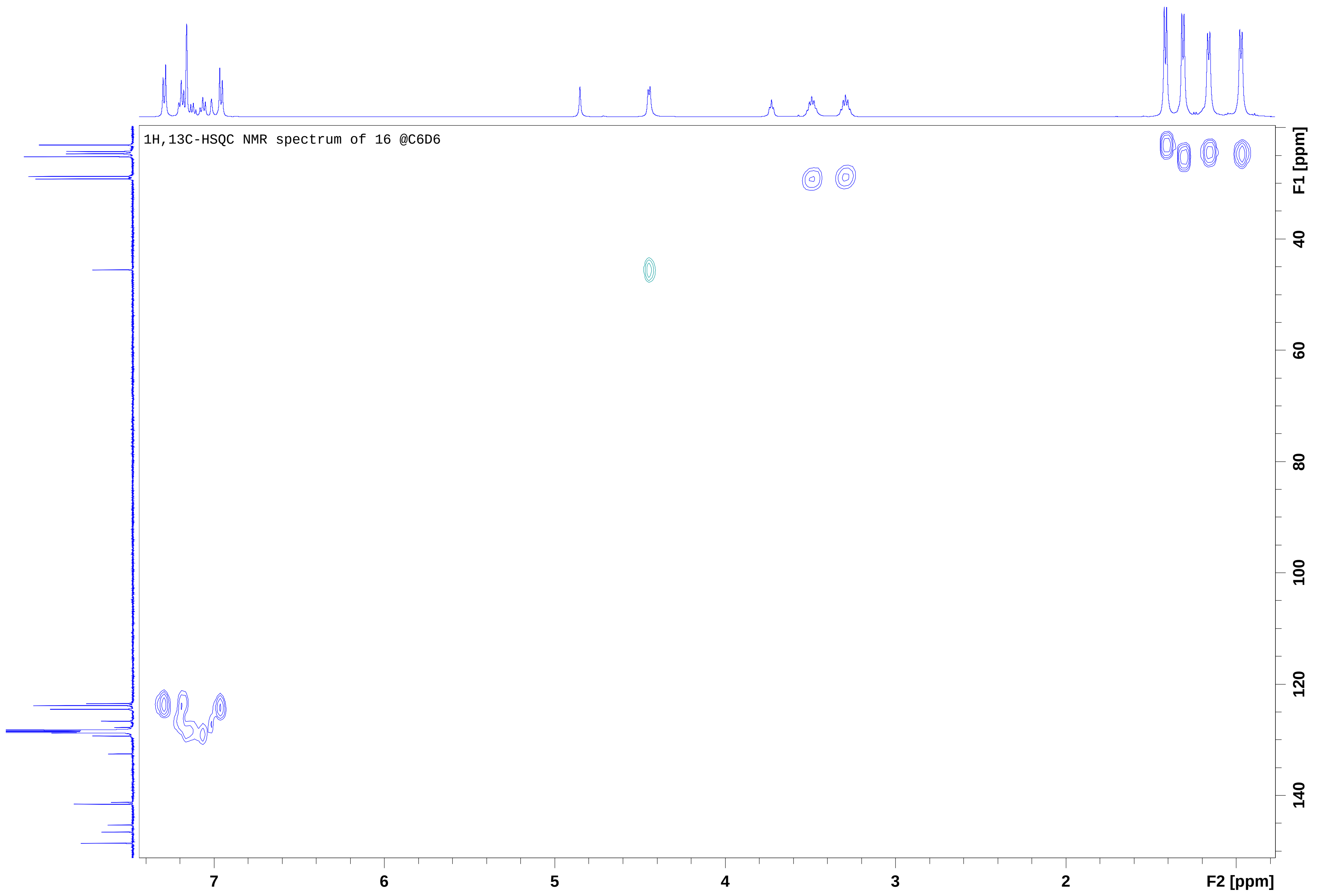

Figure S163. 1H,13C-HSQC NMR spectrum of 16 in C6D6

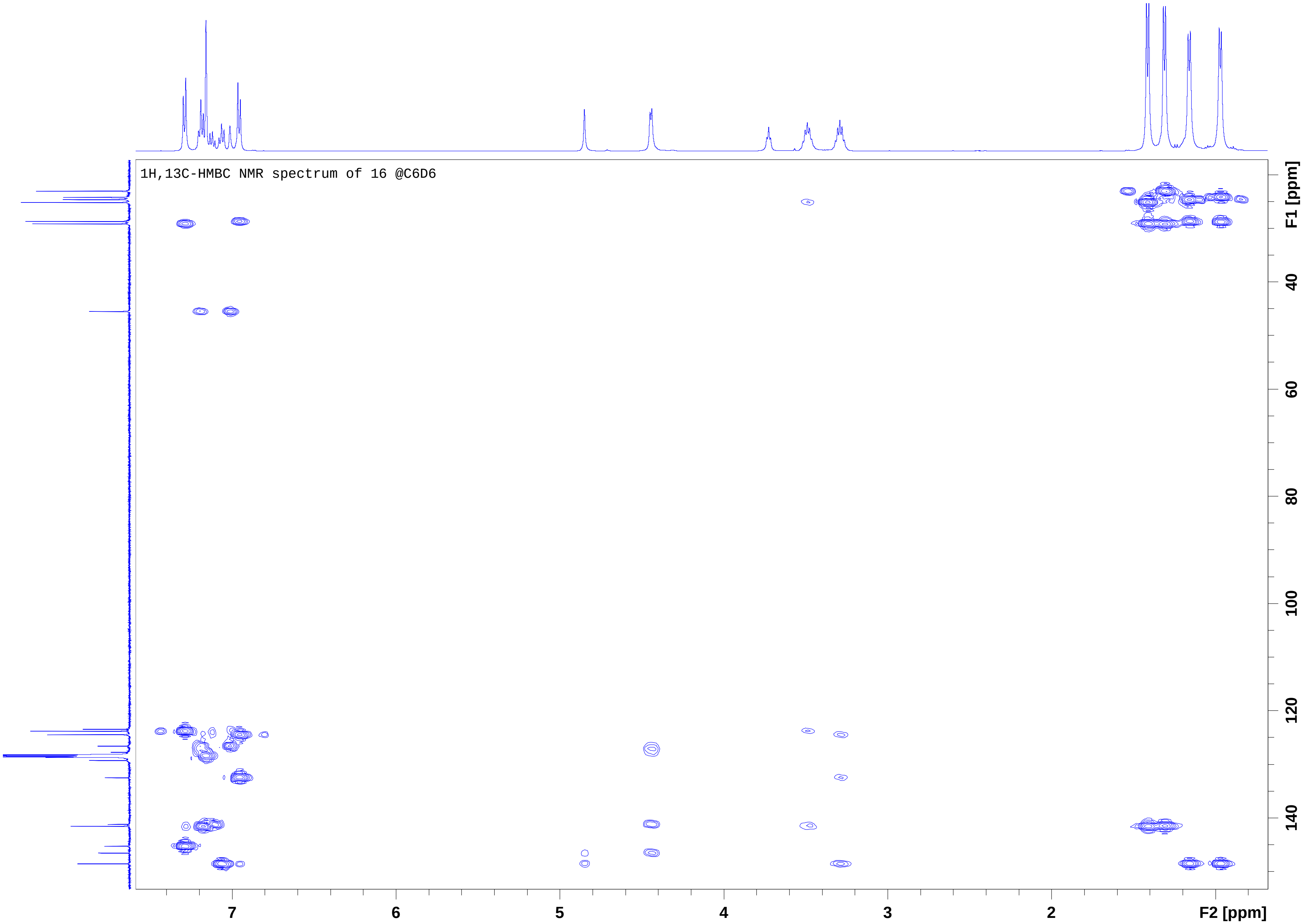

Figure S164. 1H,13C-HMBC NMR spectrum of 16 in C6D6

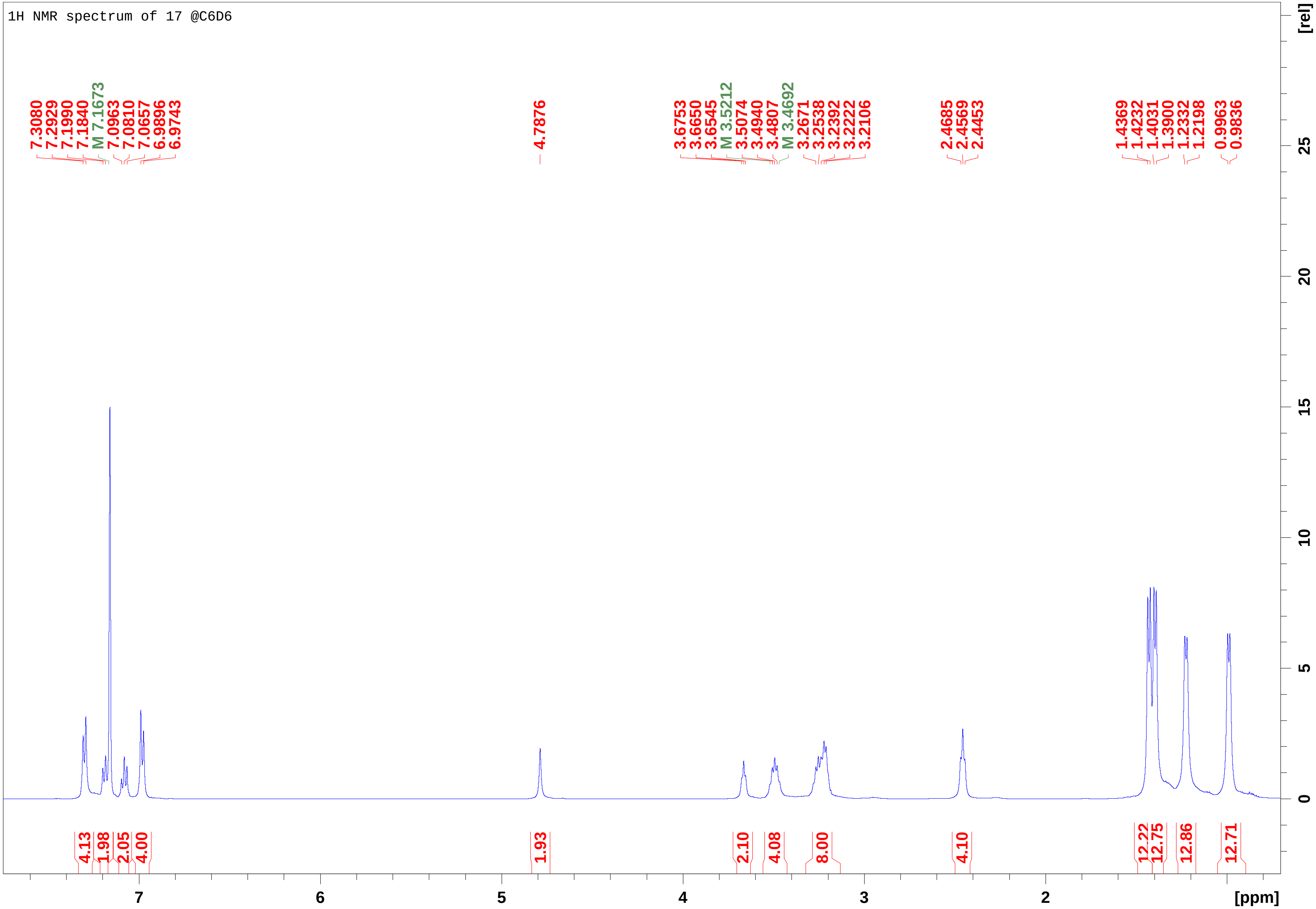

Figure S165. 1H NMR spectrum of 17 in C6D6

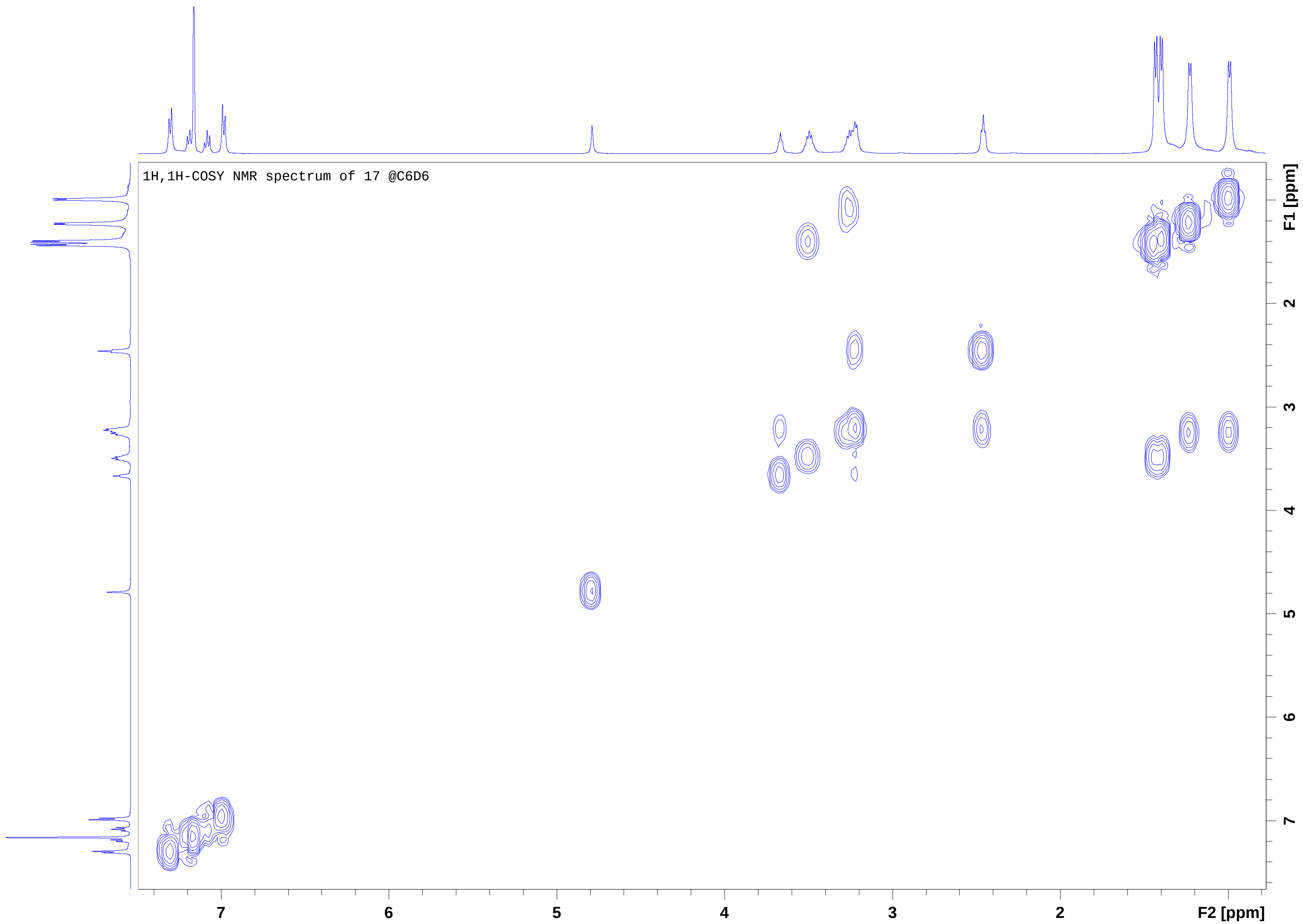

Figure S166. 1H,1H-COSY NMR spectrum of 17 in C6D6

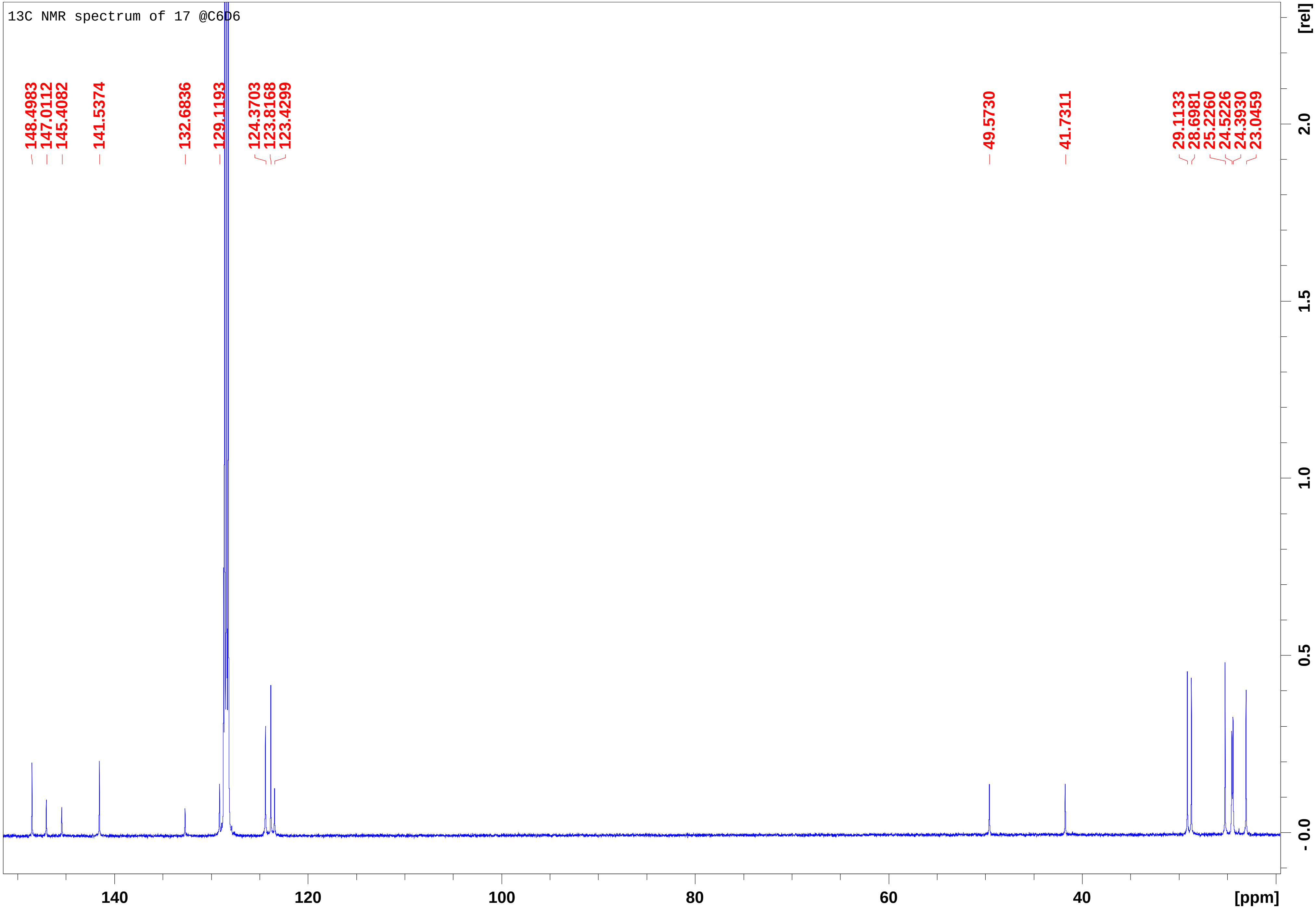

Figure S167. 13C NMR spectrum of 17 in C6D6

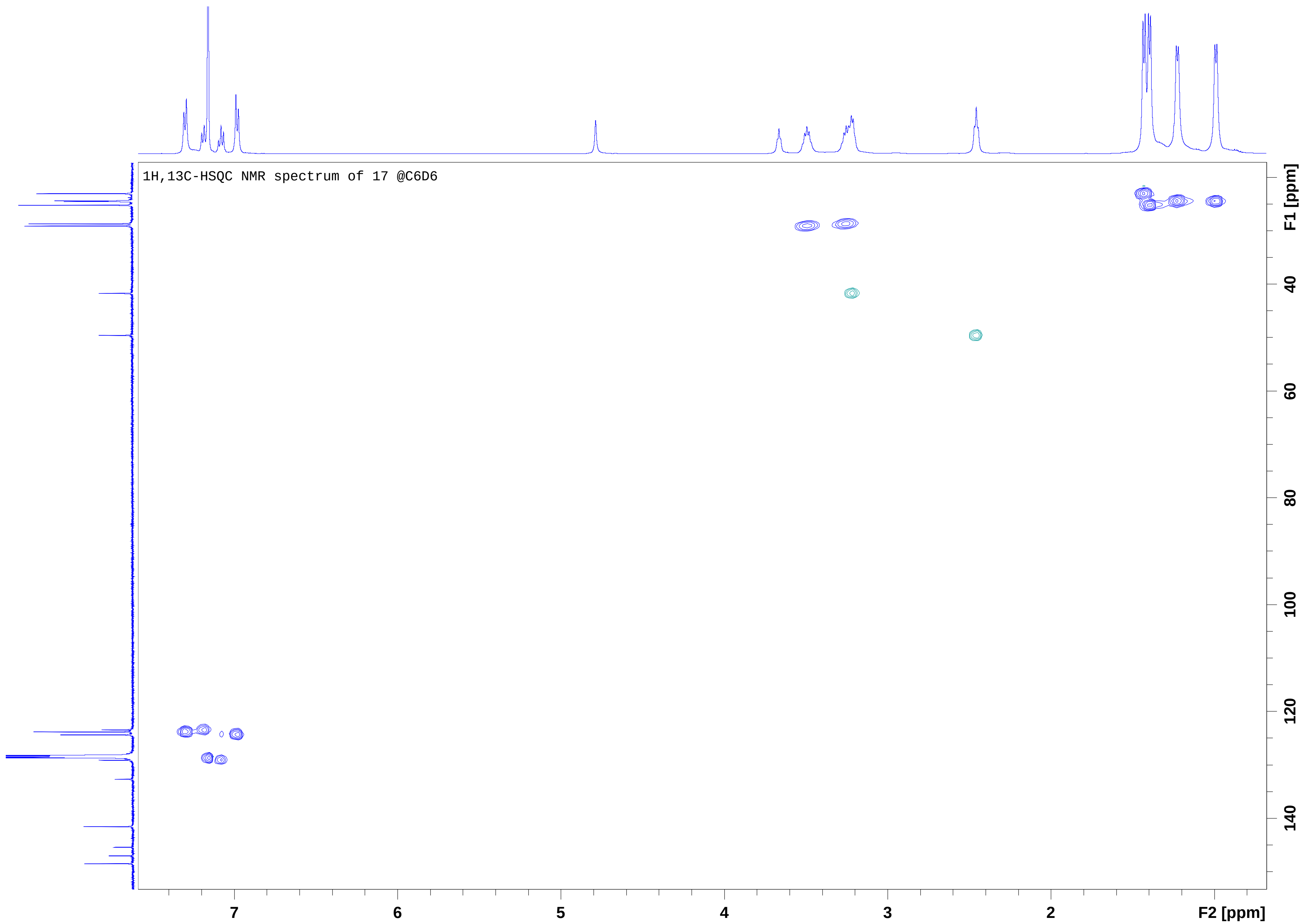

Figure S168. 1H,13C-HSQC NMR spectrum of 17 in C6D6

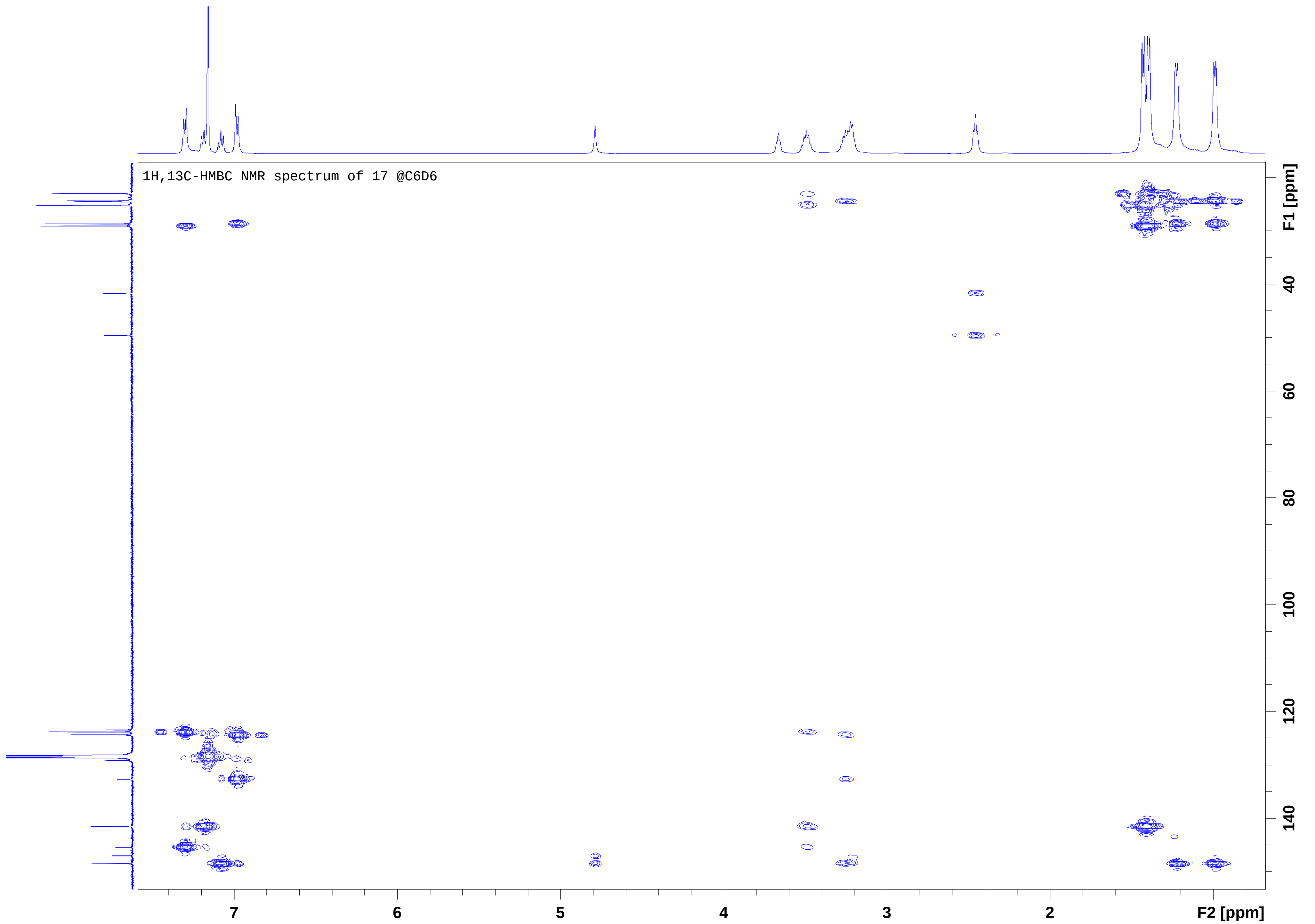

Figure S169. 1H,13C-HMBC NMR spectrum of 17 in C6D6

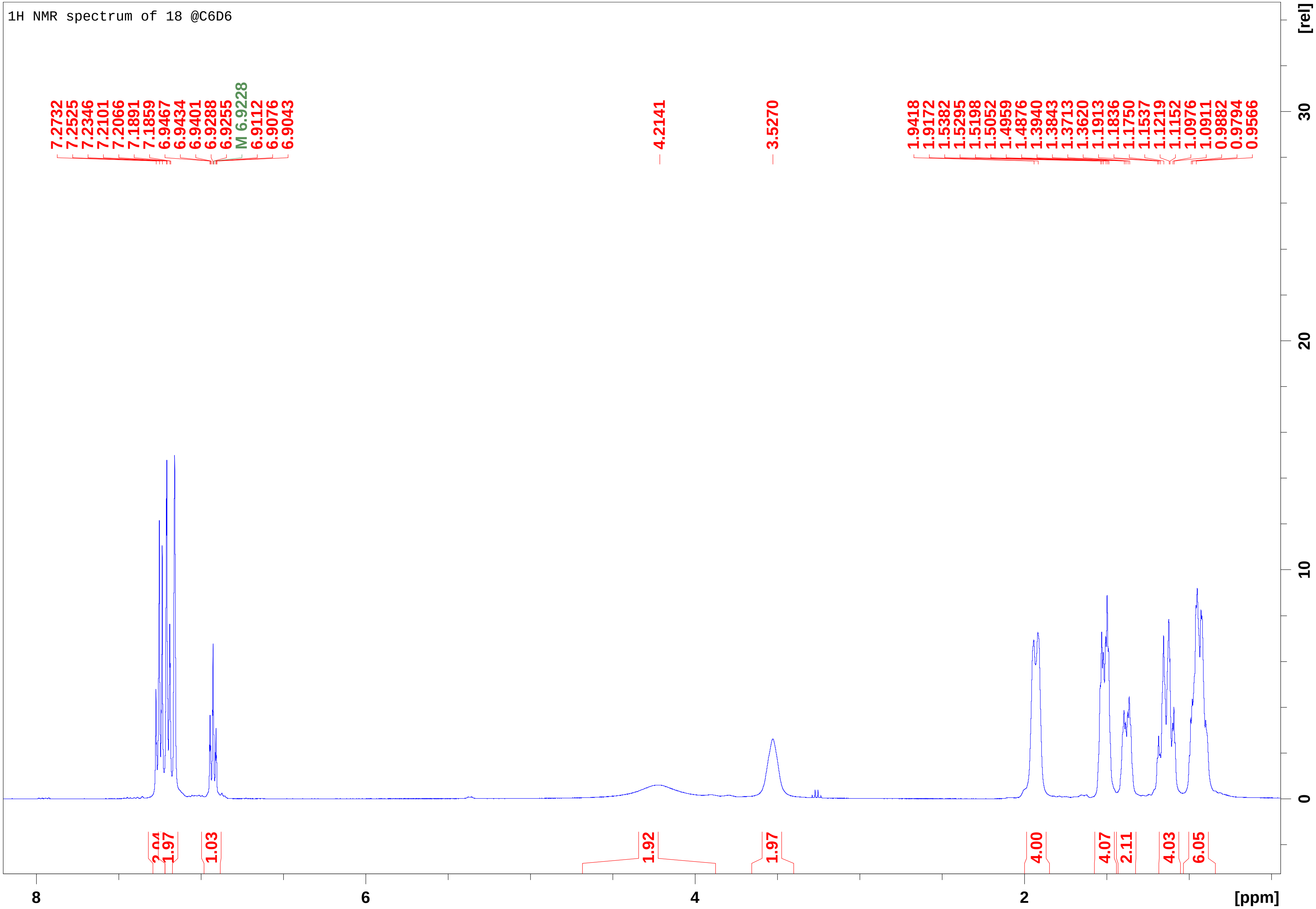

Figure S170. 1H NMR spectrum of 18 in C6D6

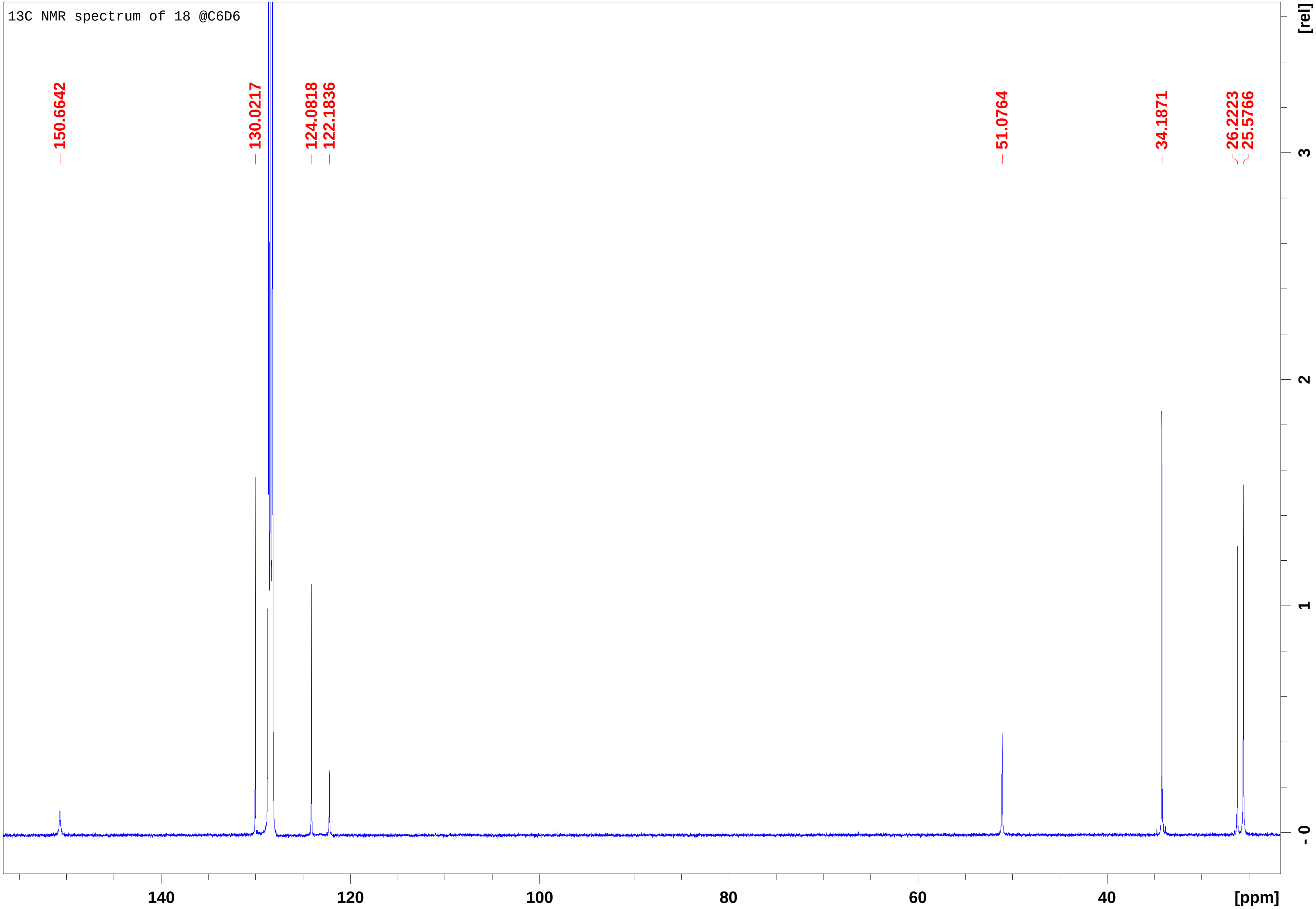

Figure S171. 13C NMR spectrum of 18 in C6D6

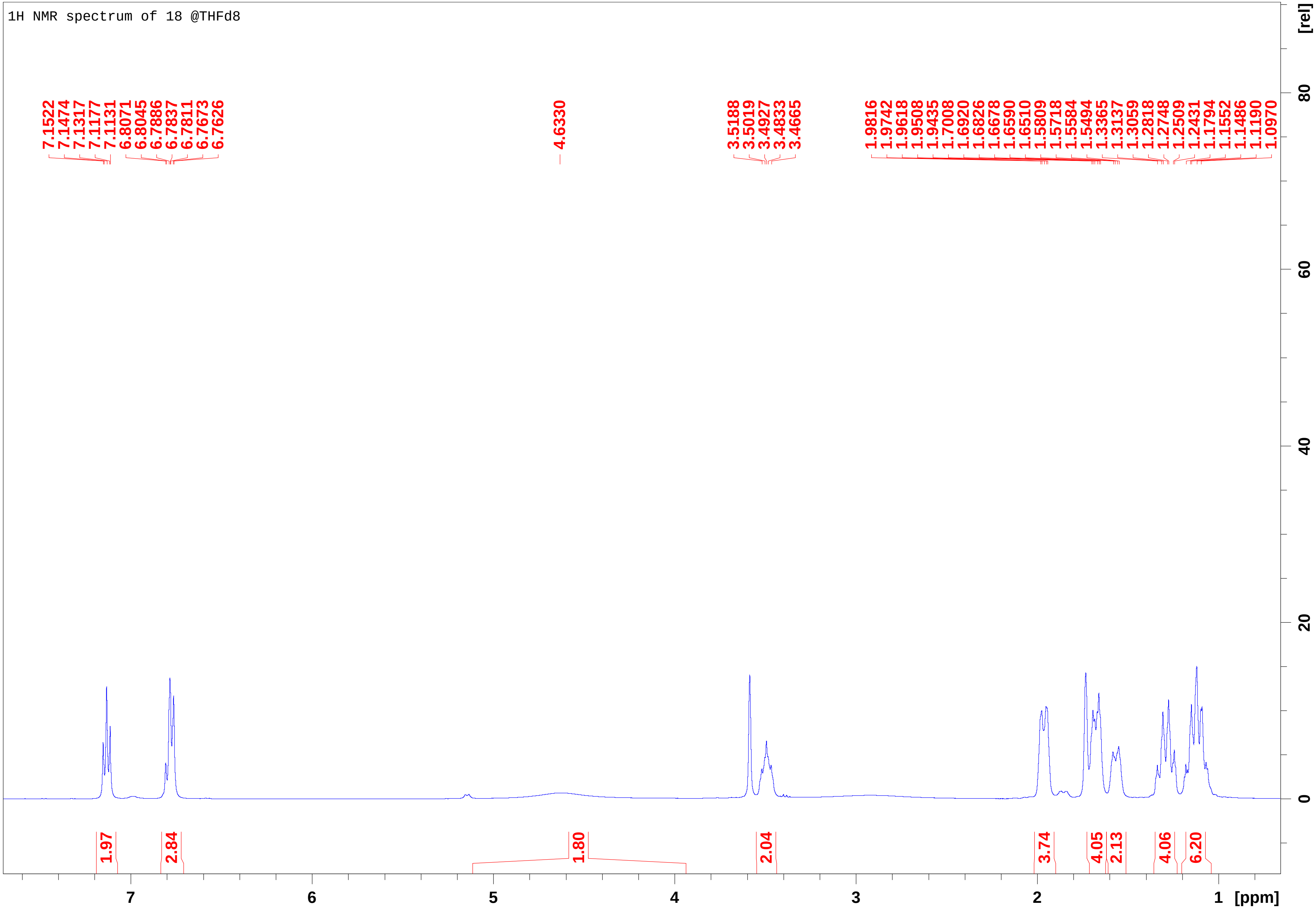

Figure S172. 1H NMR spectrum of 18 in THF-d8

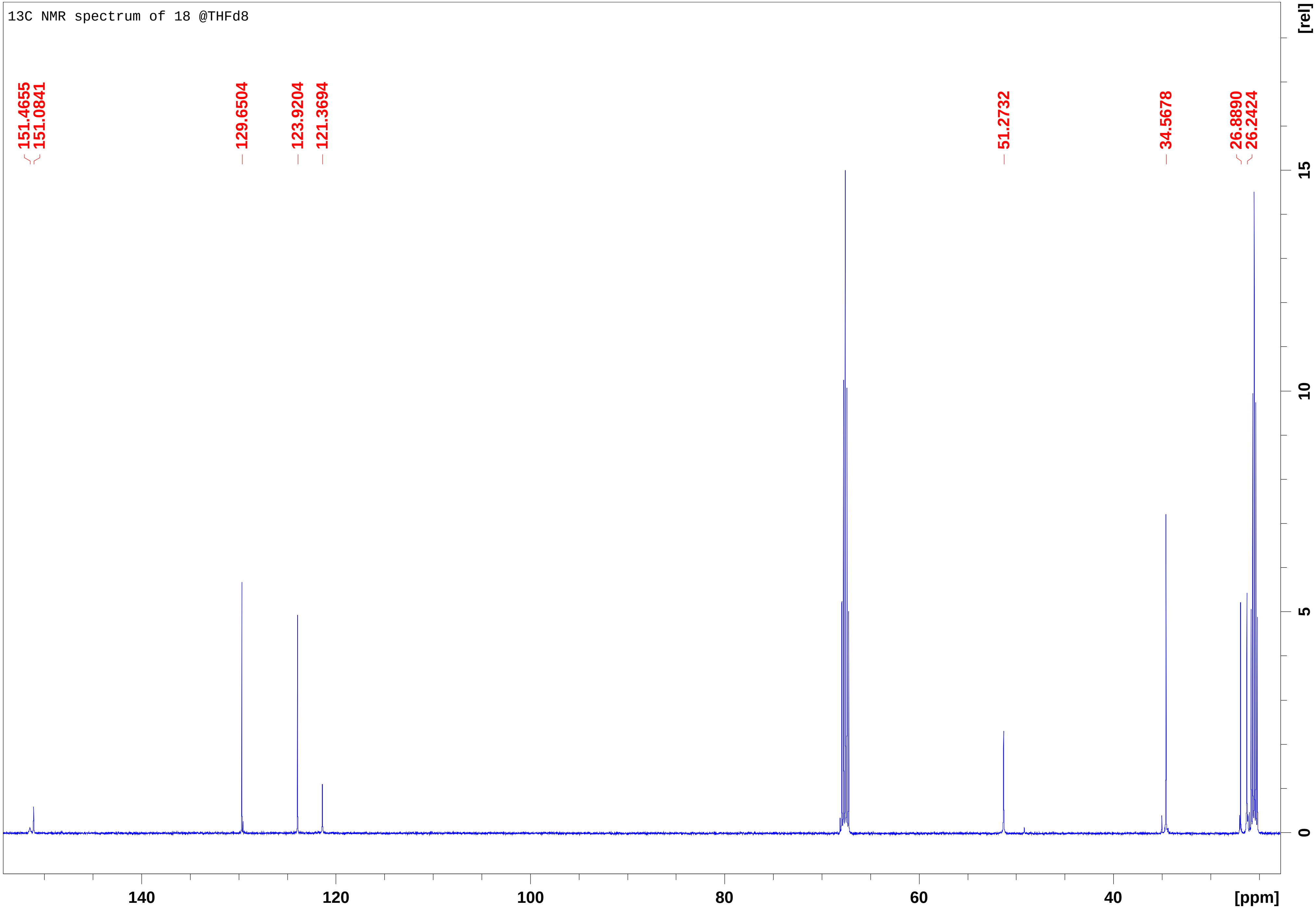

Figure S173. 13C NMR spectrum of 18 in THF-d8

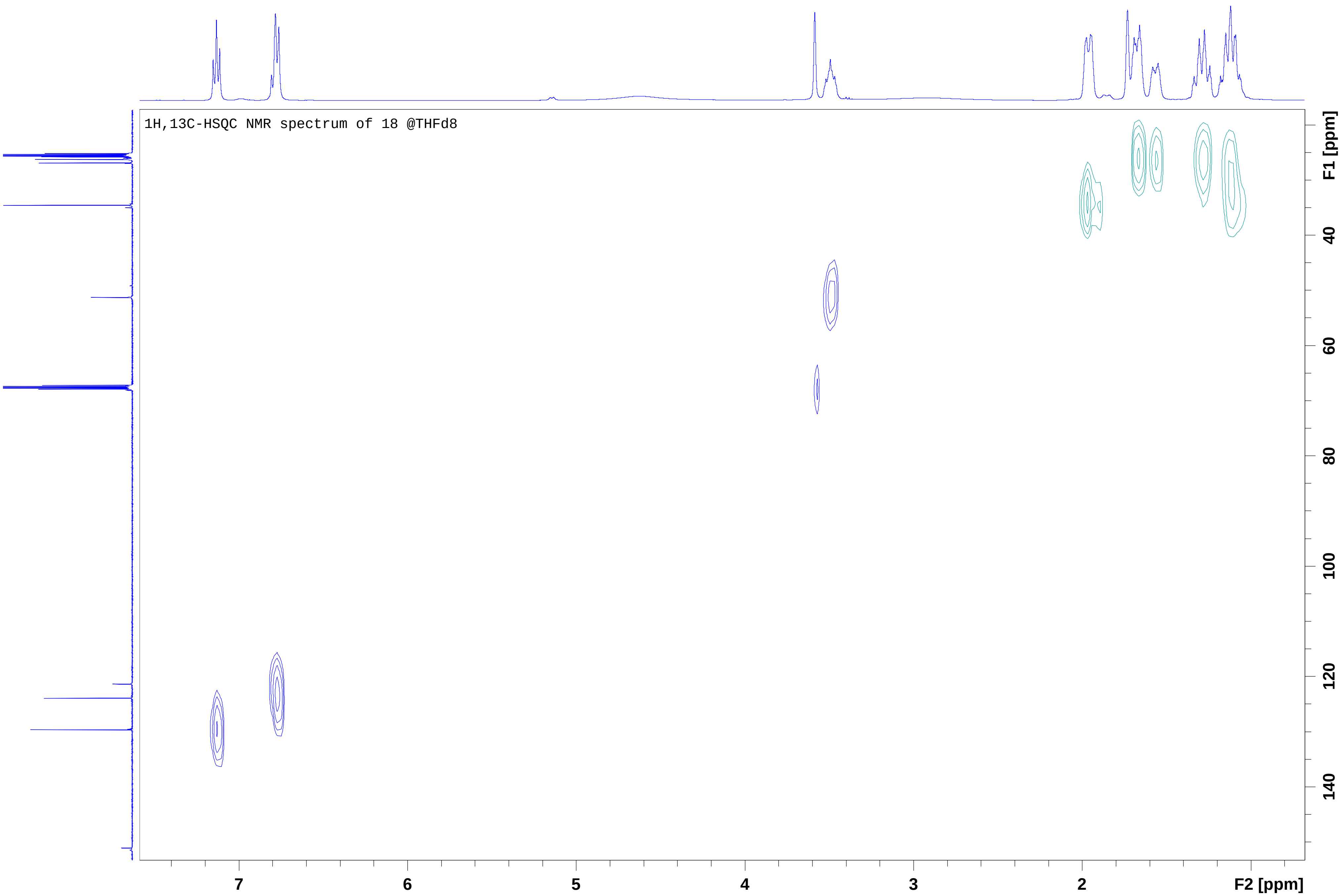

Figure S174. 1H,13C-HSQC NMR spectrum of 18 in THF-d8

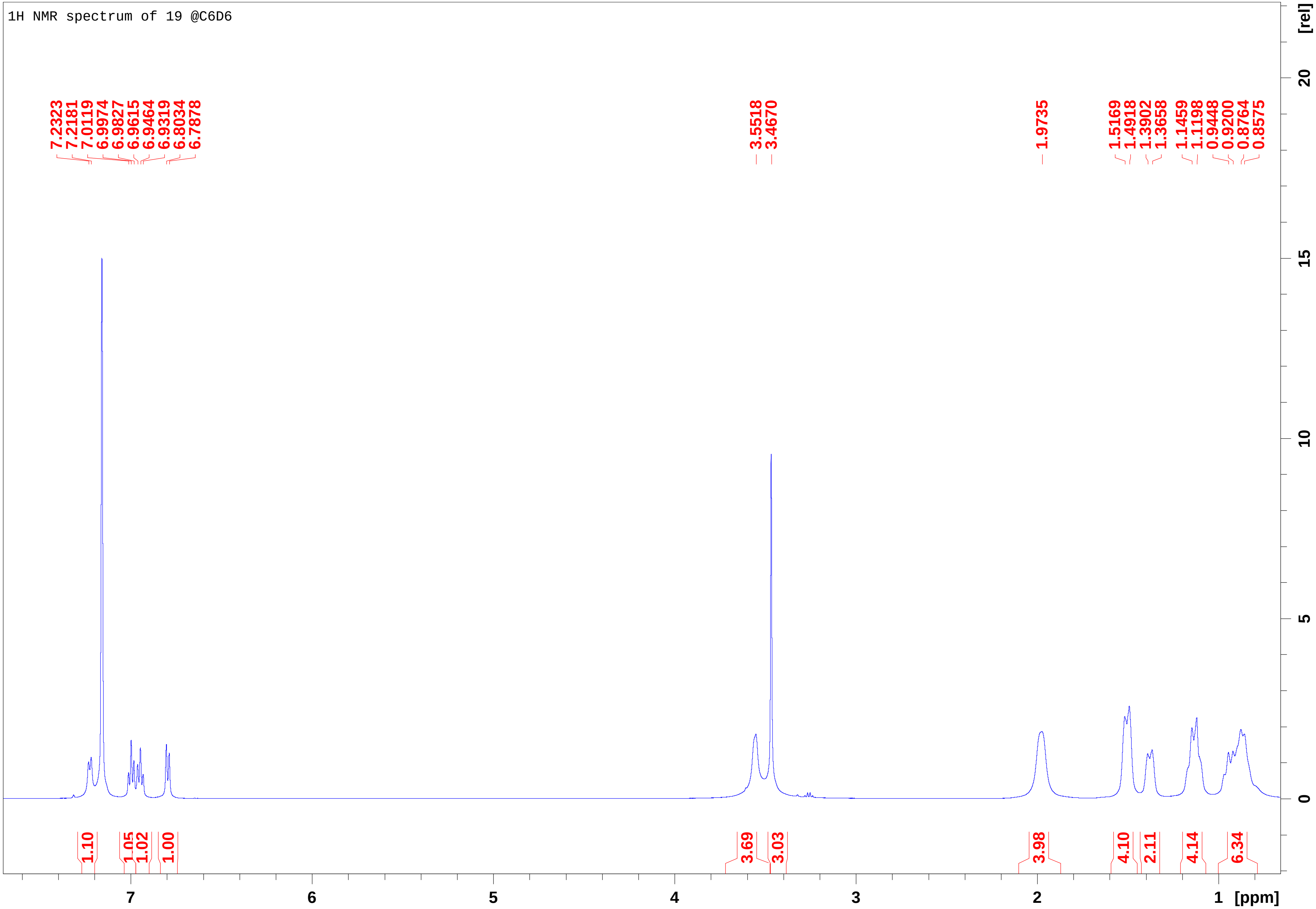

Figure S175. 1H NMR spectrum of 19 in C6D6

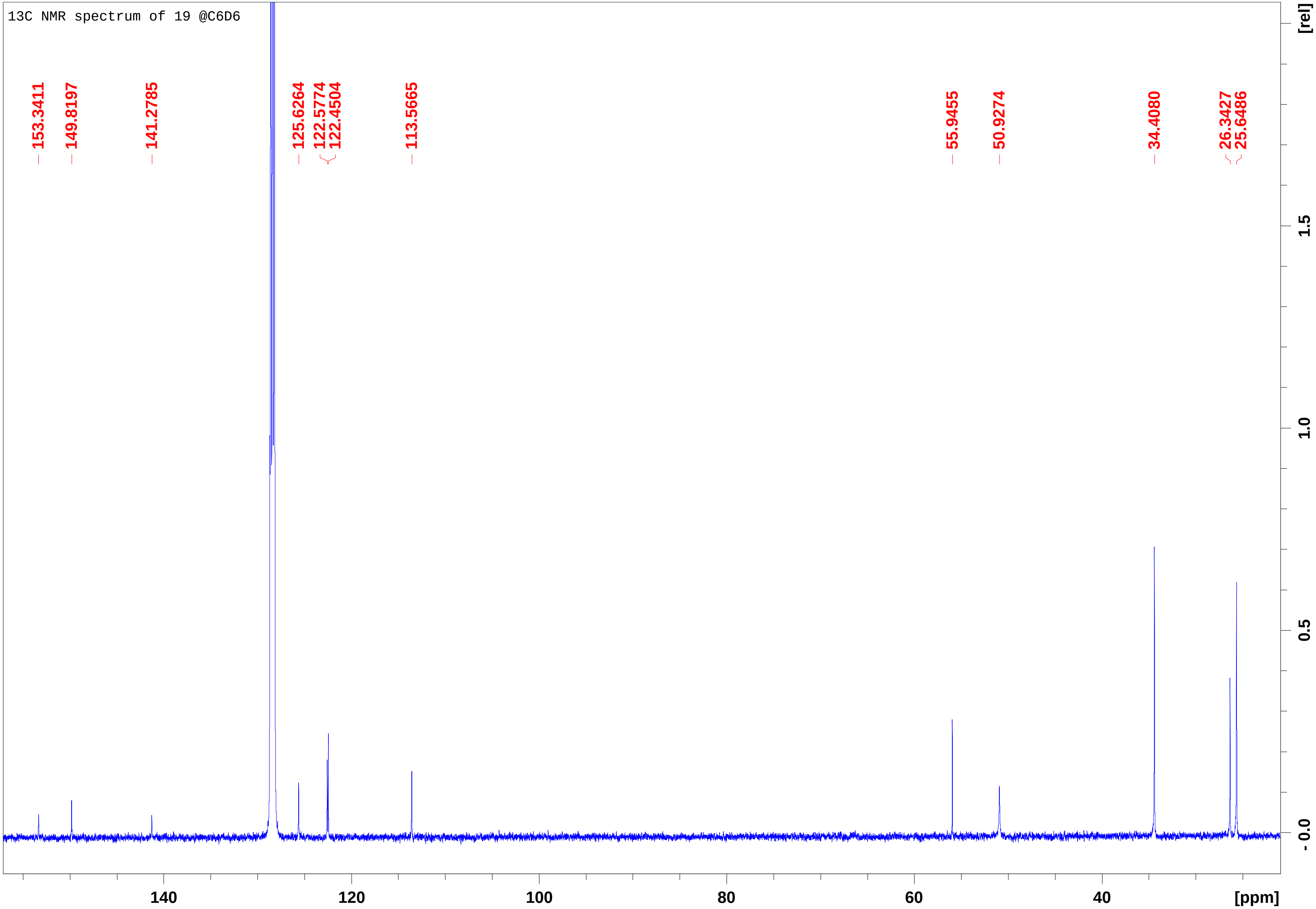

Figure S176. 13C NMR spectrum of 19 in C6D6

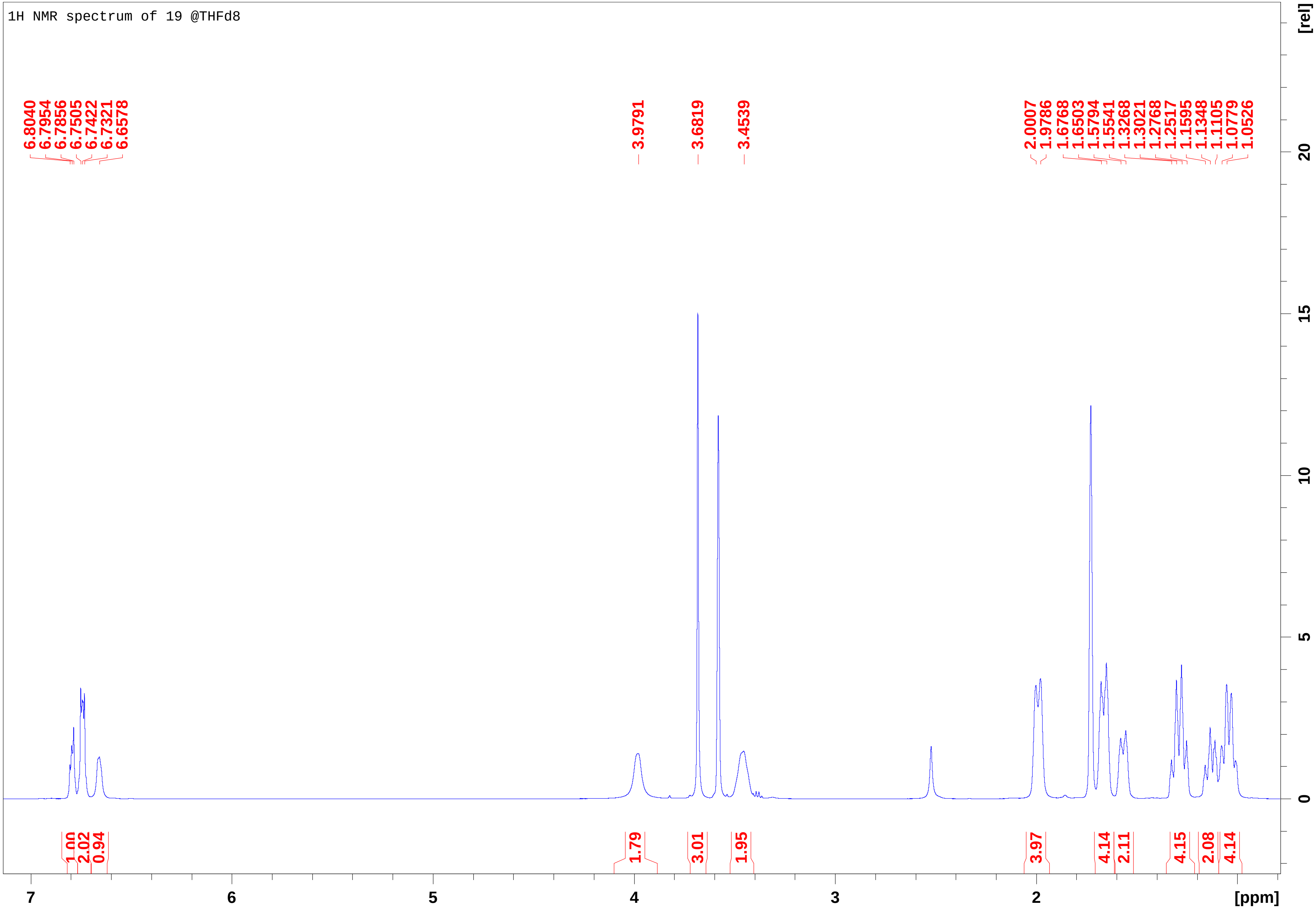

Figure S177. 1H NMR spectrum of 19 in THF-d8

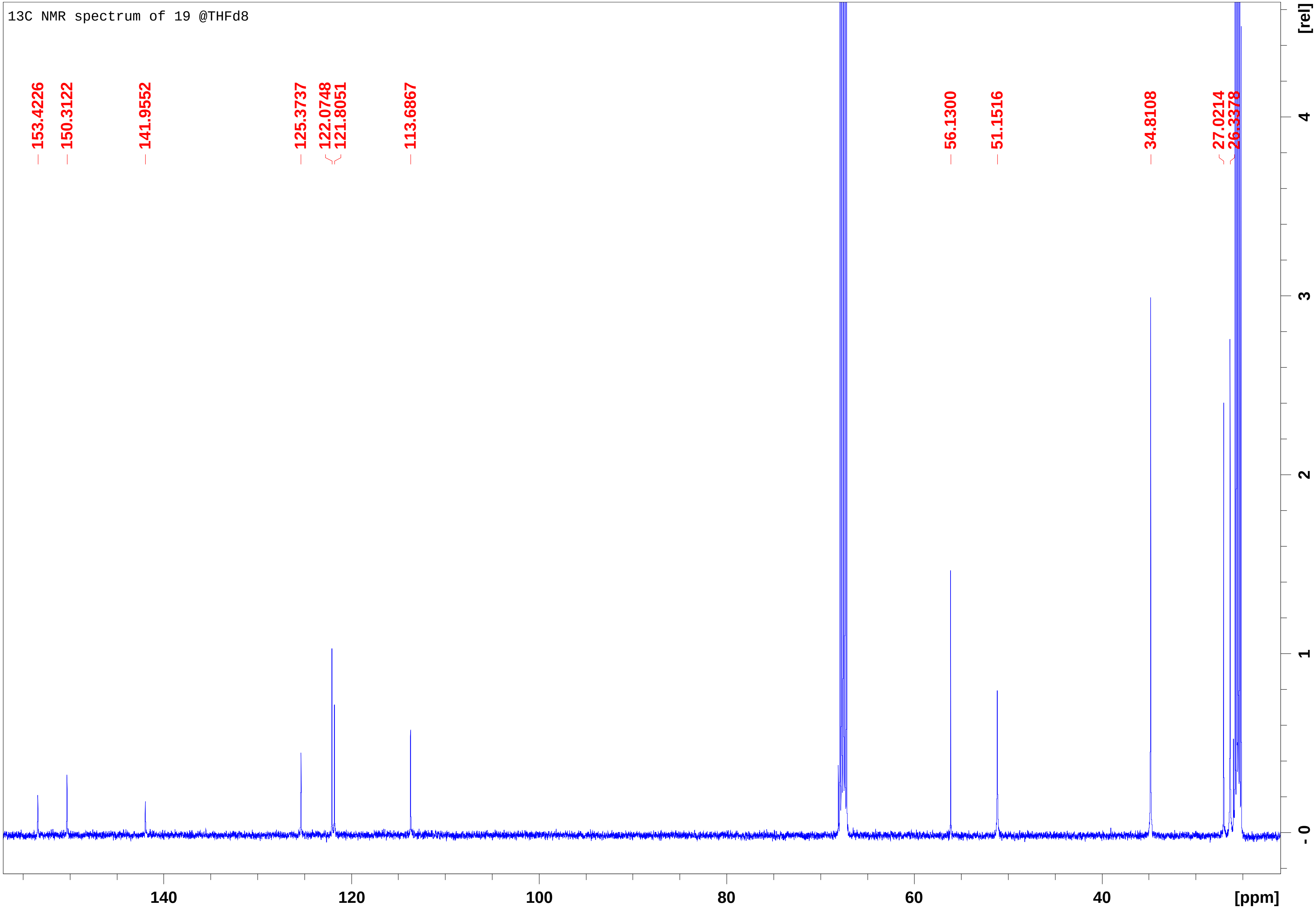

Figure S178. 13C NMR spectrum of 19 in THF-d8

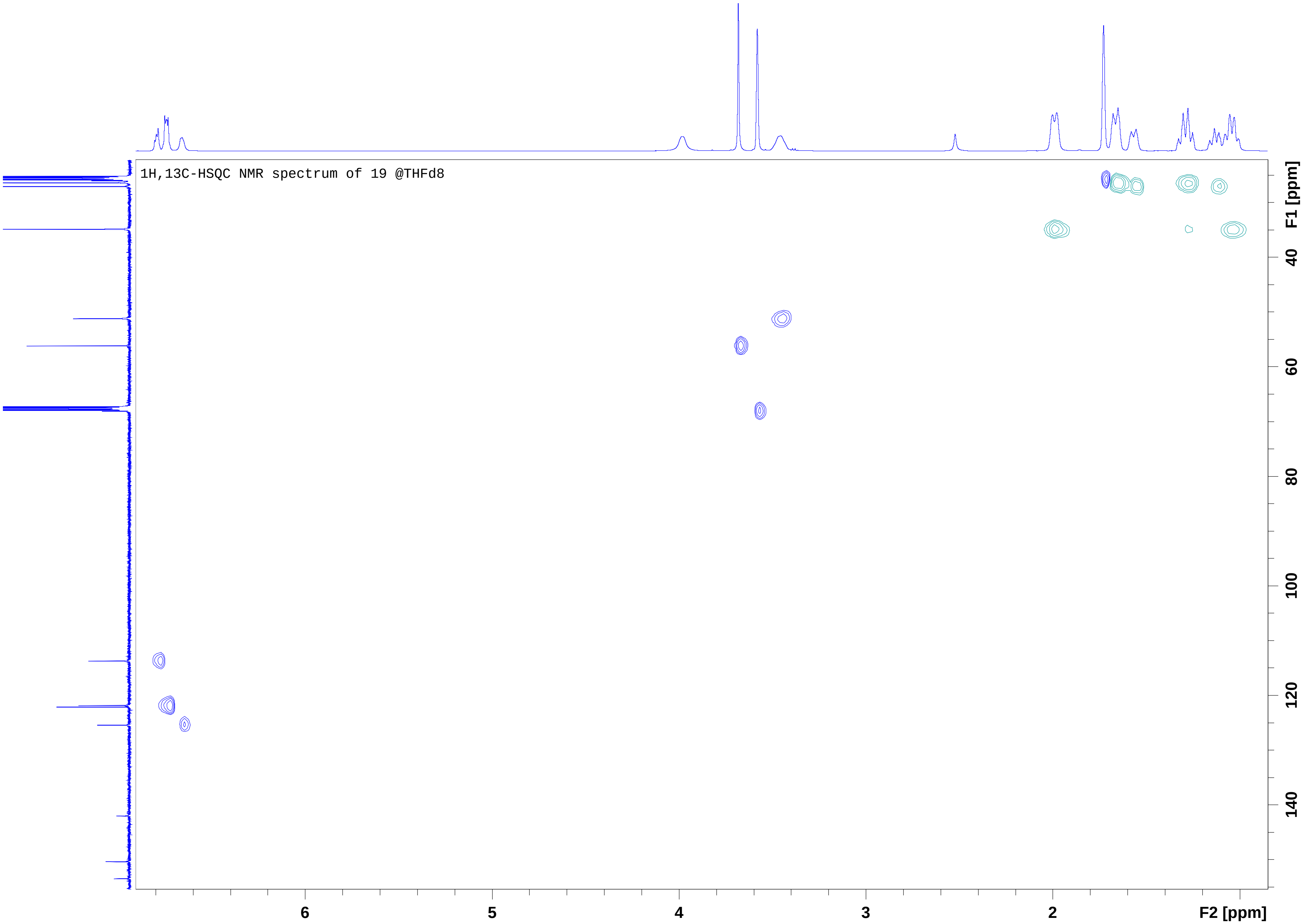

Figure S179. 1H,13C-HSQC NMR spectrum of 19 in THF-d8

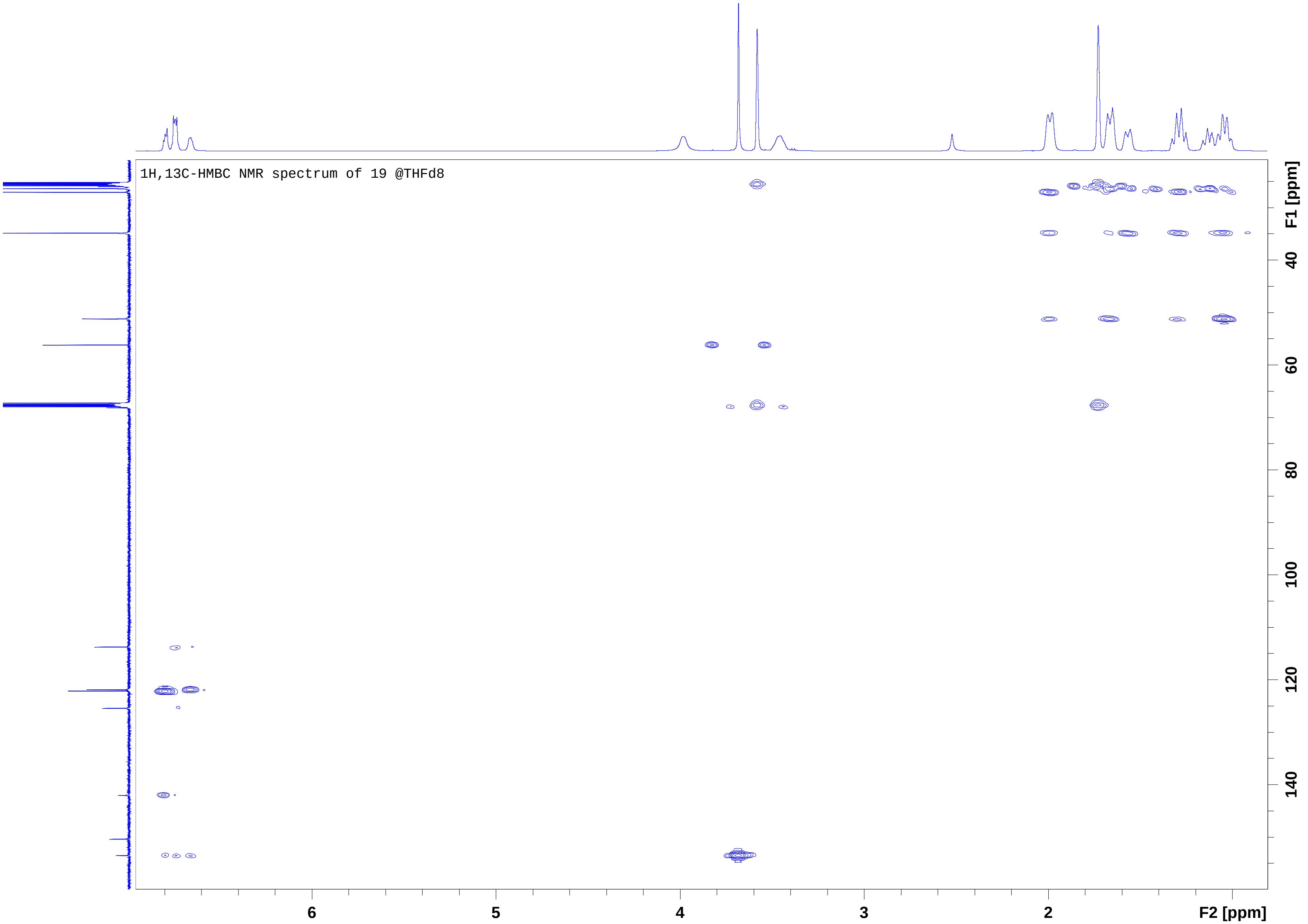

Figure S180. 1H,13C-HMBC NMR spectrum of 19 in THF-d8

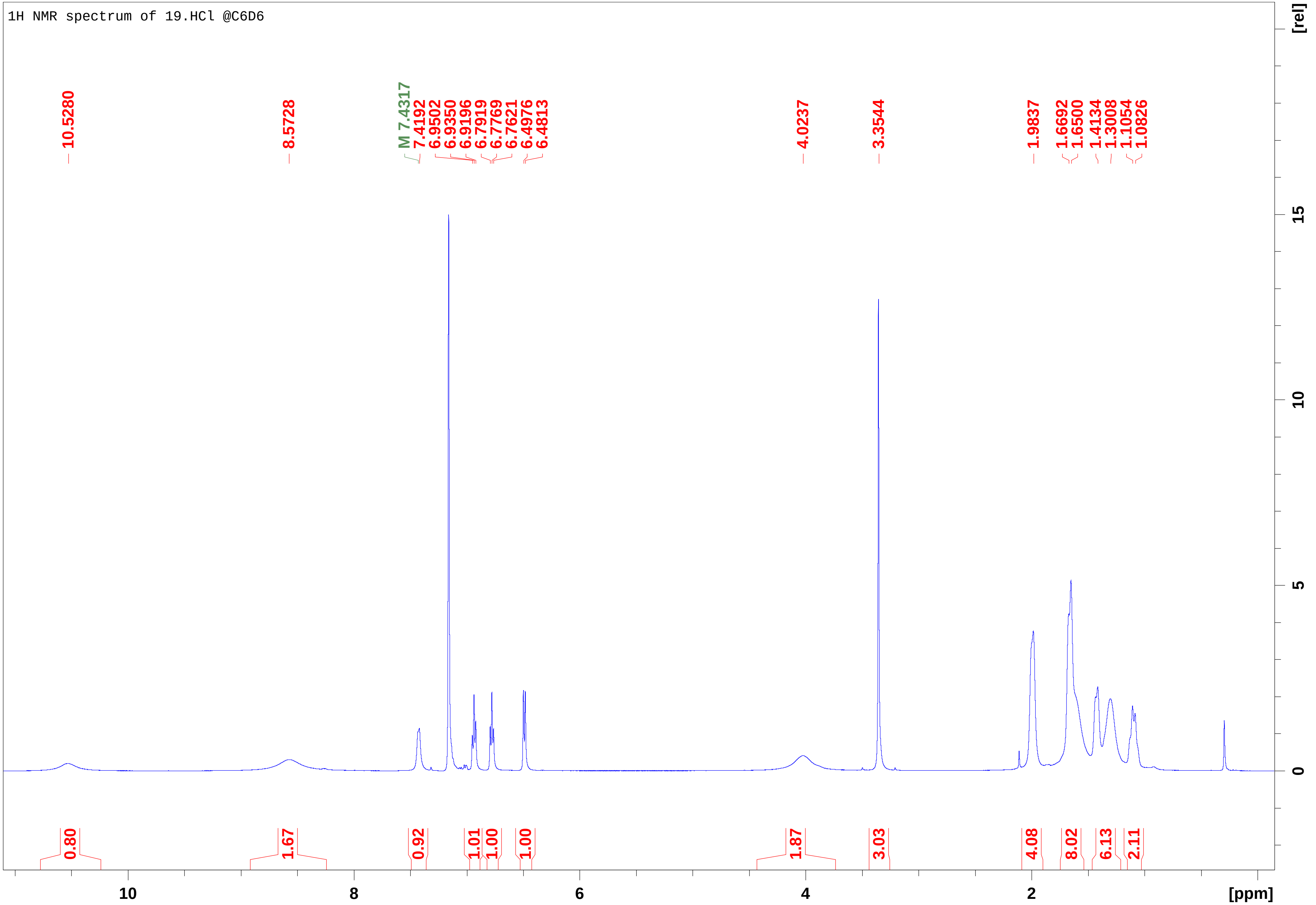

Figure S181. 1H NMR spectrum of 19.HCl in C6D6

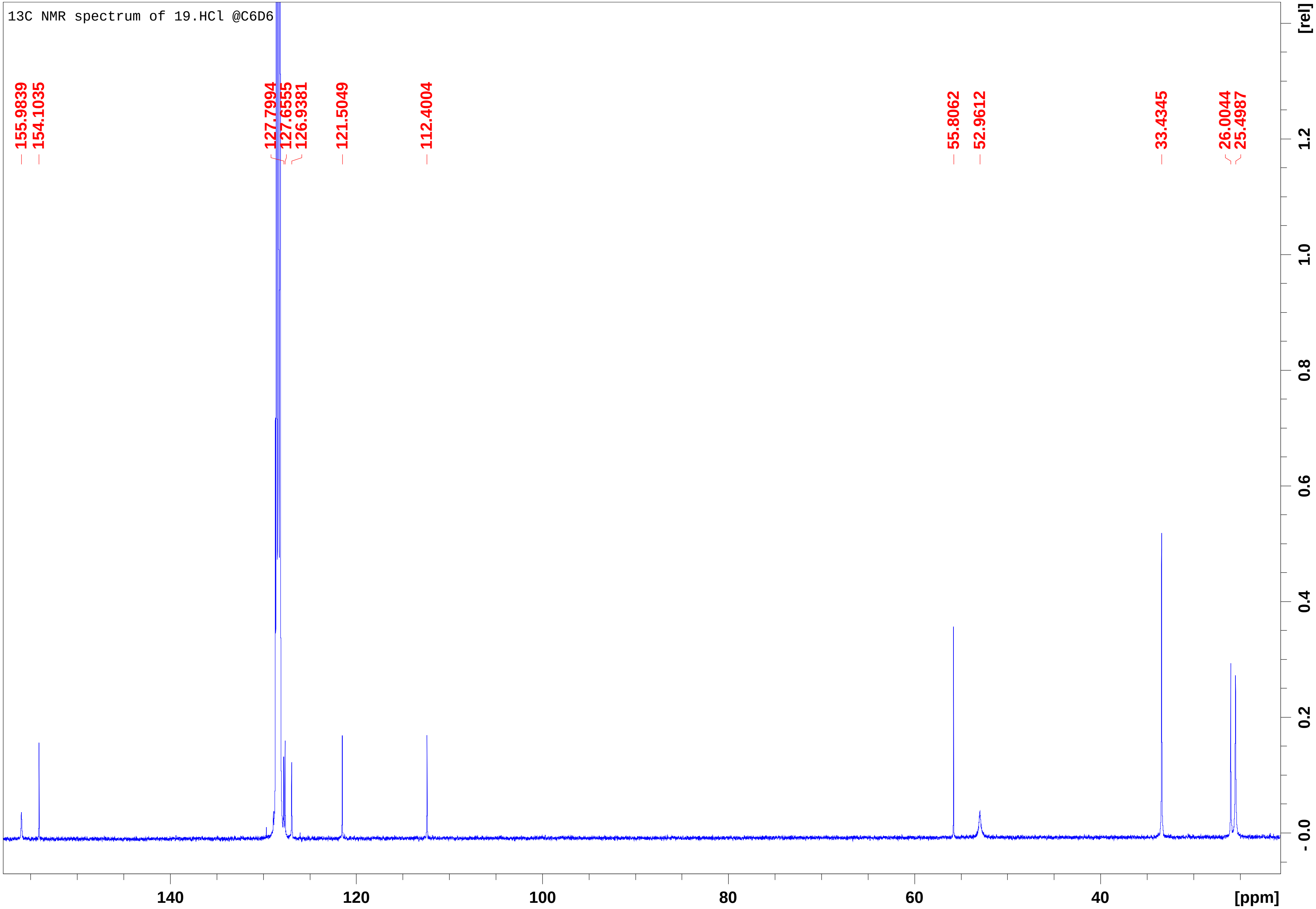

Figure S182. 13C NMR spectrum of 19.HCl in C6D6

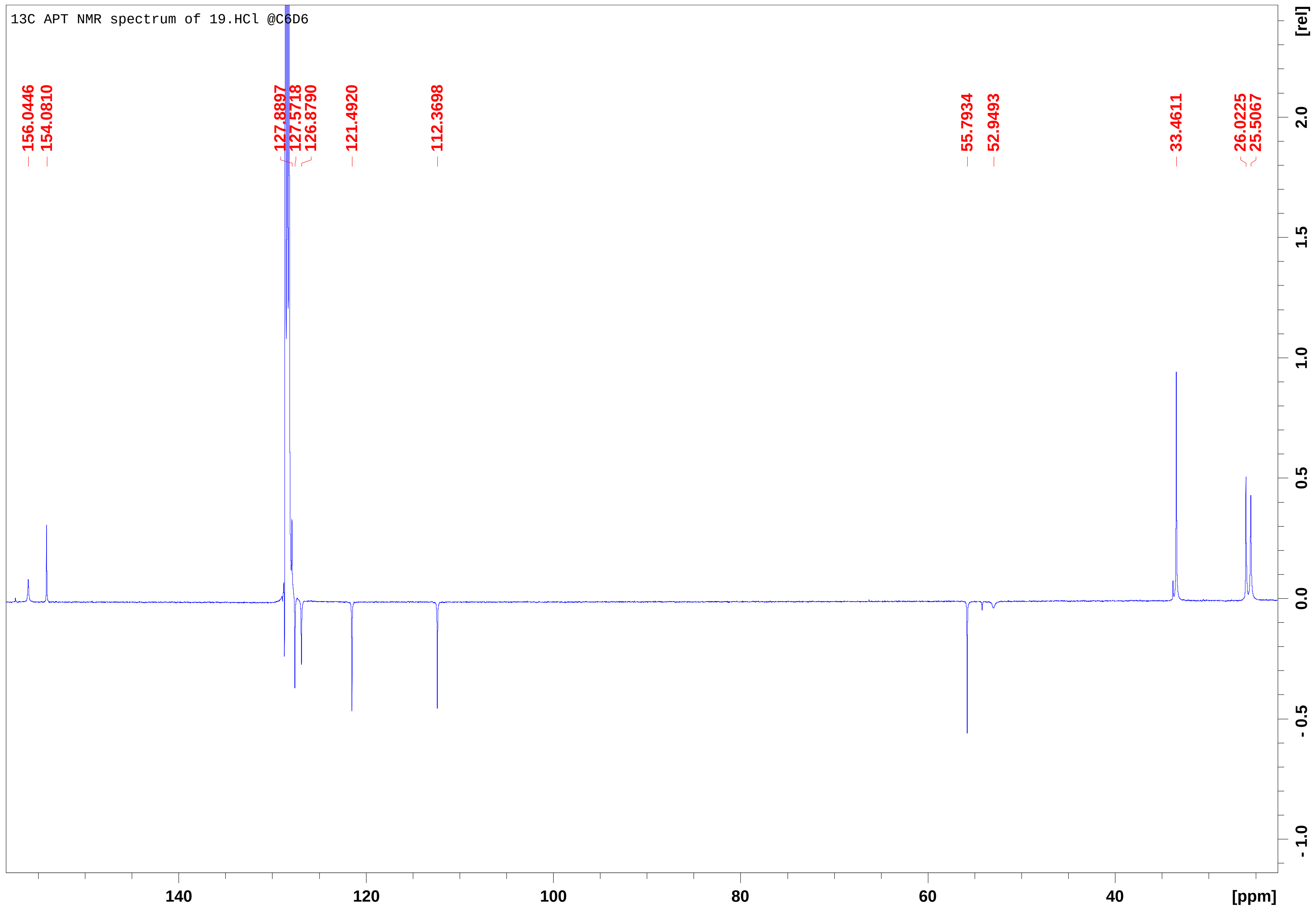

Figure S183. 13C APT NMR spectrum of 19.HCl in C6D6

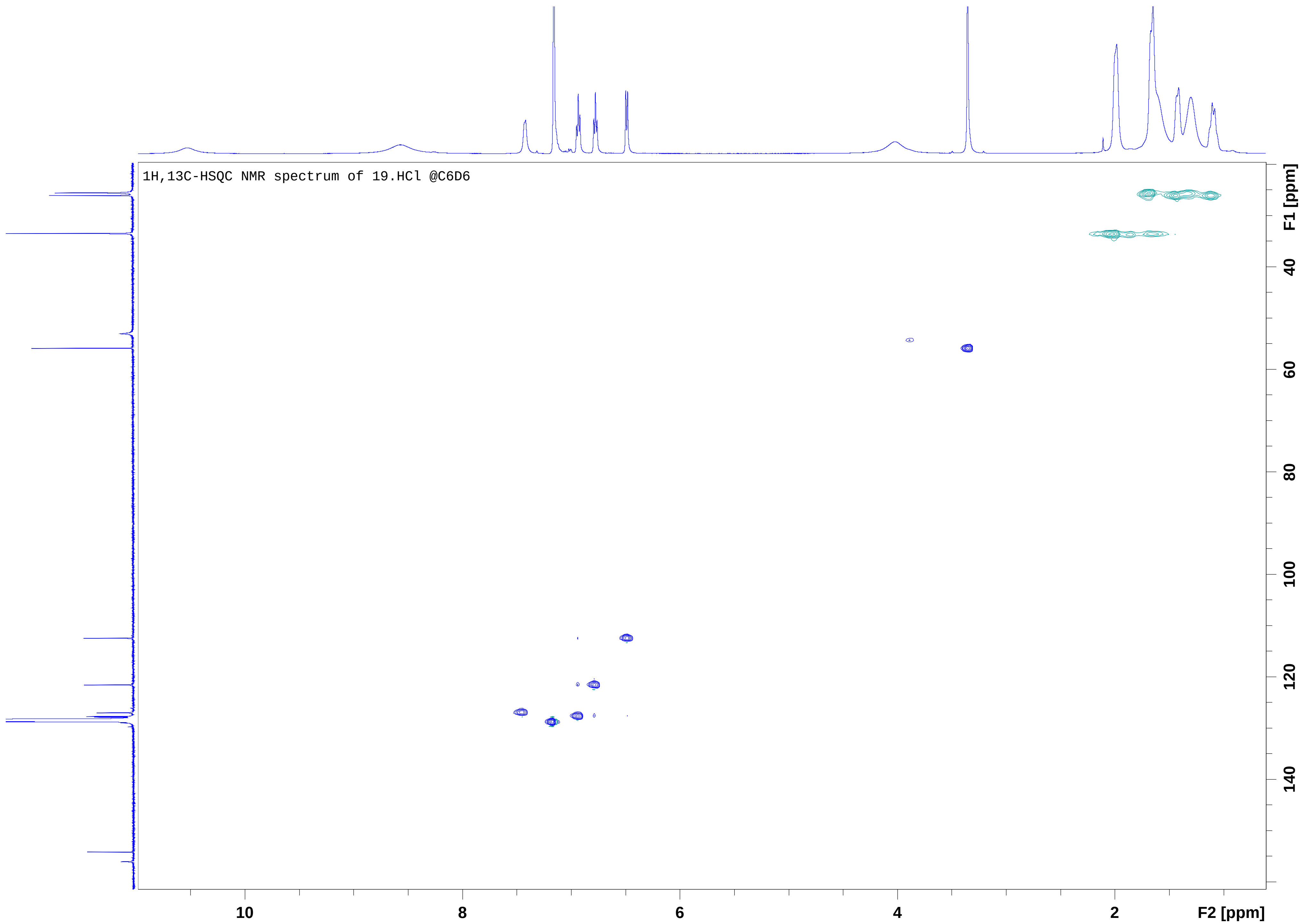

Figure S184. 1H,13C-HSQC NMR spectrum of 19.HCl in C6D6

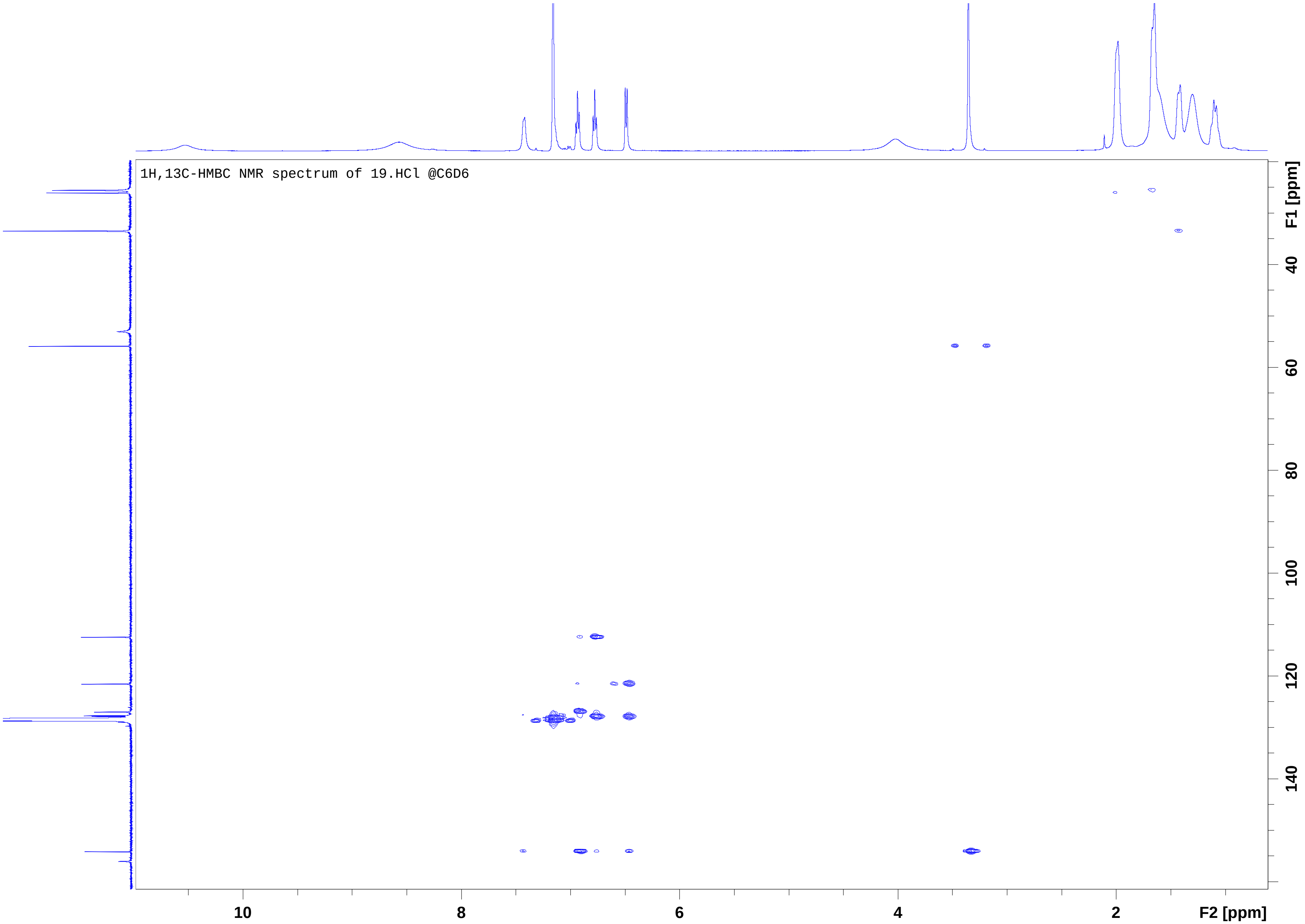

Figure S185. 1H,13C-HMBC NMR spectrum of 19.HCl in C6D6

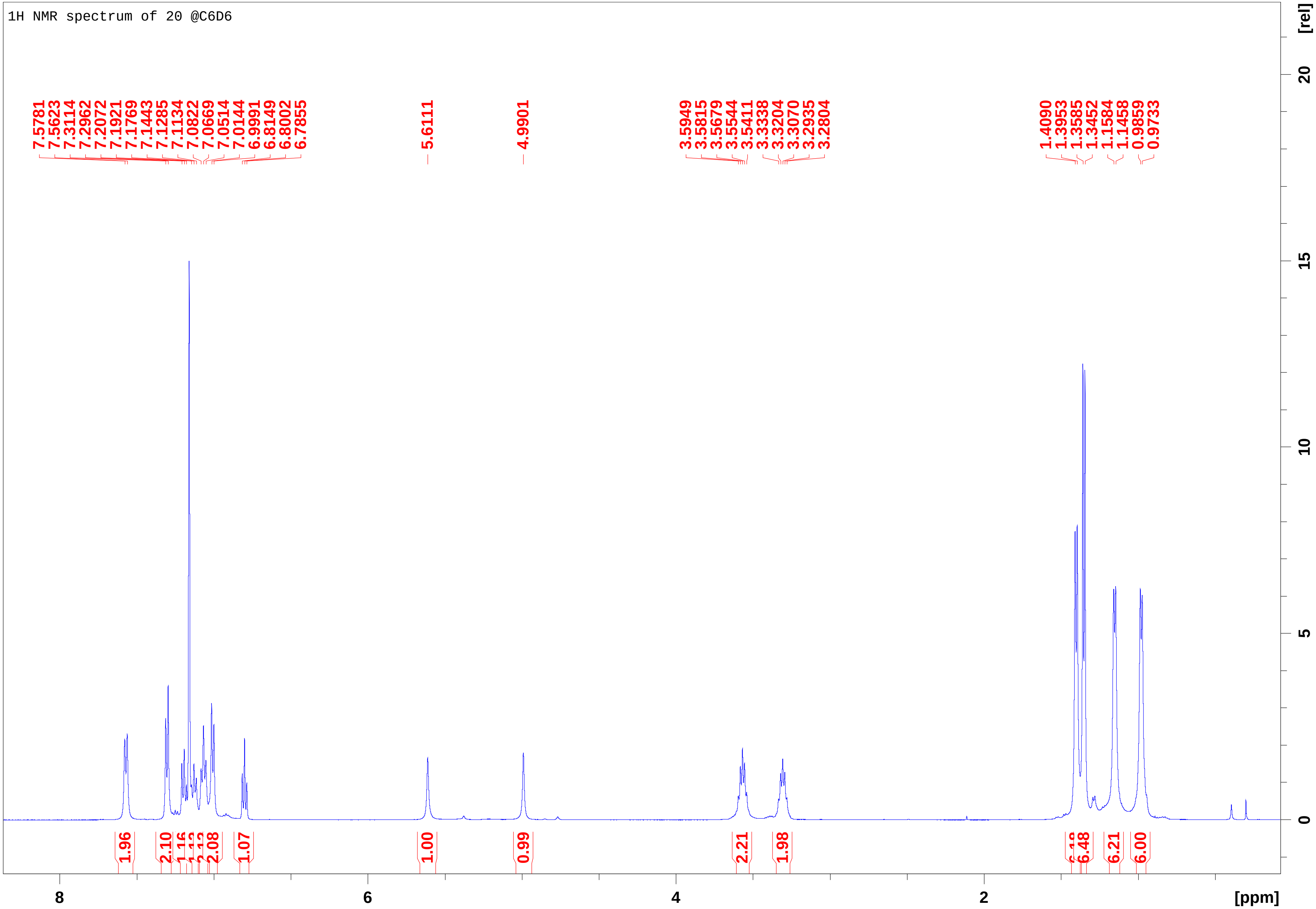

Figure S186. 1H NMR spectrum of 20 in C6D6

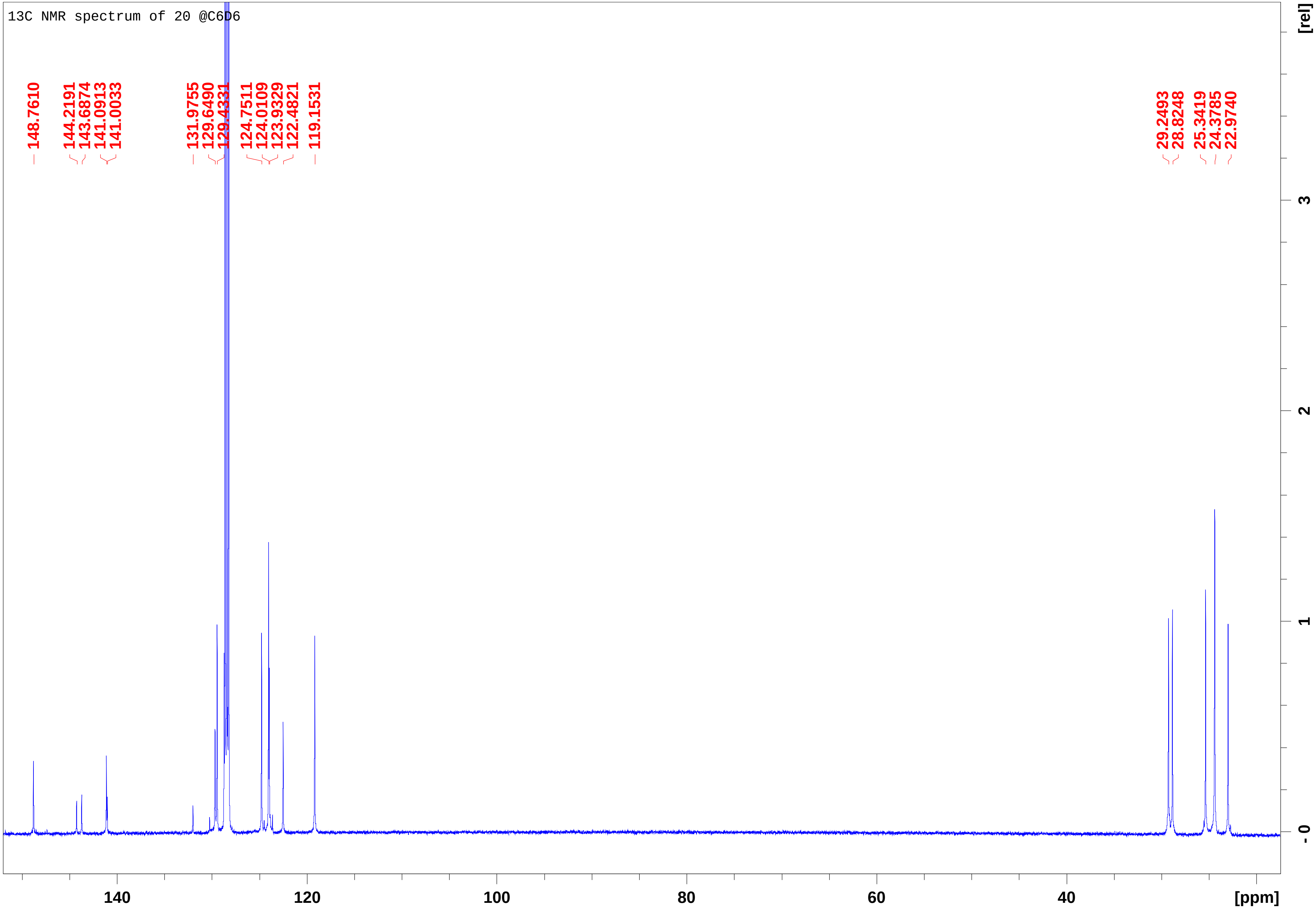

Figure S187. 13C NMR spectrum of 20 in C6D6

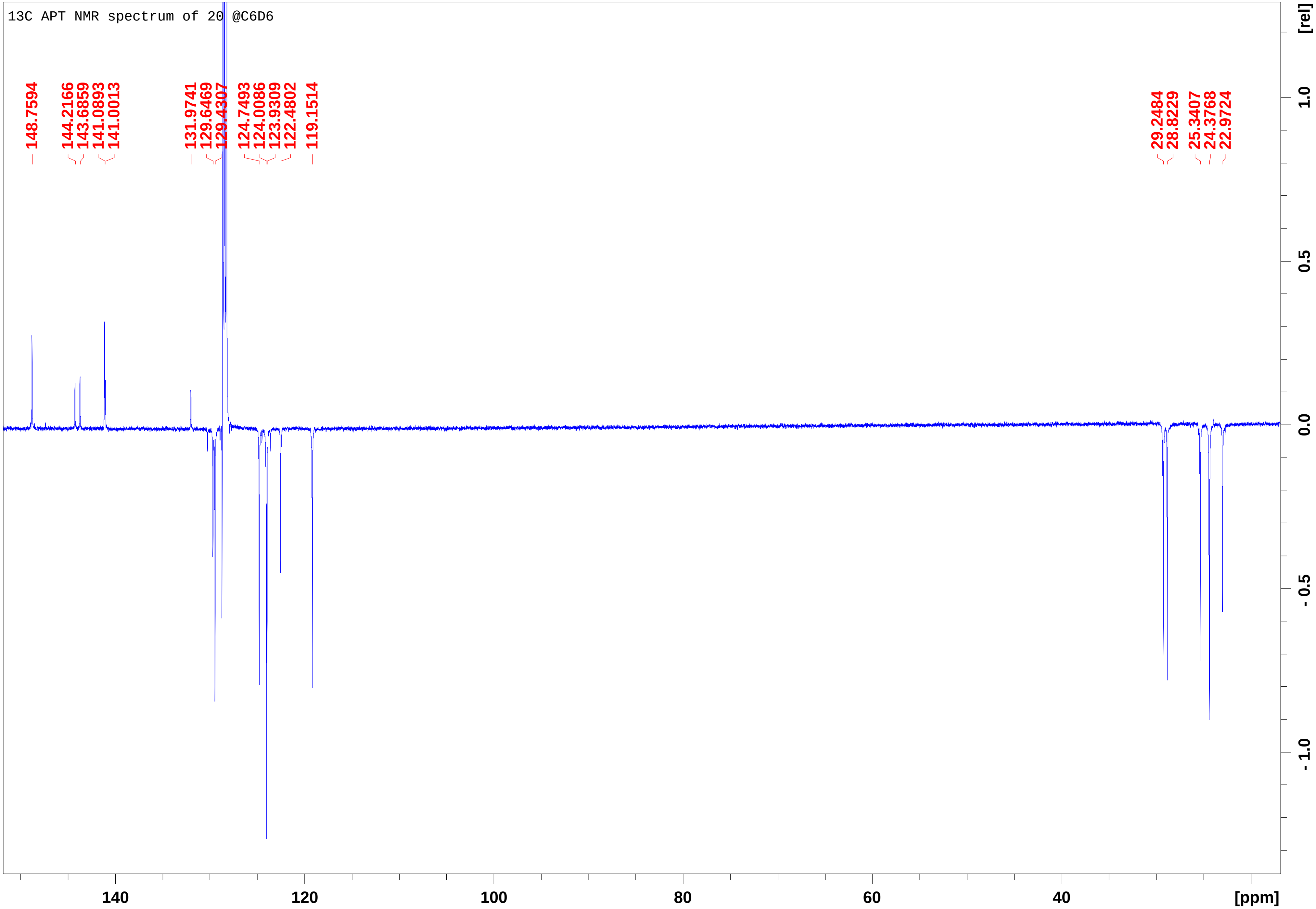

Figure S188. 13C APT NMR spectrum of 20 in C6D6

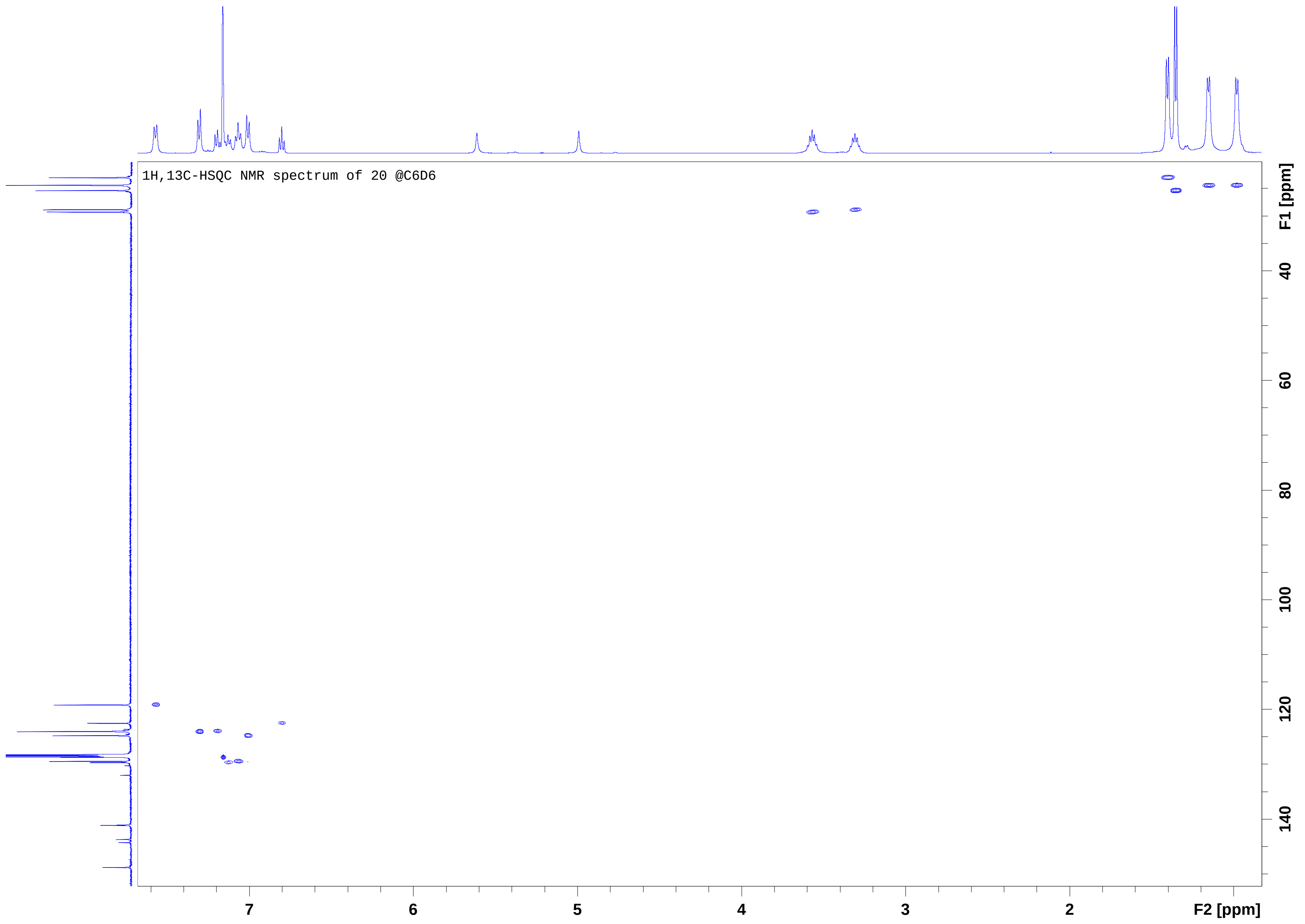

Figure S189. 1H,13C-HSQC NMR spectrum of 20 in C6D6

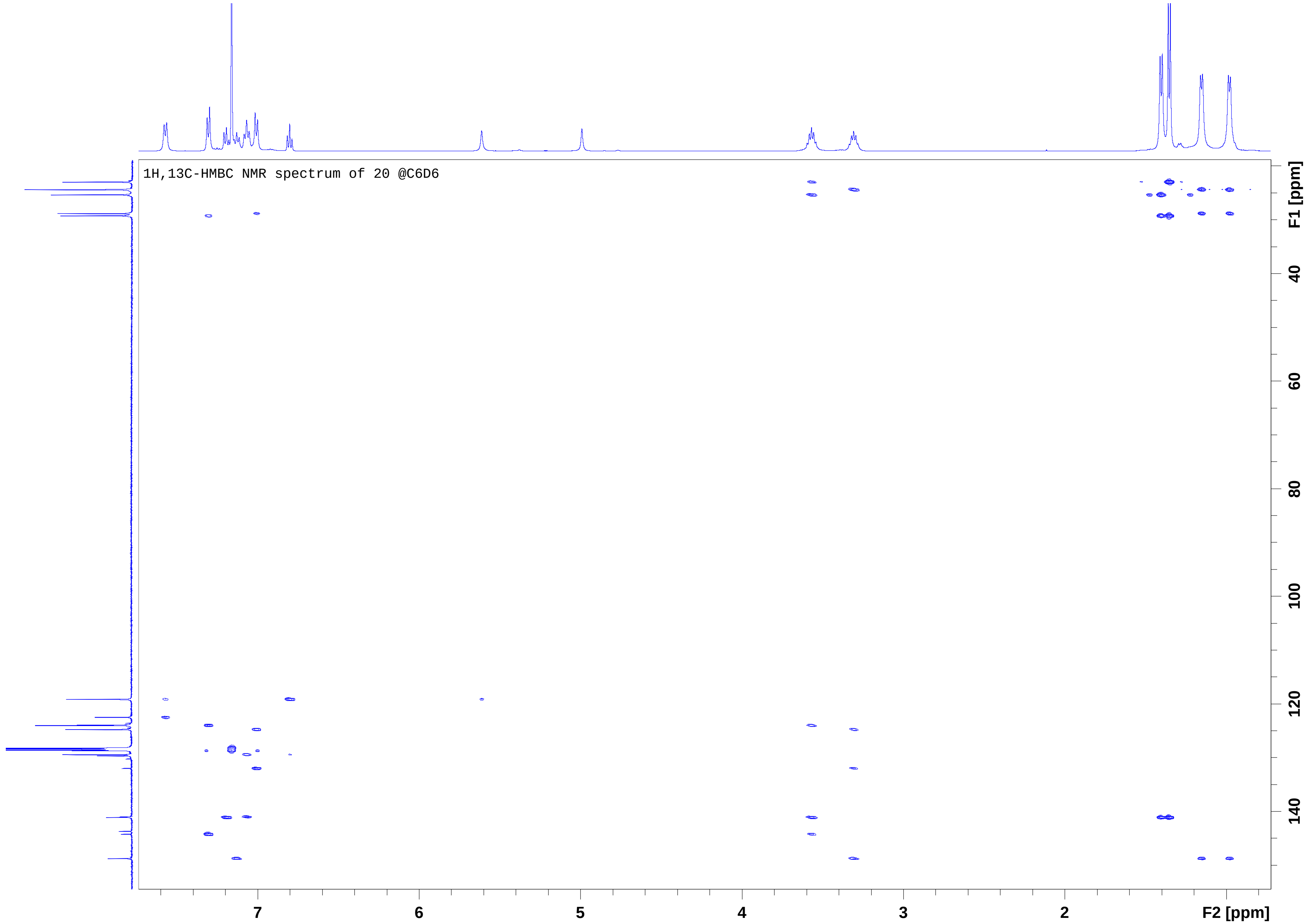

Figure S190. 1H,13C-HSQC NMR spectrum of 20 in C6D6

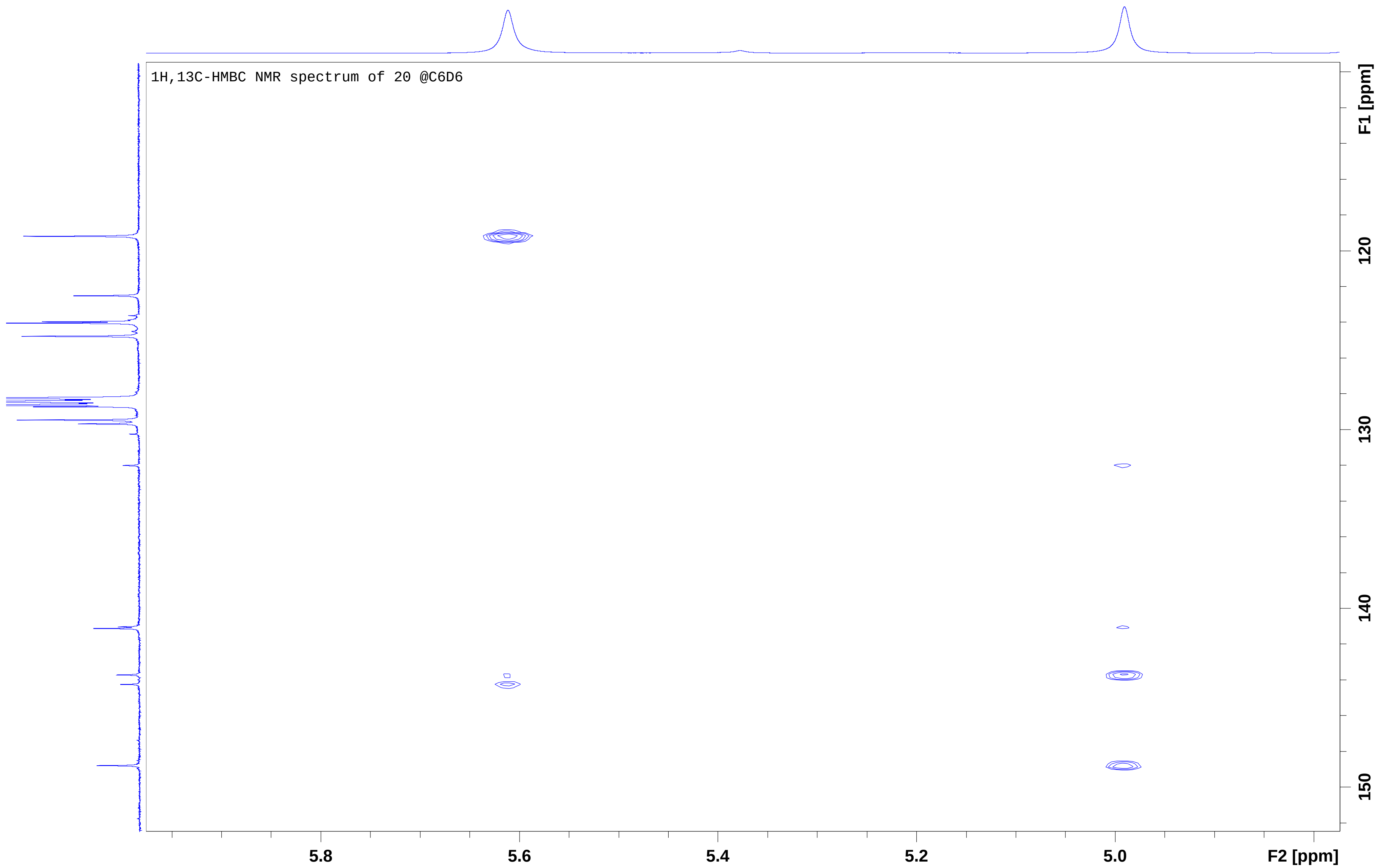

Figure S191. Detail of 1H,13C-HMBC NMR spectrum of 20 in C6D6

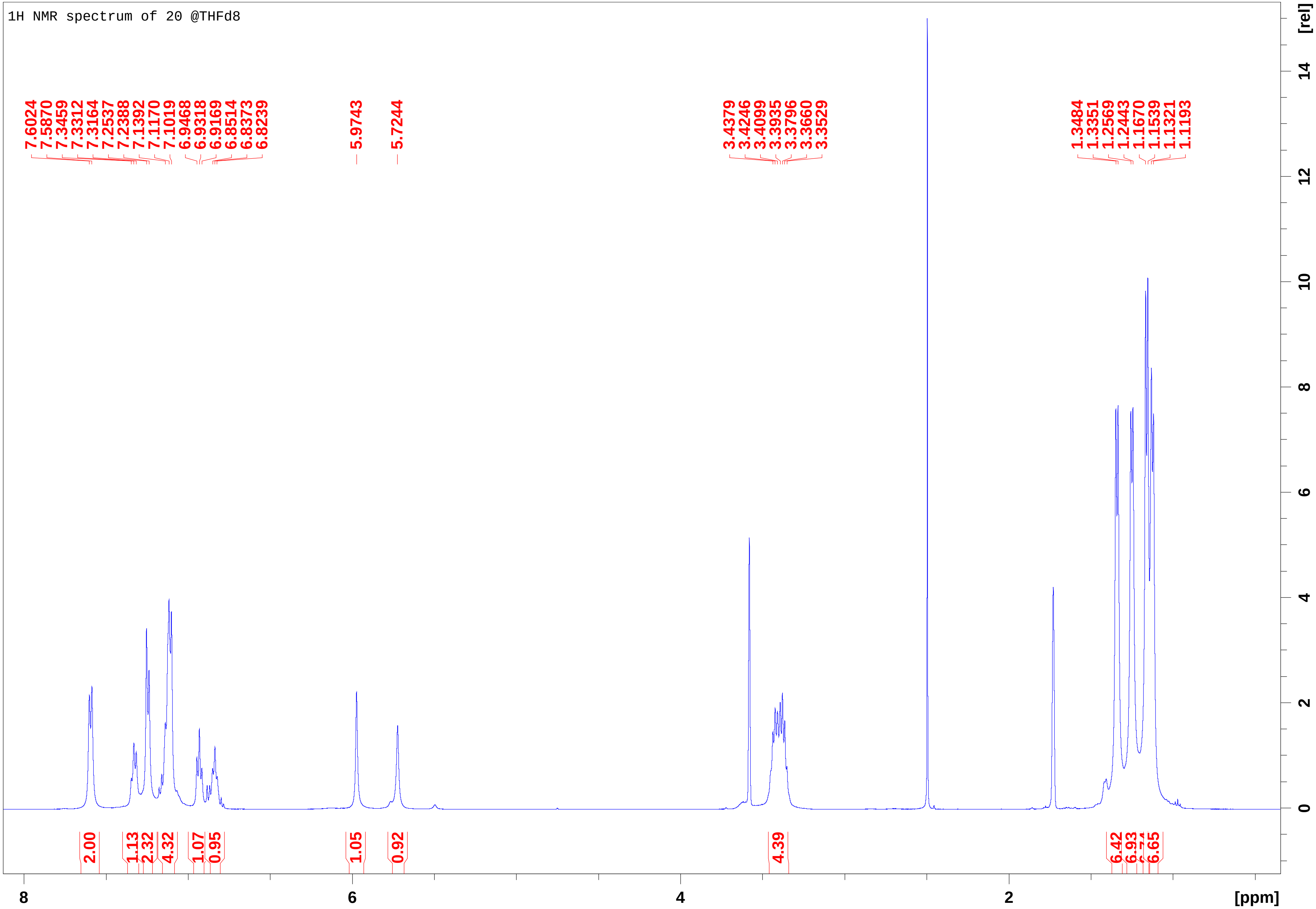

Figure S192. 1H NMR spectrum of 20 in THF-d8

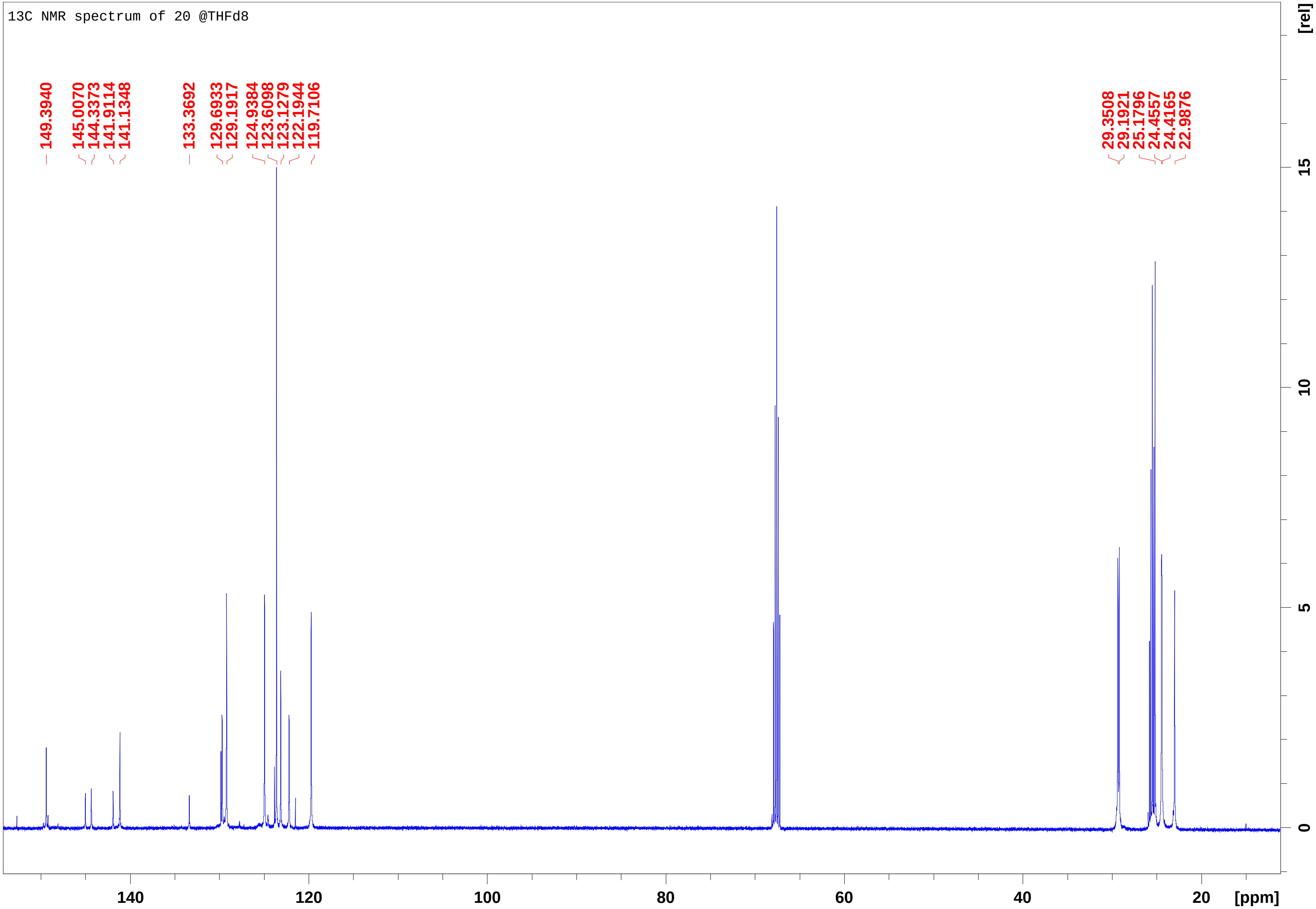

Figure S193. 13C NMR spectrum of 20 in THF-d8

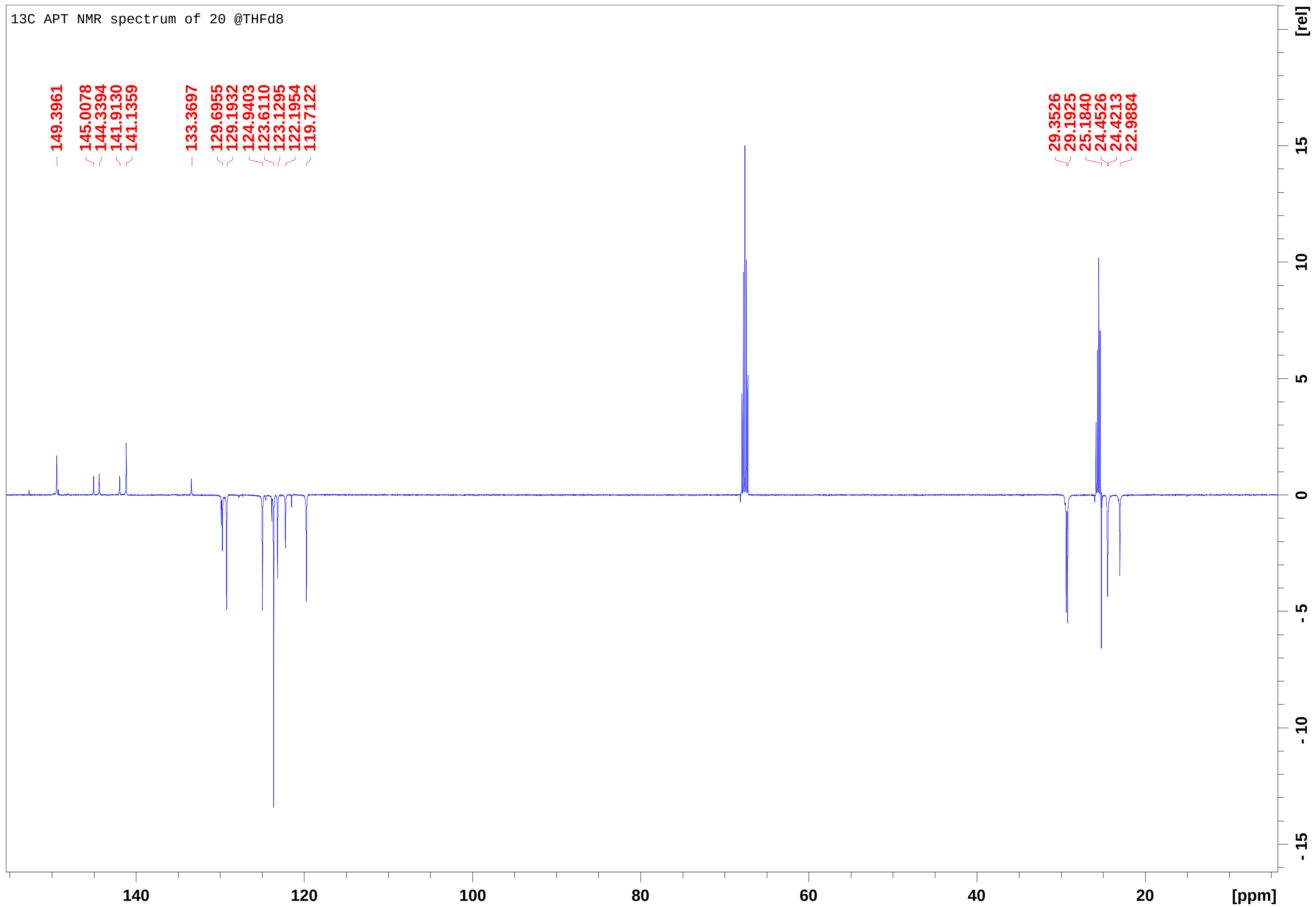

Figure S194. 13C APT NMR spectrum of 20 in THF-d8

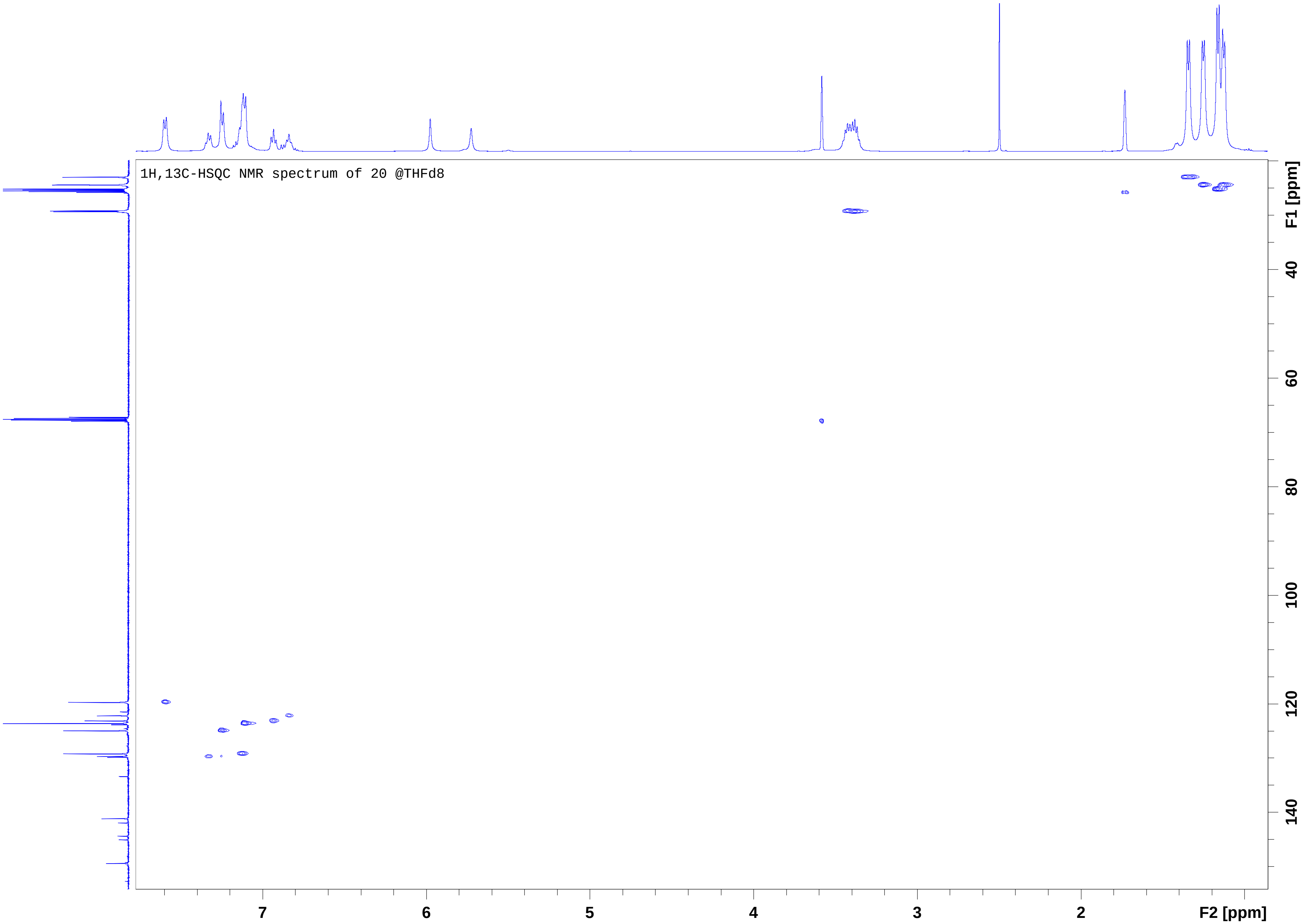

Figure S195. 1H,13C-HSQC NMR spectrum of 20 in THF-d8

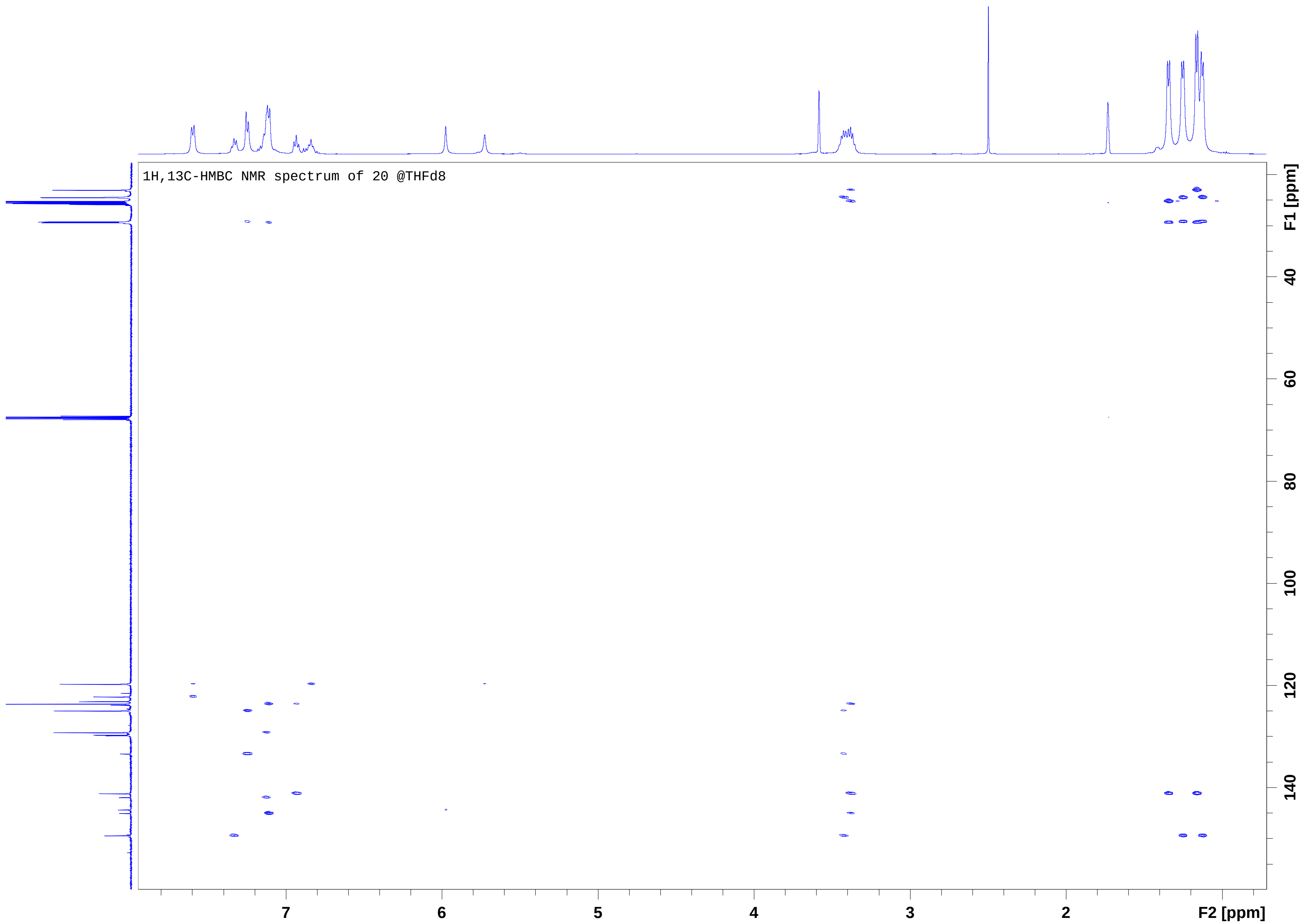

Figure S196. 1H,13C-HMBC NMR spectrum of 20 in THF-d8

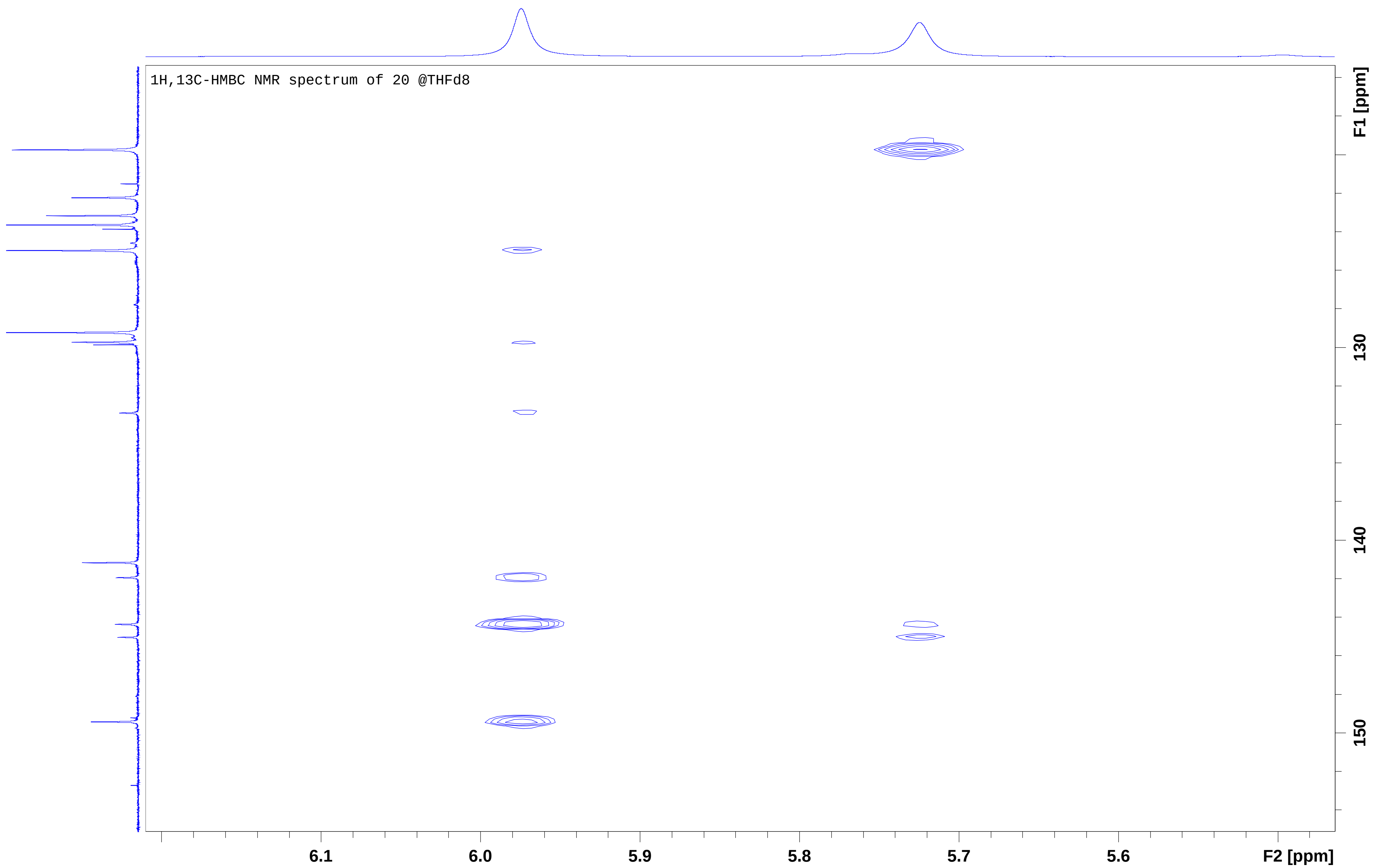

Figure S197. Detail of 1H,13C-HMBC NMR spectrum of 20 in THF-d8

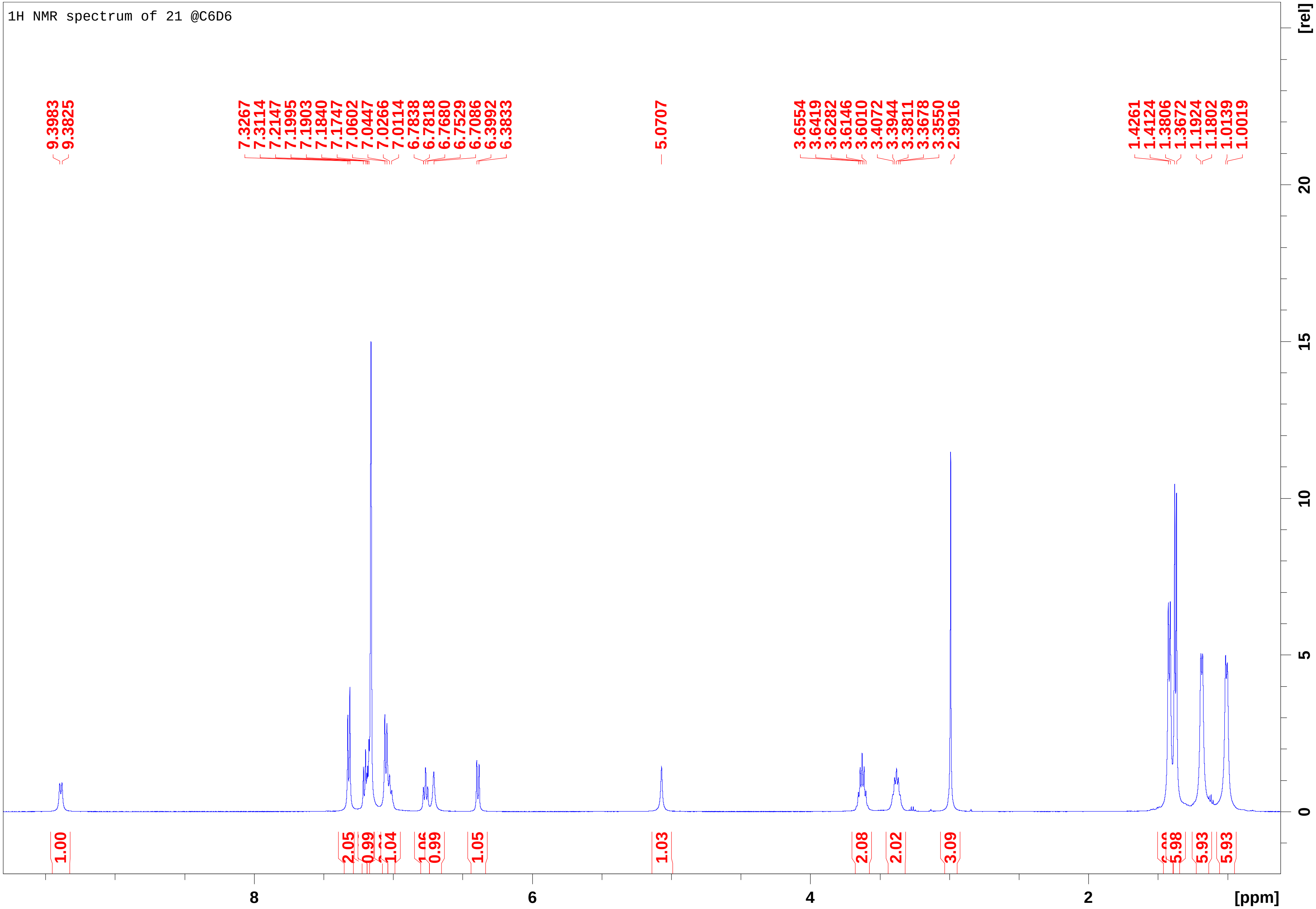

Figure S198. 1H NMR spectrum of 21 in C6D6

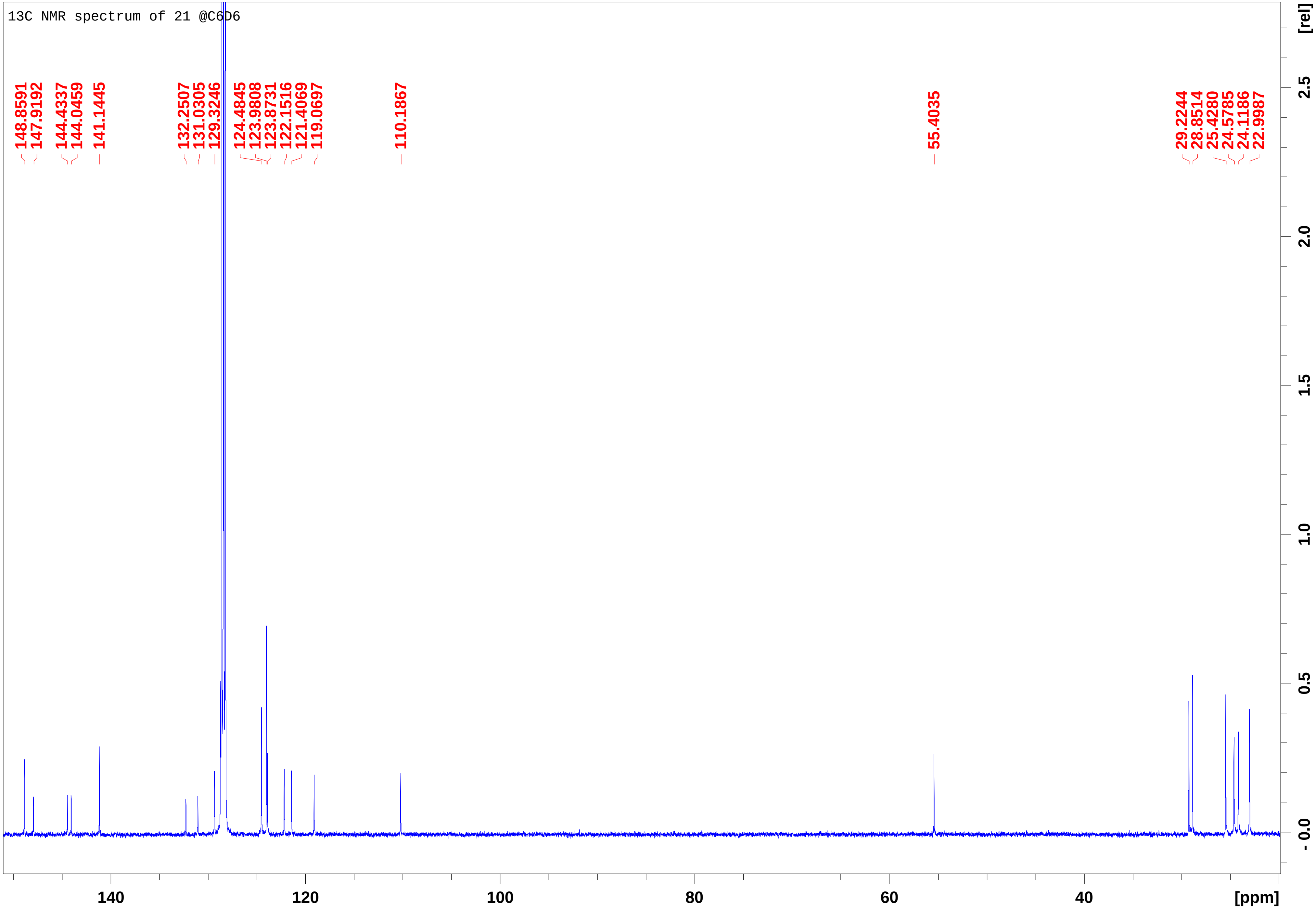

Figure S199. 13C NMR spectrum of 21 in C6D6

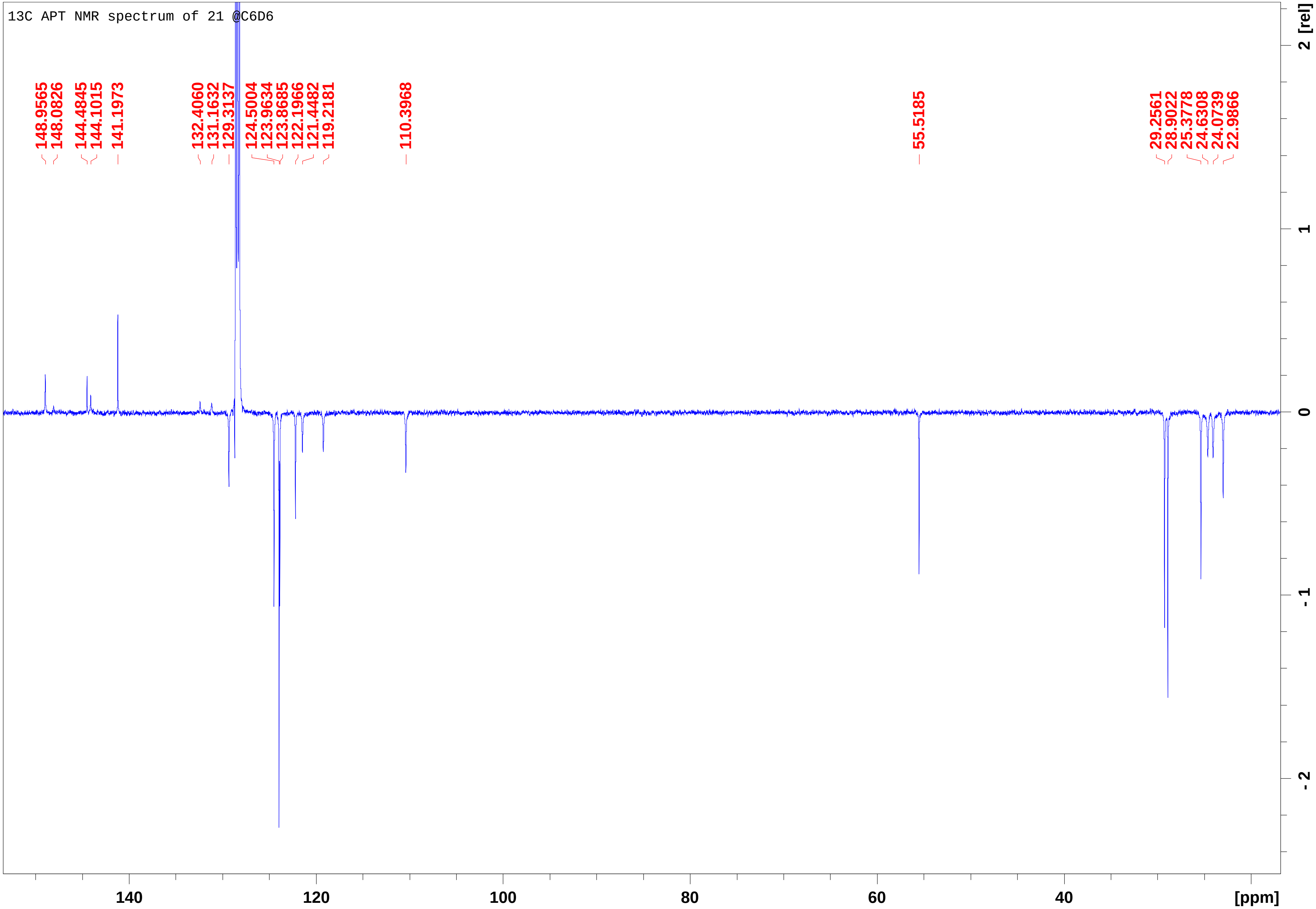

Figure S200. 13C APT NMR spectrum of 21 in C6D6

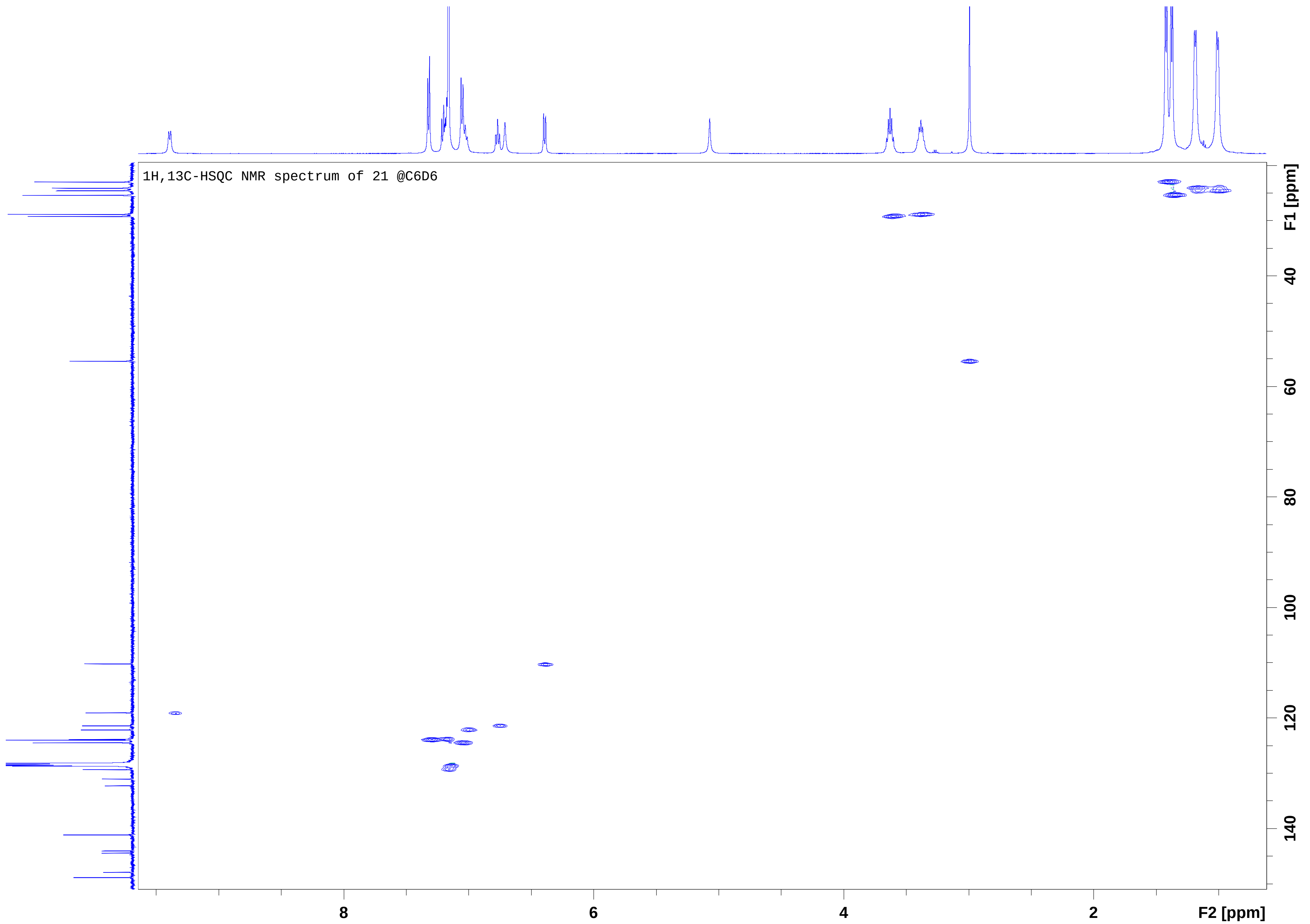

Figure S201. 1H,13C-HSQC NMR spectrum of 21 in C6D6

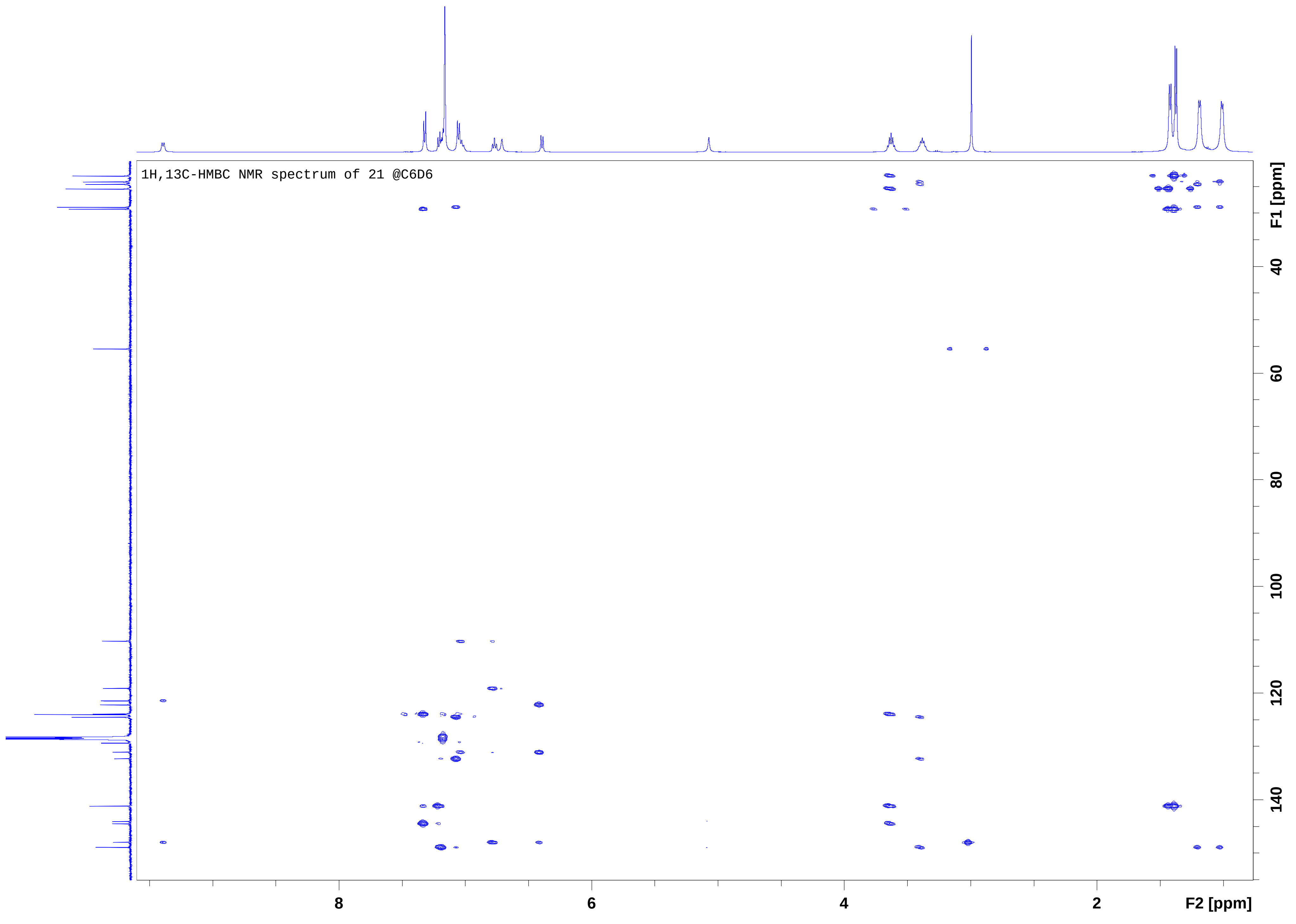

Figure S202. 1H,13C-HMBC NMR spectrum of 21 in C6D6

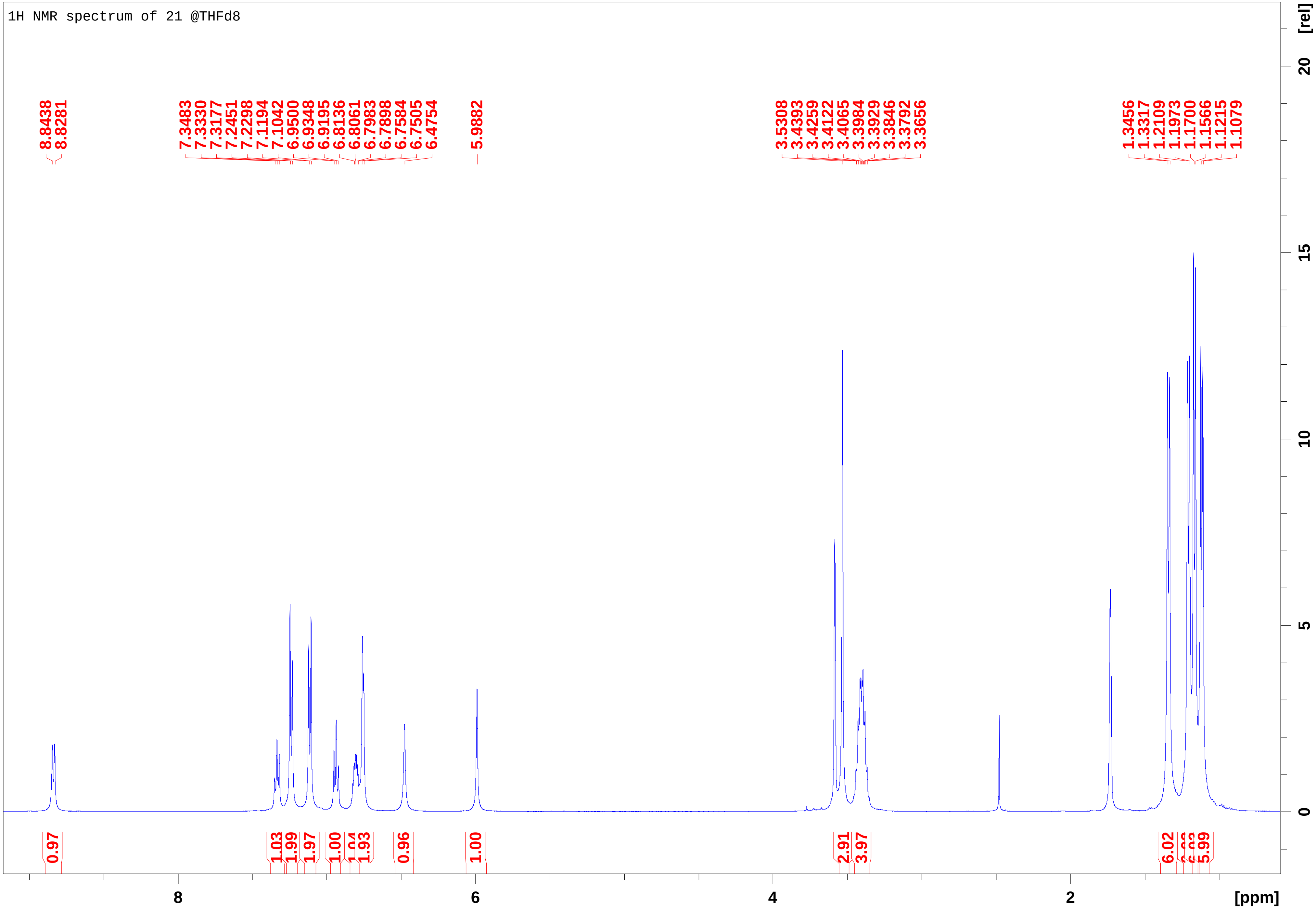

Figure S203. 1H NMR spectrum of 21 in THF-d8

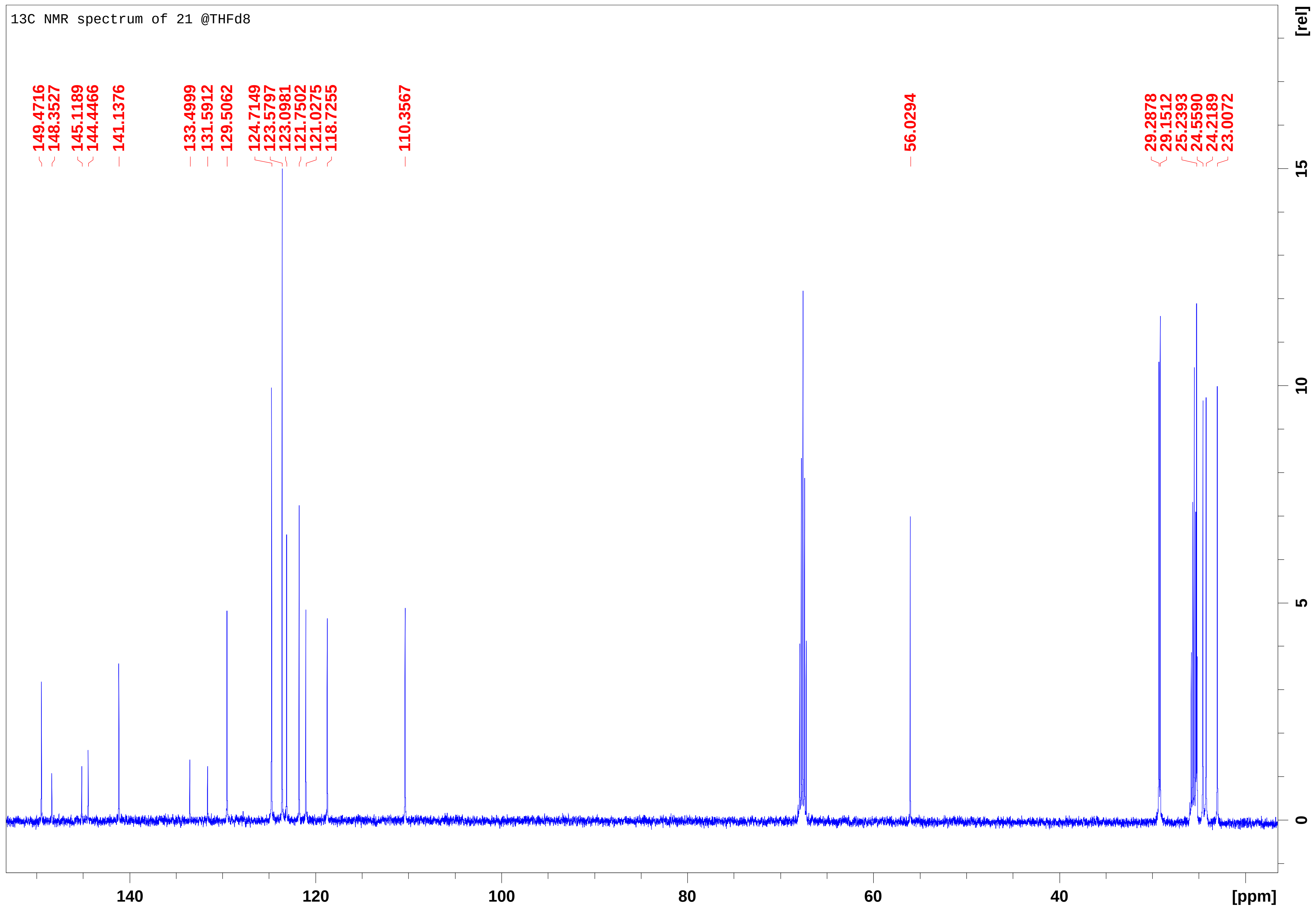

Figure S204. 13C NMR spectrum of 21 in THF-d8

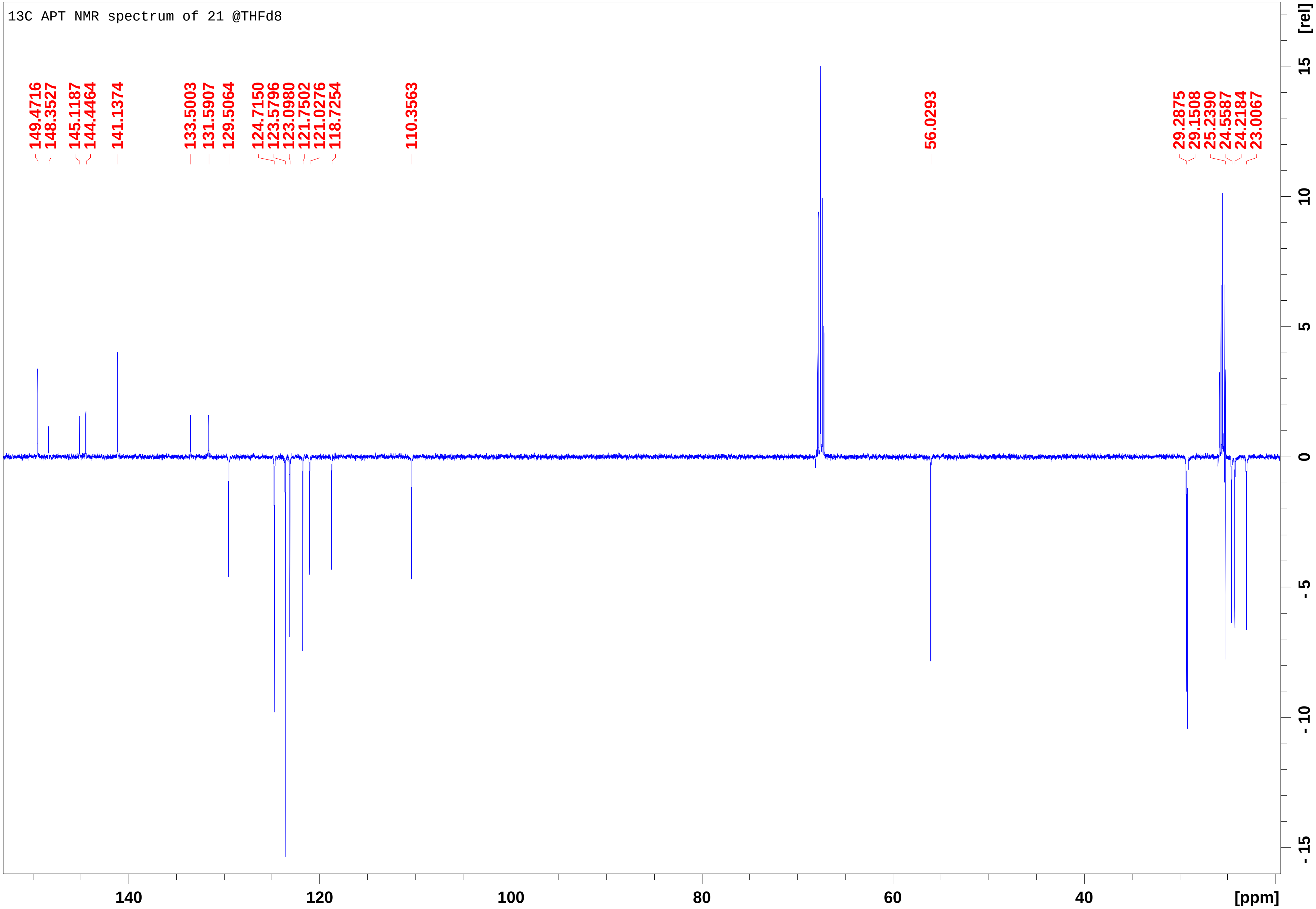

Figure S205. 13C APT NMR spectrum of 21 in THF-d8

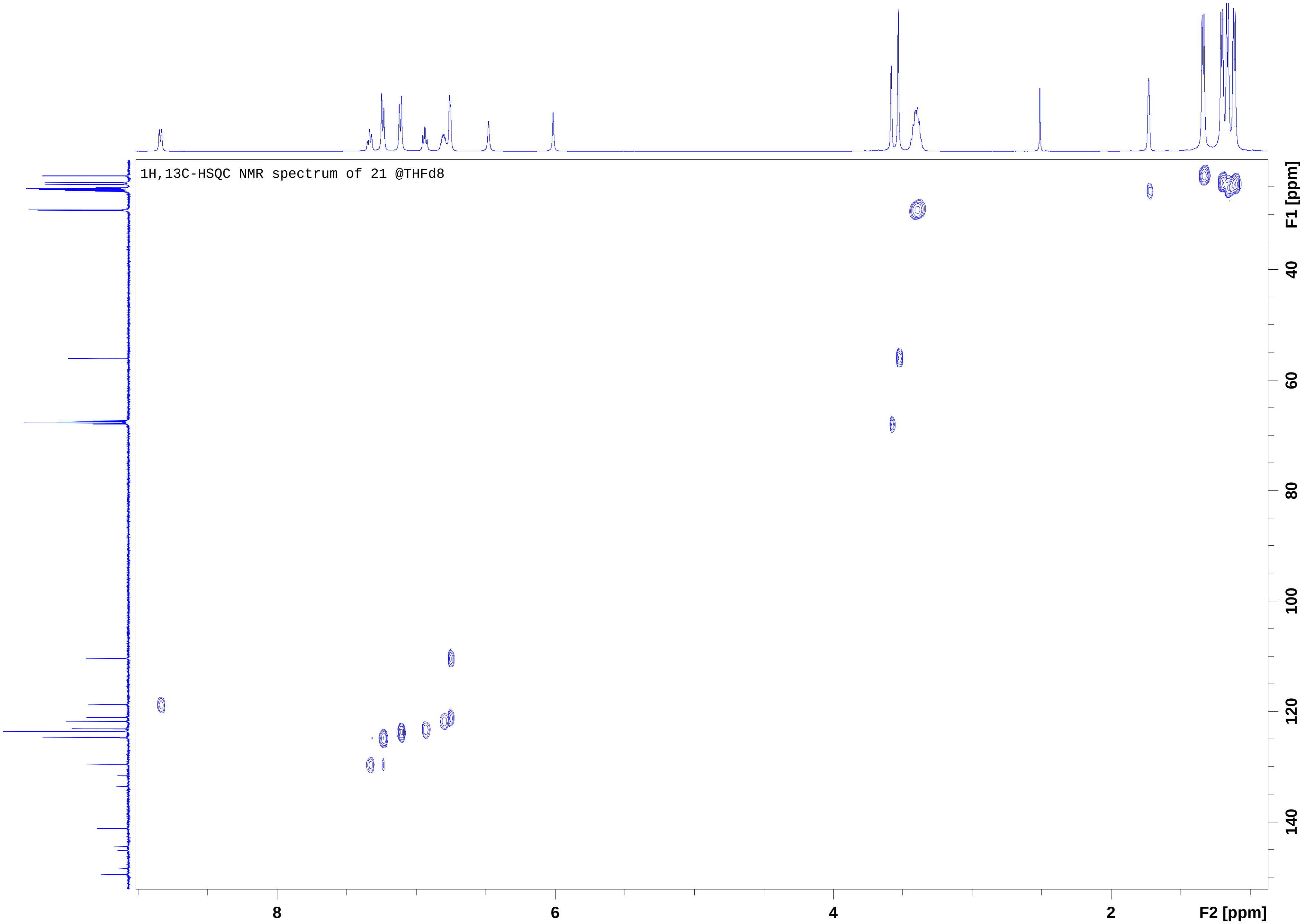

Figure S206. 1H,13C-HSQC NMR spectrum of 21 in THF-d8

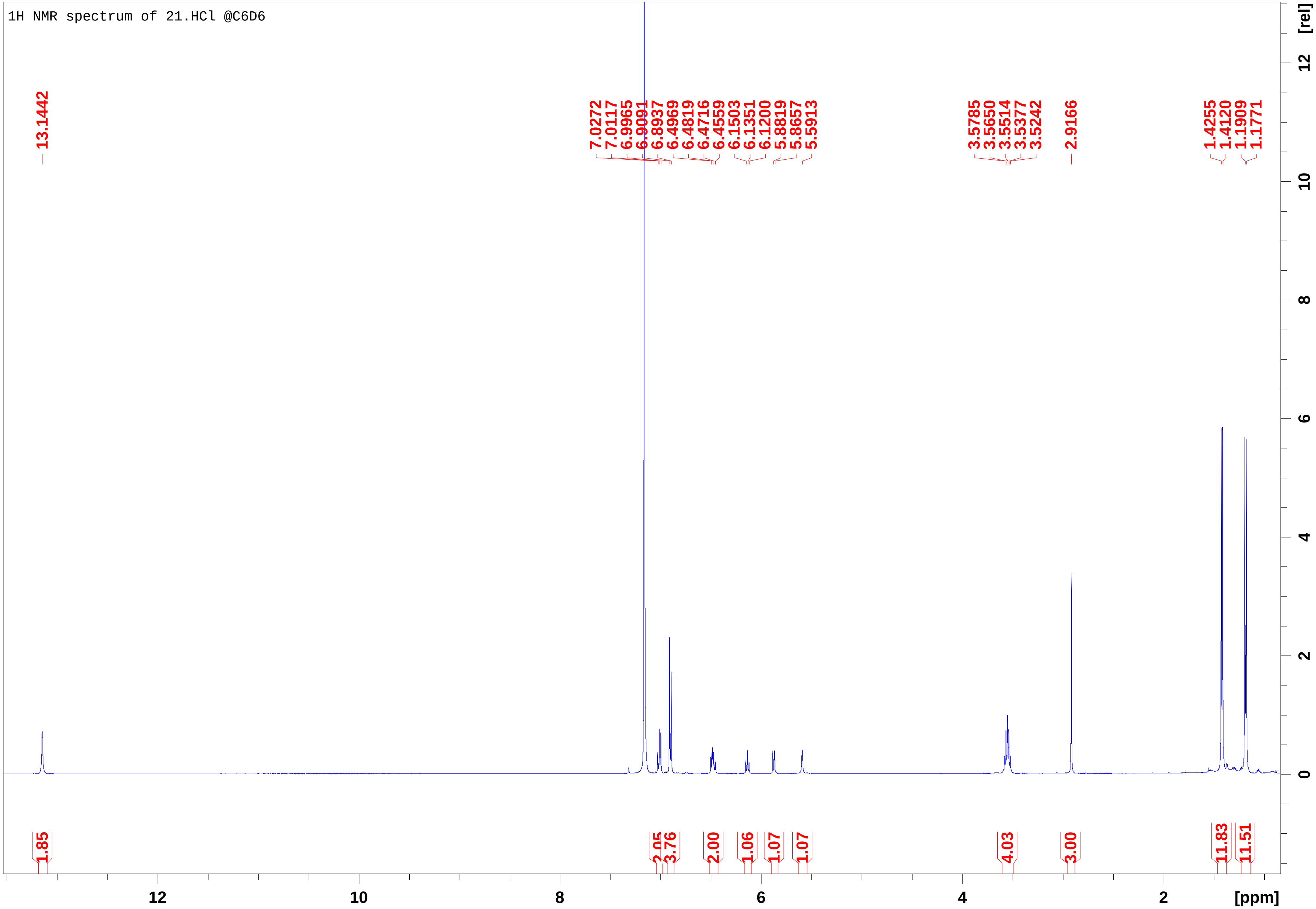

Figure S207. 1H NMR spectrum of 21.HCl in C6D6

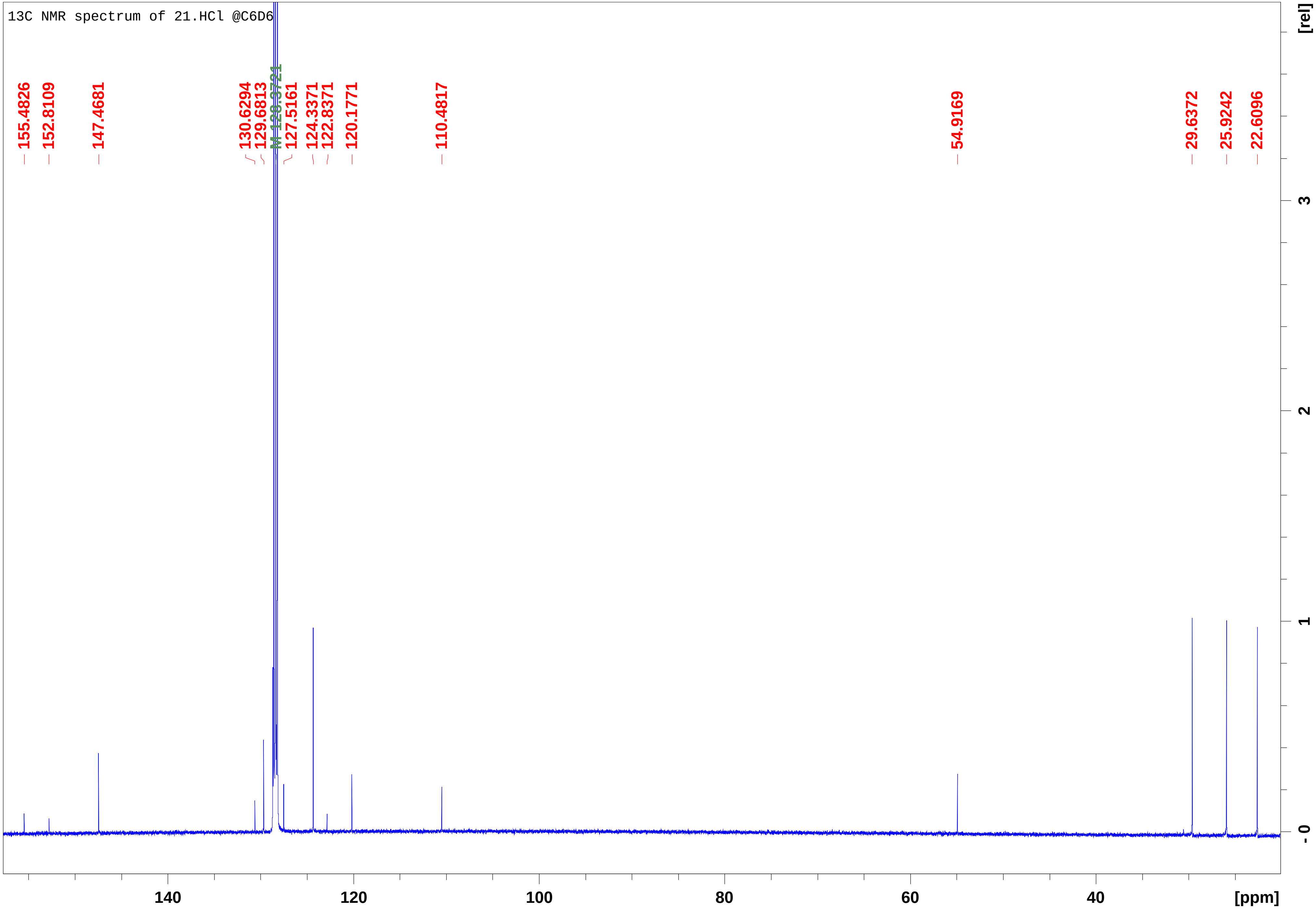

Figure S208. 13C NMR spectrum of 21.HCl in C6D6

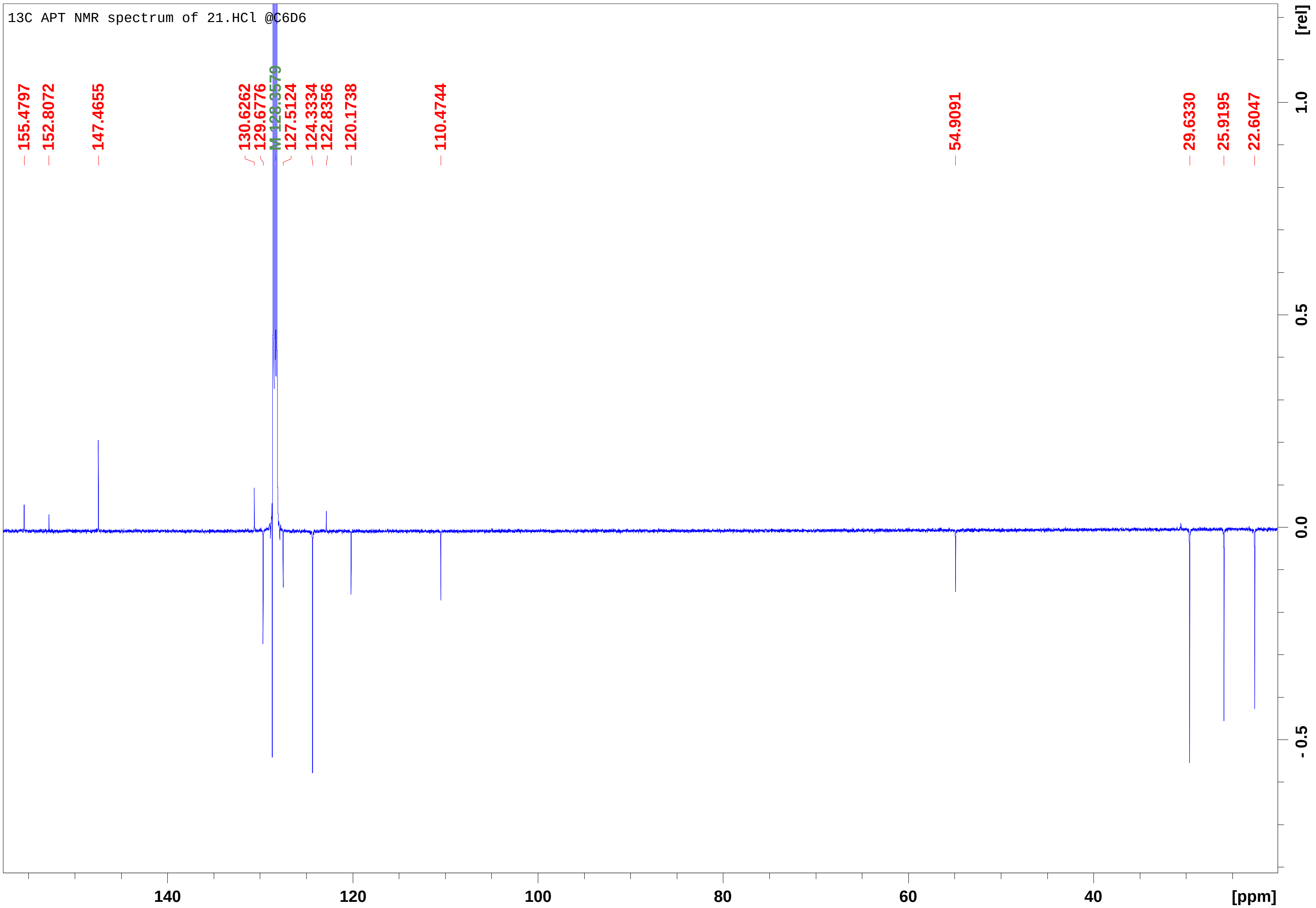

Figure S209. 13C APT NMR spectrum of 21.HCl in C6D6

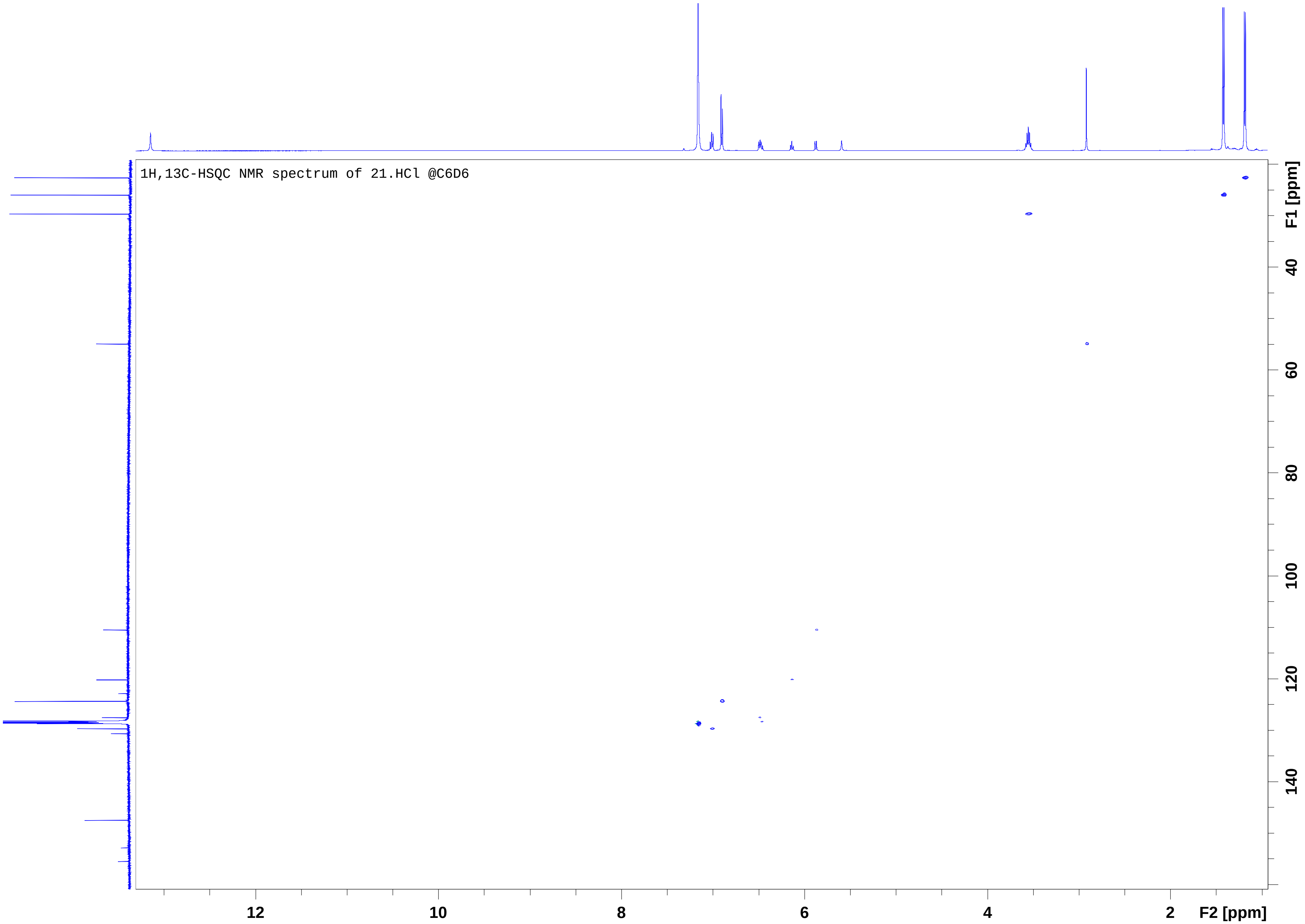

Figure S210. 1H,13C-HSQC NMR spectrum of 21.HCl in C6D6

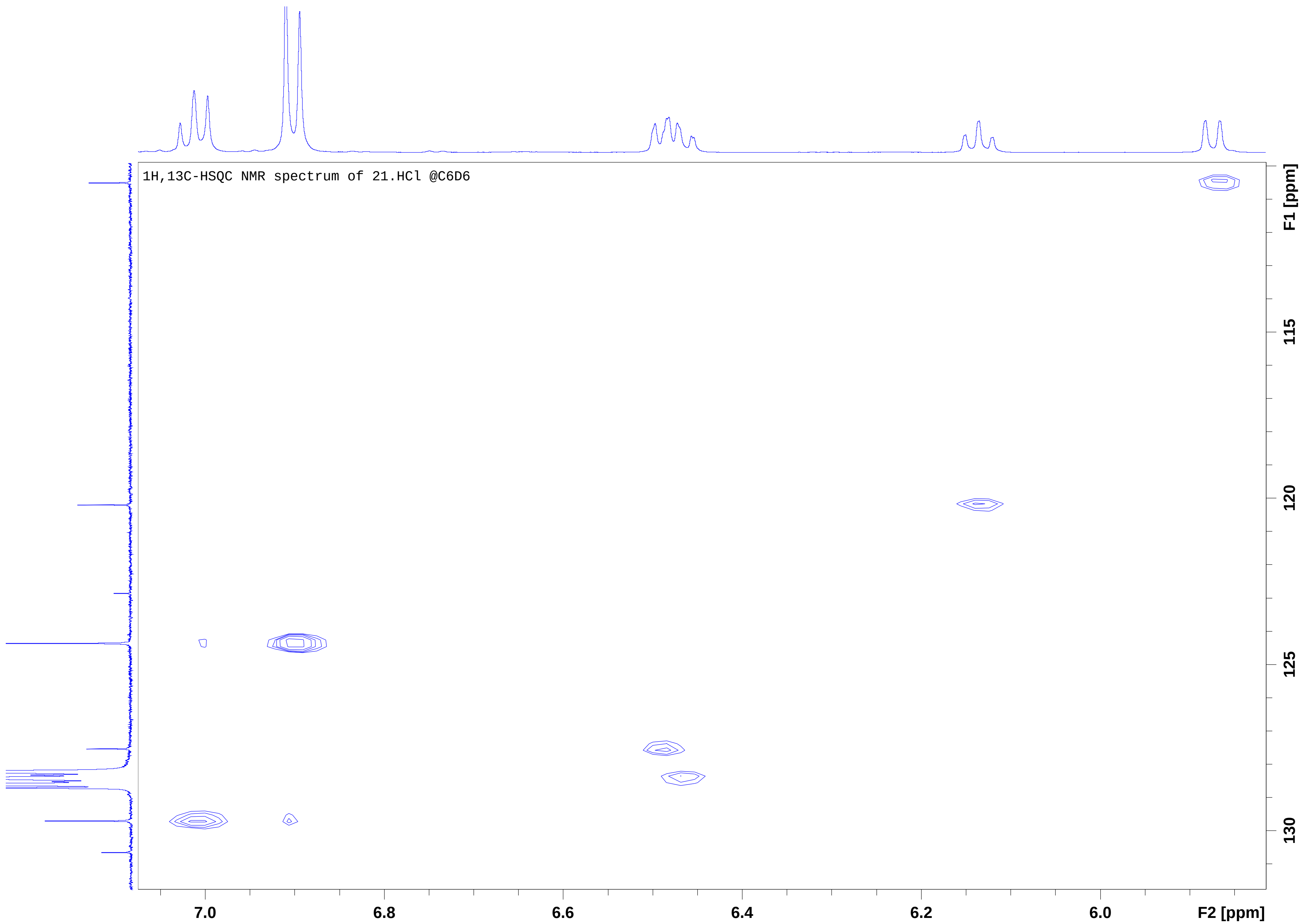

Figure S211. Detail of 1H,13C-HSQC NMR spectrum of 21.HCl in C6D6

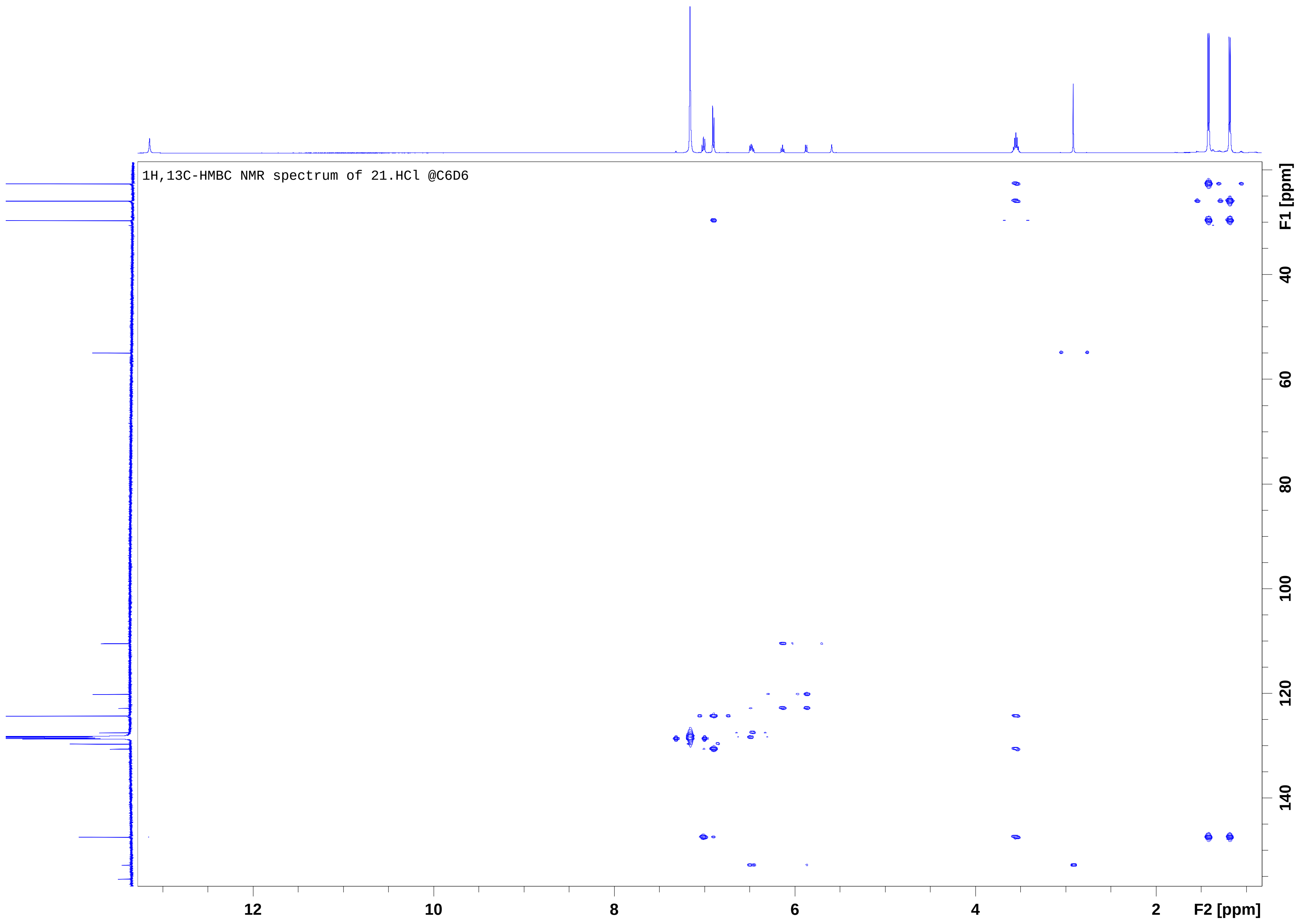

Figure S212. 1H,13C-HMBC NMR spectrum of 21.HCl in C6D6

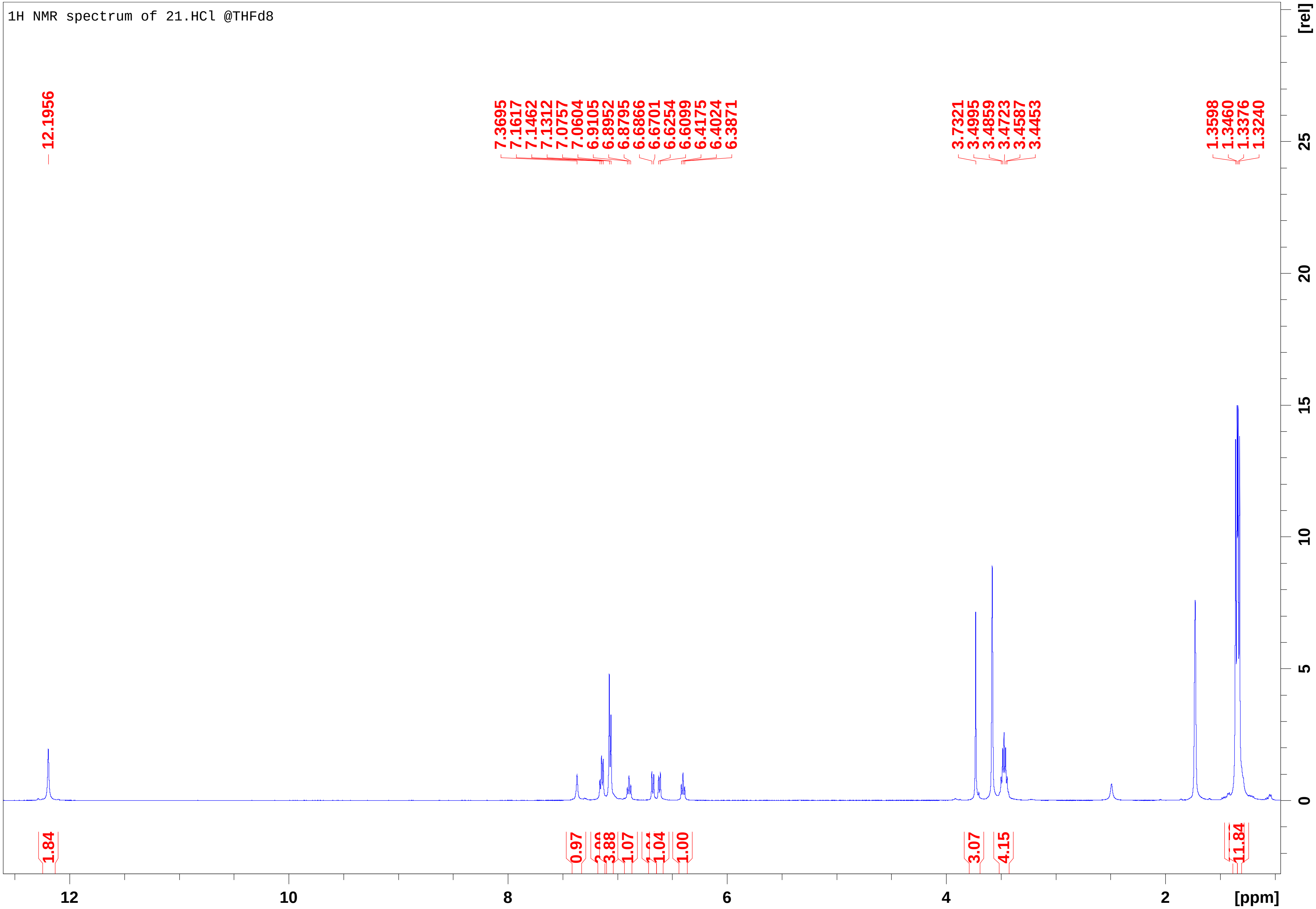

Figure S213. 1H NMR spectrum of 21.HCl in THF-d8

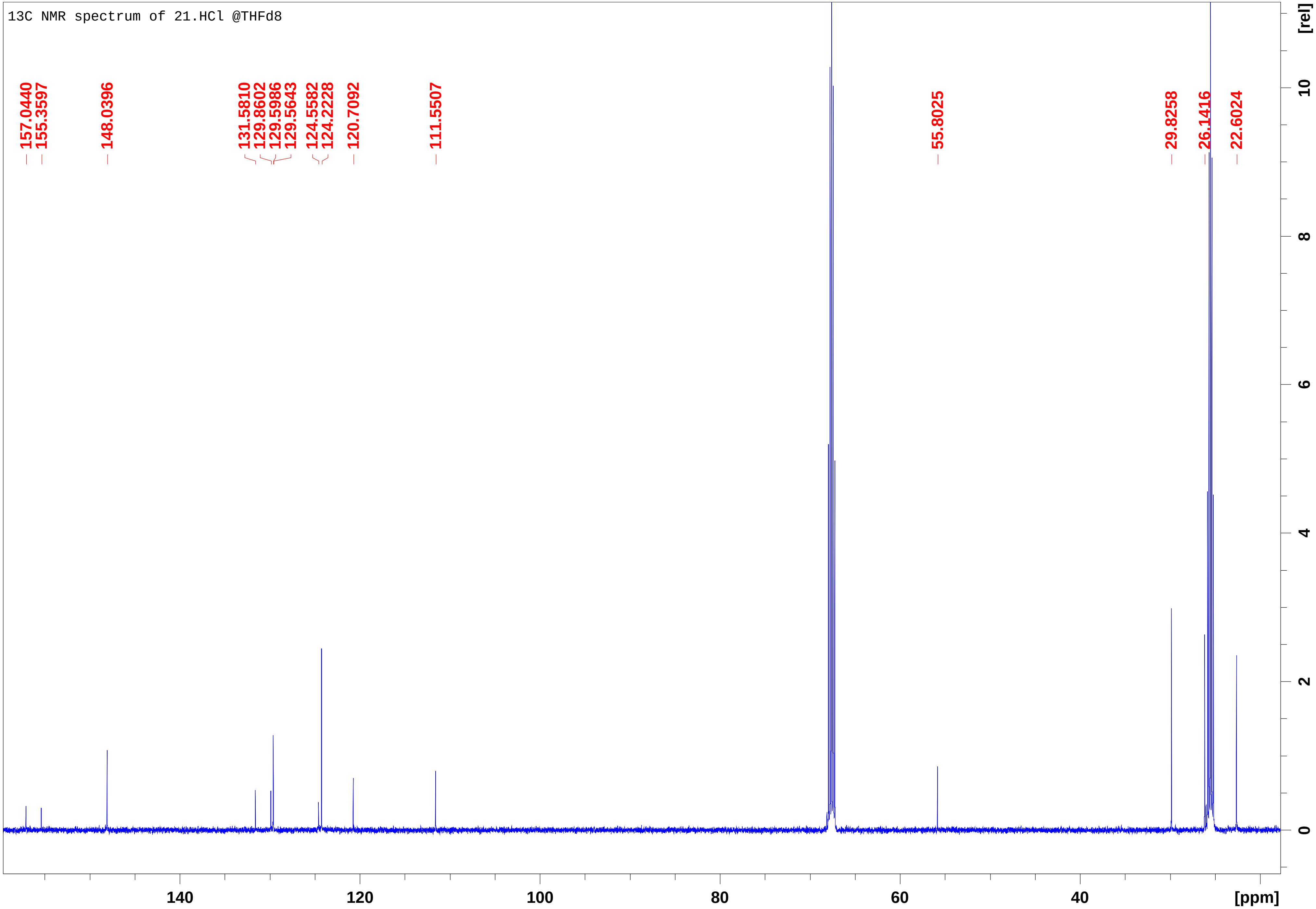

Figure S214. 13C NMR spectrum of 21.HCl in THF-d8

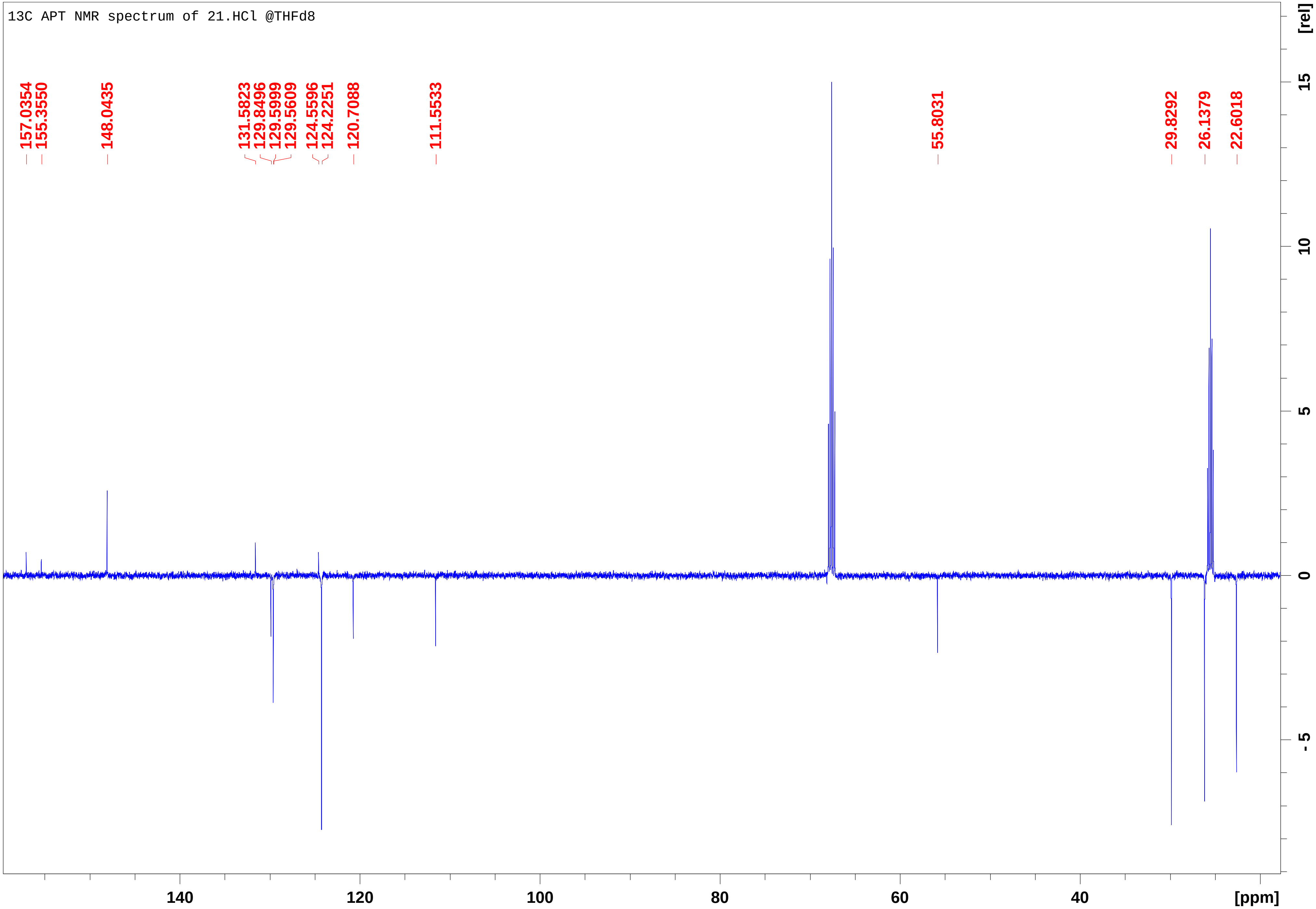

Figure S215. 13C APT NMR spectrum of 21.HCl in THF-d8

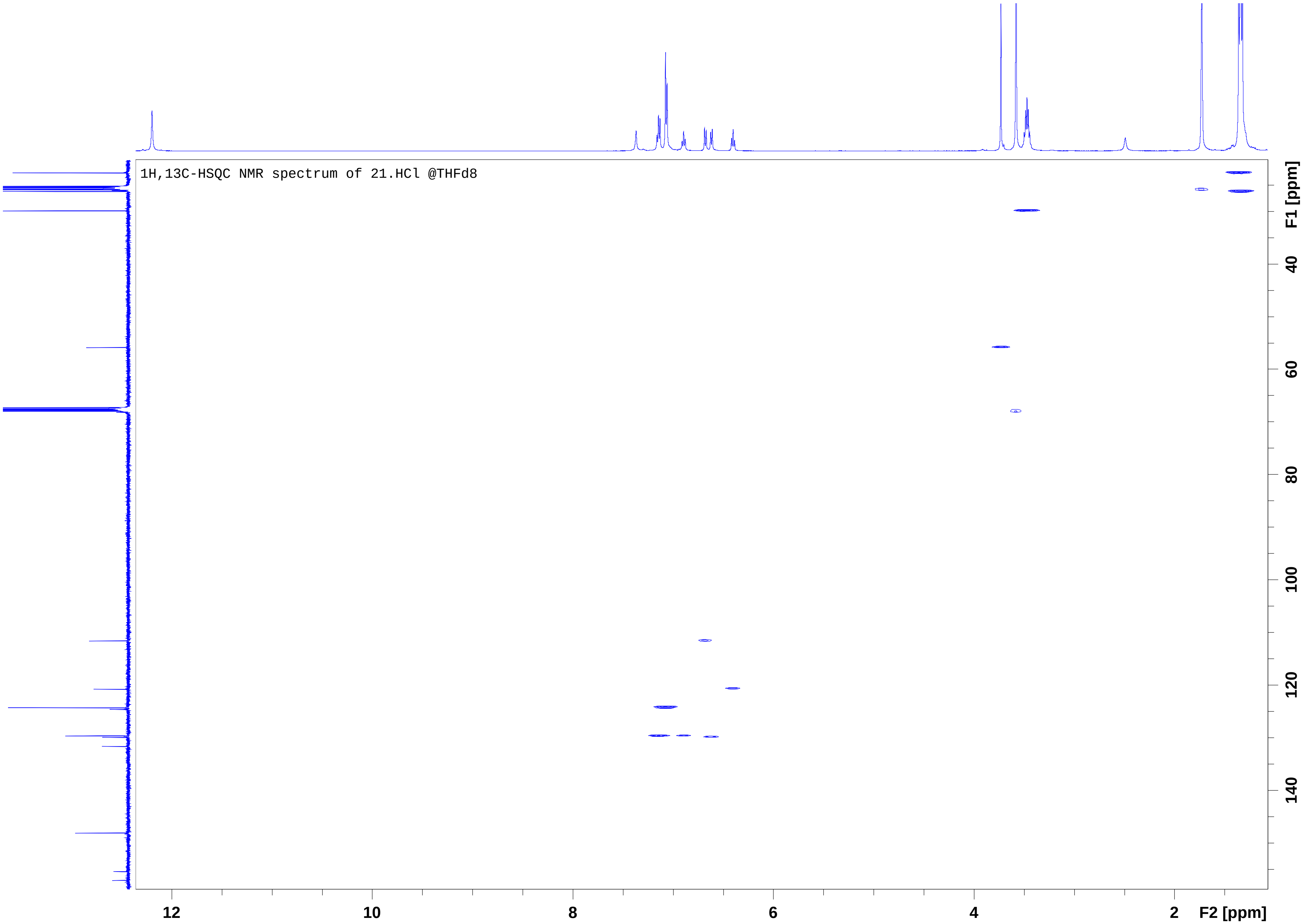

Figure S216. 1H,13C-HSQC NMR spectrum of 21.HCl in THF-d8

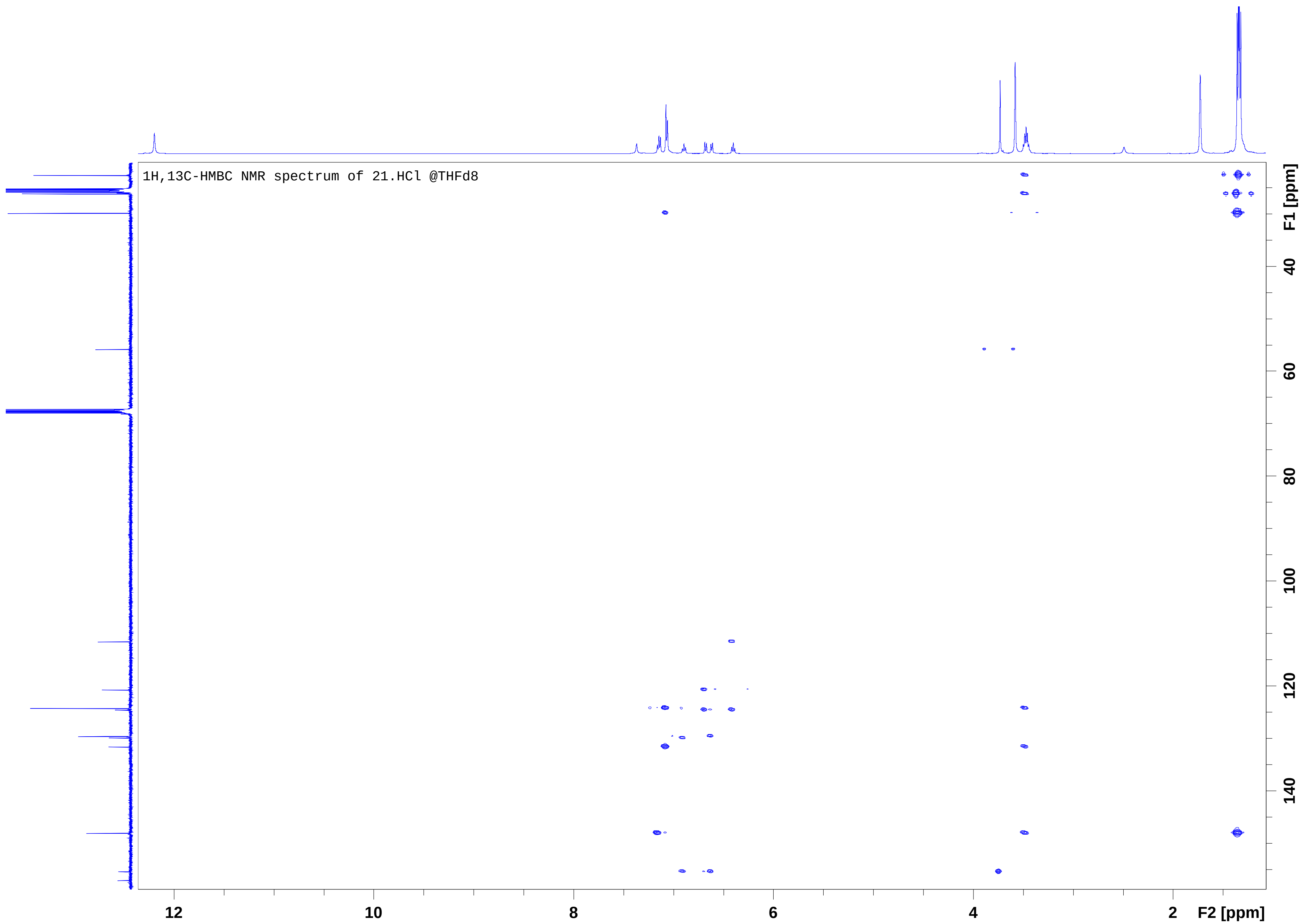

Figure S217. 1H,13C-HMBC NMR spectrum of 21.HCl in THF-d8

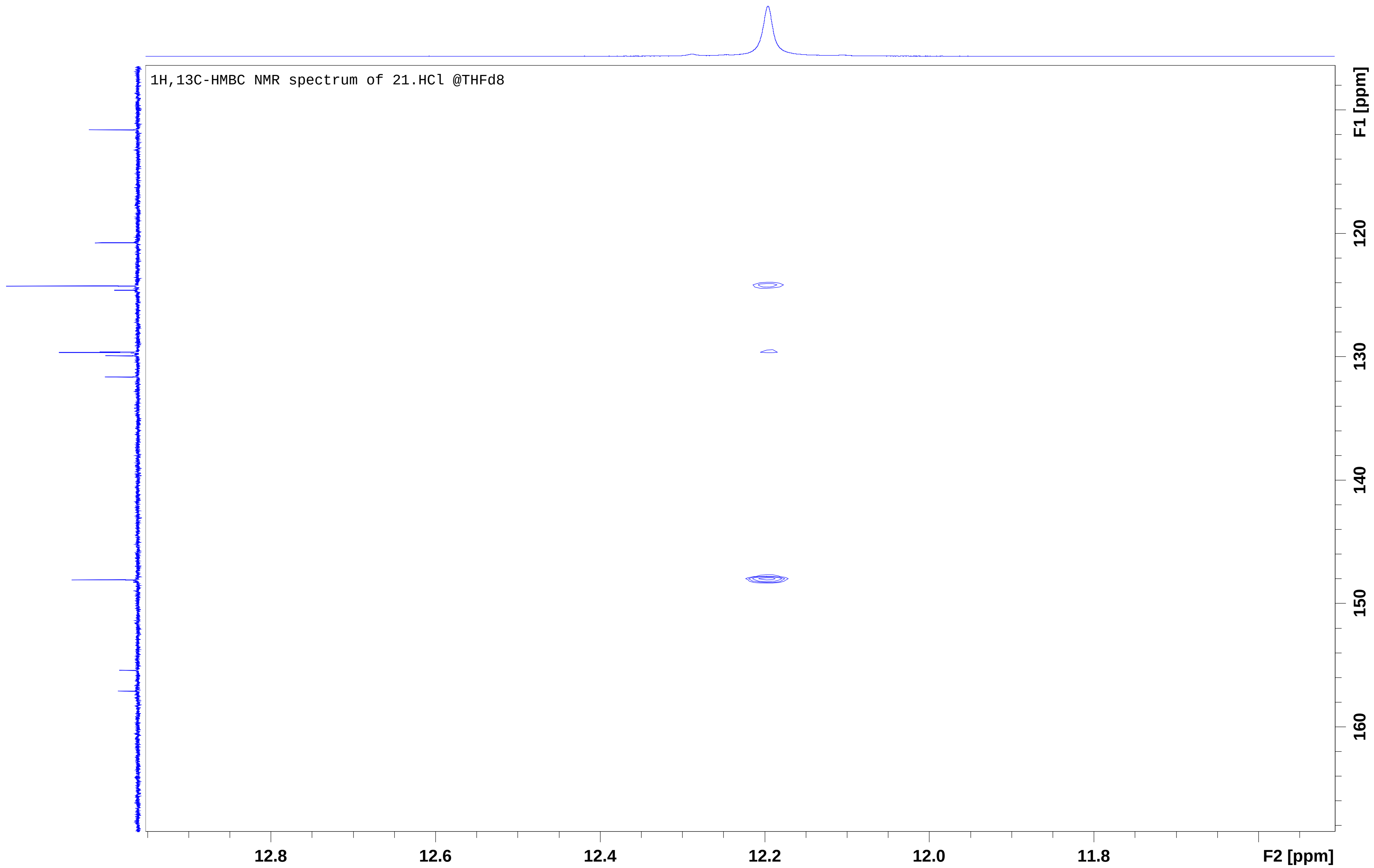

Figure S218. Detail of 1H,13C-HMBC NMR spectrum of 21.HCl in THF-d8

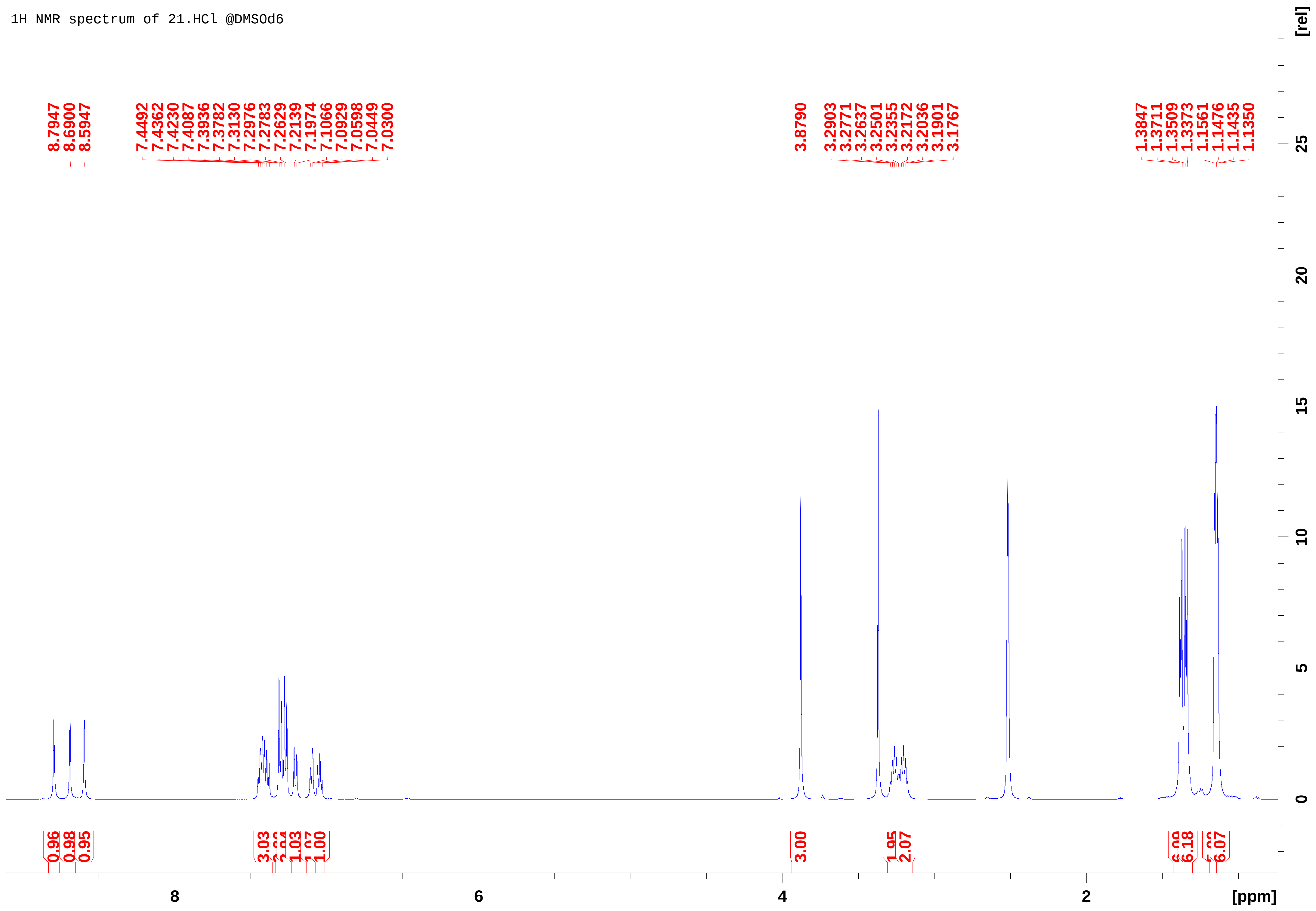

Figure S219. 1H NMR spectrum of 21.HCl in DMSO-d6

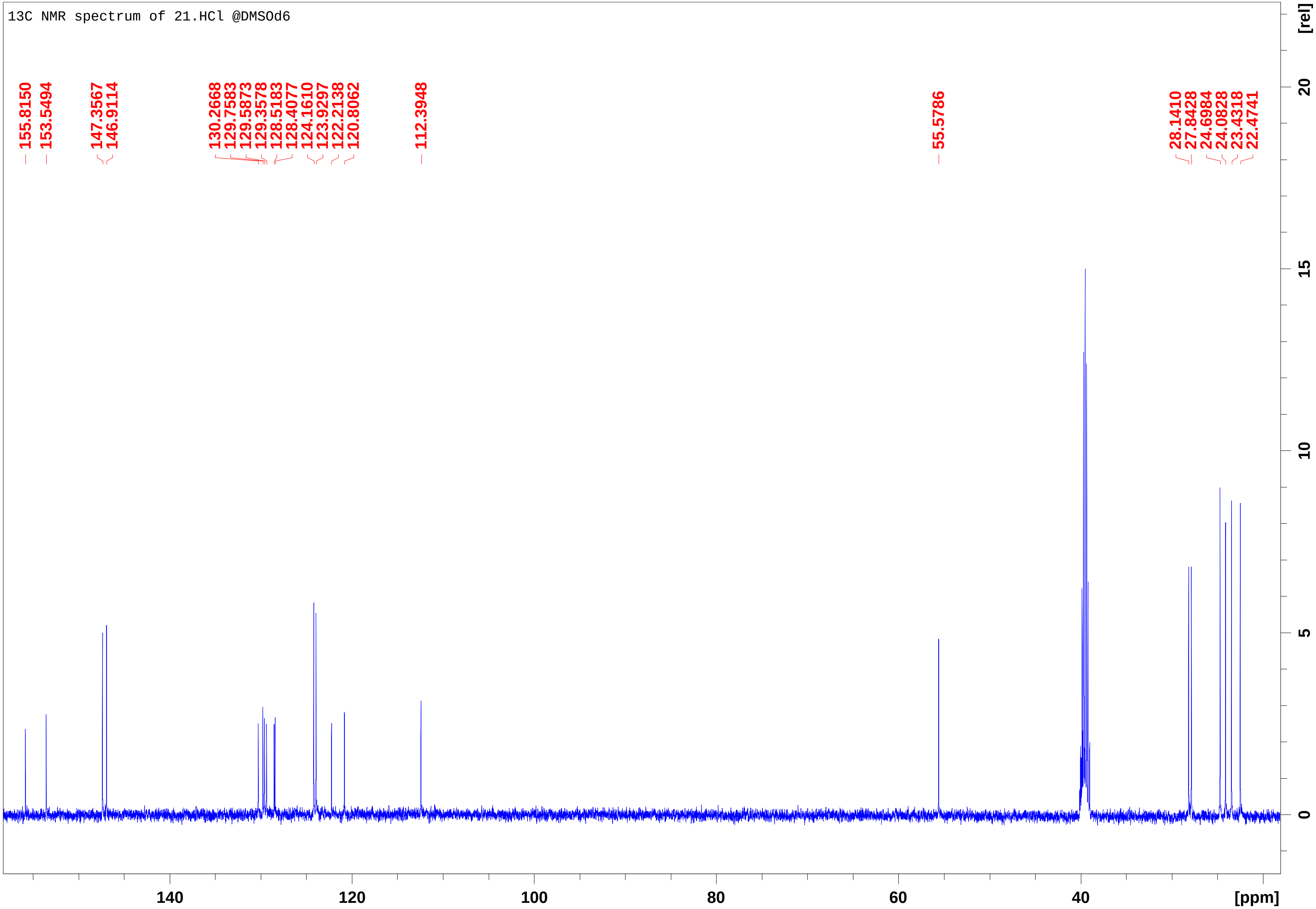

Figure S220. 13C NMR spectrum of 21.HCl in DMSO-d6

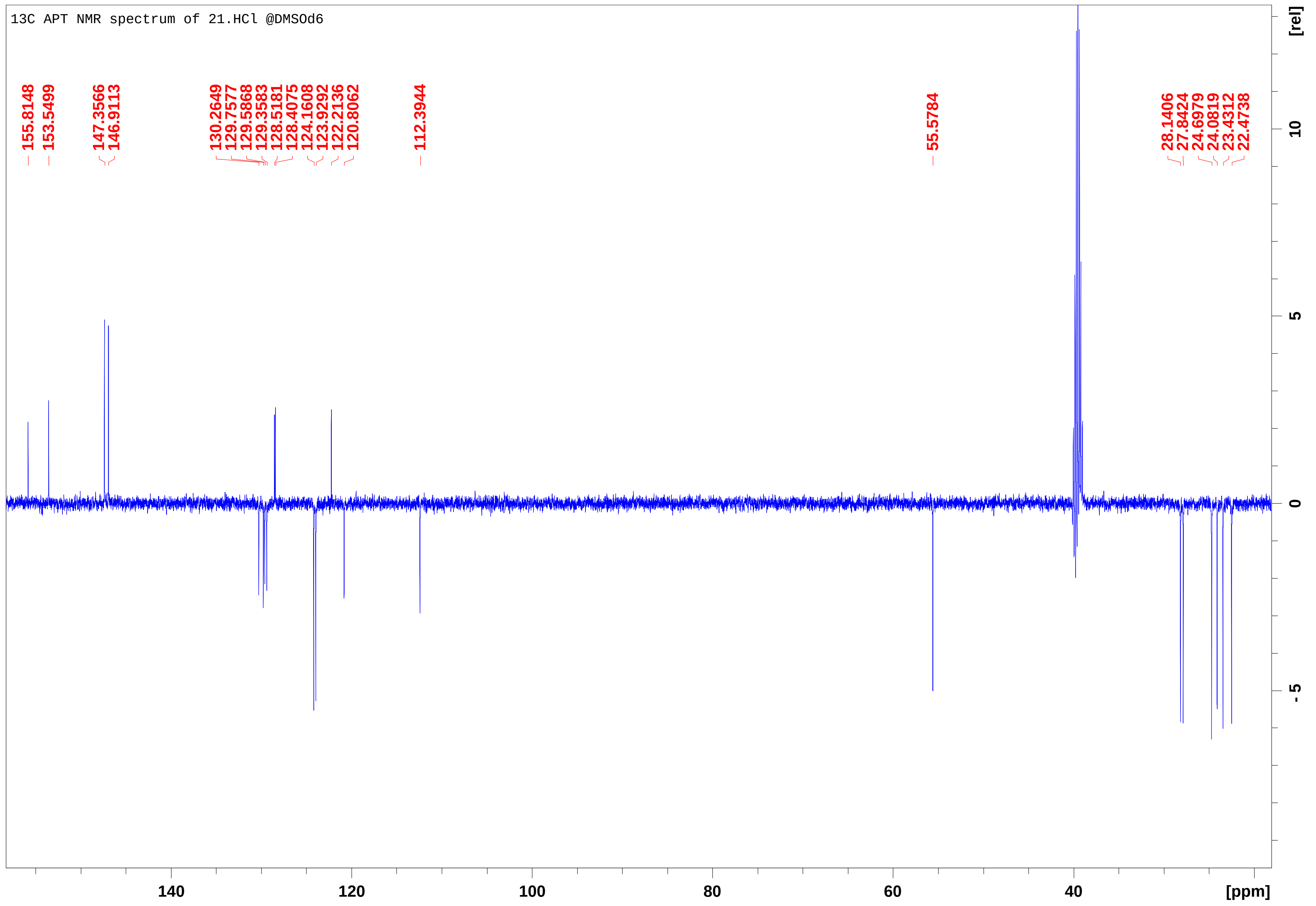

Figure S221. 13C APT NMR spectrum of 21.HCl in DMSO-d6

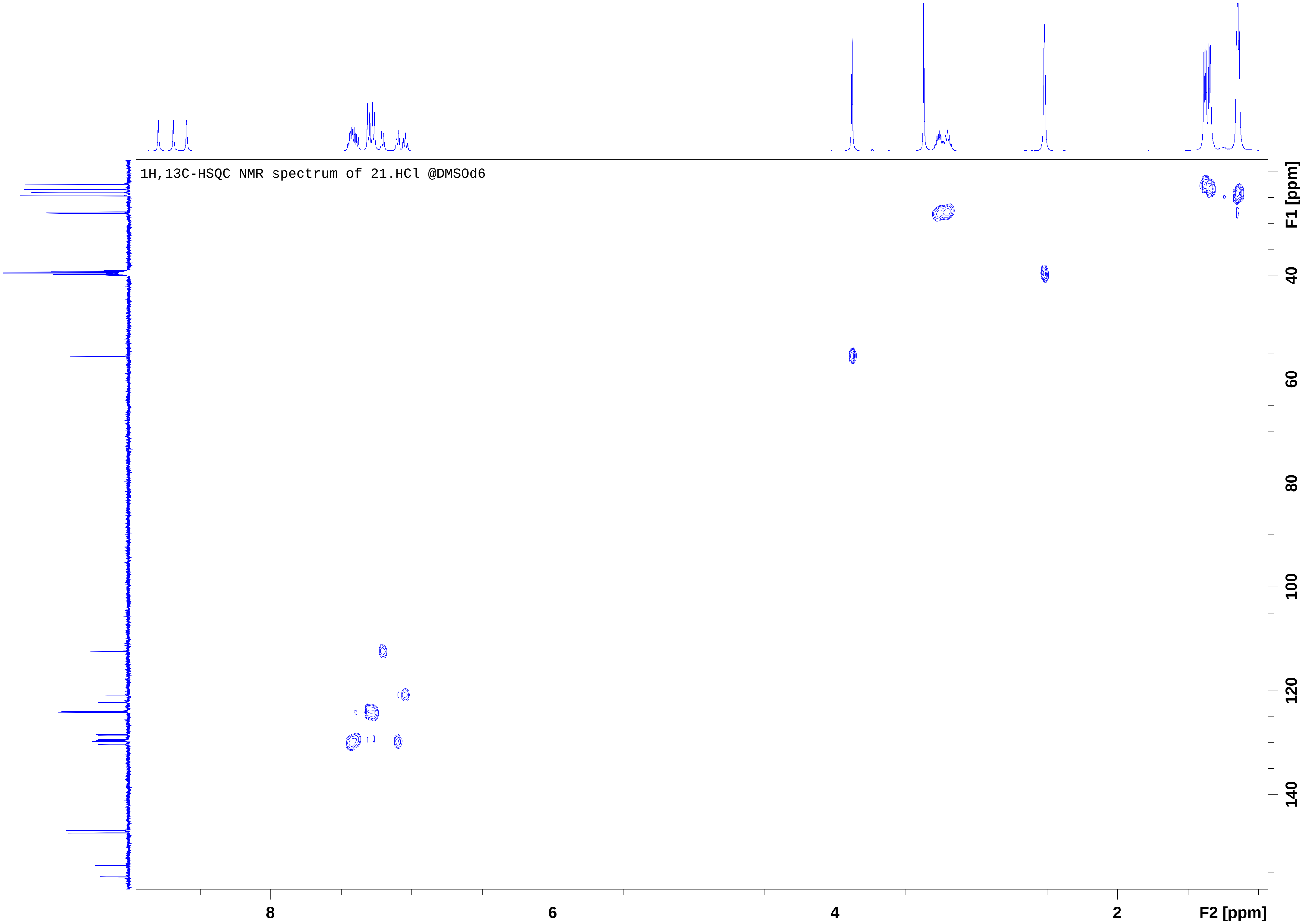

Figure S222. 1H,13C-HSQC NMR spectrum of 21.HCl in DMSO-d6

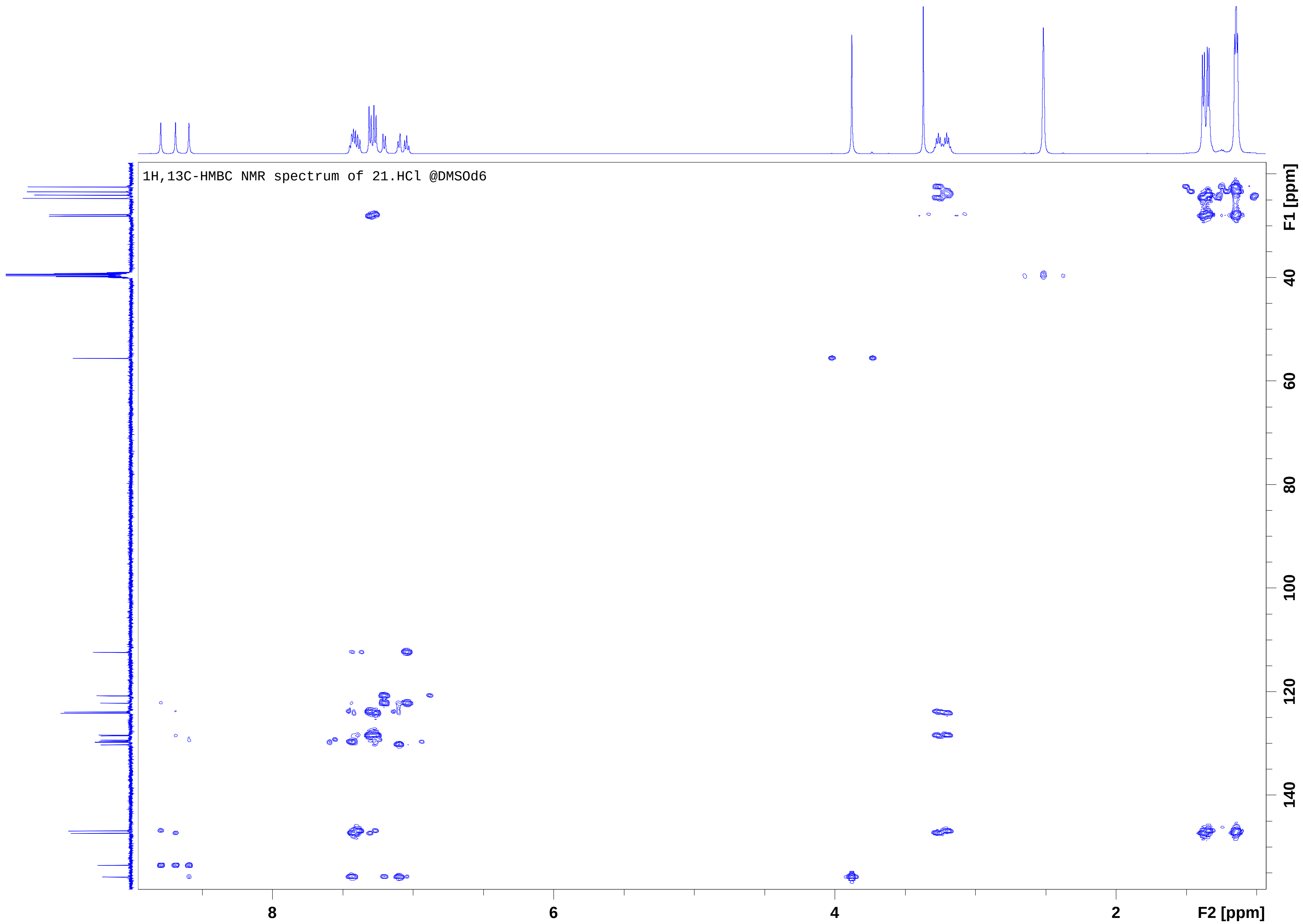

Figure S223. 1H,13C-HMBC NMR spectrum of 21.HCl in DMSO-d6

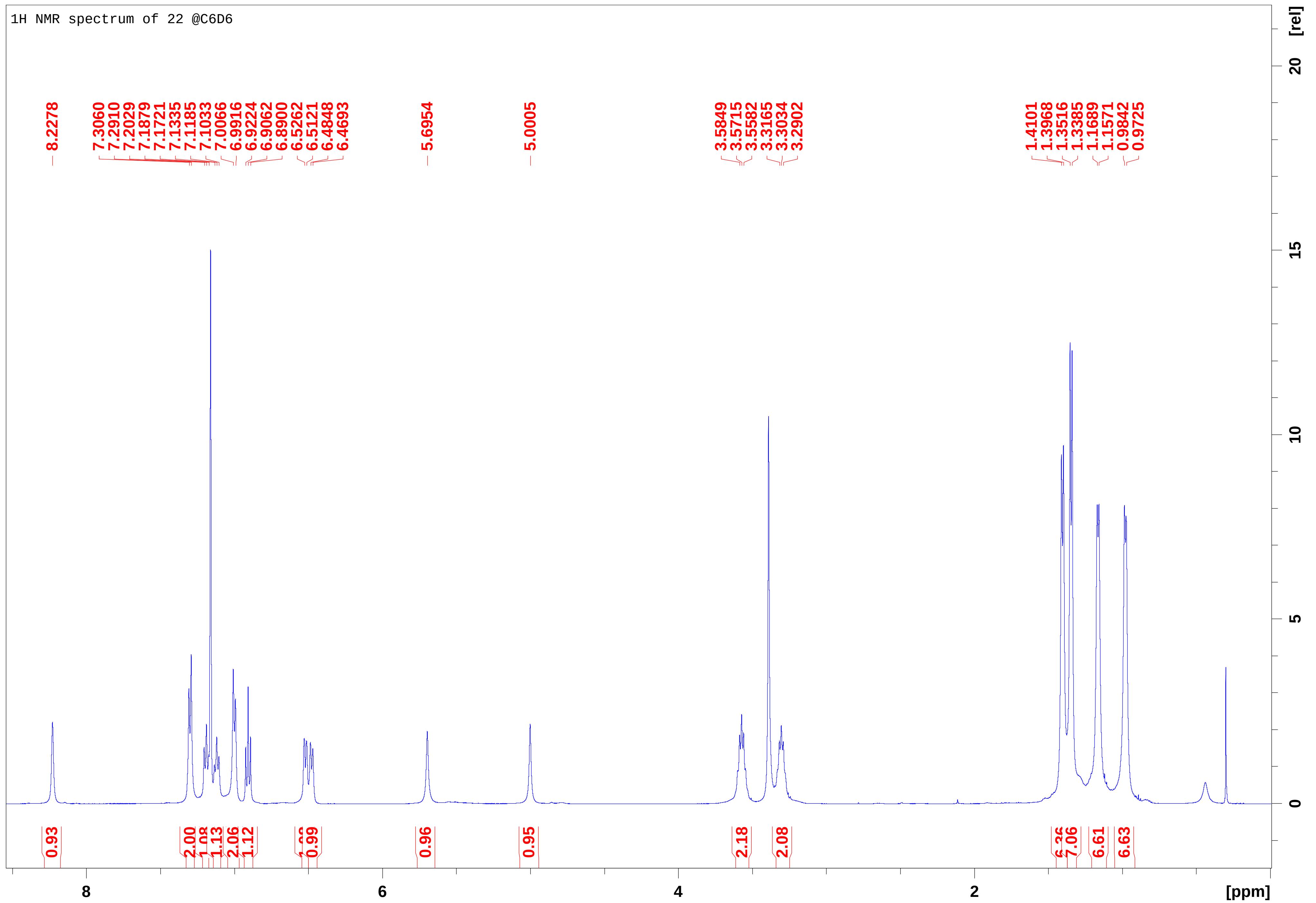

Figure S224. 1H NMR spectrum of 22 in C6D6

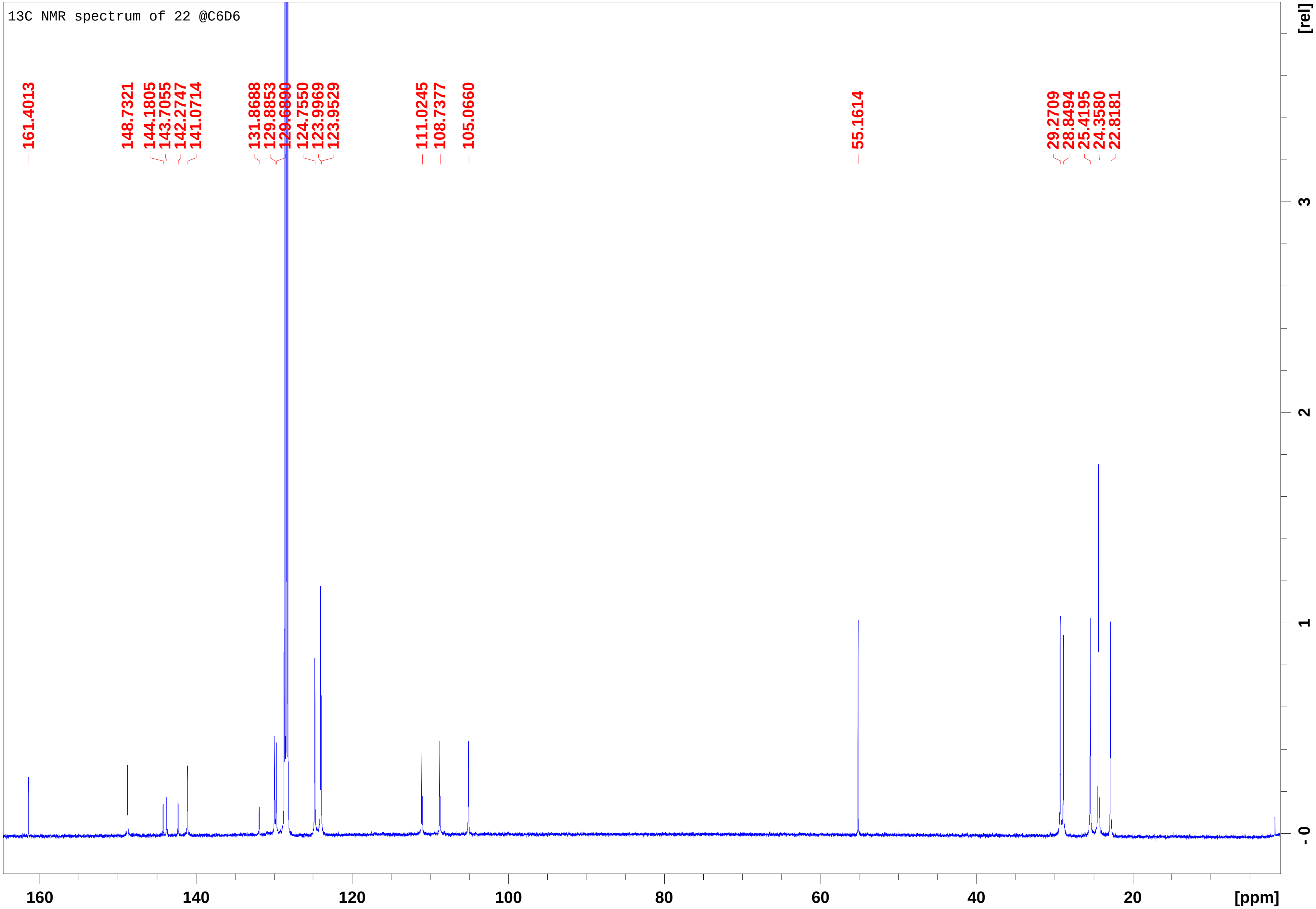

Figure S225. 13C NMR spectrum of 22 in C6D6

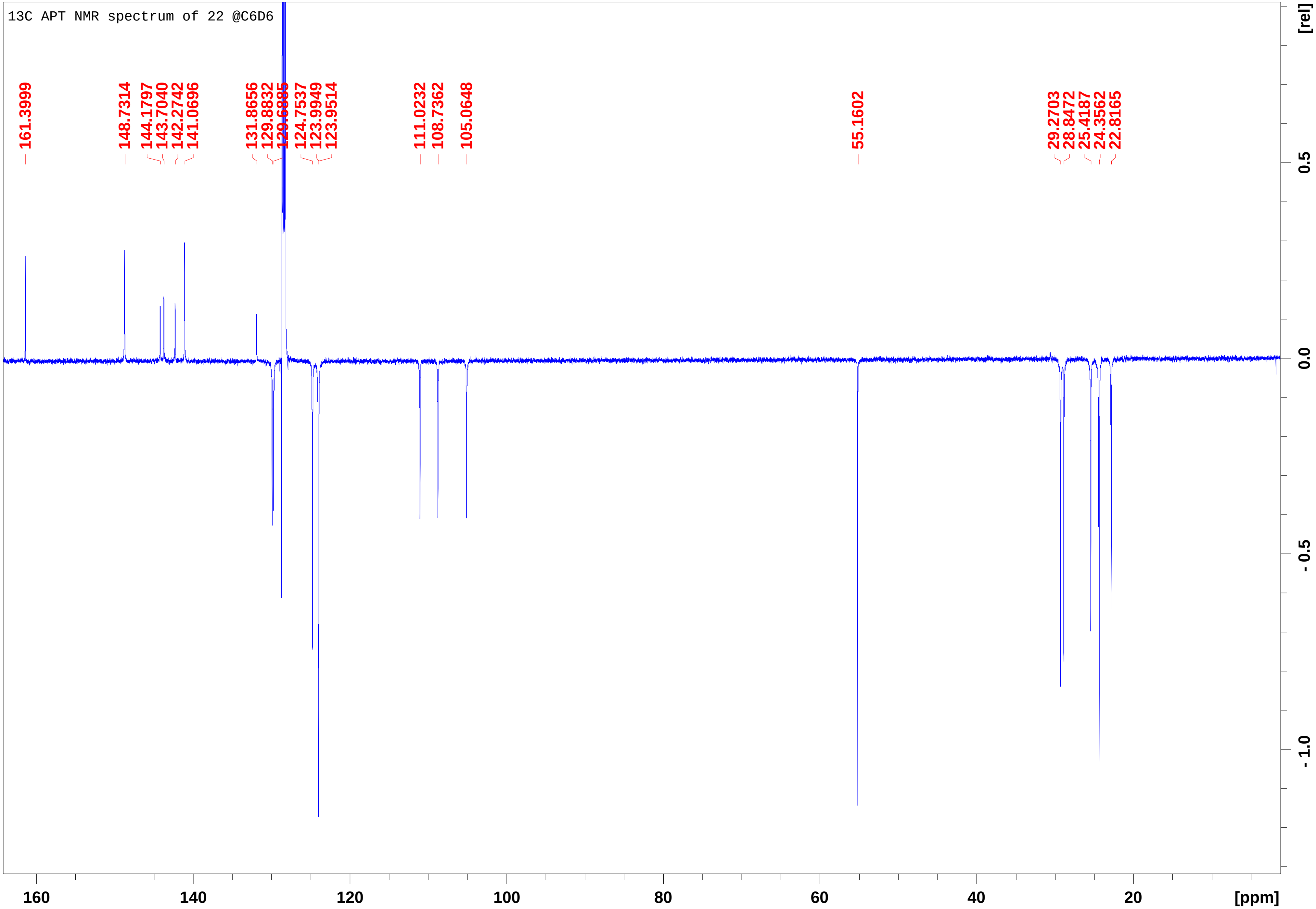

Figure S226. 13C APT NMR spectrum of 22 in C6D6

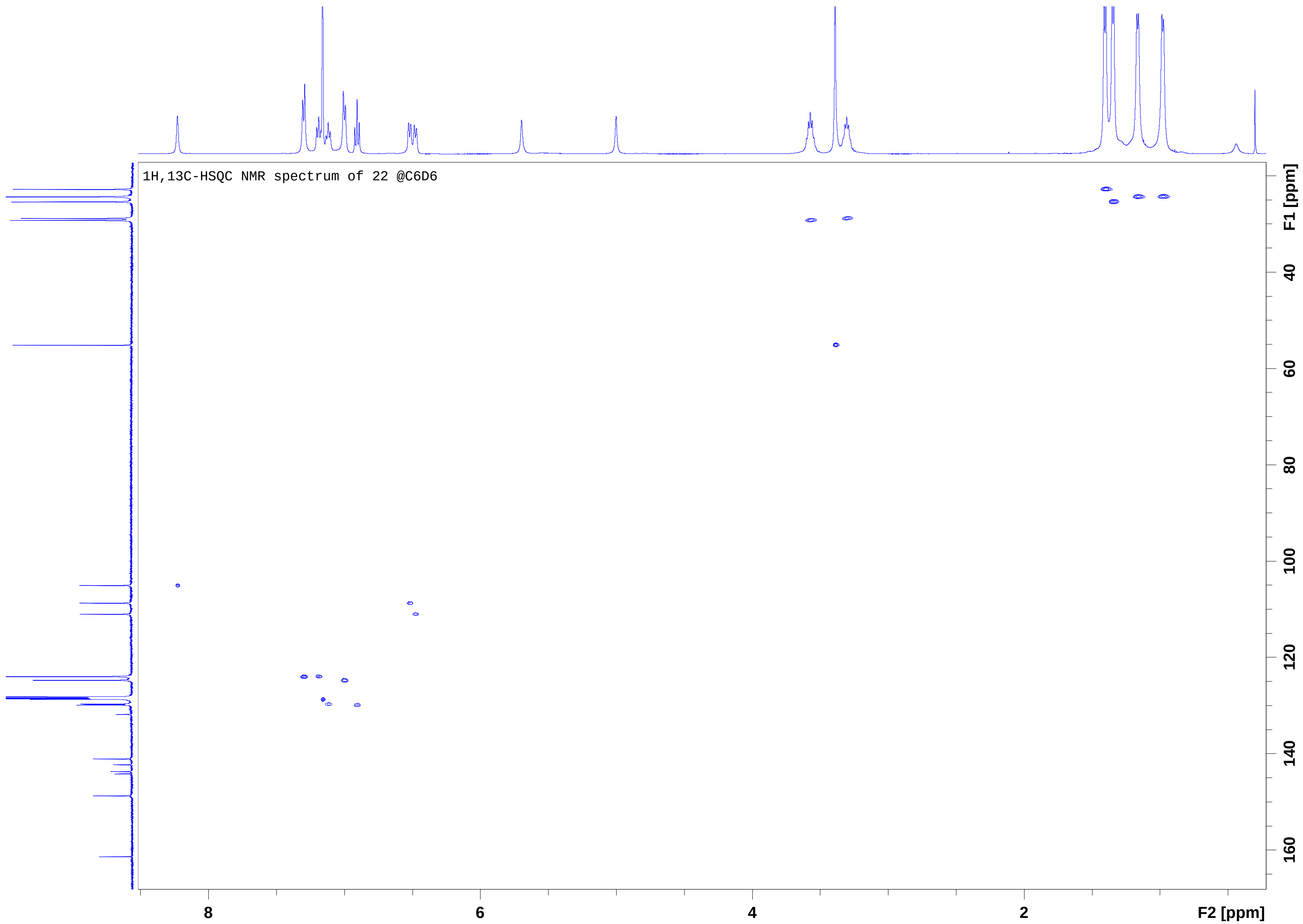

Figure S227. 1H,13C-HSQC NMR spectrum of 22 in C6D6

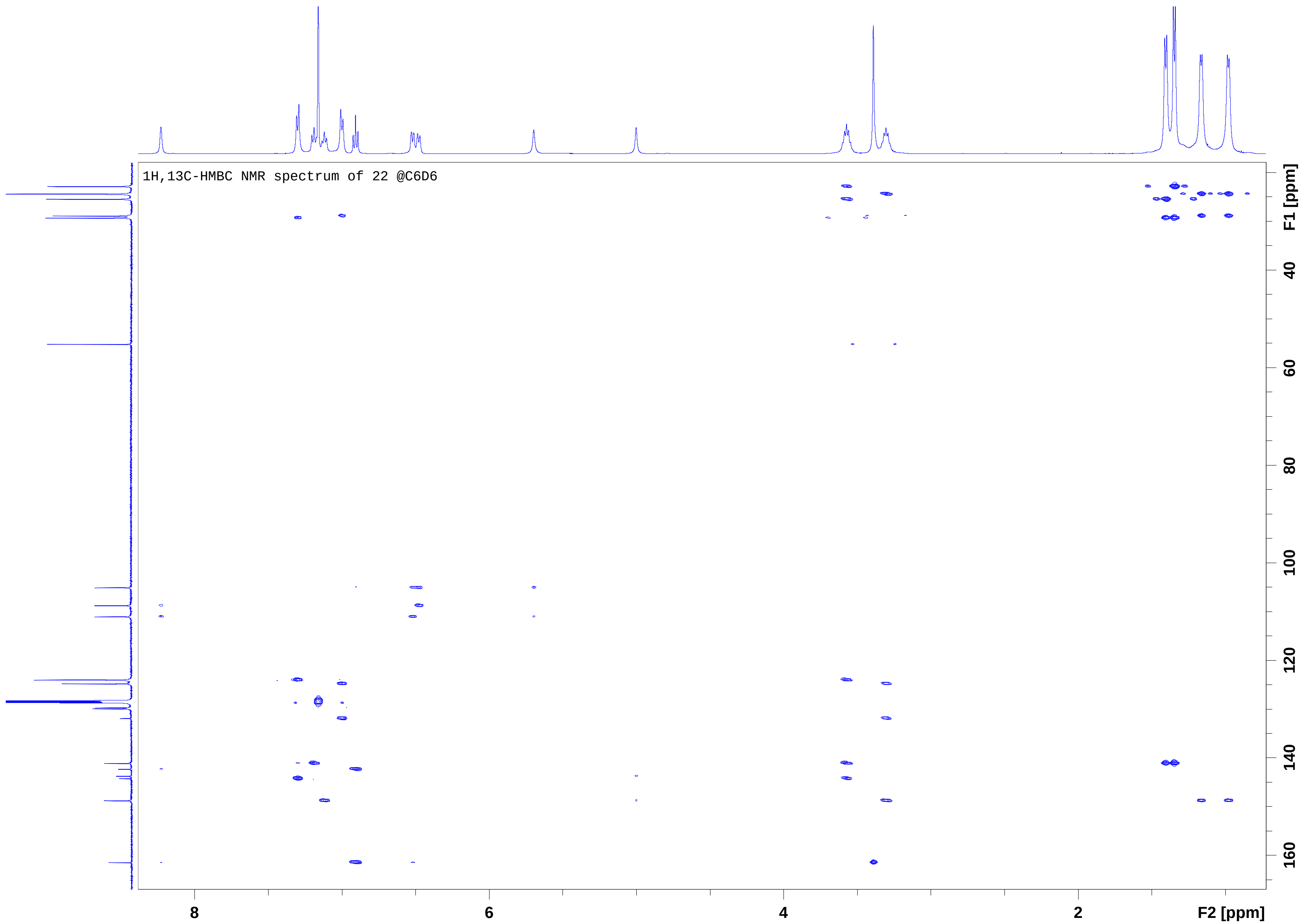

Figure S228. 1H,13C-HMBC NMR spectrum of 22 in C6D6

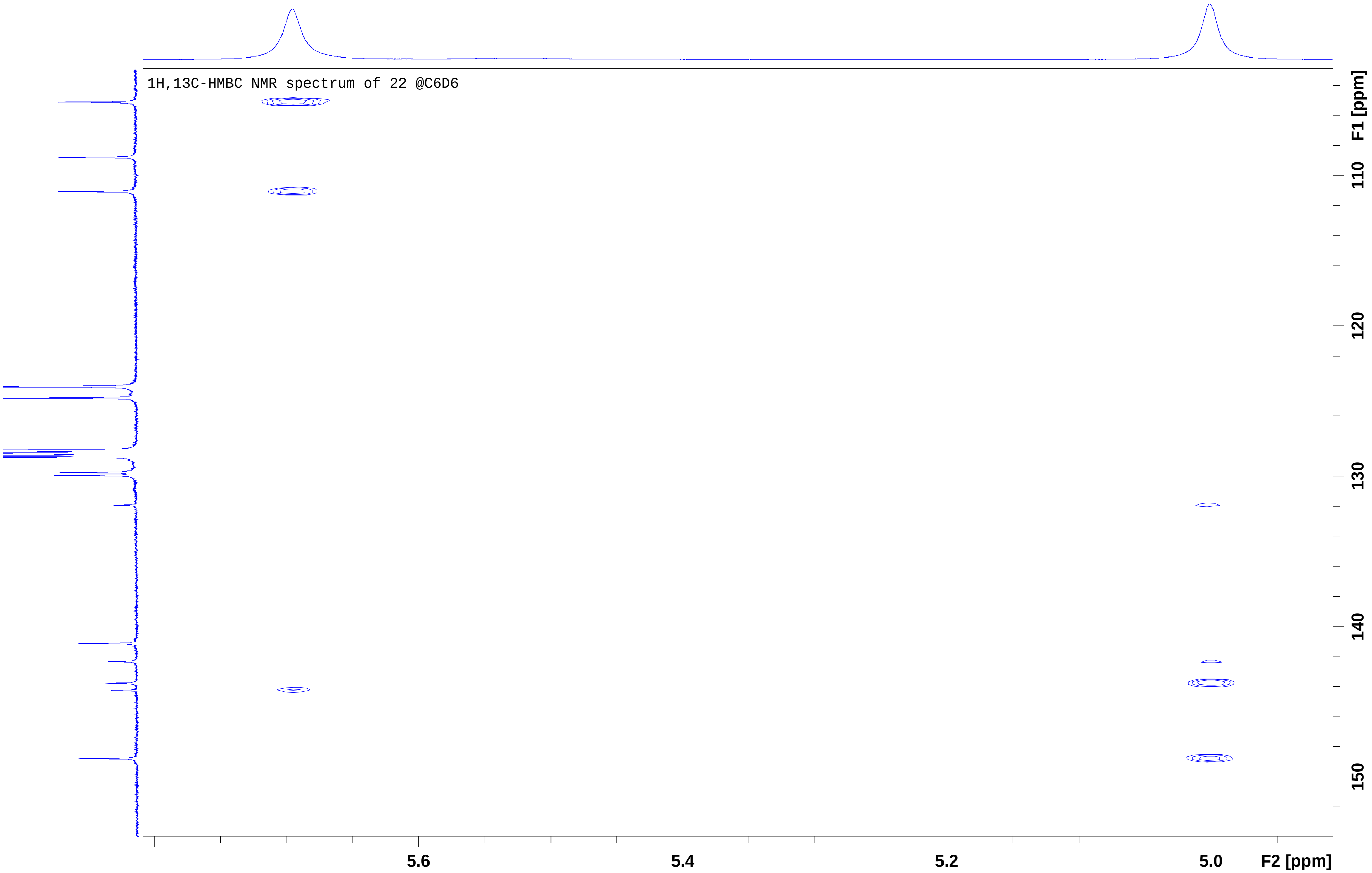

Figure S229. Detail of 1H,13C-HMBC NMR spectrum of 22 in C6D6

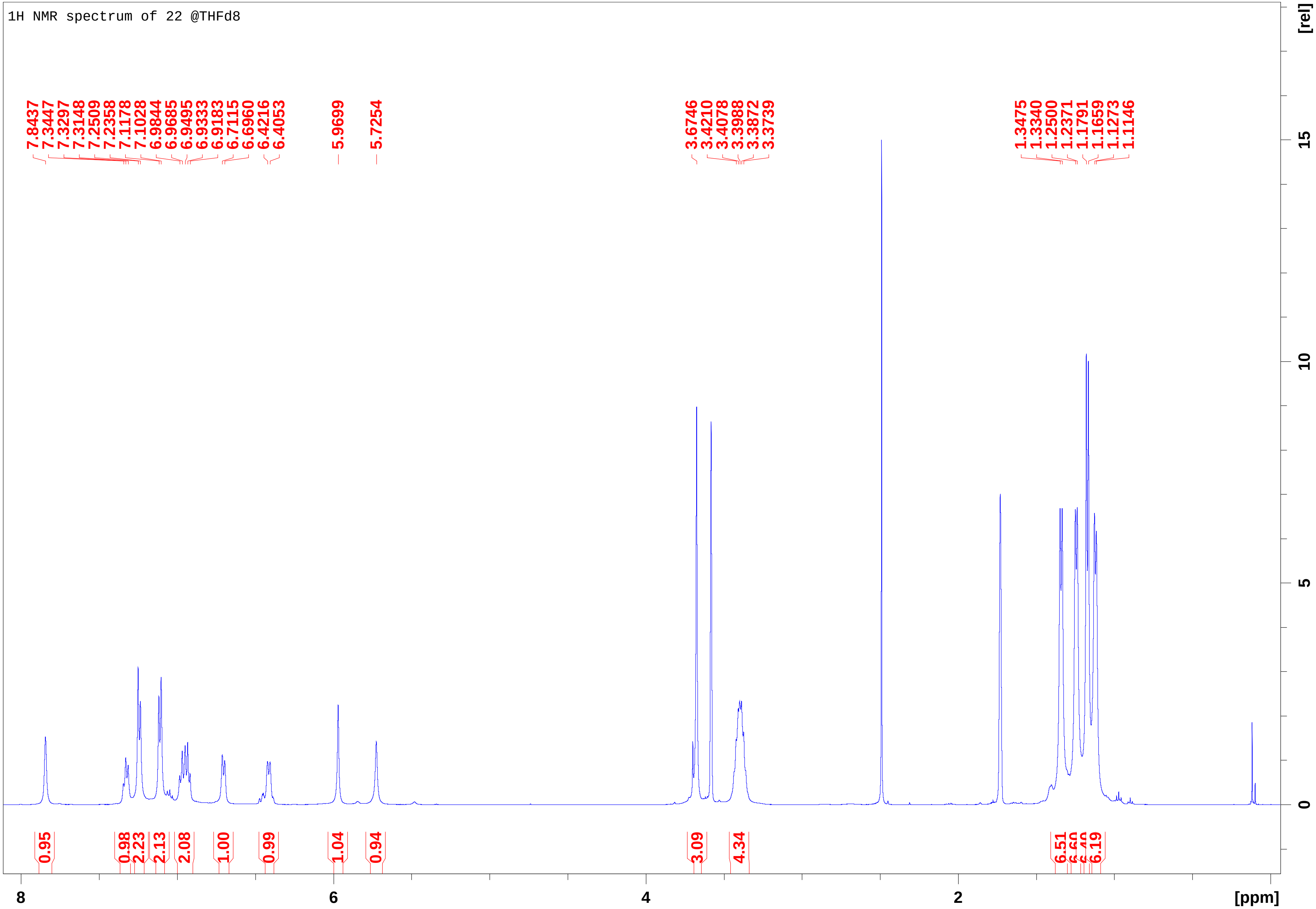

Figure S230. 1H NMR spectrum of 22 in THF-d8

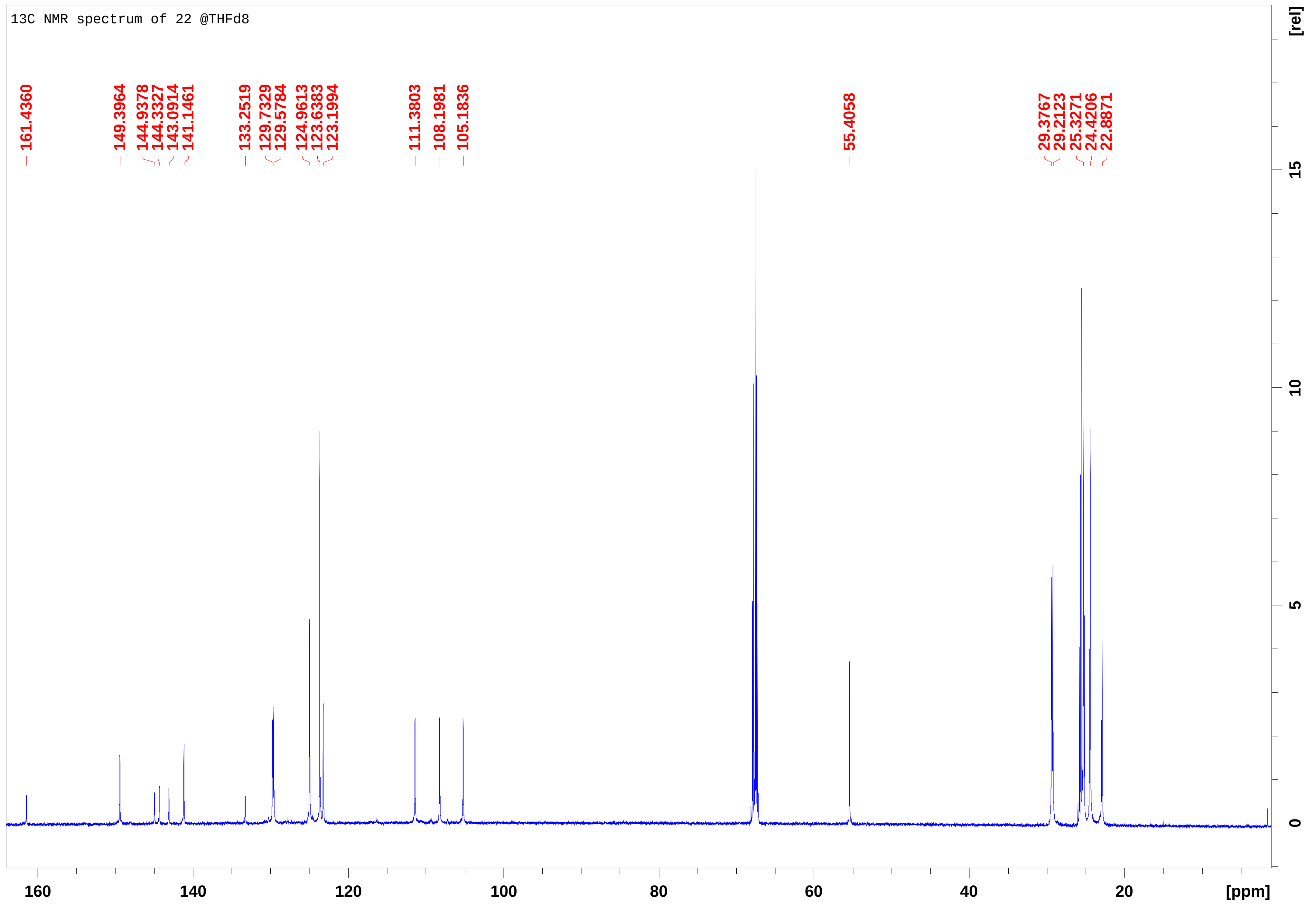

Figure S231. 13C NMR spectrum of 22 in THF-d8

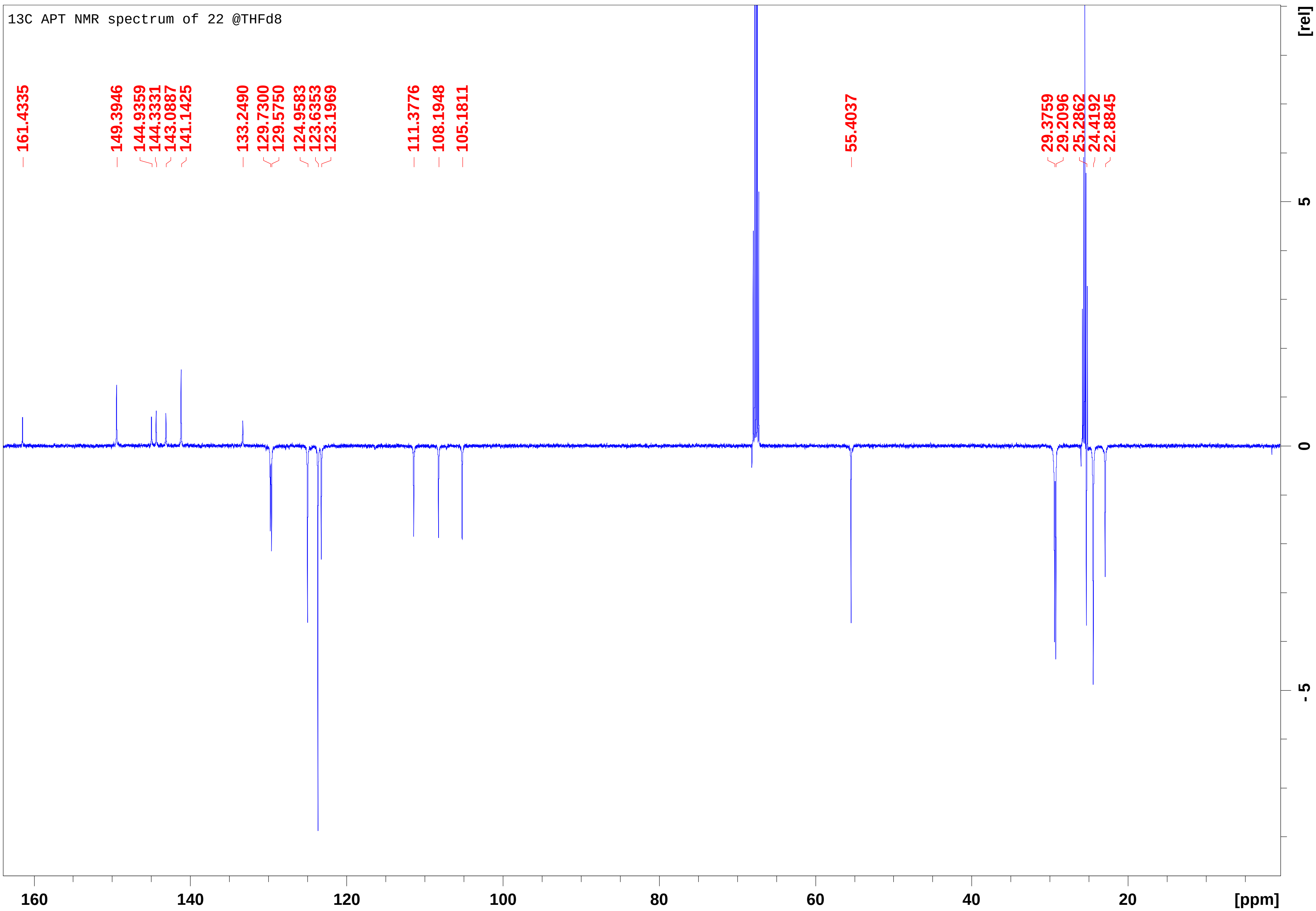

Figure S232. 13C APT NMR spectrum of 22 in THF-d8

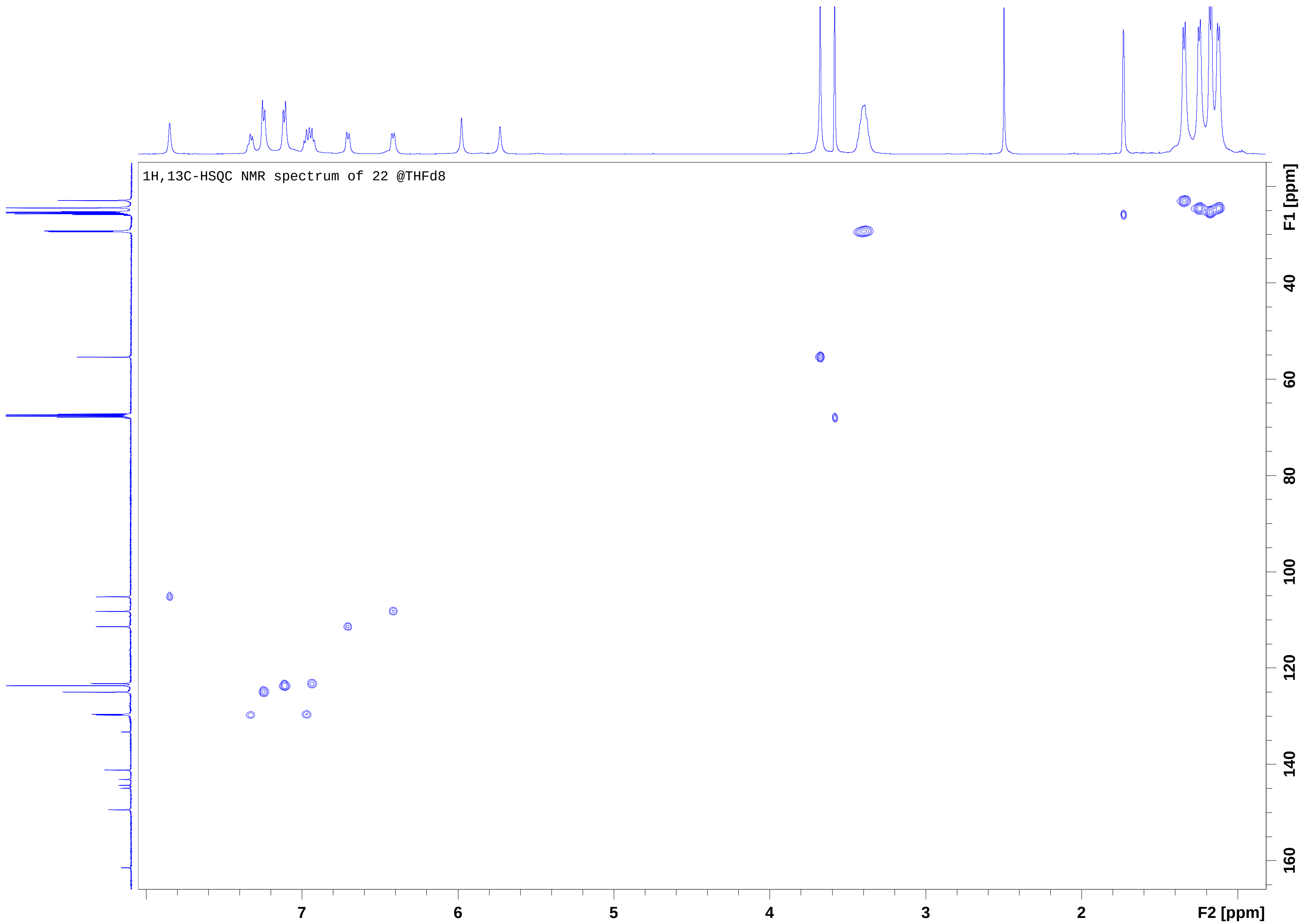

Figure S233. 1H,13C-HSQC NMR spectrum of 22 in THF-d8

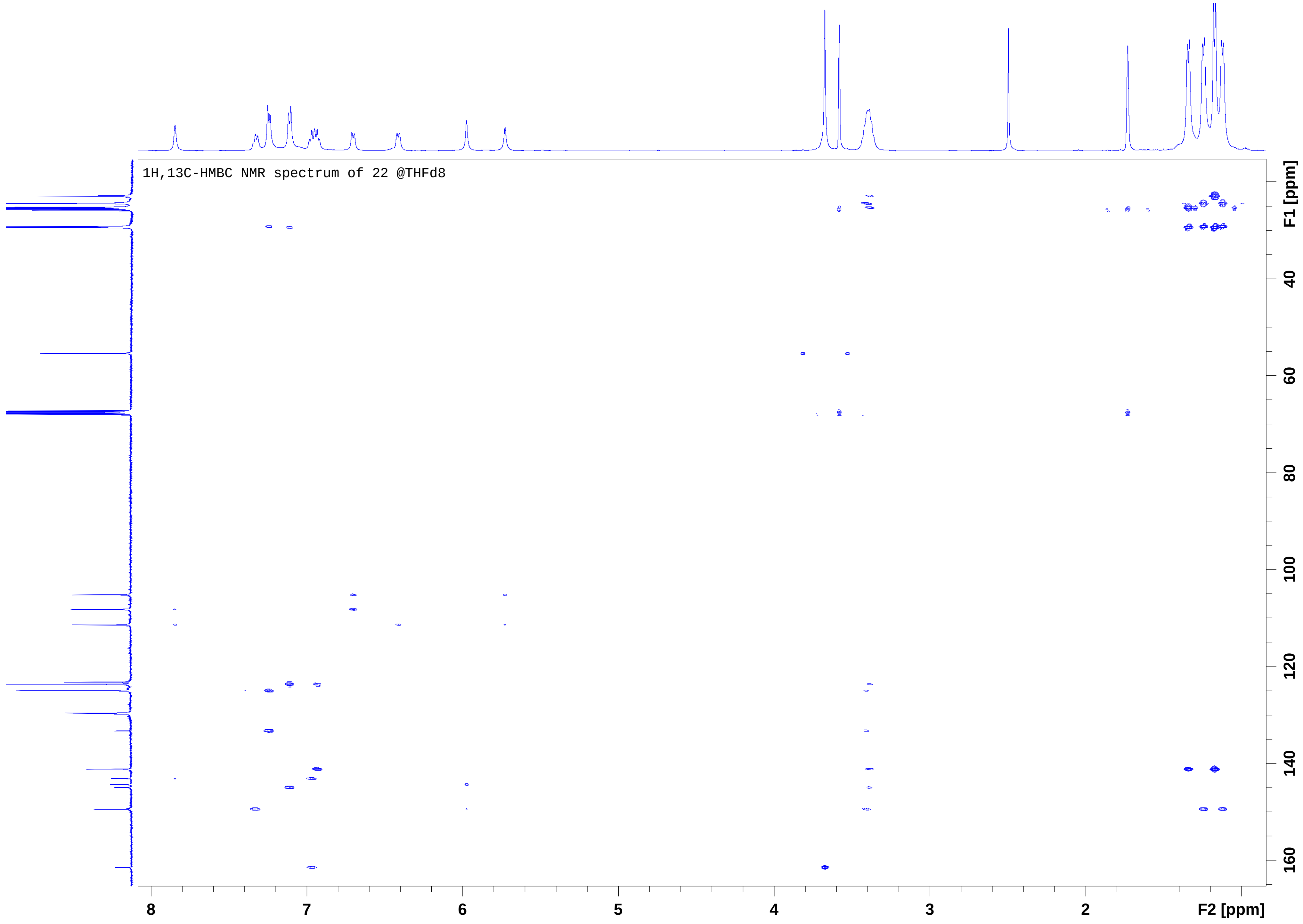

Figure S234. 1H,13C-HMBC NMR spectrum of 22 in THF-d8

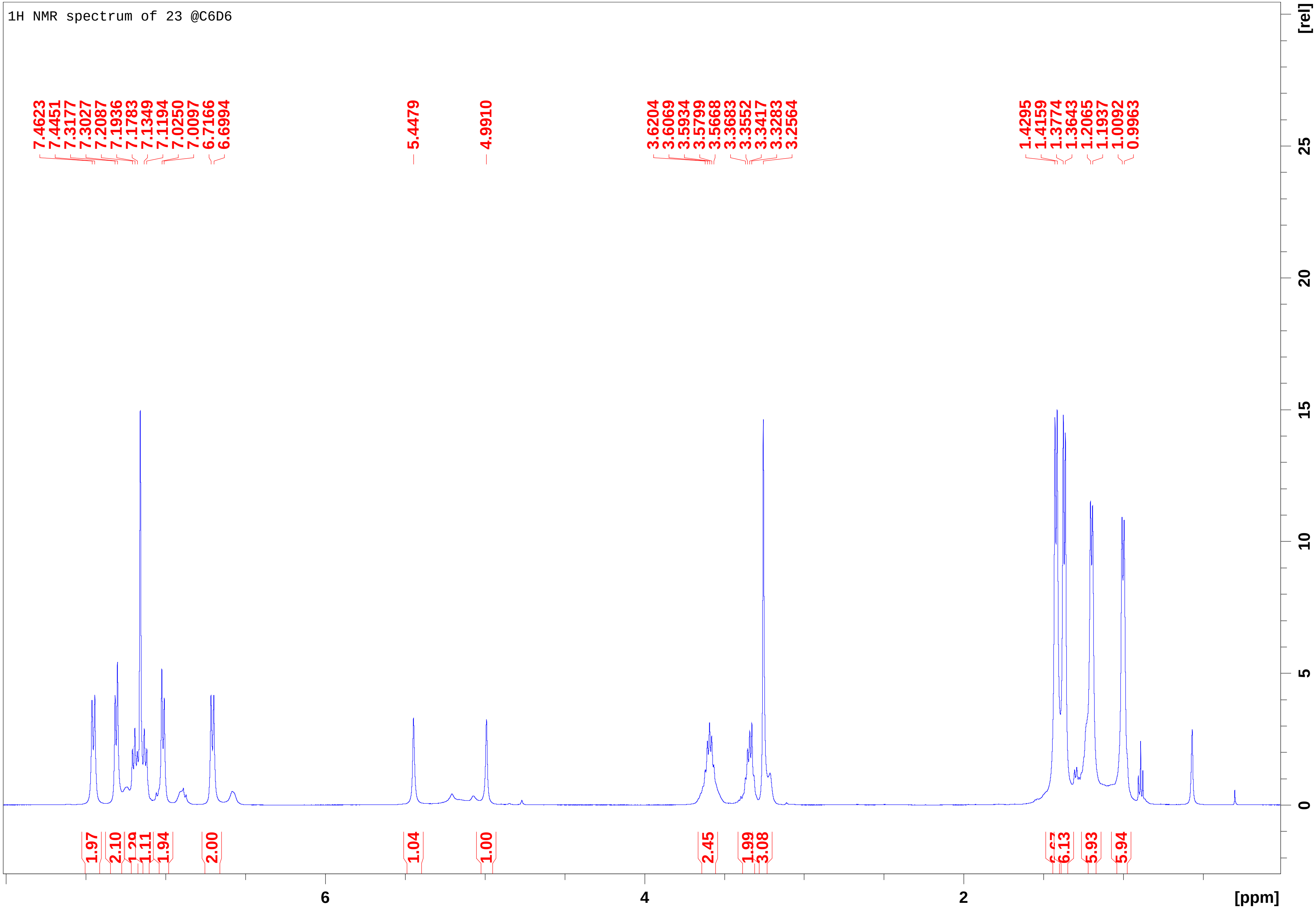

Figure S235. 1H NMR spectrum of 23 in C6D6

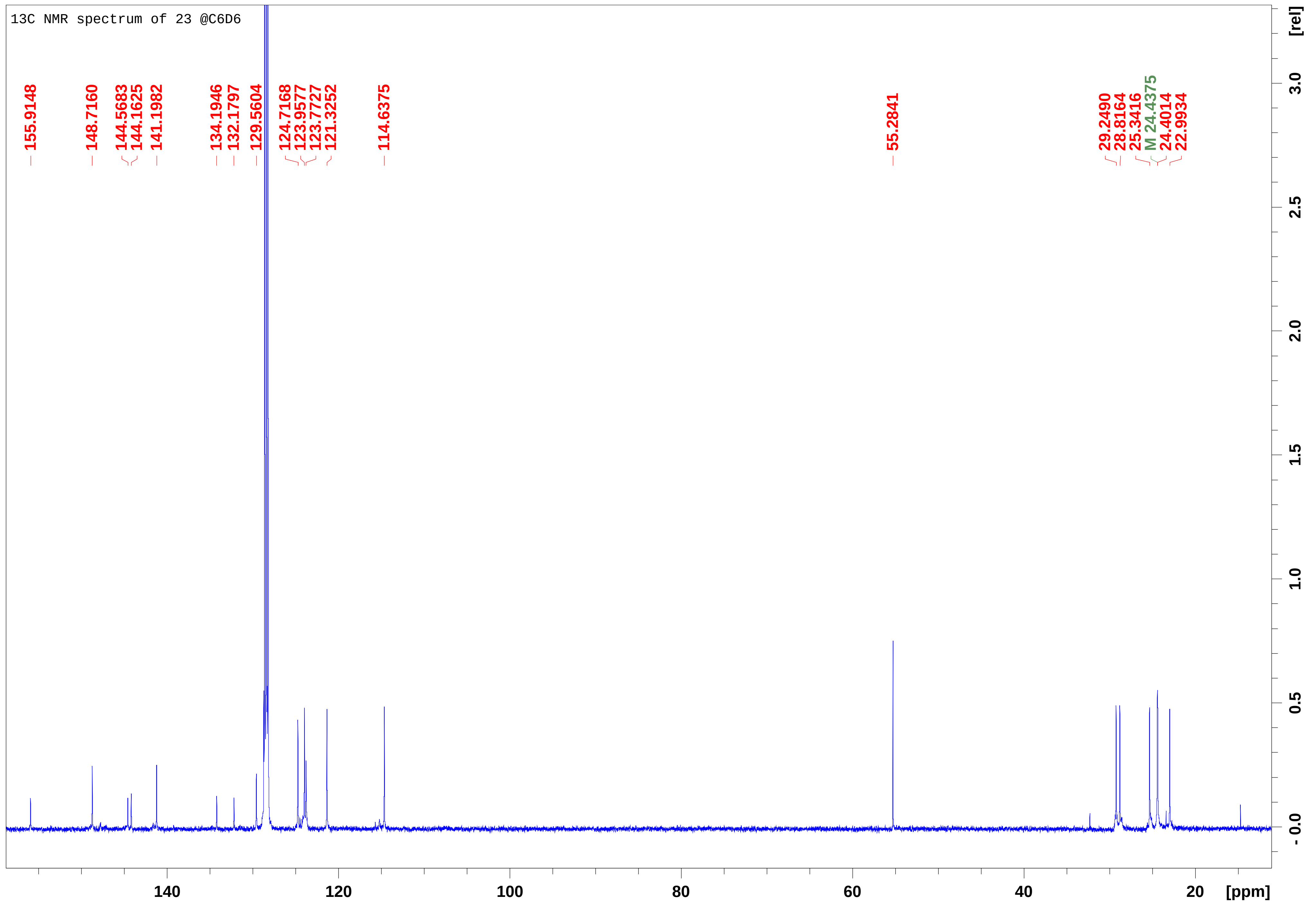

Figure S236. 13C NMR spectrum of 23 in C6D6

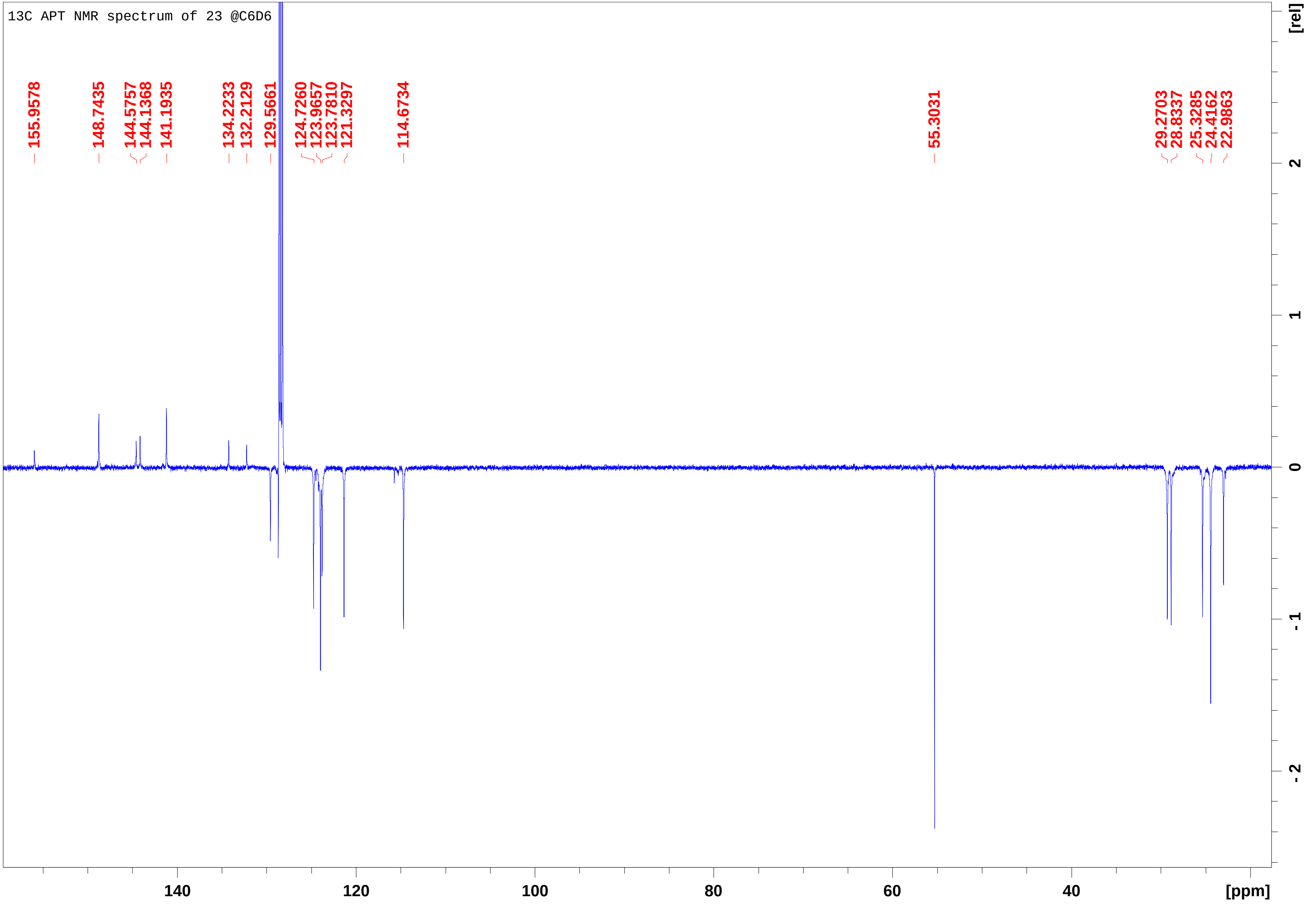

Figure S237. 13C APT NMR spectrum of 23 in C6D6

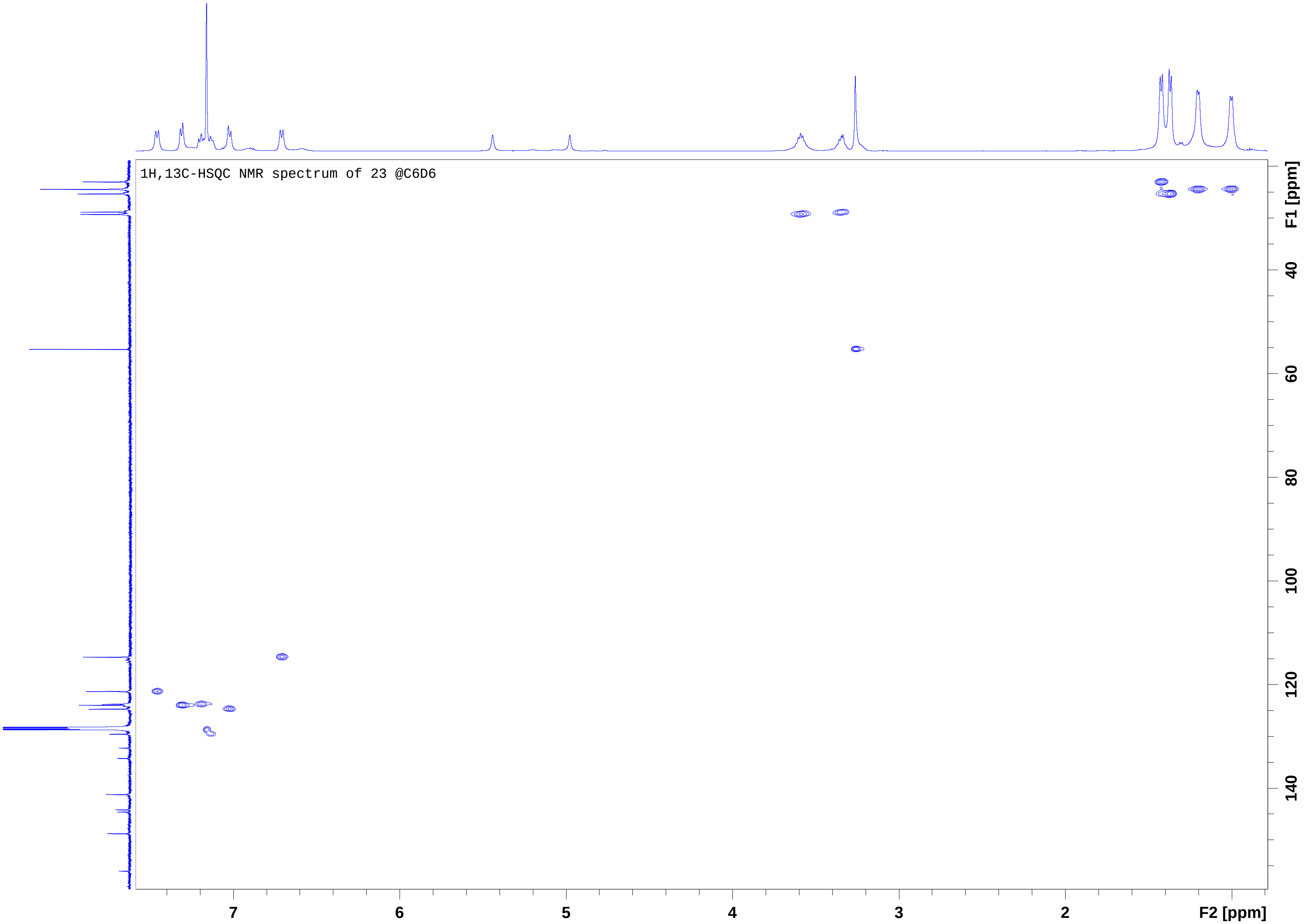

Figure S238. 1H,13C-HSQC NMR spectrum of 23 in C6D6

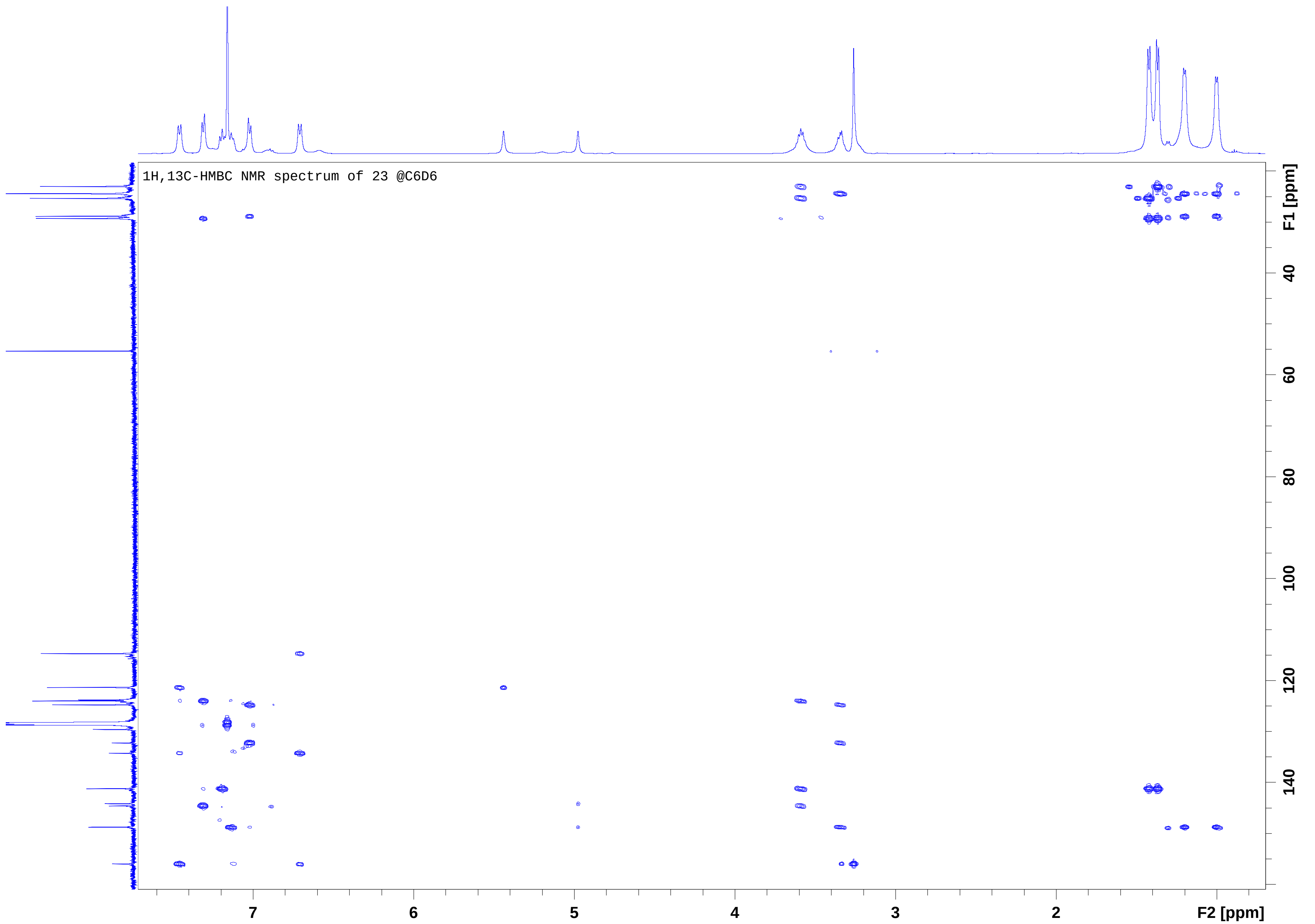

Figure S239. 1H,13C-HMBC NMR spectrum of 23 in C6D6

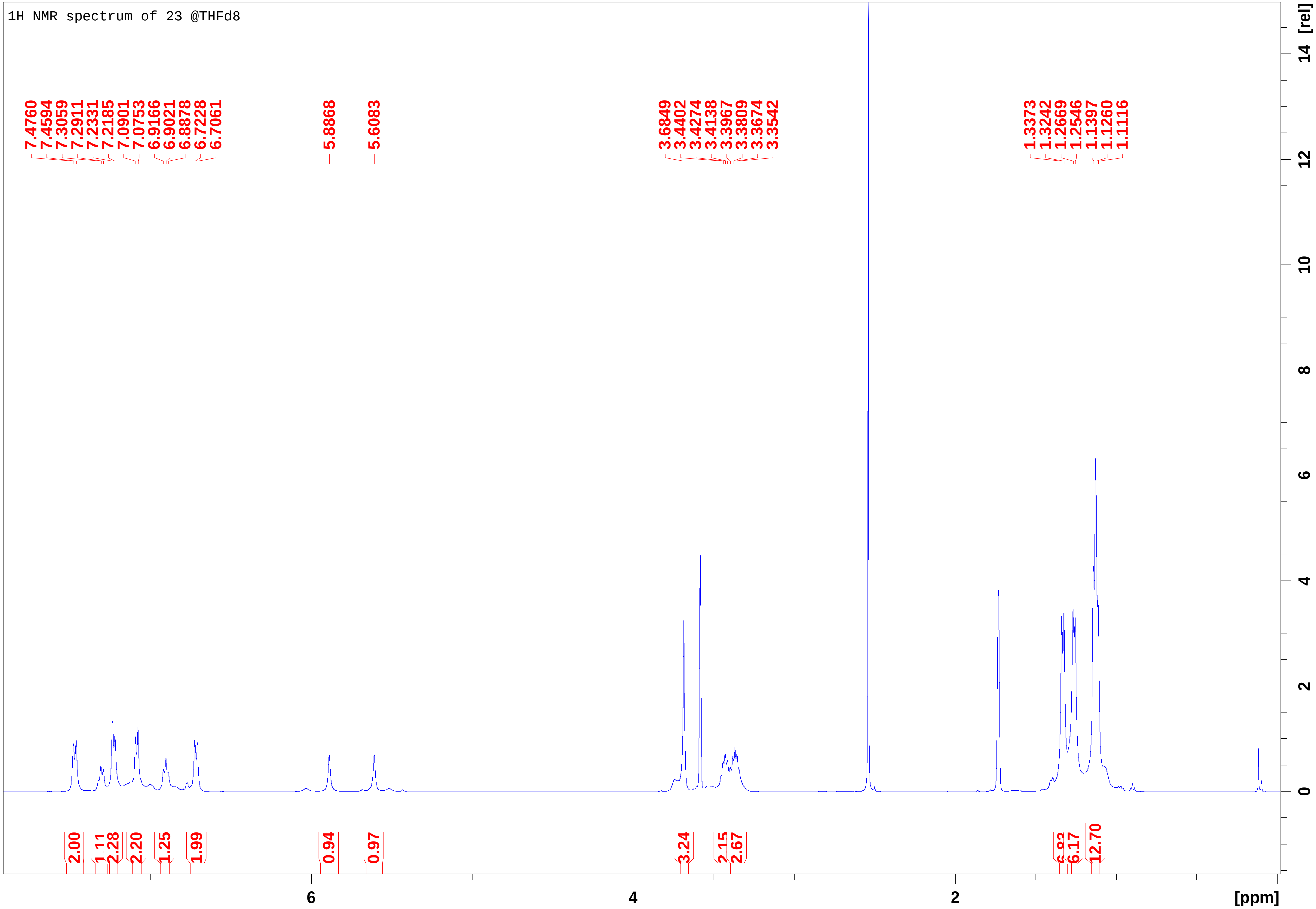

Figure S240. 1H NMR spectrum of 23 in THF-d8

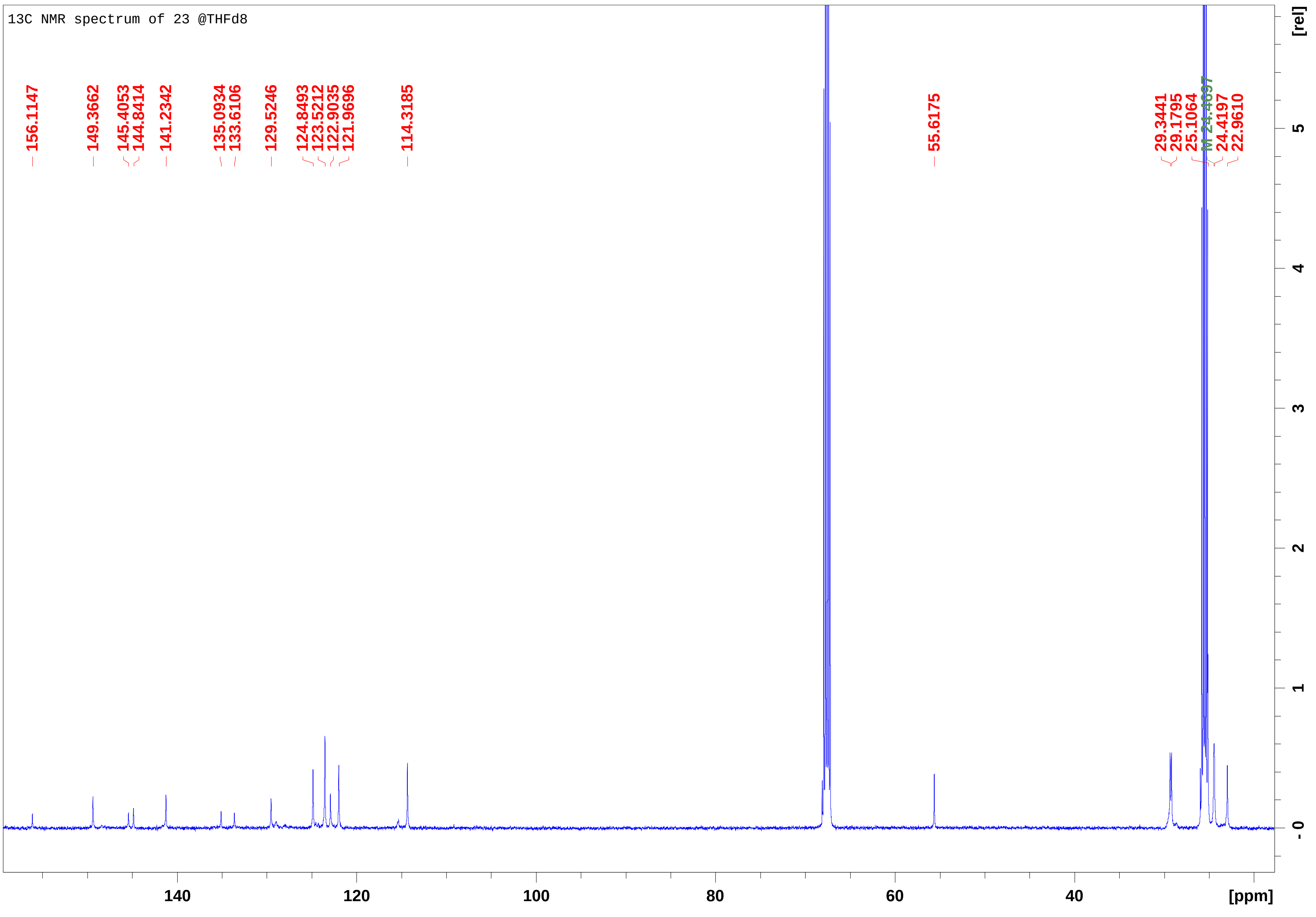

Figure S241. 13C NMR spectrum of 23 in THF-d8

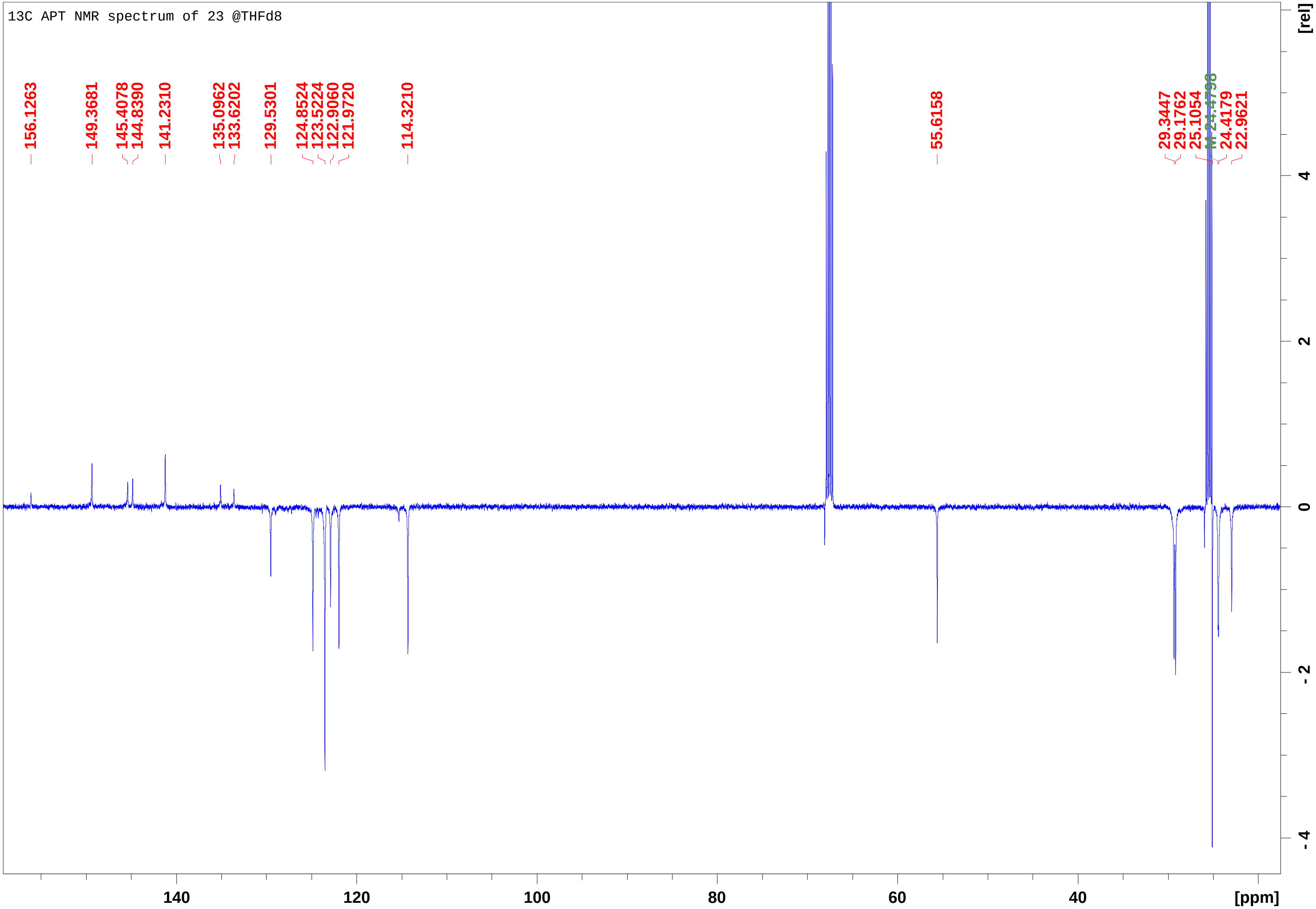

Figure S242. 13C APT NMR spectrum of 23 in THF-d8

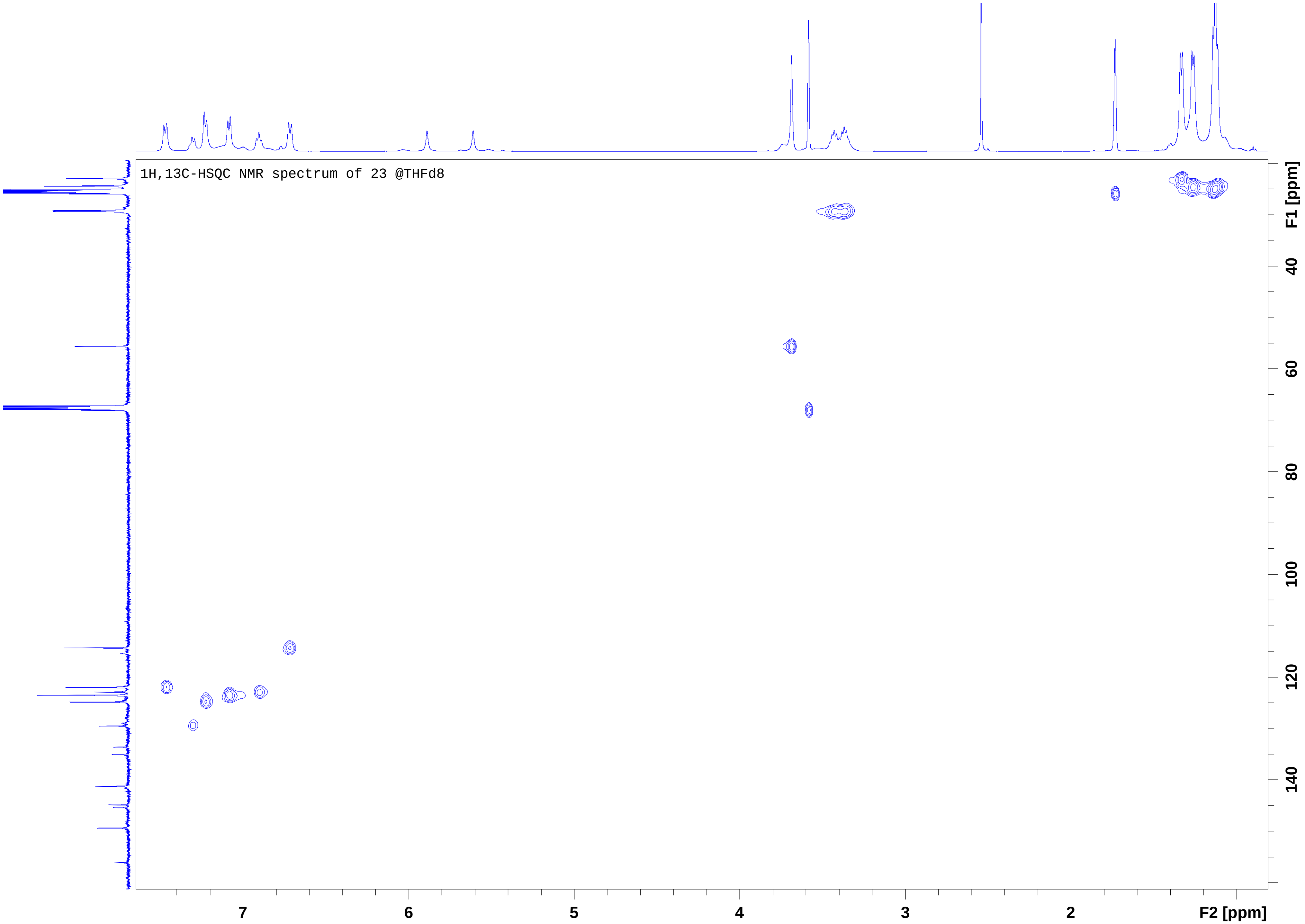

Figure S243. 1H,13C-HSQC NMR spectrum of 23 in THF-d8

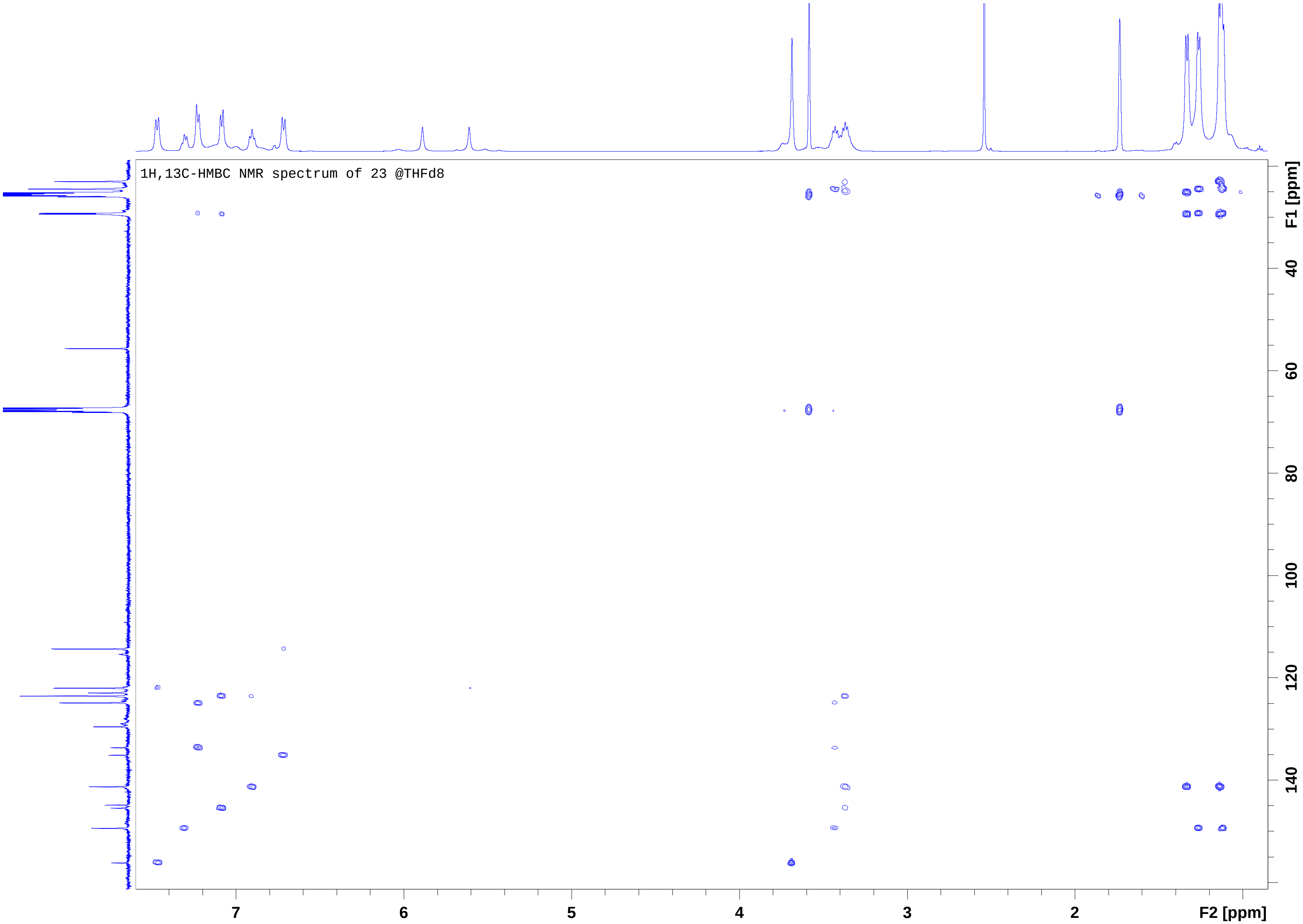

Figure S244. 1H,13C-HMBC NMR spectrum of 23 in THF-d8

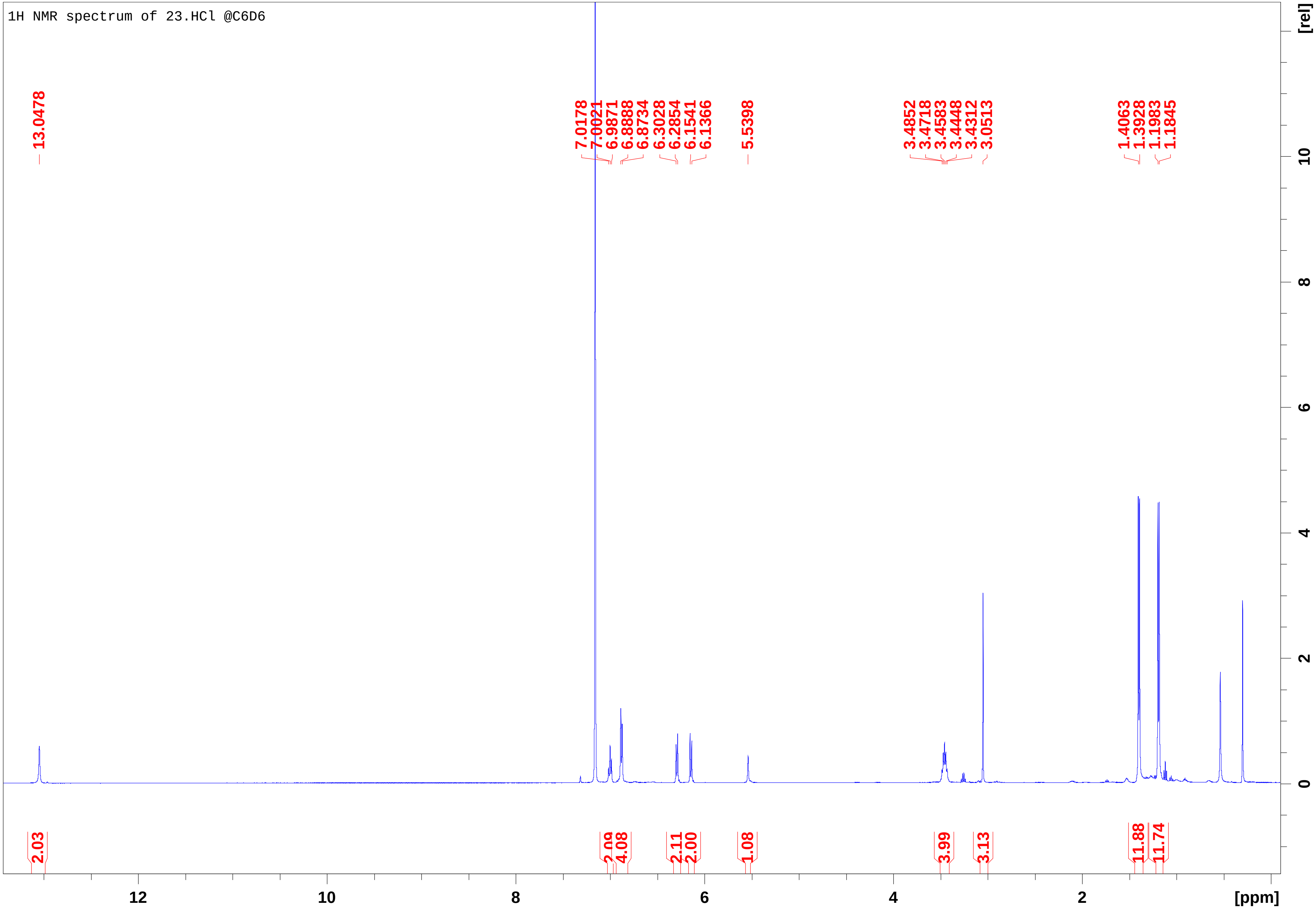

Figure S245. 1H NMR spectrum of 23.HCl in C6D6

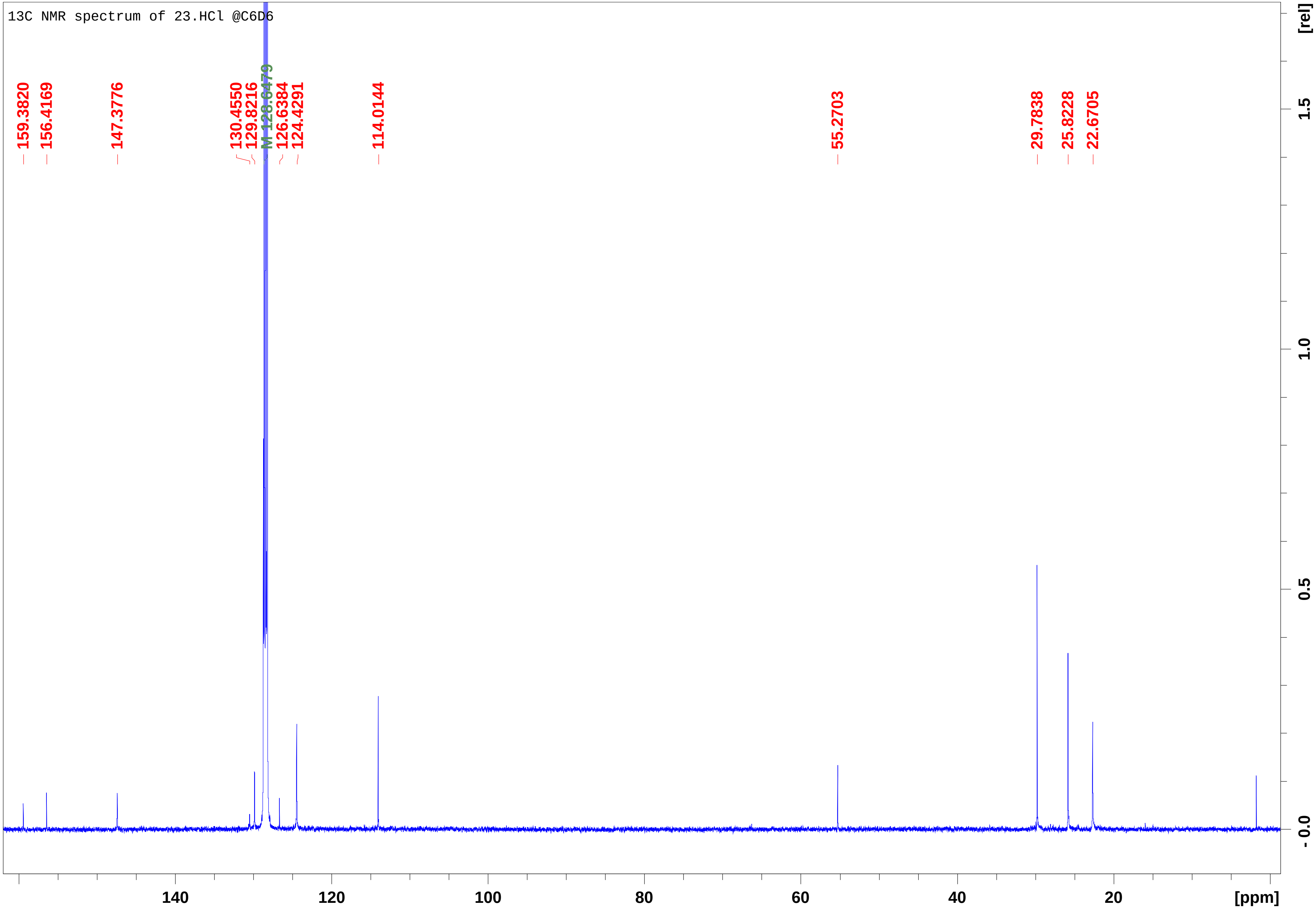

Figure S246. 13C NMR spectrum of 23.HCl in C6D6

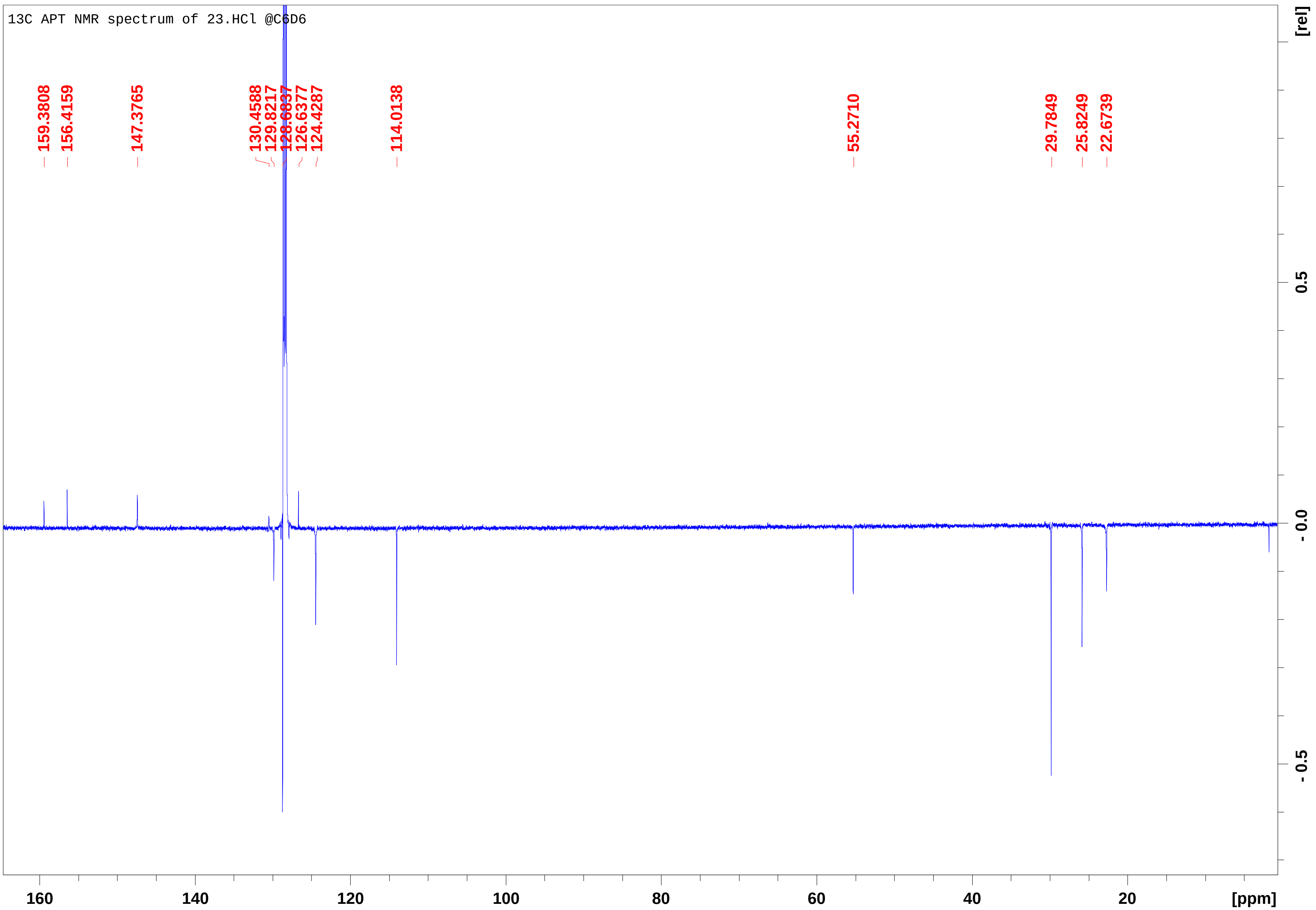

Figure S247. 13C APT NMR spectrum of 23.HCl in C6D6

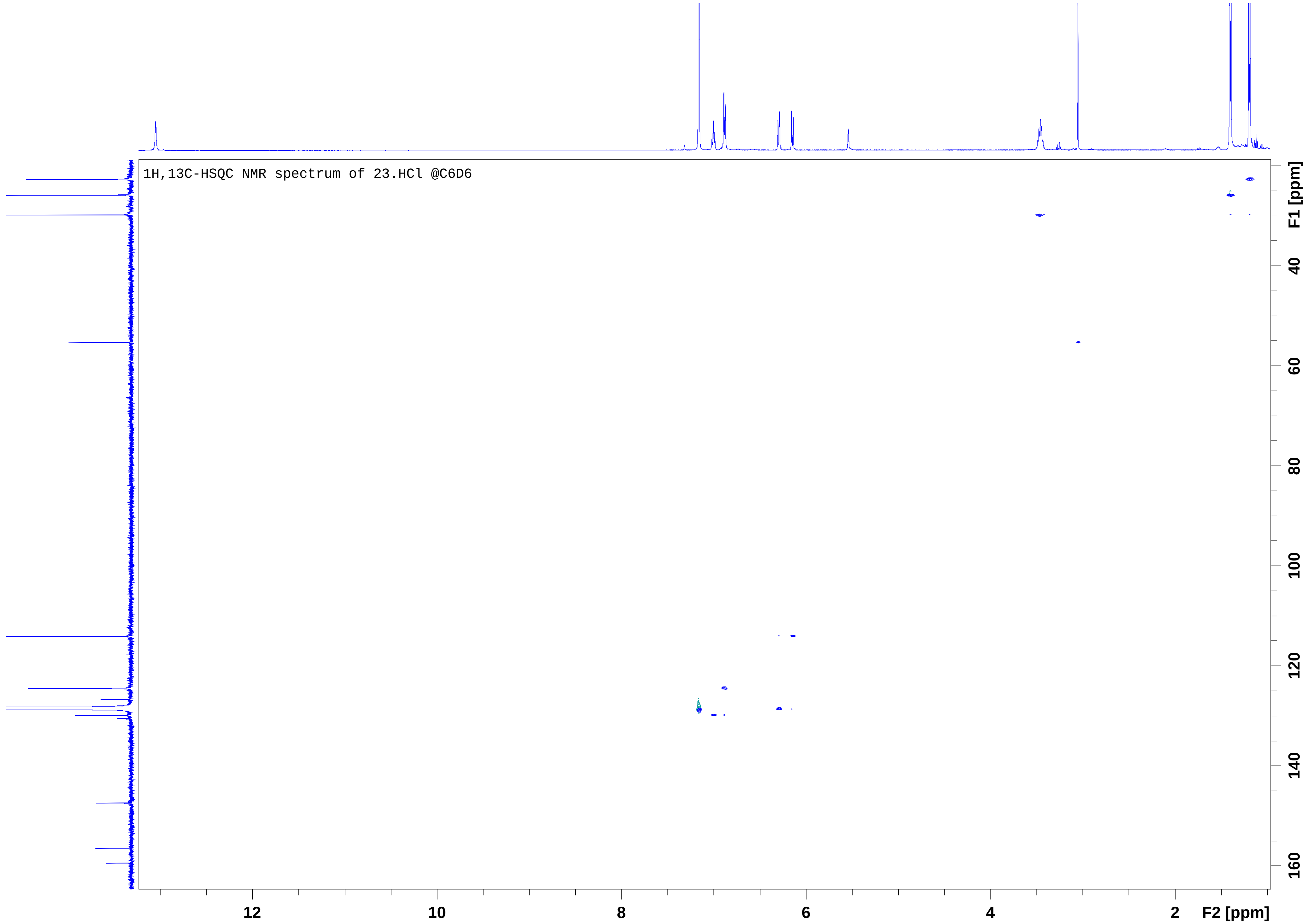

Figure S248. 1H,13C-HSQC NMR spectrum of 23.HCl in C6D6

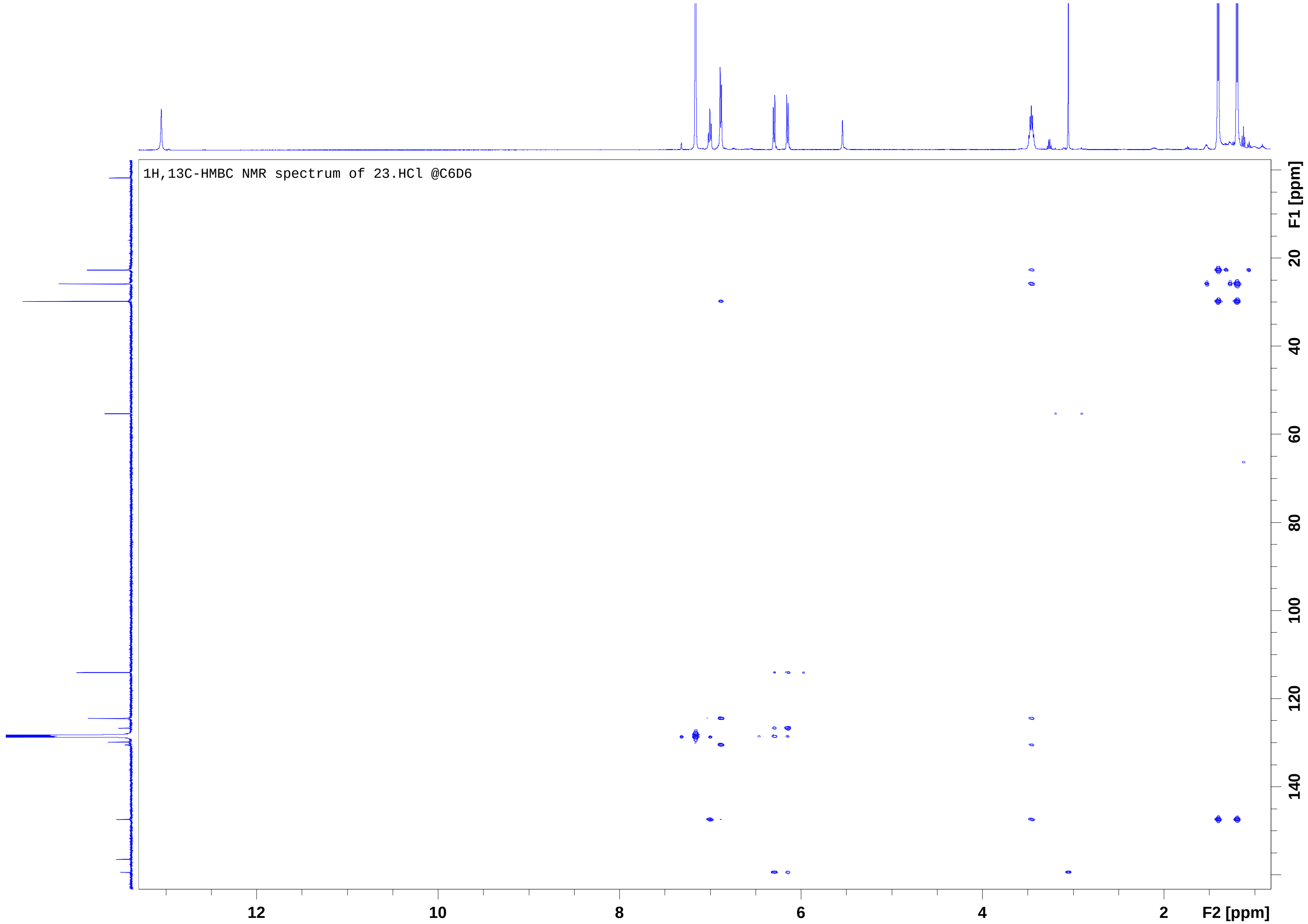

Figure S249. 1H,13C-HMBC NMR spectrum of 23.HCl in C6D6

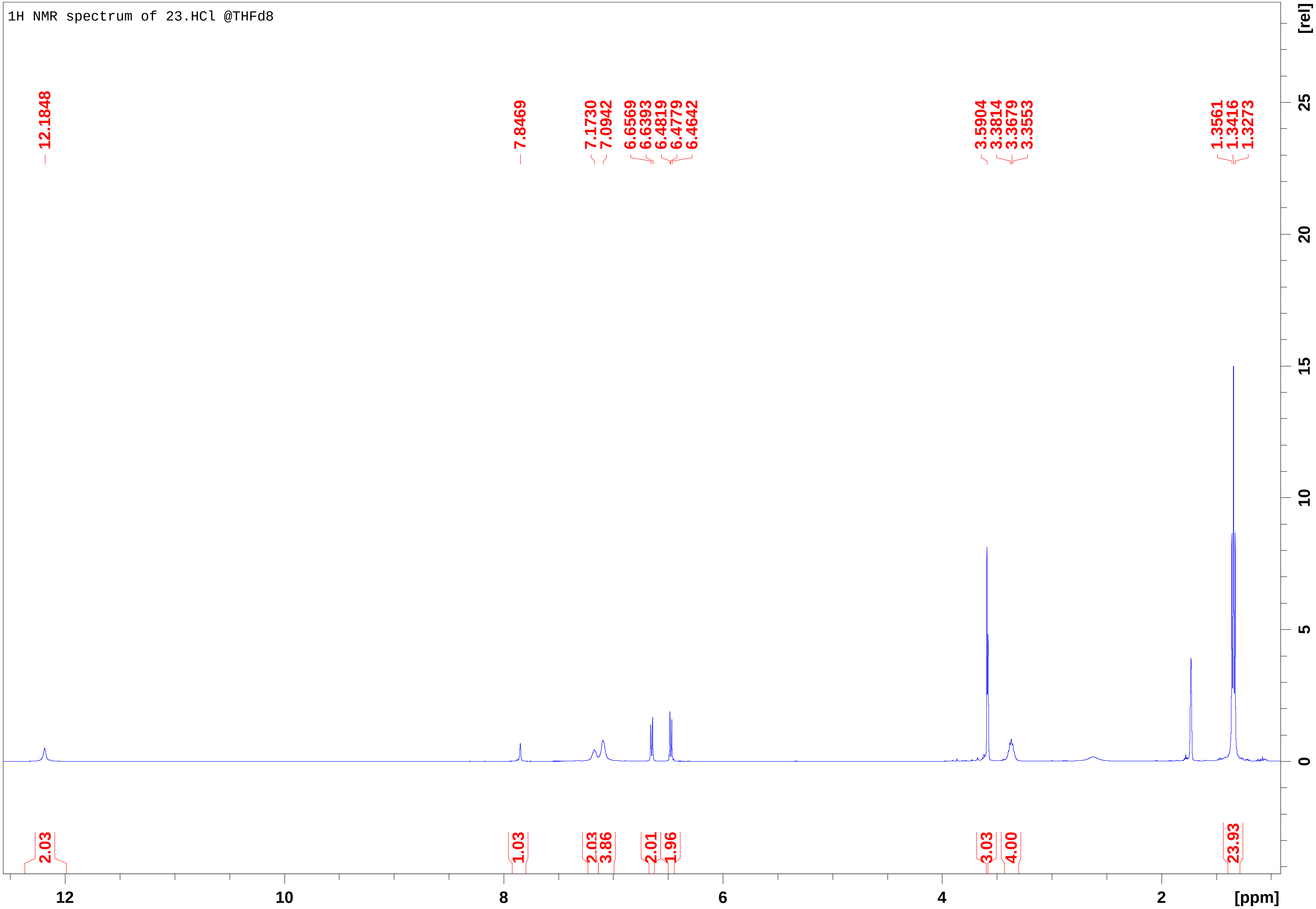

Figure S250. 1H NMR spectrum of 23.HCl in THF-d8

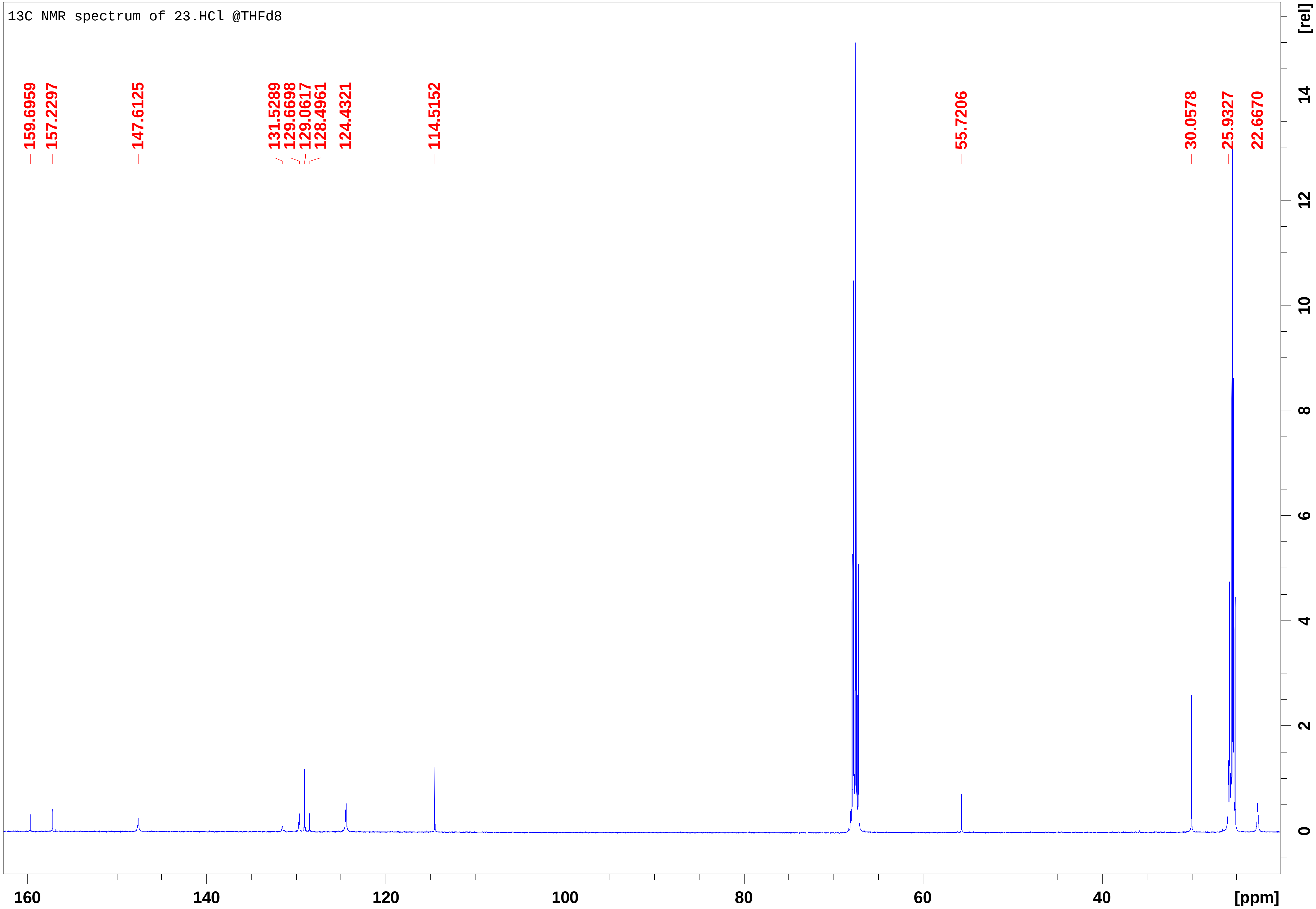

Figure S251. 13C NMR spectrum of 23.HCl in THF-d8

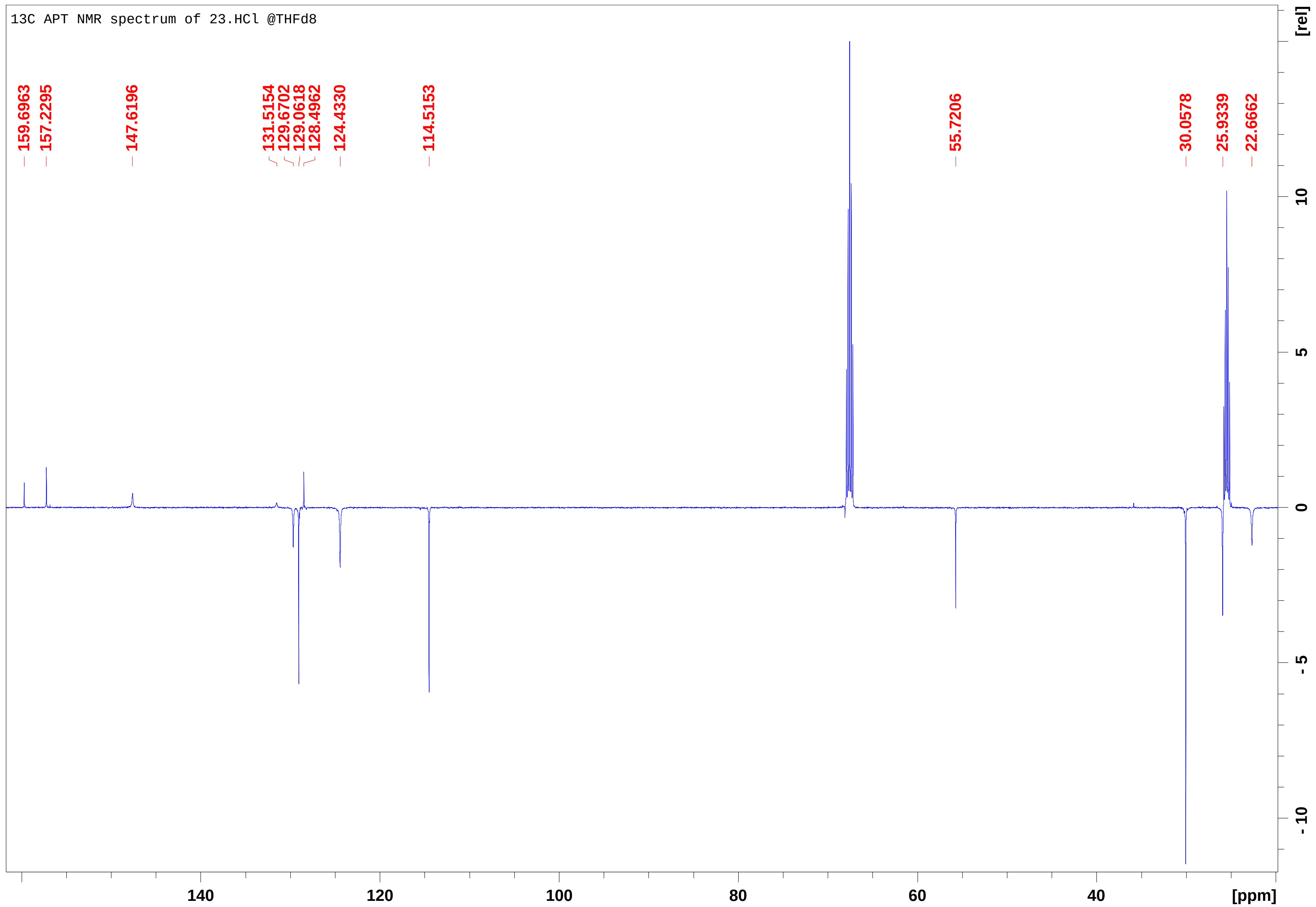

Figure S252. 13C APT NMR spectrum of 23.HCl in THF-d8

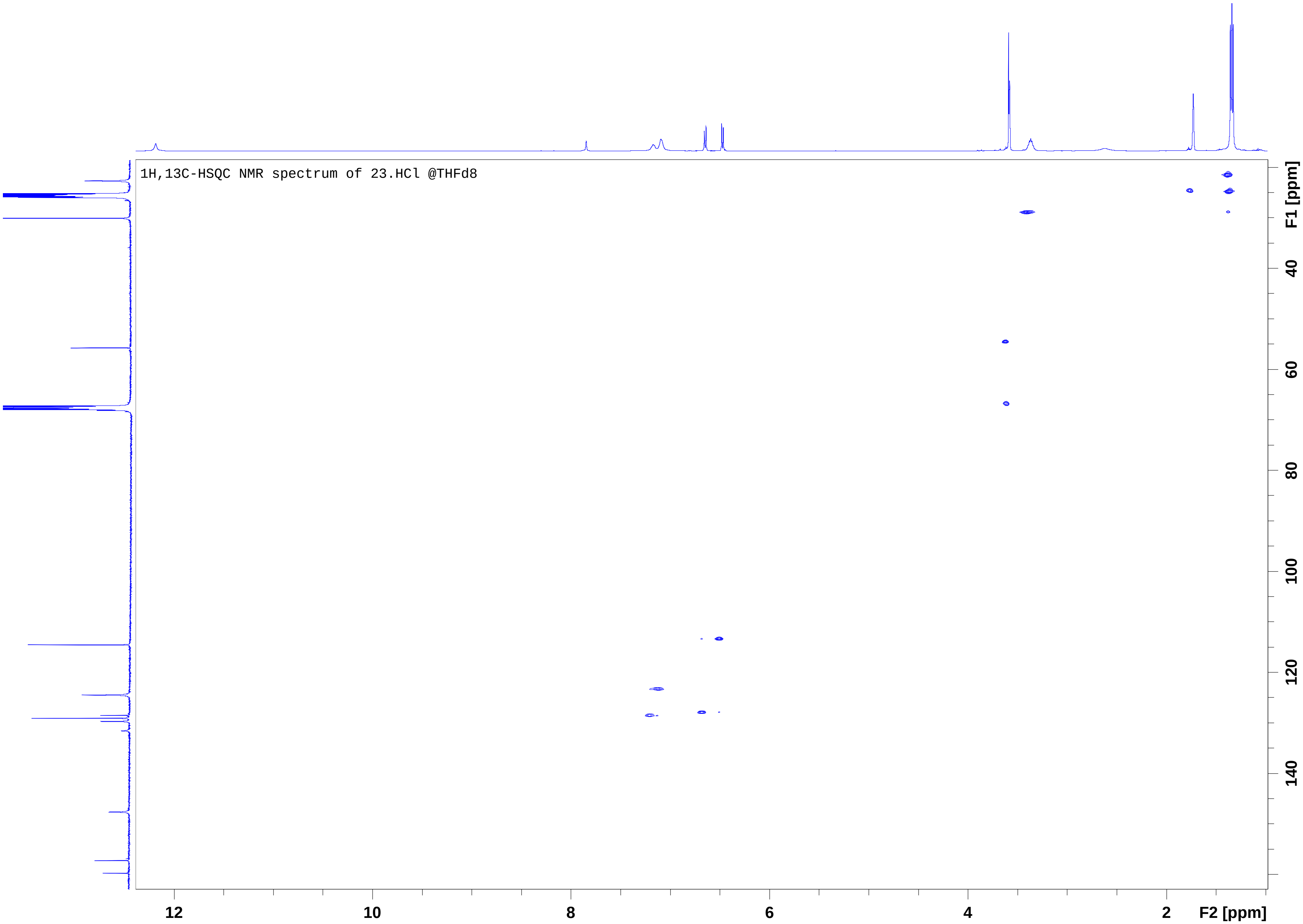

Figure S253. 1H,13C-HSQC NMR spectrum of 23.HCl in THF-d8

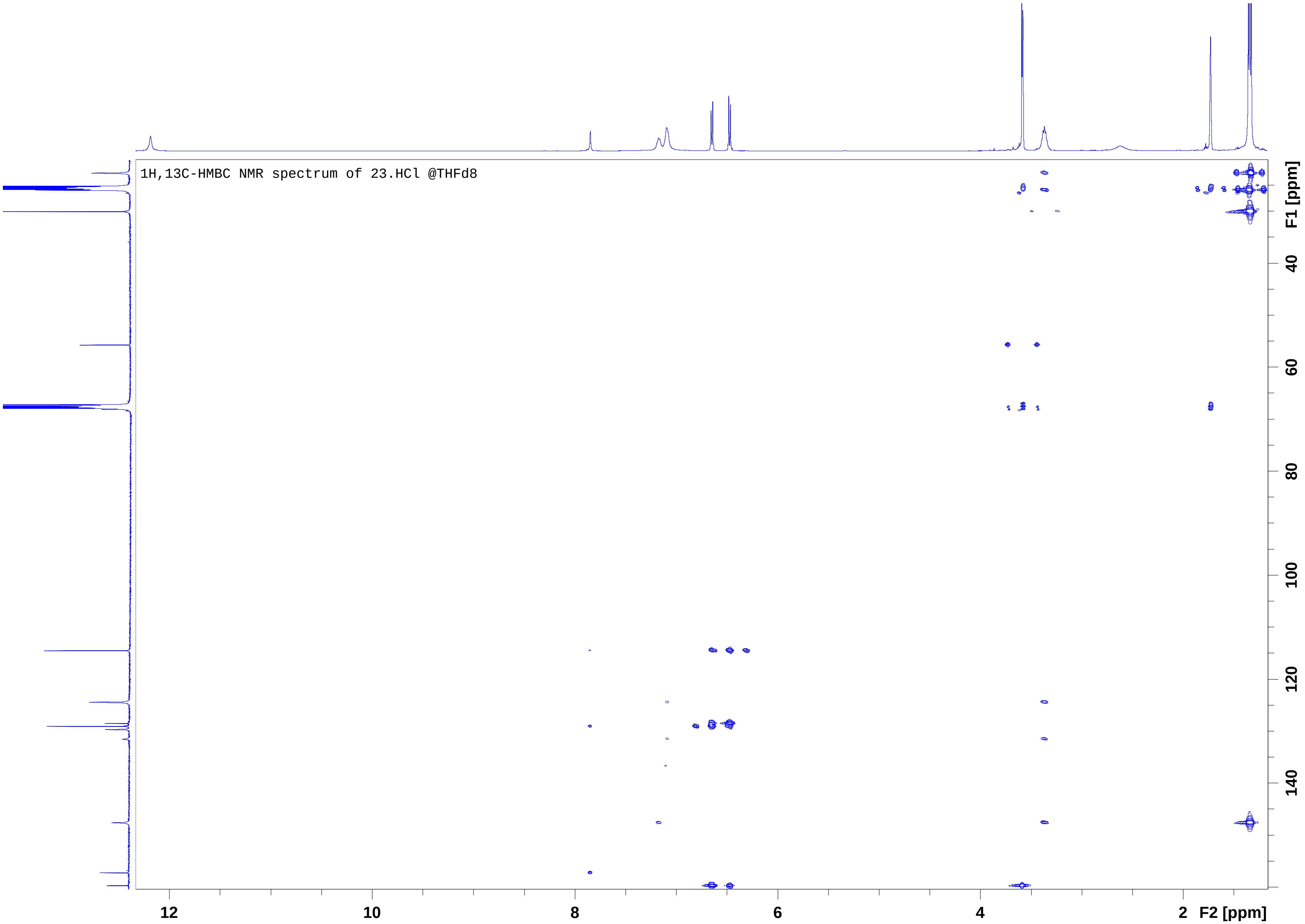

Figure S254. 1H,13C-HMBC NMR spectrum of 23.HCl in THF-d8

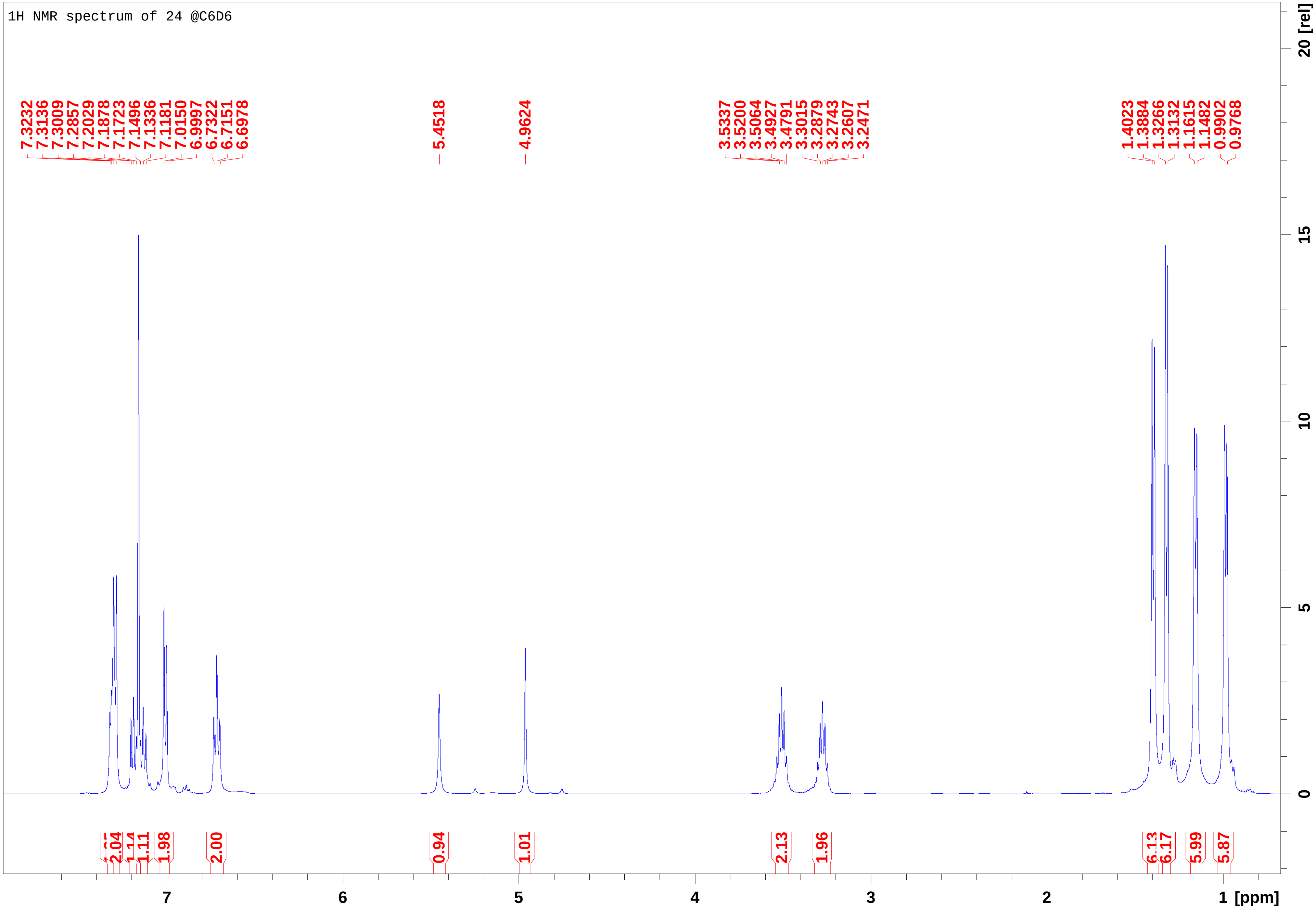

Figure S255. 1H NMR spectrum of 24 in C6D6

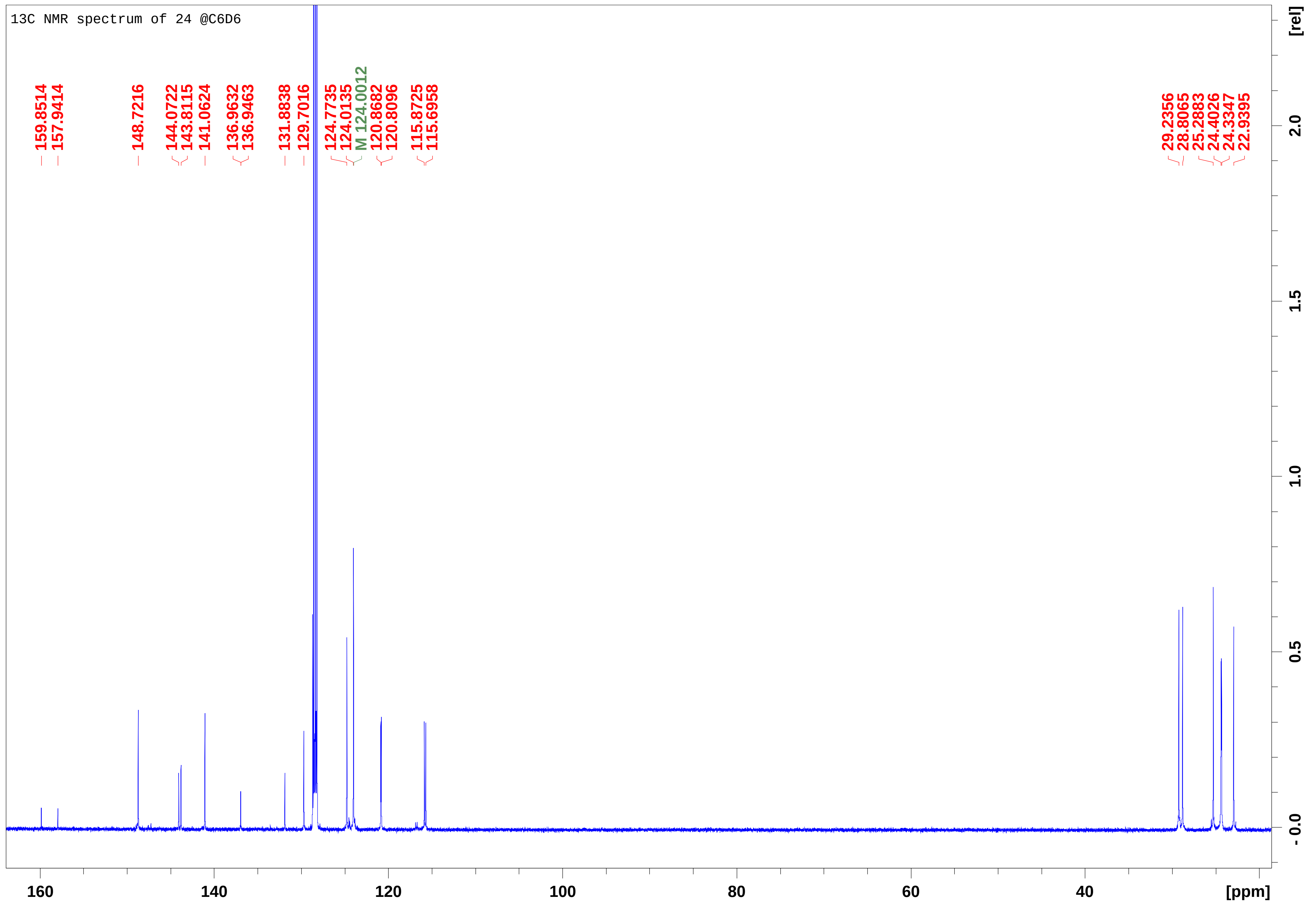

Figure S256. 13C NMR spectrum of 24 in C6D6

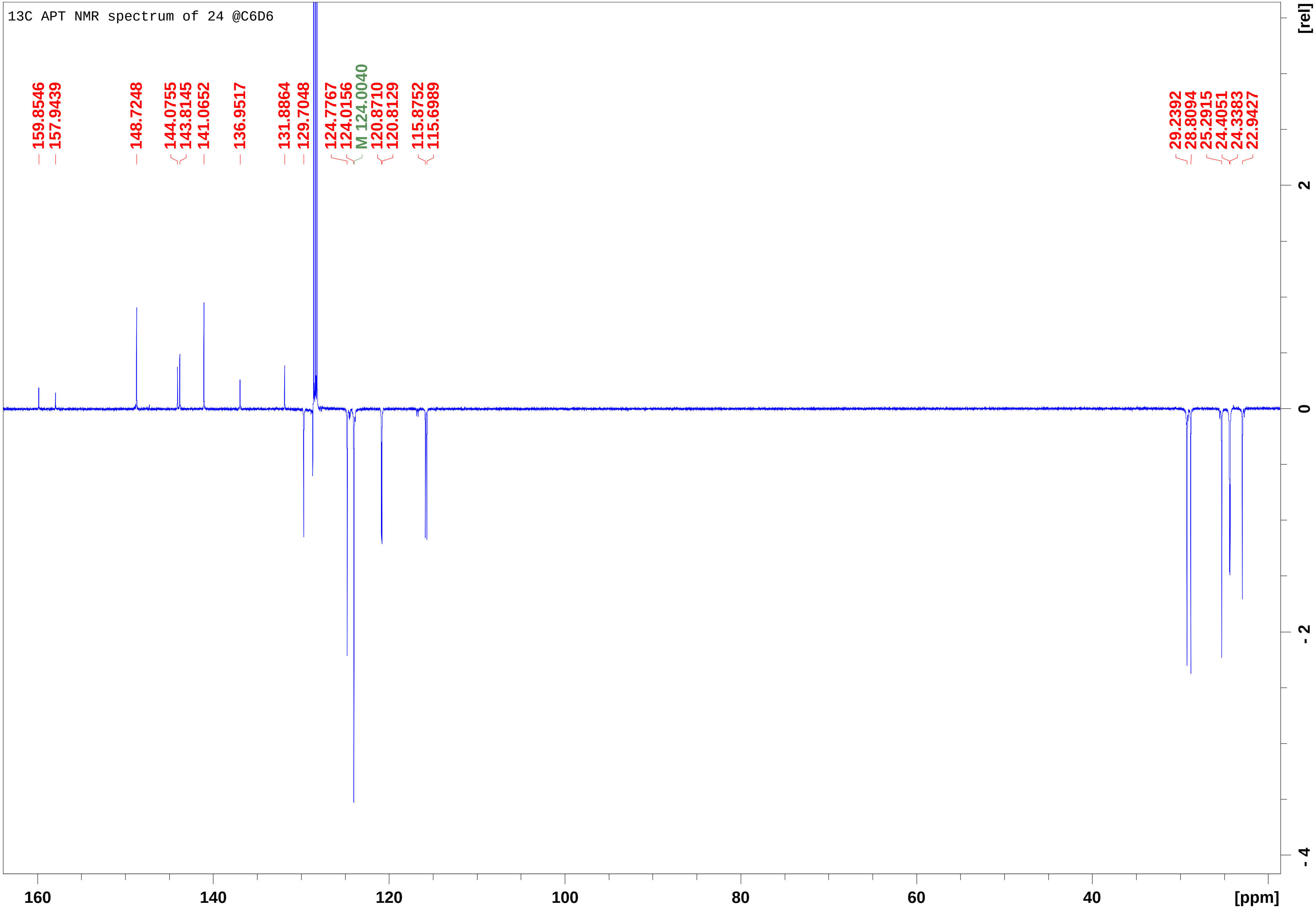

Figure S257. 13C APT NMR spectrum of 24 in C6D6

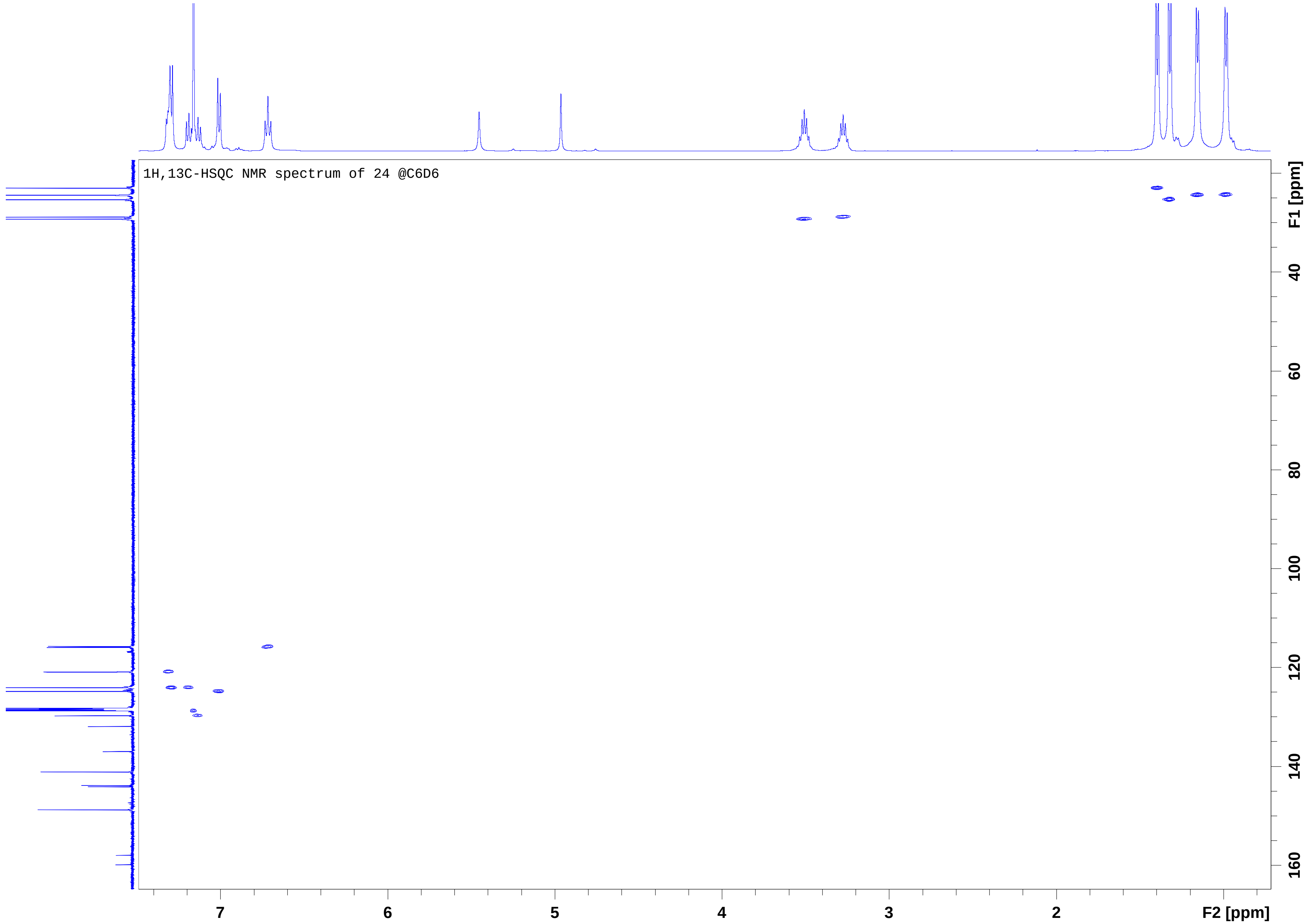

Figure S258. 1H,13C-HSQC NMR spectrum of 24 in C6D6

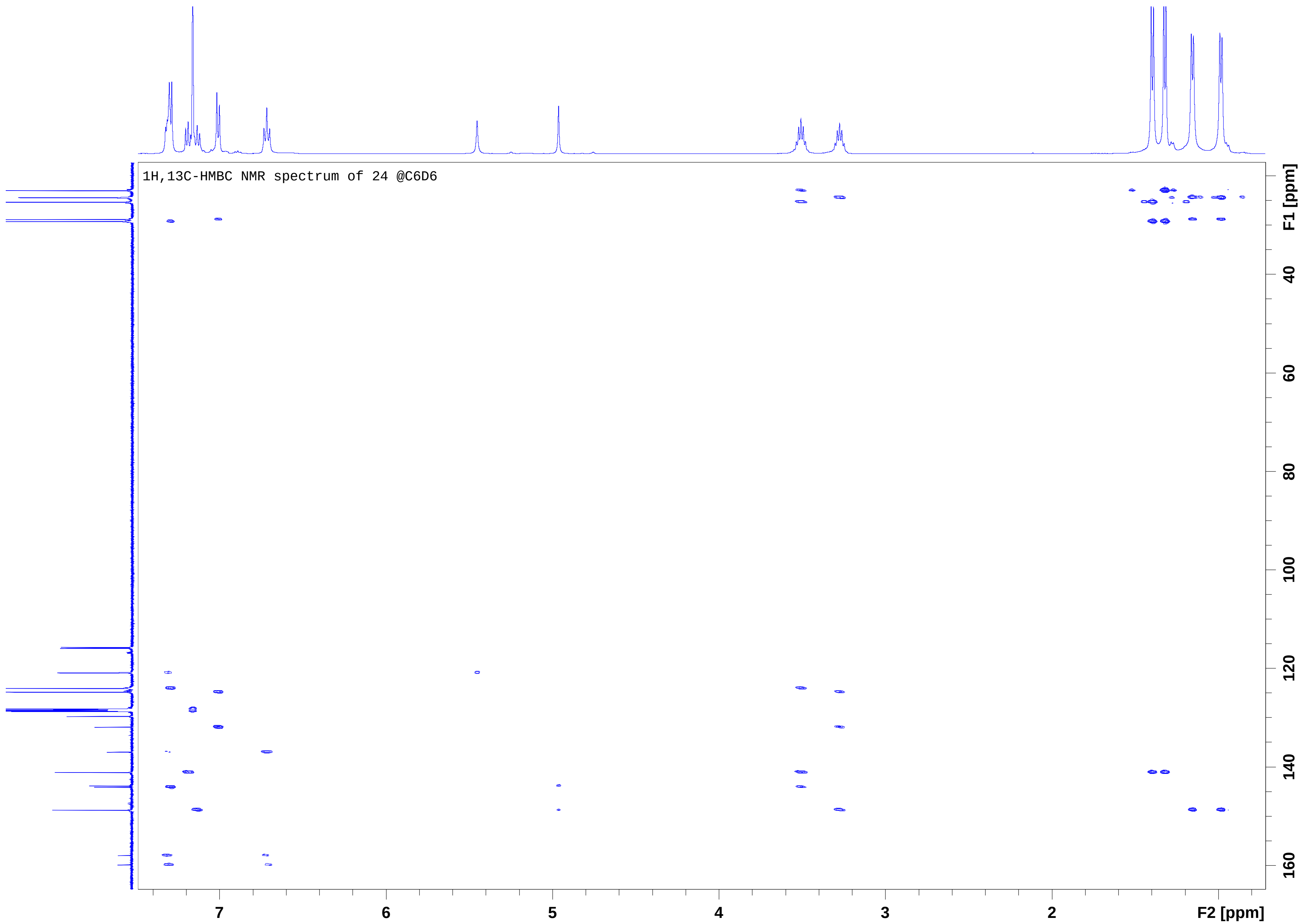

Figure S259. 1H,13C-HMBC NMR spectrum of 24 in C6D6

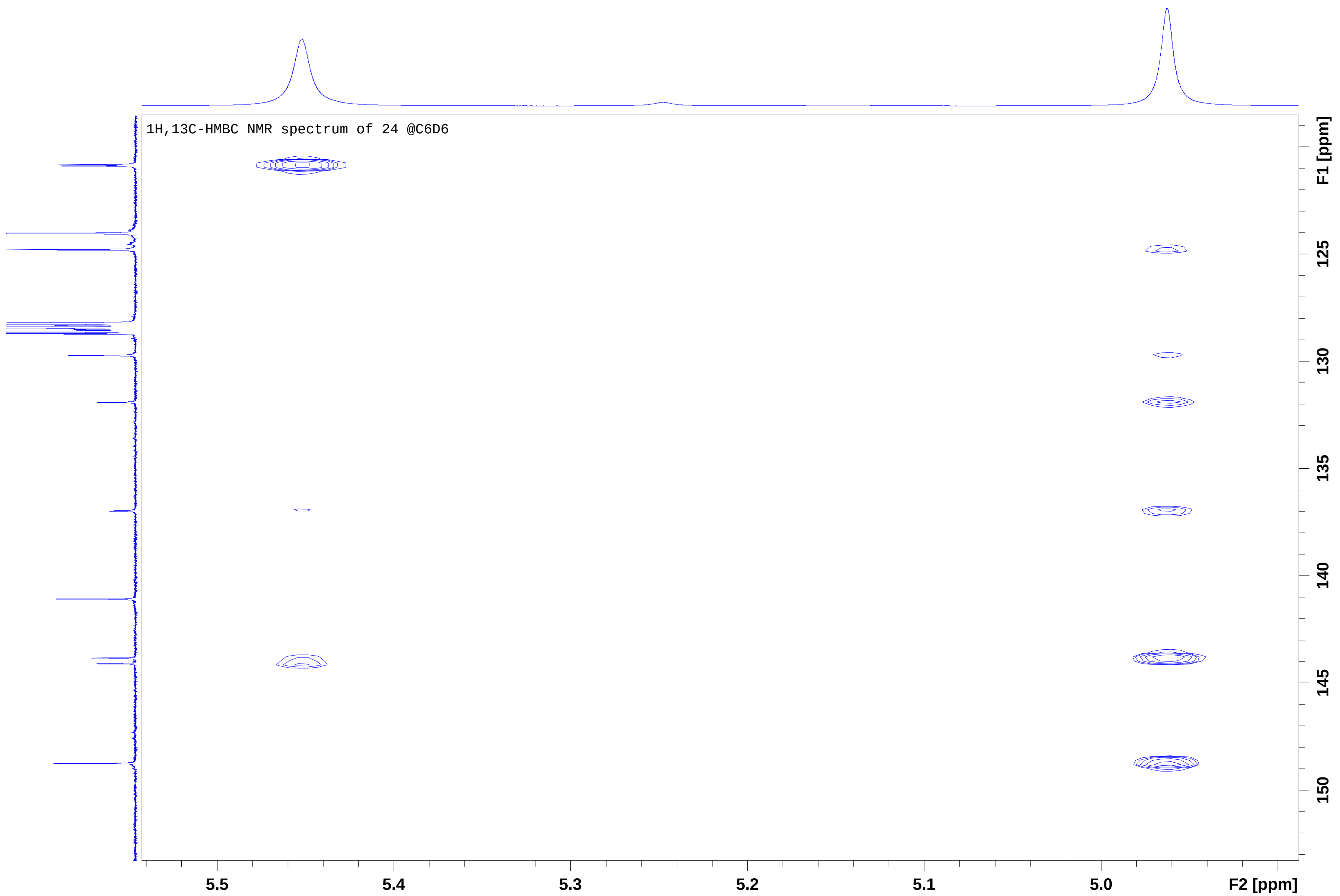

Figure S260. Detail of 1H,13C-HMBC NMR spectrum of 24 in C6D6

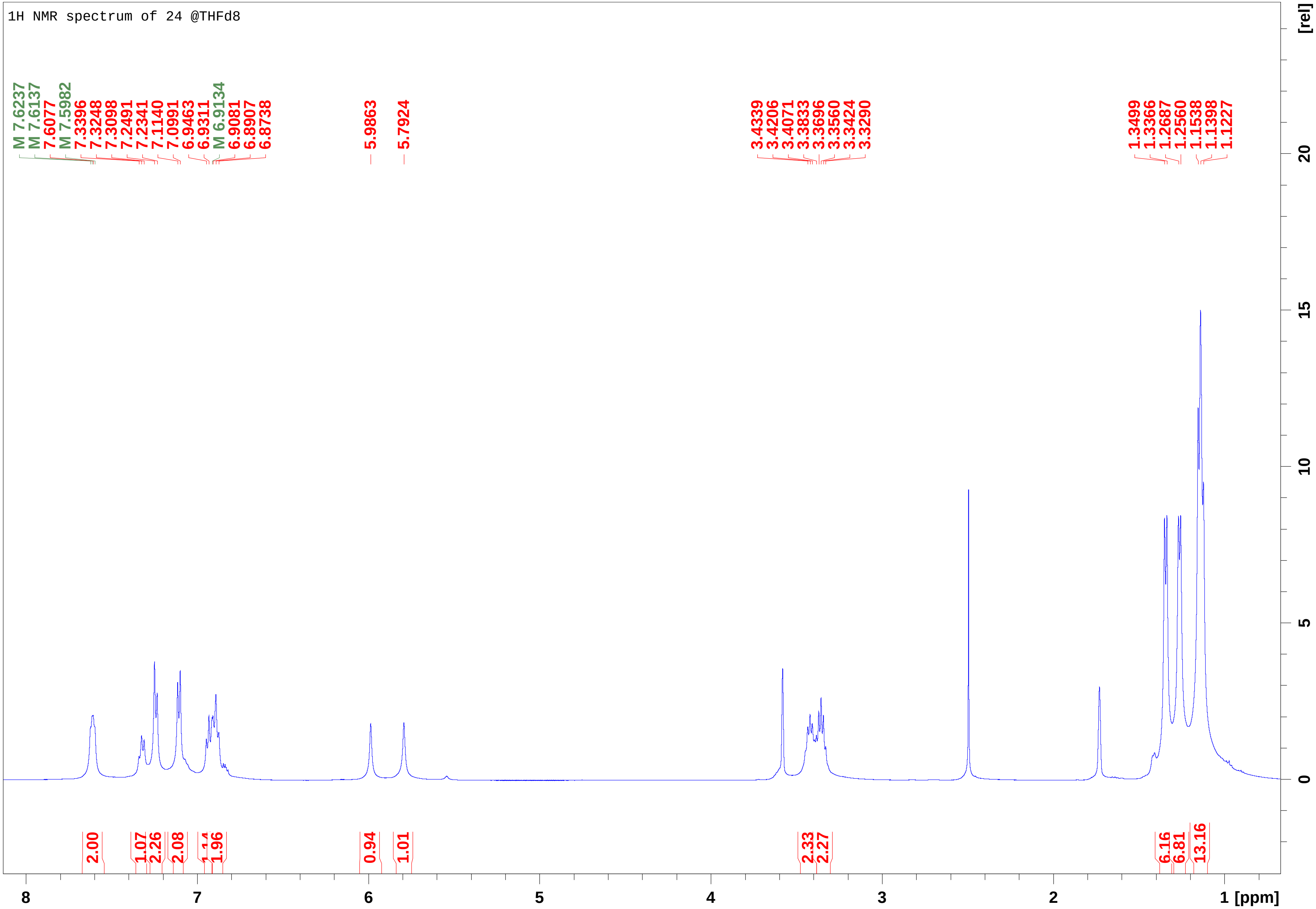

Figure S261. 1H NMR spectrum of 24 in THF-d8

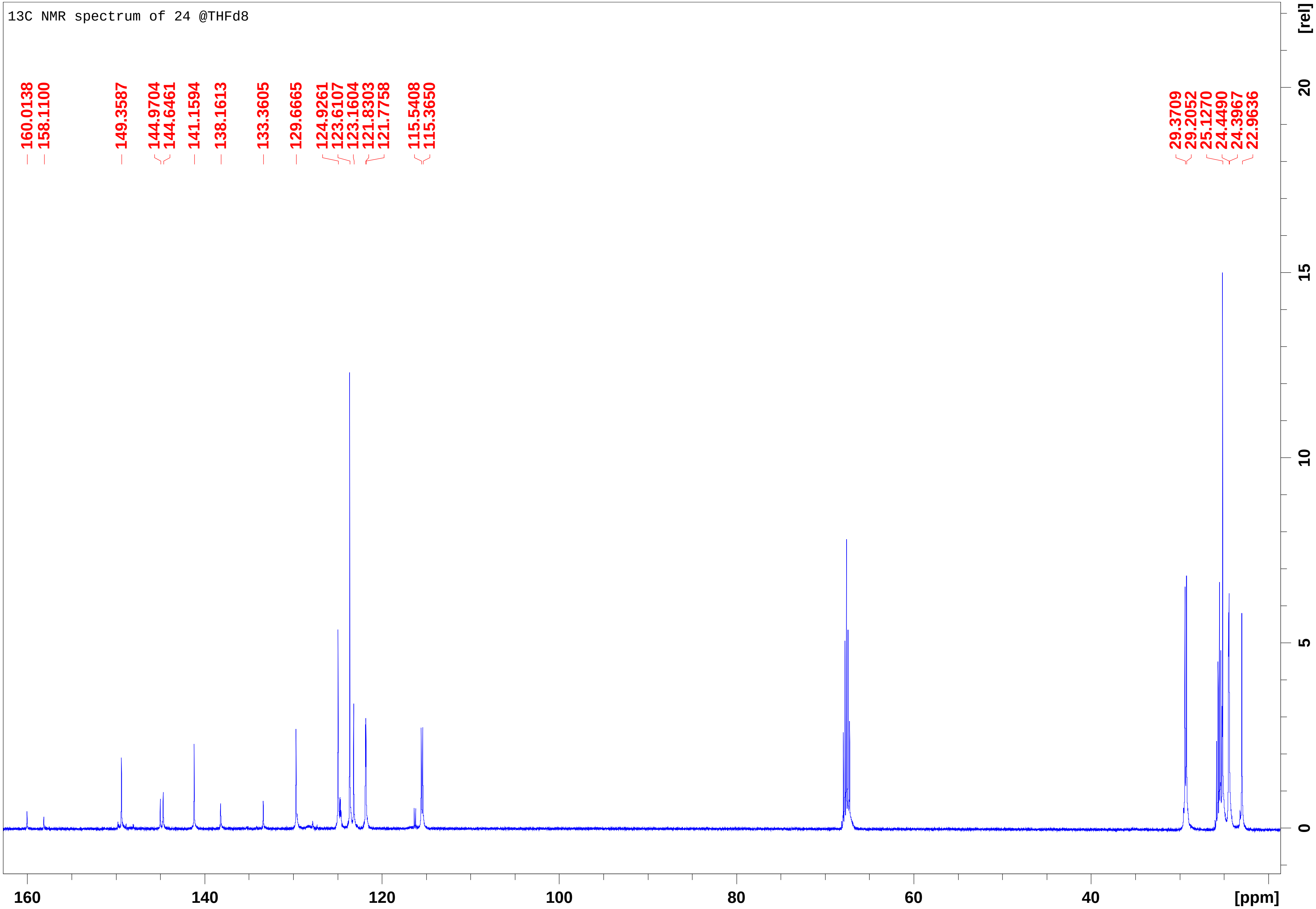

Figure S262. 13C NMR spectrum of 24 in THF-d8

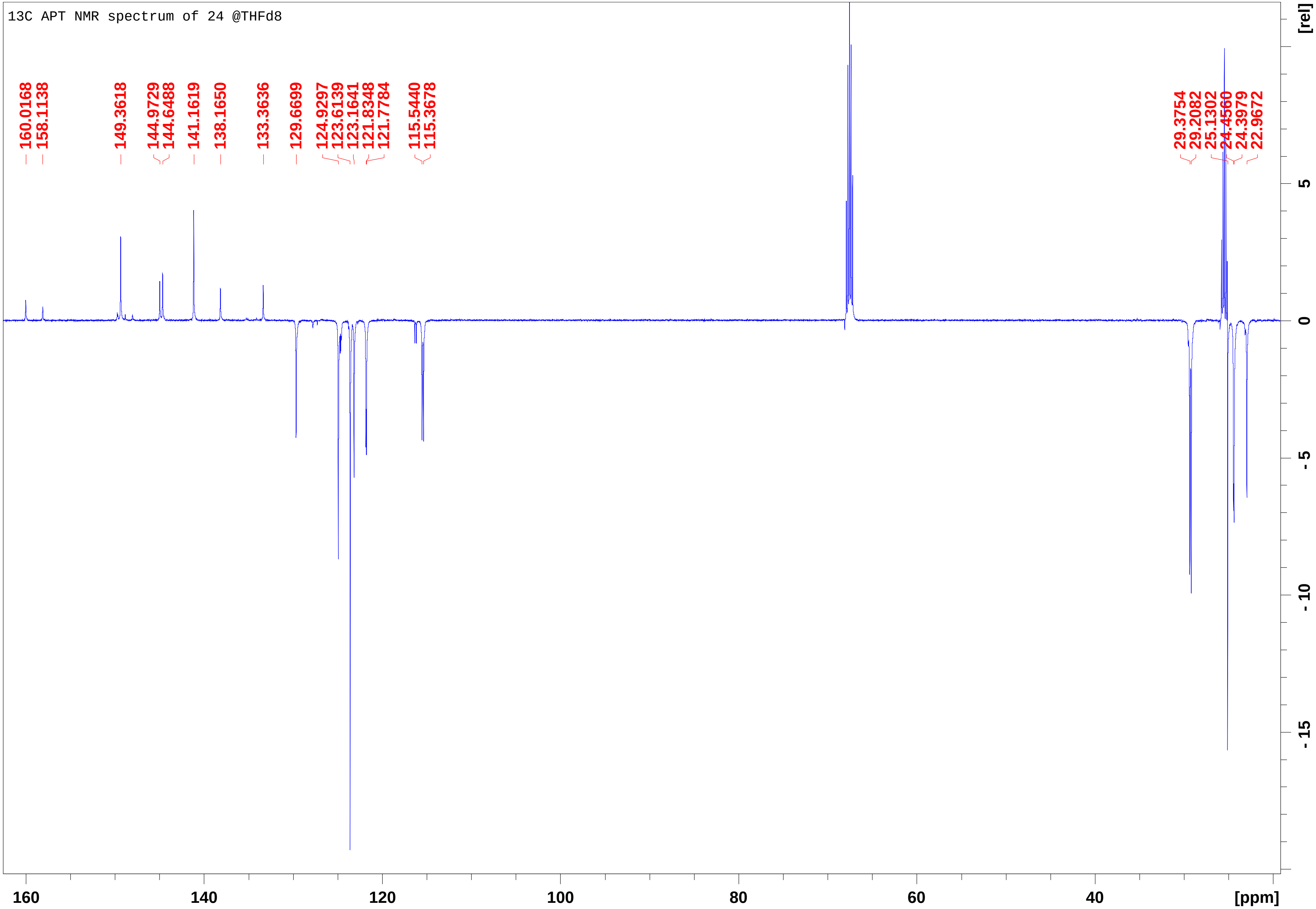

Figure S263. 13C APT NMR spectrum of 24 in THF-d8

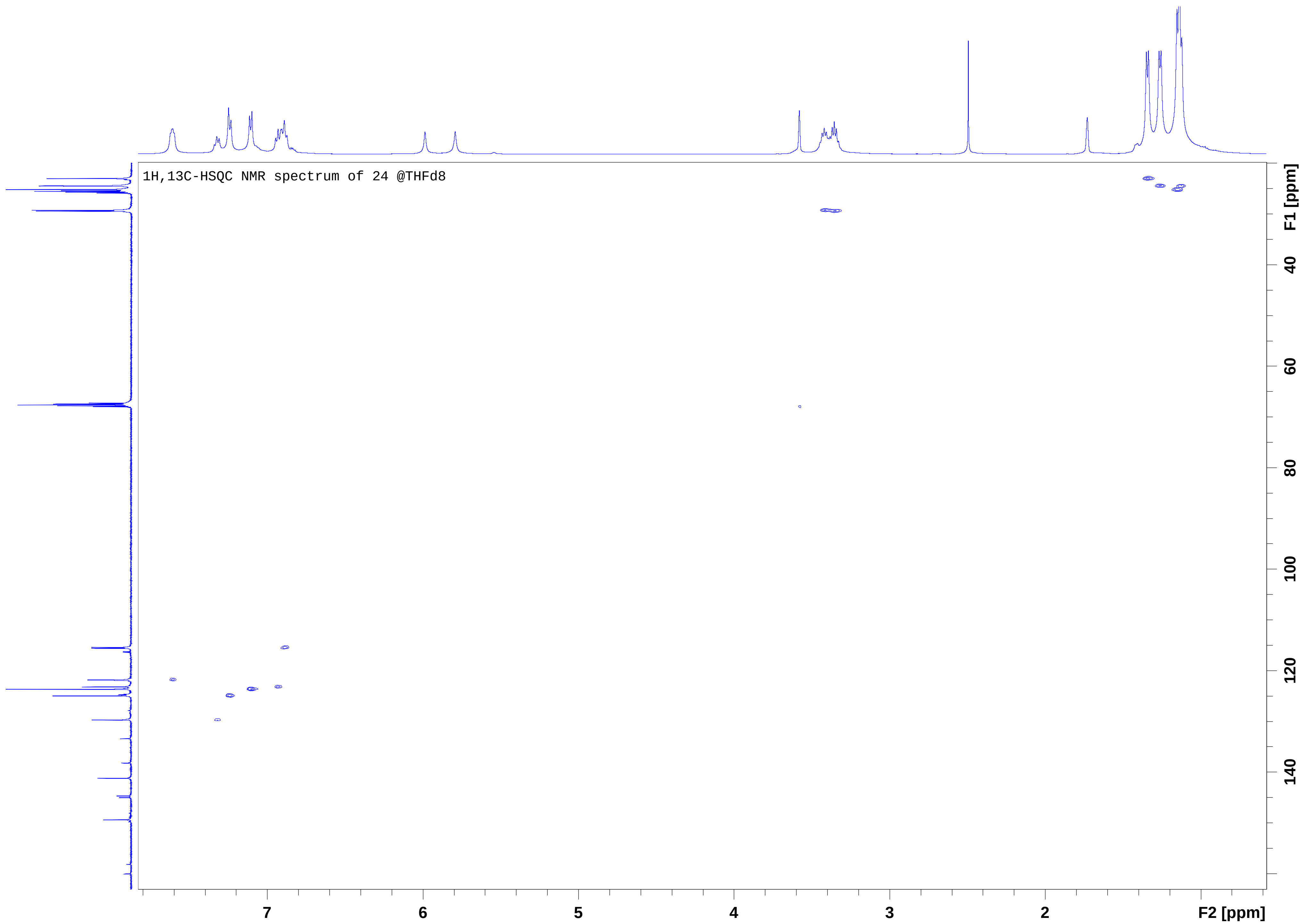

Figure S264. 1H,13C-HSQC NMR spectrum of 24 in THF-d8

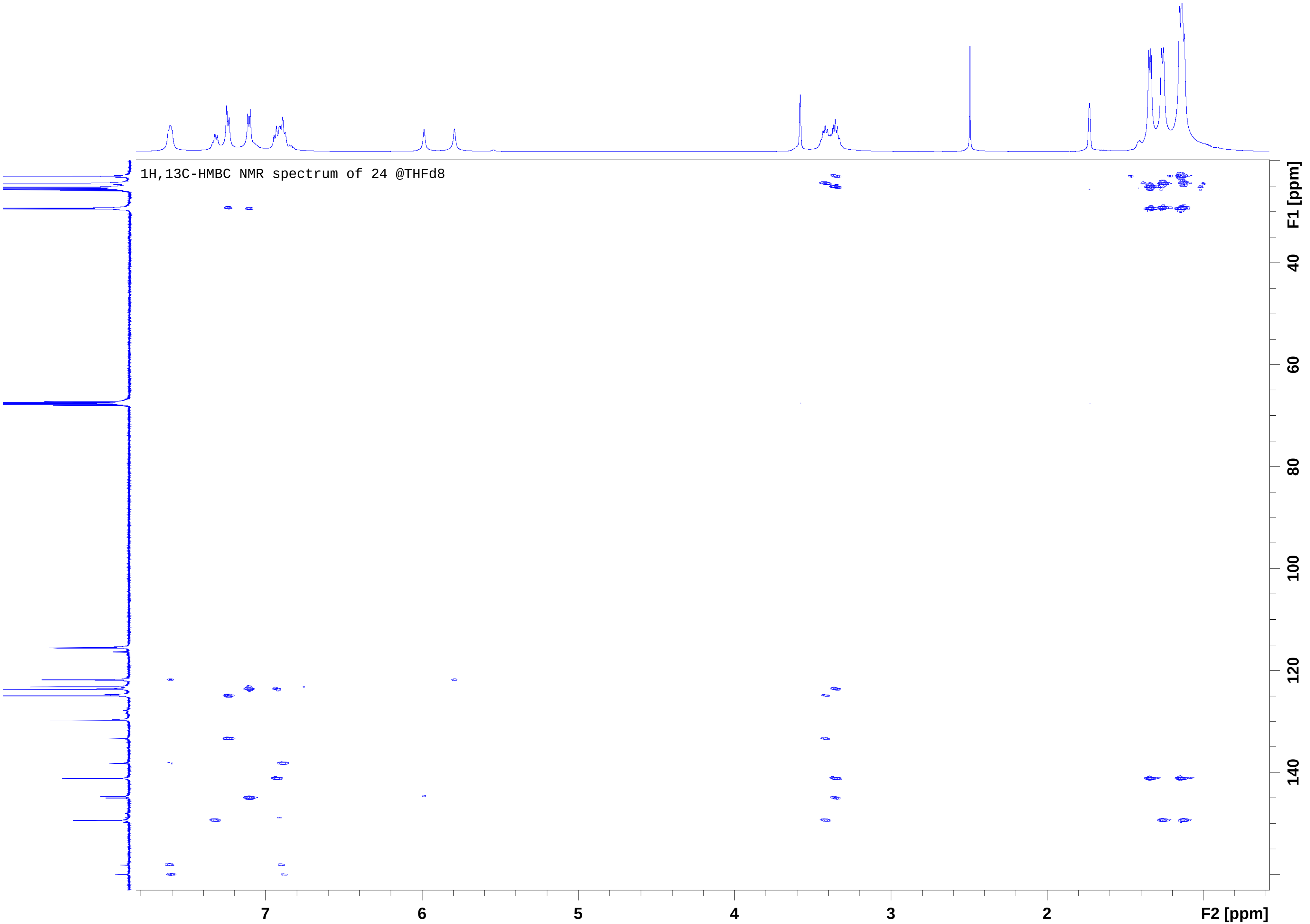

Figure S265. 1H,13C-HMBC NMR spectrum of 24 in THF-d8

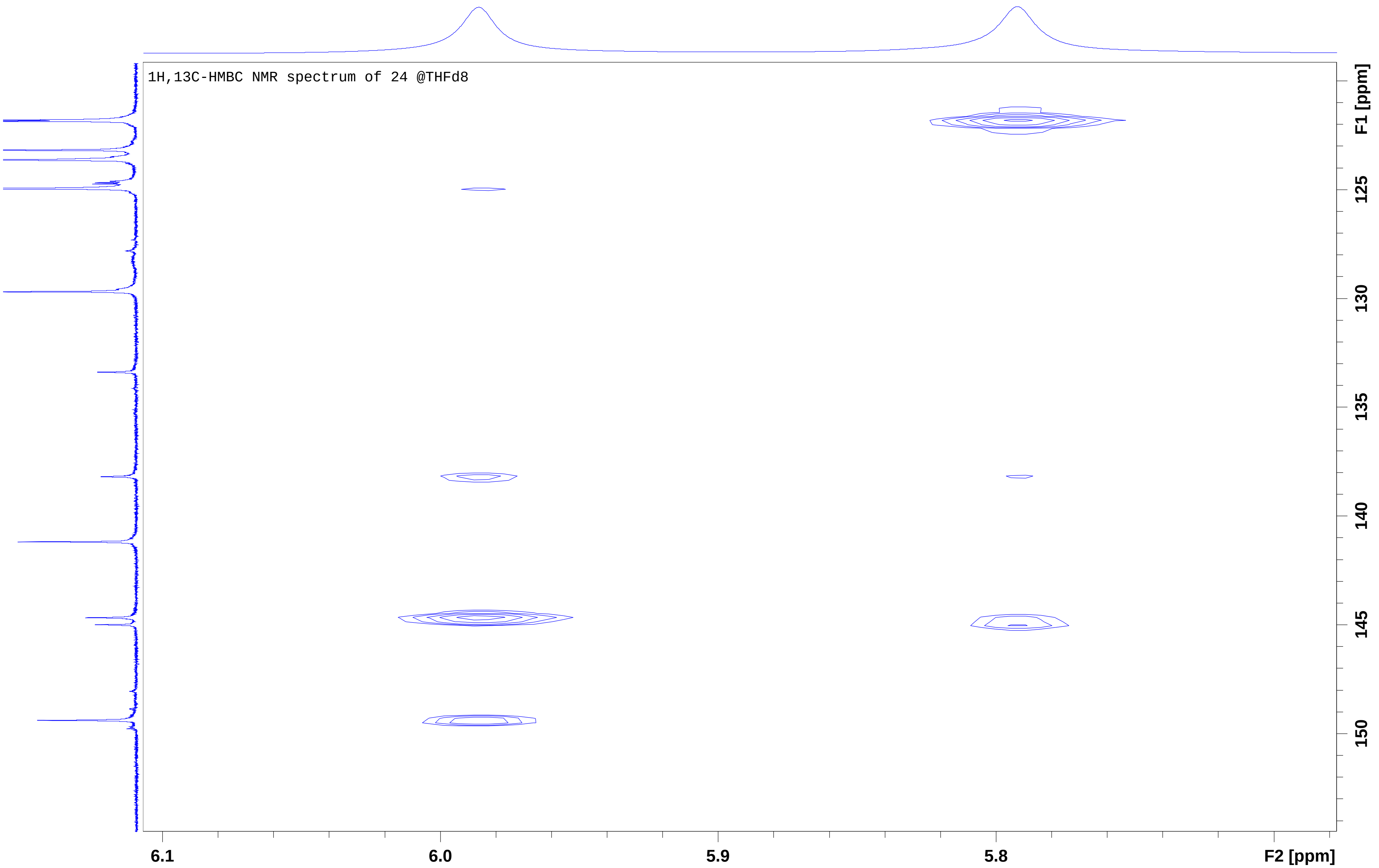

Figure S266. Detail of 1H,13C-HMBC NMR spectrum of 24 in THF-d8

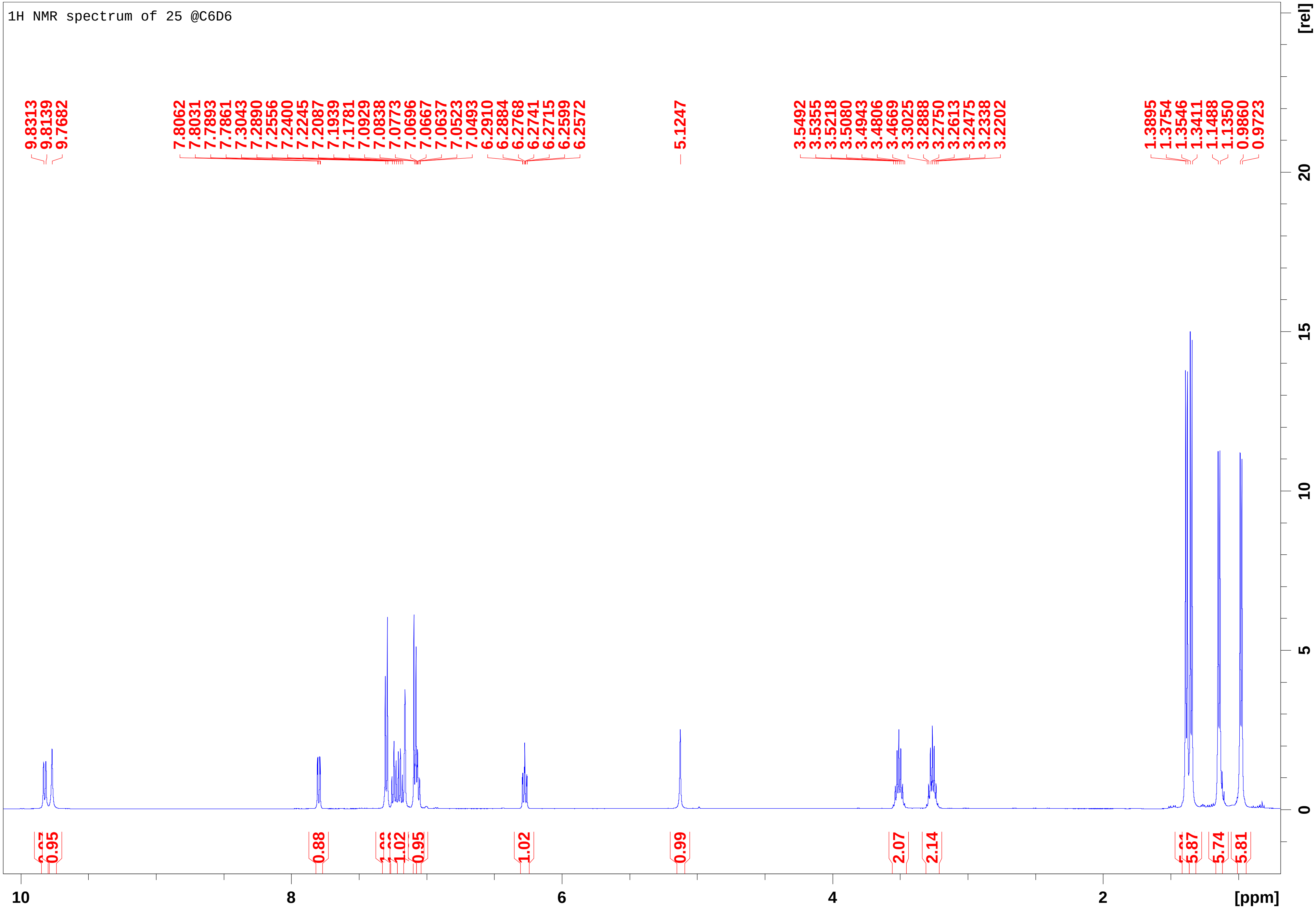

Figure S267. 1H NMR spectrum of 25 in C6D6

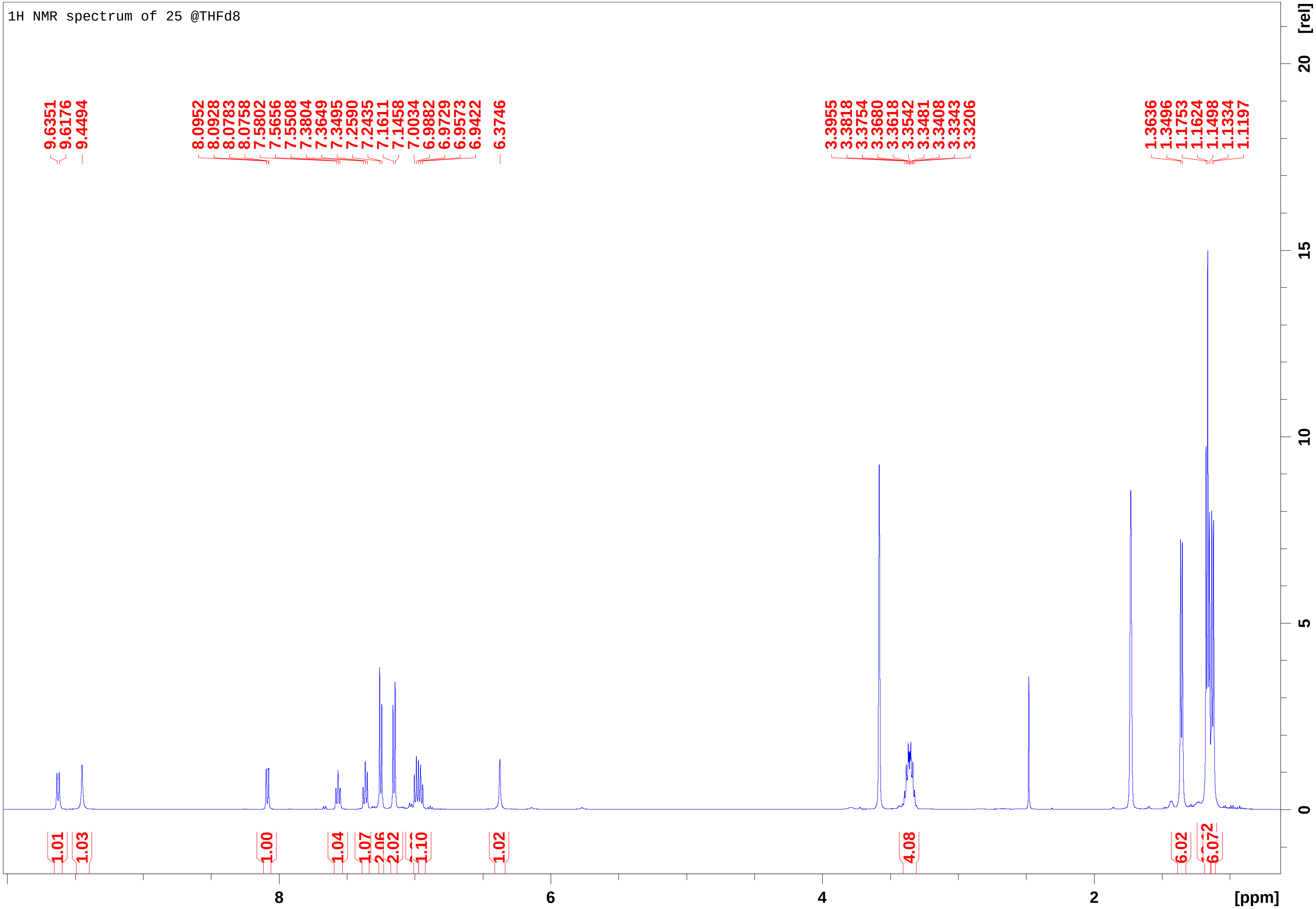

Figure S268. 1H NMR spectrum of 25 in THF-d8

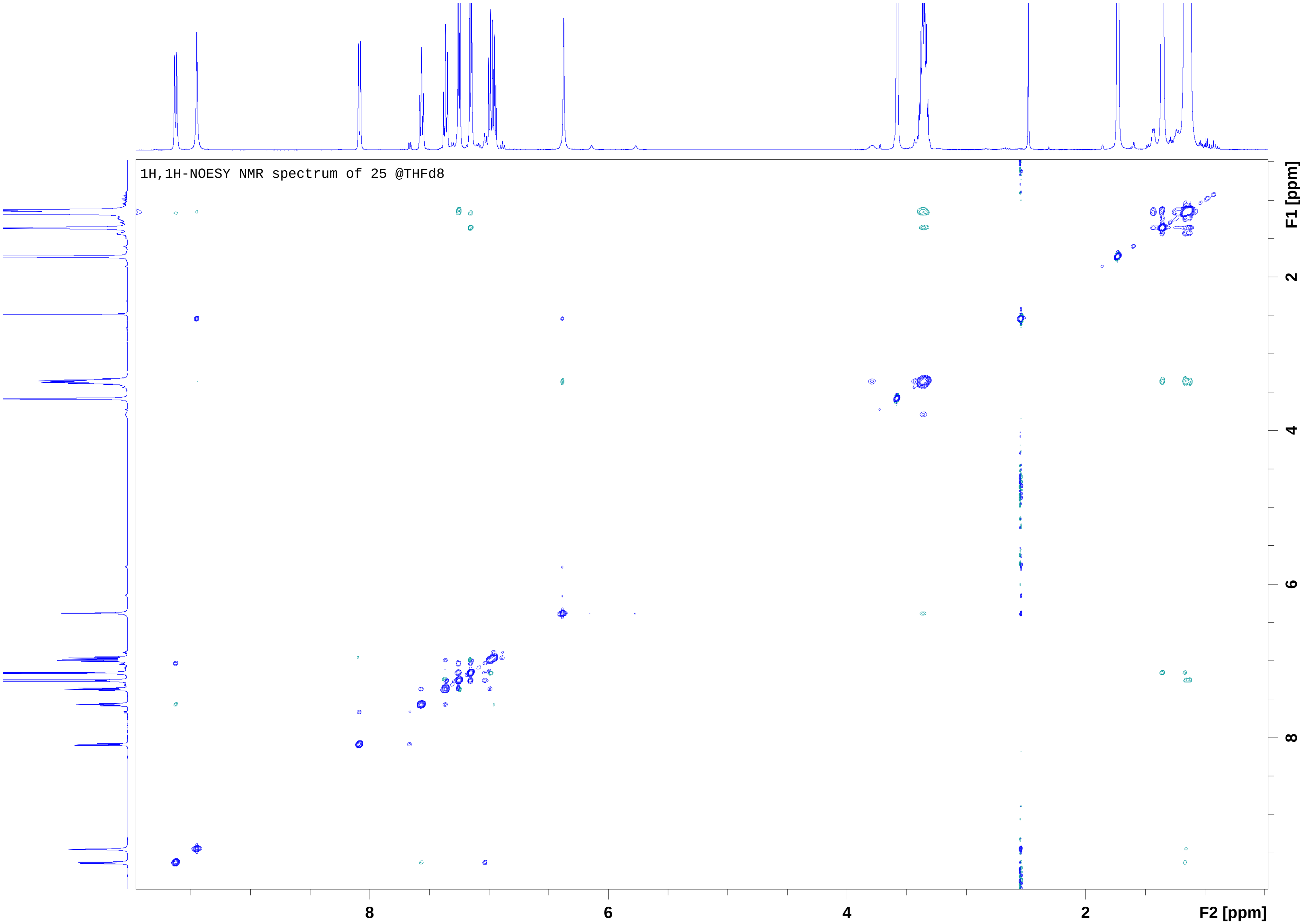

Figure S269. 1H,1H-NOESY NMR spectrum of 25 in THF-d8

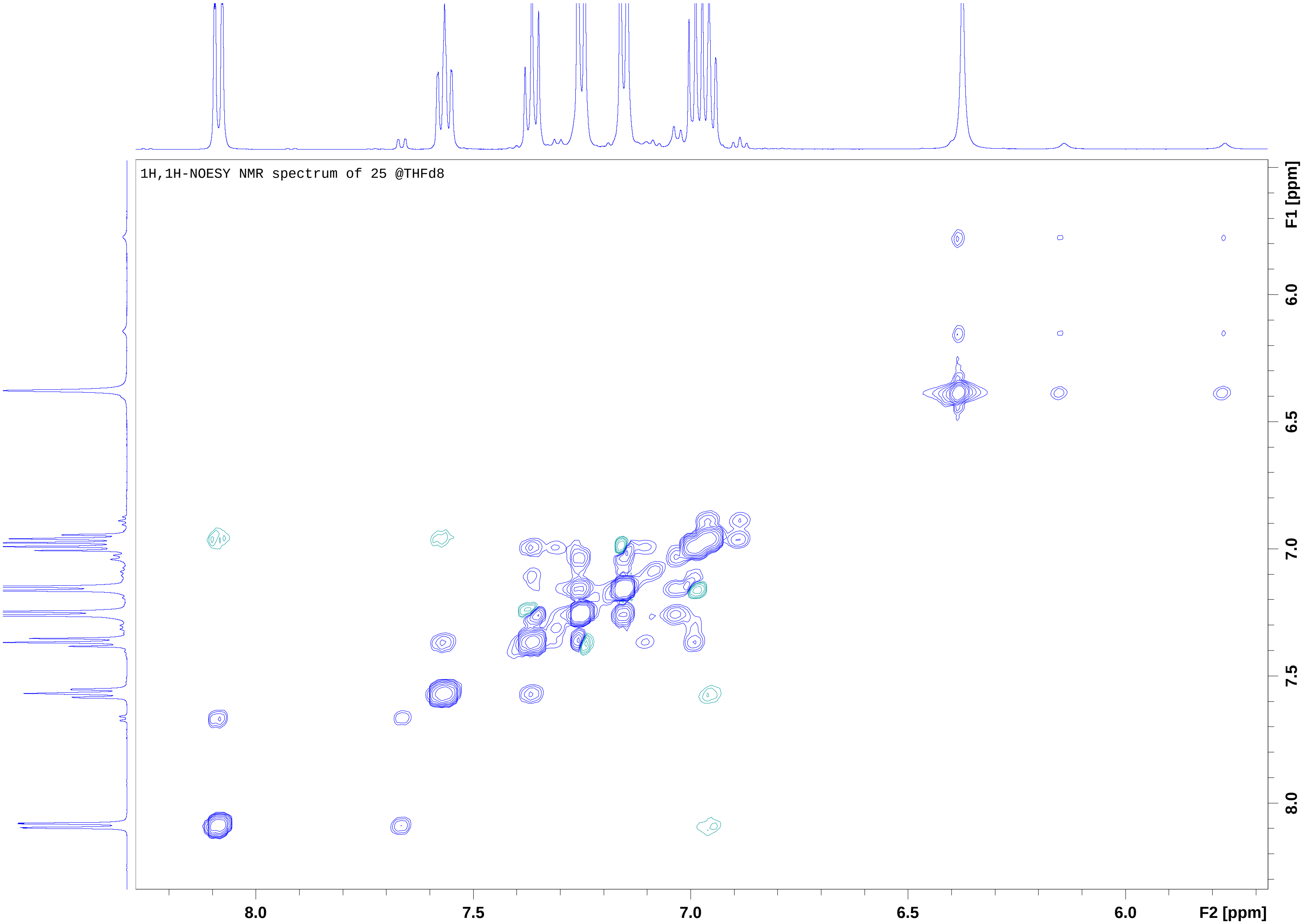

Figure S270. Detail of 1H,1H-NOESY NMR spectrum of 25 in THF-d8

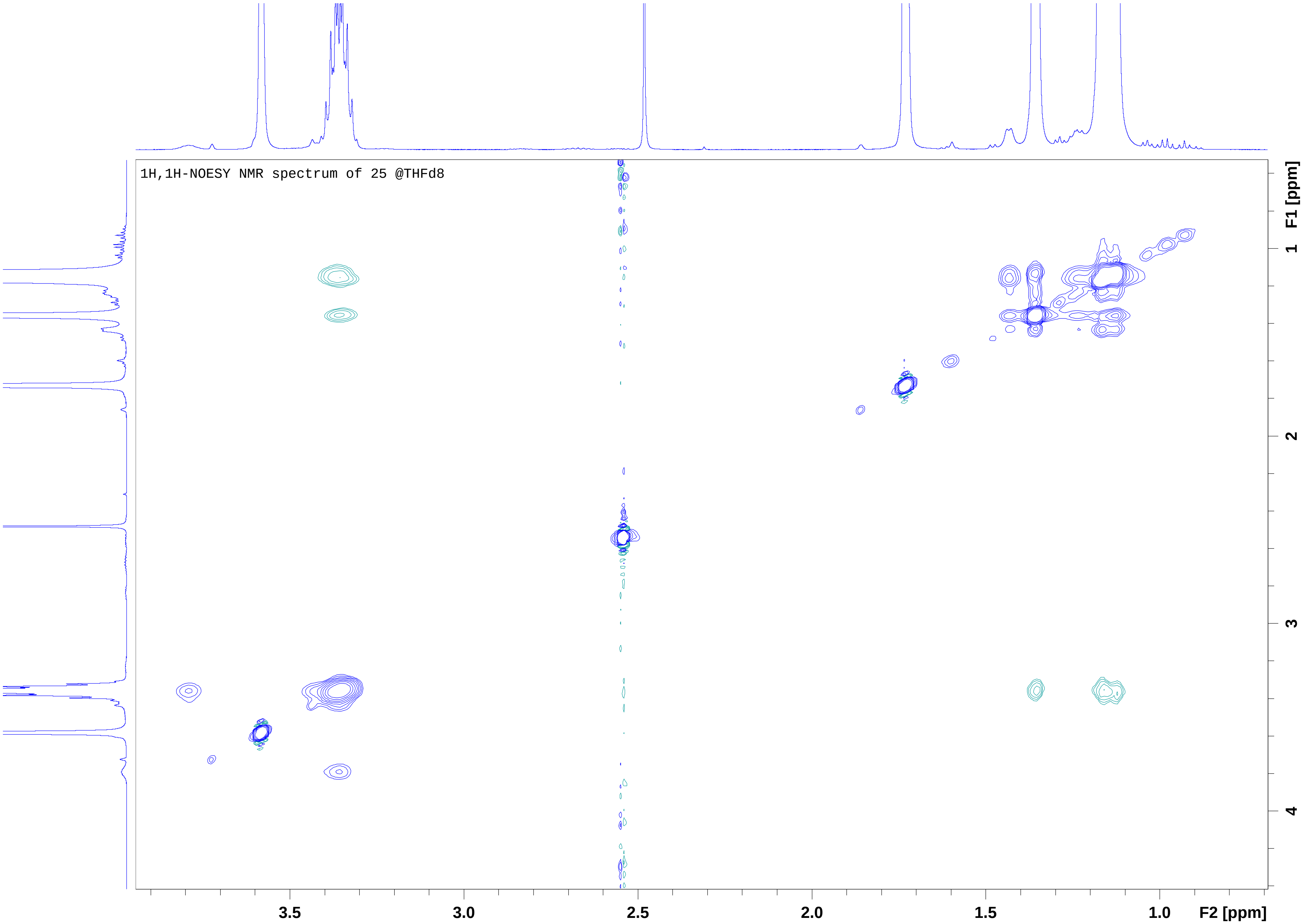

Figure S271. Detail of 1H,1H-NOESY NMR spectrum of 25 in THF-d8

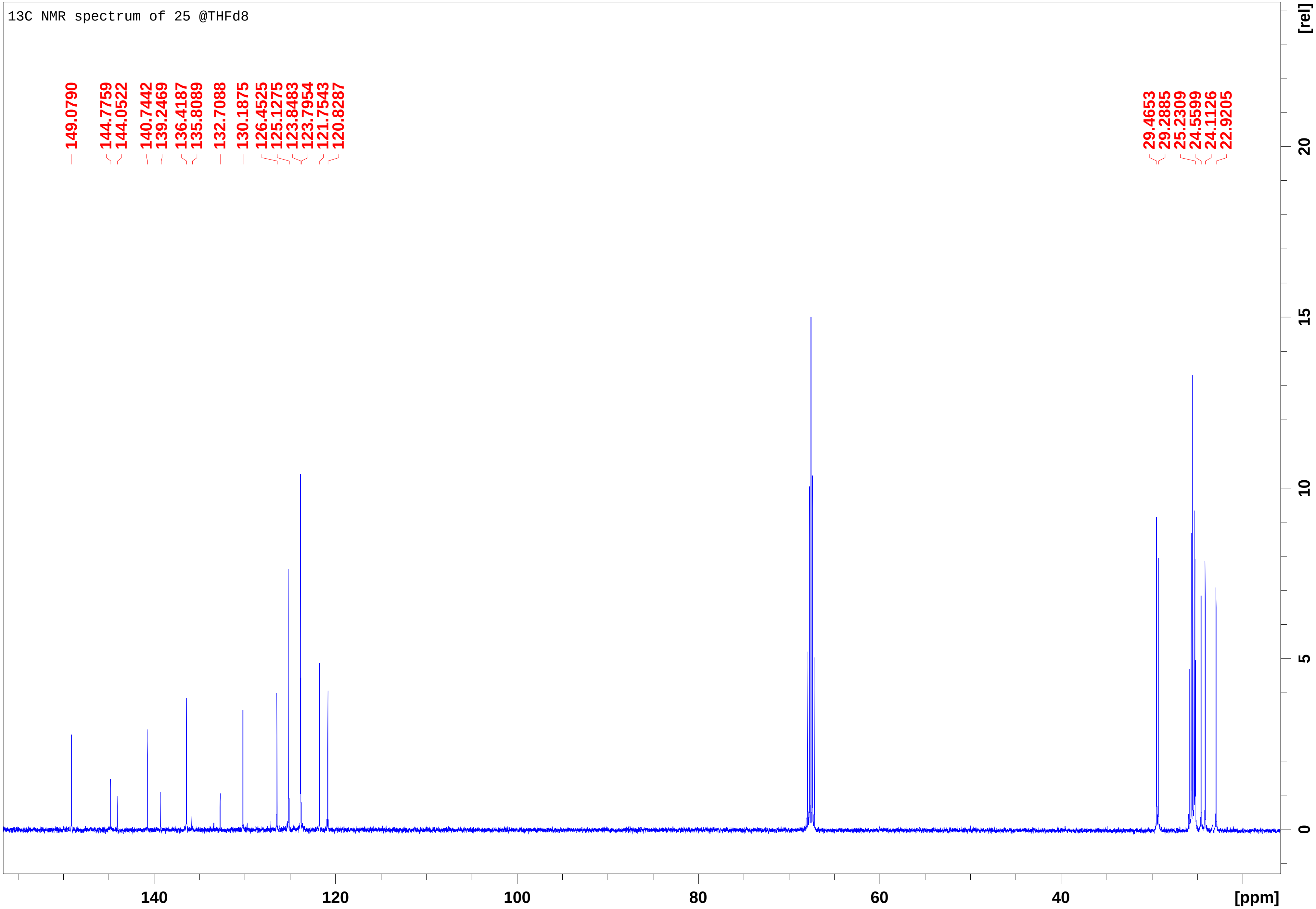

Figure S272. 13C NMR spectrum of 25 in THF-d8

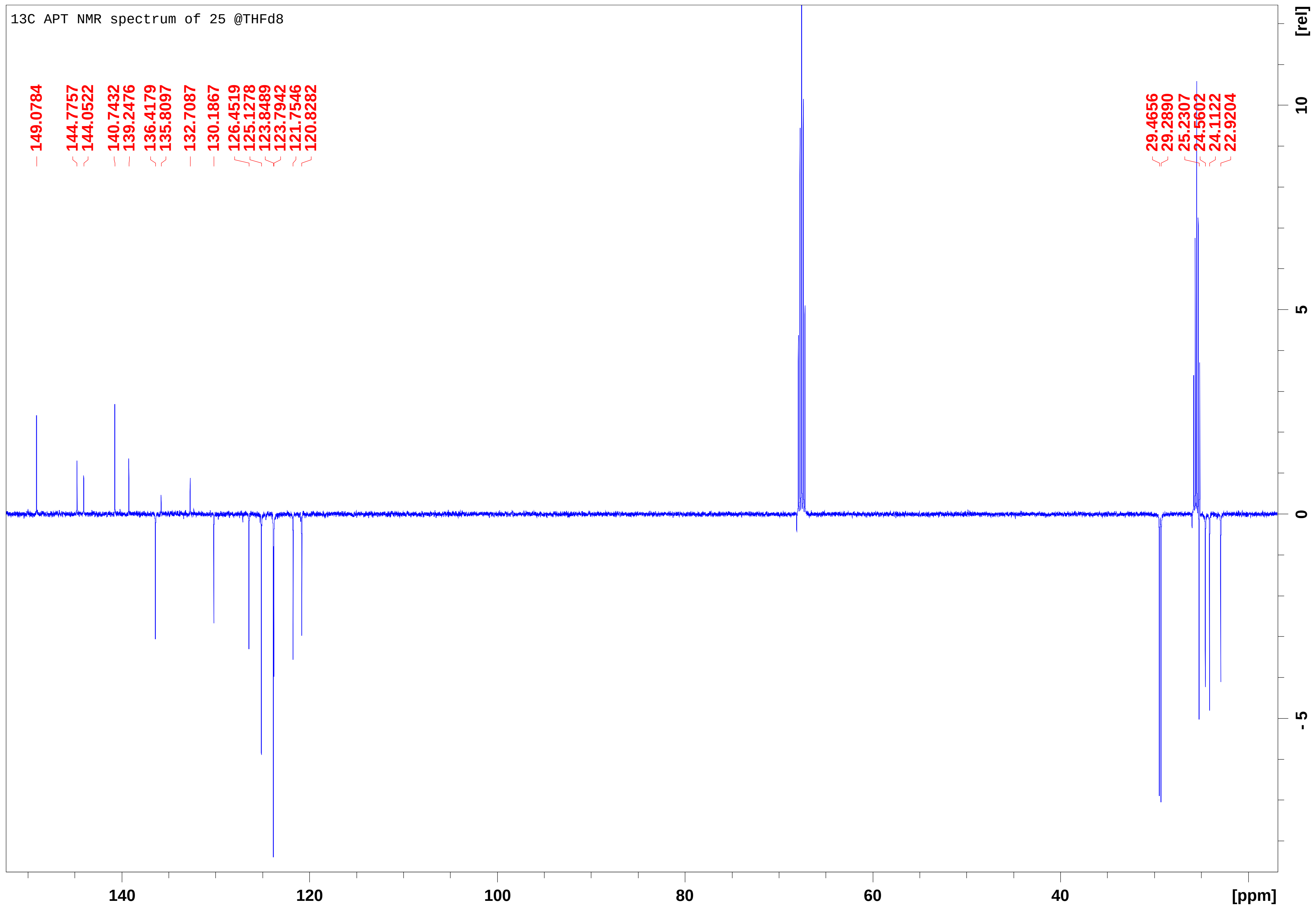

Figure S273. 13C APT NMR spectrum of 25 in THF-d8

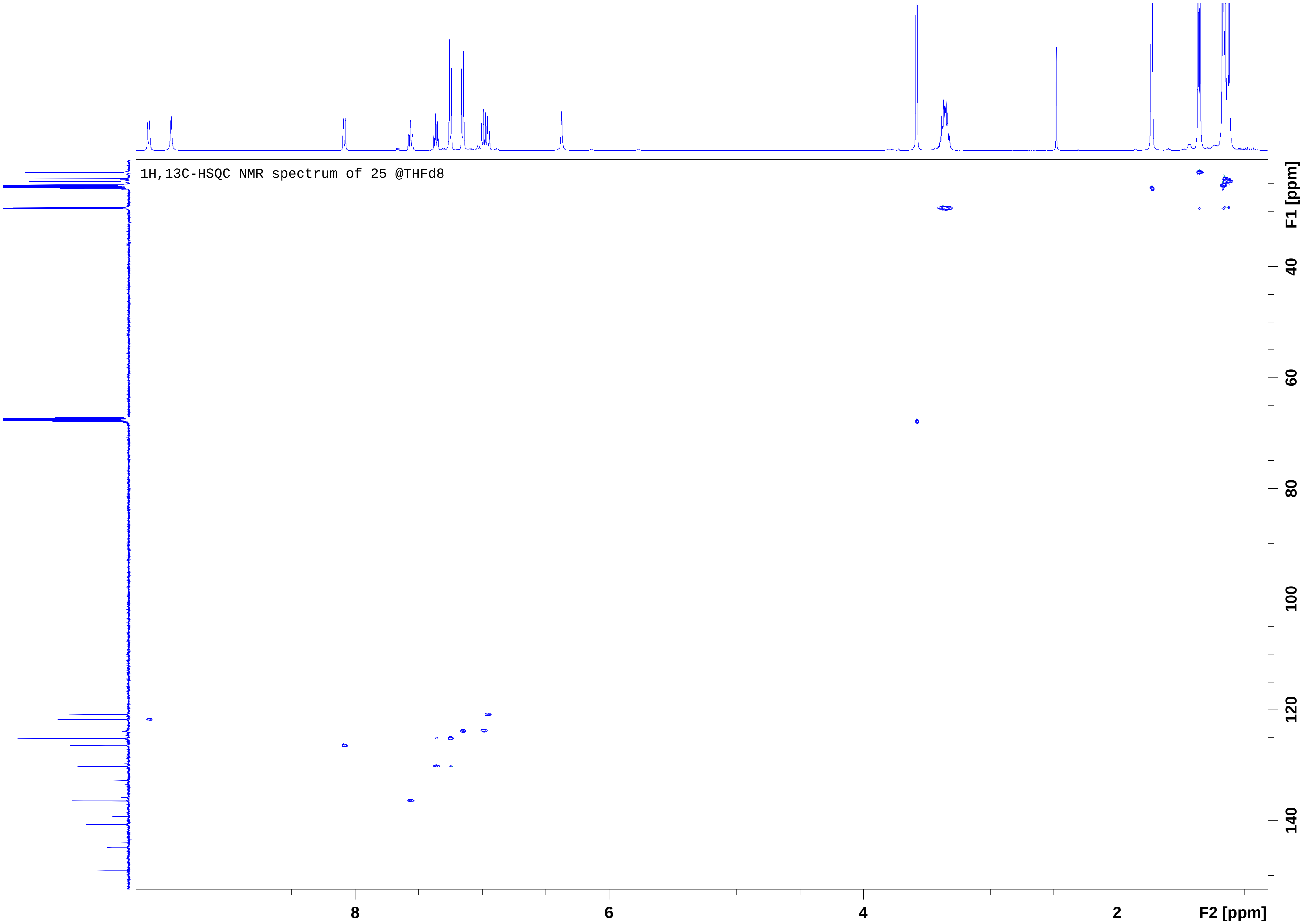

Figure S274. 1H,13C-HSQC NMR spectrum of 25 in THF-d8

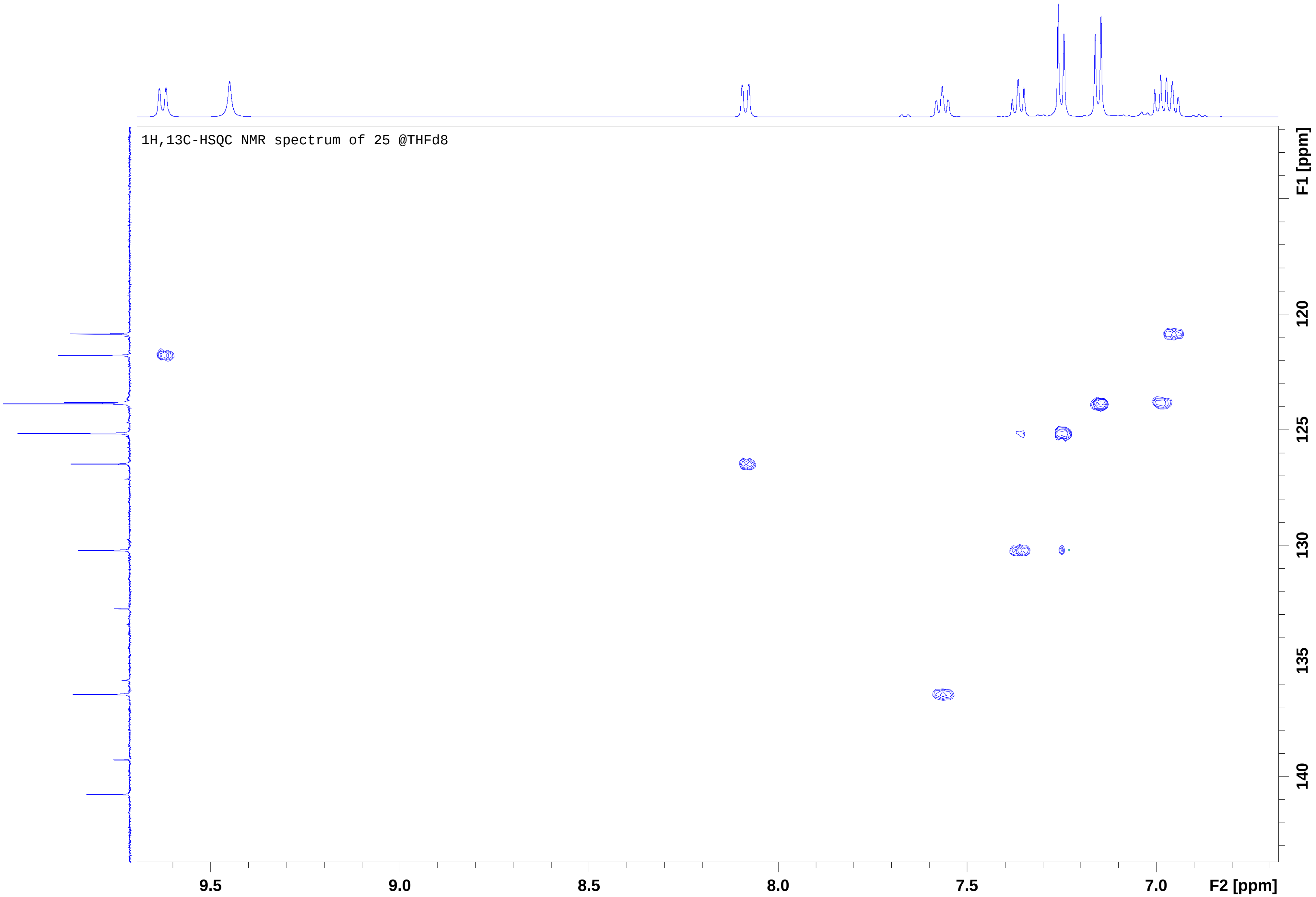

Figure S275. Detail of 1H,13C-HSQC NMR spectrum of 25 in THF-d8

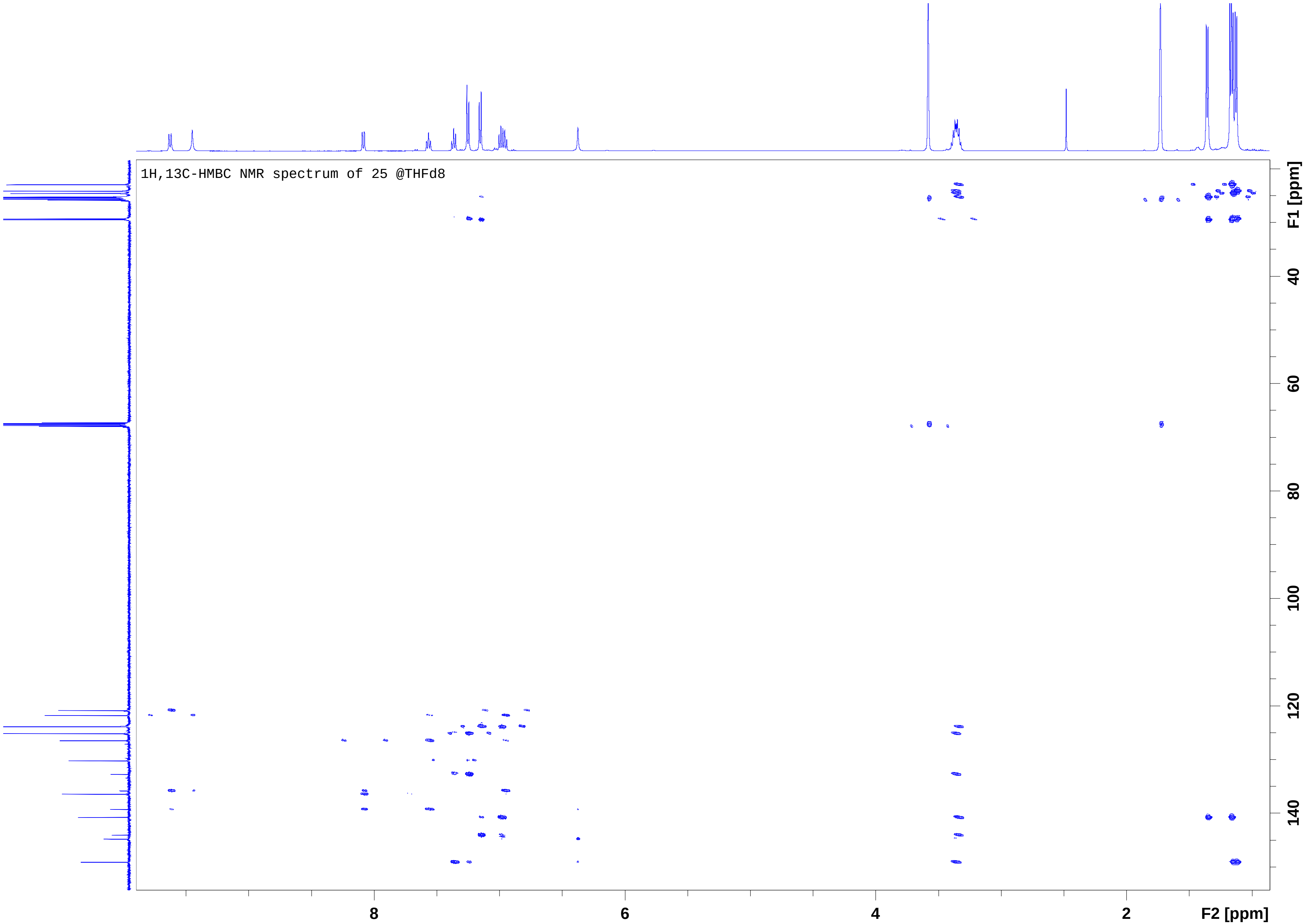

Figure S276. 1H,13C-HMBC NMR spectrum of 25 in THF-d8

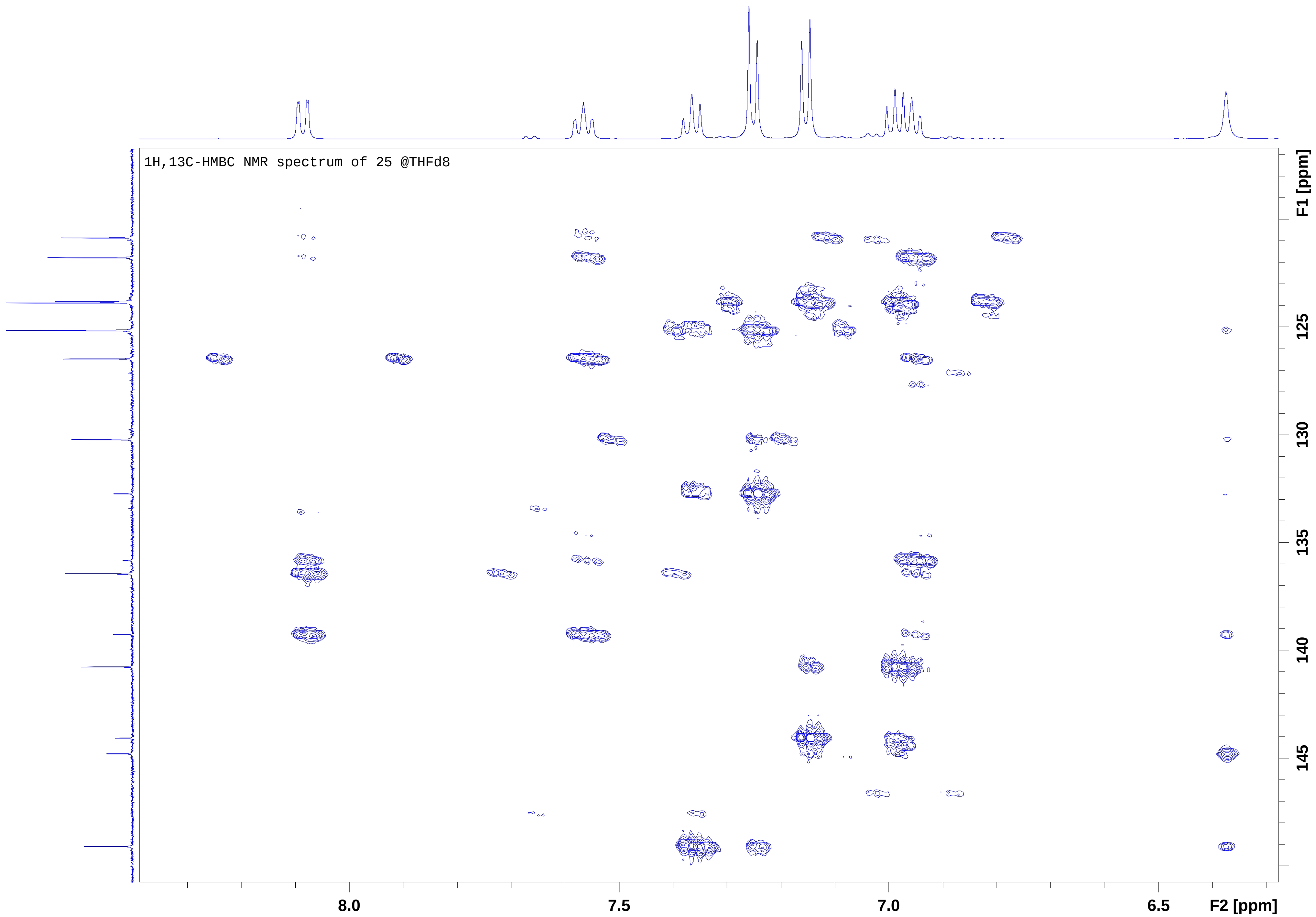

Figure S277. Detail of 1H,13C-HMBC NMR spectrum of 25 in THF-d8

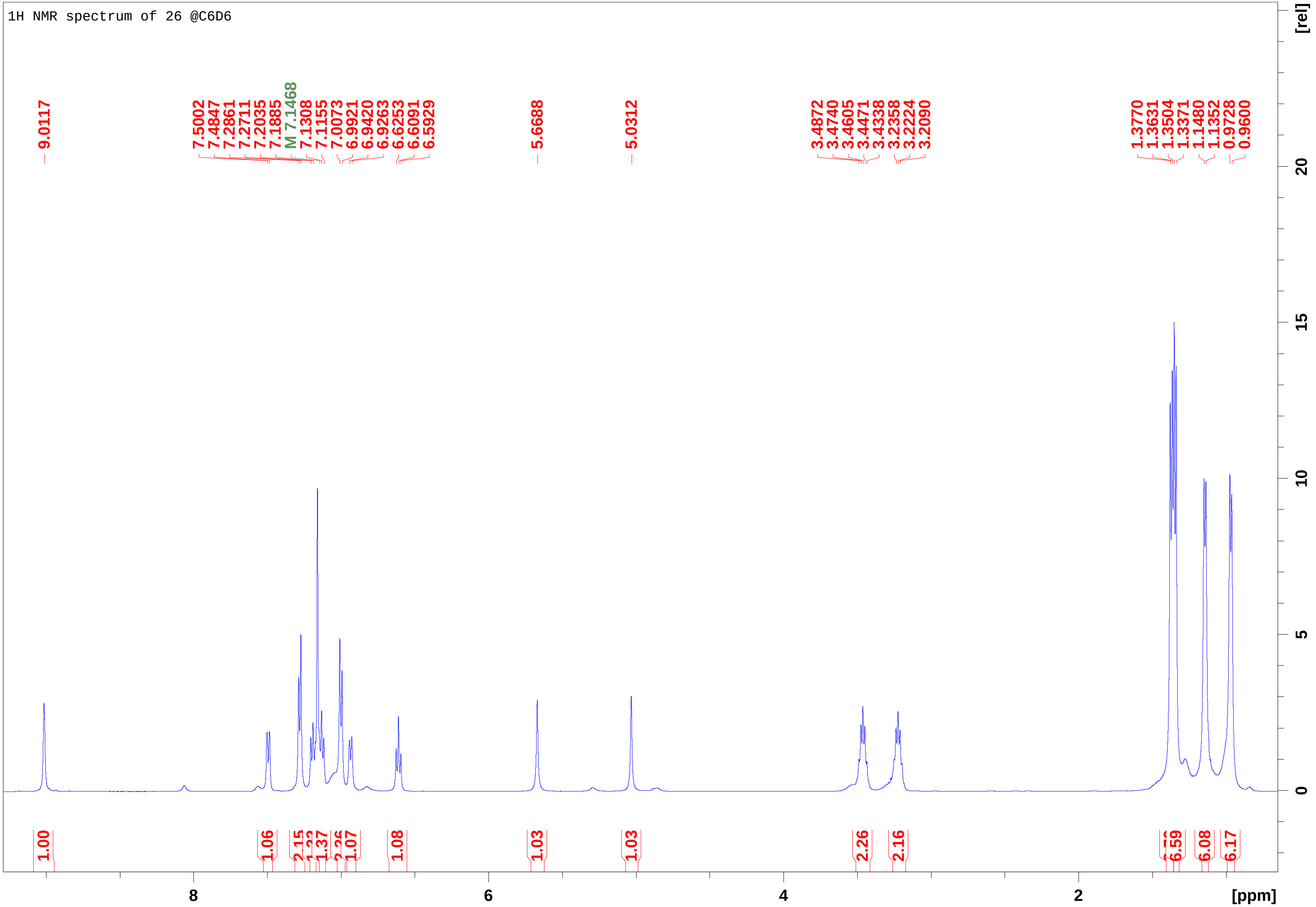

Figure S278. 1H NMR spectrum of 26 in C6D6

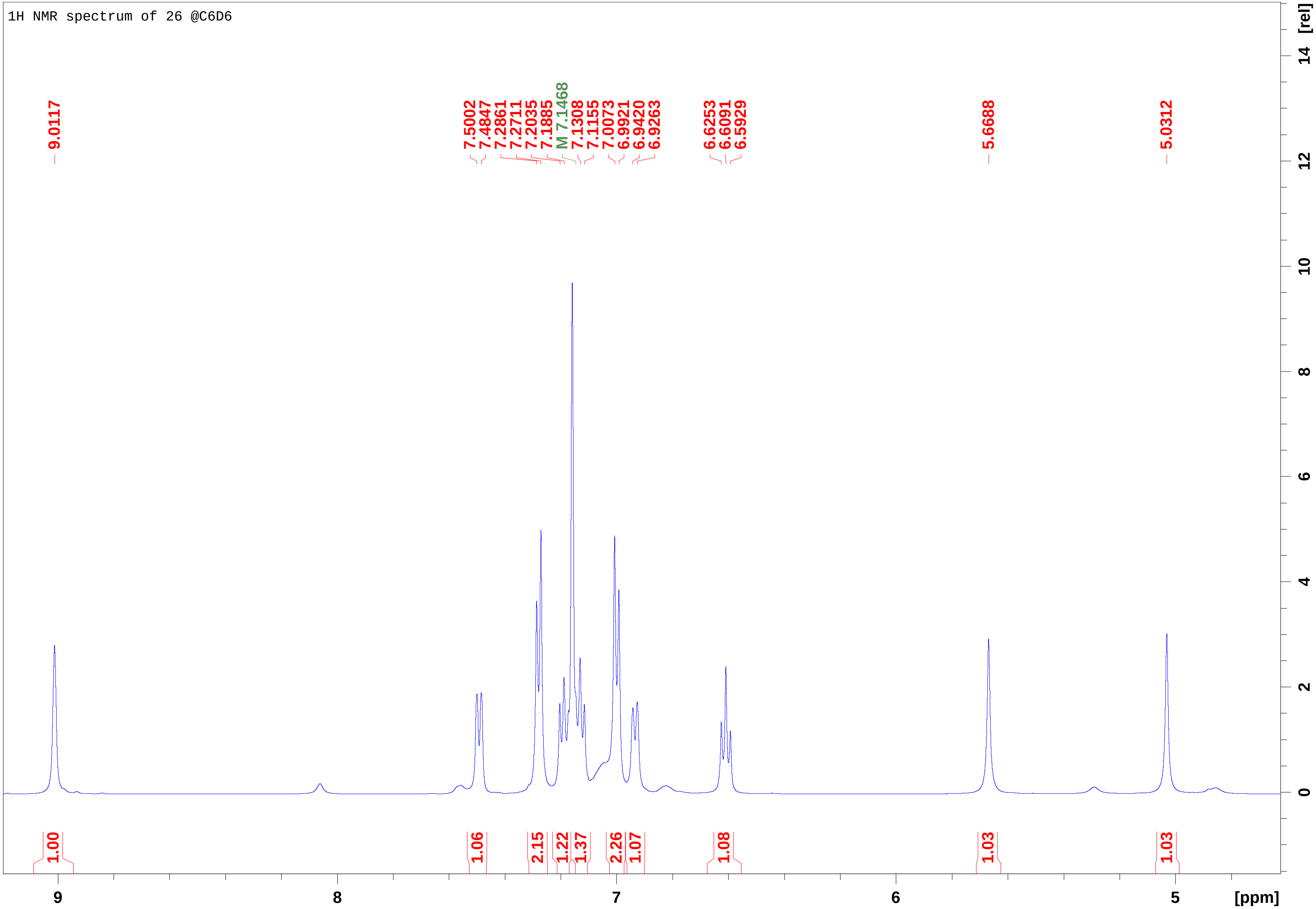

Figure S279. Detail of 1H NMR spectrum of 26 in C6D6

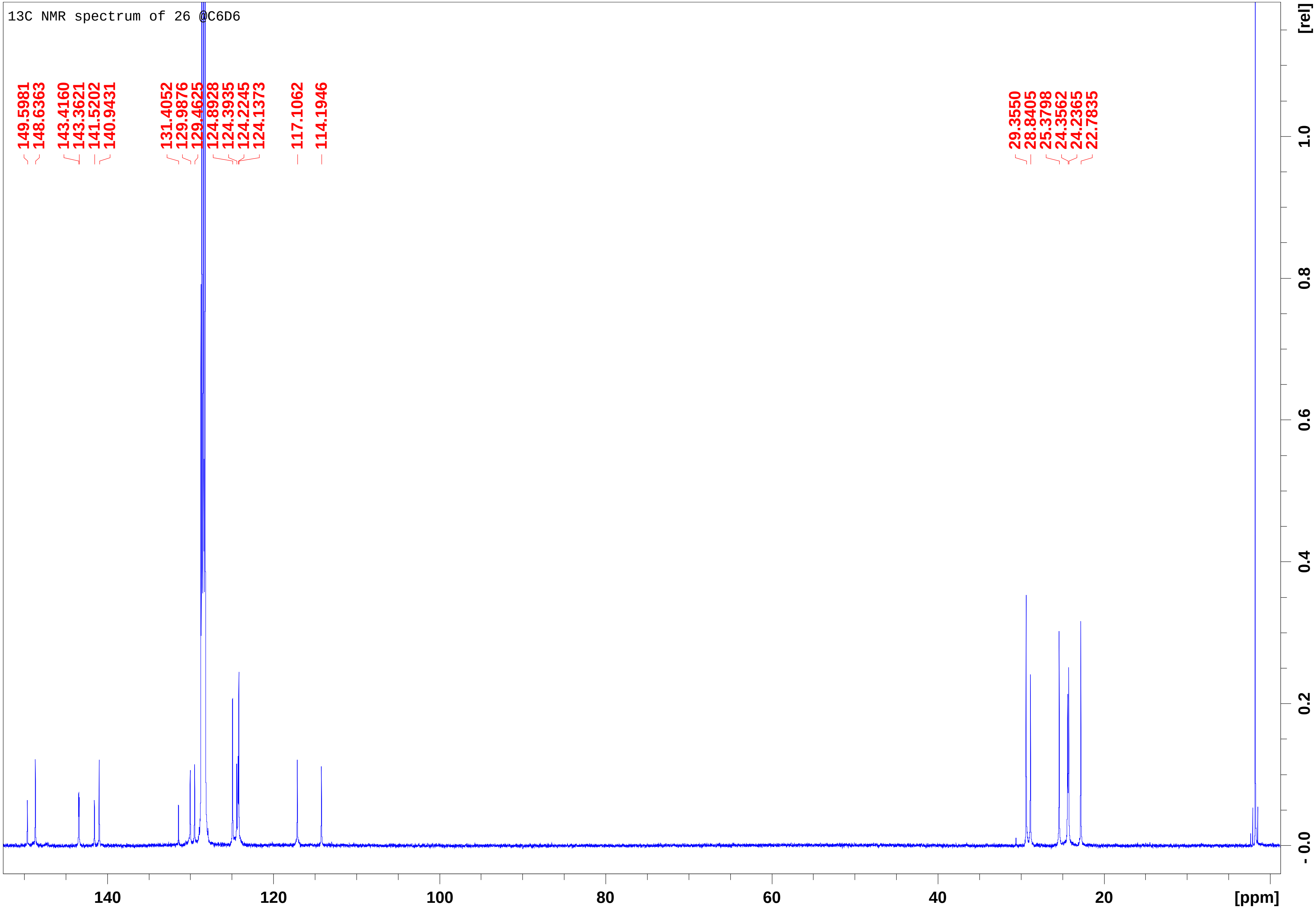

Figure S280. 13C NMR spectrum of 26 in C6D6

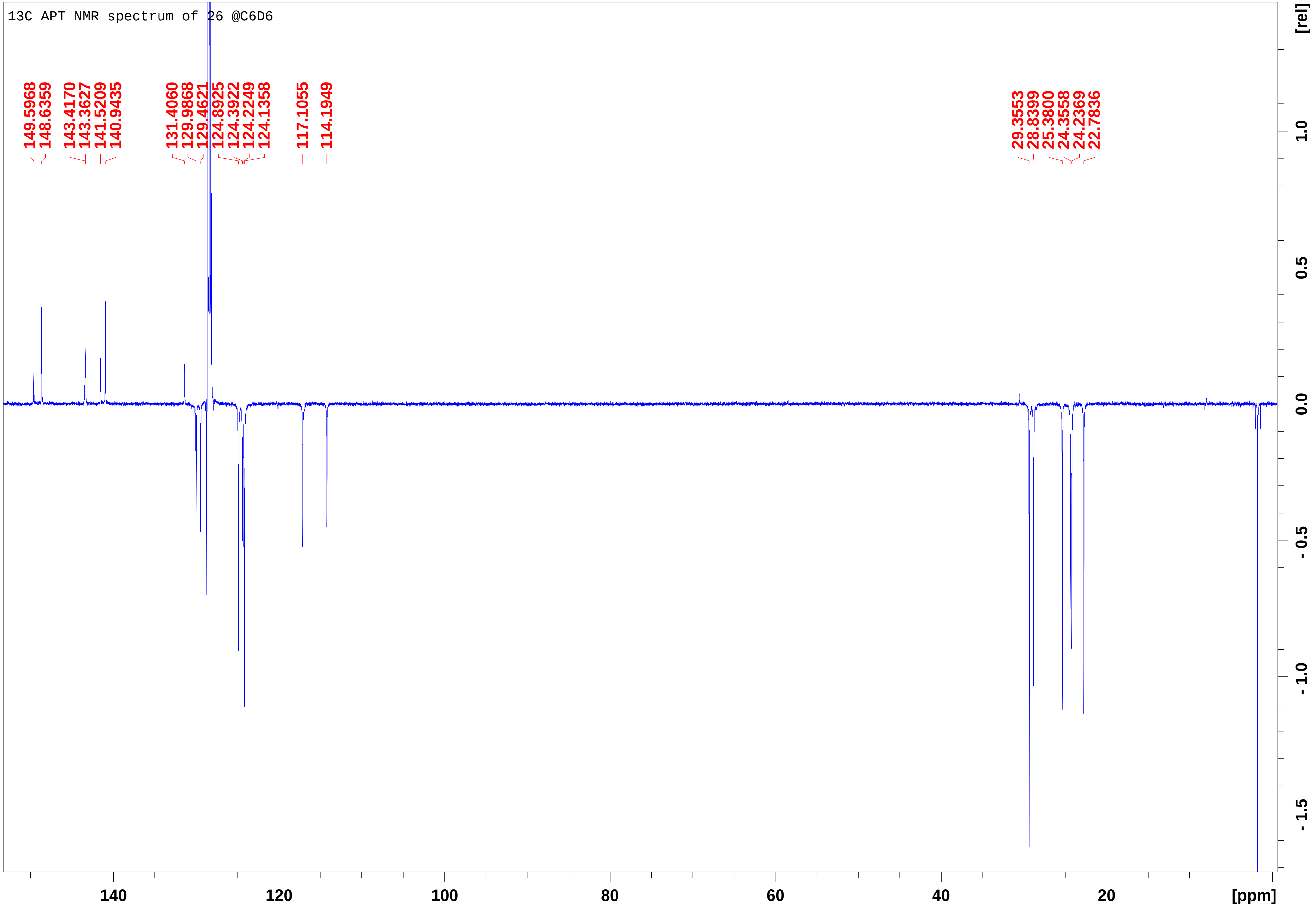

Figure S281. 13C APT NMR spectrum of 26 in C6D6

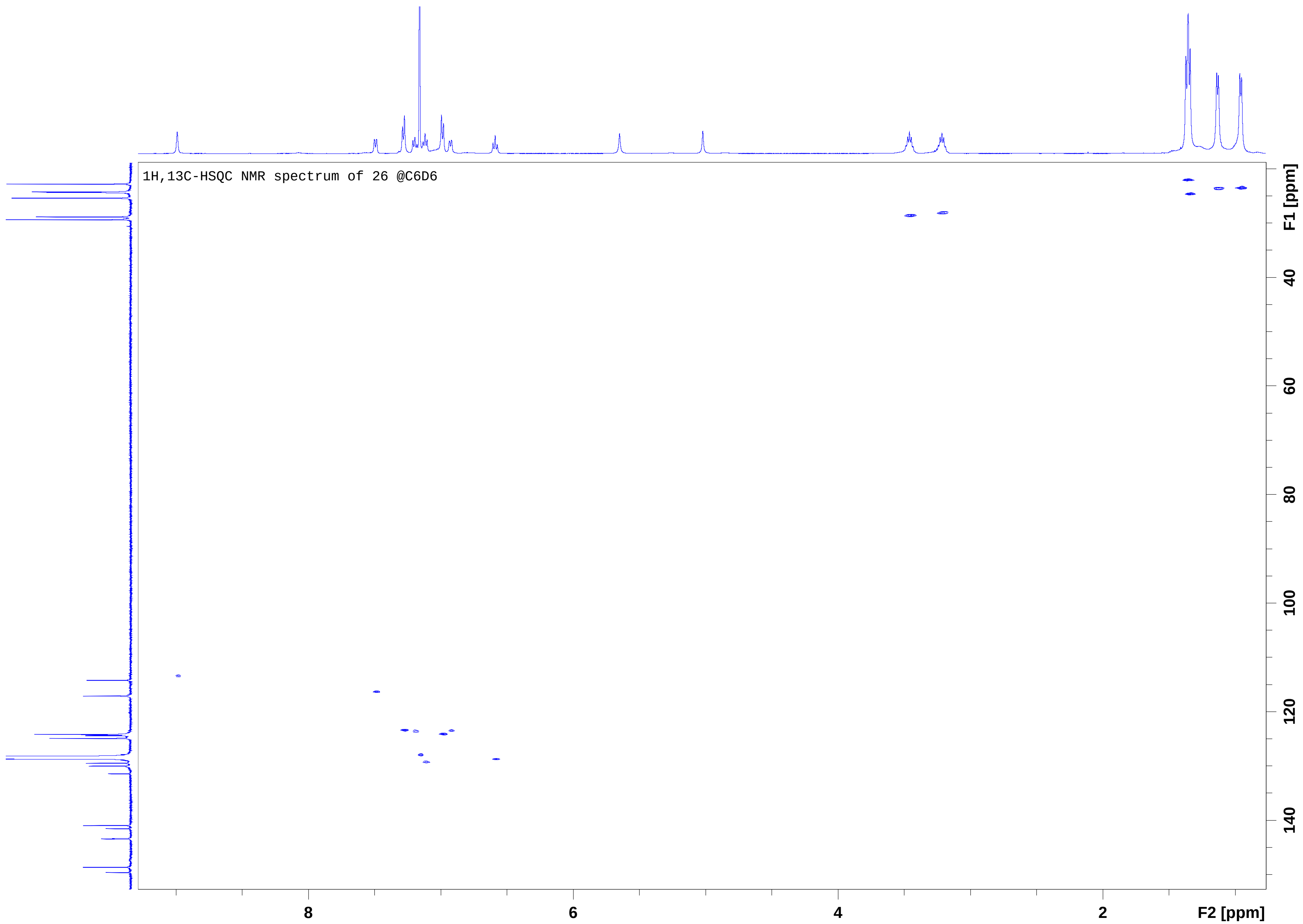

Figure S282. 1H,13C-HSQC NMR spectrum of 26 in C6D6

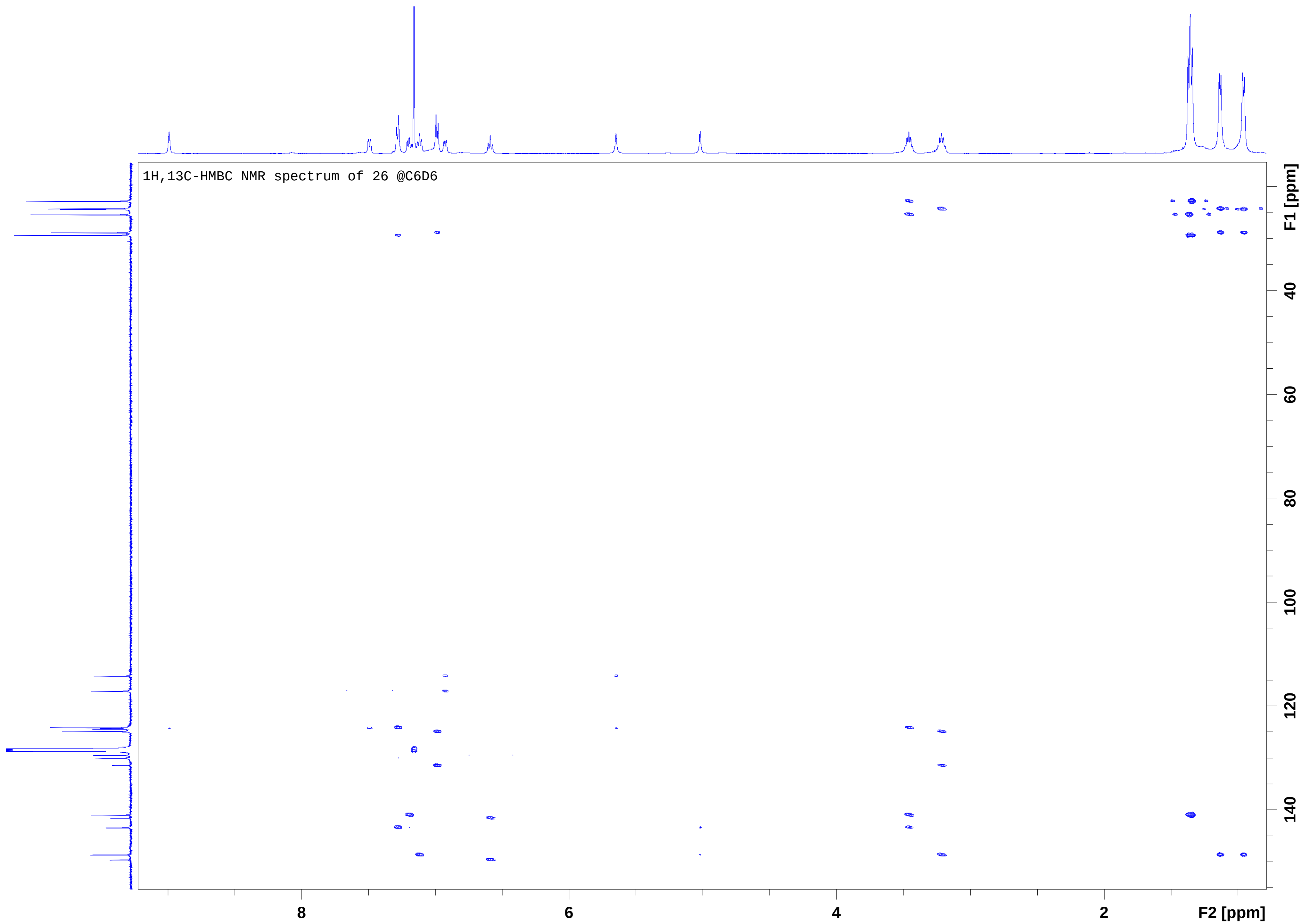

Figure S283. 1H,13C-HMBC NMR spectrum of 26 in C6D6

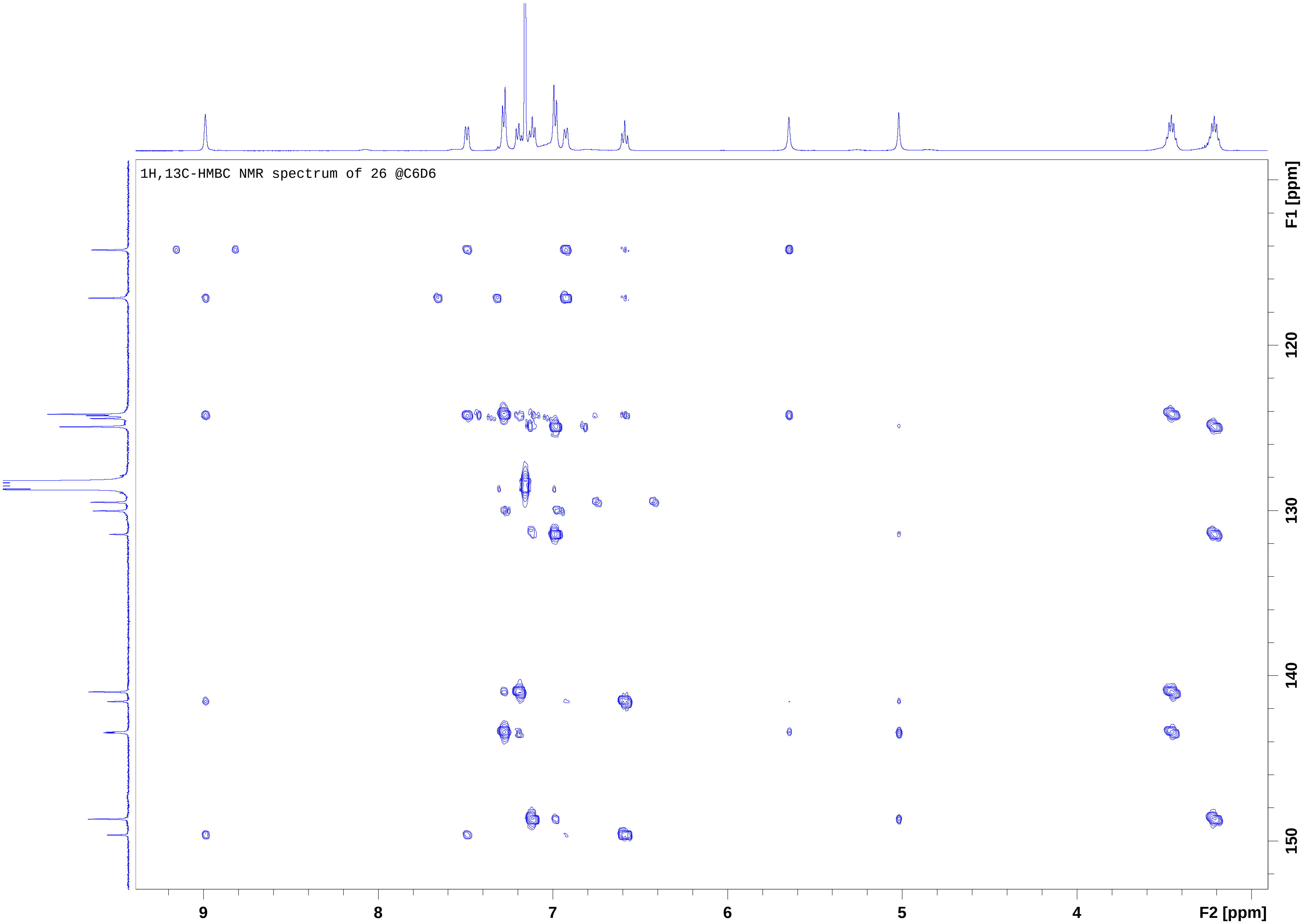

Figure S284. Detail of 1H,13C-HMBC NMR spectrum of 26 in C6D6

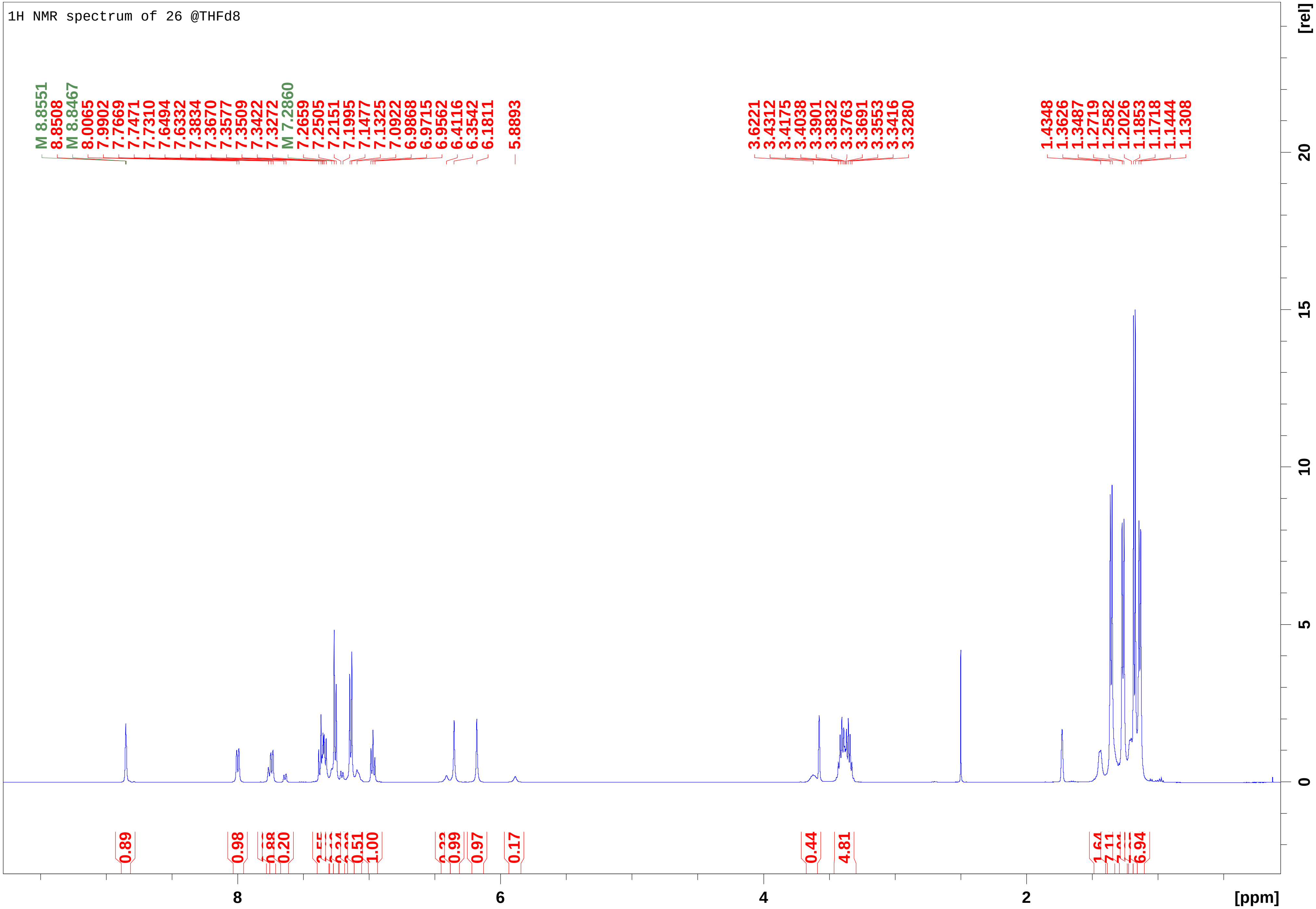

Figure S285. 1H NMR spectrum of 26 in THF-d8

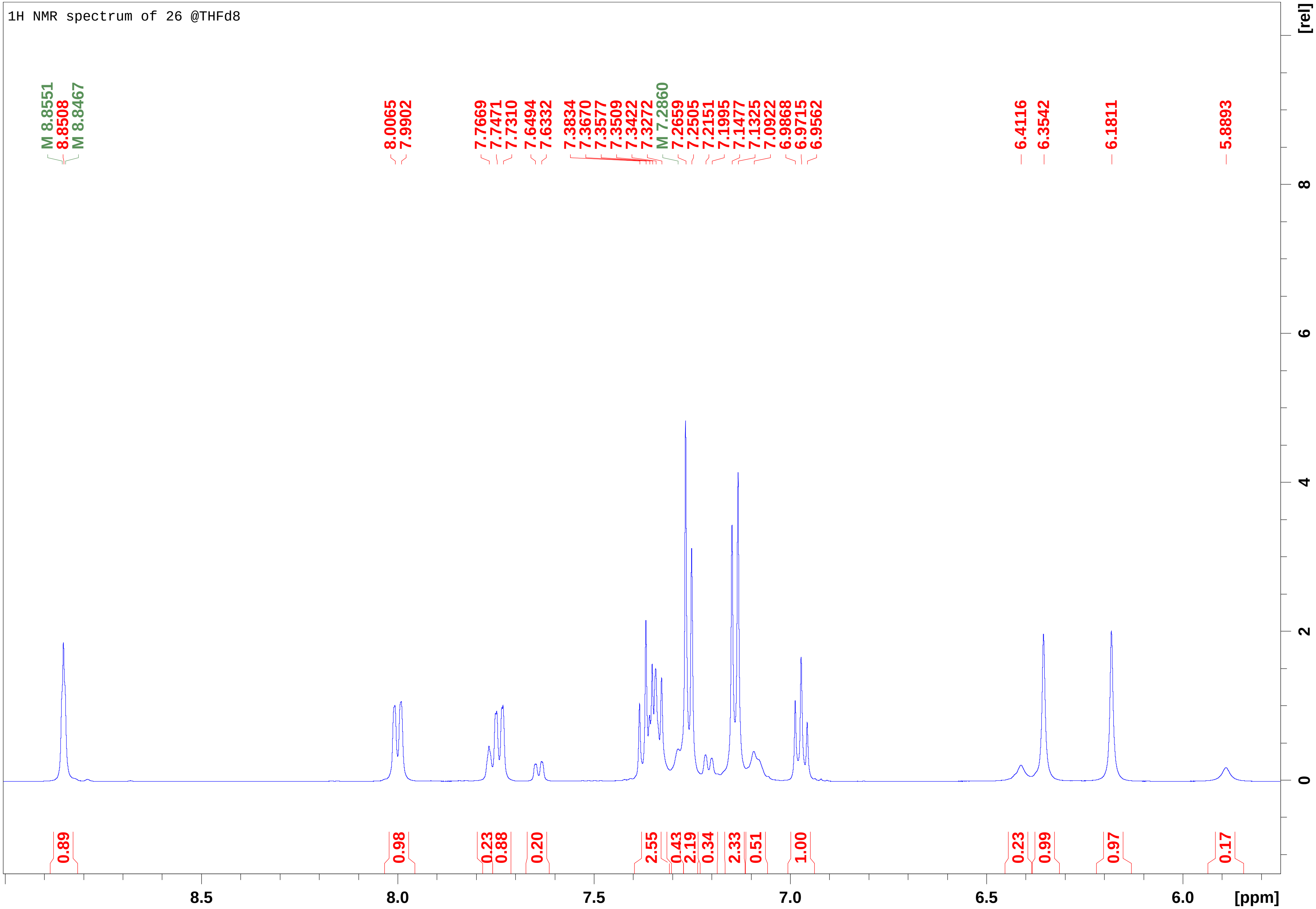

Figure S286. Detail of 1H NMR spectrum of 26 in THF-d8

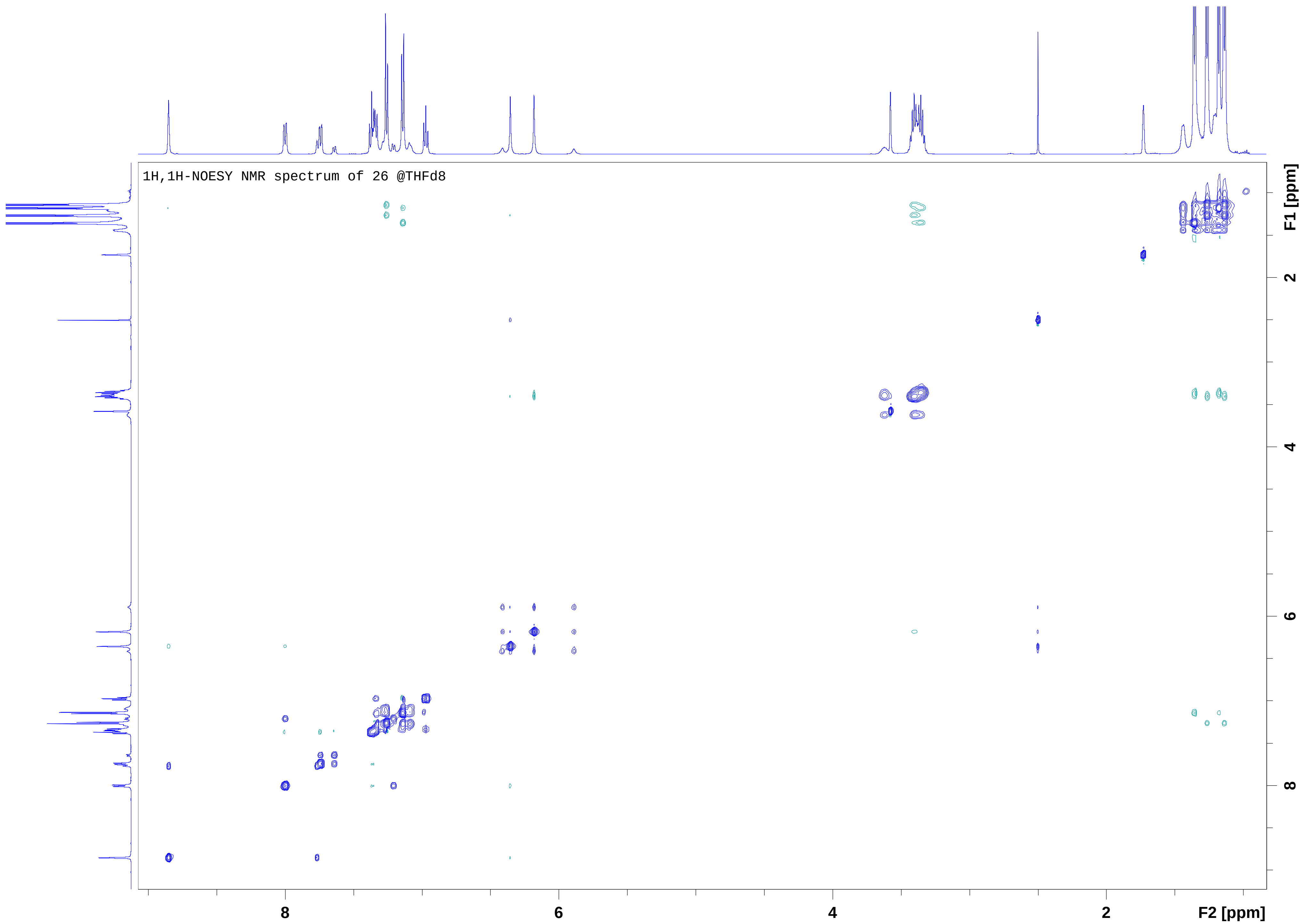

Figure S287. 1H,1H-NOESY NMR spectrum of 26 in THF-d8

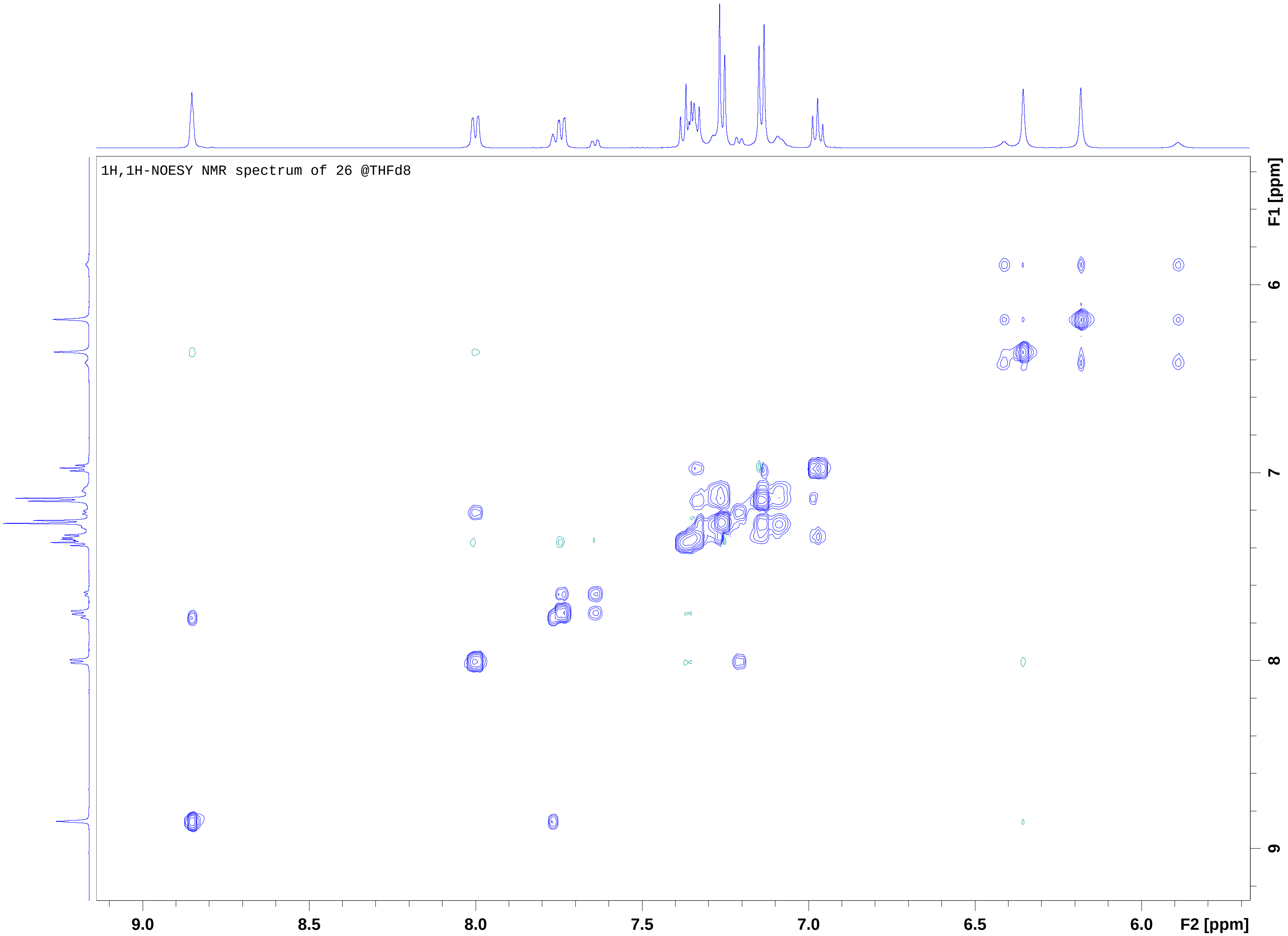

Figure S288. Detail of 1H,1H-NOESY NMR spectrum of 26 in THF-d8

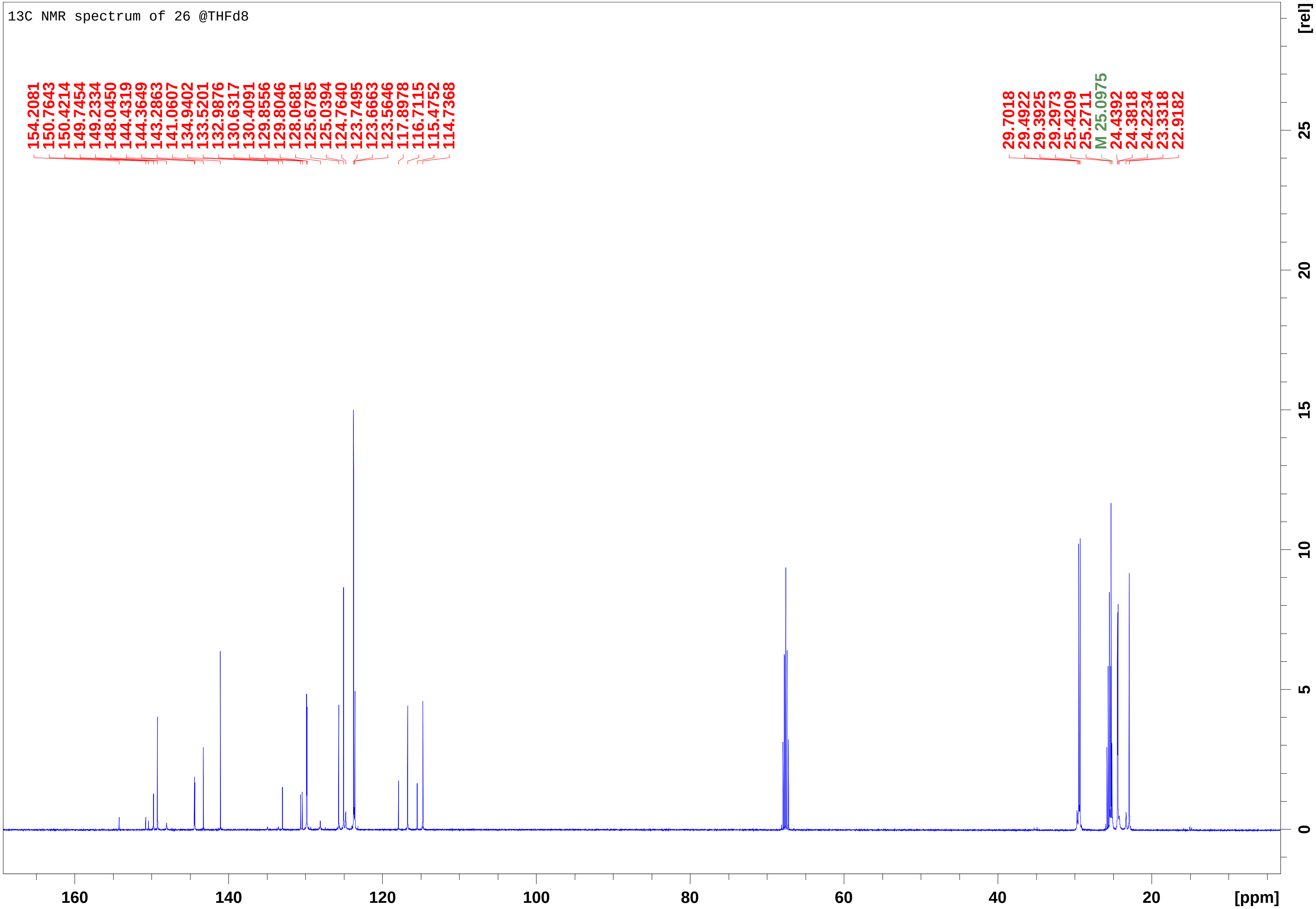

Figure S289. 13C NMR spectrum of 26 in THF-d8

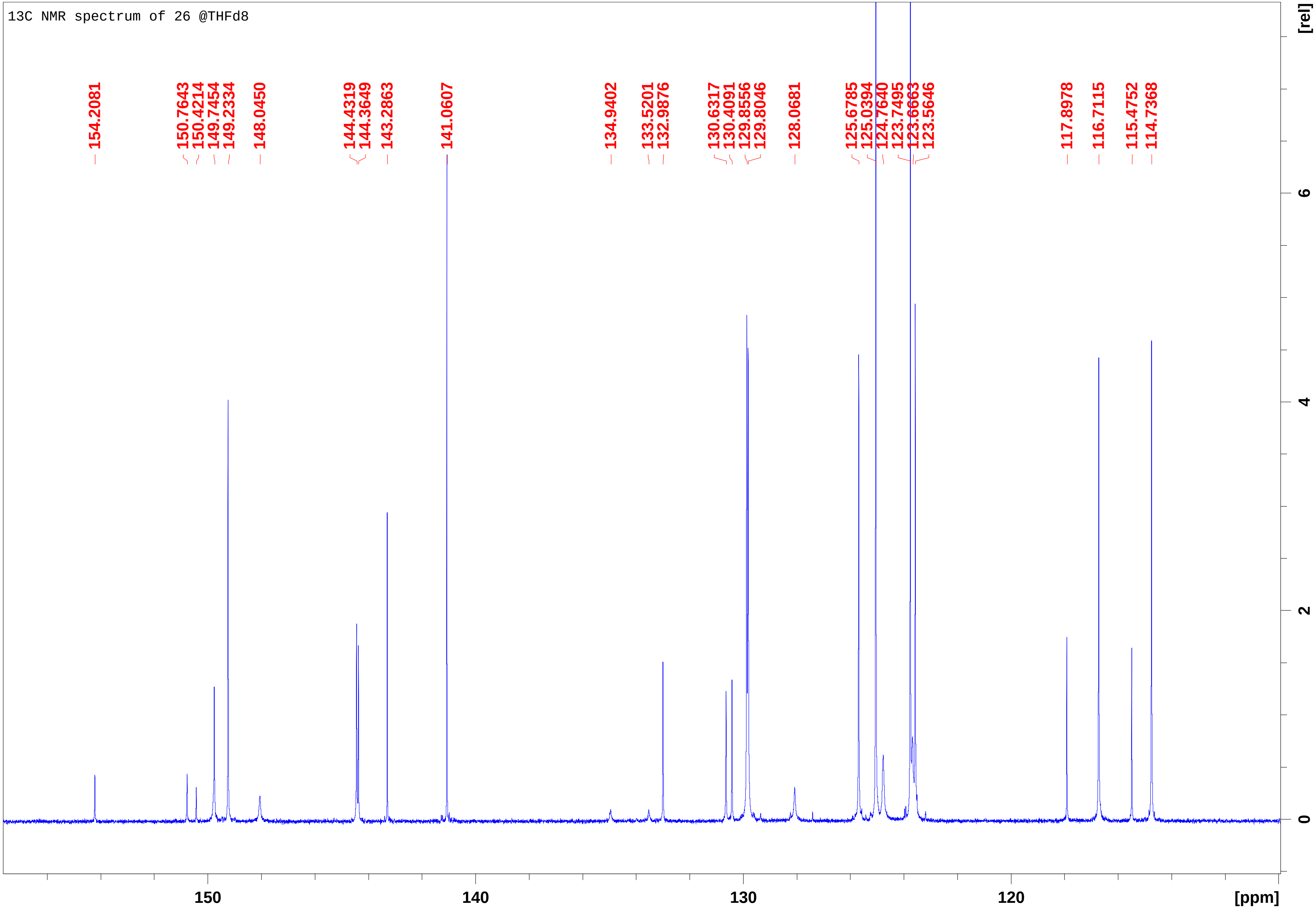

Figure S290. Detail of 13C NMR spectrum of 26 in THF-d8

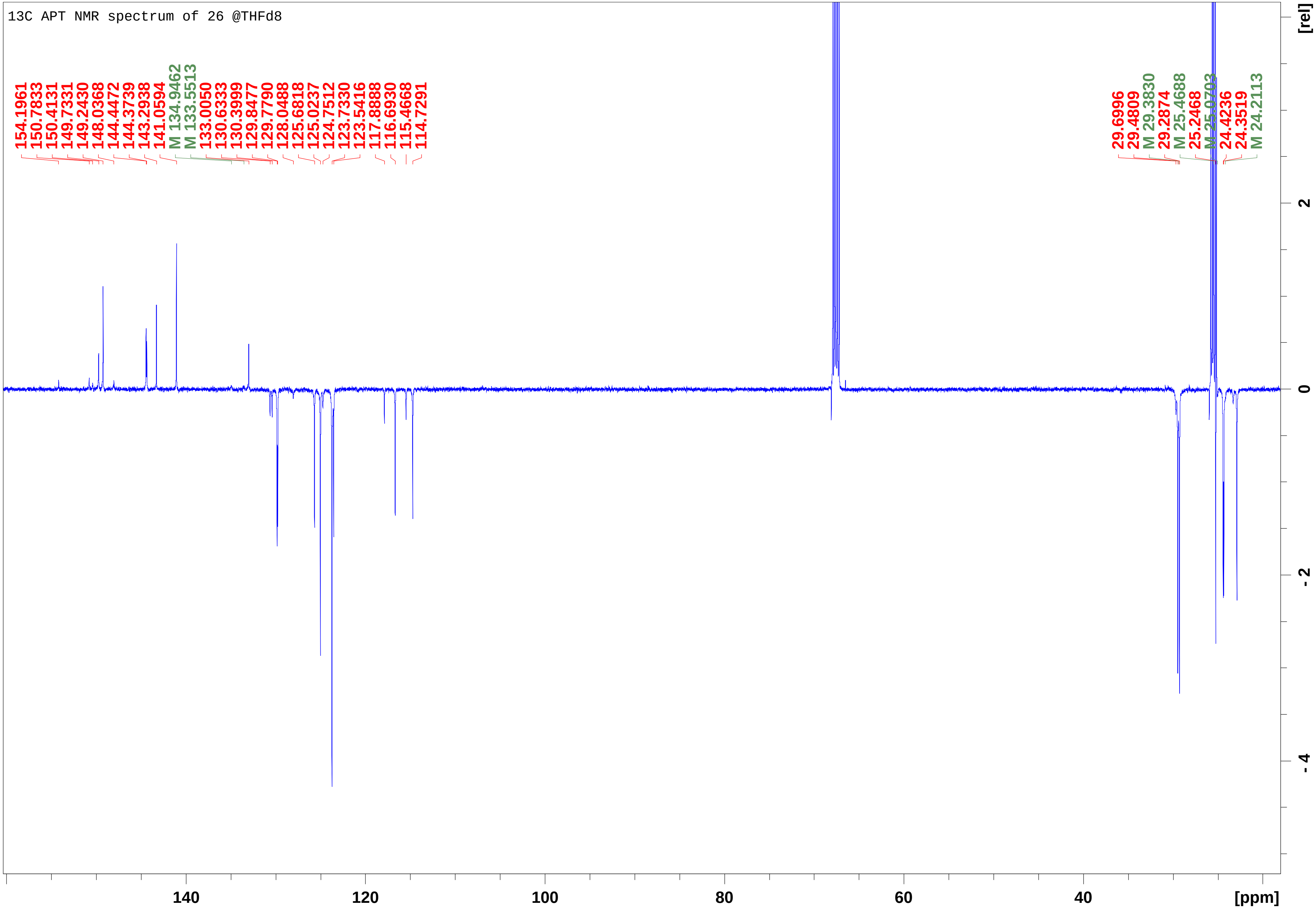

Figure S291. 13C APT NMR spectrum of 26 in THF-d8

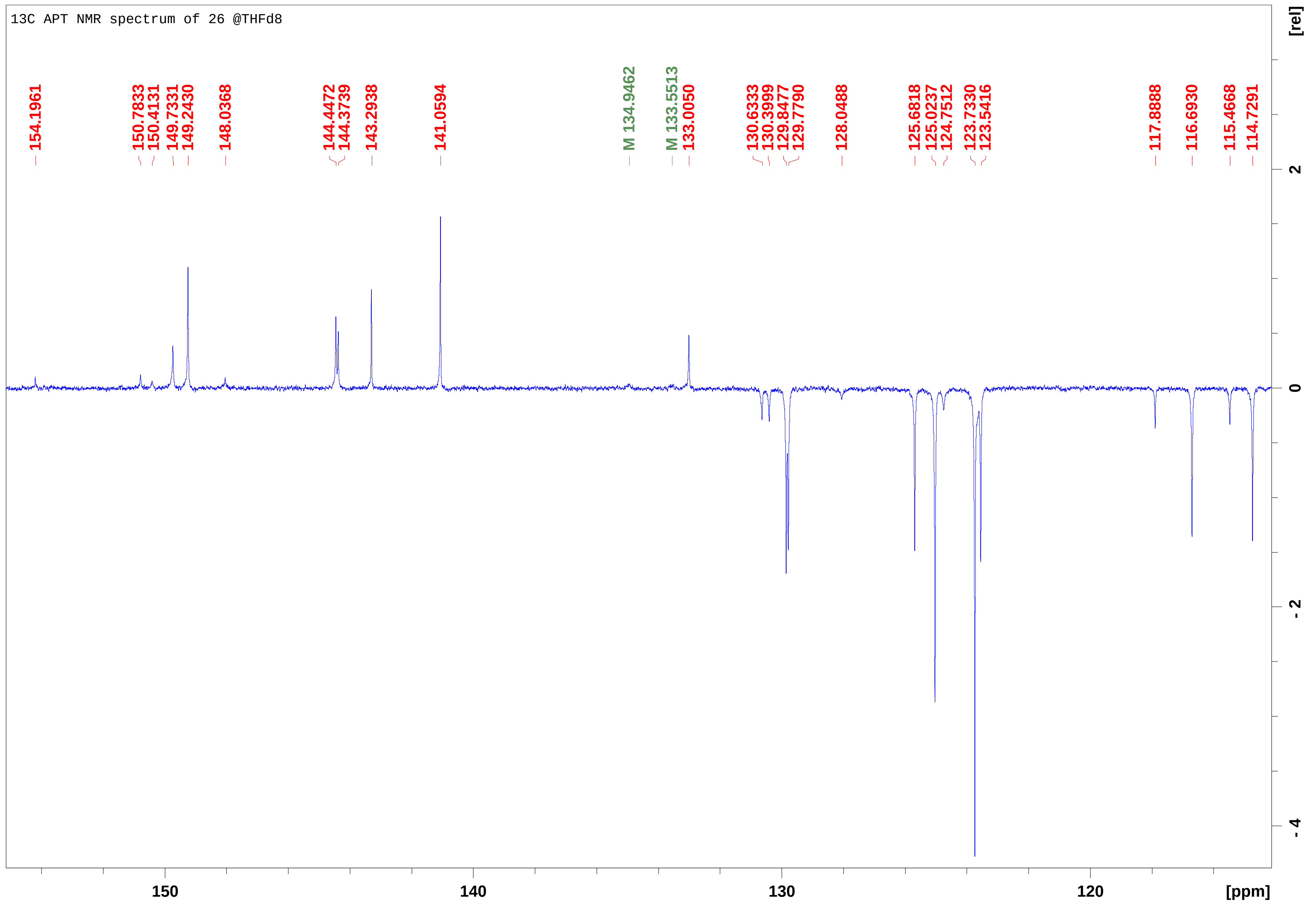

Figure S292. Detail of 13C APT NMR spectrum of 26 in THF-d8

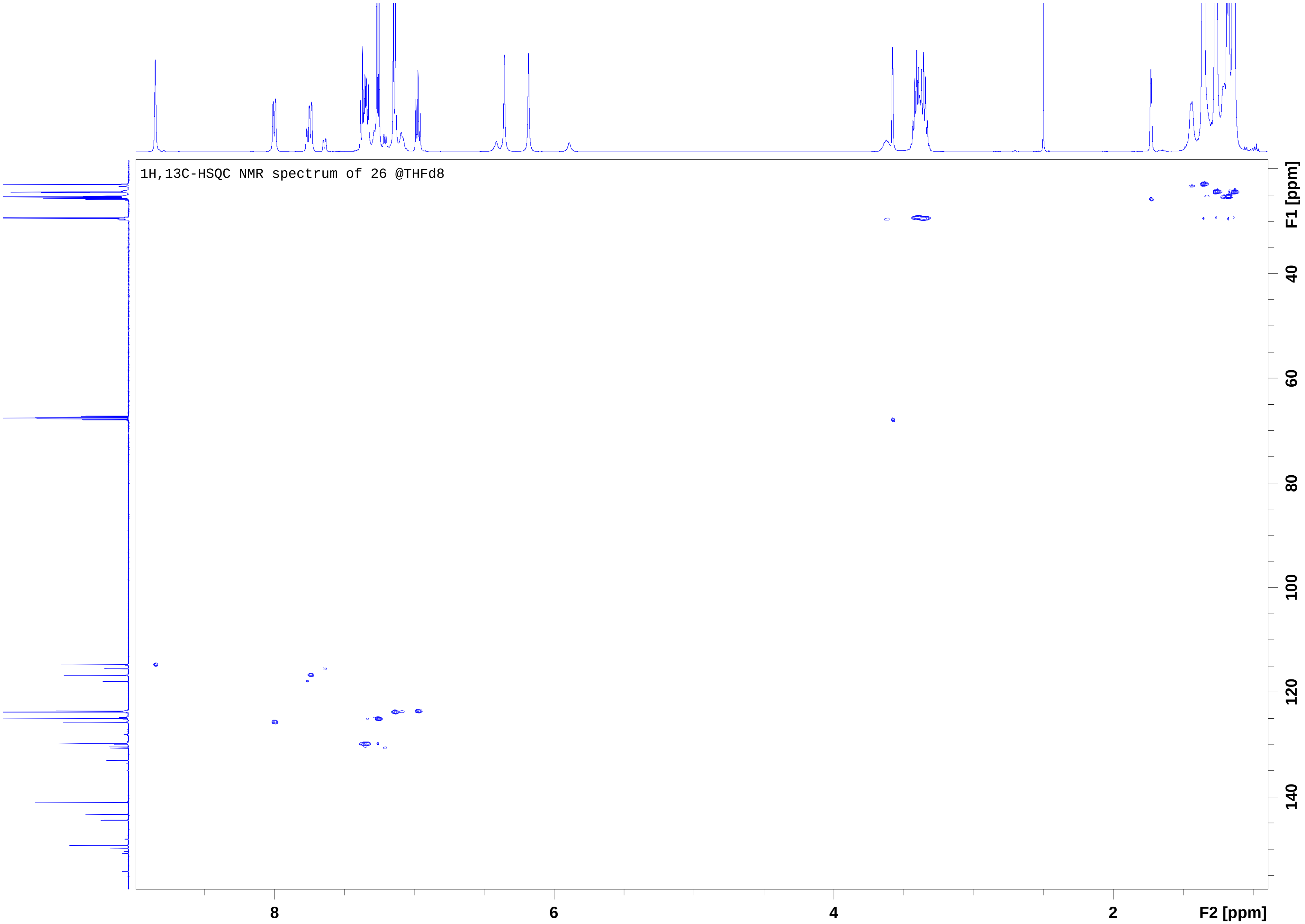

Figure S293. 1H,13C-HSQC NMR spectrum of 26 in THF-d8

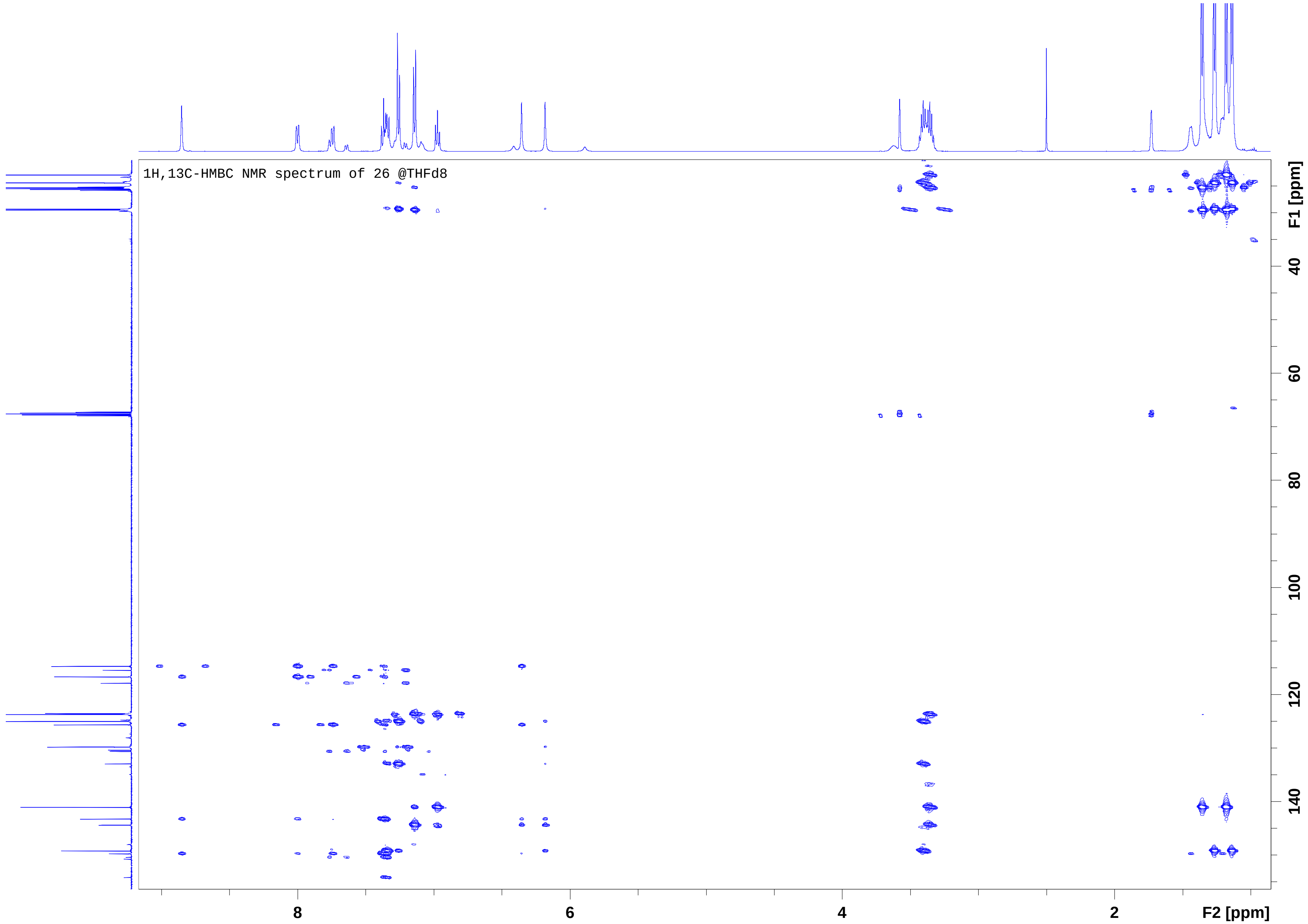

Figure S294. 1H,13C-HMBC NMR spectrum of 26 in THF-d8

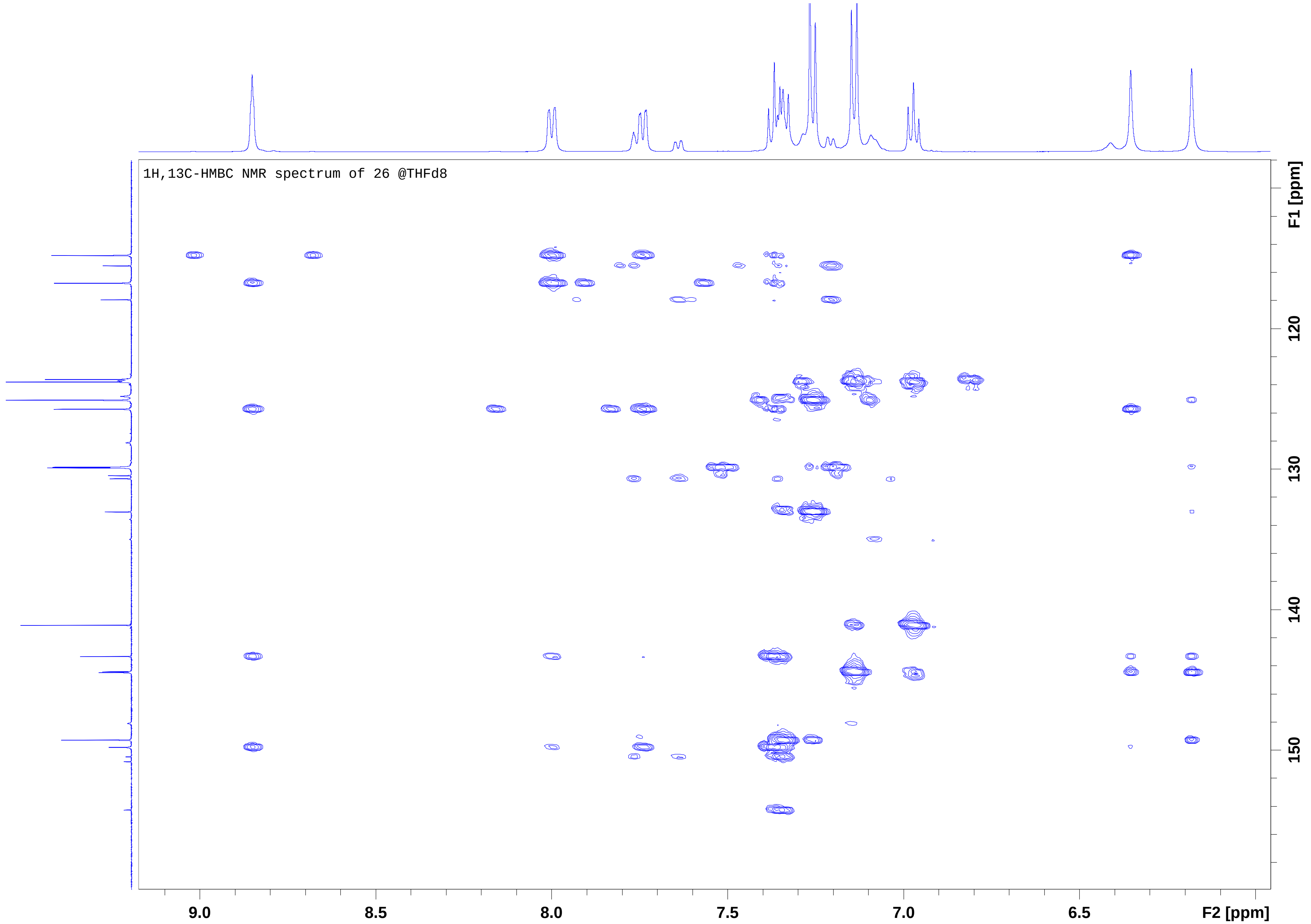

Figure S295. Detail of 1H,13C-HMBC NMR spectrum of 26 in THF-d8

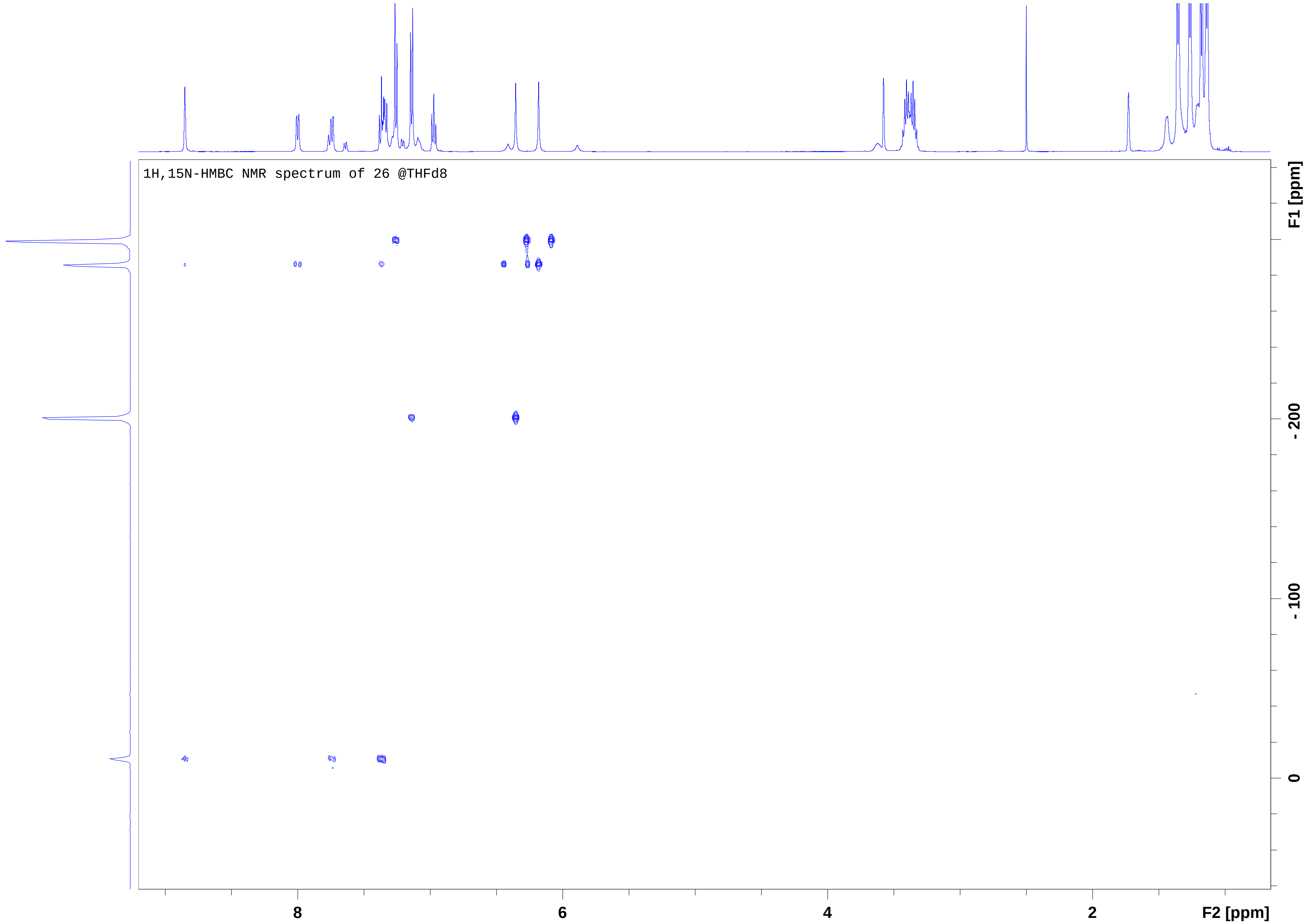

Figure S296. 1H,15N-HMBC NMR spectrum of 26 in THF-d8

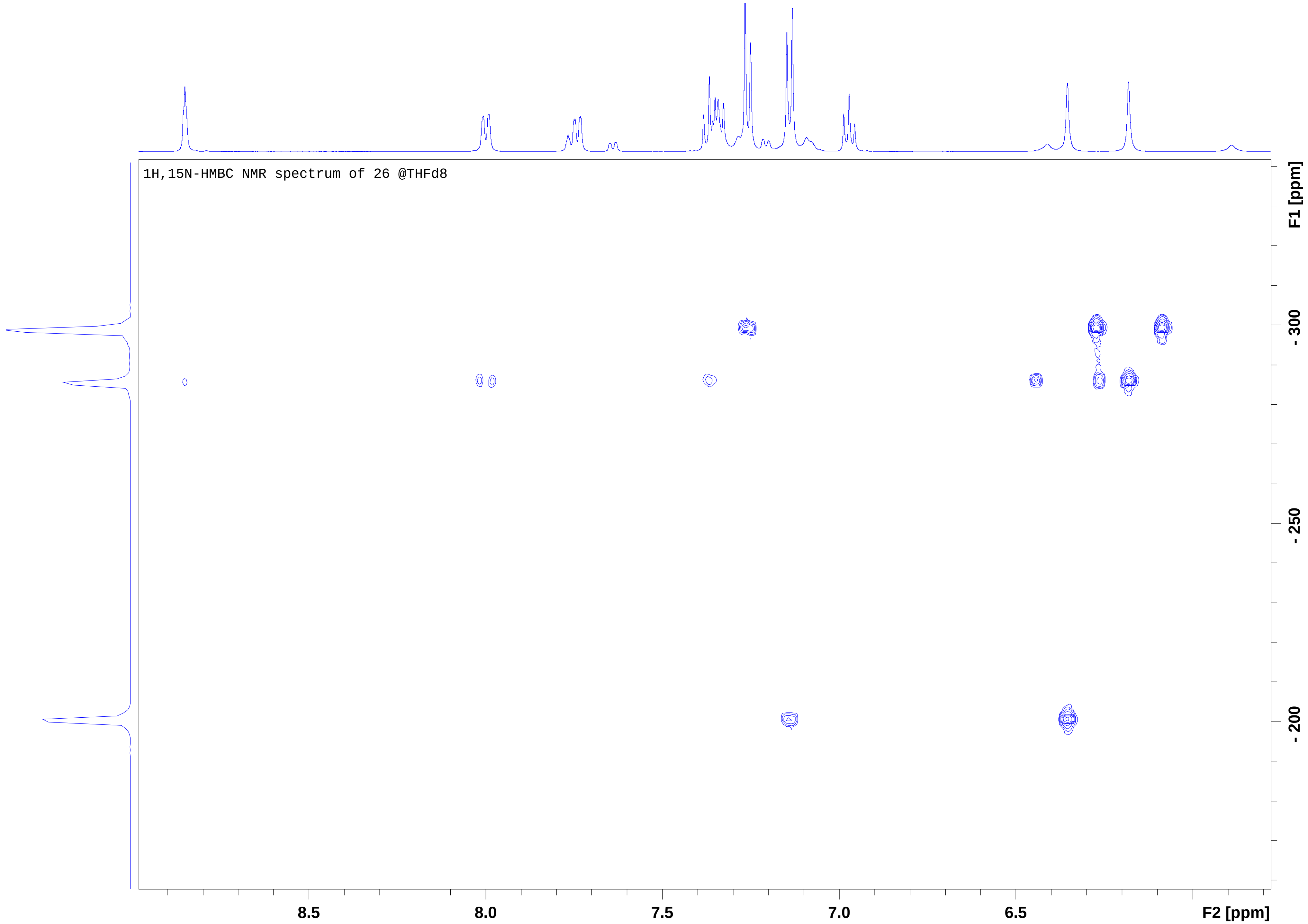

Figure S297. Detail of 1H,15N-HMBC NMR spectrum of 26 in THF-d8

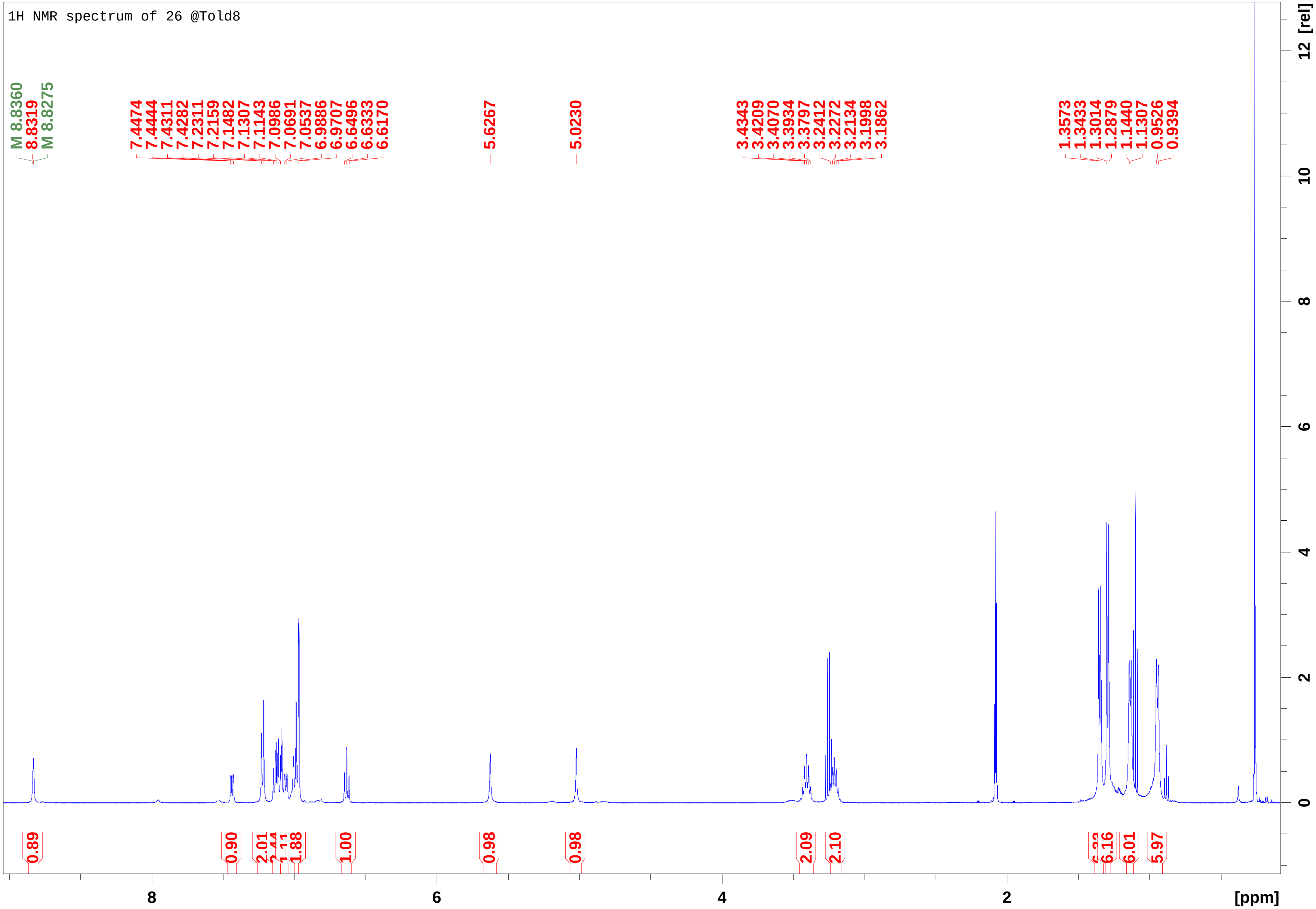

Figure S298. 1H NMR spectrum of 26 in Tol-d8

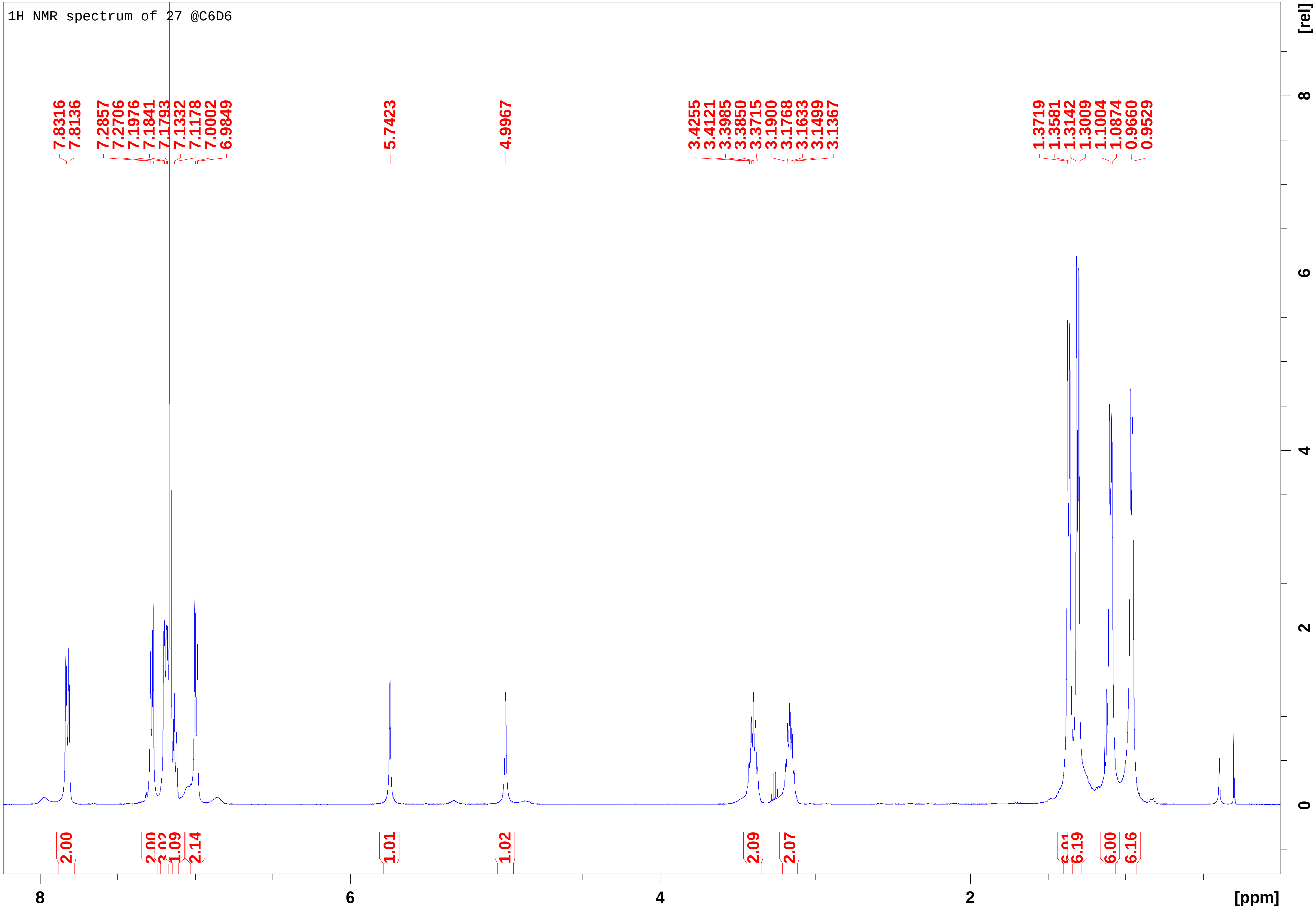

Figure S299. 1H NMR spectrum of 27 in C6D6

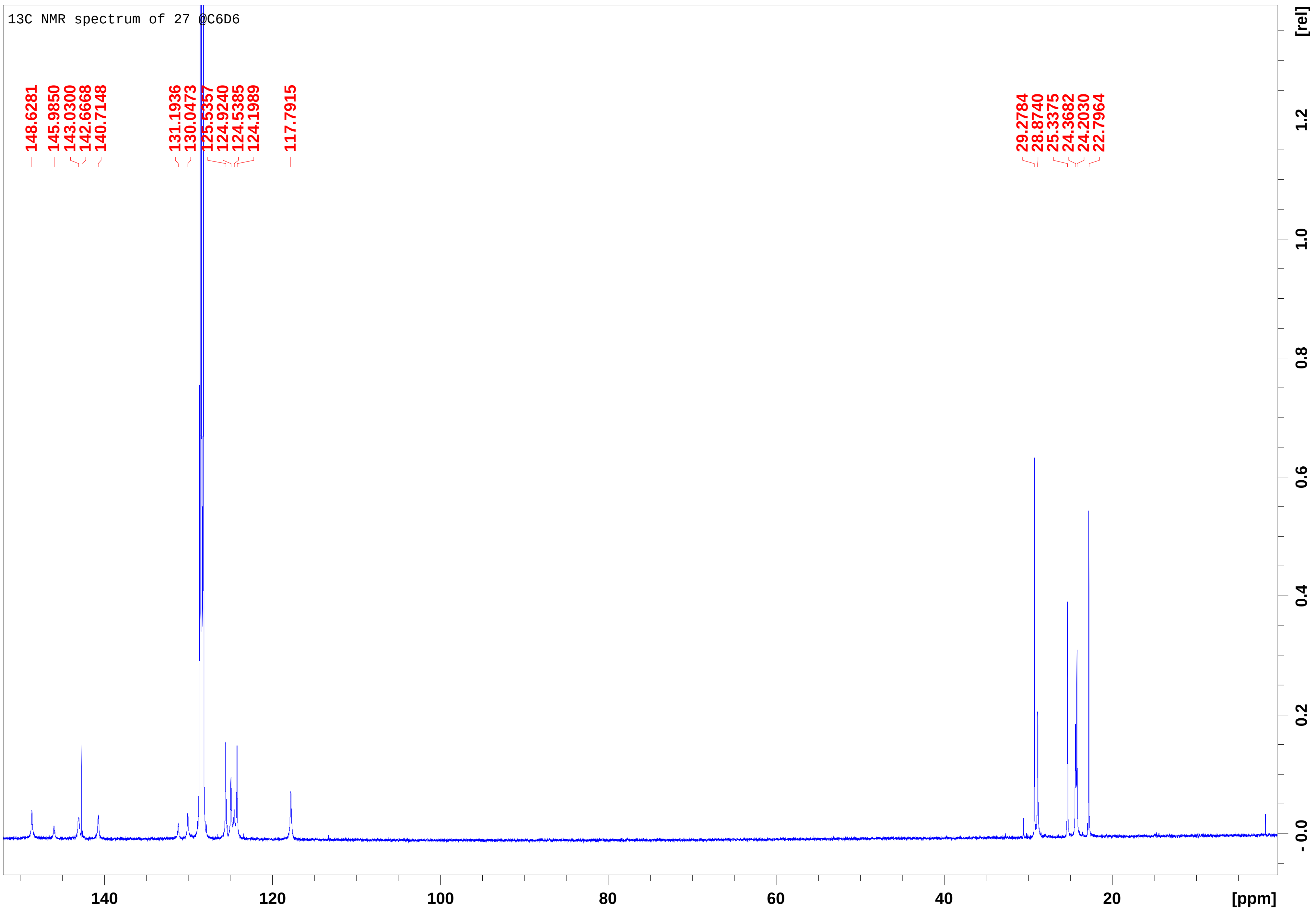

Figure S300. 13C NMR spectrum of 27 in C6D6

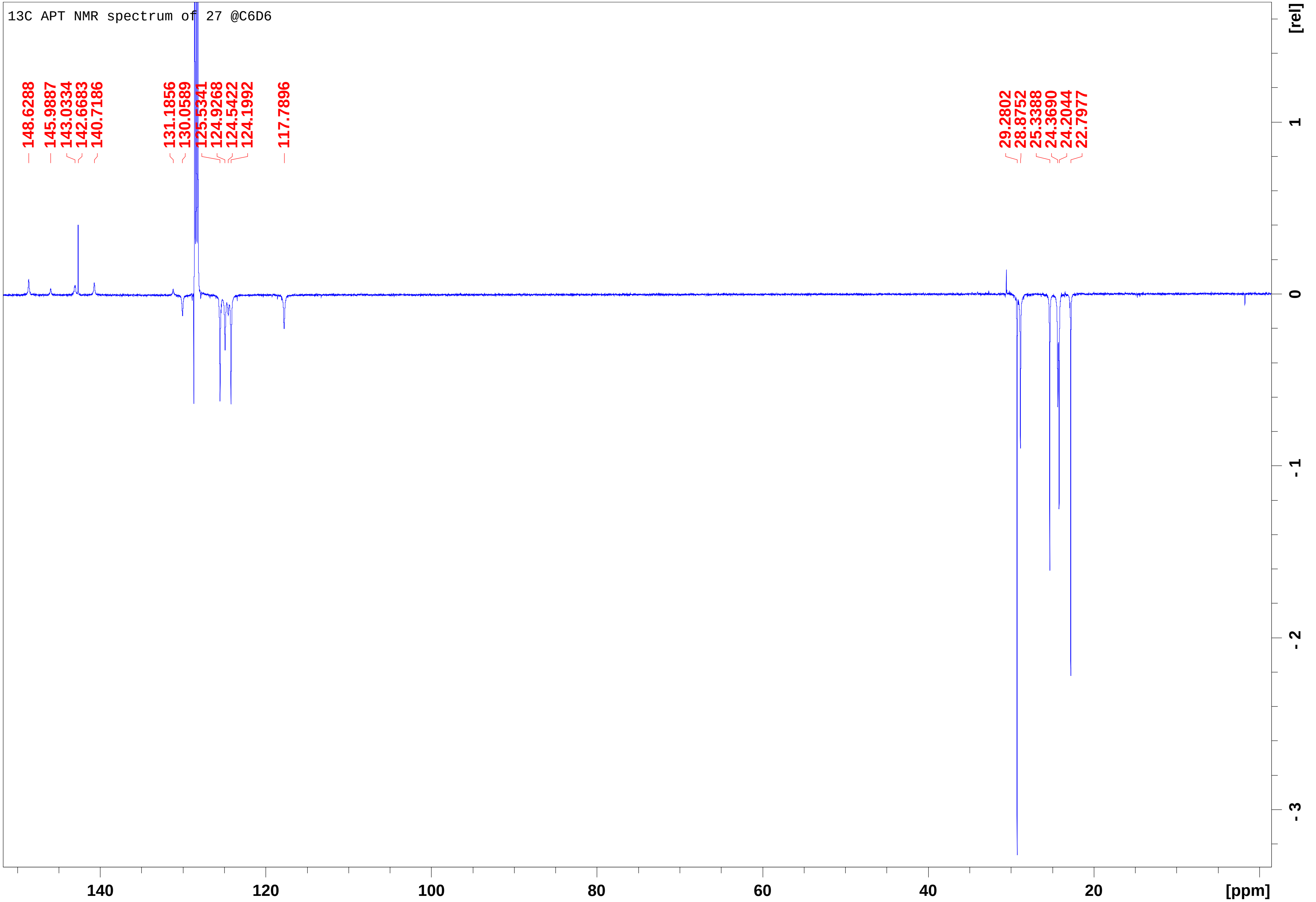

Figure S301. 13C APT NMR spectrum of 27 in C6D6

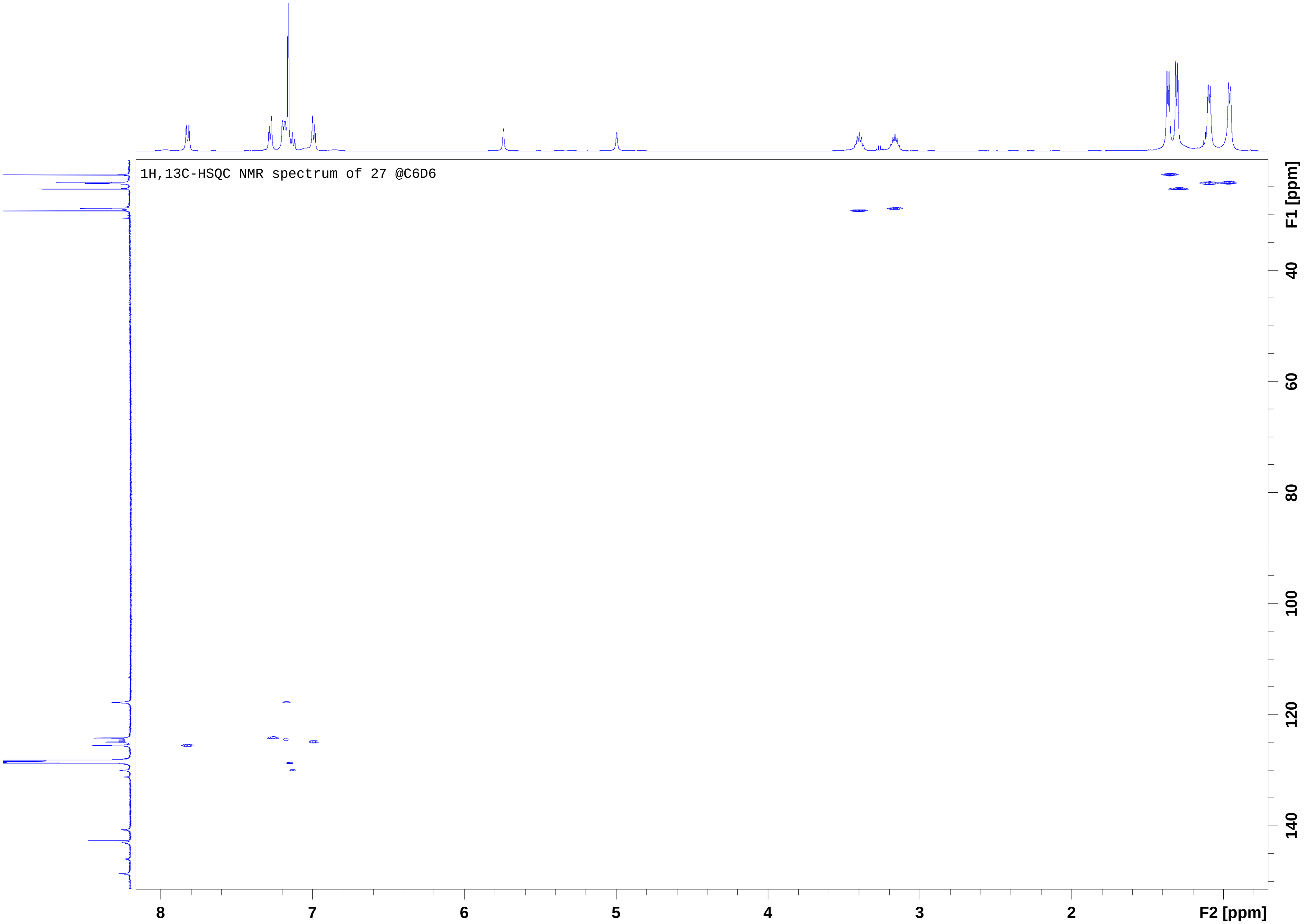

Figure S302. 1H,13C-HSQC NMR spectrum of 27 in C6D6

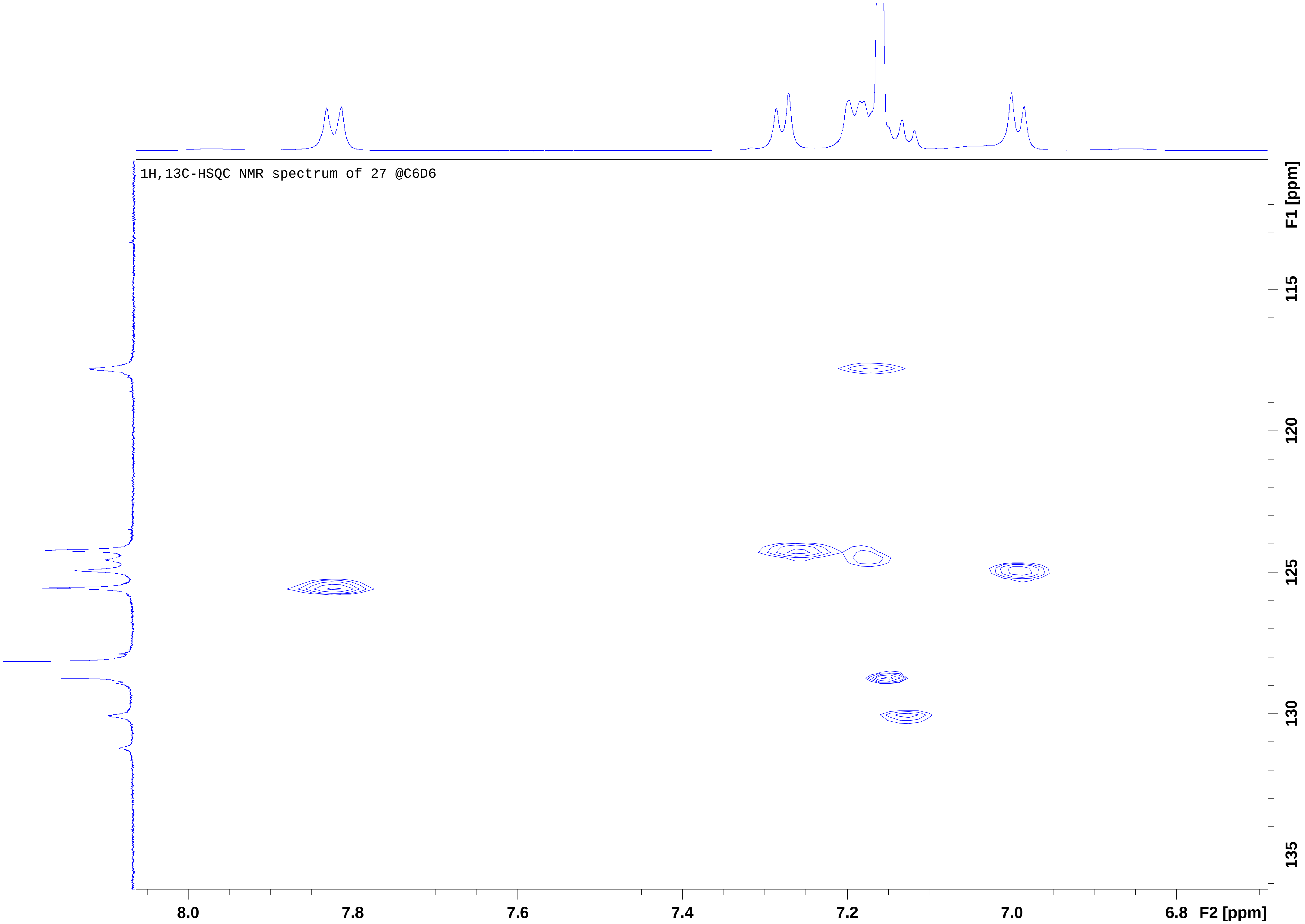

Figure S303. Detail of 1H,13C-HSQC NMR spectrum of 27 in C6D6

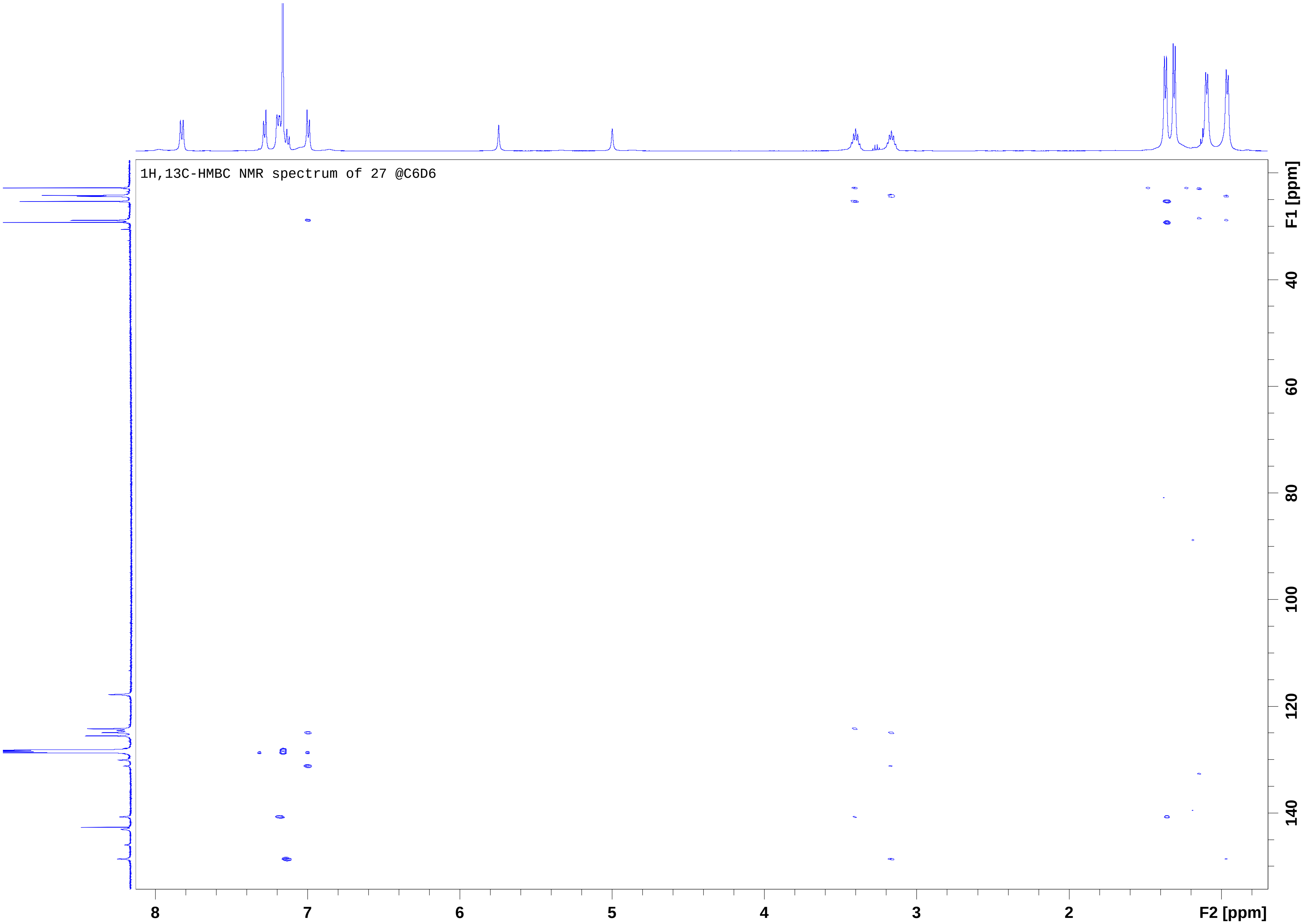

Figure S304. 1H,13C-HMBC NMR spectrum of 27 in C6D6

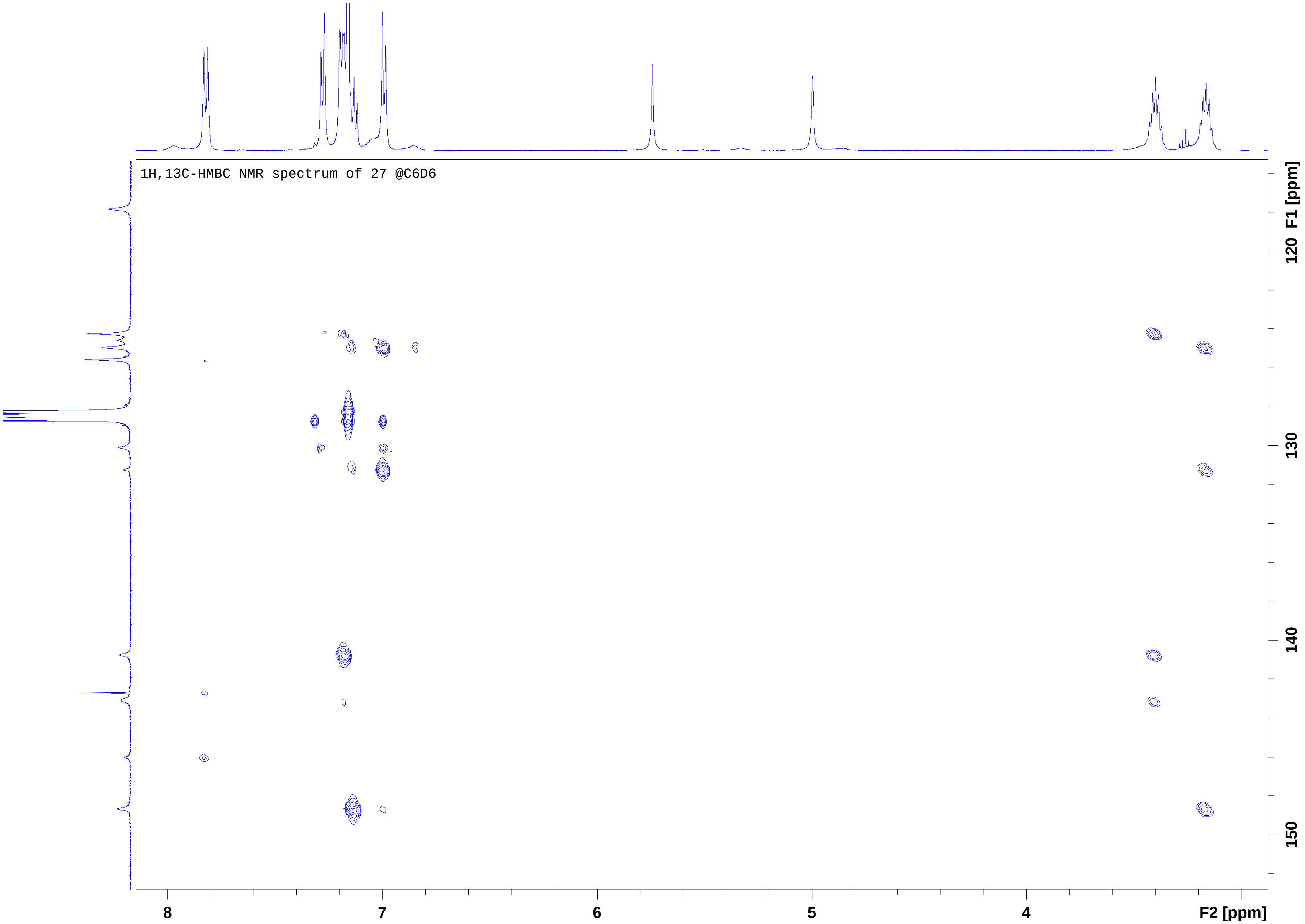

Figure S305. Detail of 1H,13C-HMBC NMR spectrum of 27 in C6D6

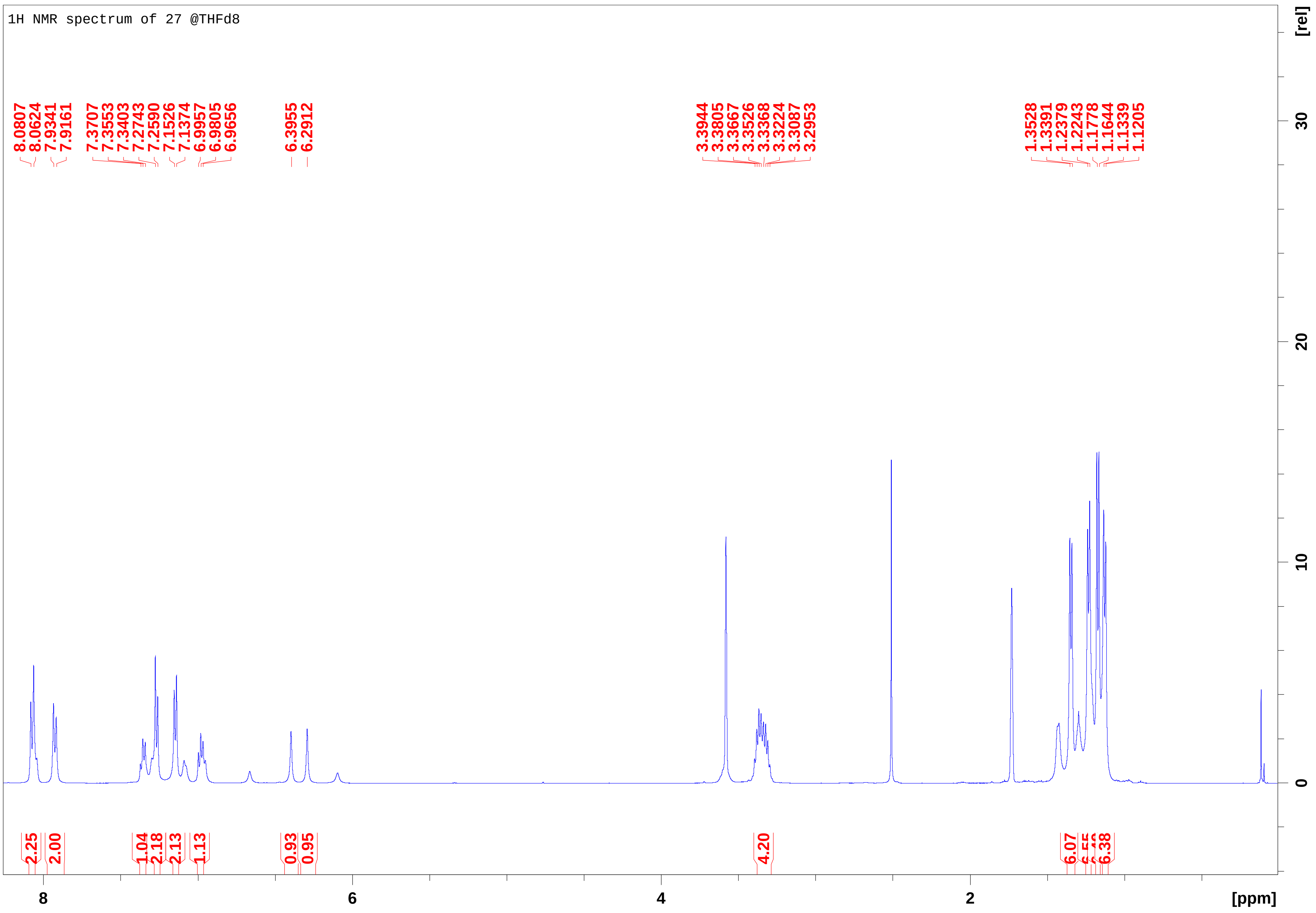

Figure S306. 1H NMR spectrum of 27 in THF-d8

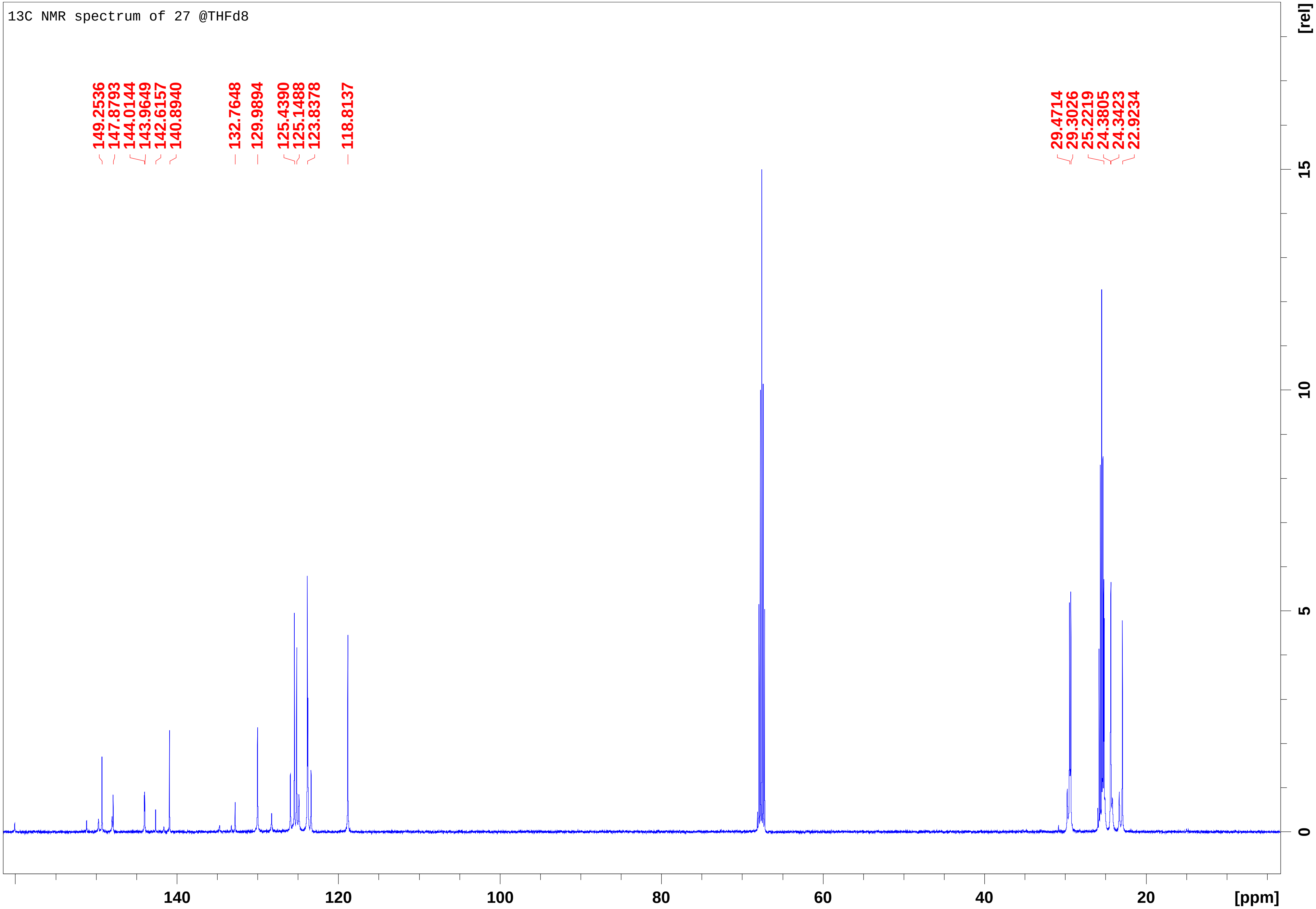

Figure S307. 13C NMR spectrum of 27 in THF-d8

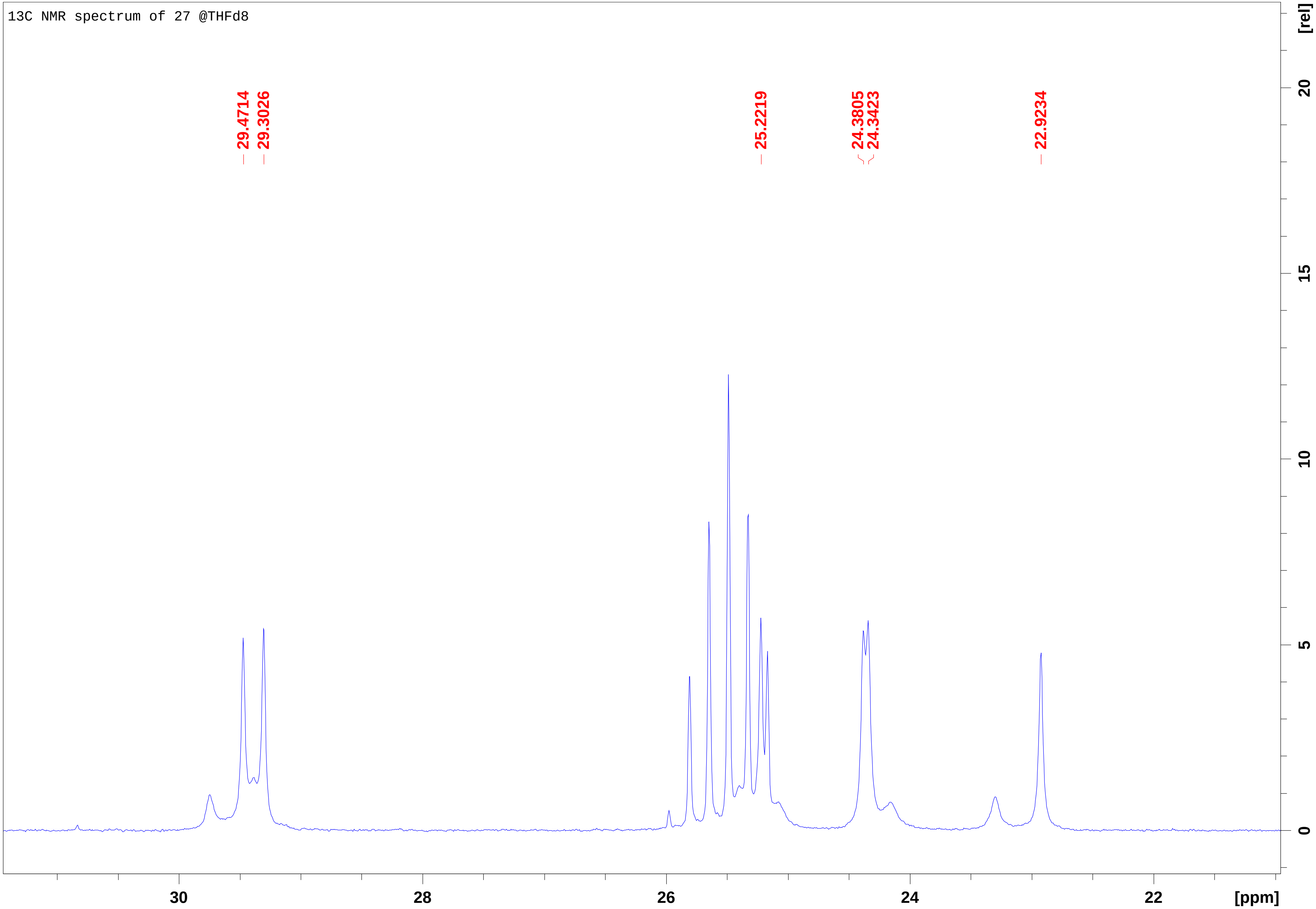

Figure S308. Detail of 13C NMR spectrum of 27 in THF-d8

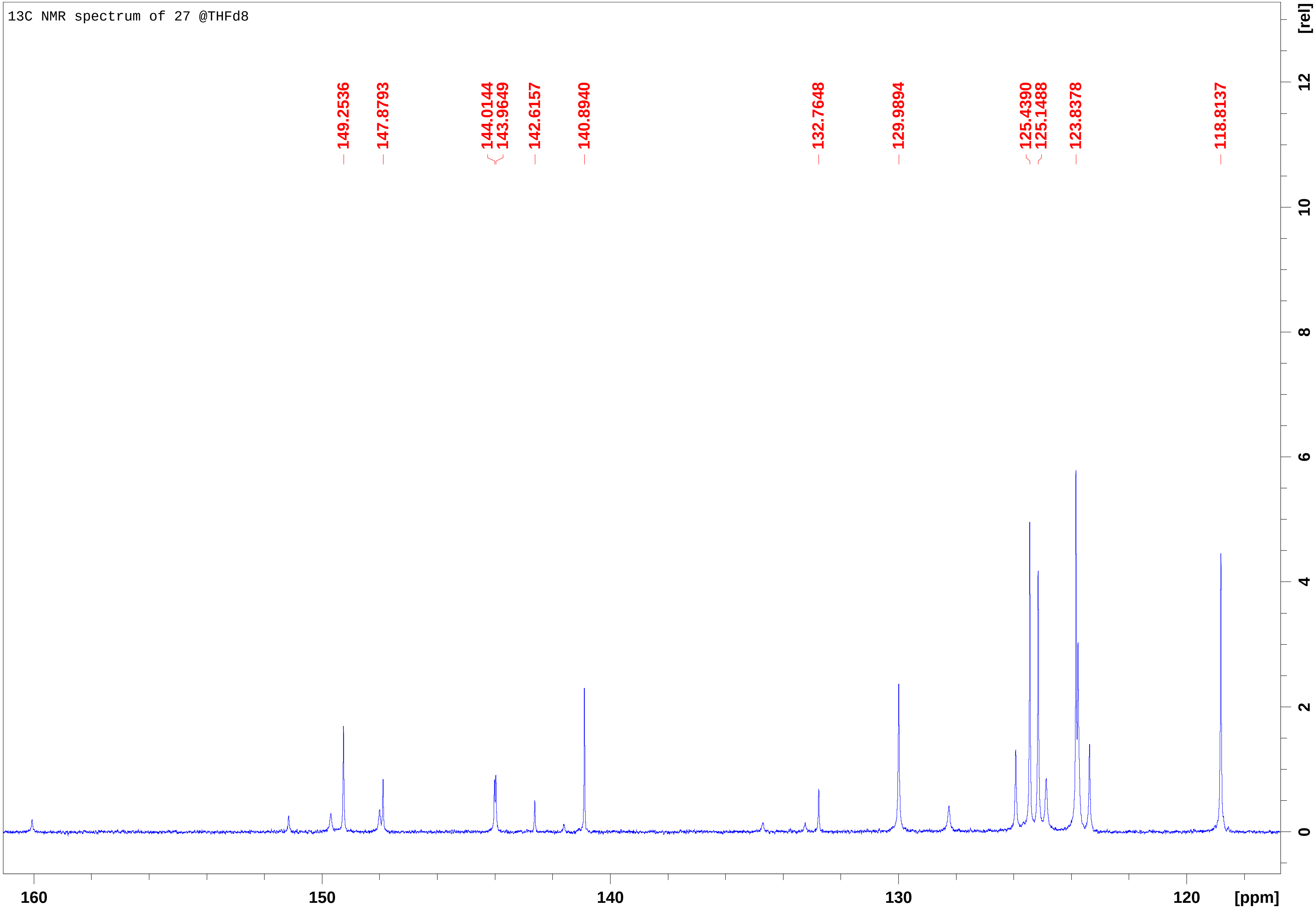

Figure S309. 13C NMR spectrum of 27 in THF-d8

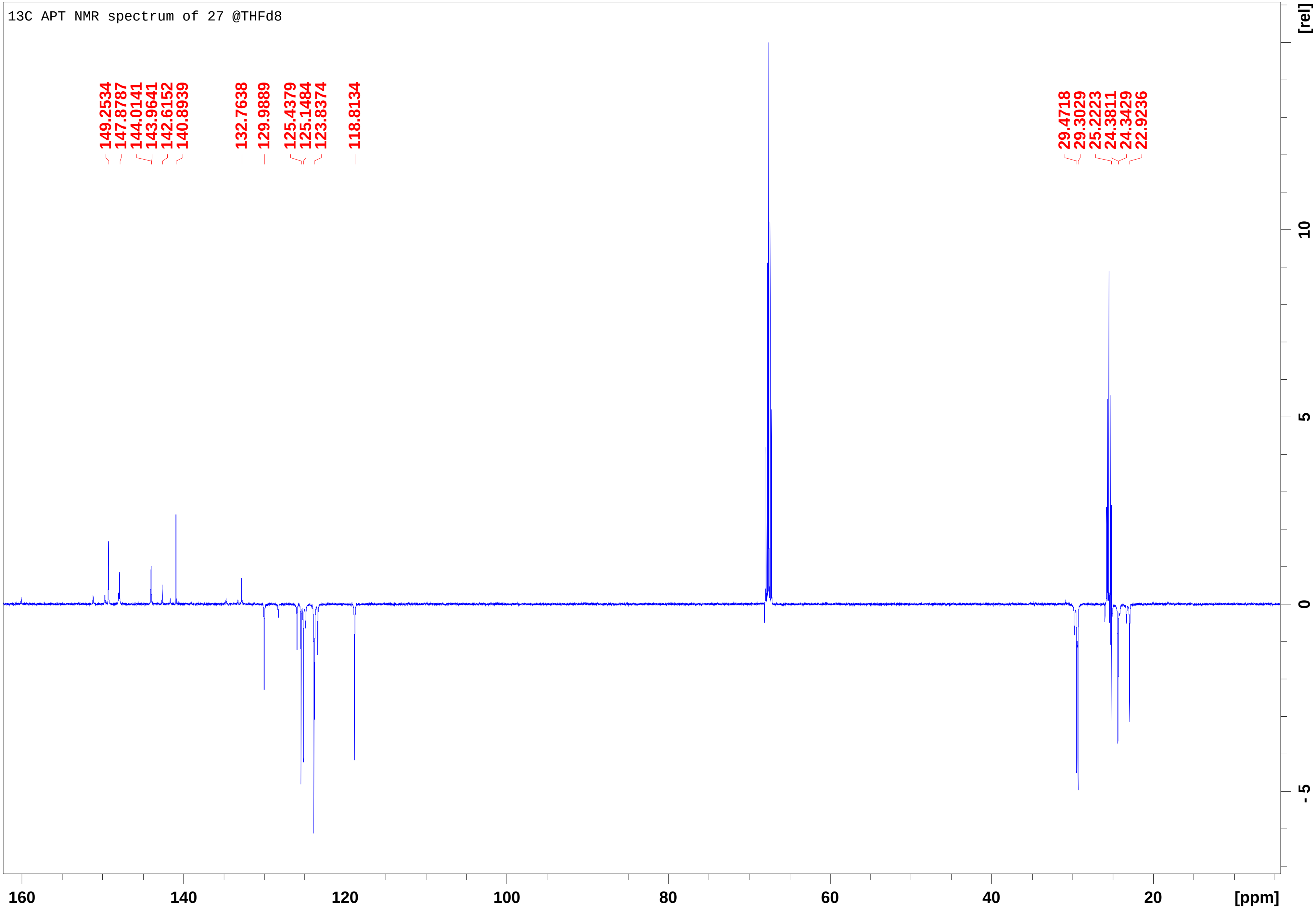

Figure S310. 13C APT NMR spectrum of 27 in THF-d8

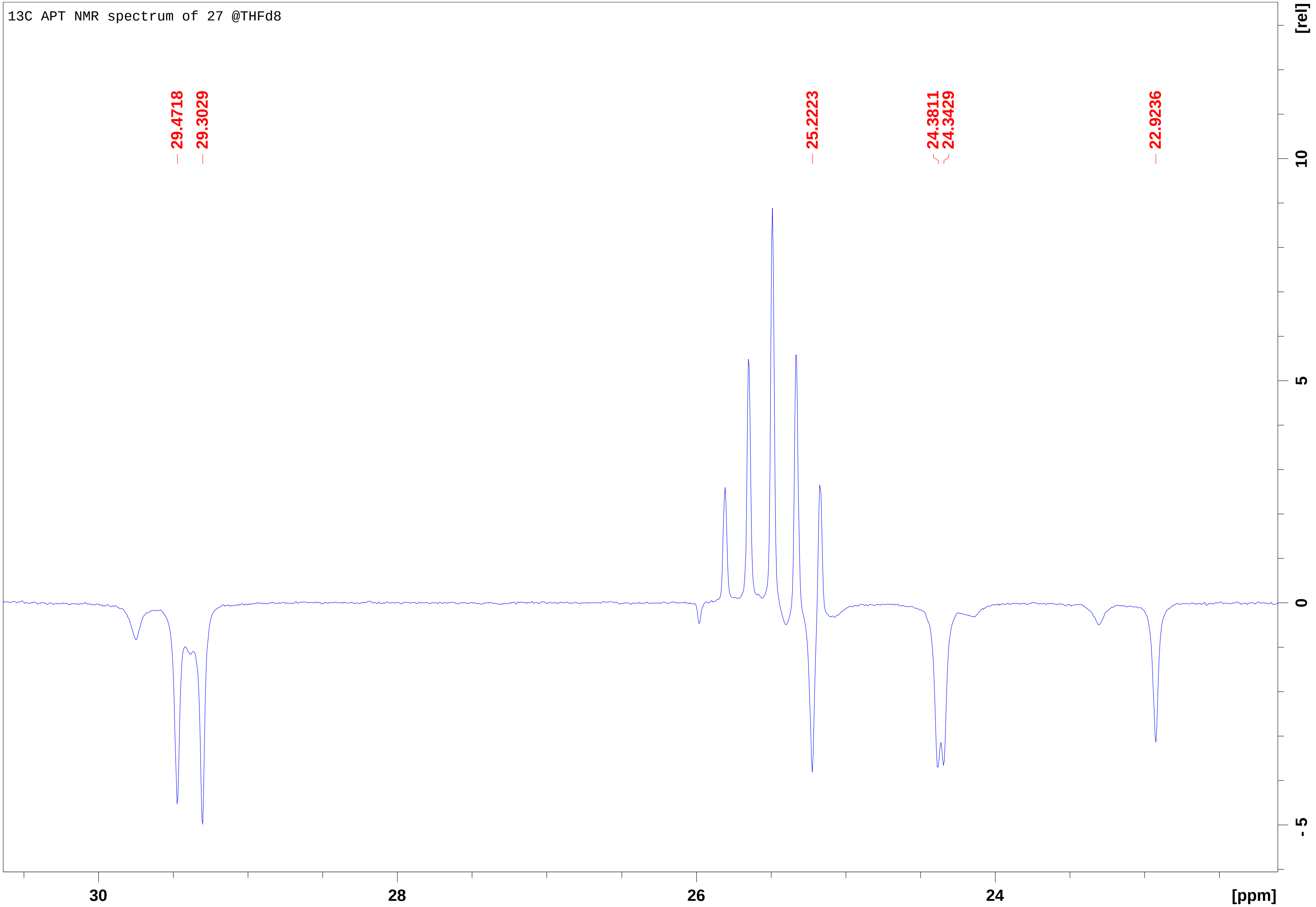

Figure S311. Detail of 13C APT NMR spectrum of 27 in THF-d8

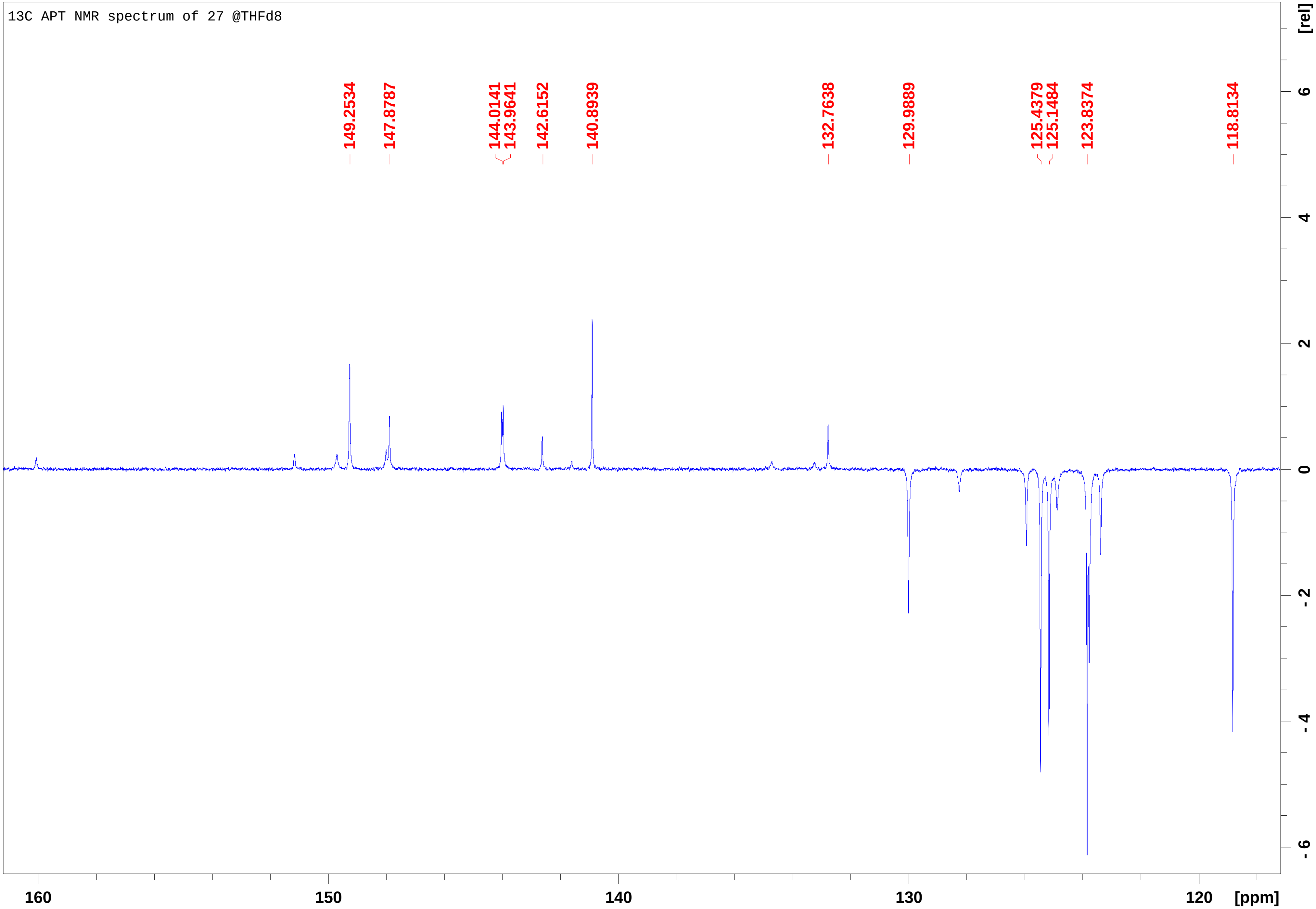

Figure S312. Detail of 13C APT NMR spectrum of 27 in THF-d8

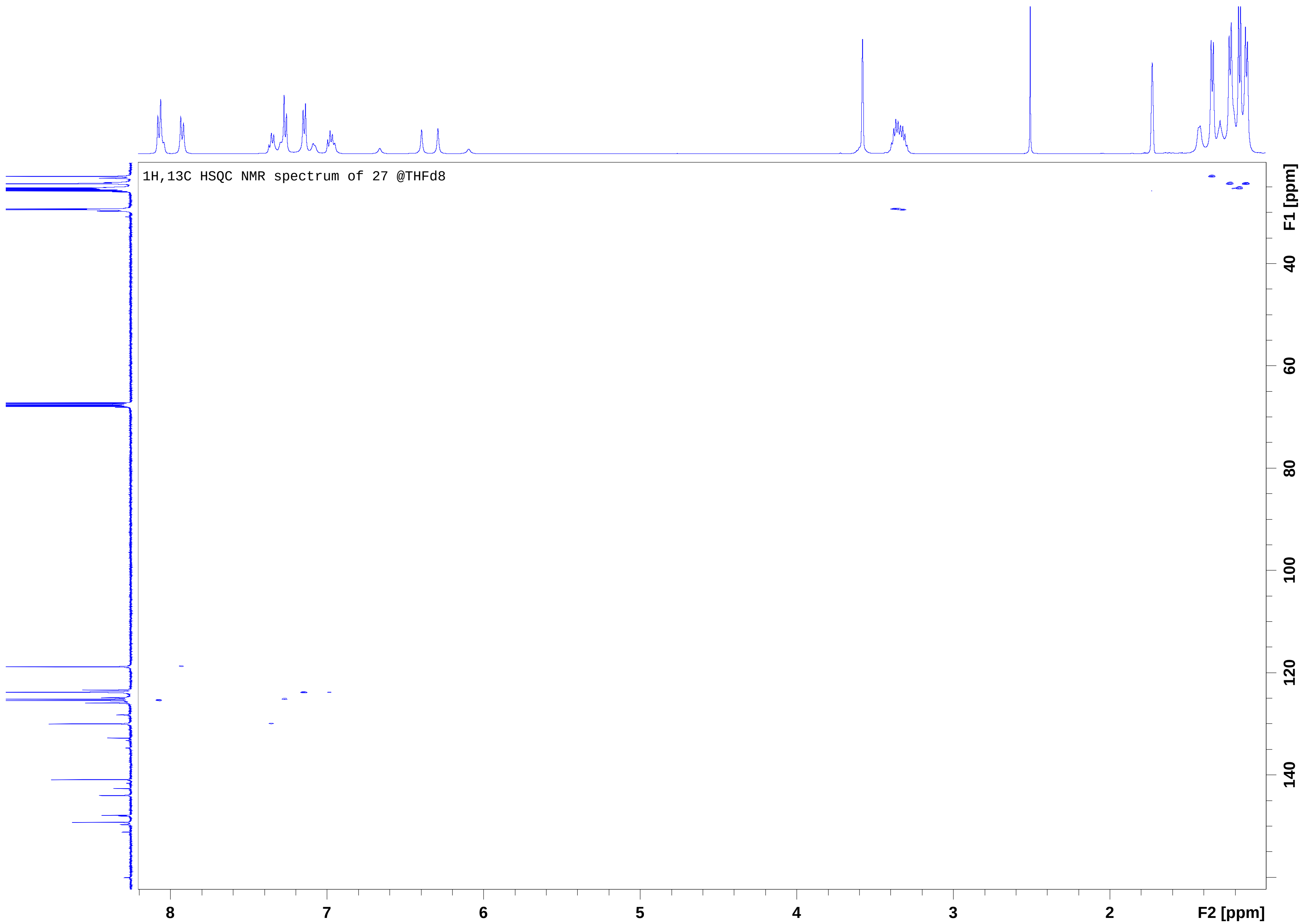

Figure S313. 1H,13C-HSQC NMR spectrum of 27 in THF-d8

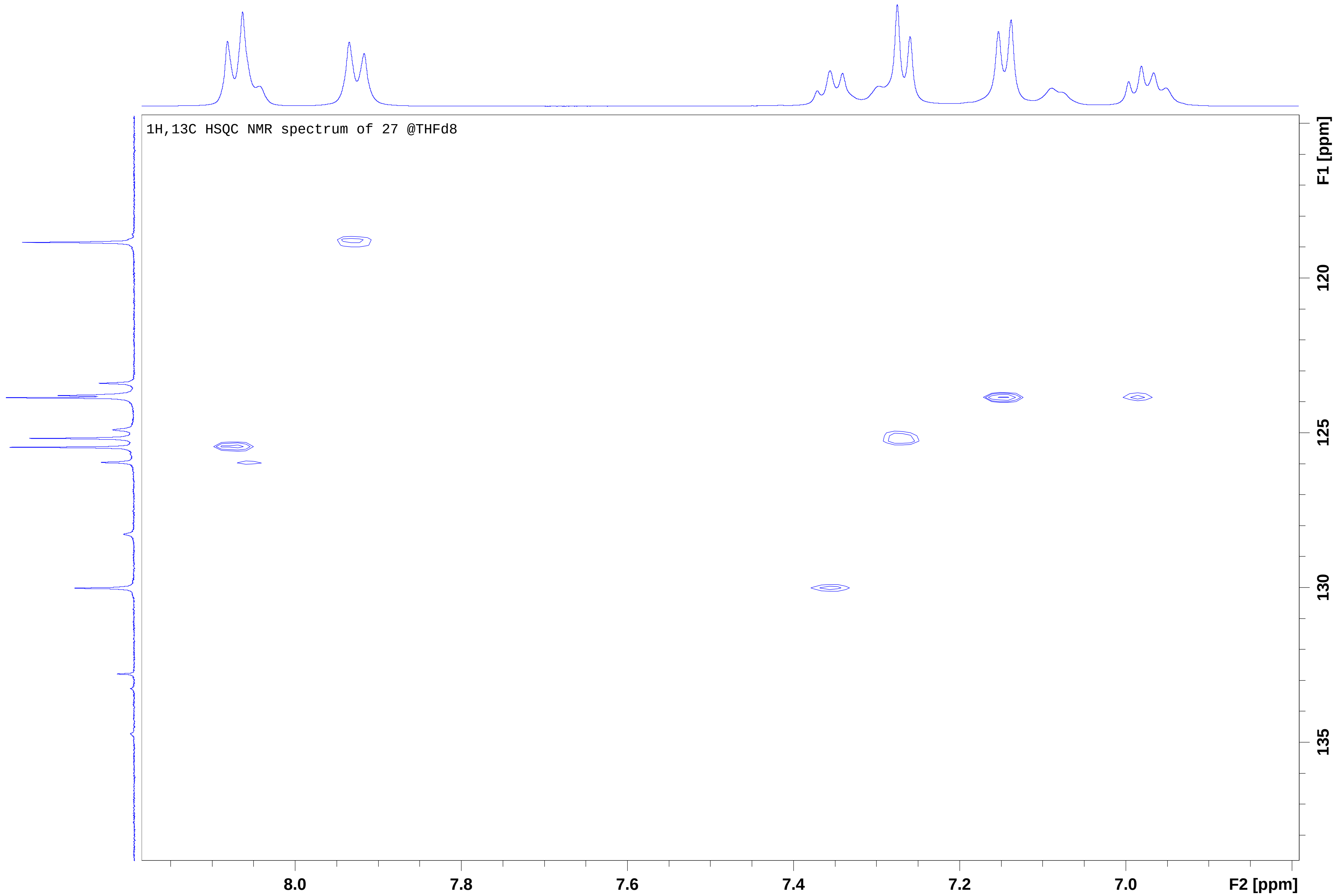

Figure S314. Detail of 1H,13C-HSQC NMR spectrum of 27 in THF-d8

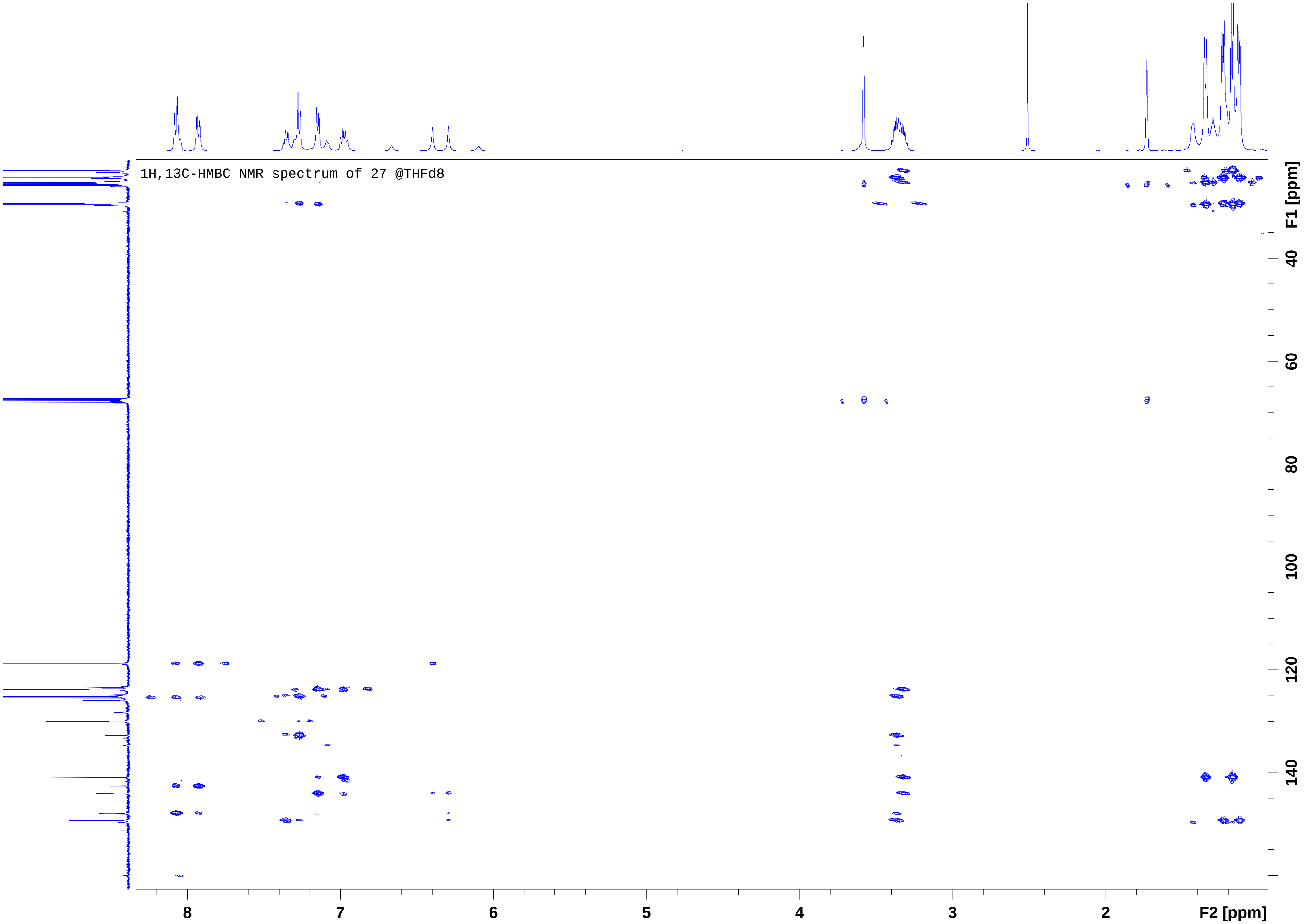

Figure S315. 1H,13C-HMBC NMR spectrum of 27 in THF-d8

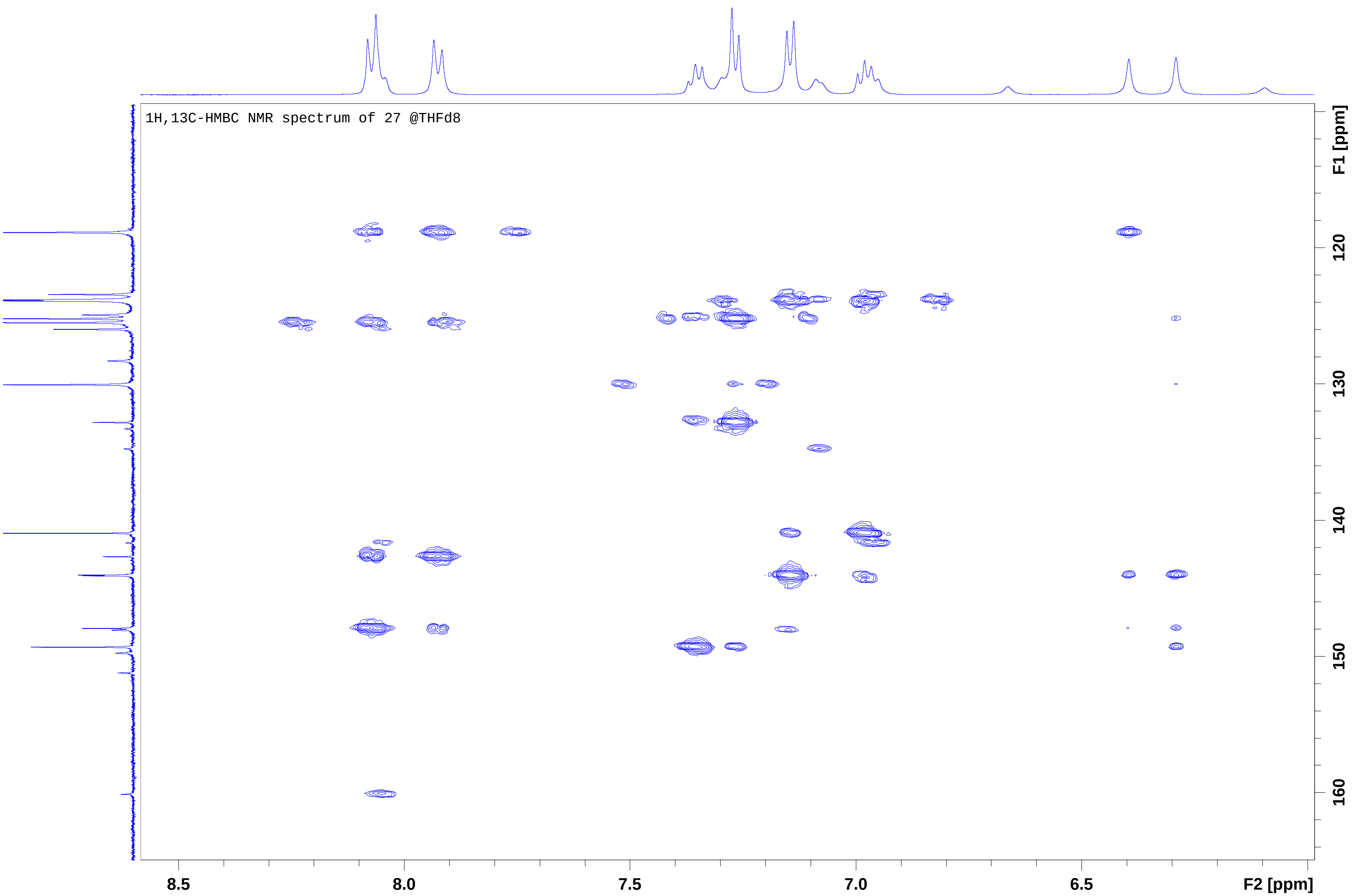

Figure S316. Detail of 1H,13C-HMBC NMR spectrum of 27 in THF-d8

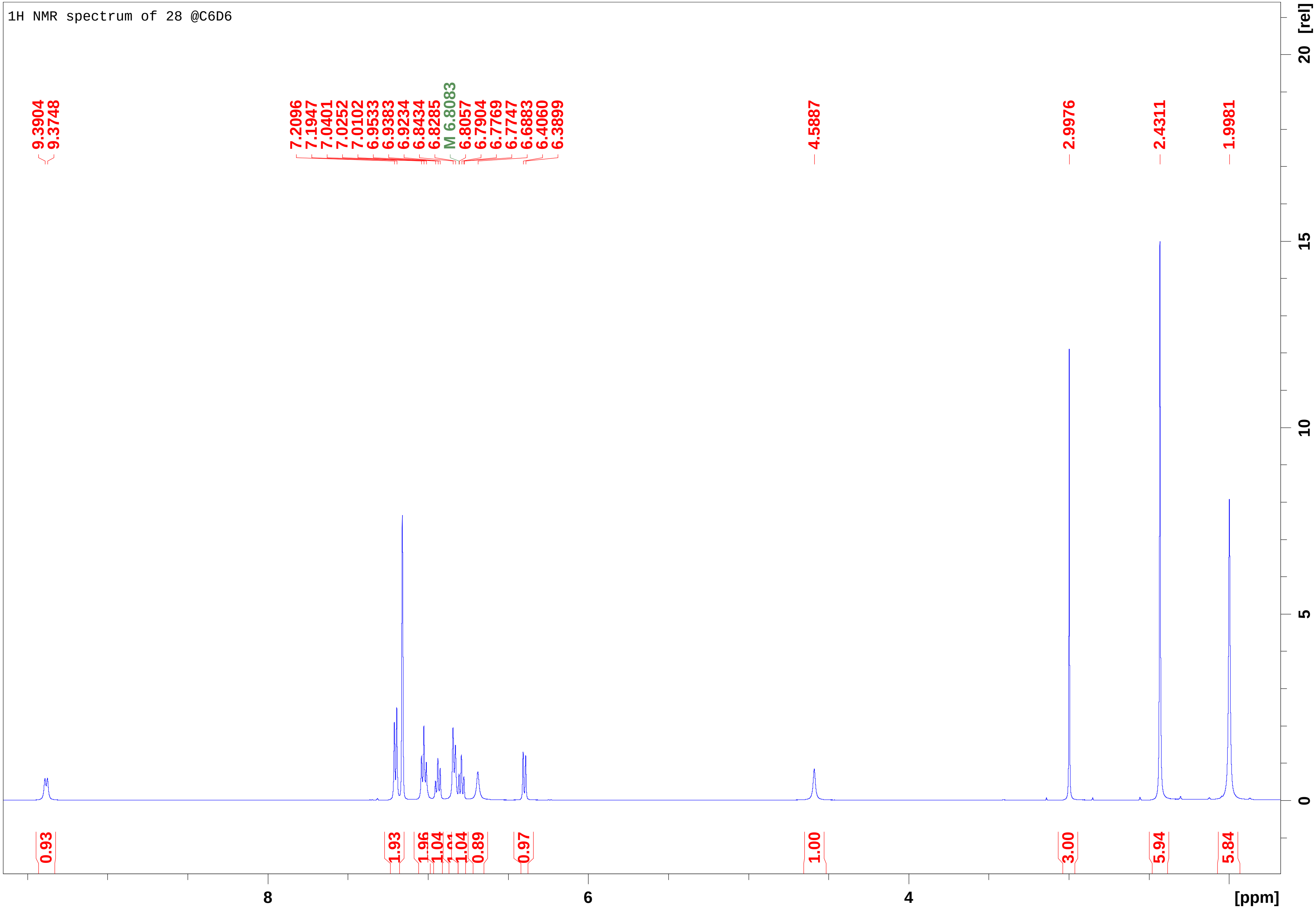

Figure S317. 1H NMR spectrum of 28 in C6D6

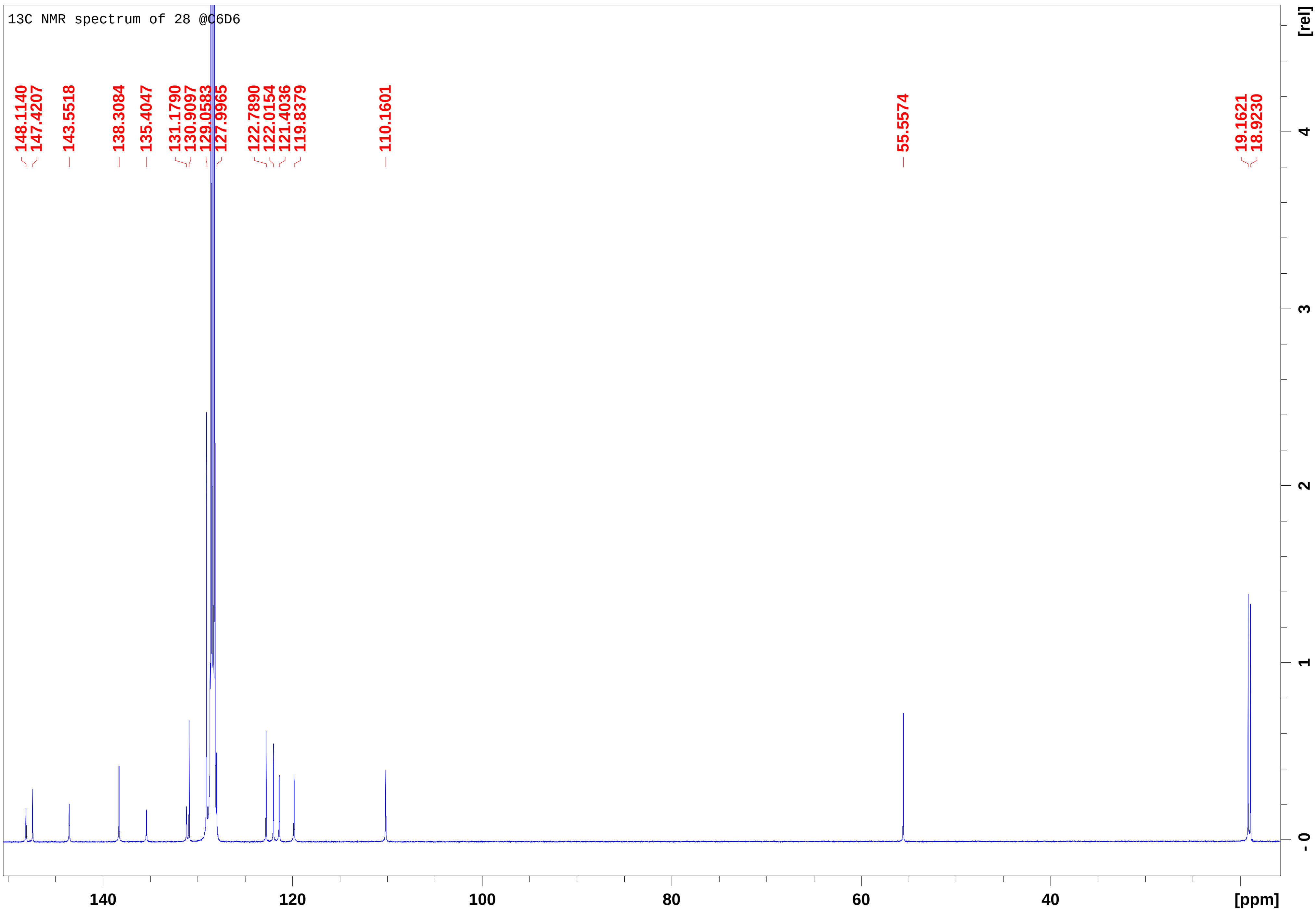

Figure S318. 13C NMR spectrum of 28 in C6D6

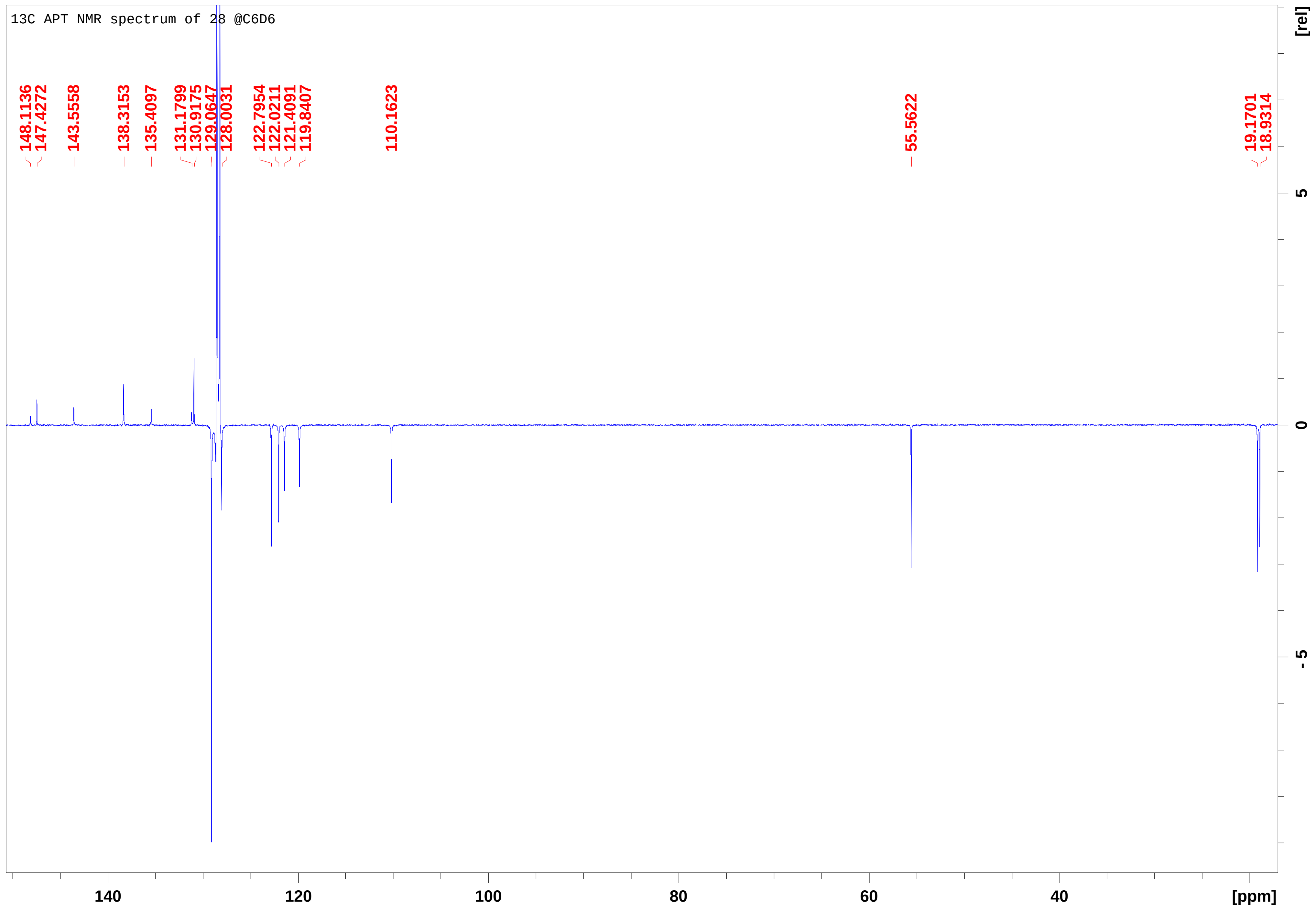

Figure S319. 13C APT NMR spectrum of 28 in C6D6

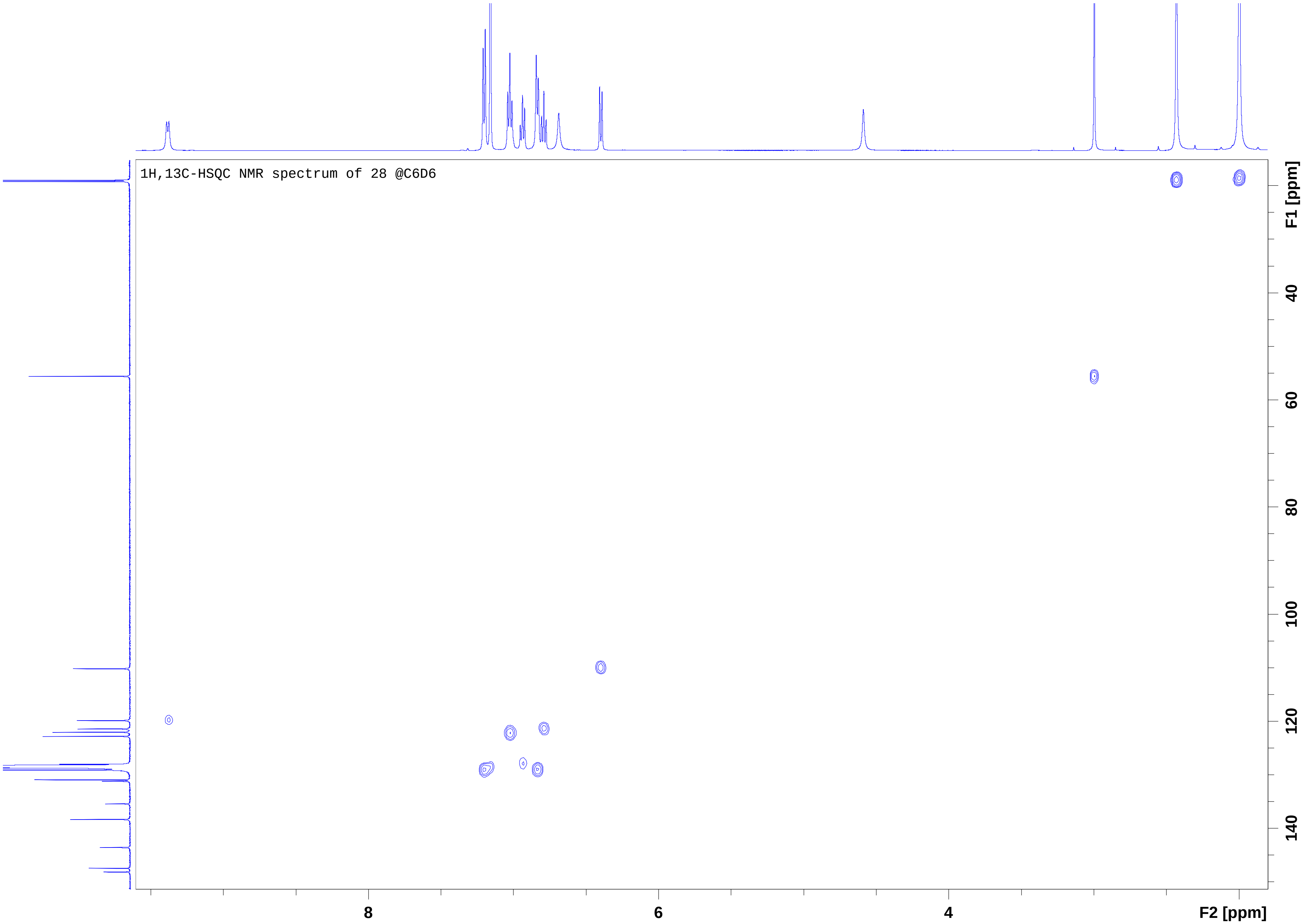

Figure S320. 1H,13C-HSQC NMR spectrum of 28 in C6D6

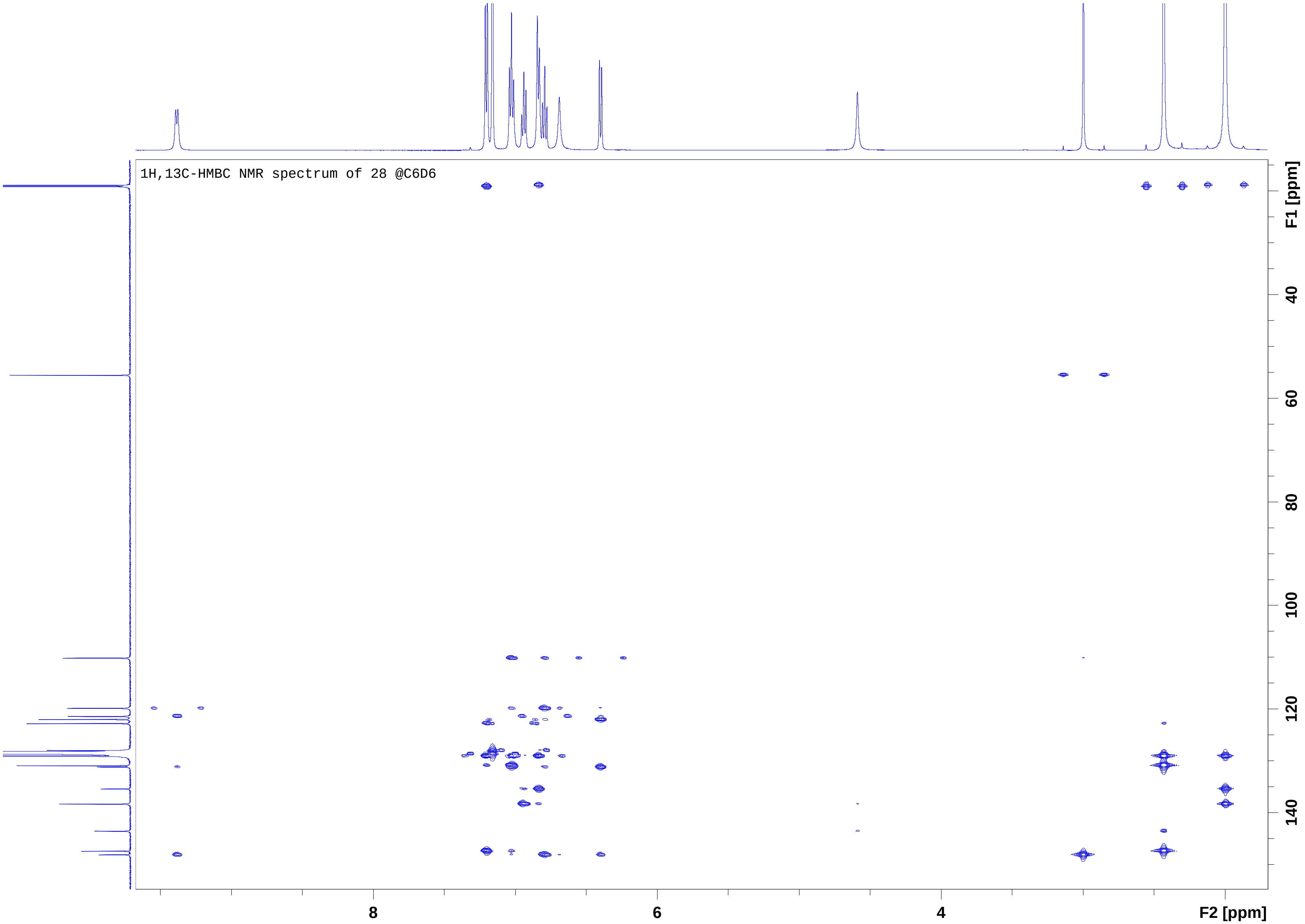

Figure S321. 1H,13C-HMBC NMR spectrum of 28 in C6D6

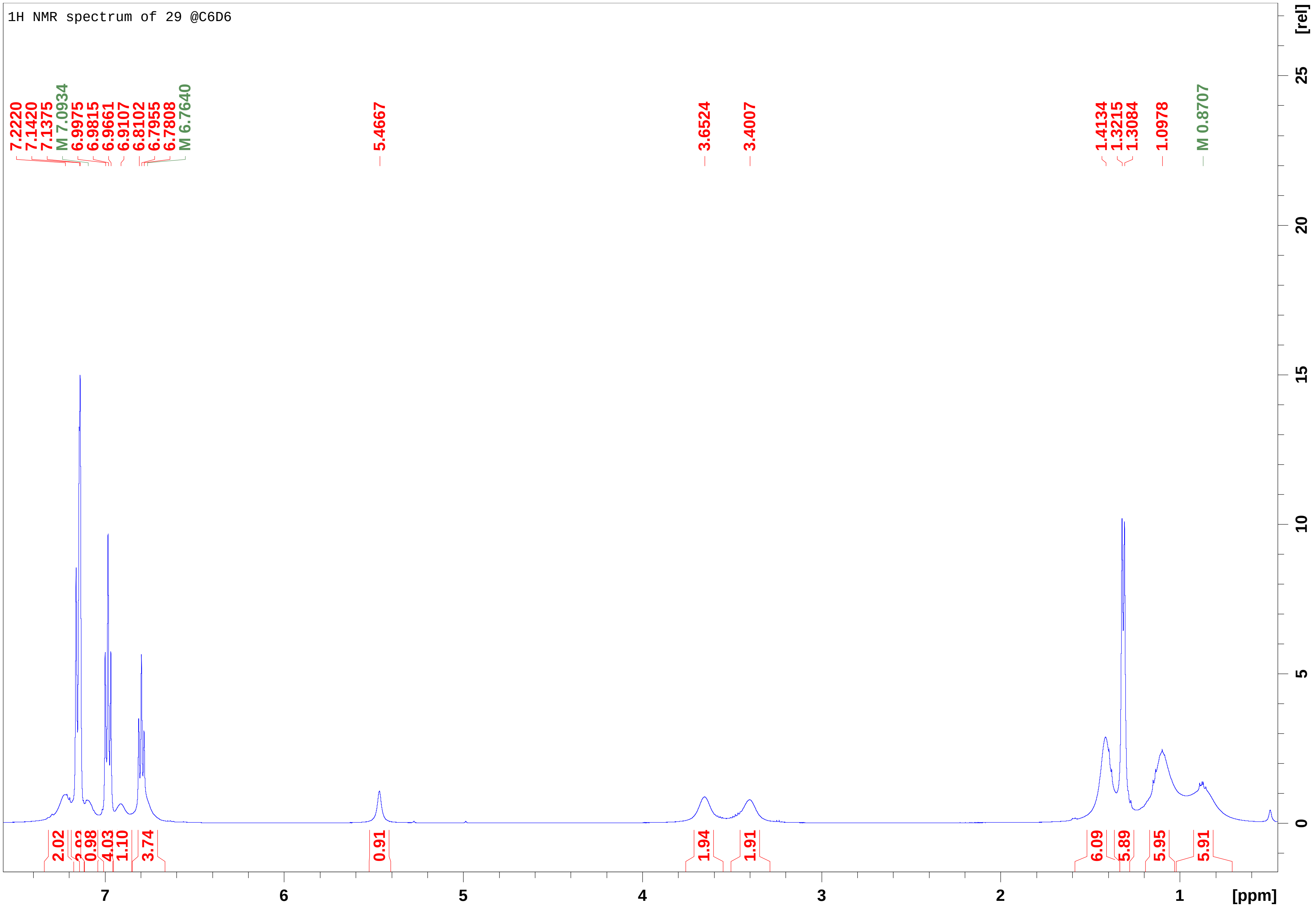

Figure S322. 1H NMR spectrum of 29 in C6D6

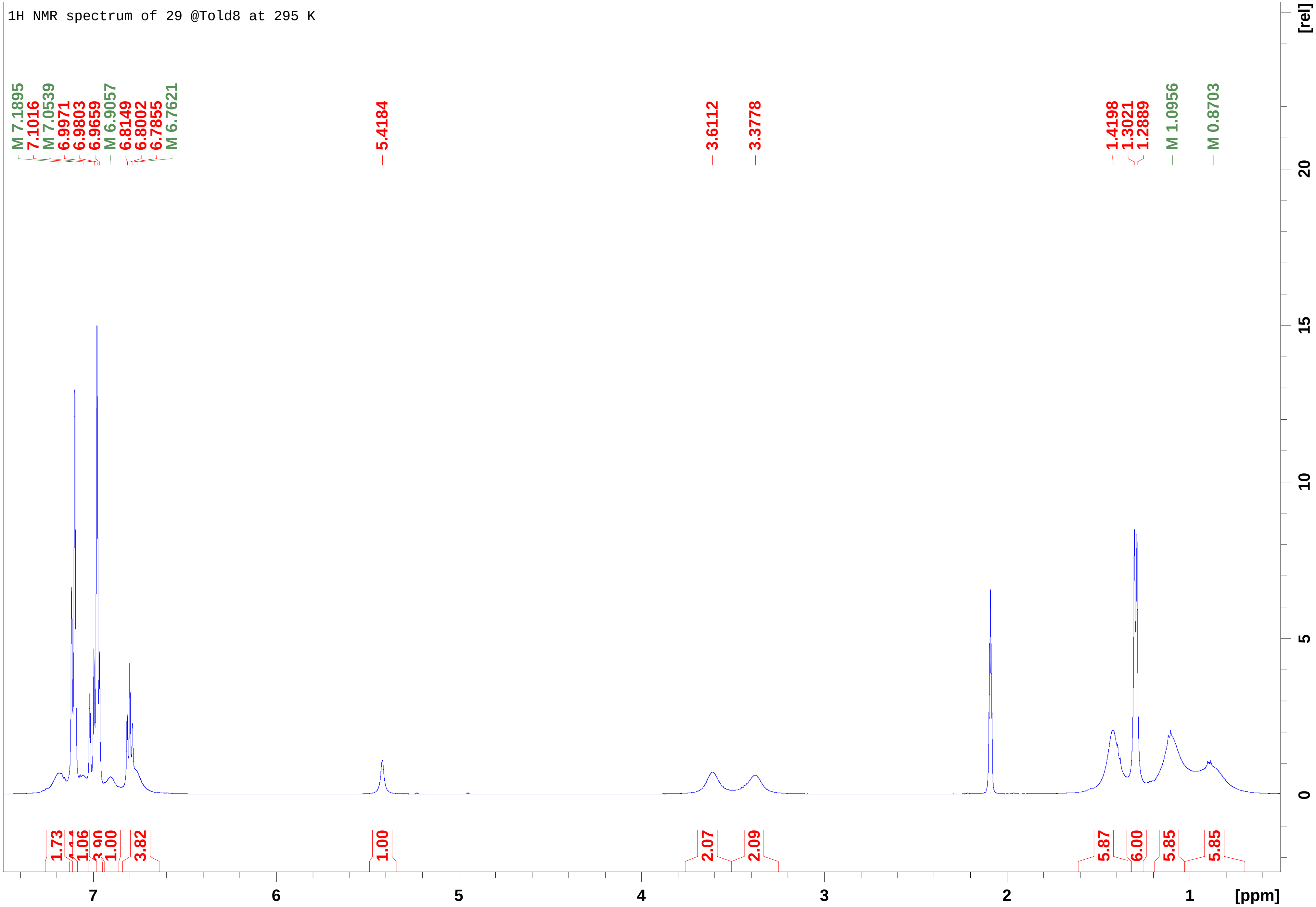

Figure S323. 1H NMR spectrum of 29 in Tol-d8 at 295 K

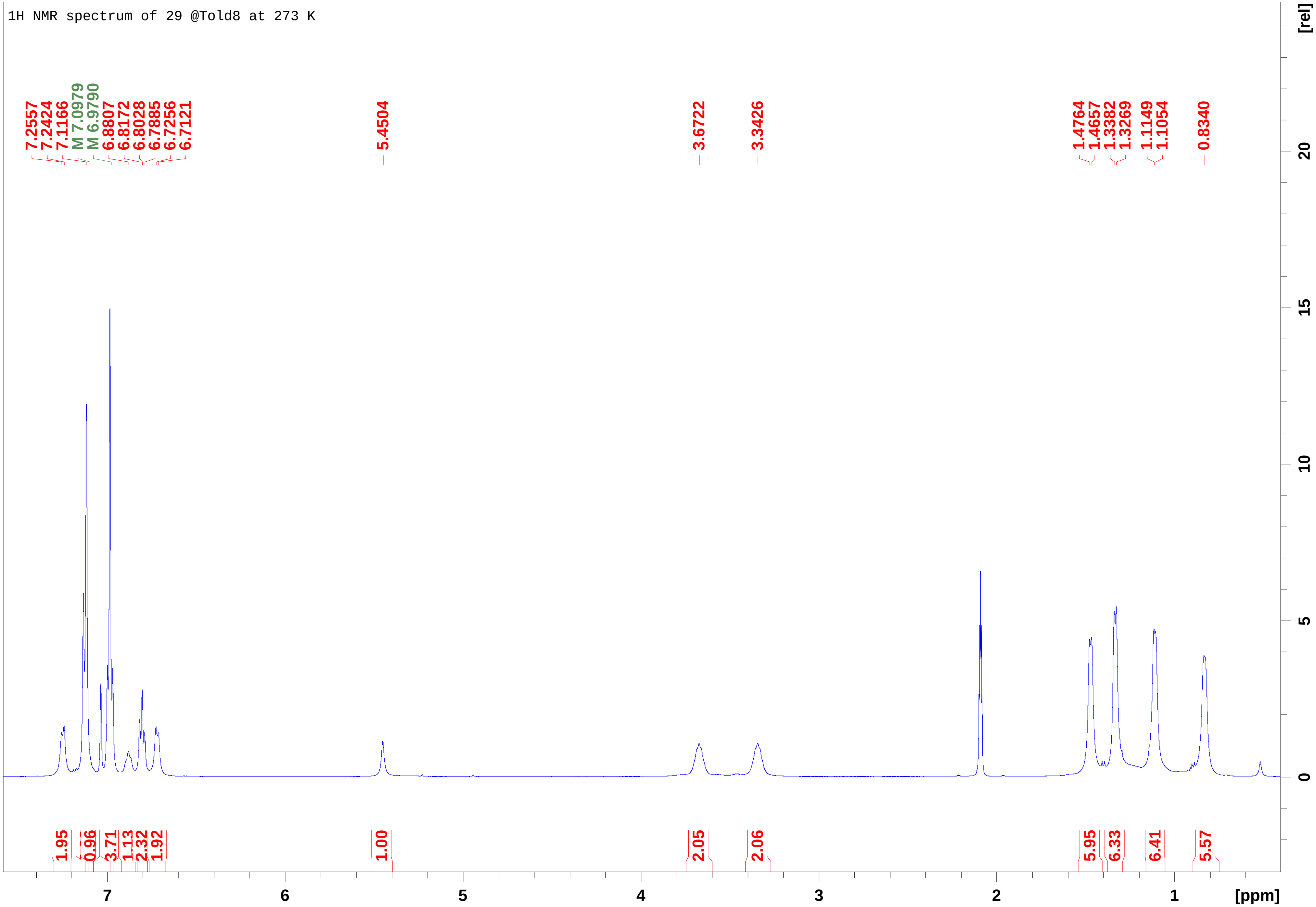

Figure S324. 1H NMR spectrum of 29 in Tol-d8 at 273 K

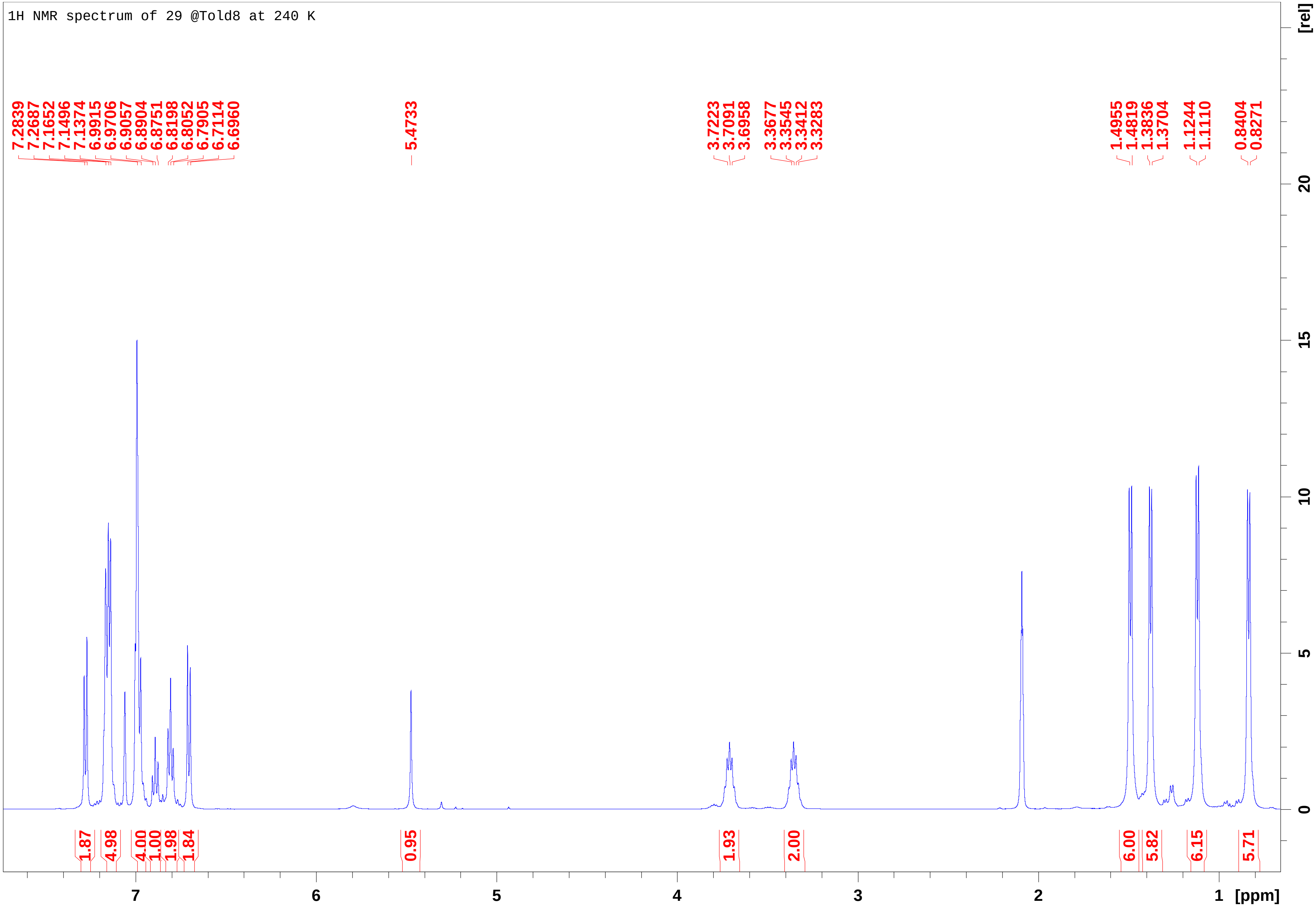

Figure S325. 1H NMR spectrum of 29 in Tol-d8 at 240 K

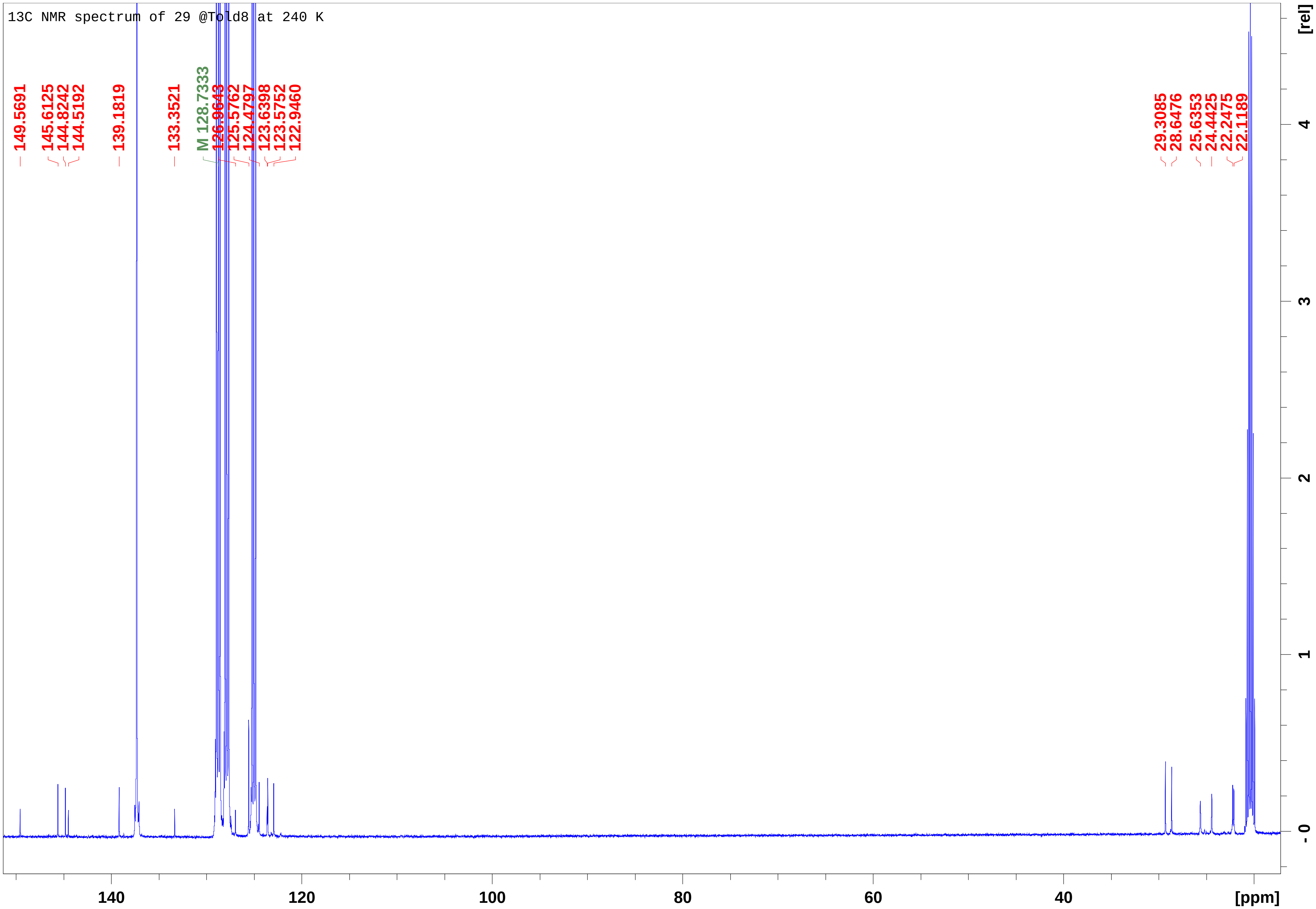

Figure S326. 13C NMR spectrum of 29 in Tol-d8 at 240 K

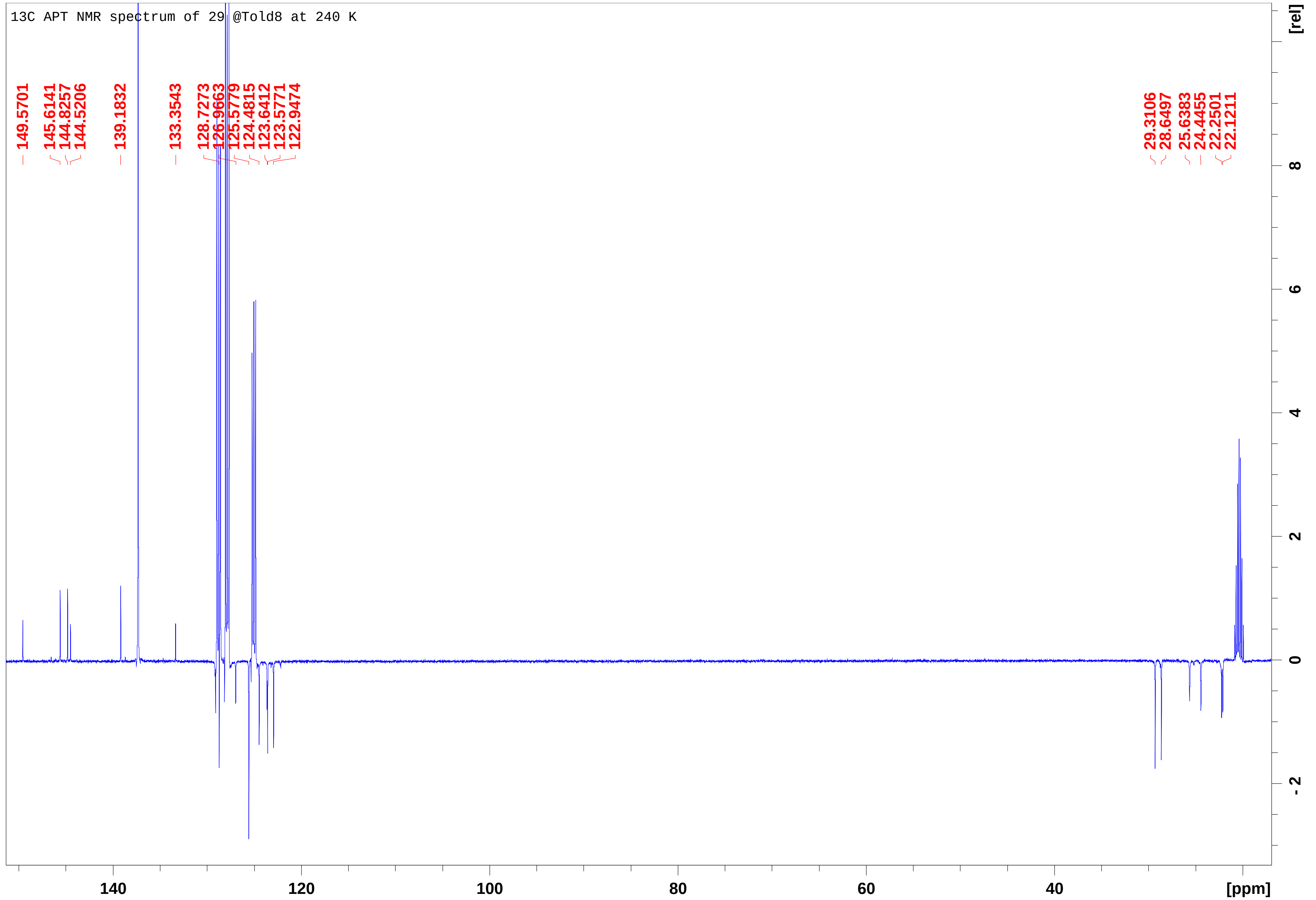

Figure S327. 13C APT NMR spectrum of 29 in Tol-d8 at 240 K

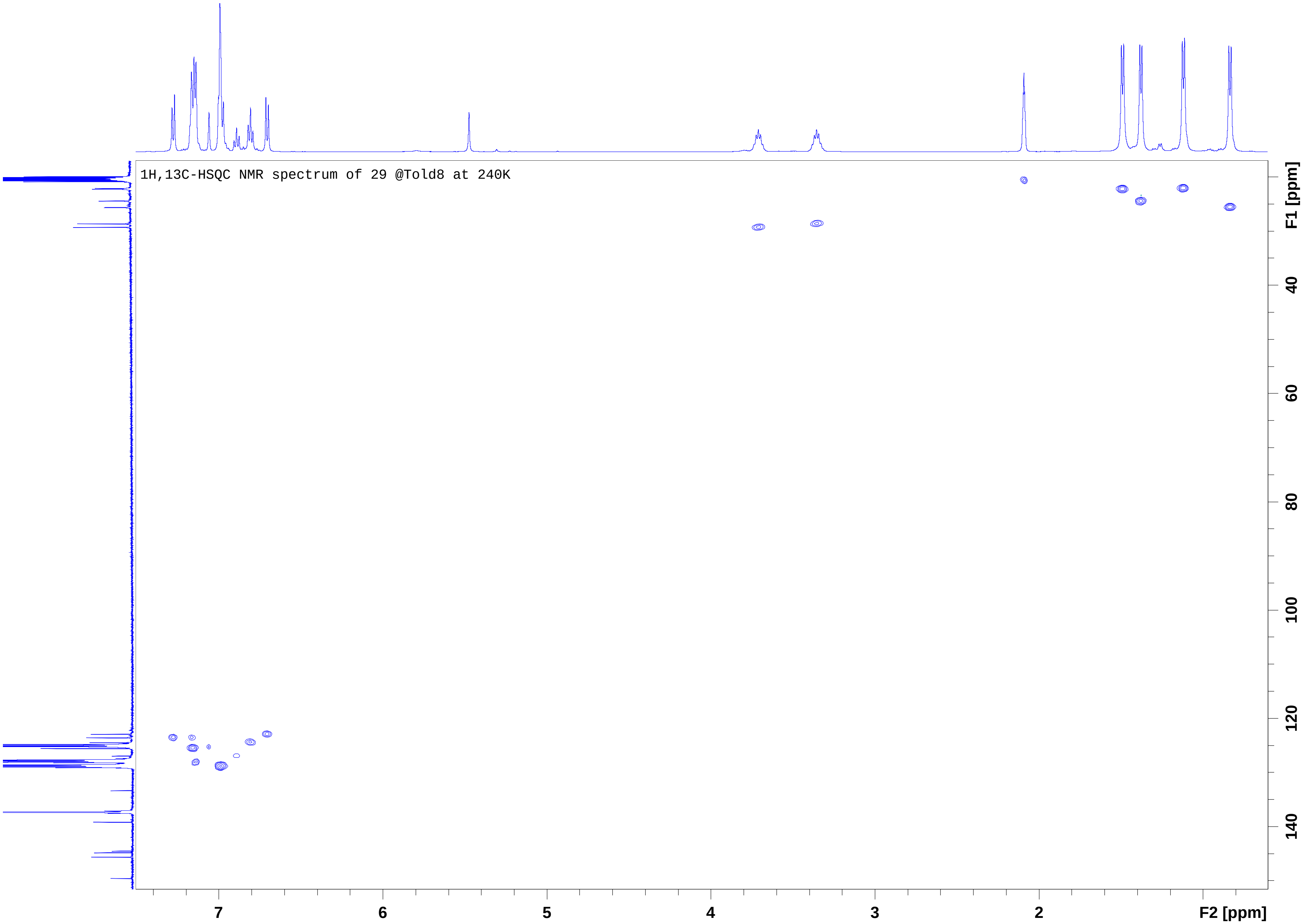

Figure S328. 1H,13C-HSQC NMR spectrum of 29 in Tol-d8 at 240 K

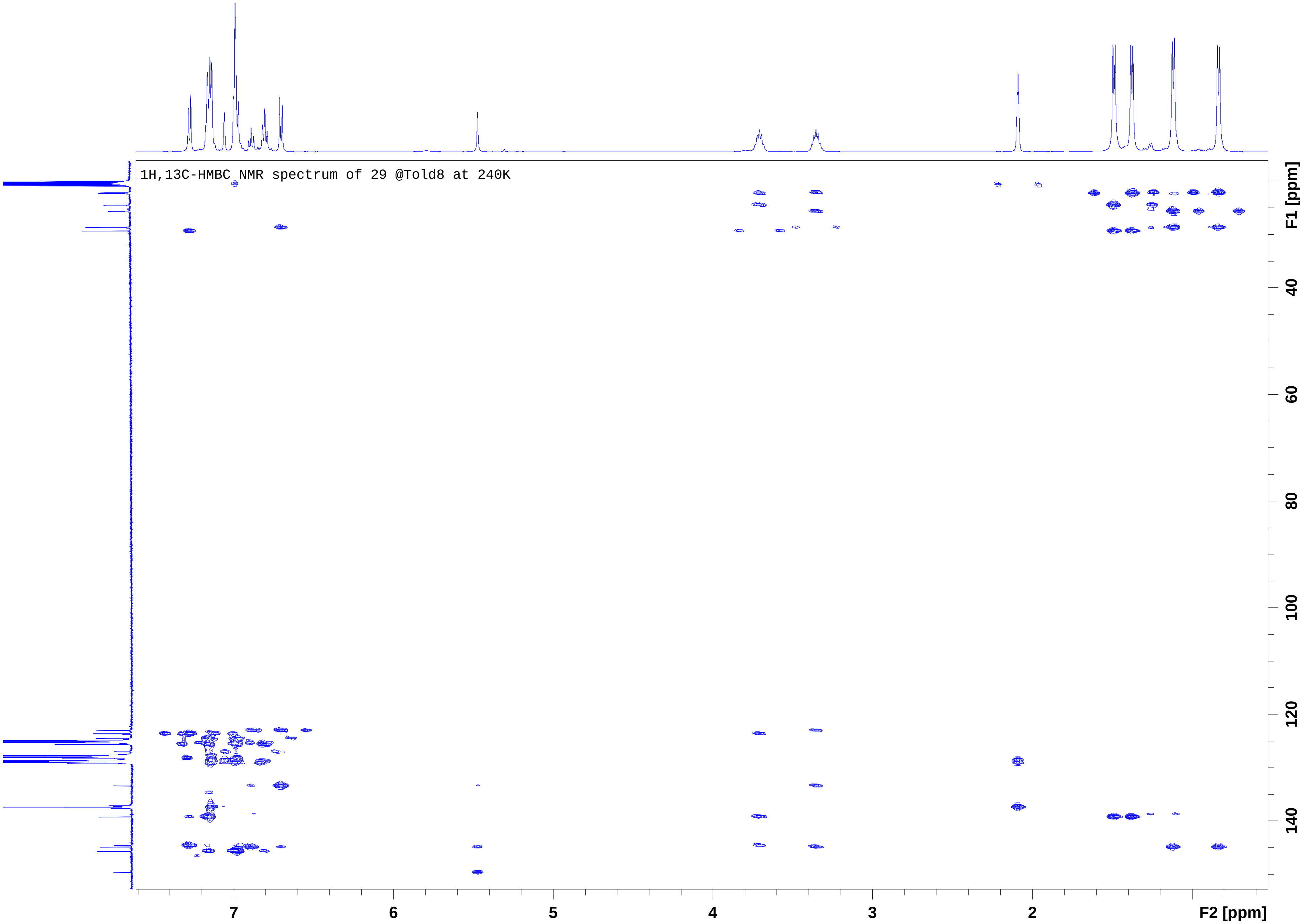

Figure S329. 1H,13C-HMBC NMR spectrum of 29 in Tol-d8 at 240 K
